# Copper-Mediated Late-Stage Radical Trifluoromethylation of Pyrazole-Type Scaffolds

**DOI:** 10.1021/acsomega.5c12212

**Published:** 2026-02-04

**Authors:** Lucie S. Eisen, Jan H. Griwatz, Sven Ruf, María Méndez, Enrique Gomez-Bengoa

**Affiliations:** † Department of Organic Chemistry I, Faculty of Chemistry, University of the Basque Country, Manuel Lardizabal 3, 20080 San Sebastián, Spain; ‡ Sanofi R&D, Integrated Drug Discovery, Industriepark Höchst, Building G838, 65926 Frankfurt am Main, Germany

## Abstract

Trifluoromethylated pyrazoles and their derivatives are of great interest due to their biological and pharmacological relevance. However, efficient methods for their preparation via late-stage trifluoromethylation strategies remain limited. Herein, we report a practical and efficient method for the direct trifluoromethylation of pyrazoles using in situ generated CF_3_ radicals and inexpensive copper­(II) salts as catalysts at room temperature. The method exhibits excellent functional group tolerance. DFT studies support the observed reactivity trends and provide insight into the electronic effects governing CF_3_ radical addition. This strategy holds promise for the generation of highly substituted pyrazoles of interest in biomedical and crop-science applications.

## Graphical abstract

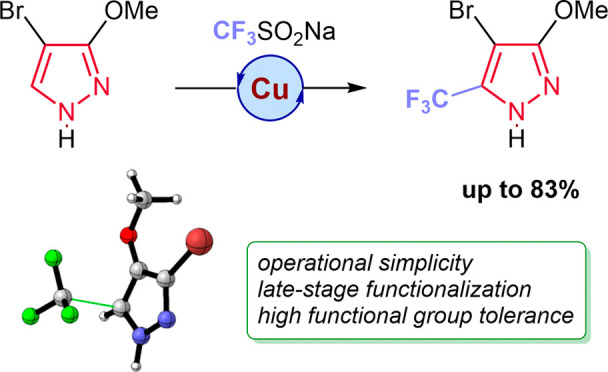

## Introduction

Nitrogen-containing heterocycles have long held a central role in medicinal and agrochemical research, with pyrazoles standing out due to their broad spectrum of biological activities and synthetic adaptability. The distinctive electronic and structural characteristics of the pyrazole ring system render it an essential scaffold in modern drug design.^1^

A particularly effective strategy to further modulate the bioactivity and physicochemical properties of heterocyclic compounds involves the introduction of fluorinated substituents, particularly the trifluoromethyl (CF_3_) group. The CF_3_ moiety exhibits high electronegativity and lipophilicity, which can markedly influence a compound’s metabolic stability, membrane permeability, and target binding affinity. Consequently, its incorporation often improves key physicochemical parameters such as lipophilicity, influencing bioavailability, and the ability to cross the blood–brain barrier.^2–4^ These properties have been harnessed to improve both pharmacokinetic and pharmacodynamic profiles across diverse classes of biologically active compounds, encompassing pharmaceuticals, agrochemicals, and material science.

Given that the pyrazole scaffold and the CF_3_-substituent each play key, independent roles, it is unsurprising that many CF_3_-substituted pyrazole derivatives demonstrate a wide range of pharmacological activities.^5^ These include anti-inflammatory, antimicrobial, and anticancer effects. Clinically relevant examples include marketed drugs such as celecoxib,^6^ a selective COX-2 inhibitor; razaxaban,^7^ a factor Xa inhibitor; and the antitumoral SC-560.^8^ In the agrochemical sector, penthiopyrad^9^ exhibits potent fungicidal activity against foliar and soilborne pathogens, while compound A^10^ demonstrates insecticidal efficacy against *Spodoptera littoralis*, and compound B^11^ shows promise as an acaricide (Figure 1).

**Figure 1:**
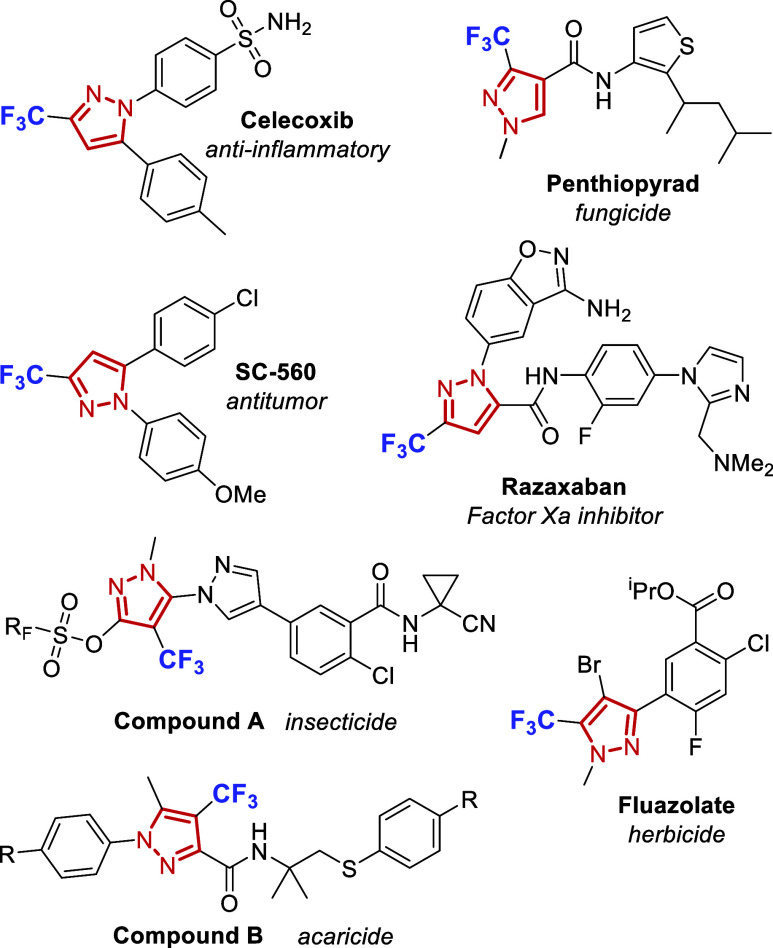
Trifluoromethylated pyrazoles in drug discovery and agrochemicals. Marketed drugs: Celecoxib (Pfizer, 1998), Razaxaban (BMS, 2019). Research tool: SC-560 (selective COX-1 inhibitor). Agrochemicals: Penthiopyrad (Mitsui Chemicals, 2000), Compound A (Syngenta, 2018), Compound B (Sumitomo Chemical, 2013).

These examples collectively underscore the broad utility of CF_3_-pyrazole derivatives across therapeutic and agricultural domains. Given this widespread application, efficient methods for accessing CF_3_-pyrazoles are of high value to both medicinal and agrochemical research. However, despite their significance, *no general and reliable method for the direct trifluoromethylation of pyrazoles has been established to date*.^12^ Current methods typically rely on prefunctionalized building blocks, limiting their practical utility in late-stage diversification strategies. This stands in contrast to the numerous well-documented strategies for the trifluoromethylation of other aromatic systems, particularly heterocycles.^13,14^ The present study aims to address this gap by developing a practical and broadly applicable approach for the direct installation of CF_3_ onto pyrazole scaffolds (Scheme 1).

**Scheme 1:**
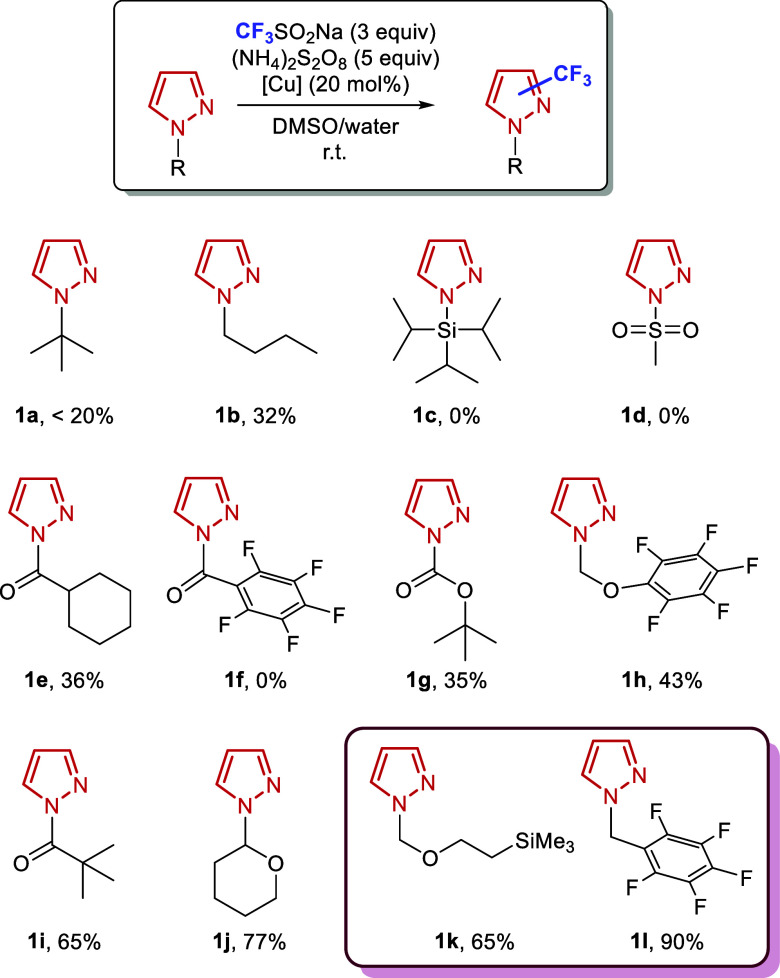
Evaluation of Pyrazole *N*-Protecting Groups in the Direct Trifluoromethylation Reaction. Yields were Determined by NMR Using an Internal Standard

Historically, the synthesis of CF_3_-pyrazoles has predominantly relied on [3 + 2] cycloaddition reactions between trifluoromethylated building blocks as C_3_ synthons and hydrazines as N_2_ counterparts.^15^ A broad range of C_3_ units has been employed, including 1,3-diketones (Figure 2a),^16^ enynes,^17^ enaminones,^18^ acetylenic imines,^19^ acetylenic nitriles,^20^ alkoxyalkenones,^21^ β-ketoesters,^22^ and ketene dithioacetals,^23^ which often afford good yields, but frequently suffer from poor regioselectivity. Notably, Nenajdenko and co-workers reported the use of CF_3_-ynones in a silver triflate catalyzed reaction (Figure 2b).^24^

**Figure 2:**
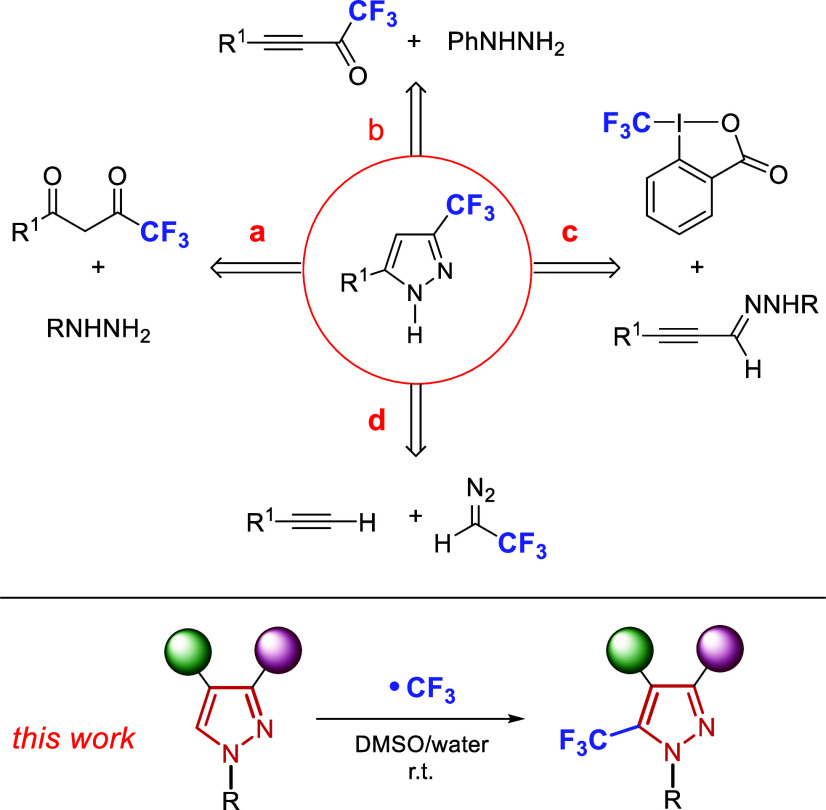
Previous work for the synthesis of CF_3_-pyrazoles and present proposal.

Alternative strategies involve other types of cycloadditions, such as reactions of alkynyl hydrazones with diverse CF_3_ synthons, including Togni reagents (Figure 2c),^25^ or TMSCF_3_.^26^ In addition, CF_3_-diazoethane has served as a valuable N–N synthon in cyclizations with terminal alkynes (Figure 2d).^27^ Finally, other reported cycloadditions include sydnones with electrophilic trifluoropropynes,^28^ bromo-trifluoropropenes,^29^ and [3 + 2] cyclizations of fluorinated nitrile imines with chalcones.^30^

Collectively, the aforementioned methodologies represent a valuable synthetic toolbox for the construction of trifluoromethyl-pyrazoles, offering strategic diversity and practical utility. However, many of these approaches suffer from significant limitations, including the requirement for elaborate substrate preparation and narrow substrate scope. These factors have hindered the development of general, scalable, and operationally simple methods. Consequently, a method allowing the direct trifluoromethylation of pyrazoles remains highly desirable.

In contrast to the limited progress specifically related to pyrazoles, direct C–H trifluoromethylation of various heterocycles has seen significant development in recent years.^31^ Among the most prominent approaches are radical C–H trifluoromethylation methods, which utilize bench-stable CF_3_ radical sources and have attracted considerable attention due to their operational simplicity and high functional group tolerance. Reagents such as sodium trifluoromethanesulfinate (Langlois reagent), Togni reagents, and trifluoromethyl iodide have been successfully employed in the late-stage functionalization of electron-rich and electron-deficient heteroaromatic scaffolds.^13b,31,32^ However, despite the broad applicability, their extension to pyrazole derivatives remains unexplored, with only limited success reported to date.^12a^ ^,^ ^33^

To address this significant gap in synthetic methodology, and inspired by previous reports on copper-mediated trifluoromethylations of unsaturated systems, we have developed a direct and efficient one-step method for the trifluoromethylation of pyrazoles using the Langlois reagent. This strategy enables the late-stage functionalization of structurally diverse pyrazoles, offering a practical route to access CF_3_-substituted analogues under operationally simple conditions.

## Results and Discussion

Based on preliminary results (see Supporting Information for details), we anticipated that the free NH group of the pyrazole could pose challenges under radical trifluoromethylation conditions. Indeed, initial attempts to directly functionalize 1*H*-pyrazole were unsuccessful, with analysis of the reaction mixture suggesting either incomplete reaction or formation of multiple products. We therefore systematically investigated the effect of various protecting groups on the pyrazole N–H moiety.

Building upon earlier studies of metal-mediated radical addition to heteroaromatic systems,^31,34^ our investigation began by evaluating the addition of the CF_3_ radical in the presence of catalytic amounts of copper salts, stoichiometric Langlois reagent (CF_3_SO_2_Na), and (NH_4_)_2_S_2_O_8_ as the oxidant at room temperature.

As illustrated in Scheme 1, substrates bearing alkyl (**1a**,**b**), silyl (**1c**), or sulfonyl (**1d**) protecting groups exhibited minimal to no conversion. Similarly, amide-protected pyrazoles, particularly those bearing strongly electron-withdrawing groups such as the pentafluorobenzoyl moiety (**1f**), afforded no product formation. These observations suggest that electron-rich substrates are essential for achieving efficient trifluoromethylation under these conditions. Notably, substrate **1j** demonstrated high reactivity; however, the corresponding adduct was unstable, and partial deprotection of the pyrazole ring was observed either during the reaction or upon isolation. Partial deprotection was also observed with **1i**. Ultimately, useful yields of the desired products were obtained when more robust protecting groups were employed, such as 2-(trimethylsilyl)­ethoxymethyl (SEM) (**1k**, 65%) and −CH_2_C_6_F_5_ (**1l**, 90%).

We next analyzed the optimal conditions for the reaction in the presence of these last two *N*-protecting groups: SEM and pentafluorobenzyl. In all cases, the presence of these groups appeared to maximize regioselectivity at C5, with lower amounts of C3 and only traces of or no detectable C4 functionalization. As initially observed, copper salts proved optimal for the completion of the reaction. Other metal salts, including those of silver, iron, manganese, gold, palladium, nickel, and cobalt were extensively tested but did not afford the desired products in appreciable amounts (see SI).

In the case of the pentafluorobenzyl group, copper­(II) salts such as copper nitrate (entry 1), copper acetate (entry 2), and copper sulfate (entry 5) provided comparable yields and selectivity (approximately 90% yield, 2:1 C3:C5), with copper sulfate affording the highest overall yield. A marked decrease in yield was observed in the presence of copper-coordinating species such as 1,10-phenanthroline (entry 3) or ethylenediamine (entry 4), and the reaction efficiency dropped significantly in the absence copper salts (entry 6). The range of copper salts tested was expanded for the SEM group, though no dramatic differences in performance were observed among them (entries 8–13). Nevertheless, copper triflate was identified as the optimal salt for this substrate, offering the highest efficiency (entry 12, 42%:23% yield for C5:C3). Cu­(I) salts such as copper­(I) chloride and copper­(I) acetate were also evaluated, yielding inferior results (entries 14–15).

During the initial solvent screening, the reaction proceeded only in polar media. For the −CH_2_C_6_F_5_ group, the reaction in acetonitrile remained competitive (entry 7). However, in the case of the SEM-protected substrate, solvents such as dry DMSO (entry 16), acetonitrile (entry 17), or DMF (entry 18), did not afford competitive yields (all below 30%). The addition of varying amounts of water to DMSO significantly enhanced reaction efficiency, with the best results obtained using a DMSO/water ratio of 5:2, as illustrated in the examples in Table 1.

**Table 1:**
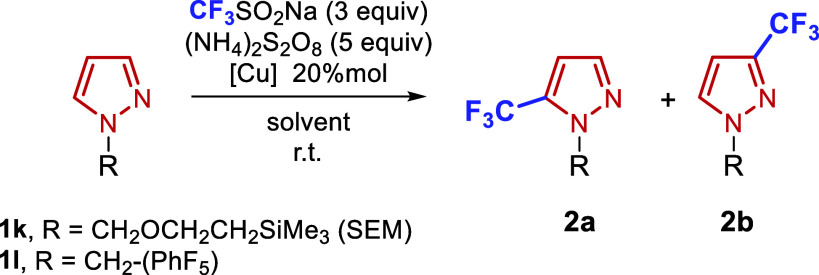
Optimization of the Reaction Conditions for Pentafluorobenzyl- and SEM-Protected Pyrazoles

entry	R	Cu source	solvent	yield **2a**:**2b** ^a^
1	C_6_F_5_CH_2_–	Cu(NO_2_)_2_·3H_2_O	DMSO/water	59:27
2	C_6_F_5_CH_2_–	Cu(OAc)_2_	DMSO/water	54:27
3^b^ ^,^ ^d^	C_6_F_5_CH_2_–	Cu(OAc)_2_	DMSO/water	32:19
4^c^ ^,^ ^d^	C_6_F_5_CH_2_–	Cu(OAc)_2_	DMSO/water	22:19
**5**	**C**_**6**_ **F**_**5**_ **CH**_**2**_ **–**	**CuSO**_**4**_ **·5H**_**2**_ **O**	**DMSO/water**	**65:28**
6^d^	C_6_F_5_CH_2_–	None	DMSO/water	6:15
7^d^	C_6_F_5_CH_2_–	Cu(OAc)_2_	acetonitrile	26:18
8	SEM–	Cu(OAc)_2_	DMSO/water	38:15
9	SEM–	Cu(NO_2_)_2_·3H_2_O	DMSO/water	37:16
10	SEM–	CuF_2_	DMSO/water	36:20
11	SEM–	CuCl_2_	DMSO/water	34:n.d.
12	SEM–	Cu(OTf)_2_	DMSO/water	42:23
13	SEM–	CuSO_4_·5H_2_O	DMSO/water	25:11
14	SEM–	CuCl	DMSO/water	33:30
15	SEM–	CuOAc	DMSO/water	27:13
16	SEM–	Cu(OAc)_2_	DMSO	20:10
17	SEM–	Cu(OAc)_2_	acetonitrile	12:6
18	SEM–	Cu(OAc)_2_	DMF	2:4

a NMR yields using dibromomethane as the internal standard.

b In the presence of 1,10-phenanthroline.

c In the presence of ethylenediamine.

d Reactions carried out at 75 °C.

Despite showing somewhat lower yields, the SEM group was considered more convenient than the perfluorobenzyl group, as it can generally be cleaved more efficiently to regenerate the free NH functionality. Therefore, we subsequently explored the reactivity of monosubstituted pyrazole substrates bearing a SEM protecting group under the optimized reaction conditions, and the results are summarized in Scheme 2. Substrates bearing 4-methoxy and 4-methyl substituents were selected due to their high reactivity in preliminary experiments with the SEM protecting group. Notably, the SEM-protected derivatives displayed low C5:C3 regioselectivity, with product distributions of 61:12 for **3a** and 34:13 for **3b**. Similarly, the perfluorobenzyl-protected pyrazole afforded the C5 and C3 products in 42% and 19% isolated yield, respectively (**3c**), which differs considerably from the NMR yield due to the somewhat challenging separation of both regioisomers. For comparison, we also decided to retest the reaction conditions with *N*-unprotected pyrazoles, which could then directly be used in further functionalization reactions. To our delight, the reactions proceeded efficiently with substrates bearing 4-methoxy (**4a**) and 4-methyl (**4b**) substituents, the latter corresponding to the alcohol dehydrogenase inhibitor fomepizole. These two substrates demonstrated enhanced reactivity compared to their SEM-protected counterparts, resulting in readily separable mono- and difunctionalized adducts, which were not observed in the cases of **3a** and **3b**.

**Scheme 2:**
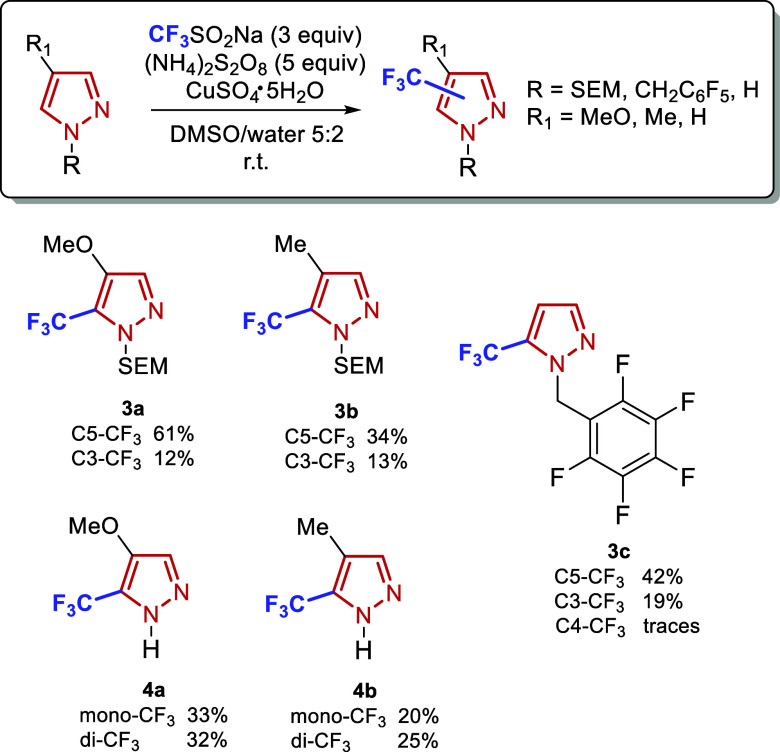
Trifluoromethylation of *N*-Substituted and NH Mono-Substituted Pyrazoles. Yields Correspond to Isolated Products; Average of Three Runs

At this stage, we hypothesized that the high reactivity of *N*-unprotected pyrazoles could be leveraged for the functionalization of less reactive substrates, particularly those bearing electron-withdrawing substituents. In such cases, it was anticipated that difunctionalization would be mitigated or suppressed. Indeed, modest to good yields were obtained for a variety of functional groups, including ester (**5c**), acetyl (**5d**), nitro (**5e**), carboxylic acid (**5f**), and fluoro (**5j**) substituents. The broad functional group compatibility became especially evident with more structurally complex substrates such as **5i**, which contains a radical-sensitive benzylic-type tertiary C–H bond, and C–H bonds adjacent to an amine group. Notably, **5i** was obtained with complete regioselectivity and in a synthetically useful yield of 56%. The striking contrast in regioselectivity between compounds **5g**–**5h** and **5i** can be related to a previous study on the influence of π- and non-π-conjugating substituents on the reactivity of pyridine substrates.^31^ These findings suggest that the presence of a π-conjugating electron-withdrawing substituent (such as the acetyl and ester groups found in **5g** and **5h** respectively) can activate the adjacent position for radical attack by stabilizing the resulting radical intermediate, thereby providing a plausible explanation for why the normally unreactive C4 becomes activated in those compounds. While regioselectivity was low in the case of **5g** and **5h**, the obtained regioisomers could be separated by regular column chromatography, providing access to two distinct potentially valuable products.

The observation that even some deactivated substrates underwent varying degrees of difunctionalization further highlights the inherently high reactivity of NH-pyrazoles. For example, in the case of **5d** (acetyl-substituted), the second CF_3_ group was introduced into a pyrazole already bearing both an acetyl and a CF_3_ group.

This finding suggested the potential for general functionalization of more complex, disubstituted systems, even in the presence of electron-withdrawing groups.

Subsequently, the optimized reaction conditions were applied to a broader set of disubstituted substrates featuring diverse functional groups (Scheme 4). The reaction generally proceeded with good efficiency to afford single adducts, with regioselectivity and overfunctionalization not being an issue—an advantage from both a practical and mechanistic standpoint. Notably, the reaction conditions demonstrated excellent tolerance toward a wide array of functional groups, including halides, various carbonyl functionalities (carboxylic acids, esters, ketones), nitriles, nitro, trifluoromethyl, fluorine, and several alkyl groups. Substrates bearing tertiary C–H positions, such as isopropyl or cyclopropyl moieties, also reacted effectively in different combinations.

**Scheme 3:**
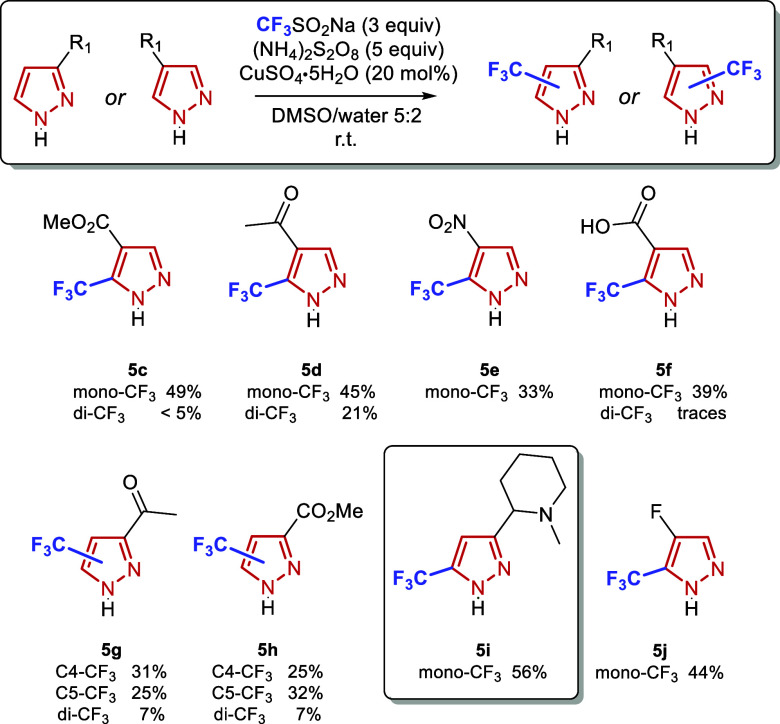
Trifluoromethylation of Mono-Substituted Unprotected Pyrazoles. Yields Correspond to Isolated Products; Average of Three Runs

**Scheme 4:**
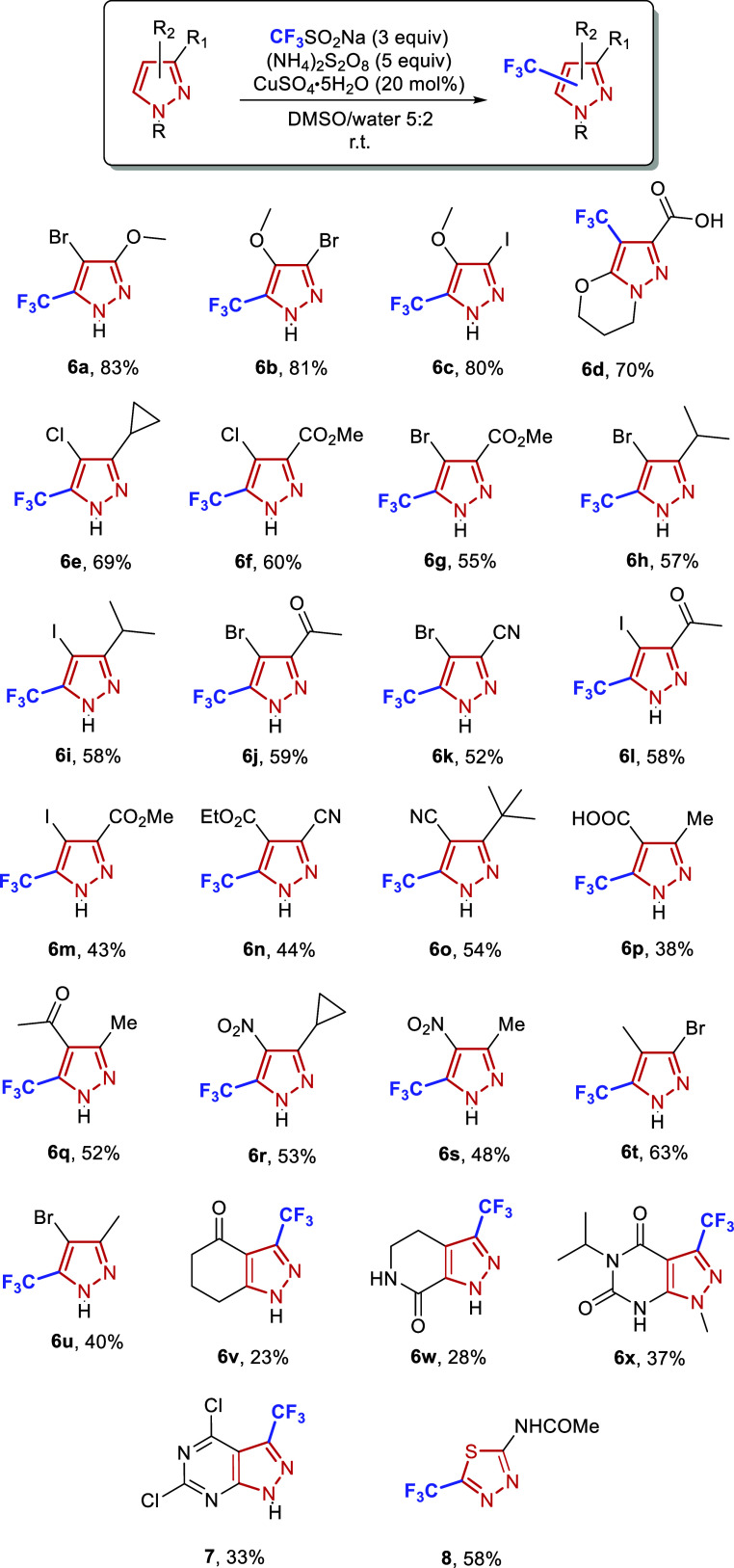
Trifluoromethylation of Di-substituted Pyrazoles. Yields Correspond to Isolated Products; Average of Three Runs

In general, the reactivity trend followed electronic effects: substrates with electron-donating groups displayed the highest yields (**6a** 83%, **6b** 81%, **6c** 80%), electron-neutral groups gave moderate yields, while electron-withdrawing groups afforded lower conversions (**6n** 44%, **6s** 48%). Significantly, the present methodology reliably furnishes polysubstituted pyrazole building blocks with up to three orthogonally functionalizable handles in synthetically useful yields, highlighting its broad functional group tolerance and strong potential for downstream diversification toward pharmaceutically or agrochemically relevant compounds. The successful derivatization of a patented herbicidal compound to form **6x** further highlights the practical utility of the method.^35^

Based on our observations, we have formulated a few guidelines to predict the reactivity and regioselectivity of pyrazole substrates.

- C4 does not react significantly, unless activated by the presence of a π-conjugating electron-withdrawing substituent on C3 or C5.
- On *N*-substituted pyrazoles, position 5 is favored over position 3 with moderate to good regioselectivity.
- *N*-substituted pyrazoles, even when electronically activated by electron-donating substituents, are not subject to disubstitution.

The reaction also presents certain limitations. Among the most evident are substrates containing aromatic rings or specific heterocycles, which can undergo undesired trifluoromethylation under the reaction conditions (see Supporting Information for examples). Similarly, functional groups such as alcohols and amines (with the exception of tertiary amines, as seen for **5i**) are not well tolerated. Nonetheless, some substrates that are generally considered challenging to functionalize were found to undergo the reaction, with varying yields (**6d** 70%, **6v** 23%, **6w** 28%, **6x** 37%). Notably, two additional pyrazole-derived heterocycles reacted efficiently with the CF_3_ radical to afford the corresponding adducts in 33% (**7**) and 58% (**8**) yield, respectively.

In order to gain insight into the reaction mechanism, a series of control experiments were conducted. In the absence of copper salts, the reaction still proceeded—albeit sluggishly and without difunctionalization—only for the more reactive substrates, such as methoxy-substituted derivatives. This indicates that copper is not strictly required for the reaction to occur, yet it dramatically enhances the overall efficiency of the process. In this context, it is noteworthy that similar reactions using other metals (Ni, Co, Ag, Pd, Au, Mn) yielded no positive results, and no alternative metal-based system matched copper’s performance.

To confirm the radical nature of the reaction, we performed a control experiment on 4-methoxypyrazole with TEMPO (2,2,6,6-tetramethylpiperidin-1-yl oxyl) as a radical scavenger (3 equiv). The reaction was completely suppressed in the presence of TEMPO, supporting a radical pathway.

To elucidate the factors governing reactivity and regioselectivity in this trifluoromethylation reaction, we performed DFT calculations focusing on three key aspects: activation barriers for CF_3_ radical addition, oxidation potentials of radical intermediates, and the role of copper coordination.^36^ The calculated activation barriers for CF_3_ radical addition to various pyrazoles aligned with the trends observed in the experimental yields (Figure 3A). Notably, most of the barriers were found to be below 15 kcal/mol, suggesting that the radical addition step is generally feasible. This result is consistent with the broad functional group tolerance observed experimentally and helps explain the differences in yield between electron-rich and electron-deficient substrates. For example, the lowest computed activation energy for CF_3_ radical attack corresponds to 4-methoxypyrazole (Δ*G* ^‡^ = 8.9 kcal/mol), and this substitution pattern affords some of the highest yields (>80% for **6b** and **6c** in Scheme 4, **3a** in Scheme 2).^37^ In contrast, the highest activation energy corresponds to 4-nitropyrazole (Δ*G* ^‡^ = 12.0 kcal/mol), consistent with the lower yields obtained for this compound (**5e**, 33%, Scheme 3), and generally for strongly electron-withdrawing groups. This behavior can be rationalized by the nucleophilic character of the pyrazole ring, which is enhanced by electron-donating substituents. Nonetheless, the low activation energies computed for all substrates are somewhat surprising and can be attributed to the central position of the CF_3_ radical in terms of radical polarity,^38^ and its high reactivity, as previously described in both experimental and theoretical studies.^39^

**Figure 3:**
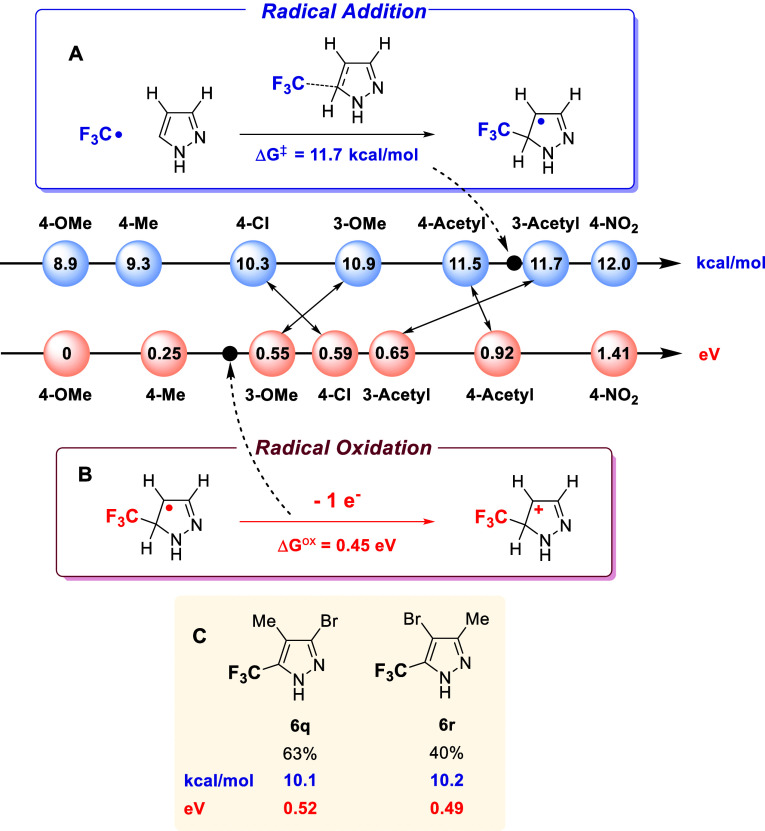
DFT calculated trends in the activation Gibbs free energy for CF_3_ radical addition (in blue) and the Gibbs energy for intermediate radical oxidation (in red).

An alternative explanation for the relatively lower reactivity of electron-poor pyrazoles involves the oxidation of the radical intermediate formed at position 4, after CF_3_ addition (Figure 3B). The presence of electron-donating groups at C4 would be expected to facilitate this oxidation, thus promoting the overall transformation. To support this hypothesis, the oxidation potentials of representative substrates were calculated, relative to 4-methoxypyrazole (set as Δ*G* ^ox^ = 0 eV), revealing a clear correlation with the electron density of the ring, particularly at C4. However, in these cases, the energy differences are too large to fully account for the observed reactivity. A striking example is 4-nitropyrazole, which shows an oxidation potential that is 1.41 eV higher than the 4-methoxy analogue. Furthermore, the comparison between the slightly more reactive 4-acetyl (**5d**) and its isomeric 3-acetyl (**5g**, Scheme 3) derivatives reveals only a marginally faster reaction for the former (11.5 vs 11.7 kcal/mol), while the oxidation of the radical at 4-acetylpyrazole appears to be significantly more difficult (0.92 vs 0.65 eV). Thus, the experimental reactivity trend is better explained by the pattern computed for the CF_3_ radical addition energies. The comparison between the more reactive **6t** and **6u** supports the same conclusion (Figure 3C), since the radical attack is easier at **6t**, while oxidation would favor the less reactive **6u**.

To examine the role of copper salts, we modeled their coordination to the nitrogen atom of the pyrazole ring during the CF_3_ addition step. Surprisingly, this coordination led to a notable increase in the activation barrier, suggesting that copper does not facilitate the radical attack. In fact, copper coordination appears to reduce the electron density of the substrate, which could negatively impact reactivity.

These findings point to a possible role of copper as the oxidizing species in the reaction, facilitating the oxidation of the more reluctant substrates, such as 4-nitropyrazole. Notably, we confirmed that only highly electron-rich substrates, such as 4-methoxypyrazole, undergo addition—although sluggishly—in the absence of copper, while electron-poor analogues, such as 4-nitropyrazole, do not react at all.

In summary, this work leverages the reactivity of in situ generated CF_3_ radicals and the catalytic activity of inexpensive copper­(II) salts to access a broad array of CF_3_-substituted pyrazoles. The reaction proceeds at room temperature, it is compatible with both air and moisture and does not require sophisticated equipment such as a photoreactor. A wide variety of functional groups are well tolerated, including methoxy, nitro, alkyl, cyclopropyl, ketones, esters, amides, halogens, nitriles, carboxylic acids, and tertiary amines. Both *N*-protected and unprotected pyrazoles are suitable substrates. Notably, this method addresses a key gap in the direct trifluoromethylation of pyrazoles, avoiding the need for prefunctionalized substrates or indirect methods. Complementary DFT studies were employed to predict and rationalize the reactivity of different pyrazole derivatives toward CF_3_ radical addition.

## Methods

### General Procedure for the Trifluoromethylation of Pyrazoles

A reaction vial was charged with the pyrazole substrate (0.50 mmol, 1.0 equiv), CF_3_SO_2_Na (234 mg, 1.50 mmol, 3.0 equiv), and CuSO_4_·5H_2_O (25 mg, 0.10 mmol, 0.20 equiv). DMSO (2 mL) and water (0.8 mL) were added, and the mixture was stirred briefly to ensure dissolution. Then, (NH_4_)_2_S_2_O_8_ (570 mg, 2.50 mmol, 5.0 equiv) was added in portions at room temperature. The reaction mixture was stirred at room temperature for 16 h. Reaction completion was monitored by either thin layer chromatography (TLC) or liquid chromatography—mass spectrometry (LC–MS). Upon completion, the reaction mixture was diluted with saturated aqueous NaHCO_3_ and ethyl acetate. The aqueous layer was extracted three times with ethyl acetate. The combined organic layers were washed once with water, once with brine, dried over MgSO_4_, filtered, and concentrated under reduced pressure. The crude material was purified by silica gel column chromatography using an appropriate solvent system (see Supporting Information for details), to afford the trifluoromethyl-pyrazole adduct.

## Supplementary Material



## Notes

General information on experimental methods and equipment; Tables S1 to S6: detailed optimization process; synthetic procedure and characterization of all compounds; examples of unsuccessful reactions showcasing the methodology’s limitations; Figure S1: thermal ellipsoids for the X-ray crystal structure of **6j**; Table S7: crystallographic data for **6j**; Table S8: fractional atomic coordinates and equivalent isotropic displacement parameters for **6j**; Table S9: anisotropic displacement parameters for **6j**; Table S10: bond lengths for **6j**; Table S11: bond angles for 6j; Table S12: torsion angles for **6j**; Table S13: hydrogen fractional coordinates and equivalent isotropic displacement parameters for **6j**; Table S14: hydrogen bond information for **6j**; details of density functional theory (DFT) calculations; Figure S2: absolute G energies of the trifluoromethyl radical addition to the pyrazole and activation energy of the transition state; Figure S3: relative oxidation potential of the substituted pyrazole radicals in eV computed as the difference with the oxidation of the reference 4-methoxypyrazole radical; Table S15: energies of the structures studied in the main manuscript; Tables S16 to S55: computed coordinates of starting materials, transition states and adduct radicals; NMR spectra of all structures (PDF)

## References

[1] Fustero S., Sánchez-Roselló M., Barrio P., Simón-Fuentes A. (2011). From 2000
to mid-2010:
A fruitful decade for the synthesis of pyrazoles. Chem. Rev..

[2] Knauber T., Arikan F., Röschenthaler G. V., Gooßen L. J. (2011). Copper-catalyzed trifluoromethylation of aryl iodides
with potassium (trifluoromethyl)­trimethoxyborate. Chem. Eur. J..

[3] Wang J., Sánchez-Roselló M., Aceña J. L., del Pozo C., Sorochinsky A. E., Fustero S., Soloshonok V. A., Liu H. (2014). Fluorine in pharmaceutical
industry: fluorine-containing drugs introduced to the market in the
last decade (2001–2011). Chem. Rev..

[4] Chu L., Qing F.-L. (2014). Oxidative Trifluoromethylation
and
trifluoromethylthiolation reactions using (trifluoromethyl)­trimethylsilane
as a nucleophilic CF_3_ source. Acc.
Chem. Res..

[5] Kaur K., Kumar V., Gupta G. K. (2015). Trifluoromethylpyrazoles
as anti-inflammatory
and antibacterial agents: A review. J. Fluorine
Chem..

[6] Penning T. D., Talley J. J., Bertenshaw S. R., Carter J. S., Collins P. W., Docter S., Graneto M. J., Lee L. F., Malecha J. W., Miyashiro J. M., Rogers R. S., Rogier D. J., Yu S. S., Anderson G. D., Burton E. G., Cogburn J. N., Gregory S. A., Koboldt C. M., Perkins W. E., Seibert K., Veenhuizen A. W., Zhang Y. Y. (1997). Synthesis and biological evaluation of the
1,5-diarylpyrazole class of cyclooxygenase-2 inhibitors: identification
of 4-[5-(4-methylphenyl)-3-(trifluoromethyl)-1*H*-pyrazol-1-yl]­benzenesulfonamide
(SC-58635, celecoxib). J. Med. Chem..

[7] Zhang D., Raghavan N., Chen S.-Y., Zhang H., Quan M., Lecureux L., Patrone L. M., Lam P. Y. S., Bonacorsi S. J., Knabb R. M., Skiles G. L., He K. (2008). Reductive
isoxazole
ring opening of the anticoagulant razaxaban is the major metabolic
clearance pathway in rats and dogs. Drug Metab.
Dispos..

[8] Lee E., Choi M. K., Youk H. J., Kim C. H., Han I. O., Yoo B. C., Lee M. K., Lim S. J. (2006). 5-(4-chlorophenyl)-1-(4-methoxyphenyl)-3-trifluoromethylpyrazole
acts in a reactive oxygen species-dependent manner to suppress human
lung cancer growth. J. Cancer Res. Clin. Oncol..

[9] Culbreath A. K., Brenneman T. B., Kemerait R. C., Hammes G. G. (2009). Effect
of the new pyrazole carboxamide fungicide penthiopyrad on late leaf
spot and stem rot of peanut. Pest Manage. Sci..

[10] Jeanguenta, A. ; El Qacemi, M. ; Stoller, A. ; Bigot, A. ; Gribkov, D. Pesticidally active pyrazole derivatives. WO Patent WO2018234240, December 27th, 2018.

[11] Koyama, K. ; Okada, I. ; Fukuchi, T. ; Kinoshita, Y. N-thioalkyl pyrazole-3-carboxamide derivative and miticide using the same active ingredient. Japanese patent JP2013216634, October 24th, 2013.

[12] Kino T., Nagase Y., Ohtsuka Y., Yamamoto K., Uraguchi D., Tokuhisa K., Yamakawa T. (2010). Trifluoromethylation
of various aromatic compounds by CF_3_I in the presence of
Fe­(II) compound, H_2_O_2_ and dimethylsulfoxide. J. Fluor. Chem..

[13] Charpentier J., Früh N., Togni A. (2015). Electrophilic trifluoromethylation by use of hypervalent iodine reagents. Chem. Rev..

[14] Baishya G., Dutta N. B. (2021). Recent advances
in direct C–H trifluoromethylation
of *N*-heterocycles. ChemistrySelect.

[15] Meyer F. (2016). Trifluoromethyl nitrogen heterocycles:
synthetic aspects and potential biological targets. Chem. Commun..

[16] Kumar V., Aggarwal V., Singh S. P. (2008). Reaction
of hydrazines and hydroxylamine with trifluoromethyl-β-diketones:
synthesis of trifluoromethylpyrazole and isoxazole derivatives. Heterocycles.

[17] Wei L., Ding S., Liu M., Yu Z., Xiao Y. (2021). Synthesis
of trifluoromethylated pyrazolidines, pyrazolines and pyrazoles via
divergent reaction of β-CF_3_-1,3-enynes with hydrazines. Org. Lett..

[18] Ji G., Wang J., Zhang S., Xu Y., Ye Y., Li M., Zhang Y., Wang J. (2014). Synthesis
of 3-trifluoromethylpyrazoles
via trifluoromethylation/cyclization of α,β-alkynic hydrazones
using a hypervalent iodine reagent. Chem. Commun..

[19] Yu H.-B., Huang W.-Y. (1998). A novel precursor for per­(poly)­fluoroalkyl heterocycles
from N-aryl per­(poly)­fluoroalkyl imidoyl iodides. J. Fluorine Chem..

[20] Fabron J., Pastor R., Cambon A. (1987). Synthèse
régiospecifique
et identification spectrale de nouveaux amino pyrazoles F-alkyl substitués. J. Fluorine Chem..

[21] Song L.-P., Chu Q.-L., Zhu S.-Z. (2001). Synthesis
of fluorinated pyrazole derivatives from β-alkoxyvinyl trifluoroketones. J. Fluorine Chem..

[22] Lee L. F., Schleppnik F. M., Schneider R. W., Campbell D. H. (1990). Synthesis and ^13^C NMR
of (trifluoromethyl)­hydroxypyrazoles. J. Heterocyclic
Chem..

[23] Mellor J. M., Schofield S. R., Korn S. R. (1997). Reactions of ketene dithioacetals
with bis-nucleophiles: Synthesis of novel heterocyclic thiols. Tetrahedron.

[24] Topchiy M.
A., Zharkova D. A., Asachenko A. F., Muzalevskiy V. M., Chertkov V. A., Nenajdenko V. G., Nechaev M. S. (2018). Mild and regioselective synthesis of 3-CF_3_-pyrazoles by the AgOTf-catalysed reaction of CF_3_-ynones
with hydrazines. Eur. J. Org. Chem..

[25] Touzot A., Soufyane M., Berber H., Toupet L., Mirand C. (2004). Synthesis
of trifluoromethylated pyrazoles from trifluoromethylenaminones and
monosubstituted hydrazines. J. Fluorine Chem..

[26] Wang Q., He L., Li K. K., Tsui G. C. (2017). Copper-mediated domino cyclization/trifluoromethylation/deprotection
with TMSCF_3_: Synthesis of 4-(trifluoromethyl) pyrazoles. Org. Lett..

[27] Fields R., Tomlinson J. P. (1979). Preparation
of trifluoromethyl-pyrazoles and -pyrazolines by the reaction of 2,2,2-trifluorodiazoethane
with carbon-carbon multiple bonds. J. Fluorine
Chem..

[28] Meazza G., Zanardi G., Piccardi P. (1993). A convenient and versatile synthesis
of 4-trifluoromethyl-substituted pyrazoles. J. Heterocycl. Chem..

[29] Lu J., Man Y., Zhang Y., Lin B., Lin Q., Weng Z. (2019). Copper-catalyzed
chemoselective synthesis of 4-trifluoromethyl pyrazoles. RSC Adv..

[30] Kowalczyk A., Utecht-Jarzyńska G., Mlostoń G., Jasiński M. (2022). Trifluoromethylated pyrazoles via
sequential (3 + 2)-cycloaddition
of fluorinated nitrile imines with chalcones and solvent-dependent
deacylative oxidation reactions. Org. Lett..

[31] O’Hara F., Blackmond D. G., Baran P. S. (2013). Radical-based regioselective C–H
functionalization of electron-deficient heteroarenes: scope, tunability,
and predictability. J. Am. Chem. Soc..

[32] Wang L., Zhang Y., Li F., Hao X., Zhang H.-Y., Zhao J. (2018). Direct C–H trifluoromethylation
of quinoxalin-2­(1*H*)-ones under transition-metal-free
conditions. Adv. Synth. Catal..

[33] O’Brien A. G., Maruyama A., Inokuma Y., Fujita M., Baran P. S., Blackmond D. G. (2014). Radical C-H functionalization of heteroarenes under
electrochemical control. Angew. Chem. Int. Ed..

[34] Fujiwara Y., Dixon J. A., ÓHara F., Funder E. D., Dixon D. D., Rodriguez R. A., Baxter R. D., Herle B., Sach N., Collins M. R., Ishihara Y., Baran P. S. (2012). Practical and innate carbon–hydrogen
functionalization of heterocycles. Nature.

[35] Soeda, K. ; Gotou, T. ; Andou, A. ; Murata, T. Herbicidal composition. Japanese Patent JP S57167902 A, October 16, 1982.

[36] All structures were optimized using Gaussian 16 at the M06–2X/6–311+G(d, p) level of theory, introducing solvation factors with the IEF-PCM method (DMSO as solvent). For more details, see Supporting Information.

[37] In line with the previous results, the introduction of a CF_3_ group does not significantly increase the activation energy (less than 2 kcal/mol higher for the second reaction with **4a** and **4b** in Scheme 2), which explains the ease with which a second reaction can occur to give difunctionalization of the substrates.

[38] Garwood J.
J. A., Chen A. D., Nagib A. A. (2024). Radical Polarity. J. Am. Chem.
Soc..

[39] Rong X. X., Pan H. Q., Dolbier
Jr W. R., Smart B. E. (1994). Reactivity of fluorinated alkyl radicals
in solution. Some absolute rates of hydrogen-atom abstraction and
cyclization. J. Am. Chem. Soc..

