# Supplementary material for: Copper-Mediated Late-Stage Radical Trifluoromethylation of Pyrazole-Type Scaffolds
Source: ACS Omega. 2026 Feb 4;11(6):10664–73. doi: 10.1021/acsomega.5c12212 (PMC12917629; doi:10.1021/acsomega.5c12212)

Supporting Information

Copper-Mediated Late-Stage Radical Trifluoromethylation of Pyrazole-Type Scaffolds

Lucie S. Eisen,[a,b] Jan H. Griwatz,[b] Sven Ruf,[b] María Méndez,[b]* Enrique Gomez-Bengoa[a]*

[a] Department of Organic Chemistry I, Faculty of Chemistry, University of the Basque Country, Manuel Lardizabal 3, 20080 San Sebastián, Spain.
[b] Sanofi R&D, Integrated Drug Discovery, Industriepark Höchst, Building G838, 65926 Frankfurt am Main, Germany
* Corresponding Authors. E-mail: maria.mendezperez@sanofi.com, enrique.gomez@ehu.eus

Table of Contents

1 General Information **S2**

2 Optimization Process **S3**

3 Substrate Preparation **S6**

4 Trifluoromethylation **S15**
4.1 General procedure A for the Trifluoromethylation of Pyrazoles S15
4.2 Product Characterization Data S15

5 Limitations **S30**

6 X-ray Crystal Structure of 6j **S31**

7 Computational Studies **S38**

8 References **S59**

9 NMR Spectra **S62**

1. General Information

Reactions were run with efficient magnetic stirring in oven-dried glassware (100 °C oven temperature) and under air, unless otherwise specified. Compound names were generated using ChemDraw. Unless otherwise noted, chemicals were obtained from commercial suppliers and used as received. Tetrahydrofuran and dichloromethane were dried on PS-MD-2 columns. When necessary, other solvents were dried on activated molecular sieves and degassed by bubbling nitrogen gas for at least 15 min. Solvents were evaporated using a Büchi rotary evaporator under reduced pressure at $T \leq 45°C$. Yields refer to chromatographically and spectroscopically pure material, unless otherwise stated. Yields given for trifluoromethylation reactions represent the average of at least three independent runs. Analytical thin-layer chromatography (TLC) was performed using aluminium sheets coated with 0.2 mm of silica gel (Merck Kieselgel 60 F254). Visualization was achieved under UV light at 254 nm or by staining with an alkaline aqueous potassium permanganate solution, ninhydrin, or vanillin. Manual column chromatography was carried out using silica gel (40-63 µm). Flash column chromatography was carried out on a Biotage Selekt system using pre-packed Biotage Sfär silica columns.

NMR spectra were recorded in deuterated solvents on Bruker Ultrashield spectrometers operating at ^{1}H resonances of 300, 400 or 500 MHz, at the temperatures indicated on the spectra. Proton and carbon chemical shifts are given in parts per million (ppm) downfield from tetramethylsilane (TMS), using the solvent or TMS (when present) as reference. ^{1}H NMR spectra are reported as follows: chemical shift (multiplicity, coupling constant, number of protons). The following abbreviations are used: s = singlet, d = doublet, t = triplet, q = quartet, quint = quintet, h = hextet, hept = heptet, m = multiplet, br = broad signal, app = apparent). ^{13}C and ^{19}F spectra were always acquired with proton decoupling, even when not explicitly mentioned. Two-dimensional NMR spectroscopy experiments (COSY, HSQC, HMBC) were used to assist in the assignment of signals in ^{1}H and ^{13}C spectra and to differentiate between regioisomers. High-resolution mass spectrometry (HRMS) was recorded by SGIker staff at the University of the Basque Country on a LC/Q-TOF system equipped with an ESI or APCI ion source, and by staff at Sanofi on a UPLC/Q-TOF system equipped with an ESI or APCI ion source. Single crystal X-ray diffraction (XRD) data was collected by staff at the Servicio Interdepartemental de Investigación (SIdI) at the Universidad Autónoma de Madrid on a XtaLAB Synergy R, HyPix-Arc 100 diffractometer at $T = 200$ K. The structure was solved with the SheIXT 2018/2 (Sheldrick, 2018) structure solution program using the Intrinsic Phasing solution method and by using Olex (Dolomanov et al., 2009) as the graphical interface. The model was refined with version 2018/3 of SheIXL 2018/3 (Sheldrick, 2015) using Least Squares minimization.

2. Optimization Process

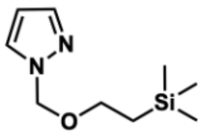

1k

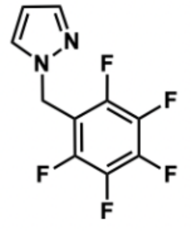

1l

All yields given in the tables are NMR yields given in %, using dibromomethane as the internal standard.

Table S1: Selected entries from the screening process on substrate **1l**

Entry	Oxidant	Catalyst	Solvent	Temp	Additive	Yield C5:C3-CF_3
1	$(NH_4)_2S_2O_8$	$CuSO_4 \cdot 5H_2O$	DMSO/H_2O 5:2	r.t.	-	65:28
2	$(NH_4)_2S_2O_8$	$Cu(NO_2)_2 \cdot 3H_2O$	DMSO/H_2O 5:2	r.t.	-	59:27
3	$(NH_4)_2S_2O_8$	$Cu(OAc)_2$	DMSO/H_2O 5:2	r.t.	-	54:27
4	$(NH_4)_2S_2O_8$	-	DMSO/H_2O 5:2	r.t.	-	6:15
5	$(NH_4)_2S_2O_8$	$Cu(OAc)_2$	DMSO/H_2O 5:2	75°C	-	45:21
6	$K_2S_2O_8$	$Cu(OAc)_2$	DMSO/H_2O 5:2	75°C	-	18:15
7	$Na_2S_2O_8$	$Cu(OAc)_2$	DMSO/H_2O 5:2	75°C	-	29:18
8	tBuOOH	$Cu(OAc)_2$	DMSO/H_2O 5:2	75°C	-	16:13
9	AIBN	$Cu(OAc)_2$	DMSO/H_2O 5:2	75°C	-	not found
10	H_2O_2	$Cu(OAc)_2$	DMSO/H_2O 5:2	75°C	-	5:4
11	CAN	$Cu(OAc)_2$	DMSO/H_2O 5:2	75°C	-	35:19
12	tBuOOH	$Cu(OAc)_2$	DMSO/H_2O 5:2	75°C	-	28:19
13	$(NH_4)_2S_2O_8$	$Cu(OAc)_2$	Acetonitrile	75°C	-	26:18
14	$(NH_4)_2S_2O_8$	$Cu(OAc)_2$	DMSO	75°C	-	37:18
15	$(NH_4)_2S_2O_8$	$Cu(OAc)_2$	DMSO/H_2O 5:2	75°C	1,10-phenanthroline	32:19
16	$(NH_4)_2S_2O_8$	$Cu(OAc)_2$	DMSO/H_2O 5:2	75°C	ethylenediamine	22:19
17	$(NH_4)_2S_2O_8$	$PdCl_2(PPh_3)_2$	DMSO/H_2O 5:2	r.t.	$ZnCl_2$	36:18

AIBN = 2, 2'-azobisisobutyronitrile
CAN = ceric ammonium nitrate

Table S2: Extensive transition metal screening on substrate **1l**

Entry	Oxidant	Catalyst	Solvent	Temp	Additive	Yield C5:C3-CF_3
1	$(NH_4)_2S_2O_8$	$CuCl_2$	DMSO/H_2O 5:2	75°C	-	31:17
2	$(NH_4)_2S_2O_8$	$CuBr_2$	DMSO/H_2O 5:2	75°C	-	38:21
3	$(NH_4)_2S_2O_8$	$Cu(acac)_2$	DMSO/H_2O 5:2	75°C	-	31:23
4	$(NH_4)_2S_2O_8$	$Cu(OTf)_2$	DMSO/H_2O 5:2	75°C	-	44:21
5	$(NH_4)_2S_2O_8$	CuO	DMSO/H_2O 5:2	75°C	-	20:23
6	$(NH_4)_2S_2O_8$	CuOAc	DMSO/H_2O 5:2	75°C	-	37:22
7	$(NH_4)_2S_2O_8$	CuCl	DMSO/H_2O 5:2	75°C	-	36:21
8	$(NH_4)_2S_2O_8$	CuBr	DMSO/H_2O 5:2	75°C	-	27:21
9	$(NH_4)_2S_2O_8$	Cu_2O	DMSO/H_2O 5:2	75°C	-	14:20
10	$(NH_4)_2S_2O_8$	$Cu(MeCN)_4PF_6$	DMSO/H_2O 5:2	75°C	-	34:22
11	$(NH_4)_2S_2O_8$	$Cu(MeCN)_4BF_4$	DMSO/H_2O 5:2	75°C	-	39:26
12	$(NH_4)_2S_2O_8$	Fe	DMSO/H_2O 5:2	75°C	-	9:17
13	$(NH_4)_2S_2O_8$	$Fe(OAc)_2$	DMSO/H_2O 5:2	75°C	-	8:17
14	$(NH_4)_2S_2O_8$	$FeCl_2$	DMSO/H_2O 5:2	75°C	-	13:18
15	$(NH_4)_2S_2O_8$	$FeCl_3$	DMSO/H_2O 5:2	75°C	-	11:20
16	$(NH_4)_2S_2O_8$	FeF_2	DMSO/H_2O 5:2	75°C	-	12:17
17	$(NH_4)_2S_2O_8$	$Fe(OTf)_2$	DMSO/H_2O 5:2	75°C	-	14:22
18	$(NH_4)_2S_2O_8$	$FeBr_2$	DMSO/H_2O 5:2	75°C	-	8:18
19	$(NH_4)_2S_2O_8$	$Fe(acac)_2$	DMSO/H_2O 5:2	75°C	-	8:18
20	$(NH_4)_2S_2O_8$	$Fe(acac)_3$	DMSO/H_2O 5:2	75°C	-	10:18
21	$(NH_4)_2S_2O_8$	Fe_2O_3	DMSO/H_2O 5:2	75°C	-	10:18
22	$(NH_4)_2S_2O_8$	$Fe_2(SO_4)_3 \cdot H_2O$	DMSO/H_2O 5:2	75°C	-	34:22
23	$(NH_4)_2S_2O_8$	$Cu(MeCN)_4BF_4$	DMSO/H_2O 5:2	75°C	-	8:17
24	$(NH_4)_2S_2O_8$	$Co(OAc)_2 \cdot 4H_2O$	DMSO/H_2O 5:2	75°C	-	7:17
25	$(NH_4)_2S_2O_8$	$Co(acac)_2 \cdot H_2O$	DMSO/H_2O 5:2	75°C	-	12:17
26	$(NH_4)_2S_2O_8$	$Co(ClO_4)_2 \cdot 6H_2O$	DMSO/H_2O 5:2	75°C	-	9:15
27	$(NH_4)_2S_2O_8$	CoF_2	DMSO/H_2O 5:2	75°C	-	9:16
28	$(NH_4)_2S_2O_8$	$CoCl_2$	DMSO/H_2O 5:2	75°C	-	7:14
29	$(NH_4)_2S_2O_8$	$CoBr_2$	DMSO/H_2O 5:2	75°C	-	5:15
30	$(NH_4)_2S_2O_8$	$Co(OH)_2$	DMSO/H_2O 5:2	75°C	-	7:18
31	$(NH_4)_2S_2O_8$	$Co(NO_3)_2 \cdot 6H_2O$	DMSO/H_2O 5:2	75°C	-	5:13
32	$(NH_4)_2S_2O_8$	Mn	DMSO/H_2O 5:2	75°C	-	9:15
33	$(NH_4)_2S_2O_8$	$Mn(OAc)_2 \cdot 2H_2O$	DMSO/H_2O 5:2	75°C	-	2:14
34	$(NH_4)_2S_2O_8$	$MnO_2 \cdot 6H_2O$	DMSO/H_2O 5:2	75°C	-	3:14
35	$(NH_4)_2S_2O_8$	$NiCl_2 \cdot DME$	DMSO/H_2O 5:2	75°C	-	7:16
36	$(NH_4)_2S_2O_8$	$NiCl_2(dppf)$	DMSO/H_2O 5:2	75°C	-	7:16
37	$(NH_4)_2S_2O_8$	$NiCl_2)(PCy_3)_2 \cdot 6H_2O$	DMSO/H_2O 5:2	75°C	-	8:18
38	$(NH_4)_2S_2O_8$	$Ni(OTf)_2 \cdot 6H_2O$	DMSO/H_2O 5:2	75°C	-	3:14
39	$(NH_4)_2S_2O_8$	$AgNTf_2$	DMSO/H_2O 5:2	75°C	-	13:18
40	$(NH_4)_2S_2O_8$	AgOAc	DMSO/H_2O 5:2	75°C	-	9:17
41	$(NH_4)_2S_2O_8$	$AgBF_4$	DMSO/H_2O 5:2	75°C	-	8:19
42	$(NH_4)_2S_2O_8$	$AgNO_3 \cdot 6H_2O$	DMSO/H_2O 5:2	75°C	-	9:20
43	$(NH_4)_2S_2O_8$	AuI	DMSO/H_2O 5:2	75°C	-	10:17
44	$(NH_4)_2S_2O_8$	ClAu·DMS	DMSO/H_2O 5:2	75°C	-	10:8
45	$(NH_4)_2S_2O_8$	$PdCl_2(PPh_3)_2$	DMSO/H_2O 5:2	75°C	-	13:19
46	$(NH_4)_2S_2O_8$	$Pd(OAc)_2$	DMSO/H_2O 5:2	75°C	-	11:20
47	$(NH_4)_2S_2O_8$	-	DMSO/H_2O 5:2	75°C	-	6:16

Table S3: Selected entries from the screening process on substrate **1k**

Entry	Oxidant	Catalyst	Solvent	Temp	Additive	Yield C5:C3-CF_3
1	$(NH_4)_2S_2O_8$	$Cu(OTf)_2$	DMSO/H_2O 5:2	r.t.	-	42:23
2	$(NH_4)_2S_2O_8$	$Cu(OAc)_2$	DMSO/H_2O 5:2	r.t.	-	38:15
3	$(NH_4)_2S_2O_8$	$Cu(NO_2)_2{\cdot}3H_2O$	DMSO/H_2O 5:2	r.t.	-	37:16
4	$(NH_4)_2S_2O_8$	CuF_2	DMSO/H_2O 5:2	r.t.	-	36:20
5	$(NH_4)_2S_2O_8$	CuCl	DMSO/H_2O 5:2	r.t.	-	33:30
6	$(NH_4)_2S_2O_8$	CuOAc	DMSO/H_2O 5:2	r.t.	-	27:13
7	$(NH_4)_2S_2O_8$	$CuSO_4{\cdot}5H_2O$	DMSO/H_2O 5:2	r.t.	-	25:11
8	$(NH_4)_2S_2O_8$	$Cu(OAc)_2$	DMSO	r.t.	-	20:10
9	$(NH_4)_2S_2O_8$	$Cu(OAc)_2$	Acetonitrile	r.t.	-	12:6
10	$(NH_4)_2S_2O_8$	$Cu(OAc)_2$	DMF	r.t.	-	20:10

Table S4: Copper(II) salt screening on substrate **3a-SM**

Entry	Oxidant	Catalyst	Solvent	Temp	Yield C5:C3-CF_3
1	$(NH_4)_2S_2O_8$	$CuSO_4{\cdot}5H_2O$	DMSO/H_2O 5:2	r.t.	59:17
2	$(NH_4)_2S_2O_8$	$Cu(OTf)_2$	DMSO/H_2O 5:2	r.t.	44:13
3	$(NH_4)_2S_2O_8$	$Cu(NO_2)_2{\cdot}3H_2O$	DMSO/H_2O 5:2	r.t.	43:12

Table S5: Copper(II) salt screening on substrate **3b-SM**

Entry	Oxidant	Catalyst	Solvent	Temp	Yield C5:C3-CF_3
1	$(NH_4)_2S_2O_8$	$CuSO_4{\cdot}5H_2O$	DMSO/H_2O 5:2	r.t.	34:14
2	$(NH_4)_2S_2O_8$	$Cu(OAc)_2$	DMSO/H_2O 5:2	r.t.	34:13
3	$(NH_4)_2S_2O_8$	$Cu(OTf)_2$	DMSO/H_2O 5:2	r.t.	26:11
4	$(NH_4)_2S_2O_8$	$Cu(NO_2)_2{\cdot}3H_2O$	DMSO/H_2O 5:2	r.t.	24:12

Table S6: Copper(II) salt screening on substrate **5f-SM**

Entry	Oxidant	Catalyst	Solvent	Temp	Yield C5-CF_3
1	$(NH_4)_2S_2O_8$	$CuSO_4{\cdot}5H_2O$	DMSO/H_2O 5:2	r.t.	44
2	$(NH_4)_2S_2O_8$	$Cu(OTf)_2$	DMSO/H_2O 5:2	r.t.	32

3. Substrate Preparation

1-butyl-1*H*-pyrazole (1b)

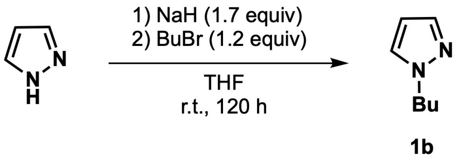

NaH 60 % dispersion in mineral oil (3.00 g, 75.0 mmol, 1.70 equiv) was washed with n-hexane three times, then THF (65 mL) was added. Pyrazole (3.0 g, 44.1 mmol, 1.0 equiv) was added slowly and the resulting suspension was stirred at room temperature for 30 min. 1-bromobutane (7.27 g, 53.0 mmol, 1.2 equiv) was added and the reaction mixture was stirred at room temperature for 120 h. The solvent was evaporated. The residue was partitioned between aqueous saturated NH_4Cl and ethyl acetate. The aqueous layer was extracted three times with ethyl acetate. The combined organic layers were washed with brine, dried over $MgSO_4$, filtered and evaporated to dryness. The crude material was purified by silica gel column chromatography (10 % ethyl acetate in n-hexane), to give 1-butyl-1*H*-pyrazole (1.95 g, 15.7 mmol, 37 %) as a colorless oil. ^{1}**H NMR** (400 MHz, $CDCl_3$) δ 7.46 (s, 1H), 7.33 (d, J = 2.4 Hz, 1H), 6.19 (t, J = 2.1 Hz, 1H), 4.09 (t, J = 7.2 Hz, 2H), 1.81 (quint, J = 7.2 Hz, 2H), 1.28 (h, J = 7.4 Hz, 2H), 0.90 (t, J = 7.4 Hz, 3H). ^{13}**C NMR** (101 MHz, $CDCl_3$) δ 138.9, 128.8, 105.5, 51.7, 32.4, 19.7, 13.5. The NMR data is consistent with the literature [1].

1-(triisopropylsilyl)-1*H*-pyrazole (1c)

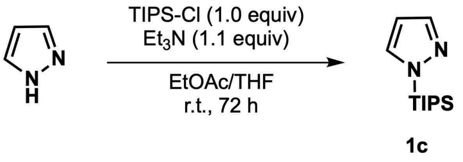

Adapted from a reported procedure [2]. Ethyl acetate (5 mL) was dried on molecular sieves overnight. Pyrazole (1.00 g, 14.7 mmol, 1.00 equiv) was dissolved in the dried ethyl acetate. The resulting solution was added slowly into a solution of anhydrous Et_3N (1.64 g, 16.2 mmol, 1.1 equiv) and triisopropylsilyl chloride (2.83 g, 14.7 mmol, 1.0 equiv) in dried THF (5 mL). A white precipitate of Et_3NHCl appeared. The reaction mixture was stirred at room temperature under N_2 atmosphere for 72 h. The reaction was quenched with 100 mL of dry n-hexane. The mixture was filtered and the solvent of the filtrate was evaporated. 1-(triisopropylsilyl)-1*H*-pyrazole (1.28 g, 5.72 mmol, 39 %) was obtained as a colorless oil. ^{1}**H NMR** (400 MHz, $CDCl_3$) δ 7.77 (d, J = 1.6 Hz, 1H), 7.62 (d, J = 2.3 Hz, 1H), 6.33 (dd, J = 2.3 Hz, 1.6 Hz, 1H), 1.54 (hept, J = 7.5 Hz, 3H), 1.09 (d, J = 7.7 Hz, 18H). ^{13}**C NMR** (101 MHz, $CDCl_3$) δ 142.9, 135.2, 105.9, 17.7, 11.8. **HRMS** (APCI/Q-TOF) m/z [M^+] *calcd* 224.1709 *found* 224.1703.

1-(methylsulfonyl)-1*H*-pyrazole (1d)

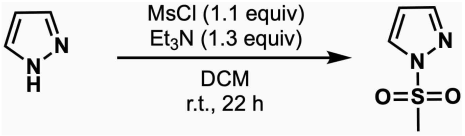

Following a reported procedure [3], pyrazole (1.00 g, 14.7 mmol, 1.00 equiv) was dissolved in DCM (40 mL). Et_3N (2.66 mL, 1.93 mg, 19.1 mmol, 1.30 equiv) was added and the solution was cooled to 0 ° C. Methanesulfonyl chloride (1.25 mL, 1.85 g, 16.2 mmol, 1.10 equiv) was added dropwise. The reaction mixture was stirred at room temperature for 22 h. The reaction was quenched with aqueous saturated NH_4Cl. The aqueous

layer was extracted three times with DCM. The combined organic layers were washed with brine, dried over $MgSO_4$ and evaporated to dryness, to give 1-(methylsulfonyl)-1*H*-pyrazole (2.12 g, 14.5 mmol, 99 %) as a yellow oil. ^{1}**H NMR** (400 MHz, $CDCl_3$) δ 8.05 (dd, J = 2.8 Hz, 0.8 Hz, 1H), 7.83 (d, J = 2.0 Hz, 1H), 6.46 (dd, J = 2.8, 1.6 Hz, 1H), 3.33 (s, 3H). ^{13}**C NMR** (101 MHz, $CDCl_3$) δ 144.6, 130.8, 108.4, 41.0. The NMR data is consistent with the literature [4].

Cyclohexyl(1*H*-pyrazol-1-yl)methanone (1e)

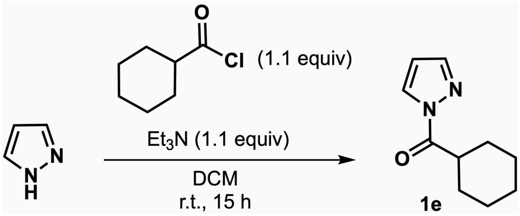

Following a reported procedure [5], pyrazole (1.00 g, 14.7 mmol, 1.00 equiv) was dissolved in dried DCM (15 mL). Molecular sieves and dried Et_3N (2.26 mL, 1.64 g, 16.2 mmol, 1.10 equiv) were added. The flask was degassed and placed under N_2 atmosphere. In a separate flask a solution of cyclohexanocarbonyl chloride (2.16 mL, 2.37 g, 16.2 mmol, 1.10 equiv) in dried DCM (7 mL) was prepared in the presence of molecular sieves. This solution was added dropwise into the pyrazole solution by means of a syringe. The reaction mixture was stirred at room temperature for 15 h. The reaction mixture was diluted with Et_2O and filtered on fritted glass. The filter cake was washed with Et_2O. The filtrate was evaporated to dryness. The crude material was purified by silica gel column chromatography (10 % ethyl acetate in n-hexane) to give cyclohexyl(1*H*-pyrazol-1-yl)methanone (2.20 g, 12.4 mmol, 84 %) as a colorless oil. ^{1}**H NMR** (400 MHz, $CDCl_3$) δ 8.18 (d, J = 2.7 Hz, 1H), 7.65 (d, J = 1.8 Hz, 1H), 6.36 (dd, J = 3.0 Hz, 1.6 Hz, 1H), 3.59 (t, J = 11.7 Hz), 2.00 – 1.86 (m, 2H), 1.84 - 1.72 (m, 2H), 1.72 – 1.62 (m, 1H), 1.51 (q, J = 12.4 Hz, 2H), 1.3 (q, J = 12.8 Hz, 2H), 1.30 – 1.15 (m, 1H). ^{13}**C NMR** (101 MHz, $CDCl_3$) δ 175.0, 143.7, 128.4, 109.3, 41.5, 29.1, 25.8, 25.4. The NMR data is consistent with the literature [6].

Perfluorophenyl(1*H*-pyrazol-1-yl)methanone (1f)

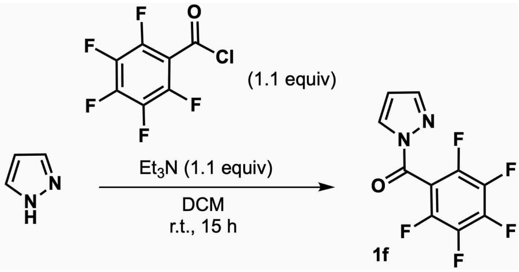

Adapted from a reported procedure [5]. Pyrazole (700 mg, 10.3 mmol, 1.00 equiv) was dissolved in dried DCM (11 mL). Molecular sieves and dried Et_3N (1.57 mL, 1.14 g, 11.3 mmol, 1.10 equiv) were added. The flask was degassed and placed under N_2 atmosphere. In a separate flask a solution of pentafluorobenzoyl chloride (1.63 mL, 2.61 g, 11.3 mmol, 1.10 equiv) in dried DCM (5 mL) was prepared in the presence of molecular sieves. This solution was added dropwise into the pyrazole solution by means of a syringe. The reaction mixture was stirred at room temperature for 15 h, after which NMR showed incomplete conversion. Pentafluorobenzoyl chloride (587 µL, 940 mg, 4.08 mmol, 0.40 equiv) was added and the mixture was stirred for another 14 h. The reaction mixture was diluted with Et_2O and filtered on fritted glass. The filter cake was washed with Et_2O. The filtrate was evaporated to dryness. The crude material was purified by silica gel column chromatography (10 % ethyl acetate in n-hexane) to give perfluorophenyl(1*H*-pyrazol-1-yl)methanone (1.20 g, 4.58 mmol, 44 %) as a pale yellow oil. ^{1}**H NMR** (400 MHz, $CDCl_3$) δ 8.38 (d, J = 3.0 Hz, 1H), 7.76 (s, 1H), 6.59 (dd, J = 2.9 Hz, 1.4 Hz, 1H). ^{13}**C NMR** (101 MHz, $CDCl_3$) δ 157.4, 146.0, 144.2 (dm, $^1J_{C\text{-}F}$ = 254.2 Hz), 143.3 (dm, $^1J_{C\text{-}F}$ = 258.8 Hz), 137.6 (dt, $^1J_{C\text{-}F}$ = 257.4 Hz, $^2J_{C\text{-}F}$ = 17.4 Hz), 129.1, 111.6,

109.7 (t, $^2J_{C\text{-}F}$ = 22.5 Hz). ^{19}**F NMR** (376 MHz, $CDCl_3$) δ -138.47 – -138.75 (m), -148.71 – -150.20 (m) -160.28 – -160.93 (m). **HRMS** (APCI/Q-TOF) m/z [M^+] *calcd* 262.0166 *found* 262.0172.

***tert*-butyl 1*H*-pyrazole-1-carboxylate (1g)**

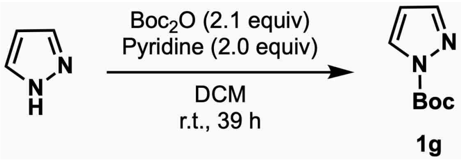

Adapted from two reported procedures [7], [8]. Pyrazole (1.00 g, 14.7 mmol, 1.00 equiv) was dissolved in dried DCM (29 mL). Pyridine (2.36 mL, 2.32 g, 29.4 mmol, 2.00 equiv) was added. The solution was cooled to 0°C. Di-*tert*-butyl dicarbonate (Boc_2O) (5.06 mL, 4.81 g, 22.0 mmol, 1.50 equiv) was added slowly over about 20 min. The reaction was stirred at room temperature for 24 h. As TLC showed incomplete conversion, more Boc_2O (2.02 mL, 1.92 g, 8.80 mmol, 0.60 equiv) was added and the reaction mixture was stirred for an additional 15 h. The reaction mixture was washed with water. The aqueous layer was extracted twice with DCM. The combined organic layers were washed with brine, dried over $MgSO_4$, and evaporated to dryness. An attempt at purification by silica gel column chromatography (10 % ethyl acetate in n-hexane) gave 2.47 g of a mixture of the desired product and unreacted Boc_2O. The mixture was dissolved in 7 mL methanol. Imidazole (420 mg, 6.2 mmol) and *N,N*-dimethylaminopyridine DMAP (81 mg, 0.66 mmol) were added. The mixture was stirred at room temperature for 10 min and then evaporated to dryness. The mixture was purified by silica gel column chromatography (10 % ethyl acetate in n-hexane) to give *tert*-butyl 1*H*-pyrazole-1-carboxylate (732 mg, 4.35 mmol, 30 %) as a colorless oil. ^{1}**H NMR** (400 MHz, $CDCl_3$) δ 8.08 (d, J = 2.8 Hz, 1H), 7.70 (s, 1H), 6.40 – 6.30 (m, 1H), 1.64 (s, 9H). ^{13}**C NMR** (101 MHz, $CDCl_3$) δ 147.6, 143.8, 130.5, 108.5, 85.3, 27.8. The data is consistent with the literature [9].

1-((perfluorophenoxy)methyl)-1*H*-pyrazole (1h)

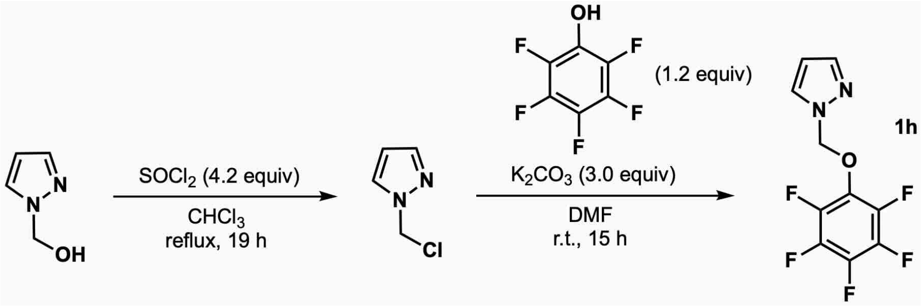

Step 1 Following a reported procedure [10], (1*H*-pyrazol-1-yl)methanol (1.60 g, 16.3 mmol, 1.00 equiv) was dissolved in chloroform (70 mL). Thionyl chloride (5.00 mL, 8.20 g, 68.9 mmol, 4.20 equiv) was added over the course of several minutes. The reaction mixture was heated under reflux for 19 h. The volatiles were evaporated, affording the crude material as a thick, colorless oil. 1-(chloromethyl)-1*H*-pyrazole hydrochloride could not be isolated due to its high sensitivity to water [10]. It was used in the next step without further purification.

Step 2 Adapted from a reported procedure [11]. The crude 1-(chloromethyl)-1*H*-pyrazole hydrochloride was dissolved in dried DMF (15 mL) under N_2 atmosphere. A separate flask under N_2 atmosphere was charged with pentafluorophenol (2.05 mL, 3.60 g, 19.6 mmol, 1.20 eq), K_2CO_3 (6.76 g, 48.9 mmol, 3.00 equiv) and dried DMF (15 mL). The mixture was stirred for 30 min, then slowly added into the (chloromethyl)-1*H*-pyrazole hydrochloride solution. The reaction mixture was stirred at room temperature for 15 h. The DMF was evaporated and the residue was partitioned between ethyl acetate and water. The aqueous layer was extracted three times with ethyl acetate. The combined organic layers were washed once with water and once with brine, dried over $MgSO_4$ and evaporated to dryness. The crude material was purified by silica gel

column chromatography (10 % ethyl acetate in n-hexane) to give 1-((perfluorophenoxy)methyl)-1*H*-pyrazole (1.29 g, 4.87 mmol, 25 % yield over two steps) as a pale yellow oil. ^{1}**H NMR** (400 MHz, $CDCl_3$) δ 7.62 (d, J = 2.5 Hz, 1H), 7.51 (d, J = 1.8 Hz, 1H), 6.36 (dd, J = 2.5, 1.8 Hz, 1H), 5.96 (s, 2H). ^{13}**C NMR** (75 MHz, $CDCl_3$) δ 142.7 (dm, $^{1}J_{C\text{-}F}$ = 257.8 Hz), 142.0, 138.9 (dtt, $^{1}J_{C\text{-}F}$ = 250.0 Hz, $^{2}J_{C\text{-}F}$ = 13.3 Hz, $^{3}J_{C\text{-}F}$ = 3.9 Hz), 138.2 (dtdd, $^{1}J_{C\text{-}F}$ = 251.4 Hz, $^{2}J_{C\text{-}F}$ = 13.5 Hz, $^{3}J_{C\text{-}F}$ = 5.6 Hz, $^{4}J_{C\text{-}F}$ = 2.3 Hz), 130.8, 108.4, 108.3 (m) 82.1. ^{19}**F NMR** (376 MHz, $CDCl_3$) δ -155.80 (dm, $^{3}J_{F\text{-}F}$ = 19.8 Hz), -160.53 (t, $^{3}J_{F\text{-}F}$ = 21.4 Hz), -162.84 (td, $^{3}J_{F\text{-}F}$ = 22.0 Hz, $^{4}J_{F\text{-}F}$ = 5.1 Hz). **HRMS** (ESI/Q-TOF) m/z [M^+] *calcd* 264.0322 *found* 264.0324.

2,2-dimethyl-1-(1*H*-pyrazol-1-yl)propan-1-one (1i)

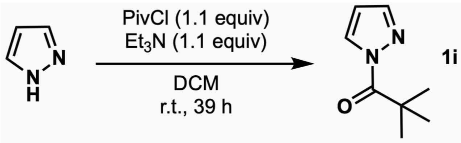

Procedure adapted from [5]. A round-bottom flask was charged with pyrazole (2.00 g, 29.4 mmol, 1.00 equiv) in dried DCM (30 mL), in the presence of molecular sieves. The flask was degassed and placed under N_2 atmosphere. In a separate flask, pivaloyl chloride (3.96 mL, 3.90 g, 32.3 mmol, 1.10 equiv) and Et_3N (4.50 mL, 3.27 g, 32.3 mmol, 1.10 equiv) were dissolved in DCM (10 mL) in the presence of molecular sieves. This solution was added dropwise into the pyrazole solution. The reaction mixture was stirred at room temperature for 15 h. The reaction mixture was filtered over fritted glass and the filter cake was washed with Et_2O. A white solid appeared in the filtrate upon contact with Et_2O. Evaporation gave the white solid and a colorless oil. The crude material was purified by silica gel column chromatography (5 % ethyl acetate in n-hexane) to give 2,2-dimethyl-1-(1*H*-pyrazol-1-yl)propan-1-one (2.95 g, 19.4 mmol, 66 %) as a pale yellow oil. ^{1}**H NMR** (400 MHz, $CDCl_3$) δ 8.24 (d, J = 2.9 Hz, 1H), 7.65 (s, 1H), 6.34 – 6.30 (m 1H), 1.49 (s, 9H). ^{13}**C NMR** (101 MHz, $CDCl_3$) δ 176.6, 143.1, 130.1, 108.0, 41.4, 27.7. **HRMS** (ESI/Q-TOF) m/z [M^+] *calcd* 152.0950 *found* 152.0954.

1-(tetrahydro-2*H*-pyran-2-yl)-1*H*-pyrazole (1j)

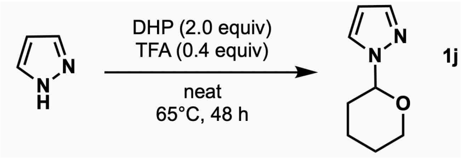

Following a reported procedure [12]. To a solution of pyrazole (3.00 g, 44.1 mmol, 1.00 equiv) in 3,4-dihydro-2*H*-pyrane (8.04 mL, 7.41 g, 88.1 mmol, 2.00 equiv) was added trifluoroacetic acid (1.21 mL, 1.81 g, 15.9 mmol, 0.40 equiv). The reaction mixture was stirred at 65°C for 48 h. Once cooled down to room temperature, the mixture was diluted with water and some saturated aqueous $NaHCO_3$. The aqueous solution was extracted three times with ethyl acetate. The combined organic layers were dried over $MgSO_4$ and evaporated to dryness. The crude material was purified by Kugelrohr distillation at 120°C and 3.5 mbar to give 1-(tetrahydro-2*H*-pyran-2-yl)-1*H*-pyrazole (5.70 g, 37.5 mmol, 59 %) as a colorless oil. ^{1}**H NMR** (400 MHz, $CDCl_3$) δ 7.60 (d, J = 2.5 Hz, 1H), 7.55 (d, J = 1.9 Hz, 1H), 6.29 (t, J = 2.1 Hz, 1H), 5.39 (dd, J = 9.6 Hz, 2.8 Hz, 1H), 4.09 – 4.02 (m, 1H), 3.69 (td, J = 11.4 Hz, 2.8 Hz, 1H), 2.19 – 1.97 (m, 3H), 1.76 – 1.51 (m, 3H). ^{13}**C NMR** (101 MHz, $CDCl_3$) δ 139.3, 127.7, 105.9, 87.4, 67.7, 30.5, 24.9, 22.4. The data is consistent with the literature [13].

1-((2-(trimethylsilyl)ethoxy)methyl)-1*H*-pyrazole (1k)

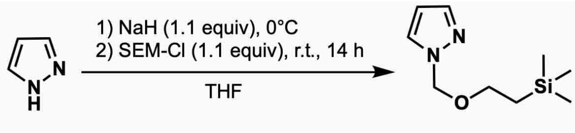

1k

Following a reported procedure [13], NaH 60 % dispersion in mineral oil (970 mg, 24.2 mmol, 1.10 equiv) was washed three times with n-hexane. The flask was degassed and placed under N_2 atmosphere. Previously dried and degassed THF (25 mL) was added. Pyrazole (1.50 g, 22.0 mmol, 1.00 equiv) was added portionwise. The mixture was stirred at 0°C for 1 h. 2-(trimethylsilyl)ethoxymethyl chloride (4.29 mL, 4.04 g, 24.2 mmol, 1.10 equiv) was added dropwise at 0°C. The reaction mixture was stirred at room temperature for 14 h. The solvent was evaporated. The residue was quenched with brine and the aqueous solution was extracted three times with ethyl acetate. The combined organic layers were washed with water and brine, dried over $MgSO_4$ and evaporated to dryness, to give 1-((2-(trimethylsilyl)ethoxy)methyl)-1*H*-pyrazole (3.80 g, 19.2 mmol, 87 %) as a yellow oil. **^{1}H NMR** (400 MHz, $CDCl_3$) δ 7.52 (d, J = 2.2 Hz, 1H), 7.50 (d, J = 1.8 Hz, 1H), 6.28 (d, J = 2.1 Hz, 1H), 5.39 (s, 2H), 3.63 - 3.38 (m, 2H), 0.92 - 0.76 (m, 2H), -0.08 (s, 9H). **^{13}C NMR** (101 MHz, $CDCl_3$) δ 139.3, 129.0, 106.3, 79.5, 66.0, 17.3, -1.9. The data is consistent with the literature [13].

1-((perfluorophenyl)methyl)-1*H*-pyrazole (1l)

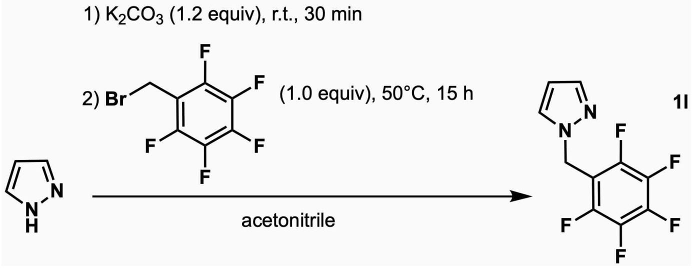

Pyrazole (785 mg, 11.5 mmol, 1.20 equiv) and K_2CO_3 (1.59 g, 11.5 mmol, 1.20 equiv) were stirred in acetonitrile (9 mL) for 30 min at room temperature. 1-(bromomethyl)-2,3,4,5,6-pentafluorobenzene (2.50 g, 9.60 mmol, 1.00 equiv) was added and the reaction mixture was stirred at 50°C for 15 h. The solvent was evaporated. The residue was partitioned between water and ethyl acetate. The aqueous layer was extracted three times with ethyl acetate. The combined organic layers were washed with water and brine, dried over $MgSO_4$ and evaporated to dryness. The crude material was purified by silica gel column chromatography (5 % ethyl acetate in n-hexane) to give 1-((perfluorophenyl)methyl)-1*H*-pyrazole (2.01 g, 8.10 mmol, 84 %) as a white solid. **^{1}H NMR** (400 MHz, $CDCl_3$) δ 7.51 (d, J = 1.9 Hz, 1H), 7.50 (d, J = 2.3 Hz, 1H), 6.27 (t, J = 2.1 Hz, 1H), 5.40 (s, 2H). **^{13}C NMR** (126 MHz, $CDCl_3$) δ 145.6 (dm, $^1J_{C\text{-}F}$ = 250.0 Hz), 142.0 (dtt, $^1J_{C\text{-}F}$ = 256.0 Hz, $^2J_{C\text{-}F}$ = 13.4, $^3J_{C\text{-}F}$ = 5.3 Hz), 140.6, 137.7 (dt, $^1J_{C\text{-}F}$ = 254.2 Hz, $^2J_{C\text{-}F}$ = 12.9 Hz), 129.8, 110.0 (td, $^2J_{C\text{-}F}$ = 17.8 Hz, $^3J_{C\text{-}F}$ = 4.0 Hz), 106.5, 42.7. **^{19}F NMR** (376 MHz, $CDCl_3$) δ -142.61 (dd, $^3J_{F\text{-}F}$ = 22.2 Hz, $^4J_{F\text{-}F}$ = 8.4 Hz), -152.77 (t, $^3J_{F\text{-}F}$ = 20.4 Hz) , -158.08 – -167.31 (m). **HRMS** (ESI/Q-TOF) m/z [M^+] *calcd* 248.0373 *found* 248.0375.

4-methoxy-1-((2-(trimethylsilyl)ethoxy)methyl)-1*H*-pyrazole (3a-SM)

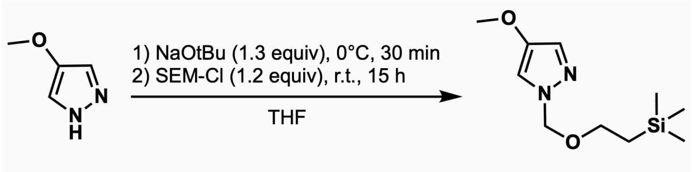

3a-SM

Adapted from a reported procedure [14]. 4-methoxy-1*H*-pyrazole (200 mg, 2.04 mmol, 1.00 equiv) was dissolved in THF (10 mL). Sodium *tert*-butoxide (255 mg, 2.65 mmol, 1.30 equiv) was added and the mixture was stirred at 0°C for 30 min. 2-(trimethylsilyl)ethoxymethyl chloride (433 µL, 408 mg, 2.45 mmol, 1.20 equiv) was added slowly at 0°C and the reaction mixture was stirred at room temperature for 15 h. The solvent was evaporated. The residue was quenched with saturated aqueous NH_4Cl. The aqueous solution was extracted three times with ethyl acetate. The combined organic layers were washed with brine, dried over $MgSO_4$ and evaporated to dryness. The crude material was purified by silica gel column chromatography (10 % ethyl acetate in n-hexane) to give 4-methoxy-1-((2-(trimethylsilyl)ethoxy)methyl)-1*H*-pyrazole (316 mg, 1.39 mmol, 68 %) as a colorless oil. ^{1}**H NMR** (400 MHz, $CDCl_3$) δ 7.25 (d, J = 0.9 Hz, 1H), 7.19 (d, J = 0.9 Hz, 1H), 5.29 (s, 2H), 3.73 (s, 3H), 3.52 – 3.44 (m, 2H), 0.92 – 0.83 (m, 2H), -0.05 (s, 9H). ^{13}**C NMR** (101 MHz, $CDCl_3$) δ 147.9, 127.9, 113.5, 81.0, 66.5, 58.8, 17.8, -1.4. **HRMS** (ESI/Q-TOF) m/z [M$^+$] *calcd* 228.1294 *found* 228.1293.

4-methyl-1-((2-(trimethylsilyl)ethoxy)methyl)-1H-pyrazole (3b-SM)

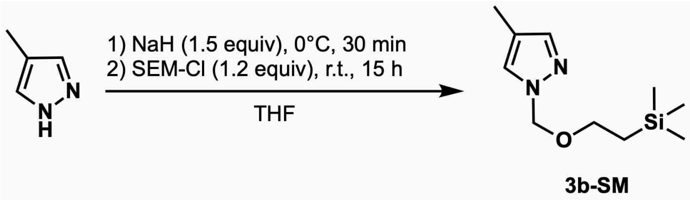

Following a reported procedure [15], 4-methyl-1*H*-pyrazole (505 µL, 500 mg, 6.09 mmol, 1.00 equiv) was dissolved in degassed THF (6 mL) under N_2 atmosphere. NaH 60 % dispersion in mineral oil (365 mg, 9.13 mmol, 1.50 equiv) was washed three times with n-hexane and added carefully into the solution. The mixture was stirred at 0°C for 30 min. 2-(trimethylsilyl)ethoxymethyl chloride (1.30 mL, 1.22 g, 7.31 mmol, 1.20 equiv) was added at 0°C. The reaction mixture was stirred at room temperature for 15 h. The solvent was evaporated and the residue was partitioned between brine and ethyl acetate. The aqueous layer was extracted three times with ethyl acetate. The combined organic layers were washed with brine, dried over $MgSO_4$ and evaporated to dryness. The crude material was purified by silica gel column chromatography (5 % ethyl acetate in n-hexane) to give 4-methyl-1-((2-(trimethylsilyl)ethoxy)methyl)-1H-pyrazole (763 mg, 3.60 mmol, 59 %) as an orange oil. ^{1}**H NMR** (400 MHz, $CDCl_3$) δ 7.33 (s, 1H), 7.31 (s, 1H), 5.35 (s, 2H), 3.59 – 3.49 (m, 2H), 2.08 (s, 3H), 0.95 – 0.83 (m, 2H), -0.04 (s, 9H). ^{13}**C NMR** (101 MHz, $CDCl_3$) δ 140.4, 128.0, 117.2, 79.9, 66.4, 17.7, 8.8, -1.5. **HRMS** (ESI/Q-TOF) m/z [M$^+$] *calcd* 212.1345 *found* 212.1349. The ^{1}H NMR data is in accordance with the literature [16].

1-methyl-2-(1*H*-pyrazol-3-yl)piperidine (5i-SM)

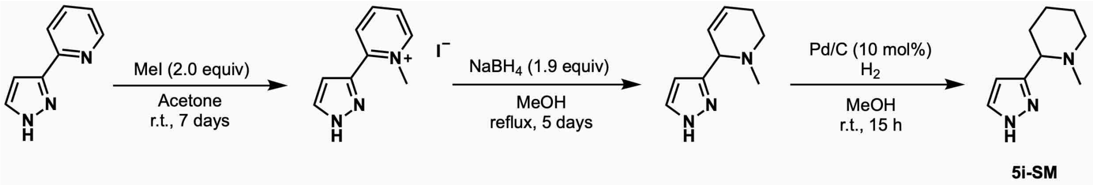

Step 1 Adapted from a reported procedure [17]. To a solution of 2-(1*H*-pyrazol-3-yl)pyridine (1.20 g, 8.27 mmol, 1.00 equiv) in acetone (8 mL) was added iodomethane (1.00 mL, 2.28 g, 16.5 mmol, 2.0 equiv). The reaction mixture was stirred at room temperature for 7 days. The formed precipitate was filtered on fritted glass and washed with acetone, to give 1-methyl-2-(1*H*-pyrazol-3-yl)pyridin-1-ium iodide (1.85 g, 6.44 mmol, 78 %). The compound was used in the next step without further purification. ^{1}**H NMR** (400 MHz, DMSO-d_6) δ 9.05 (d, J = 6.3 Hz, 1H), 8.58 (td, J = 7.9 Hz, 1.6 Hz, 1H), 8.42 (dd, J = 8.3 Hz, 1.8 Hz, 1H), 8.15 (d, J = 2.5 Hz, 1H), 8.04 (ddd, J = 7.8 Hz, 6.2 Hz, 1.6 Hz, 1H), 7.16 (d, J = 2.4 Hz, 1H), 4.48 (s, 3H). ^{13}**C NMR** (101 MHz, DMSO-d_6) δ 147.1, 144.9, 131.1, 128.5, 125.8, 124.1, 108.6, 104.6, 48.3. **HRMS** (ESI/Q-TOF) m/z [M$^+$] *calcd* 160.0869 *found* 160.0876.

Step 2 Adapted from a reported procedure [17]. 1-methyl-2-(1*H*-pyrazol-3-yl)pyridin-1-ium iodide (1.75 g, 6.10 mmol, 1.00 equiv) was dissolved in methanol (50 mL). $NaBH_4$ (346 mg, 9.15 mmol, 1.50 equiv) was added portionwise and the reaction mixture was heated under reflux for 4 days. Another portion of $NaBH_4$ (100 mg, 2.64 mmol, 0.40 equiv) was added and the mixture was heated under reflux for another day. After the mixture cooled down, the solvent was evaporated. The residue was partitioned between saturated aqueous $NaHCO_3$ and DCM. The aqueous layer was extracted three times with DCM. The combined organic layers were washed twice with brine, dried over $MgSO_4$, and evaporated to dryness. The crude material was purified by silica gel column chromatography (2 % methanol in DCM) to give 1-methyl-2-(1*H*-pyrazol-3-yl)-1,2,3,6-tetrahydropyridine (551 mg, 3.38 mmol, 55 %) as a brown oil. ^{1}**H NMR** (500 MHz, $CDCl_3$) δ 7.54(d, J = 2.1 Hz, 1H), 6.29 (d, J = 2.1 Hz, 1H), 5.86 (ddd, J = 9.9 Hz, 5.0 Hz, 2.5 Hz, 1H), 5.75 (ddd, J = 10.2 Hz, 2.0 Hz, 1.9 Hz, 1H), 3.84 (dd, J = 7.9 Hz, 5.0 Hz, 1H), 3.33 (d, J = 6.9 Hz, 1H), 3.14 (ddd, J = 16.9 Hz, 2.9 Hz, 2.9 Hz, 1H), 2.55 (ddd, J = 18.0 Hz, 5.5 Hz, 2.9 Hz, 1H), 2.47 (ddd, J = 17.9 , 4.5 Hz, 2.1 Hz, 1H), 2.22 (s, 3H). ^{13}**C NMR** (101 MHz, $CDCl_3$) δ 146.9, 134.9, 124.8, 124.5, 104.7, 56.9, 53.6, 42.2, 31.8. **HRMS** (ESI/Q-TOF) m/z [M^+] *calcd* 163.1109 *found* 163.1111.

Step 3 Adapted from a reported procedure [18]. 1-methyl-2-(1*H*-pyrazol-3-yl)-1,2,3,6-tetrahydropyridine (596 mg, 3.65 mmol, 1.00 equiv) was dissolved in methanol (35 mL). Pd/C 10 % wt (456 mg, 0.420 mmol, 0.10 equiv) was added. The reaction mixture was placed under H_2 atmosphere and stirred overnight at room temperature. The next day, the reaction mixture was filtered over celite and the filter cake was thoroughly washed with methanol. The filtrate was concentrated under reduced pressure. The almost clean crude product was purified by silica gel column chromatography (2 % to 5 % methanol in dichloromethane) to give 1-methyl-2-(1*H*-pyrazol-3-yl)piperidine (417 mg, 2.52 mmol, 69 %) as a dark yellow oil. ^{1}**H NMR** (400 MHz, $CDCl_3$) δ 7.52 (s, 1H), 6.30 (s, 1H), 3.32 (dd, J = 9.7 Hz, 4.3 Hz, 1H), 3.17 (d, J = 11.8 Hz, 1H), 2.29 (td, J = 11.7, 3.4 Hz, 1H), 2.14 (s, 3H), 1.91 – 1.69 (m, 5H), 1.49 – 1.34 (m, 1H). ^{13}**C NMR** (101 MHz, $CDCl_3$) δ 148.4, 135.0, 104.1, 63.1, 57.0, 43.8, 33.6, 25.3, 24.2. **HRMS** (ESI/Q-TOF) m/z [M^+] *calcd* 165.1266 *found* 165.1267.

3-bromo-4-methoxy-1*H*-pyrazole (6b-SM)

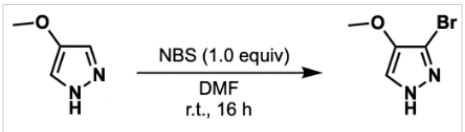

6b-SM

Following a reported procedure [19]. To a solution of 4-methoxy-1*H*-pyrazole (500 mg, 5.10 mmol, 1.00 equiv) in DMF (25 mL) was added *N*-bromosuccinimide (907 mg, 5.10 mmol, 1.00 equiv). The reaction mixture was stirred at room temperature for 16 h. The solvent was evaporated. The residue was quenched with saturated aqueous $Na_2S_2O_3$ and the aqueous layer was extracted three times with DCM. The combined organic layers were washed with brine, dried over $MgSO_4$, and evaporated to dryness. The crude material was purified by silica gel column chromatography (5 % to 10 % ethyl acetate in n-hexane) to give 3-bromo-4-methoxy-1*H*-pyrazole (503 mg, 2.84 mmol, 56 %) as a white solid. ^{1}**H NMR** (400 MHz, $CDCl_3$) δ 9.64 (br s), 7.24 (s, 1H), 3.83 (s, 3H).^{13}**C NMR** (101 MHz, $CDCl_3$) δ 144.1, 115.1, 114.6, 59.8. **HRMS** (ESI/Q-TOF) m/z [M^+] *calcd* 175.9585 *found* 175.9583.

3-iodo-4-methoxy-1*H*-pyrazole (6c-SM)

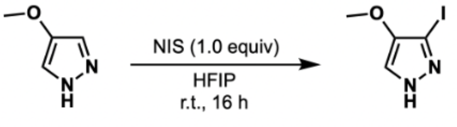

6c-SM

Adapted from a reported procedure [20]. 4-methoxy-1*H*-pyrazole (400 mg, 4.08 mmol, 1.00 equiv) and *N*-iodosuccinimide (918 mg, 4.08 mmol, 1.00 equiv) were stirred in HFIP (15 mL) at room temperature for 16 h.

The solvent was evaporated and the crude material was purified by silica gel column chromatography (5 % to 10 % ethyl acetate in n-hexane) to give 3-iodo-4-methoxy-1*H*-pyrazole (731 mg, 3.26 mmol, 80 %) as a white solid. ^{1}**H NMR** (400 MHz, $CDCl_3$) δ 7.19 (s, 1H), 3.83 (s, 3H).^{13}**C NMR** (101 MHz, $CDCl_3$) δ 148.3, 114.1, 84.5, 59.7. **HRMS** (ESI/Q-TOF) m/z [M^+] *calcd* 223.9447 *found* 223.9447.

4-bromo-3-isopropyl-1*H*-pyrazole (6h-SM)

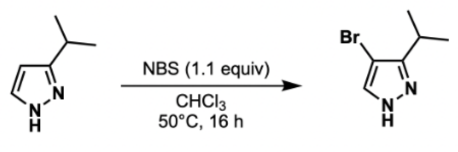

Adapted from a reported procedure [21]. To a solution of 3-isopropyl-1*H*-pyrazole (700 mg, 6.35 mmol, 1.00 equiv) in chloroform (20 mL) was added *N*-bromosuccinimide (1.21 mg, 6.79 mmol, 1.10 equiv) and the reaction mixture was stirred at 50°C for 16 h. The solvent was evaporated. The residue was partitioned between water and DCM. The aqueous layer was extracted three times with DCM. The combined organic layers were washed with brine, dried over $MgSO_4$, and evaporated to dryness. The crude material was purified by silica gel column chromatography (5 % to 10 % ethyl acetate in n-hexane) to give 4-bromo-3-isopropyl-1*H*-pyrazole (473 mg, 2.50 mmol, 39 %) as a brown oil. ^{1}**H NMR** (400 MHz, $CDCl_3$) δ 9.97 (s, 1H), 7.49 (s, 1H), 3.13 (hept, J = 7.0 Hz, 1H), 1.31 (d, J = 7.1 Hz, 6H). ^{13}**C NMR** (101 MHz, $CDCl_3$) δ 149.9, 136.6, 91.9, 25.8, 21.5. **HRMS** (ESI/Q-TOF) m/z [M^+] *calcd* 187.9949 *found* 187.9949.

4-iodo-3-isopropyl-1*H*-pyrazole (6i-SM)

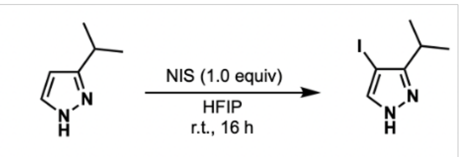

3-isopropyl-*1H*-pyrazole (600 mg, 5.45 mmol, 1.00 equiv) was dissolved in HFIP (20 mL). *N*-iodosuccinimide (1.23 g, 5.45 mmol, 1.00 equiv) was added and the reaction mixture was stirred at room temperature for 16 h. The solvent was evaporated, and the crude material was purified by silica gel chromatography (5% to 10% ethyl acetate in n-hexane) to give 4-iodo-3-isopropyl-1*H*-pyrazole (709 mg, 3.00 mmol, 55%) as a yellow oil. ^{1}**H NMR** (400 MHz, $CDCl_3$) δ 11.68 (br s), 7.52 (s, 1H), 3.09 (hept, J = 7.1 Hz, 1H), 1.31 (d, J = 7.0 Hz, 6H). ^{13}**C NMR** (75 MHz, $CDCl_3$) δ 153.4, 141.2, 57.1, 26.9, 21.7. **HRMS** (ESI/Q-TOF) m/z [M^+] *calcd* 235.9810 *found* 235.9811.

1-(4-bromo-1*H*-pyrazol-3-yl)ethan-1-one (6j-SM)

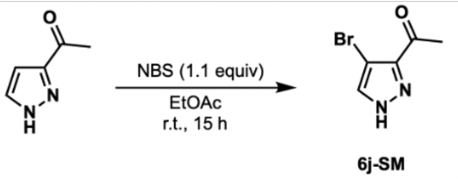

Adapted from a reported procedure [22]. A solution of 1-(1*H*-pyrazol-3-yl)ethan-1-one (700 mg, 6.36 mmol, 1.00 equiv) and *N*-bromosuccinimide (2.04 g, 11.5 mmol, 1.80 equiv) in ethyl acetate (26 mL) was stirred at room temperature for 21 h. The reaction mixture was quenched with saturated aqueous $Na_2S_2O_3$. The aqueous layer was extracted three times with ethyl acetate. The combined organic layers were washed with brine, dried over $MgSO_4$, and evaporated to dryness. The crude material was purified by silica gel column

chromatography (10 % to 20 % ethyl acetate in n-hexane) to give 1-(4-bromo-1*H*-pyrazol-3-yl)ethan-1-one (564 mg, 2.98 mmol, 47 %). **^{1}H NMR** (400 MHz, CD_3OD) δ 7.83 (s, 1H), 2.56 (s, 3H). **^{13}C NMR** (101 MHz, CD_3OD) δ 195.2, 143.1, 133.1, 94.7, 27.3. **HRMS** (ESI/Q-TOF) m/z [M$^+$] *calcd* 187.9585 *found* 187.9581.

1-(4-iodo-1*H*-pyrazol-3-yl)ethan-1-one (6l-SM)

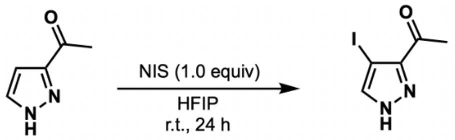

6l-SM

Adapted from a reported procedure [20]. 1-(1*H*-pyrazol-3-yl)ethan-1-one (600 mg, 5.45 mmol, 1.00 equiv) and *N*-iodosuccinimide (1.23 mg, 5.45 mmol, 1.00 equiv) were stirred in HFIP (20 mL) at room temperature for 24 h. The solvent was evaporated and the crude material was purified by silica gel column chromatography (5 % to 15 % ethyl acetate in n-hexane) to give 1-(4-iodo-1*H*-pyrazol-3-yl)ethan-1-one (530 mg, 2.25 mmol, 41 %) as an off-white solid. **^{1}H NMR** (400 MHz, $CDCl_3$) δ 7.75 (s, 1H), 2.69 (s, 3H). **^{13}C NMR** (101 MHz, $CDCl_3$) δ 191.0, 145.6, 141.6, 59.7, 28.3. **HRMS** (ESI/Q-TOF) m/z [M$^+$] *calcd* 234.9447 *found* 234.9458.

Methyl 4-iodo-1*H*-pyrazole-3-carboxylate (6m-SM)

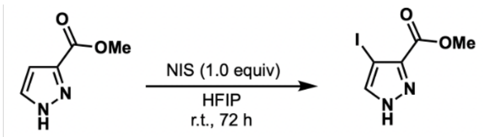

6m-SM

Adapted from a reported procedure [20]. Methyl 1*H*-pyrazole-3-carboxylate (687 mg, 5.45 mmol, 1.00 equiv) and *N*-iodosuccinimide (1.23 mg, 5.45 mmol, 1.00 equiv) were stirred in HFIP (20 mL) for 72 h. The solvent was evaporated and the crude material was purified by silica gel column chromatography (10 % ethyl acetate in n-hexane) to give methyl 4-iodo-1*H*-pyrazole-3-carboxylate (589 mg, 2.34 mmol, 43 %) as a white solid. **^{1}H NMR** (400 MHz, CD_3CN) δ 7.78 (s, 1H), 3.86 (s, 3H). **HRMS** (ESI/Q-TOF) m/z [M$^+$] *calcd* 251.9396 *found* 251.9396. The data is consistent with the literature [23].

***N*-(1,3,4-thiadiazol-2-yl)acetamide (8-SM)**

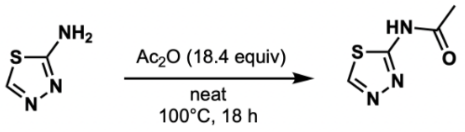

8-SM

Following a reported procedure [24], 1,3,4-thiadiazol-2-amine (500 mg, 4.94 mmol, 1.00 equiv) was mixed with Ac_2O (10.0 mL, 9.26 g, 90.7 mmol, 18.4 equiv). The reaction mixture was stirred at 100°C for 18 h. The white precipitate was filtered off and recrystallized from hot ethanol. *N*-(1,3,4-thiadiazol-2-yl)acetamide (495 mg, 3.46 mmol, 70 %) was obtained as white, needle-shaped crystals. **^{1}H NMR** (400 MHz, DMSO-d_6) δ 12.54 (s, 1H), 9.14 (s, 1H), 2.19 (s, 3H). **^{13}C NMR** (101 MHz, DMSO-d_6) δ 168.6, 158.5, 148.5, 22.4. The data is consistent with the literature [25].

4. Trifluoromethylation

4.1. General procedure A for the Trifluoromethylation of Pyrazoles

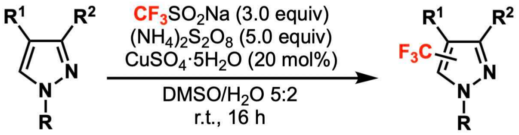

A reaction vial was charged with pyrazole substrate (0.500 mmol, 1.00 equiv), CF_3SO_2Na (234 mg, 1.50 mmol, 3.00 equiv) and $CuSO_4{\cdot}5H_2O$ (25.0 mg, 0.100 mmol, 0.200 equiv). DMSO (2.0 mL) and water (0.8 mL) were added. Lastly, $(NH_4)_2S_2O_8$ (570 mg, 2.50 mmol, 5.00 equiv) was added portionwise. The reaction mixture was stirred at room temperature for 16 h. In some cases, a different reaction scale is specified, but the ratios of the reagents remain unchanged.

Work-up AA The reaction mixture was diluted with ethyl acetate and saturated aqueous $NaHCO_3$. The aqueous layer was extracted three times with ethyl acetate. The combined organic layers were washed once with distilled water and once with brine, dried over $MgSO_4$, and evaporated to dryness.

Work-up AB The reaction mixture was diluted with ethyl acetate and 25 % aqueous ammonia solution. The aqueous layer was extracted three times with ethyl acetate. The combined organic layers were washed once with 25 % aqueous ammonia solution, dried over $MgSO_4$, and evaporated to dryness.

Work-up AC The reaction mixture was diluted with distilled water. The aqueous layer was extracted three times with ethyl acetate. The combined organic layers were washed once with brine, dried over $MgSO_4$, and evaporated to dryness.

4.2. Product Characterization Data

4-methoxy-5-(trifluoromethyl)-1-((2-(trimethylsilyl)ethoxy)methyl)-1*H*-pyrazole (3a-5C-CF_3) and 4-methoxy-3-(trifluoromethyl)-1-((2-(trimethylsilyl)ethoxy)methyl)-1*H*-pyrazole (3a-3C-CF_3)

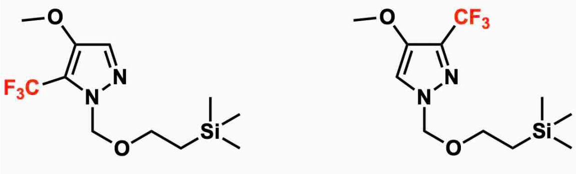

Prepared from 4-methoxy-1-((2-(trimethylsilyl)ethoxy)methyl)-1*H*-pyrazole according to General Procedure A and Work-up AA. The crude material was purified by silica gel column chromatography (2.5 % ethyl acetate in n-hexane), to give 4-methoxy-5-(trifluoromethyl)-1-((2-(trimethylsilyl)ethoxy)methyl)--1*H*-pyrazole (91 mg, 0.31 mmol, 61 %) as a light pink oil and 4-methoxy-3-(trifluoromethyl)-1-((2-(trimethylsilyl)ethoxy)methyl)-1*H*-pyrazole (18 mg, 0.06 mmol, 12 %) as a yellow oil.

4-methoxy-5-(trifluoromethyl)-1-((2-(trimethylsilyl)ethoxy)methyl)-1*H*-pyrazole
^{1}**H NMR** (400 MHz, $CDCl_3$) δ 7.33 (s, 1H), 5.46 (s, 2H), 3.84 (s, 3H), 3.62 – 3.47 (m, 2H), 0.94 – 0.85 (m, 2H), -0.04 (s, 9H). ^{13}**C NMR** (126 MHz, $CDCl_3$) δ 147.0, 125.9, 120.4 (q, $^1J_{C\text{-}F}$ = 268.4 Hz, CF_3), 116.8 (q, $^1J_{C\text{-}F}$ = 40.0 Hz, C-CF_3), 80.82 (q, J = 1.9 Hz), 66.9, 59.2, 17.8, -1.2. ^{19}**F NMR** (376 MHz, $CDCl_3$) δ - 57.94 (s, CF_3). **HRMS** (ESI/Q-TOF) m/z [M$^+$] *calcd* 296.1168 *found* 296.1169.

4-methoxy-3-(trifluoromethyl)-1-((2-(trimethylsilyl)ethoxy)methyl)-1*H*-pyrazole ^{1}**H NMR** (400 MHz, $CDCl_3$) δ 7.26 (s, 1H), 5.34 (s, 2H), 3.79 (s, 3H), 3.64 – 3.45 (m, 2H), 0.97 – 0.82 (m, 2H), -0.02 (s, 9H). ^{13}**C NMR** (101 MHz, $CDCl_3$) δ 145.3, 130.1 (q, $^2J_{C\text{-}F}$ = 37.5 Hz, C-CF_3), 121.2 (q, $^1J_{C\text{-}F}$ = 268.4 Hz, CF_3), 114.2, 81.7, 67.2, 59.5, 17.8, -1.4. ^{19}**F NMR** (376 MHz, $CDCl_3$) δ - 61.78 (s, CF_3).**HRMS** (ESI/Q-TOF) m/z [M$^+$] *calcd* 296.1168 *found* 296.1169.

4-methyl-5-(trifluoromethyl)-1-((2-(trimethylsilyl)ethoxy)methyl)-1*H*-pyrazole (3b-5C-CF_3)

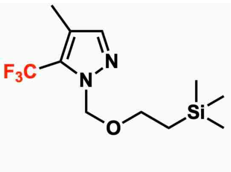

3b-C5-CF_3

Prepared from 4-methyl-1-((2-(trimethylsilyl)ethoxy)methyl)-1*H*-pyrazole according to General Procedure A and Work-up AA. The crude material was purified by silica gel column chromatography (0 % to 1 % ethyl acetate in n-hexane) to give 4-methyl-5-(trifluoromethyl)-1-((2-(trimethylsilyl)ethoxy)methyl)-1*H*-pyrazole (48 mg, 0.17 mmol, 34 %). **^{1}H NMR** (400 MHz, $CDCl_3$) δ 7.35 (s, 1H), 5.51 (s, 2H), 3.60 – 3.48 (m, 2H), 2.18 (q, J = 2.0 Hz, 3H), 0.95 – 0.83 (m, 2H), -0.04 (s, 9H). **^{13}C NMR** (126 MHz, $CDCl_3$) δ 140.5, 128.6 (q, $^{2}J_{C-F}$ = 38.1 Hz, C-CF_3), 121.1 (q, $^{1}J_{C-F}$ = 269.8 Hz, CF_3), 120.2 (q, $^{3}J_{C-F}$ = 1.8 Hz), 80.0 (q, $^{4}J_{C-F}$ = 1.6 Hz), 67.0, 17.9, 9.0 (q, $^{4}J_{C-F}$ = 1.9 Hz), -1.4. **^{19}F NMR** (376 MHz, $CDCl_3$) δ - 57.41 (s, CF_3). **HRMS** (ESI/Q-TOF) m/z [M^+] *calcd* 280.1219 *found* 280.1225.

1-((perfluorophenyl)methyl)-5-(trifluoromethyl)-1*H*-pyrazole (3c-C5-CF_3) and 1-((perfluorophenyl)methyl)-3-(trifluoromethyl)-1*H*-pyrazole (3c-C3-CF_3)

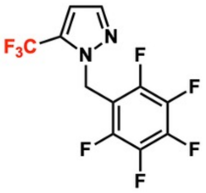

3c-C5-CF_3

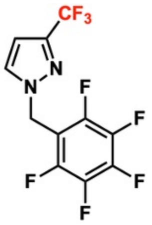

3c-C3-CF_3

Prepared from 1-((perfluorophenyl)methyl)-1*H*-pyrazole according to General Procedure A and Work-up AA. The crude material was purified by silica gel column chromatography (1 % to 2.5 % ethyl acetate in n-hexane) to give 1-((perfluorophenyl)methyl)-5-(trifluoromethyl)-1*H*-pyrazole (67 mg, 0.21 mmol, 42 %) as a yellow oil and 1-((perfluorophenyl)methyl)-3-(trifluoromethyl)-1*H*-pyrazole (30 mg, 0.09 mmol, 19 %) as a yellow oil.

1-((perfluorophenyl)methyl)-5-(trifluoromethyl)-1*H*-pyrazole **^{1}H NMR** (400 MHz, $CDCl_3$) δ 7.51 (d, J = 1.4 Hz, 1H), 6.66 (d, J = 1.3 Hz, 1H), 5.46 (s, 2H). **^{13}C NMR** (126 MHz, $CDCl_3$) δ 145.9 (dm, $^{1}J_{C-F}$ = 250.9 Hz), 142.0 (dm, $^{1}J_{C-F}$ = 257.8 Hz), 139.5, 137.2 (dm, $^{1}J_{C-F}$ = 257.1 Hz), 132.3 (q, $^{2}J_{C-F}$ = 39.5 Hz, C-CF_3), 120.2 (q, $^{1}J_{C-F}$ = 268.9 Hz, CF_3), 108.6 (td, $^{2}J_{C-F}$ = 17.0 Hz, $^{3}J_{C-F}$ = 4.4 Hz), 108.3 (d_{app}, $^{3}J_{C-F}$ = 2.3 Hz), 41.9. **^{19}F NMR** (376 MHz, $CDCl_3$) δ -59.42 (s, CF_3), -142.04 (d, $^{3}J_{F-F}$ = 19.1 Hz), -152.22 (t, $^{3}J_{F-F}$ = 20.8 Hz), -161.23 (td, $^{3}J_{F-F}$ = 19.9 Hz, $^{4}J_{F-F}$ = 6.1 Hz). **HRMS** (ESI/Q-TOF) m/z [M^-] *calcd* 316.0247 *found* 316.0251.

1-((perfluorophenyl)methyl)-3-(trifluoromethyl)-1*H*-pyrazole **^{1}H NMR** (400 MHz, $CDCl_3$) δ 7.54 (s, 1H), 6.54 (d, J = 2.4 Hz, 1H), 5.44 (s, 2H). **^{13}C NMR** (126 MHz, $CDCl_3$) δ 145.6 (dm, $^{1}J_{C-F}$ = 257.7 Hz), 143.5 (q, $^{3}J_{C-F}$ = 38.6 Hz, C-CF_3), 142.3 (dm, $^{1}J_{C-F}$ = 257.4 Hz,), 137.9 (dt, $^{1}J_{C-F}$ = 255.1 Hz, $^{2}J_{C-F}$ = 15.1 Hz), 130.9, 121.1 (q, $^{1}J_{C-F}$ = 268.4 Hz, CF_3), 109.0 (td, $^{2}J_{C-F}$ = 17.7 Hz, $^{3}J_{C-F}$ = 4.8 Hz), 105.4 (q, $^{3}J_{C-F}$ = 2.1 Hz), 43.5. **^{19}F NMR** (376 MHz, $CDCl_3$) δ -62.10 (s, CF_3), -142.01 – -142.16 (m), -151.60 (tt, $^{3}J_{F-F}$ = 21.2 Hz, $^{4}J_{F-F}$ = 2.3 Hz), -160.41 (td, $^{3}J_{F-F}$ = 19.9 Hz, $^{4}J_{F-F}$ = 6.9 Hz). **HRMS** (ESI/Q-TOF) m/z [M^-] *calcd* 316.0247 *found* 316.0248.

4-methoxy-5-(trifluoromethyl)-1*H*-pyrazole (4a-monoCF_3) and 4-methoxy-3,5-bis(trifluoromethyl)-1*H*-pyrazole (4a-diCF_3)

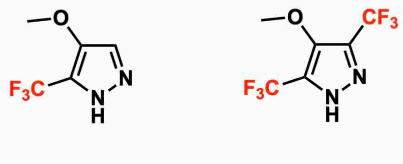

4a-monoCF_3 **4a-diCF_3**

Prepared from commercial 4-methoxy-1*H*-pyrazole according to General Procedure A and Work-up AA. The crude material was purified by silica gel column chromatography (10 % ethyl acetate in n-hexane), giving 4-methoxy-5-(trifluoromethyl)-1*H*-pyrazole (28 mg, 0.17 mmol, 33 %) as a white solid and 4-methoxy-3,5-bis(trifluoromethyl)-1*H*-pyrazole (37 mg, 0.16 mmol, 32 %) as a white solid.
4-methoxy-5-(trifluoromethyl)-1*H*-pyrazole (4a-monoCF_3) ^{1}**H NMR** (400 MHz, $CDCl_3$) δ 9.53 (br s), 7.31 (q, $^5J_{H\text{-}F} = 1.1$ Hz, 1H), 3.85 (s, 3H). ^{13}**C NMR** (126 MHz, $CDCl_3$) δ 144.4, 129.5 (q, $^2J_{C\text{-}F} = 37.7$ Hz, $\underline{C}$-CF_3), 121.5 (q, $J = 268.2$ Hz, CF_3), 114.4, 59.7. ^{19}**F NMR** (376 MHz, $CDCl_3$) δ -61.53 (s, CF_3). **HRMS** (ESI/Q-TOF) m/z [M^+] *calcd* 166.0354 *found* 166.0350.
4-methoxy-3,5-bis(trifluoromethyl)-1*H*-pyrazole (4a-diCF_3) ^{1}**H NMR** (400 MHz, $CDCl_3$) δ 10.63 (br s), 3.91 (s, 3H). ^{13}**C NMR** (126 MHz, $CDCl_3$) δ 141.7, 129.5 (m, $\underline{C}$-CF_3), 119.7 (q, $^1J_{C\text{-}F} = 269.1$ Hz, CF_3), 63.6. ^{19}**F NMR** (376 MHz, $CDCl_3$) δ -60.89 (s, CF_3). **HRMS** (ESI/Q-TOF) m/z [M^-] *found* 234.0228 *calcd* 234.0229.

4-methyl-5-(trifluoromethyl)-1*H*-pyrazole (4b-monoCF_3) and 4-methyl-3,5-bis(trifluoromethyl)-1*H*-pyrazole (4b-diCF_3)

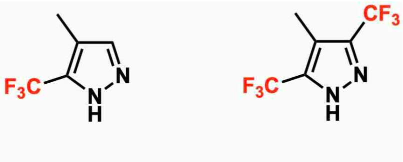

4b-monoCF_3 **4b-diCF_3**

Prepared from commercial 4-methyl-1*H*-pyrazole according to General Procedure A and Work-up AA. The crude material was purified by silica gel column chromatography (5 % ethyl acetate in n-hexane) to give 4-methyl-5-(trifluoromethyl)-1*H*-pyrazole (15 mg, 0.10 mmol, 20 %) as a white solid and 4-methyl-3,5-bis(trifluoromethyl)-1*H*-pyrazole (28 mg, 0.13 mmol, 25 %) as a white solid.
4-methyl-5-(trifluoromethyl)-1*H*-pyrazole (4b-monoCF_3) ^{1}**H NMR** (400 MHz, $CDCl_3$) δ 12.62 (s, 1H), 7.46 (s, 1H), 2.21 (s, 3H). ^{13}**C NMR** (126 MHz, $CDCl_3$) δ 140.4 (q, $^2J_{C\text{-}F} = 35.6$ Hz, $\underline{C}$-CF_3), 129.9, 122.4 (q, $^1J_{C\text{-}F} = 268.9$ Hz, CF_3), 115.0, 8.0. ^{19}**F NMR** (376 MHz, $CDCl_3$) δ -61.42 (s, CF_3). **HRMS** (ESI/Q-TOF) m/z [M^+] *calcd* 150.0405 *found* 150.0405. The data is consistent with the literature [26].
4-methyl-3,5-bis(trifluoromethyl)-1*H*-pyrazole (4b-diCF_3) ^{1}**H NMR** (400 MHz, $CDCl_3$) δ 11.04 (br s), 2.28 (hept, $^5J_{H\text{-}F} = 1.1$ Hz, 3H). ^{13}**C NMR** (126 MHz, $CDCl_3$) δ 139.2 – 136.1 (m), 120.3 (q, $^1J_{C\text{-}F} = 269.3$ Hz, CF_3), 116.2, 6.9. ^{19}**F NMR** (376 MHz, $CDCl_3$) δ -61.30 (s, CF_3).

Methyl 3-(trifluoromethyl)-1*H*-pyrazole-4-carboxylate (5c-monoCF_3)

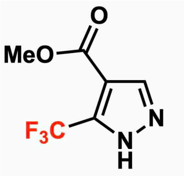

5c-monoCF_3

Prepared from commercial methyl 1*H*-pyrazole-4-carboxylate according to General Procedure A and Work-up AA. The crude material was purified by silica gel column chromatography (5 % ethyl acetate in n-hexane), giving methyl 3-(trifluoromethyl)-1*H*-pyrazole-4-carboxylate (51 mg, 0.25 mmol, 49 %) as a white solid. ^{1}**H NMR** (400 MHz, DMSO-d_6) δ 8.22 (s, 1H), 3.90 (s, 3H). ^{13}**C NMR** (126 MHz, DMSO-d_6) δ 153.6, 133.3 (q, $^{2}J_{C\text{-}F}$ = 40.0 Hz, C-CF$_3$), 127.0, 112.69 (q, $^{1}J_{C\text{-}F}$ = 268.4 Hz, CF$_3$), 103.5, 42.7. ^{19}**F NMR** (376 MHz, DMSO-d_6) δ -62.18 (s, CF$_3$). **HRMS** (ESI/Q-TOF) m/z [M$^+$] *calcd* 194.0303 *found* 194.0303.

1-(5-(trifluoromethyl)-1*H*-pyrazol-4-yl)ethan-1-one (5d-monoCF$_3$) and 1-(3,5-bis(trifluoromethyl)-1*H*-pyrazol-4-yl)ethan-1-one (5d-diCF$_3$)

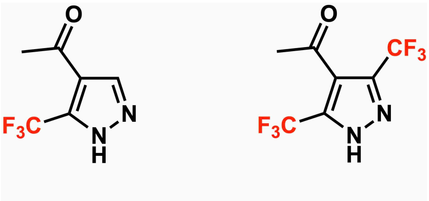

5d-monoCF$_3$ 5d-diCF$_3$

Prepared from commercial 1-(1*H*-pyrazol-4-yl)ethan-1-one according to General Procedure A and Work-up AA. The crude material was purified by silica gel column chromatography (10 % to 30 % ethyl acetate in n-hexane), giving 1-(5-(trifluoromethyl)-1*H*-pyrazol-4-yl)ethan-1-one (40 mg, 0.23 mmol, 45 %) as a white solid and 1-(3,5-bis(trifluoromethyl)-1*H*-pyrazol-4-yl)ethan-1-one (26 mg, 0.11 mmol, 21 %) as a pale yellow oil.

1-(5-(trifluoromethyl)-1*H*-pyrazol-4-yl)ethan-1-one (5d-monoCF$_3$) ^{1}**H NMR** (400 MHz, CD$_3$CN) δ 11.77 (bs, 1H), 8.32 (q, $^{5}J_{H\text{-}F}$ = 1.1 Hz, 1H), 2.44 (s, 3H). ^{13}**C NMR** (126 MHz, CD$_3$CN) δ 191.1, 140.9 (q, $^{2}J_{C\text{-}F}$ = 37.7 Hz, C-CF$_3$), 136.4, 122.0 (q, $^{1}J_{C\text{-}F}$ = 267.9 Hz, CF$_3$), 121.2, 28.8. ^{19}**F NMR** (376 MHz, CD$_3$CN) δ -62.96 (s, CF$_3$). **HRMS** (ESI/Q-TOF) m/z [M$^+$] *calcd* 178.0354 *found* 178.0358.

1-(3,5-bis(trifluoromethyl)-1*H*-pyrazol-4-yl)ethan-1-one (5d-diCF$_3$) ^{1}**H NMR** (400 MHz, CDCl$_3$) δ 2.60 (s, 3H). ^{13}**C NMR** (126 MHz, CDCl$_3$) δ 192.5, 139.2 – 136.1 (m, C-CF$_3$), 120.5, 119.4 (q, $^{1}J_{C\text{-}F}$ = 270.2 Hz, CF$_3$), 30.8. ^{19}**F NMR** (376 MHz, CDCl$_3$) δ -60.40 (s, CF$_3$). **HRMS** (ESI/Q-TOF) m/z [M$^-$] *calcd* 246.0228 *found* 246.0228.

4-nitro-3-(trifluoromethyl)-1*H*-pyrazole (5e-monoCF$_3$)

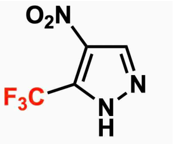

5e-monoCF$_3$

Prepared from commercial 4-nitro-1*H*-pyrazole according to General procedure A and Work-up AA. The crude material was purified by silica gel column chromatography (5 % to 20 % ethyl acetate in n-hexane) to give 4-nitro-3-(trifluoromethyl)-1*H*-pyrazole (30 mg, 0.17 mmol, 33 %) as a white solid. ^{1}**H NMR** (300 MHz, CDCl$_3$) δ 8.50 – 8.48 (m, 1H). ^{13}**C NMR** (75 MHz, CDCl$_3$) δ 136.9 (q, $^{2}J_{C\text{-}F}$ = 40.1 Hz, C-CF$_3$), 133.3, 131.3, 119.3 (q, $^{1}J_{C\text{-}F}$ = 270.0 Hz, CF$_3$). ^{19}**F NMR** (282 MHz, CDCl$_3$) δ -62.69 (s, CF$_3$). **HRMS** (ESI/Q-TOF) m/z [M$^-$] *calcd* 180.0099 *found* 180.0100. The ^{1}H NMR data is consistent with the literature [27].

3-(trifluoromethyl)-1*H*-pyrazole-4-carboxylic acid (5f)

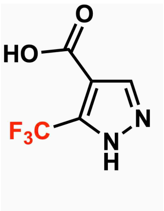

5f-monoCF_3

Prepared from commercial 1*H*-pyrazole-4-carboxylic acid according to General Procedure A and Work-up AC. The crude material was purified by silica gel column chromatography (25 % ethyl acetate in n-hexane) to give 3-(trifluoromethyl)-1*H*-pyrazole-4-carboxylic acid (36 mg, 0.20 mmol, 39 %) as a white solid. ^{1}**H NMR** (400 MHz, CD_3CN) δ 8.22 (q, J = 1.1 Hz, 1H). ^{13}**C NMR** (126 MHz, DMSO-d_6) δ 162.1, 140.4 (q, $^{2}J_{C\text{-}F}$ = 37.7 Hz, C-CF_3), 135.9, 121.0 (q, $^{1}J_{C\text{-}F}$ = 268.6 Hz, CF_3), 112.6. ^{19}**F NMR** (376 MHz, CD_3CN) δ -60.70 (s, CF_3). **HRMS** (ESI/Q-TOF) m/z [M$^-$] *calcd* 180.0147 *found* 180.0148.

1-(5-(trifluoromethyl)-1H-pyrazol-3-yl)ethan-1-one (5g-5C-CF_3) and 1-(4-(trifluoromethyl)-1*H*-pyrazol-3-yl)ethan-1-one (5g-4C-CF_3)

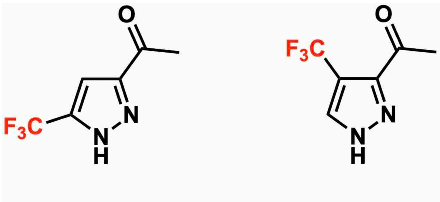

5g-C5-CF_3 5g-C4-CF_3

Prepared from commercial 1-(1*H*-pyrazol-3-yl)ethan-1-one according to General Procedure A and Work-up AA. The crude material was purified by silica gel column chromatography (10 % to 30 % ethyl acetate in n-hexane), giving 1-(5-(trifluoromethyl)-1*H*-pyrazol-4-yl)ethan-1-one (22 mg, 0.12 mmol, 25 %) as a white solid and 1-(4-(trifluoromethyl)-1*H*-pyrazol-4-yl)ethan-1-one (28 mg, 0.16 mmol, 31 %) as a white solid.
1-(5-(trifluoromethyl)-1*H*-pyrazol-3-yl)ethan-1-one (5g-C5-CF_3) ^{1}**H NMR** (400 MHz, $CDCl_3$) δ 7.06 (s, 1H), 2.58 (s, 3H). ^{13}**C NMR** (126 MHz, $CDCl_3$) δ 188.3, 144.2 (d_{app}, $^{2}J_{C\text{-}F}$ = 38.8 Hz, C-CF_3), 142.0, 120.6 (q, $^{1}J_{C\text{-}F}$ = 268.9 Hz, CF_3), 107.2, 27.2. ^{19}**F NMR** (376 MHz, $CDCl_3$) δ - 62.35 (s, CF_3). **HRMS** (ESI/Q-TOF) m/z [M$^-$] *calcd* 178.0354 *found* 178.0355.
1-(4-(trifluoromethyl)-1*H*-pyrazol-3-yl)ethan-1-one (5g-C4-CF_3) ^{1}**H NMR** (400 MHz, CD_3CN) δ 8.07 (s, 1H), 2.55 (s, 3H). ^{13}**C NMR** (126 MHz, CD_3CN) δ 193.5, 148.5, 132.8, 123.5 (q, $^{1}J_{C\text{-}F}$ = 265.5 Hz, CF_3), 112.5 (q, $^{1}J_{C\text{-}F}$ = 39.1 Hz, C-CF_3), 27.3. ^{19}**F NMR** (376 MHz, $CDCl_3$) δ -52.68 (s, CF_3). **HRMS** (ESI/Q-TOF) m/z [M$^-$] *calcd* 178.0354 *found* 178.0350.

Methyl 5-(trifluoromethyl)-1*H*-pyrazole-3-carboxylate (5h-5C-CF_3) and methyl 4-(trifluoromethyl)-1*H*-pyrazole-3-carboxylate (5h-4C-CF_3)

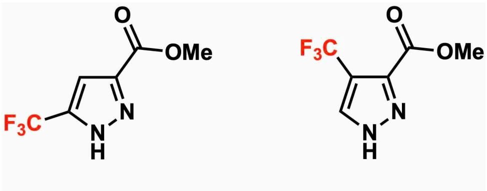

5h-C5-CF_3 5h-C4-CF_3

Prepared from commercial methyl 1*H*-pyrazole-3-carboxylate according to General Procedure A and Work-up AA. The crude material was purified by silica gel column chromatography (5 % ethyl acetate in n-hexane),

giving methyl 5-(trifluoromethyl)-1*H*-pyrazole-3-carboxylate (31 mg, 0.16 mmol, 32 %) as a light brown solid and methyl 4-(trifluoromethyl)-1*H*-pyrazole-3-carboxylate (24 mg, 0.12 mmol, 25 %) as a light brown solid.

Methyl 5-(trifluoromethyl)-1*H*-pyrazole-3-carboxylate ^{1}**H NMR** (400 MHz, $CDCl_3$) δ 7.10 (s, 1H), 3.98 (s, 3H). ^{13}**C NMR** (126 MHz, $CDCl_3$) δ 159.2, 144.3 (d_{app}, $^{2}J_{C\text{-}F}$ = 36.5 Hz, C-CF_3), 135.3, 120.6 (q, $^{1}J_{C\text{-}F}$ = 268.9 Hz, CF_3), 107.6, 53.0. ^{19}**F NMR** (376 MHz, $CDCl_3$) δ -62.42 (s, CF_3). **HRMS** (ESI/Q-TOF) m/z [M^+] *calcd* 194.0303 *found* 194.0304. The data is consistent with the literature [28].

Methyl 4-(trifluoromethyl)-1*H*-pyrazole-3-carboxylate ^{1}**H NMR** (300 MHz, DMSO-d_6) δ 14.14 (s, 1H), 8.49 (s, 1H), 3.85 (s, 3H). ^{13}**C NMR** (300 MHz, DMSO-d_6) δ 160.8, 139.3, 132.0, 122.3 (q, $^{1}J_{C\text{-}F}$ = 266.2 Hz, CF_3), 112.5 (q, $^{2}J_{C\text{-}F}$ = 39.1 Hz, C-CF_3), 52.0. ^{19}**F NMR** (376 MHz, $CDCl_3$) δ -57.34 (s, CF_3). **HRMS** (ESI/Q-TOF) m/z $[M+H]^+$ *calcd* 195.0376 *found* 195.0378.

1-methyl-2-(5-(trifluoromethyl)-1*H*-pyrazol-3-yl)piperidine (5i)

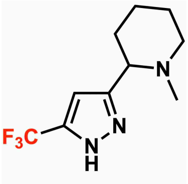

5i

Prepared from 1-methyl-2-(1*H*-pyrazol-3-yl)piperidine according to General Procedure A and Work-up AB. The crude material was purified by silica gel column chromatography (0 % to 2 % methanol in DCM) to give 1-methyl-2-(5-(trifluoromethyl)-1*H*-pyrazol-3-yl)piperidine (66 mg, 0.28 mmol, 56 %) as a brown oil. ^{1}**H NMR** (400 MHz, $CDCl_3$) δ 6.42 (s, 1H), 3.20 (t, J = 9.8 Hz, 2H), 2.24 (t, J = 11.0 Hz, 1H), 2.13 (s, 3H), 1.87 (t, J = 13.1 Hz 2H), 1.81 – 1.63 (m, 3H), 1.40 (qt, J = 11.5 Hz, 3.8 Hz, 1H). ^{13}**C NMR** (126 MHz, $CDCl_3$) δ 146.7, 142.7 (d_{app}, $^{2}J_{C\text{-}F}$ = 37.7 Hz, C-CF_3), 121.5 (q, $^{1}J_{C\text{-}F}$ = 268.6 Hz, CF_3) 102.7, 61.9, 57.0, 44.2, 34.0, 25.3, 24.2. ^{19}**F NMR** (376 MHz, $CDCl_3$) δ -61.85 (s, CF_3). **HRMS** (ESI/Q-TOF) m/z [M^+] *calcd* 233.1140 *found* 233.1141.

4-fluoro-5-(trifluoromethyl)-1*H*-pyrazole (5l)

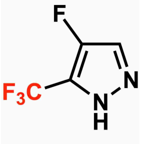

5l

Prepared from commercial 4-fluoro-1*H*-pyrazole according to General Procedure A and Work-up AA. The crude material was purified by silica gel column chromatography (5 % to 15 % ethyl acetate in n-hexane) to give 4-fluoro-5-(trifluoromethyl)-1*H*-pyrazole (34 mg, 0.22 mmol, 44 %) as a white solid. ^{1}**H NMR** (400 MHz, $CDCl_3$) δ 12.74 (br s), 7.56 (dd, J = 4.6 Hz, 0.9 Hz, 1H). ^{13}**C NMR** (126 MHz, $CDCl_3$) δ 146.4 (d, $^{1}J_{C\text{-}F}$ = 254.6 Hz), 129.3 (qd, $^{2}J_{C\text{-}F}$ = 38.9 Hz, $^{2}J_{C\text{-}F}$ = 9.2 Hz, C-CF_3), 120.5 (qd, $^{1}J_{C\text{-}F}$ = 268.4 Hz, $^{3}J_{C\text{-}F}$ = 3.2 Hz, CF_3), 117.2 (d, $^{2}J_{C\text{-}F}$ = 25.7 Hz). ^{19}**F NMR** (376 MHz, $CDCl_3$) δ -61.54 (d, $^{4}J_{F\text{-}F}$ = 6.1 Hz, CF_3), -174.10 (quint, $^{4}J_{F\text{-}F}$ = 6.5 Hz). **HRMS** (ESI/Q-TOF) m/z [M^-] *calcd* 154.0154 *found* 154.0154.

4-bromo-3-methoxy-5-(trifluoromethyl)-1*H*-pyrazole (6a)

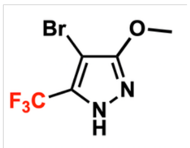

6a

Prepared from 3-bromo-4-methoxy-1*H*-pyrazole according to General Procedure A and Work-up AA. The crude material was purified by silica gel flash chromatography (0 % to 10 % ethyl acetate in n-heptane) to give 3-bromo-4-methoxy-5-(trifluoromethyl)-1*H*-pyrazole (102 mg, 0.42 mmol, 83 %) as a light brown solid. ^{1}**H NMR** (400 MHz, $CDCl_3$) δ 4.01 (s, 3H). ^{13}**C NMR** (126 MHz, $CDCl_3$) δ 160.6, 133.0 (q, $^{2}J_{C\text{-}F}$ = 38.9 Hz, C-CF_3), 119.2 (q, $^{1}J_{C\text{-}F}$ = 269.8 Hz, CF_3), 80.1, 57.6. ^{19}**F NMR** (376 MHz, $CDCl_3$) δ -62.53 (s, CF_3). **HRMS** (ESI/Q-TOF) m/z $[M+H]^+$ *calcd* 244.9532 *found* 244.9509.

3-bromo-4-methoxy-5-(trifluoromethyl)-1*H*-pyrazole (6b)

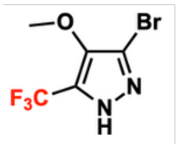

6b

Prepared from 3-bromo-4-methoxy-1*H*-pyrazole according to General Procedure A and Work-up AA. The crude material was purified by silica gel column chromatography (5 % ethyl acetate in n-hexane) to give 3-bromo-4-methoxy-5-(trifluoromethyl)-1*H*-pyrazole (100 mg, 0.41 mmol, 81 %) as a white solid. ^{1}**H NMR** (400 MHz, $CDCl_3$) δ 3.91 (s, 3H). ^{13}**C NMR** (126 MHz, $CDCl_3$) δ 141.3, 130.5 (d_{app}, $^{2}J_{C\text{-}F}$ = 39.1 Hz, C-CF_3), 119.9 (q, $^{1}J_{C\text{-}F}$ = 269.3 Hz, CF_3), 110.9, 61.8. ^{19}**F NMR** (376 MHz, $CDCl_3$) δ -61.57 (s, CF_3). **HRMS** (ESI/Q-TOF) m/z $[M^-]$ *calcd* 243.9459 *found* 243.9461.

3-iodo-4-methoxy-5-(trifluoromethyl)-1*H*-pyrazole (6c)

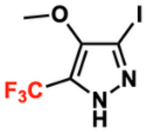

6c

Prepared from 3-iodo-4-methoxy-1*H*-pyrazole according to General Procedure A and Work-up AA. The crude material was purified by silica gel column chromatography (5 % ethyl acetate in n-hexane) to give 3-iodo-4-methoxy-5-(trifluoromethyl)-1*H*-pyrazole (117 mg, 0.40 mmol, 80 %) as a white solid. ^{1}**H NMR** (400 MHz, $CDCl_3$) δ 10.82 (br s), 3.88 (s, 3H). ^{13}**C NMR** (75 MHz, DMSO-*d*$_6$) 145.0, 132.3 (q, $^{2}J_{C\text{-}F}$ = 36.2 Hz, CF_3), 120.9 (q, $^{1}J_{C\text{-}F}$ = 268.4 Hz, CF_3), 76.0, 62.9. ^{19}**F NMR** (376 MHz, $CDCl_3$) δ -61.72 (s, CF_3). **HRMS** (ESI/Q-TOF) m/z $[M^-]$ *calcd* 291.9320 *found* 291.9321.

3-(trifluoromethyl)-6,7-dihydro-5*H*-pyrazolo[5,1-*b*][1,3]oxazine-2-carboxylic acid (6d)

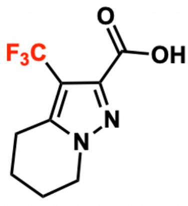

6d

Prepared from commercial 6,7-dihydro-5*H*-pyrazolo[5,1-*b*][1,3]oxazine-2-carboxylic acid according to General Procedure A and Work-up AC. The crude material was purified by silica gel flash chromatography (0 % to 15 % ethyl acetate in n-heptane) to give 3-(trifluoromethyl)-6,7-dihydro-5*H*-pyrazolo[5,1-*b*][1,3]oxazine-2-carboxylic acid (83 mg, 0.35 mmol, 70 %) as a white solid. ^{1}**H NMR** (400 MHz, DMSO-d_6) δ 13.15 (br s), 4.64 - 4.29 (m, 2H), 4.17 (t, $J = 6.1$ Hz, 2H), 2.25 (p, $J = 5.9$ Hz, 2H). ^{13}**C NMR** (101 MHz, DMSO-d_6) δ 161.7, 150.1, 138.8, 122.1 (q, $^{1}J_{C\text{-}F} = 266.1$ Hz, $\underline{C}F_3$), 91.8 (q, $^{2}J_{C\text{-}F} = 38.6$ Hz, $\underline{C}$-CF_3), 66.6, 44.5, 20.5. ^{19}**F NMR** (376 MHz, $CDCl_3$) δ -53.27 (s, CF_3). **HRMS** (ESI/Q-TOF) m/z $[M+H]^+$ *calcd* 237.0482 *found* 237.0469.

4-chloro-3-cyclopropyl-5-(trifluoromethyl)-1*H*-pyrazole (6e)

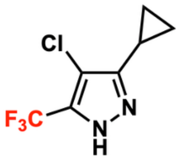

6e

Prepared from commercial 4-chloro-3-cyclopropyl–1*H*-pyrazole according to General Procedure A and Work-up AA. The crude material was purified by silica gel column chromatography (5 % ethyl acetate in n-hexane) to give 4-chloro-3-cyclopropyl-5-(trifluoromethyl)-1*H*-pyrazole (73 mg, 0.35 mmol, 69 %) as a white solid. ^{1}**H NMR** (400 MHz, $CDCl_3$) δ 12.54 (br s), 1.80 (tt, $J = 8.6$ Hz, 5.3 Hz, 1H), 1.19 – 0.89 (m, 2H), 0.89 – 0.67 (m, 2H). ^{13}**C NMR** (126 MHz, $CDCl_3$) δ 144.7, 139.1 (d_{app}, $^{2}J_{C\text{-}F} = 40.0$ Hz, $\underline{C}$-CF_3), 120.6 (q, $^{1}J_{C\text{-}F} = 270.2$ Hz, CF_3), 107.3, 6.4, 5.2. ^{19}**F NMR** (376 MHz, $CDCl_3$) δ -62.66 (s, CF_3). **HRMS** (ESI/Q-TOF) m/z $[M^+]$ *calcd* 210.0172 *found* 210.0175.

Methyl 4-chloro-5-(trifluoromethyl)-1*H*-pyrazole-3-carboxylate (6f)

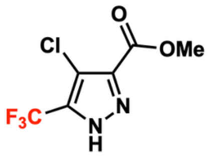

6f

Prepared from commercial methyl 4-chloro-1*H*-pyrazole-3-carboxylate according to General Procedure A and Work-up AA. The crude material was purified by silica gel column chromatography (5 % to 10 % ethyl acetate in n-hexane), giving methyl 4-chloro-5-(trifluoromethyl)-1*H*-pyrazole-3-carboxylate (68.3 mg, 30 mmol, 60 %) as a white solid. ^{1}**H NMR** (400 MHz, $CDCl_3$) δ 4.02 (s, 3H) ^{13}**C NMR** (126 MHz, $CDCl_3$) δ 158.5, 141.3 (q, $^{2}J_{C\text{-}F} = 38.1$ Hz, $\underline{C}$-CF_3), 132.0, 120.0 (q, $^{1}J_{C\text{-}F} = 270.0$ Hz, CF_3), 113.2, 53.2. ^{19}**F NMR** (376 MHz, $CDCl_3$) δ -62.77 (s, CF_3). **HRMS** (ESI/Q-TOF) m/z $[M^-]$ *calcd* 227.9913 *found* 227.9914. The ^{1}H NMR data is consistent with the literature [29].

Methyl 4-bromo-5-(trifluoromethyl)-1*H*-pyrazole-3-carboxylate (6g)

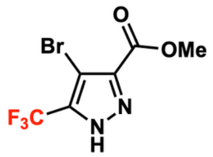

6g

Prepared from commercial methyl 4-bromo-1*H*-pyrazole-3-carboxylate according to General Procedure A and Work-up AA. The crude material was purified by silica gel column chromatography (5 % to 25 % ethyl acetate in n-hexane), giving methyl 4-bromo-5-(trifluoromethyl)-1*H*-pyrazole-3-carboxylate (75 mg, 0.28 mmol, 55 %) as a white solid. ^{1}**H NMR** (400 MHz, $CDCl_3$) δ 4.02 (s, 3H) ^{13}**C NMR** (126 MHz, $CDCl_3$) δ 158.4, 143.2 (d_{app}, $^{2}J_{C\text{-}F}$ = 38.6 Hz, C-CF_3), 133.8, 120.2 (q, $^{1}J_{C\text{-}F}$ = 270.2 Hz, CF_3), 96.7, 53.2 ^{19}**F NMR** (376 MHz, $CDCl_3$) δ -62.69 (s, CF_3). **HRMS** (ESI/Q-TOF) m/z [M^-] *calcd* 271.9408 *found* 271.9408. The ^{1}H NMR data is consistent with the literature [29].

4-bromo-5-isopropyl-3-(trifluoromethyl)-1*H*-pyrazole (6h)

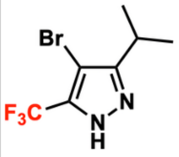

6h

Prepared from 4-bromo-5-isopropyl-1*H*-pyrazole according to General Procedure A and Work-up AA. The crude material was purified by silica gel column chromatography (5 % ethyl acetate in n-hexane) to give 4-bromo-5-isopropyl-3-(trifluoromethyl)-1*H*-pyrazole (73 mg, 0.28 mmol, 57 %) as a white solid. ^{1}**H NMR** (400 MHz, $CDCl_3$) δ 3.14 (hept, *J* = 7.1 Hz, 1H), 1.29 (d, *J* = 7.0 Hz, 6H). ^{13}**C NMR** (126 MHz, $CDCl_3$) δ 150.2, 141.0 (d_{app}, $^{2}J_{C\text{-}F}$ = 34.5 Hz, C-CF_3), 120.9 (q, $^{1}J_{C\text{-}F}$ = 269.6 Hz, CF_3) 89.9, 25.7, 20.5. ^{19}**F NMR** (376 MHz, $CDCl_3$) δ -62.50 (s, CF_3). **HRMS** (ESI/Q-TOF) m/z [M^+] *calcd* 303.9320 *found* 303.9320.

4-iodo-5-isopropyl-3-(trifluoromethyl)-1*H*-pyrazole (6i)

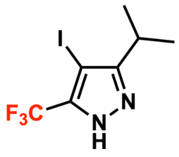

6i

Prepared from 4-iodo-5-isopropyl-1*H*-pyrazole according to General Procedure A and Work-up AA. The crude material was purified by silica gel column chromatography (5 % ethyl acetate in n-hexane) to give 4-iodo-5-isopropyl-3-(trifluoromethyl)-1*H*-pyrazole (89 mg, 0.29 mmol, 58 %) as a white solid. ^{1}**H NMR** (400 MHz, $CDCl_3$) δ 12.04 (br s), 3.08 (hept, *J* = 7.1 Hz, 1H), 1.25 (d, *J* = 7.1 Hz, 6H). ^{13}**C NMR** (126 MHz, $CDCl_3$) δ 153.4, 143.9 (q, $^{2}J_{C\text{-}F}$ = 36.1 Hz, C-CF_3), 121.0 (d, $^{1}J_{C\text{-}F}$ = 269.8 Hz, CF_3), 55.0, 26.9, 20.7. ^{19}**F NMR** (376 MHz, $CDCl_3$) δ -62.03 (s, CF_3). **HRMS** (ESI/Q-TOF) m/z [M^+] *calcd* 303.9684 *found* 303.9685.

1-(4-bromo-5-(trifluoromethyl)-1*H*-pyrazol-3-yl)ethan-1-one (6j)

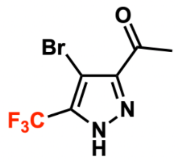

6j

Prepared from 1-(4-bromo-1*H*-pyrazol-3-yl)ethan-1-one according to General Procedure A and Work-up AA. The crude material was purified by silica gel column chromatography (5 % to 10 % ethyl acetate in n-hexane), giving 1-(5-(trifluoromethyl)-1*H*-pyrazol-4-yl)ethan-1-one (76 mg, 0.30 mmol, 59 %) as a white solid. ^{1}**H NMR** (400 MHz, $CDCl_3$) δ 2.75 (s, 3H). ^{13}**C NMR** (126 MHz, $CDCl_3$) δ 188.1, 143.1 (q, $^{2}J_{C\text{-}F}$ = 38.0 Hz, C-CF_3), 140.7, 120.2 (q, $^{1}J_{C\text{-}F}$ = 270.2 Hz, CF_3), 95.6, 29.2. ^{19}**F NMR** (376 MHz, $CDCl_3$) δ -62.59 (s, CF_3). **HRMS** (ESI/Q-TOF) m/z [M$^-$] *calcd* 255.9459 *found* 255.9457.

4-bromo-5-(trifluoromethyl)-1*H*-pyrazole-3-carbonitrile (6k)

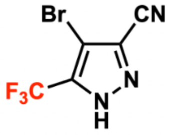

6k

Prepared from commercial 4-bromo-1H-pyrazole-3-carbonitrile according to General Procedure A and Work-up AA. The crude material was purified by silica gel column chromatography (5 % ethyl acetate in n-hexane), giving 4-bromo-5-(trifluoromethyl)-1*H*-pyrazole-3-carbonitrile (62 mg, 0.26 mmol, 52 %) as a white solid. ^{13}**C NMR** (126 MHz, DMSO-d_6) δ 137.3 (q, $^{2}J_{C\text{-}F}$ = 37.0 Hz, CF_3), 120.9, 119.8 (q, $^{1}J_{C\text{-}F}$ = 269.5 Hz, CF_3), 109.9, 99.7. ^{19}**F NMR** (376 MHz, DMSO-d_6) δ -60.95 (s, CF_3). **HRMS** (ESI/Q-TOF) m/z [M$^-$] *calcd* 238.9306 *found* 238.9305.

1-(4-iodo-5-(trifluoromethyl)-1*H*-pyrazol-3-yl)ethan-1-one (6l)

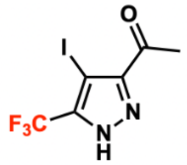

6l

Prepared from 1-(4-iodo-1*H*-pyrazol-3-yl)ethan-1-one according to General Procedure A and Work-up AA. The crude material was purified by silica gel column chromatography (5 % ethyl acetate in n-hexane), giving 1-(4-iodo-5-(trifluoromethyl)-1*H*-pyrazol-3-yl)ethan-1-one (93 mg, 0.29 mmol, 58 %) as a white solid. ^{1}**H NMR** (400 MHz, $CDCl_3$) δ 2.81 (s, 3H). ^{13}**C NMR** (126 MHz, $CDCl_3$) δ 188.4, 146.3 (q, $^{2}J_{C\text{-}F}$ = 36.3 Hz, C-CF_3), 144.2, 120.4 (q, $^{1}J_{C\text{-}F}$ = 270.5 Hz, CF_3), 59.4, 29.4 ^{19}**F NMR** (376 MHz, $CDCl_3$) δ -62.19 (s, CF_3). **HRMS** (ESI/Q-TOF) m/z [M$^-$] *calcd* 303.9320 *found* 303.9320.

Methyl 4-iodo-5-(trifluoromethyl)-1*H*-pyrazole-3-carboxylate (6m)

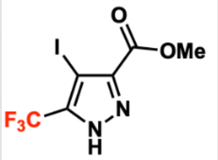

6m

Prepared from methyl 4-iodo-1*H*-pyrazole-3-carboxylate according to General Procedure A and Work-up AA. The crude material was purified by silica gel column chromatography (5 % ethyl acetate in n-hexane) to give methyl 4-iodo-5-(trifluoromethyl)-1*H*-pyrazole-3-carboxylate (69 mg, 0.21 mmol, 43 %) as a white solid. ^{1}**H NMR** (400 MHz, $CDCl_3$) δ 4.01 (s, 3H). ^{13}**C NMR** (126 MHz, $CDCl_3$) δ 158.4, 146.6 (q, $^{2}J_{C\text{-}F}$ = 36.9 Hz, C-CF_3), 136.8, 120.3 (q, $^{1}J_{C\text{-}F}$ = 270.7 Hz, CF_3), 60.4, 53.1. ^{19}**F NMR** (376 MHz, $CDCl_3$) δ -62.31 (s, CF_3). **HRMS** (ESI/Q-TOF) m/z [M^-] *calcd* 319.9270 *found* 319.9269.

Ethyl 3-cyano-5-(trifluoromethyl)-1*H*-pyrazole-4-carboxylate (6n)

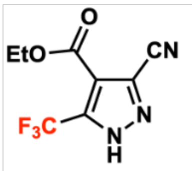

6n

Prepared from commercial ethyl 3-cyano-5-1*H*-pyrazole-4-carboxylate according to General Procedure A and Work-up procedure AA. The crude material was purified by silica gel column chromatography (5 % to 15 % ethyl acetate in n-hexane), giving ethyl 3-cyano-5-(trifluoromethyl)-1*H*-pyrazole-4-carboxylate (51.6 mg, 0.22 mmol, 44 %) as a yellow oil. ^{1}**H NMR** (400 MHz, $CDCl_3$) δ 4.45 (q, J = 7.1 Hz, 2H), 1.42 (t, J = 7.1 Hz, 3H). ^{13}**C NMR** (126 MHz, $CDCl_3$) δ 159.0, 138.8 (q, $^{2}J_{C\text{-}F}$ = 38.6 Hz, C-CF_3), 124.3, 118.9 (q, $^{1}J_{C\text{-}F}$ = 270.7 Hz, CF_3), 118.0, 109.8, 62.9, 13.9. ^{19}**F NMR** (376 MHz, $CDCl_3$) δ -61.44 (s, CF_3). **HRMS** (ESI/Q-TOF) m/z [M^-] *calcd* 233.0407 *found* 233.0408.

3-(tert-butyl)-5-(trifluoromethyl)-1*H*-pyrazole-4-carbonitrile (6o)

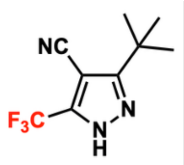

6o

Prepared from commercial 3-(tert-butyl)-1*H*-pyrazole-4-carbonitrile according to General Procedure A and Work-up AA. The crude material was purified by silica gel column chromatography (5 % ethyl acetate in n-hexane), giving 3-(tert-butyl)-5-(trifluoromethyl)-1*H*-pyrazole-4-carbonitrile (59 mg, 0.27 mmol, 54 %) as a white solid. ^{1}**H NMR** (400 MHz, $CDCl_3$) δ 1.52 (s, 9H). ^{13}**C NMR** (126 MHz, $CDCl_3$) δ 160.4, 146.0 (q, $^{2}J_{C\text{-}F}$ = 38.6 Hz, C-CF_3), 119.8 (q, $^{1}J_{C\text{-}F}$ = 270.2 Hz, CF_3), 111.7, 87.8, 32.9, 26.8. ^{19}**F NMR** (376 MHz, $CDCl_3$) δ -62.66 (s, CF_3). **HRMS** (ESI/Q-TOF) m/z [M^-] *calcd* 217.0827 *found* 217.0828.

3-methyl-5-(trifluoromethyl)-1*H*-pyrazole-4-carboxylic acid (6p)

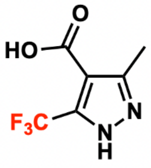

6p

Prepared from commercial 3-methyl-1*H*-pyrazole-4-carboxylic acid according to General Procedure A and Work-up AC. The crude material was purified by silica gel column chromatography (10 % to 25 %ethyl acetate in n-hexane), giving 3-methyl-5-(trifluoromethyl)-1*H*-pyrazole-4-carboxylic acid (37 mg, 0.19 mmol, 38 %) as a white solid. ^{1}**H NMR** (400 MHz, DMSO-d_6) δ 13.70 (br s), 12.75 (br s), 2.45 (s, 3H). ^{13}**C NMR** (126 MHz, DMSO-d_6) δ 162.9, 146.3, 141.0 (q, $^{2}J_{C\text{-}F}$ = 36.3 Hz, $\underline{C}$-CF_3), 121.1 (q, $^{1}J_{C\text{-}F}$ = 268.9 Hz, CF_3) 108.9, 11.0. ^{19}**F NMR** (376 MHz, DMSO-d_6) δ -60.70 (s, CF_3). **HRMS** (ESI/Q-TOF) m/z [M$^+$] *calcd* 194.0303 *found* 194.0304.

1-(3-methyl-5-(trifluoromethyl)-1*H*-pyrazol-4-yl)ethan-1-one (6q)

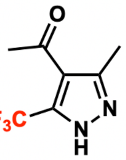

6q

Prepared from commercial 1-(3-methyl-1*H*-pyrazol-4-yl)ethan-1-one according to General Procedure A and Work-up AA. The crude material was purified by silica gel column chromatography (5 % ethyl acetate in n-hexane) to give 1-(3-methyl-5-(trifluoromethyl)-1*H*-pyrazol-4-yl)ethan-1-one (50 mg, 0.26 mmol, 52 %) as a white solid. ^{1}**H NMR** (400 MHz, $CDCl_3$) δ 2.58 (s, 1H), 2.54 (q, J = 1.0 Hz, 1H). ^{13}**C NMR** (75 MHz, MeOD) 194.2, 147.2, 142.6 (q, $^{2}J_{C\text{-}F}$ = 37.3 Hz, $\underline{C}$-CF_3), 124.4 (q, $^{1}J_{C\text{-}F}$ = 268.7 Hz, CF_3), 118.7, 30.4, 12.1. ^{19}**F NMR** (376 MHz, $CDCl_3$) δ -60.35 (s, CF_3). **HRMS** (ESI/Q-TOF) m/z [M$^+$] *calcd* 192.0510 *found* 192.0512.

5-cyclopropyl-4-nitro-3-(trifluoromethyl)-1*H*-pyrazole (6r)

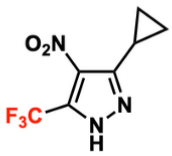

6r

Prepared from commercial 5-cyclopropyl-4-nitro-1*H*-pyrazole according to General Procedure A and Work-up AA. The crude material was purified by silica gel column chromatography (5 % to 10 % ethyl acetate in n-hexane), giving 5-cyclopropyl-4-nitro-3-(trifluoromethyl)-1*H*-pyrazole (59 mg, 0.27 mg, 53 %) as a pale yellow solid. ^{1}**H NMR** (400 MHz, DMSO-d_6) δ 14.09 (br s), 2.56 (tt, J = 8.5 Hz, 5.3 Hz, 1H), 1.22 – 1.16 (m, 2H), 1.11 – 1.06 (m, 2H). ^{13}**C NMR** (126 MHz, DMSO-d_6) δ 149.2, 136.0 (q, $^{2}J_{C\text{-}F}$ = 38.6 Hz, $\underline{C}$-CF_3), 129.7, 119.8 (q, $^{1}J_{C\text{-}F}$ = 268.9 Hz, CF_3), 8.9, 6.8. ^{19}**F NMR** (376 MHz, DMSO-d_6) δ -62.10 (s, CF_3). **HRMS** (ESI/Q-TOF) m/z [M$^-$] *calcd* 221.0412 *found* 221.0413.

3-methyl-4-nitro-5-(trifluoromethyl)-1*H*-pyrazole (6s)

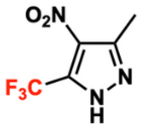

6s

Prepared from 3-methyl-4-nitro-1*H*-pyrazole according to General Procedure A and Work-up AA. The crude material was purified by silica gel column chromatography (5 % to 10 % ethyl acetate in n-hexane), giving 3-methyl-4-nitro-5-(trifluoromethyl)-1H-pyrazole (47 mg, 0.24 mmol, 48 %) as a yellow oil. ^{1}**H NMR** (400 MHz, $CDCl_3$) δ 2.75 (s, 3H). ^{13}**C NMR** (126 MHz, $CDCl_3$) δ 143.9, 138.1 (q, $^{2}J_{C\text{-}F}$ = 39.5 Hz, C-CF_3), 129.9, 119.6 (q, $^{1}J_{C\text{-}F}$ = 269.8 Hz, CF_3), 11.7. ^{19}**F NMR** (376 MHz, $CDCl_3$) δ -63.29 (s, CF_3). **HRMS** (ESI/Q-TOF) m/z [M^-] *calcd* 195.0256 *found* 195.0259.

3-bromo-4-methyl-5-(trifluoromethyl)-1*H*-pyrazole (6t)

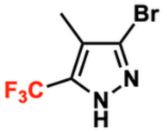

6t

Prepared from commercial 3-bromo-4-methyl-1*H*-pyrazole according to General Procedure A and Work-up AA. The crude material was purified by silica gel column chromatography (2 % to 5 % ethyl acetate in n-hexane) to give 3-bromo-4-methyl-5-(trifluoromethyl)-1*H*-pyrazole (73 mg, 0.32 mmol, 63 %) as a white solid. ^{1}**H NMR** (400 MHz, $CDCl_3$) δ 11.25 (br s), 2.13 (s, 3H). ^{13}**C NMR** (75 MHz, DMSO-d_6) δ 138.9 (br s), 121.3 (q, $^{1}J_{C\text{-}F}$ = 270.1 Hz, CF_3), 114.5, 114.1, 7.5. ^{19}**F NMR** (376 MHz, $CDCl_3$) δ - 62.19 (s, CF_3). **HRMS** (ESI/Q-TOF) m/z [M^-] *calcd* 227.9510 *found* 227.9510.

4-bromo-3-methyl-5-(trifluoromethyl)-1*H*-pyrazole (6u)

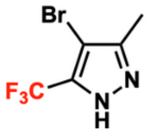

6u

Prepared from commercial 4-bromo-3-methyl-1*H*-pyrazole according to General Procedure A and Work-up AA. The crude material was purified by silica gel column chromatography (5 % ethyl acetate in n-hexane), giving 4-bromo-3-methyl-5-(trifluoromethyl)-1*H*-pyrazole (46 mg, 0.20 mmol, 40 %) as a white solid. ^{1}**H NMR** (400 MHz, $CDCl_3$) δ 11.18 (br s), 2.31 (s, 3H). ^{13}**C NMR** (126 MHz, $CDCl_3$) δ 141.5, 140.9 (q, $^{2}J_{C\text{-}F}$ = 34.9 Hz, C-CF_3), 120.8 (q, $^{1}J_{C\text{-}F}$ = 269.6 Hz, CF_3), 92.0, 9.9. ^{19}**F NMR** (376 MHz, $CDCl_3$) δ -62.44 (s, CF_3). **HRMS** (ESI/Q-TOF) m/z [M^+] *calcd* 227.9510 *found* 227.9505. The data is consistent with the literature [30].

3-(trifluoromethyl)-2,5,6,7-tetrahydro-4*H*-indazol-4-one (6v)

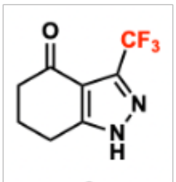

6v

Prepared from commercial 2,5,6,7-tetrahydro-4*H*-indazol-4-one according to General Procedure A and Work-up The crude material was purified by silica gel column chromatography (5 % to 30 % ethyl acetate in n-hexane), giving 3-(trifluoromethyl)-2,5,6,7-tetrahydro-4*H*-indazol-4-one (23.7 mg, 0.12 mmol, 23 %) as a white solid. ^{1}**H NMR** (400 MHz, CD_3CN) δ 11.53 (br s, 1H), 2.91 (t, $J = 6.3$ Hz, 2H), 2.52 – 2.43 (m, 2H), 2.21 – 2.09 (m, 2H). ^{13}**C NMR** (75 MHz, MeOD) δ 193.5, 154.7, 140.9 (q, $^{2}J_{C\text{-}F} = 39.3$ Hz, C-CF_3), 122.2 (q, $^{1}J_{C\text{-}F} = 268.1$ Hz, CF_3), 115.9, 39.5, 24.3, 21.8. ^{19}**F NMR** (376 MHz, CD_3CN) δ -63.60 (s, CF_3). **HRMS** (ESI/Q-TOF) m/z [M^+] *calcd* 204.0510 *found* 204.0513.

3-(trifluoromethyl)-1,4,5,6-tetrahydro-7*H*-pyrazolo[3,4-*c*]pyridin-7-one (6w)

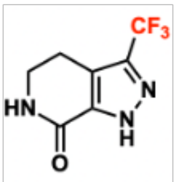

6w

Prepared from commercial 1,4,5,6-tetrahydro-7*H*-pyrazolo[3,4-*c*]pyridin-7-one according to General Procedure A and Work-up AA. The crude material was purified by silica gel column chromatography (5 % to 35 % ethyl acetate in n-hexane), giving 3-(trifluoromethyl)-1,4,5,6-tetrahydro-7*H*-pyrazolo[3,4-*c*]pyridin-7-one (28.6 mg, 0.14 mmol, 28 %) as a white solid. ^{1}**H NMR** (400 MHz, DMSO-d_6) δ 14.53 (br s), 7.96 (br s), 3.45 (td, $J = 6.8$ Hz, 2.8 Hz, 2H), 2.81 (t, $J = 6.9$ Hz, 2H). ^{13}**C NMR** (126 MHz, DMSO-d_6) δ 158.3, 137.0 (q, $^{2}J_{C\text{-}F} = 37.0$ Hz, C-CF_3), 134.9, 121.7 (d_{app}, $^{1}J_{C\text{-}F} = 268.9$ Hz, CF_3), 119.6, 40.8, 19.1. ^{19}**F NMR** (376 MHz, DMSO-d_6) δ -59.80 (s, CF_3). **HRMS** (ESI/Q-TOF) m/z [M^+] *calcd* 205.0463 *found* 205.0472.

5-isopropyl-1-methyl-3-(trifluoromethyl)-1,7-dihydro-4*H*-pyrazolo[3,4-*d*]pyrimidine-4,6(5*H*)-dione (6x)

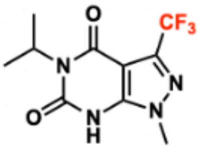

6x

Prepared from 5-isopropyl-1-methyl-1,7-dihydro-4*H*-pyrazolo[3,4-*d*]pyrimidine-4,6(5*H*)-dione according to General Procedure A and Work-up AA. The crude material was purified by silica gel flash chromatography (0 % to 40 % ethyl acetate in n-heptane), giving 5-isopropyl-1-methyl-3-(trifluoromethyl)-1,7-dihydro-4*H*-pyrazolo[3,4-*d*]pyrimidine-4,6(5*H*)-dione (50.8 mg, 0.19 mmol, 37 % as a white solid. ^{1}**H NMR** (400 MHz, DMSO-d_6) δ 12.46 (br s), 5.08 (hept, $J = 6.9$ Hz, 1H), 3.82 (s, 3H), 1.39 (d, $J = 6.9$ Hz, 6H). ^{13}**C NMR** (126 MHz, DMSO-d_6) δ 156.1, 150.3, 144.6, 136.1 (q, $^{2}J_{C\text{-}F} = 39.2$ Hz, C-CF_3), 120.2 (q, $^{1}J_{C\text{-}F} = 268.9$ Hz, CF_3), 96.1, 44.1, 35.9, 19.2. ^{19}**F NMR** (376 MHz, DMSO-d_6) δ -61.50 (s, CF_3). **HRMS** (ESI/Q-TOF) m/z $[M+H]^+$ *calcd* 277.0907 *found* 277.0893.

4,6-dichloro-3-(trifluoromethyl)-2*H*-pyrazolo[3,4-*d*]pyrimidine (7)

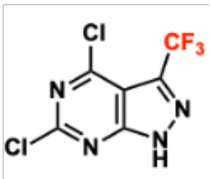

7

Prepared from commercial 4,6-dichloro-2*H*-pyrazolo[3,4-*d*]pyrimidine according to General Procedure A and Work-up AA. The crude material was purified by silica gel column chromatography (5 % ethyl acetate in n-hexane) to give 4,6-dichloro-3-(trifluoromethyl)-2*H*-pyrazolo[3,4-d]pyrimidine (43 mg, 0.17 mmol, 33 %) as a white solid. ^{13}**C NMR** (126 MHz, $CDCl_3$) δ 158.5, 156.9, 156.5, 136.0 (q, $^{2}J_{C\text{-}F}$ = 41.1 Hz, C-CF_3), 119.6 (q, $^{1}J_{C\text{-}F}$ = 270.2 Hz, CF_3), 109.1. ^{19}**F NMR** (376 MHz, $CDCl_3$) δ -61.10 (s, CF_3). **HRMS** (ESI/Q-TOF) m/z [M^-] *calcd* 255.9530 *found* 255.9531.

***N*-(5-(trifluoromethyl)-1,3,4-thiadiazol-2-yl)acetamide (8)**

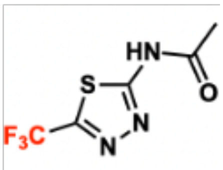

8

Prepared from *N*-(1,3,4-thiadiazol-2-yl)acetamide according to General Procedure A and Work-up AA. The crude material was purified by silica gel column chromatography (20 % ethyl acetate in n-hexane) to give *N*-(5-(trifluoromethyl)-1,3,4-thiadiazol-2-yl)acetamide (61 mg, 0.29 mmol, 58 %) as a white solid. ^{1}**H NMR** (400 MHz, DMSO-d_6) δ 2.26 (s, 3H). ^{13}**C NMR** (126 MHz, DMSO-d_6) δ 169.7, 161.5, 150.4 (d_{app}, $^{2}J_{C\text{-}F}$ = 33.6 Hz, C-CF_3), 120.1 (q, $^{1}J_{C\text{-}F}$ = 271.6 Hz, CF_3), 22.3. ^{19}**F NMR** (376 MHz, DMSO-d_6) δ -53.51 (s, CF_3).

5. Limitations

Tests were additionally conducted on marketed drugs and their derivatives, as shown by the selected examples below.

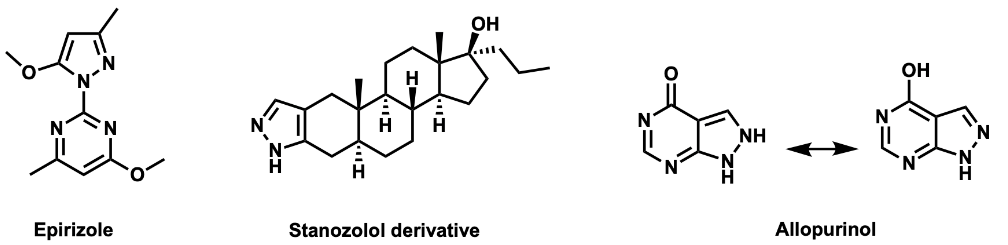

In the case of epirizole, a lack of selectivity was observed between the pyrazole and the pyrimidine, leading to a mixture of trifluoromethylation products. For allopurinol and the stanozolole derivative, only traces of products were observed analytically and could not be isolated. This was attributed to the presence of hydroxyl groups, which had already been observed to be incompatible with the reaction on simpler substrates.

6. X-ray Crystal Structure of 6j

The structure of product **6j** was confirmed by X-ray diffraction. Single clear colorless irregular-shaped crystals of **6j** were obtained from dichloromethane. A suitable crystal 0.17×0.07×0.04 mm^3 was selected and placed on a miTeGen micromount on an XtaLAB Synergy R, HyPix-Arc 100 diffractometer. The crystal was kept steady at T = 200.00(10) K during data collection. Data was measured using ω scans of 0.5°per frame for 0.1 s using Cu K_α radiation. The diffraction pattern was indexed and the total number of runs and images was based on the strategy calculation from the program CrysAlisPro (Rigaku). The maximum resolution that was achieved was Θ = 68.537° (0.83Å). The unit cell was refined using CrysAlisPro (Rigaku, V1.171.43.144a, 2024) on 18498 reflections, 66 % of the observed reflections. Data reduction, scaling and absorption corrections were performed using CrysAlisPro (Rigaku, V1.171.43.144a, 2024). The final completeness is 99.80 % out to 68.537° in Θ. A gaussian absorption correction was performed using CrysAlisPro V1.171.43.144a (Rigaku Oxford Diffraction, 2024). Numerical absorption correction based on gaussian interaction over a multifaceted crystal model. Empirical absorption correction using spherical harmonics, implemented in SCALE3 ABSPACK scaling algorithm. The absorption coefficient μ of this material is 6.972 mm^{-1} at this wavelength (λ = 1.542 Å) and the minimum and maximum transmissions are 0.860 and 0.988. The structure was solved and the space group *P*-1 (# 2) determined by the SheIXT 2018/2 (Sheldrick, 2018) structure solution program using Intrinsic Phasing and refined by Least Squares using version 2018/3 of SheIXL 2018/3 (Sheldrick, 2015). All non-hydrogen atoms were refined anisotropically. Most hydrogen atom positions were calculated geometrically an refined using the riding model, but some hydrogen atoms were refined freely.

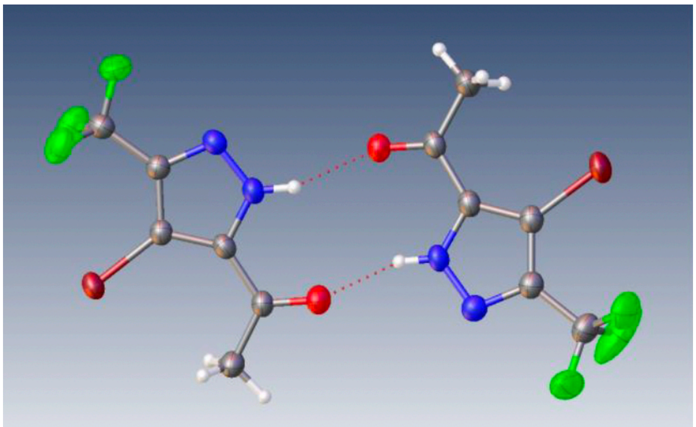

Figure S1: Thermal Ellipsoids for **6j**

Table S7: Crystallographic data for **6j**

**Compound**	**6j**
Formula	$C_6H_4BrF_3N_2O$
D_{calc} / cm^{-3}	2.054
μ / mm^3	6.972
Formula weight	257.2
Color	clear colorless
Shape	irregular
Size / mm^3	0.17 x 0.07 x 0.04
T / K	200.00(10)
Crystal System	triclinic
Space Group	P-1
a / Å	7.9554(2)
b / Å	9.2791(2)
c / Å	11.5040(2)
α / °	92.1520(10)
β / °	101.077(2)
γ / °	93.183(2)
V / Å^3	831.10(3)
Z	4
Z'	2
Wavelength / Å	1.54184
Radiation type	Cu K_α
Θ_{min} / °	3.920
Θ_{max} / °	68.537
Measured Refl.	27978
Independent Refl.	3040
Reflections with I > 2(I)	2719
R_{int}	0.0529
Parameters	241
Restraints	0
Largest Peak	1.258
Deepest Hole	-0.868
GooF	1.041
wR_2 (all data)	0.1247
wR_2	0.1218
R_1(all data)	0.0460
R_1	0.0426

Table S8: Fractional Atomic Coordinates (x 10^4) and Equivalent Isotropic Displacement Parameters ($Å^2$ x 10^3) for **6j**. U_{eq} is defined as 1 / 3 of the trace of the orthogonalized U_{ij}

Atom	x	y	z	U_{eq}
Br1	6398.7(4)	1004.5(3)	3498.0(3)	40.62(17)
F1	2455(3)	-488(2)	3304.7(19)	56.1(6)
F2	2042(3)	1269(3)	2188.8(18)	64.1(6)
F3	440(3)	907(3)	3445(2)	78.2(8)
O1	7125(3)	4504(3)	6819(2)	48.9(6)
N1	4029(3)	3340(3)	5626(2)	37.1(6)
N2	2635(3)	2655(3)	4971(2)	38.9(6)
C1	8775(4)	3458(4)	5547(3)	40.5(7)
C2	7153(4)	3707(3)	5959(3)	33.9(6)
C3	5519 (4)	3003(3)	5305(3)	33.4(6)
C4	5035(4)	2003(3)	4350(3)	32.6(6)
C5	3238(4)	1832(3)	4190(3)	34.2(6)
C6	2026(4)	877(4)	3287(3)	40.3(7)
Br2	3894.8(5)	8294.2(5)	10942.2(4)	57.59(19)
F4	7826(3)	7998(3)	12329(3)	71.1(17)
F5	9789(3)	7454(4)	11440(2)	94.7(10)
F6	8520(5)	9394(3)	11096(3)	118.7(15)
O2	3043(3)	5237(3)	7354(2)	44.3(6)
N3	6146(4)	5947(3)	8761(2)	36.2(6)
N4	7569(3)	6419(3)	9516(2)	40.2(6)
C7	1397(4)	6467(4)	8547(3)	46.2(8)
C8	3031(4)	5993(3)	8236(3)	35.0(6)
C9	4692(4)	6430(3)	9023(3)	33.9(6)
C10	5219(4)	7299(3)	10056(3)	37.1(7)
C11	7015(4)	7242(3)	10317(3)	36.9(7)
C12	8287(5)	8043(4)	11286(3)	47.7(8)

Table S9: Anisotropic Displacement Parameters (x 10^4) **6j**. The anisotropic displacement factor exponent takes the form: $-2\pi^2[h^2a^{*2} \cdot U_{11} + ... + 2hka^* \cdot b^* \cdot U_{12}]$

**Atom**	***U11***	***U22***	***U33***	***U23***	***U13***	***U12***
Br1	37.7(2)	44.5(2)	40.9(2)	-14.09(16)	12.94(16)	5.93(15)
F1	64.0(13)	38.1(10)	58.6(12)	-8.7(9)	-3.6(10)	-2.1(9)
F2	80.1(16)	65.1(13)	38.0(11)	0.5(10)	-7.4(10)	-7.9(12)
F3	33.0(11)	106(2)	88.8(18)	-46.9(15)	9.1(11)	-6.1(12)
O1	41.9(13)	59.6(14)	43.5(13)	-19.7(11)	9.0(10)	0.3(11)
N1	34.6(14))	40.8(14)	35.4(14)	-11.9(11)	7.8(11)	5.2(11)
N2	31.6(13)	44.7(14)	40.6(14)	-9.8(11)	8.7(11)	6.0(11)
C1	35.0(17)	46.9(17)	38.7(16)	-11.2(13)	8.9(13)	-2.5(13)
C2	34.5(15)	34.8(14)	31.8(14)	-7.1(11)	6.4(12)	2.8(12)
C3	32.3(15)	36.4(15)	32.8(15)	-4.6(12)	9.1(12)	7.6(12)
C4	33.3(15)	33.3(14)	30.4(14)	-4.0(11)	5.2(12)	3.8(12)
C5	31.3(15)	36.4(15)	33.8(15)	-4.0(12)	4.2(12)	3.8(12)
C6	31.9(17)	45.1(17)	43.5(17)	-6.4(14)	7.6(14)	5.1(13)
Br2	49.3(3)	70.1(3)	52.3(3)	-31.8(2)	12.1(2)	10.3(2)
F4	70.0(15)	96.7(18)	40.3(11)	-23.7(11)	2.9(0)	-3.5(13)
F5	43.5(13)	153(3)	76.3(18)	-53.4(18)	-7.0(12)	12.0(15)
F6	166(3)	69.6(17)	84(2)	12.0(15)	-49(2)	-57.9(19)
O2	40.1(13)	48.9(13)	41.7(12)	-15.8(10)	5.6(10)	4.6(10)
N3	36.7(14)	41.4(14)	30.6(13)	-11.1(11)	8.9(11)	5.3(11)
N4	34.6(14)	47.7(15)	36.9(14)	-7.0(11)	4.9(11)	2.2(11)
C7	37.9(17)	53.6(19)	45.4(18)	-15.7(15)	5.9(14)	8.9(14)
C8	37.8(16)	33.3(14)	34.5(15)	-5.2(12)	8.6(12)	7.0(12)
C9	37.4(16)	33.2(14)	31.6(14)	-4.4(11)	8.2(12)	5.4(12)
C10	39.9(17)	37.1(16)	33.9(16)	-5.1(12)	7.0(13)	3.8(13)
C11	37.3(16)	39.7(15)	33.0(15)	-4.4(12)	6.3(13)	1.3(13)
C12	45(2)	57(2)	37.2(18)	-9.0(15)	1.5(15)	-1.7(16)

Table S10: Bond Lengths in Åfor **6j**

**Atom**	**Atom**	**Length / Å**	**Atom**	**Atom**	**Length / Å**
Br1	C4	1.857(3)	C4	C5	1.405(4)
F1	C6	1.329(4)	C5	C6	1.499(4)
F2	C6	1.330(4)	Br2	C10	1.858(3)
F3	C6	1.310(4)	F4	C12	1.322(5)
O1	C2	1.217(4)	F5	C12	1.325(5)
N1	N2	1.329(4)	F6	C12	1.290(5)
N1	C3	1.357(4)	O2	C8	1.214(4)
N2	C5	1.332(4)	N3	N4	1.329(4)
C1	C2	1.484(4)	N3	C9	1.346(4)
C2	C3	1.475(4)	N4	C11	1.329(4)
C3	C4	1.391(4)	C7	C8	1.497(5)
C8	C9	1.478(4)	C10	C11	1.407(5)
C9	C10	1.392(4)	C11	C12	1.496(4)

Table S11: Bond Angles in ° for **6j**

Atom	Atom	Atom	Angle / °	Atom	Atom	Atom	Angle / °
N2	N1	C3	114.2(3)	N4	N3	C9	114.6(3)
N1	N2	C5	104.2(3)	C11	N4	N3	104.1(3)
O1	C2	C1	121.6(3)	O2	C8	C7	122.0 (3)
O1	C2	C3	118.5(3)	O2	C8	C9	118.1(3)
C3	C2	C1	119.9(3)	C9	C8	C7	119.9(3)
N1	C3	C2	119.2(3)	N3	C9	C8	119.4(3)
N1	C3	C4	105.1(3)	N3	C9	C10	105.1(3)
C4	C3	C2	135.7(3)	C10	C9	C8	135.5(3)
C3	C4	Br1	129.4(2)	C9	C10	Br2	129.0(3)
C3	C4	C5	104.6(3)	C9	C10	C11	104.6(3)
C5	C4	Br1	125.9(2)	C11	C10	Br2	126.4(2)
N2	C5	C4	111.8(3)	N4	C11	C10	111.6 (3)
N2	C5	C6	1201(3)	N4	C11	C12	119.6(3)
C4	C5	C6	128.1(3)	C10	C11	C12	128.6(3)
F1	C6	F2	105.3(3)	F4	C12	F5	104.8 (3)
F1	C6	C5	111.9 (3)	F4	C12	C11	112.8 (3)
F2	C6	C5	111.7 (3)	F5	C12	C11	110.9(3)
F3	C6	F1	108.4(3)	F6	C12	F4	106.1(4)
F3	C6	F2	107.3(3)	F6	C12	F5	108.8(4)
F3	C6	C5	111.8(3)	F6	C12	C11	113.0(3)

Table S12: Torsion Angles in ° for **6j**

Atom	Atom	Atom	Atom	Angle / °
Br1	C4	C5	N2	177.2(2)
Br1	C4	C5	C6	-2.8(5)
O1	C2	C3	N1	4.6(4)
O1	C2	C3	C4	-176.2(3)
N1	N2	C5	C4	-0.3(3)
N1	N2	C5	C6	179.9(3)
N1	C3	C4	Br1	-177.0(2)
N1	C3	C4	C5	-0.1(3)
N2	N1	C3	C2	179.4(3)
N2	N1	C3	C4	0.0(4)
N2	C5	C6	F1	126.4(3)
N2	C5	C6	F2	115.8(3)
N2	C5	C6	F3	-4.5(4)
C1	C2	C3	N1	-174.1(3)
C1	C2	C3	C4	5.0(5)
C2	C3	C4	Br1	3.8(5)
C2	C3	C4	C5	-179.4(3)
C3	N1	N2	C5	0.2(4)
C3	C4	C5	N2	0.2(3)
C3	C4	C5	C6	-179.7(3)
C4	C5	C6	F1	53.6(4)
C4	C5	C6	F2	-64.3(4)
C4	C5	C6	F3	175.5(3)
Br2	C10	C11	N4	179.1(2)
Br2	C10	C11	C12	-5.7(5)
O2	C8	C9	N3	-1.9(4)
O2	C8	C9	C10	-0.6(3)
N3	N4	C11	C10	-0.6(3)
N3	N4	C11	C10	-0.6(3)
N3	N4	C11	C12	-176.3(3)
N3	C9	C10	Br2	-178.7(2)
N3	C9	C10	C11	-0.1(3)
N4	N3	C9	C8	179.1(3)
N4	N3	C9	C10	-0.3(4)
N4	C11	C12	F4	-138.0(3)
N4	C11	C12	F5	-20.8(5)
N4	C11	C12	F6	101.7(4)
C7	C8	C9	N3	177.2(3)
C7	C8	C9	C10	-3.6(5)
C8	C9	C10	Br2	2.0(6)
C8	C9	C10	C11	-179.4(3)
C9	N3	N4	C11	0.5(4)
C9	C10	C11	N4	0.4(4)
C9	C10	C11	C12	175.7(3)
C10	C11	C12	F4	47.0(5)
C10	C11	C12	F5	164.3(3)
C10	C11	C12	F6	-73.3(5)

Table S13: Hydrogen Fractional Atomic Coordinates (x 10^4) and Equivalent Isotropic Displacement Parameters ($Å^2$ x 10^3) for **6j**. U_{eq} is defined as 1 / 3 of the trace of the orthogonalized U_{ij}.

**Atom**	**x**	**y**	**z**	***eq***
H1	3988.78	3958.96	6217.73	44
H1A	3719.35	4054.86	6040.77	61
H1B	8661.3	3718.81	4719.07	61
H1C	9013.82	2436.19	5608.81	61
H7A	435.94	6180.43	7895.33	69
H7B	1477.2	7520.19	8680.33	69
H7C	1209.9	6010.87	9270.3	69
H3	6190(40)	5410(40)	8150(30)	31(8)

Table S14: Hydrogen Bond information for **6j**

**D**	**H**	**A**	**d(D-H) / Å**	**d(H-A) / Å**	**d(D-A) / Å**	**D-H-A / deg**
N1	H1	O2	0.88	2.01	2.855(4)	160.5
C1	H1C	$F1^1$	0.98	2.59	3.282(4)	127.9
N3	H3	O1	0.85(4)	2.01(4)	2.820(4)	159(3)

^{1}1-x, -y, 1-z

7. Computational Studies

All structures were optimized using density functional theory (DFT) as implemented in Gaussian 16 [31], with M06-2X ([32]) as functional and 6-311+G(d,p) as basis set, introducing solvation factors with the IEF-PCM method ([33], [34], [35]) (dimethyl sulfoxide as solvent). The stationary points were characterized by frequency calculations in order to verify that they have the right number of imaginary frequencies.

The activation energies have been computed as the difference between the Free Energy of the transition state and the sum of the trifluoromethyl radical and the corresponding pyrazole substrate (see Figure S2), in the following way:

$$\Delta G^{\neq} = G^{TS} - (G^{CF_3} + G^{pyrazole}) \tag{1}$$

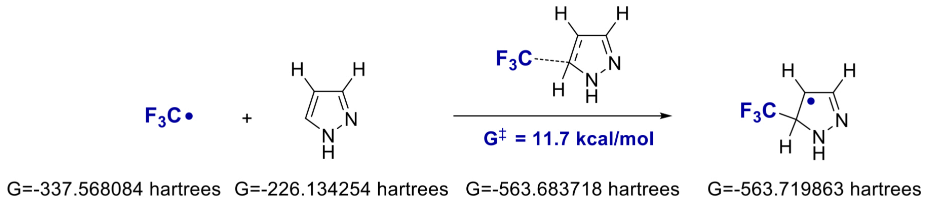

Figure S2: Absolute G energies (hartrees) of the trifluoromethyl radical addition to pyrazole. Activation energy in kcal/mol of the transition state.

The relative oxidation potential of the radicals has been measured with respect to 4-OMe-pyrazole substrate, which is the most reductive substrate of the study. The energies are given in eV, and have been computed by the following difference at equilibrium (see Figure S3):

$$\Delta G^{ox} = (G^{catOMe} + G^{RadX}) - (G^{RadOMe} + G^{catX}) \tag{2}$$

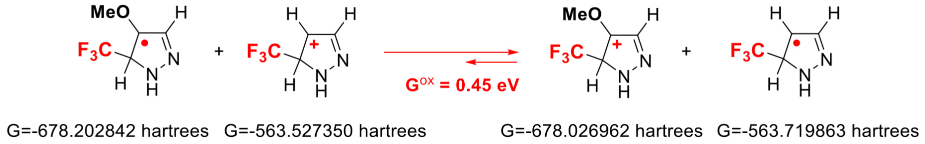

Figure S3: Relative oxidation potential of the substituted pyrazole radicals in eV, computed as the difference with the oxidation of the reference 4-methoxypyrazole radical.

Table S15: Energies of the structures studied in the main manuscript and figure above. Energies are given in Hartrees. ΔG in kcal/mol.

Compound X-Pyrazole	Starting Pyrazole	Transition State	$\Delta\Delta G^{\neq}$	Imaginary Frequency	Adduct Radical	Adduct Cation	Oxidation (eV)
**CF_3 radical**	-337.568084						
**4-OMe-**	-340.609040	-678.162908	**8.9**	-274.7	-678.202842	-678.026962	0.0
**4-Me-**	-265.411786	-602.965028	**9.3**	-334.3	-603.003974	-602.818892	0.25
**4-Cl-**	-685-737355	-1023.289031	**10.3**	-368.8	-1023.326813	-1023.129164	0.59
**3-OMe-**	-340.616983	-678.167708	**10.9**	-377.7	-678.206500	-678.010354	0.55
**4-Acetyl-**	-378.736220	716.286049	**11.5**	-376.0	-716.324900	-716.115087	0.92
**Pyrazole**	-226.134254	-563.683718	**11.7**	-357.0	-563.719863	-563.527350	0.45
**3-Acetyl-**	-378.735665	-716.285034	**11.7**	-383.8	-716.321369	-716.121475	0.65
**4-NO_2-**	-430.623251	-768.172184	**12.0**	-381.3	-768.215582	-767.990305	1.41
**6q**	-2838.997530	-3176.549474	**10.1**	-360.4	-3176.587602	-3176.392766	0.52
**6r**	-2838.997897	-3176.549715	**10.2**	-365.3	-3176.588471	-3176.394780	0.49

Table S16: 4-Methoxypyrazole. Standard Orientation.

Center Number	Atomic Number	Atomic Type	X-Coordinate (Å)	Y-Coordinate (Å)	Z-Coordinate (Å)
1	6	0	0.298314	1.029127	-0.000138
2	1	0	-0.237651	1.966006	-0.000068
3	6	0	-0.208985	-0.288581	-0.000177
4	6	0	-0.903701	-1.107309	0.000024
5	1	0	1.007753	-2.180064	-0.000052
6	7	0	1.631351	1.014672	0.000002
7	7	0	1.967527	-0.272989	0.000168
8	6	0	-2.462697	0.311211	0.000147
9	1	0	-2.367078	0.935340	0.893960
10	1	0	-2.367159	0.935713	-0.893408
11	1	0	-3.435451	-0.176500	0.000051
12	8	0	-1.489766	-0.725117	-0.000100
13	1	0	2.943571	-0.538026	-0.000003

Table S17: 4-Methoxypyrazole (Transition State). Standard oridnetation.

Center Number	Atomic Number	Atomic Type	X-Coordinate (Å)	Y-Coordinate (Å)	Z-Coordinate (Å)
1	6	0	1.477588	1.052760	-0.630354
2	1	0	2.229892	0.958038	0.402342
3	6	0	1.148248	0.158690	0.402342
4	6	0	0.059368	0.745587	1.070938
5	1	0	-0.384982	0.507788	2.025571
6	7	0	0.709170	2.143550	-0.565454
7	7	0	-0.090694	1.961579	0.484749
8	6	0	-1.503667	-0.481877	-0.052330
9	9	0	-1.052495	-1.652507	-0.504025
10	9	0	-1.841975	0.270669	-1.100671
11	9	0	-2.600112	-0.704987	0.677375
12	6	0	2.563027	-1.573285	-0.244611
13	1	0	2.076608	-1.666779	-1.219569
14	1	0	3.447617	-0.936591	-0.330694
15	1	0	2.854456	-2.556052	0.118849
16	8	0	1.652334	-1.041327	0.715812
17	1	0	-0.787732	2.658486	0.712545

Table S18: 4-Methoxy-5-trifluoromethylpyrazole (Adduct Radical). Standard orientation.

Center Number	Atomic Number	Atomic Type	X-Coordinate (Å)	Y-Coordinate (Å)	Z-Coordinate (Å)
1	6	0	-1.596487	1.269312	0.242057
2	1	0	-2.597441	1.428400	0.613671
3	6	0	-1.043217	0.102650	-0.219443
4	6	0	0.360819	0.404151	-0.653745
5	1	0	0.511400	0.262689	-1.732893
6	7	0	-0.734743	2.309147	0.171763
7	7	0	0.473698	1.788313	-0.223119
8	6	0	1.405061	-0.455686	0.048939
9	9	0	1.244799	-0.466310	-0.374421
10	9	0	2.642713	-0.003729	-0.199603
11	9	0	1.350917	-1.724718	-0.374421
12	6	0	-2.869030	-1.306023	0.107809
13	1	0	-2.941198	-1.055287	1.168370
14	1	0	-3.544067	-0.674098	-0.473453
15	1	0	-3.110102	-2.353279	-0.047455
16	8	0	-1.525418	-1.130827	-0.344499
17	1	0	1.023330	2.422364	-0.786901

Table S19: 4-Methoxy-5-(trifluoromethyl)-pyrazole (Adduct Cation). Standard orientation.

Center Number	Atomic Number	Atomic Type	X-Coordinate (Å)	Y-Coordinate (Å)	Z-Coordinate (Å)
1	6	0	-1.527692	1.317649	0.271383
2	1	0	-2.490511	1.566483	0.684331
3	6	0	-1.049554	0.116225	-0.2312269
4	6	0	0.362713	0.360341	-0.675943
5	1	0	0.475263	0.214289	-1.755782
6	7	0	-0.560192	2.252567	0.181674
7	7	0	0.497534	1.755981	-0.324278
8	6	0	1.395092	-0.503266	0.059074
9	9	0	1.277972	-0.362614	1.377451
10	9	0	2.623531	-0.137919	-0.292734
11	9	0	1.219469	-1.780199	-0.249836
12	6	0	-2.944129	-1.212533	0.110706
13	1	0	-2.989092	-0.980571	1.173418
14	1	0	-3.591821	-0.559717	-0.472467
15	1	0	-3.176047	-2.255300	-0.070731
16	8	0	-1.575680	-1.041560	-0.348030
17	1	0	1.308925	2.343557	-0.493933

Table S20: 4-Methylpyrazole

Center Number	Atomic Number	Atomic Type	X-Coordinate (Å)	Y-Coordinate (Å)	Z-Coordinate (Å)
1	6	0	0.181538	1.112651	0.000009
2	1	0	-0.090051	2.158618	0.000035
3	6	0	-0.677022	-0.011797	0.000012
4	6	0	0.190792	-1.084013	0.000017
5	1	0	0.011835	-2.147600	0.000011
6	7	0	1.461789	0.762056	-0.000012
7	7	0	1.444423	-0.575337	-0.000002
8	6	0	-2.174977	-0.034635	-0.000011
9	1	0	-2.578421	0.468257	-0.881278
10	1	0	-2.543880	-1.060825	0.000069
11	1	0	-2.578455	0.468412	0.881148
12	1	0	2.313512	-1.087139	-0.000044

Table S21: 4-Methylpyrazole (Transition State). Standard orientation.

Center Number	Atomic Number	Atomic Type	X-Coordinate (Å)	Y-Coordinate (Å)	Z-Coordinate (Å)
1	6	0	1.993345	-0.207507	0.675540
2	1	0	2.665942	0.048706	1.481076
3	6	0	1.314920	0.663251	-0.190304
4	6	0	0.554390	-0.195713	-1.002525
5	1	0	0.067399	-0.003181	-1.947574
6	7	0	1.741023	-1.488631	0.0404004
7	7	0	0.907695	-1.461497	-0.638746
8	6	0	-1.402045	0.006331	0.054530
9	9	0	-1.818529	1.268308	0.121628
10	9	0	-1.226259	-0.459374	1.286277
11	9	0	-2.334431	-0.725583	-0.553269
12	6	0	1.312955	2.157181	-0.197916
13	1	0	2.261001	2.542850	0.178395
14	1	0	1.157669	2.544517	-1.205690
15	1	0	0.515690	2.552949	0.437421
16	1	0	0.562855	-2.326360	-1.028111

Table S22: 4-Methyl-5-(trifluoromethyl)-pyrazole (Adduct Radical). Standard orientation.

Center Number	Atomic Number	Atomic Type	X-Coordinate (Å)	Y-Coordinate (Å)	Z-Coordinate (Å)
1	6	0	2.199024	-0.122688	0.290287
2	6	0	1.184048	0.687359	-0.132449
3	6	0	0.112590	-0.239245	-0.639623
4	1	0	-0.069456	-0.132738	-1.719360
5	7	0	1.948495	-1.448140	0.145326
6	7	0	0.666026	-1.536151	-0.286866
7	6	0	-1.234920	-0.039402	0.040433
8	9	0	-1.140484	-0.046947	1.374334
9	9	0	-2.102969	-1.002923	-0.301457
10	9	0	-1.779781	1.133182	-0.316665
11	1	0	0.447023	-2.355763	-0.836029
12	1	0	3.150265	0.191007	0.697157
13	6	0	1.098901	2.172748	-0.144188
14	1	0	2.050127	2.607622	0.162119
15	1	0	0.849165	2.542897	-1.142542
16	1	0	0.322488	2.534565	0.536763

Table S23: 4-Methyl-5-(trifluoromethyl)-pyrazole (Adduct Cation). Standard orientation.

Center Number	Atomic Number	Atomic Type	X-Coordinate (Å)	Y-Coordinate (Å)	Z-Coordinate (Å)
1	6	0	2.183322	-0.188469	0.296371
2	6	0	1.215551	0.659937	-0.137466
3	6	0	0.109393	-0.210987	-0.636752
4	1	0	-0.026620	-0.110383	-1.721880
5	7	0	1.793010	-1.506007	0.146767
6	7	0	0.641526	-1.531244	-0.349540
7	6	0	-1.245651	0.001746	0.048039
8	9	0	-1.129321	-0.084811	1.370244
9	9	0	-2.116649	-0.916406	-0.356124
10	9	0	-1.713488	1.204909	-0.259823
11	1	0	0.192150	-2.422534	-0.561554
12	1	0	3.152164	0.028566	0.715182
13	6	0	1.195780	2.136828	-0.137468
14	1	0	2.172253	2.530688	0.133673
15	1	0	0.897420	2.513743	-1.117366
16	1	0	0.455616	2.493123	0.586348

Table S24: 4-Chloropyrazole. Standard orientation.

Center Number	Atomic Number	Atomic Type	X-Coordinate (Å)	Y-Coordinate (Å)	Z-Coordinate (Å)
1	6	0	-0.564752	1.126944	0.000584
2	1	0	-0.287219	2.169486	0.000660
3	6	0	0.274980	-0.002521	-0.000157
4	6	0	-0.563279	-1.094301	0.000510
5	1	0	-0.366577	-2.153247	0.000184
6	7	0	-1.836962	0.755486	-0.000458
7	7	0	-1.810698	-0.581434	-0.000177
8	17	0	1.999059	-0.018707	-0.000117
9	1	0	-2.678271	-1.097328	-0.000025

Table S25: 4-Chloropyrazole (Transition State). Standard orientation.

Center Number	Atomic Number	Atomic Type	X-Coordinate (Å)	Y-Coordinate (Å)	Z-Coordinate (Å)
1	6	0	1.570880	1.117341	-0.698655
2	1	0	2.278496	1.149044	-1.512182
3	6	0	1.262474	0.044275	0.142316
4	6	0	0.242280	0.498816	0.990435
5	1	0	-0.105275	0.097555	1.930701
6	7	0	0.857374	2.191134	-0.368083
7	7	0	0.108492	1.817015	0.672471
8	6	0	-1.513915	-0.354437	-0.060770
9	9	0	-1.462594	-1.675873	-0.161941
10	9	0	-1.514646	0.182805	-1.273145
11	9	0	-2.625553	-0.001826	0.575728
12	17	0	1.920580	-1.539027	0.108977
13	1	0	-0.529324	2.477893	1.092422

Table S26: 4-Chloro-5-trifluoromethylpyrazole (Adduct Radical). Standard orientation.

Center Number	Atomic Number	Atomic Type	X-Coordinate (Å)	Y-Coordinate (Å)	Z-Coordinate (Å)
1	6	0	-1.740171	1.218598	0.334576
2	6	0	-1.166537	0.056876	-0.096198
3	6	0	0.186247	0.392298	-0.650800
4	1	0	0.282415	0.179921	-1.724561
5	7	0	-0.941184	2.289443	0.135987
6	7	0	0.228316	1.810866	-0.343170
7	6	0	1.339244	-0.327330	0.047485
8	9	0	1.267055	-0.222942	1.376772
9	9	0	2.515848	0.186446	-0.333667
10	9	0	1.349697	-1.629478	-0.258564
11	1	0	0.780694	2.451596	-0.895851
12	1	0	-2.718758	1.344914	0.772419
13	17	0	-1.838845	-1.513381	-0.092528

Table S27: 4-Chloro-5-trifluoromethylpyrazole (Adduct Cation). Standard orientation.

Center Number	Atomic Number	Atomic Type	X-Coordinate (Å)	Y-Coordinate (Å)	Z-Coordinate (Å)
1	6	0	-1.692680	1.260685	0.337071
2	6	0	-1.180522	0.083308	-0.103547
3	6	0	0.183809	0.366190	-0.644598
4	1	0	0.242445	0.182491	-1.725457
5	7	0	-0.776267	2.270737	0.135375
6	7	0	0.259162	1.792512	-0.389866
7	6	0	1.335053	-0.380680	0.054469
8	9	0	1.280112	-0.204303	1.369916
9	9	0	2.498979	0.079495	-0.386099
10	9	0	1.251414	-1.675621	-0.208335
11	1	0	1.039479	2.404483	-0.633189
12	1	0	-2.649045	1.478354	0.782434
13	17	0	-1.891862	-1.428307	-0.087180

Table S28: 3-Methoxypyrazole. Standard orientation.

Center Number	Atomic Number	Atomic Type	X-Coordinate (Å)	Y-Coordinate (Å)	Z-Coordinate (Å)
1	6	0	-0.184190	0.295159	-0.000209
2	6	0	0.924353	1.172961	0.000166
3	6	0	2.009058	0.324944	-0.000149
4	1	0	3.069018	0.521590	0.000645
5	7	0	0.175672	-0.975877	0.000088
6	7	0	1.525808	-0.932276	0.000138
7	8	0	-1.468760	0.693088	-0.000373
8	6	0	-2.437450	-0.352039	0.000226
9	1	0	-3.407213	0.138854	-0.000276
10	1	0	-2.329183	-0.975628	-0.889206
11	1	0	-2.329184	-0.974386	0.890560
12	1	0	0.916649	2.249245	0.000625
13	1	0	2.049003	-1.793464	-0.001145

Table S29: 3-Methoxypyrazole (Transition State). Standard orientation.

Center Number	Atomic Number	Atomic Type	X-Coordinate (Å)	Y-Coordinate (Å)	Z-Coordinate (Å)
1	6	0	1.482033	0.038757	0.396835
2	6	0	0.457806	-0.003454	1.354425
3	6	0	-0.443487	0.979896	0.926349
4	1	0	-1.245640	1.462924	1.465234
5	7	0	1.295037	0.992011	-0.511168
6	7	0	0.148363	1.588498	-0.139273
7	6	0	-1.904903	-0.282059	-0.135937
8	9	0	-2.419068	-1.212456	0.658974
9	9	0	-1.251595	-0.864924	-1.132222
10	9	0	-2.888832	0.460793	-0.635373
11	8	0	2.545302	-0.777948	0.381899
12	6	0	3.489851	-0.550872	-0.663326
13	1	0	3.901539	0.457461	-0.594191
14	1	0	4.274542	-1.288727	-0.519786
15	1	0	3.019296	-0.683912	-1.638882
16	1	0	0.357149	-0.670792	2.193062
17	1	0	-0.225443	2.338733	-0.700028

Table S30: 3-Methoxypyrazole (Adduct Radical). Standard orientation.

Center Number	Atomic Number	Atomic Type	X-Coordinate (Å)	Y-Coordinate (Å)	Z-Coordinate (Å)
1	6	0	-1.525576	-0.243159	-0.217227
2	6	0	-0.395140	-0.894301	-0.624722
3	6	0	0.656926	0.154547	-0.748874
4	1	0	1.027810	0.301485	-1.773094
5	7	0	-1.364028	1.097732	-0.059374
6	7	0	-0.049354	1.329861	-0.255955
7	6	0	1.885502	-0.110969	0.113751
8	9	0	1.580838	-0.292548	1.401599
9	9	0	2.752128	0.911368	0.051405
10	9	0	2.529203	-1.208541	-0.307331
11	1	0	0.184309	2.253267	-0.594904
12	8	0	-2.708328	-0.825451	0.005844
13	6	0	-3.783114	0.030820	0.401267
14	1	0	-4.641536	-0.621674	0.534884
15	1	0	-3.544645	0.537297	1.337171
16	1	0	-3.988629	0.770553	-0.373308
17	1	0	-0.268126	-1.944595	-0.826419

Table S31: 3-Methoxypyrazole (Adduct Cation). Standard orientation.

Center Number	Atomic Number	Atomic Type	X-Coordinate (Å)	Y-Coordinate (Å)	Z-Coordinate (Å)
1	6	0	-1.522710	-0.305561	-0.230160
2	6	0	-0.393343	-0.949089	-0.616046
3	6	0	0.651551	0.081932	-0.761616
4	1	0	0.994035	0.202711	-1.799331
5	7	0	-1.276853	1.070473	-0.103716
6	7	0	-0.077647	1.279377	-0.379749
7	6	0	1.886293	-0.104081	0.136430
8	9	0	1.536109	-0.217543	1.412977
9	9	0	2.699809	0.940685	0.017989
10	9	0	2.533847	-1.200662	-0.228873
11	1	0	0.293109	2.232004	-0.364109
12	8	0	-2.711587	-0.803213	0.007625
13	6	0	-3.749015	0.100679	0.442363
14	1	0	-4.622594	-0.525656	0.587479
15	1	0	-3.460443	0.576479	1.378993
16	1	0	-3.938054	0.848928	-0.326393
17	1	0	-0.256406	-2.003305	-0.798043

Table S32: 4-Acetylpyrazole. Standard orientation.

Center Number	Atomic Number	Atomic Type	X-Coordinate (Å)	Y-Coordinate (Å)	Z-Coordinate (Å)
1	6	0	0.985018	-1.115938	-0.000178
2	1	0	0.803674	-2.179931	-0.000308
3	6	0	0.028949	-0.069245	0.000172
4	6	0	0.791133	1.089267	0.000280
5	1	0	0.518500	2.132847	0.000408
6	7	0	2.216835	-0.643824	-0.000219
7	7	0	2.071716	0.695015	0.000057
8	6	0	-1.433962	-0.201243	0.000093
9	8	0	-1.960000	-1.299204	0.000234
10	6	0	-2.245068	1.069517	-0.000286
11	1	0	-2.001688	1.668307	0.880562
12	1	0	-2.002731	1.666873	-0.882406
13	1	0	-3.305811	0.828449	0.000519
14	1	0	2.891779	1.284610	0.000004

Table S33: 4-Acetylpyrazole (Transition State). Standard orientation.

Center Number	Atomic Number	Atomic Type	X-Coordinate (Å)	Y-Coordinate (Å)	Z-Coordinate (Å)
1	6	0	0.829363	1.774437	-0.545501
2	1	0	1.450700	2.160138	-1.339037
3	6	0	0.973584	0.557852	0.150110
4	6	0	-0.125729	0.528060	1.034333
5	1	0	-0.304558	-0.079151	1.910711
6	7	0	-0.203592	2.473827	-0.099271
7	7	0	-0.735696	1.731035	0.881637
8	6	0	-1.487844	-0.728342	-0.181191
9	9	0	-0.875954	-1.813187	-0.634276
10	9	0	-1.856465	0.032875	-1.197357
11	9	0	-2.551139	-1.075924	0.528008
12	6	0	2.025622	-0.450385	-0.031365
13	8	0	2.863923	-0.316887	-0.904471
14	6	0	2.037225	-1.619994	0.918168
15	1	0	2.209653	-1.260120	1.935915
16	1	0	1.073841	-2.133897	0.908179
17	1	0	2.827137	-2.312994	0.637024
18	1	0	-1.554429	2.063454	1.371714

Table S34: 4-Acetyl-5-trifluoromethylpyrazole (Adduct Radical). Standard orientation.

Center Number	Atomic Number	Atomic Type	X-Coordinate (Å)	Y-Coordinate (Å)	Z-Coordinate (Å)
1	6	0	-0.898064	1.860149	0.249093
2	6	0	-0.884007	0.523751	-0.108182
3	6	0	0.481602	0.274038	-0.691362
4	1	0	0.482435	-0.116539	-1.716449
5	7	0	0.235695	2.506626	-0.064018
6	7	0	1.040922	1.610237	-0.615947
7	6	0	1.353940	-0.665735	0.147388
8	9	0	1.403768	-0.290173	1.427477
9	9	0	2.610999	-0.679456	-0.315483
10	9	0	0.895128	-1.919953	0.107664
11	1	0	1.932489	1.899048	-0.991956
12	6	0	-2.032539	-0.371416	-0.015218
13	8	0	-3.049093	0.001918	0.555427
14	6	0	-1.955666	-1.743063	-0.637167
15	1	0	-1.293557	-1.773087	-1.502953
16	1	0	-1.570242	-2.447619	0.104172
17	1	0	-2.959110	-2.055075	-0.922094
18	1	0	-1.716243	2.399777	0.701390

Table S35: 4-Acetyl-5-trifluoromethylpyrazole (Adduct Cation). Standard orientation.

Center Number	Atomic Number	Atomic Type	X-Coordinate (Å)	Y-Coordinate (Å)	Z-Coordinate (Å)
1	6	0	-0.968566	1.853340	0.179796
2	6	0	-0.887544	0.541569	-0.125302
3	6	0	0.494116	0.309838	-0.637940
4	1	0	0.509012	0.034667	-1.702249
5	7	0	0.247544	2.497566	-0.066477
6	7	0	1.049003	1.651246	-0.505777
7	6	0	1.381007	-0.681548	0.141108
8	9	0	1.331317	-0.434892	1.443914
9	9	0	2.640430	-0.560863	-0.264562
10	9	0	0.976740	-1.922880	-0.078114
11	1	0	2.002931	1.927270	-0.755128
12	6	0	-2.070705	-0.404740	-0.014237
13	8	0	-3.047309	0.030933	0.537229
14	6	0	-1.968060	-1.785618	-0.577243
15	1	0	-1.432805	-1.803427	-1.527177
16	1	0	-1.419160	-2.410856	0.132551
17	1	0	-2.970525	-2.188923	-0.700496
18	1	0	-1.804672	2.422791	0.552211

Table S36: Pyrazole. Standard orientation.

Center Number	Atomic Number	Atomic Type	X-Coordinate (Å)	Y-Coordinate (Å)	Z-Coordinate (Å)
1	6	0	-1.102435	-0.368713	0.000055
2	6	0	-0.688283	0.979241	-0.000300
3	6	0	0.690060	0.914725	0.000073
4	1	0	1.450848	1.679529	-0.000158
5	7	0	-0.063596	-1.195817	0.000026
6	7	0	1.010041	-0.394557	0.000170
7	1	0	1.937945	-0.797481	-0.000797
8	1	0	-2.107112	-0.765535	-0.000249
9	1	0	-1.302848	1.864583	0.000860

Table S37: Pyrazole (Transition State). Standard orientation.

Center Number	Atomic Number	Atomic Type	X-Coordinate (Å)	Y-Coordinate (Å)	Z-Coordinate (Å)
1	6	0	-2.089017	-0.670648	0.461796
2	6	0	-1.239855	-1.118544	-0.554606
3	6	0	-0.645450	0.049075	-1.060218
4	1	0	-0.107620	0.220684	-1.982124
5	7	0	-2.085233	0.661578	0.555989
6	7	0	-1.249538	1.075883	-0.399617
7	6	0	1.266634	-0.017541	0.043270
8	9	0	0.973885	0.079737	1.335889
9	9	0	2.039077	1.011153	-0.300940
10	9	0	1.932268	-1.148331	-0.168927
11	1	0	-1.065417	2.064633	-0.514895
12	1	0	-2.708689	-1.252917	1.127568
13	1	0	-1.035818	-2.131718	-0.860809

Table S38: 3-(Trifluoromethyl)pyrazole (Adduct Radical). Standard orientation.

Center Number	Atomic Number	Atomic Type	X-Coordinate (Å)	Y-Coordinate (Å)	Z-Coordinate (Å)
1	6	0	-2.237002	0.646308	-0.195640
2	6	0	-1.086574	1.168006	0.312176
3	6	0	-0.218732	0.003694	0.678389
4	1	0	-0.000762	-0.067139	1.753291
5	7	0	-2.254258	-0.714064	-0.215663
6	7	0	-1.033260	-1.101853	0.203120
7	6	0	1.130582	0.012624	-0.030662
8	9	0	1.010875	0.105994	-1.358314
9	9	0	1.823339	-1.106453	0.226988
10	9	0	1.873543	1.048981	0.381626
11	1	0	-0.963123	-2.027975	0.600597
12	1	0	-0.820083	2.203182	0.450144
13	1	0	-3.102855	1.182849	-0.554503

Table S39: 3-(Trifluoromethyl)pyrazole (Adduct Cation). Standard orientation.

Center Number	Atomic Number	Atomic Type	X-Coordinate (Å)	Y-Coordinate (Å)	Z-Coordinate (Å)
1	6	0	-2.239855	0.644501	-0.204240
2	6	0	-1.105137	1.172253	0.289990
3	6	0	-0.224620	0.040029	0.661121
4	1	0	-0.059730	-0.016079	1.746232
5	7	0	-2.164003	-0.752354	-0.203666
6	7	0	-1.052312	-1.084857	0.255405
7	6	0	1.143763	0.018062	-0.038176
8	9	0	1.005576	0.064262	-1.359082
9	9	0	1.803261	-1.091117	0.274917
10	9	0	1.849699	1.068384	0.355363
11	1	0	-0.814531	-2.074479	0.351966
12	1	0	-0.847749	2.212804	0.419578
13	1	0	-3.135518	1.115400	-0.572908

Table S40: 3-Acetylpyrazole. Standard orientation.

Center Number	Atomic Number	Atomic Type	X-Coordinate (Å)	Y-Coordinate (Å)	Z-Coordinate (Å)
1	6	0	0.056284	0.101427	0.000020
2	6	0	0.996110	1.157620	0.000964
3	6	0	2.215955	0.525234	0.000082
4	1	0	3.226507	0.899307	0.000446
5	7	0	0.659448	-1.086916	0.000057
6	7	0	1.955694	-0.805091	-0.000530
7	6	0	-1.421136	0.205454	-0.000205
8	8	0	-1.951196	1.298548	-0.000983
9	6	0	-2.209911	-1.075572	0.000528
10	1	0	-1.948084	-1.672835	-0.874991
11	1	0	-1.953696	-1.667472	0.881413
12	1	0	-3.273403	-0.846949	-0.003316
13	1	0	0.798021	2.215859	0.001606
14	1	0	2.630423	-1.557219	-0.002312

Table S41: 3-Acetylpyrazole (Transition State). Standard orientation.

Center Number	Atomic Number	Atomic Type	X-Coordinate (Å)	Y-Coordinate (Å)	Z-Coordinate (Å)
1	6	0	-1.253565	0.189008	-0.351854
2	6	0	-0.276353	-0.329490	-1.213526
3	6	0	0.746697	0.627745	-1.199853
4	1	0	1.562088	0.785567	-1.890877
5	7	0	-0.916854	1.399904	0.107984
6	7	0	0.255418	1.665148	-0.450210
7	6	0	2.107812	-0.298871	0.238720
8	9	0	2.422918	-1.532743	-0.130711
9	9	0	1.464667	-0.333916	1.398121
10	9	0	3.217793	0.418778	0.375170
11	6	0	-2.536203	-0.446924	0.042682
12	8	0	-2.823944	-1.539787	-0.398780
13	6	0	-3.426308	0.312341	0.985927
14	1	0	-2.893783	0.507067	1.919115
15	1	0	-3.682488	1.281830	0.554030
16	1	0	-4.327301	-0.265331	1.179449
17	1	0	-0.281255	-1.274626	-1.729681
18	1	0	0.723474	2.536496	-0.242009

Table S42: 3-Acetyl-5-(trifluoromethyl)-pyrazole (Adduct Radical). Standard orientation.

Center Number	Atomic Number	Atomic Type	X-Coordinate (Å)	Y-Coordinate (Å)	Z-Coordinate (Å)
1	6	0	1.291083	-0.054567	-0.226902
2	6	0	0.239527	0.734541	-0.609645
3	6	0	-0.933038	-0.179065	-0.774167
4	1	0	-1.333839	-0.211657	-1.797183
5	7	0	0.968206	-1.369734	-0.122099
6	7	0	-0.342774	-1.443911	-0.366480
7	6	0	-2.107488	0.178773	0.134244
8	9	0	-1.748713	0.261514	1.418181
9	9	0	-3.079580	-0.739696	0.050285
10	9	0	-2.632169	1.358313	-0.218476
11	1	0	-0.726328	-2.344781	-0.615149
12	6	0	2.685578	0.401370	0.046995
13	8	0	2.964961	1.573213	-0.081715
14	1	0	0.233521	1.799378	-0.775619
15	6	0	3.680307	-0.638460	0.474986
16	1	0	3.337505	-1.118364	1.394235
17	1	0	3.749045	-1.418773	-0.285810
18	1	0	4.650723	-0.172725	0.630328

Table S43: 3-Acetyl-5-(trifluoromethyl)-pyrazole (Adduct Cation). Standard orientation.

Center Number	Atomic Number	Atomic Type	X-Coordinate (Å)	Y-Coordinate (Å)	Z-Coordinate (Å)
1	6	0	1.297247	-0.013896	-0.218178
2	6	0	0.249424	0.757077	-0.578800
3	6	0	-0.912887	-0.143345	-0.744471
4	1	0	-1.246821	-0.192431	-1.791719
5	7	0	0.899098	-1.352258	-0.113661
6	7	0	-0.314733	-1.422318	-0.383888
7	6	0	-2.132477	0.180628	0.138402
8	9	0	-1.792726	0.207336	1.421879
9	9	0	-3.073351	-0.740531	-0.027946
10	9	0	-2.614431	1.364716	-0.204525
11	1	0	-0.786941	-2.329952	-0.382773
12	6	0	2.718989	0.397946	0.056937
13	8	0	2.985822	1.567685	-0.039689
14	1	0	0.235050	1.826466	-0.735027
15	6	0	3.682574	-0.683776	0.424615
16	1	0	3.322805	-1.219050	1.306589
17	1	0	3.747403	-1.409156	-0.390576
18	1	0	4.658731	-0.246813	0.618162

Table S44: 4-Nitropyrazole. Standard orientation.

Center Number	Atomic Number	Atomic Type	X-Coordinate (Å)	Y-Coordinate (Å)	Z-Coordinate (Å)
1	6	0	-0.816602	1.139354	-0.000086
2	1	0	-0.542052	2.182002	-0.000270
3	6	0	0.023833	0.007515	-0.000218
4	6	0	-0.804264	-1.098428	-0.000046
5	1	0	-0.594556	-2.154989	0.000066
6	7	0	-2.076764	0.755809	0.000037
7	7	0	-2.039146	-0.591235	0.000096
8	1	0	-2.906341	-1.110807	0.000342
9	7	0	1.449171	-0.011575	-0.000018
10	8	0	2.007809	-1.094026	-0.000022
11	8	0	2.028731	1.059296	0.000167

Table S45: 4-Nitropyrazole (Transition State). Standard orientation.

Center Number	Atomic Number	Atomic Type	X-Coordinate (Å)	Y-Coordinate (Å)	Z-Coordinate (Å)
1	6	0	1.341160	1.498303	-0.569273
2	1	0	2.095648	1.640750	-1.326151
3	6	0	1.083208	0.359889	0.206349
4	6	0	-0.044821	0.650499	0.992329
5	1	0	-0.409186	0.172610	1.889882
6	7	0	0.487853	2.458668	-0.252620
7	7	0	-0.297023	1.948989	0.708386
8	6	0	-1.567369	-0.477244	-0.170925
9	9	0	-0.994483	-1.141225	-1.156350
10	9	0	-2.417231	0.409078	-0.650164
11	9	0	-2.178375	-1.308377	0.649719
12	1	0	-1.021823	2.524868	1.115164
13	7	0	1.716440	-0.905960	0.140402
14	8	0	1.233313	-1.811307	0.803173
15	8	0	2.694463	-1.022952	-0.577148

Table S46: 4-Nitro-5-(trifluoromethyl)-pyrazole (Adduct Radical). Standard orientation.

Center Number	Atomic Number	Atomic Type	X-Coordinate (Å)	Y-Coordinate (Å)	Z-Coordinate (Å)
1	6	0	-1.174092	1.671647	0.305711
2	6	0	-0.956052	0.371991	-0.093553
3	6	0	0.413291	0.287156	-0.684868
4	1	0	0.437830	-0.071658	-1.720383
5	7	0	-0.120769	2.451391	0.014403
6	7	0	0.781980	1.687042	-0.567018
7	6	0	1.415683	-0.551361	0.125763
8	9	0	1.406196	-0.205952	1.415009
9	9	0	2.653684	-0.344534	-0.339606
10	9	0	1.158729	-1.851976	0.043738
11	1	0	1.649458	2.094194	-0.890697
12	1	0	-2.056090	2.085488	0.768345
13	7	0	-1.897386	-0.666016	-0.094866
14	8	0	-1.585179	-1.718870	-0.641192
15	8	0	-2.982124	-0.464803	0.439257

Table S47: 4-Nitro-5-(trifluoromethyl)-pyrazole (Adduct Cation). Standard orientation.

Center Number	Atomic Number	Atomic Type	X-Coordinate (Å)	Y-Coordinate (Å)	Z-Coordinate (Å)
1	6	0	-1.174291	1.698767	0.249943
2	6	0	-0.947280	0.432090	-0.114281
3	6	0	0.429836	0.310294	-0.653643
4	1	0	0.454994	0.031100	-1.716342
5	7	0	-0.014753	2.455572	-0.009919
6	7	0	0.842288	1.699802	-0.498844
7	6	0	1.405887	-0.601659	0.137735
8	9	0	1.424460	-0.249441	1.416653
9	9	0	2.625213	-0.458908	-0.361970
10	9	0	1.032630	-1.861225	0.039290
11	1	0	1.760337	2.076289	-0.760754
12	1	0	-2.052206	2.174774	0.657316
13	7	0	-1.923583	-0.650584	-0.093945
14	8	0	-1.648170	-1.624092	-0.753428
15	8	0	-2.916383	-0.466718	0.562734

Table S48: 6t-SM. Standard orientation.

Center Number	Atomic Number	Atomic Type	X-Coordinate (Å)	Y-Coordinate (Å)	Z-Coordinate (Å)
1	6	0	-0.236240	-0.300558	0.000001
2	6	0	-1.179221	0.749407	-0.000128
3	6	0	-2.380701	0.070645	-0.000068
4	1	0	-3.397118	0.431058	0.000000
5	7	0	-0.780521	-1.502281	0.000040
6	7	0	-2.095160	-1.250218	0.000036
7	1	0	-2.747615	-2.020108	0.000013
8	6	0	-0.921701	2.220800	0.000053
9	1	0	-0.343798	2.516763	0.878301
10	1	0	-0.355888	2.519455	-0.885128
11	1	0	-1.861846	2.771934	0.007291
12	35	0	1.632663	-0.096953	-0.000005

Table S49: 6t-SM (Transition State). Standard orientation.

Center Number	Atomic Number	Atomic Type	X-Coordinate (Å)	Y-Coordinate (Å)	Z-Coordinate (Å)
1	6	0	-0.908037	-0.404519	0.340718
2	6	0	-0.007416	0.469512	0.962723
3	6	0	1.066870	-0.376171	1.292921
4	1	0	1.876752	-0.210951	1.989276
5	7	0	-0.507648	-1.668599	0.330749
6	7	0	0.671810	-1.638630	0.959334
7	6	0	2.382866	0.121580	-0.410015
8	9	0	1.749390	-0.293571	-1.499837
9	9	0	3.551322	-0.505828	-0.318674
10	9	0	2.592513	1.430398	-0.494995
11	1	0	1.195934	-2.494192	1.071696
12	6	0	-0.110815	1.945716	1.139150
13	1	0	-0.050578	2.458771	0.175389
14	1	0	-1.064712	2.212998	1.598156
15	1	0	0.694890	2.314320	1.773327
16	35	0	-2.553750	0.075827	-0.421996

Table S50: 6t (Adduct Radical). Standard orientation.

Center Number	Atomic Number	Atomic Type	X-Coordinate (Å)	Y-Coordinate (Å)	Z-Coordinate (Å)
1	6	0	0.952152	-0.349450	-0.201496
2	6	0	-0.029914	0.556904	-0.469018
3	6	0	-1.250929	-0.281186	-0.734947
4	1	0	-1.622455	-0.178289	-1.764665
5	7	0	0.577414	-1.650095	-0.247364
6	7	0	-0.759036	-1.618682	-0.447000
7	6	0	-2.421218	0.055365	0.182739
8	9	0	-2.068887	0.060336	1.471751
9	9	0	-3.416059	-0.830386	0.043002
10	9	0	-2.915866	1.266672	-0.105794
11	1	0	-1.153058	-2.430456	-0.903078
12	6	0	0.010698	2.040685	-0.519431
13	1	0	1.039490	2.394807	-0.462475
14	1	0	-0.439129	2.407301	-1.445610
15	1	0	-0.550260	2.479811	0.311319
16	35	0	2.743981	0.045850	0.197067

Table S51: 6t (Adduct Cation). Standard orientation.

Center Number	Atomic Number	Atomic Type	X-Coordinate (Å)	Y-Coordinate (Å)	Z-Coordinate (Å)
1	6	0	0.956785	-0.294611	-0.210623
2	6	0	-0.032107	0.600161	-0.481123
3	6	0	-1.245017	-0.227501	-0.745366
4	1	0	-1.594778	-0.128070	-1.782028
5	7	0	0.493456	-1.598336	-0.269112
6	7	0	-0.726430	-1.569583	-0.548428
7	6	0	-2.424231	0.048355	0.202122
8	9	0	-2.039772	-0.040517	1.471246
9	9	0	-3.396371	-0.831105	-0.004727
10	9	0	-2.889284	1.270103	-0.018940
11	1	0	-1.246092	-2.442440	-0.659700
12	6	0	0.017824	2.074050	-0.507937
13	1	0	1.050254	2.416859	-0.530954
14	1	0	-0.528811	2.462473	-1.368045
15	1	0	-0.460922	2.469783	0.394933
16	35	0	2.734300	0.017365	0.202798

Table S52: 6u-SM. Standard orientation.

Center Number	Atomic Number	Atomic Type	X-Coordinate (Å)	Y-Coordinate (Å)	Z-Coordinate (Å)
1	6	0	-1.176675	0.715935	0.000283
2	6	0	-0.208486	-0.314115	0.000069
3	6	0	-0.905566	-1.500202	-0.000194
4	1	0	-0.580810	-2.527355	-0.000228
5	7	0	-2.391863	0.182927	0.000208
6	7	0	-2.204813	-1.145847	-0.000127
7	1	0	-3.003266	-1.762837	0.000107
8	35	0	1.653475	-0.092005	0.000017
9	6	0	-0.949531	2.190138	-0.000157
10	1	0	-0.373833	2.493589	0.876705
11	1	0	-0.389455	2.495460	-0.886505
12	1	0	-1.905969	2.711215	0.008747

Table S53: 6u-SM (Transition State). Standard orientation.

Center Number	Atomic Number	Atomic Type	X-Coordinate (Å)	Y-Coordinate (Å)	Z-Coordinate (Å)
1	6	0	-0.779507	1.595142	0.163896
2	6	0	-0.636681	0.326405	-0.419426
3	6	0	0.534051	0.371047	-1.187242
4	1	0	0.873792	-0.285794	-1.974104
5	7	0	0.192507	2.400421	-0.265799
6	7	0	0.934376	1.669783	-1.107380
7	6	0	1.961170	-0.551112	0.243446
8	9	0	1.878257	0.161588	1.358629
9	9	0	3.190429	-0.447870	-0.250932
10	9	0	1.698454	-1.823621	0.510232
11	1	0	1.742428	2.083061	-1.549432
12	35	0	-1.732132	-1.159478	-0.143613
13	6	0	-1.828182	2.054350	1.118593
14	1	0	-2.823163	1.925837	0.687909
15	1	0	-1.788068	1.474567	2.043053
16	1	0	-1.677928	3.106785	1.354330

Table S54: 6u (Adduct Radical). Standard orientation.

Center Number	Atomic Number	Atomic Type	X-Coordinate (Å)	Y-Coordinate (Å)	Z-Coordinate (Å)
1	6	0	-1.034836	1.549128	0.054871
2	6	0	-0.563205	0.293883	-0.226055
3	6	0	0.851075	0.415578	-0.702612
4	1	0	0.994071	0.096732	-1.744409
5	7	0	-0.090531	2.493485	-0.183207
6	7	0	1.046932	1.843394	-0.516677
7	6	0	1.866955	-0.356374	0.137155
8	9	0	1.717079	-0.132413	1.445221
9	9	0	3.116741	-0.000586	-0.190522
10	9	0	1.759443	-1.674792	-0.064385
11	1	0	1.693707	2.356715	-1.099782
12	35	0	-1.511659	-1.303428	-0.110005
13	6	0	-2.389328	1.931366	0.546423
14	1	0	-3.164044	1.441007	-0.044841
15	1	0	-2.517998	1.625310	1.586928
16	1	0	-2.515808	3.010698	0.479938

Table S55: 6u (Adduct Cation). Standard orientation.

Center Number	Atomic Number	Atomic Type	X-Coordinate (Å)	Y-Coordinate (Å)	Z-Coordinate (Å)
1	6	0	-1.022955	1.533617	0.056159
2	6	0	-0.586356	0.276280	-0.229353
3	6	0	0.825971	0.372904	-0.698419
4	1	0	0.940545	0.073441	-1.748242
5	7	0	0.015027	2.428482	-0.188181
6	7	0	1.027319	1.805730	-0.584887
7	6	0	1.863424	-0.383186	0.148856
8	9	0	1.725960	-0.092871	1.437965
9	9	0	3.087651	-0.034379	-0.233323
10	9	0	1.714699	-1.688540	-0.014411
11	1	0	1.879329	2.312305	-0.830569
12	35	0	-1.524985	-1.291008	-0.102670
13	6	0	-2.332846	2.034968	0.538671
14	1	0	-2.978191	1.200297	0.804481
15	1	0	-2.185904	2.669138	1.413260
16	1	0	-2.815938	2.625229	-0.241538

8. References

(1) Frizzo, C. P.; Bacim, C.; Moreira, D. N.; V., R. L.; Zimmer, G. C.; Bonacorso, H. G.; Zanatta, N.; Martins, M. A. P. Sonochemical heating profile for solvents and ionic liquid doped solvents, and their application in the N-alkylation of pyrazoles. *Ultrason. Sonochem.* **2016**, *32*, 432–439.

(2) Tamao, K.; Kawachi, A.; Nakagawa, Y.; Ito, Y. Electronic spectra of (amino)(phenyl)disilanes. *J. Organomet. Chem.* **1994**, *473*, 29–24.

(3) Panteleev, J.; Maguire, R. J.; Kung, D. W. Alkylation of nitrogen-containing heterocycles via in situ sulfonyl transfer. *Synlett* **2015**, *26*, 953–959.

(4) Huang, C.; Kang, C.; Liu, H.-J.; Wang, C.-L.; Tang, S.; Qin, Y.-S.; Wei, Z.; Cai, H. N-Sulfonylation of azoles with sulfonyl hydrazides enabled by electrocatalysis. *Green Chem.* **2024**, *26*, 8706–8710.

(5) Bryan, V. J. Indium-mediated allylations in aqueous media : an expansion of the scope and a demonstration of the synthetic utility of the reaction, PhD thesis, McGill University, 1999.

(6) Nandi, J.; Ovan, J. M.; Kelly, C. B.; Leadbeater, N. E. Oxidative functionalisation of alcohols and aldehydes via the merger of oxammonium cations and photoredox catalysis. *Org. Biomol. Chem.* **2017**, *15*, 8295–8301.

(7) Slack, E. D.; Colacot, T. J. Understanding the activation of air-stable Ir(COD)(Phen)Cl precatalysts for C-H borylation of aromatics and heteroaromatics. *Org. Lett.* **2021**, *23*, 1561–1565.

(8) Amos, S. G. A.; Nicolai, S.; Waser, J. Photocatalytic Umpolung of N- and O-substituted alkenes for the synthesis of 1,2-aminoalcohols and diols. *Chem. Sci.* **2020**, *11*, 11274–11279.

(9) Mistico, L.; Querolle, O.; Meerpoel, L.; Angibaud, P.; Durandetti, M.; Maddaluno, J. Access to Silylated Pyrazole Derivatives by Palladium-Catalyzed C-H Activation of a TMS group. *Chem. Eur. J.* **2016**, *22*, 9687–9692.

(10) Mancano, G.; Page, M. J.; Bhadbhade, M.; Messerle, B. A. Hemilabile and bimetallic coordination in Rh and Ir complexes of NCN pincer ligands. *Inorg. Chem.* **2014**, *53*, 10159–10170.

(11) Mizutani, T.; Yoshimura, C.; Kondo, H.; Kitade, M.; Ohkubo, S. Novel pyrrolopyrimidine compound or salt thereof, pharmaceutical composition containing same, especially agent for prevention and/or treatment of tumors etc. based on NAE inhibitory effect, Canadian Intellectual Property Organization, CA2946833, 2015.

(12) Ge, L.; Zhang, C.; Pan, C.; Wang, D.-X.; Liu, D.-L.; Li, Z.-Q.; Shen, P.; Tian, L.; Feng, C. Photoredox-catalyzed C-C-bond cleavage of cyclopropanes for the formation of C(sp3)-heteroatom bonds. *Nat. Commun.* **2022**, *13*, 5938.

(13) Despotopoulou, C. Br/Mg exchange on 1,2-dibromocyclopentene derivatives and regio-and chemoselective functionalization of pyrazoles and related heterocycles, PhD thesis, Ludwigs-Maximilian Universität München, 2009.

(14) Buesking, A. W.; Sparks, R. B.; Combs, A. P.; Douty, B.; Falahat-Pisheh, N.; Shao, L.; Shepard, S.; Yue, E. W. Heterocyclic compounds as Pi3K-gamma inhibitors, World Patent WO2017/223414, 2017.

(15) Lerner, C.; Li, M.; Liu, Y.; Schmitt, S.; Wang, J.; Wang, M.; Wang, Y.; Yang, S.; Zhou, C. Imidazole-pyrazole derivatives with antibacterial properties, World Intellectial Property Organization, WO2022/049011, 2022.

(16) Liang, J.; Jakalian, A.; Lambrecht, M. J.; Larouche-Gauthier, R.; Huestis, M.; Ung, M. U.; Wang, X.; Yadav, A.; Zbieg, J. R.; Broccatelli, F. Lactams as CBL-B inhibitors, World Patent WO2022/169997 A1, 2022.

(17) Del Giudice, M. R.; Mustazza, C.; Borioni, A.; Gatta, F.; Tayebati, K.; Amenta, F.; Tucci, P.; Pieretti, S. Synthesis of 1-Methyl-5-(pyrazol-3- and -5-yl- and 1, 2, 4-triazol-3- and 5-yl)-1, 2, 3, 6-tetrahydropyridine Derivatives and Their Evaluation as Muscarinic Receptor Ligands. *Arch. Pharm.* **2003**, *336*, 139–195.

(18) Pradhan, S.; Maiti, S.; Dutta, S.; Russell, C. A.; Tyagi, S.; Maiti, D. A modular approach for accessing 3D-heterocycles via 1,2-dicyanation of planar heteroarenes. *Angew. Chem. Int. Ed.* **2025**, *64*, e202412979.

(19) Letallec, J.-P.; Marguet, F.; Petit, F.; Ronan, B.; Terrier, C. Pyridazine compounds, their preparation and their therapeutic use, World Patent WO2024/188994A1, 2024.

(20) Tang, R.-J.; Milcent, T.; Crousse, B. Regioselective Halogenation of Arenes and Heterocycles in Hexafluoroisopropanol. *J. Org. Chem.* **2017**, *83*, 930–938.

(21) Gu, C.; Yuen, O. Y.; Ng, S. S.; So, C. M. Palladium-catalyzed chemoselective amination of chloro(hetero)aryl triflated enabled by alkyl-pyrazole-based phosphine ligands. *Adv. Synth. Catal.* **2024**, *366*, 1565–1574.

(22) Cotesta, S.; Gerspacher, M.; Leblanc, C.; Lorthois, E. L. J.; Machauer, R.; Mah, R.; Mura, C.; Rigollier, P.; Schneider, N.; Stutz, S.; Vaupel, A.; Warin, N.; Wilcken, R. Pyrazolyl derivatives useful as anti cancer agents, World Patent WO2021/120890A1, 2021.

(23) Tanwar, L.; Börgel, J.; Lehmann, J.; Ritter, T. Selective C-H iodination of (hetero)arenes. *Org. Lett.* **2021**, *23*, 5024–5027.

(24) Kadirova, S. A.; Nuralieva, G. A.; Alieva, M. A.; Talipov, S. A.; Tilyakov, Z. G.; Parpiev, N. A. Synthesis and crystal structure of 2-aminoacetyl-1,3,4-thiadiazole. *Russ. J. Gen. Chem.* **2005**, *75*, 2052–2054.

(25) Schuettelkopf, A. W.; Gros, L.; Blair, D. E.; Frearson, J. A.; van Aalten, D. M. F.; Gilbert, I. H. Acetazolamide-based fungal chitinase inhibitors. *Bioorg. Med. Chem.* **2010**, *18*, 8334–8340.

(26) Guillou, S.; Bonhomme, F. J.; Ermolenko, M. S.; Janin, Y. L. Simple preparations of 4- and 5-iodinated pyrazoles as useful building blocks. *Tetrahedron* **2011**, *67*, 8451–8457.

(27) Yun, C.; Bai, G.; Ning, Y.; Cai, S.; Zhang, T.; Song, P.; Zhou, J.; Duan, W.; Ding, J.; Xie, H.; Zhang, H. Design and synthesis of Imidazo[1,2-b]pyridazine IRAK4 inhibitors for the treatment of mutant MYD88 L265P diffuse large B-cell lymphoma. *Eur. J. Med. Chem.* **2020**, *190*, 112092.

(28) Slobodyanyuk, E. Y.; Artamonov, O. S.; Shishkin, O. V.; Mykhailiuk, P. K. One-Pot Synthesis of CF3-Substituted Pyrazolines/Pyrazoles from Electron-Deficient Alkenes/Alkynes and CF3CHN2 Generated in situ: Optimized Synthesis of Tris(trifluoromethyl)pyrazole. *Eur. J. Org. Chem* **2014**, *2014*, 2487–2495.

(29) Saito, Y. Method for controlling harmful arthropods using heterocyclic compound, Japanese Patent JP2019167374, 2019.

(30) Jeon, S. L.; Choi, J. H.; Kim, B. T.; Jeong, I. H. Synthesis of novel 1,4,5-trisubstituted 3-trifluoromethylpyrazoles via microwave-assisted Stille coupling reactions. *J. Fluor. Chem.* **2007**, *128*, 1191–1197.

(31) Frisch, M.; Trucks, G. W.; Schlegel, H. B.; Scuseria, G. E.; Robb, M. A.; Cheeseman, J. R.; Scalmani, G.; Barone, V.; Petersson, G. A.; Nakatsuji, H.; Li, X.; Caricato, M.; Marenich, A. V.; Bloino, J.; Janesko, B. G.; Gomperts, R.; Menucci, B.; Hratchian, H. P.; Ortiz, J. V.; Izmaylov, A. F.; Sonnenberg, J. L.; Williams-Young, D.; Ding, F.; Lipparini, F.; Egidi, F.; Goings, J.; Peng, B.; Petrone, A.; Henderson, T.; Ranasinghe, D.; Zakrzewski, V. G.; Gao, J.; Rega, N.; Zheng, G.; Liang, W.; Hada, M.; Ehara, M.; Toyota, K.; Fukuda, R.; Hasegawa, J.; Ishida, M.; Nakajima, T.; Honda, Y.; Kitao, O.; Nakai, H.; Vreven, T.; Throssell, K.; Montgomery Jr., J. A.; Peralta, J. E.; Ogliaro, F.; Bearpark, M. J.; Heyd, J. J.; Brothers, E. N.; Kudin, K. N.; Staroverov, V. N.; Keith, T. A.; Kobayashi, R.; Normand, J.; Raghavachari, K.; Rendell, A. P.; Burant, J. C.; Iyengar, S. S.; Tomasi, J.; Cossi, M.; Millam, J. M.; Klene, M.; Adamo, C.; Cammi, R.; Ochterski, J. W.; Martin, R. L.; Morokuma, K.; Farkas, O.; Foresman, J. B.; Fox, D. J. Gaussian 16 Revision A.03, Gaussian Inc. Wallingford CT, 2016.

(32) Zhao, Y.; Truhlar, D. G. The M06 suide of density functionals for main group thermochemistry, thermochemical kinetics, noncovalent interactions, excited states, and transition elements: two new functionals and systematic testing of four M06-class functionals and 12 other functionals. *Theor. Chem. Acc.* **2008**, *120*, 215–241.

(33) Cancès, E.; Mennucci, B.; Tomasi, J. A new integral equation formalism for the polarizable continuum model: Theoretical background and applications to isotropic and anisotropic dielectrics. *J. Chem. Phys* **1997**, *107*, 3032–3041.

(34) Tomasi, J.; Mennucci, B.; Cancès, J. The IEF version of the PCM solvation method: an overview of a new method addressed to study molecular solutes at the QM ab initio level. *J. Mol. Struc.: THEOCHEM* **1999**, *464*, 211–226.

(35) Cossi, M.; Barone, V.; Mennucci, B.; Tomasi, J. Ab initio study of ionic solutions by a polarizable continuum dielectric model. *Chem. Phys. Lett.* **1998**, *286*, 253–260.

9. NMR Spectra

^{1}H NMR of 1-butyl-*1H*-pyrazole (**1b**)

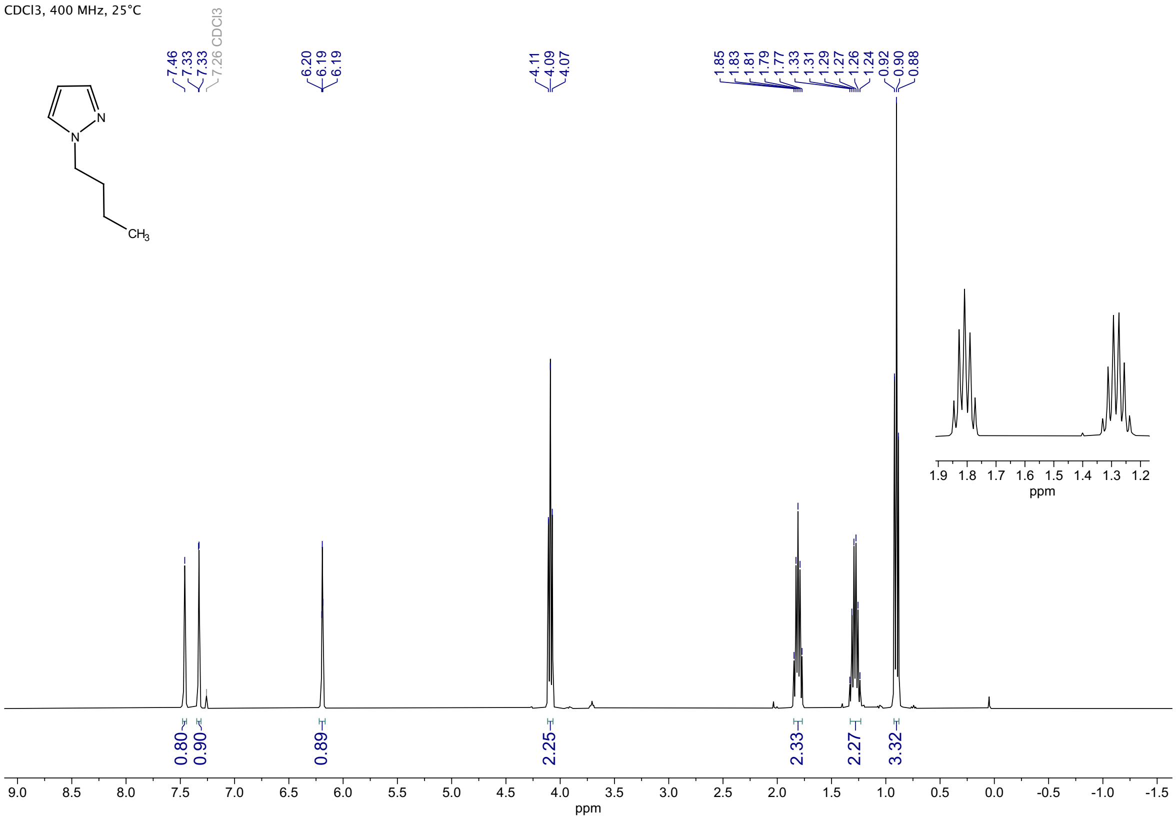

^{13}C NMR of 1-butyl-*1H*-pyrazole (**1b**)

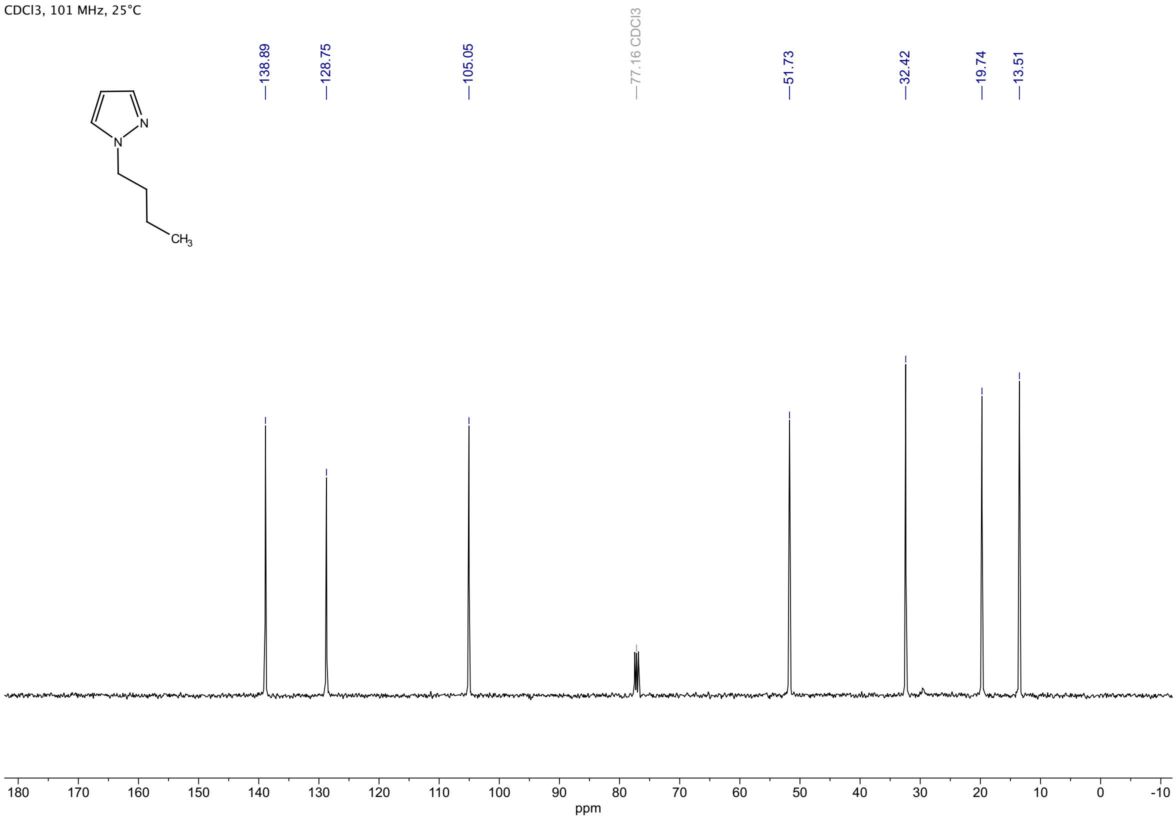

^{1}H NMR of 1-(triisopropylsilyl)-*1H*-pyrazole (**1c**)

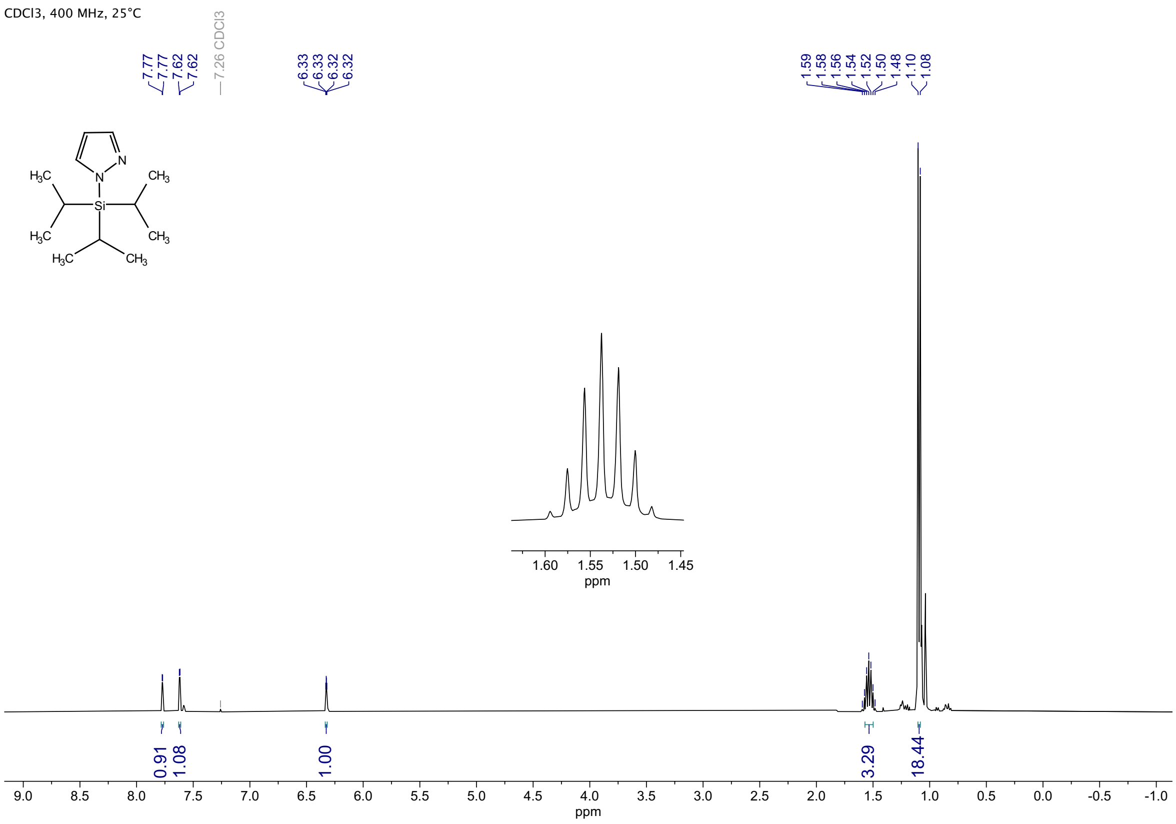

^{13}C NMR of 1-(triisopropylsilyl)-*1H*-pyrazole (**1c**)

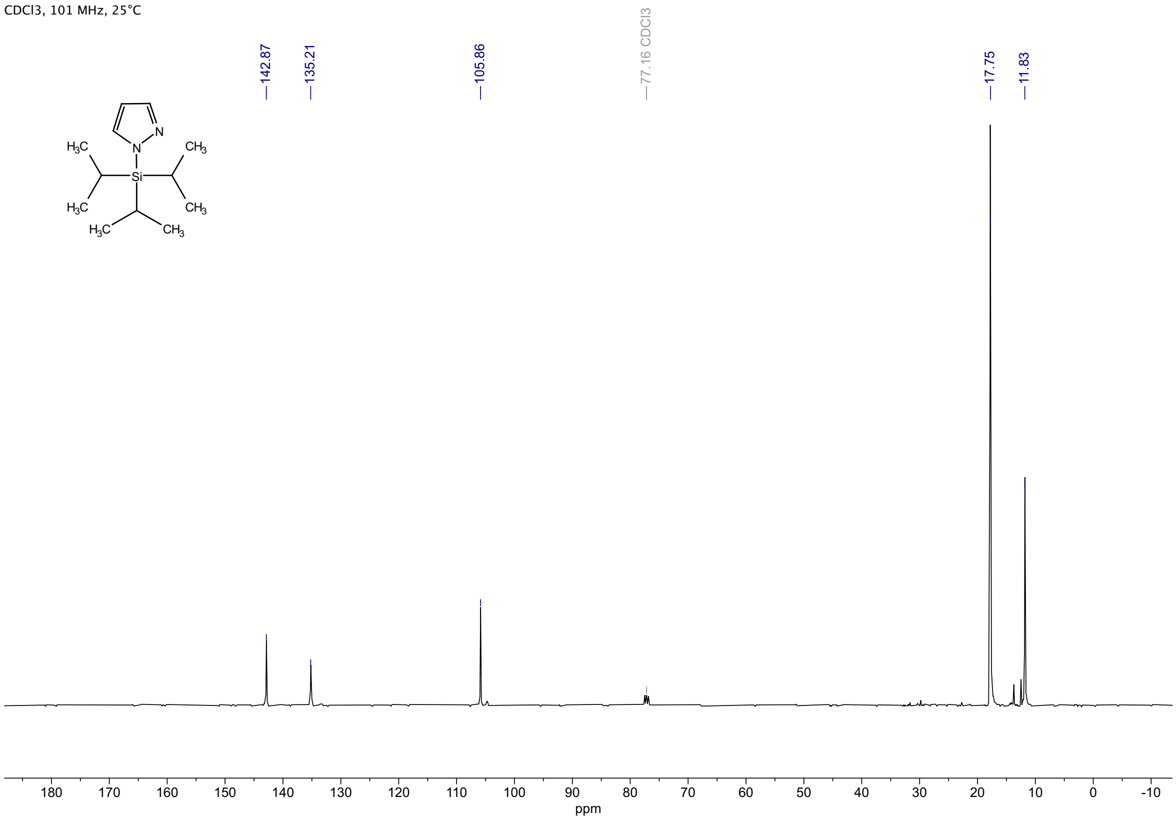

^{1}H NMR of 1-(methylsulfonyl)-*1H*-pyrazole (**1d**)

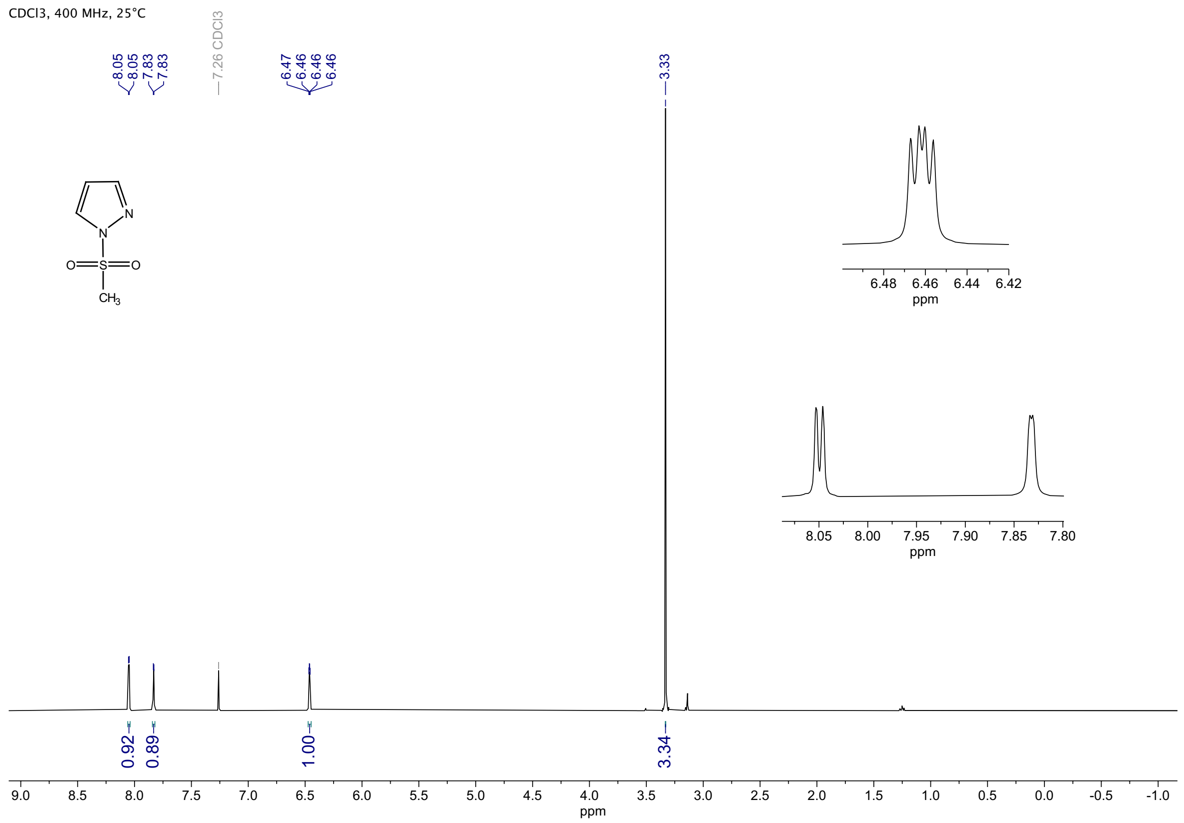

^{13}C NMR of 1-(methylsulfonyl)-*1H*-pyrazole (**1d**)

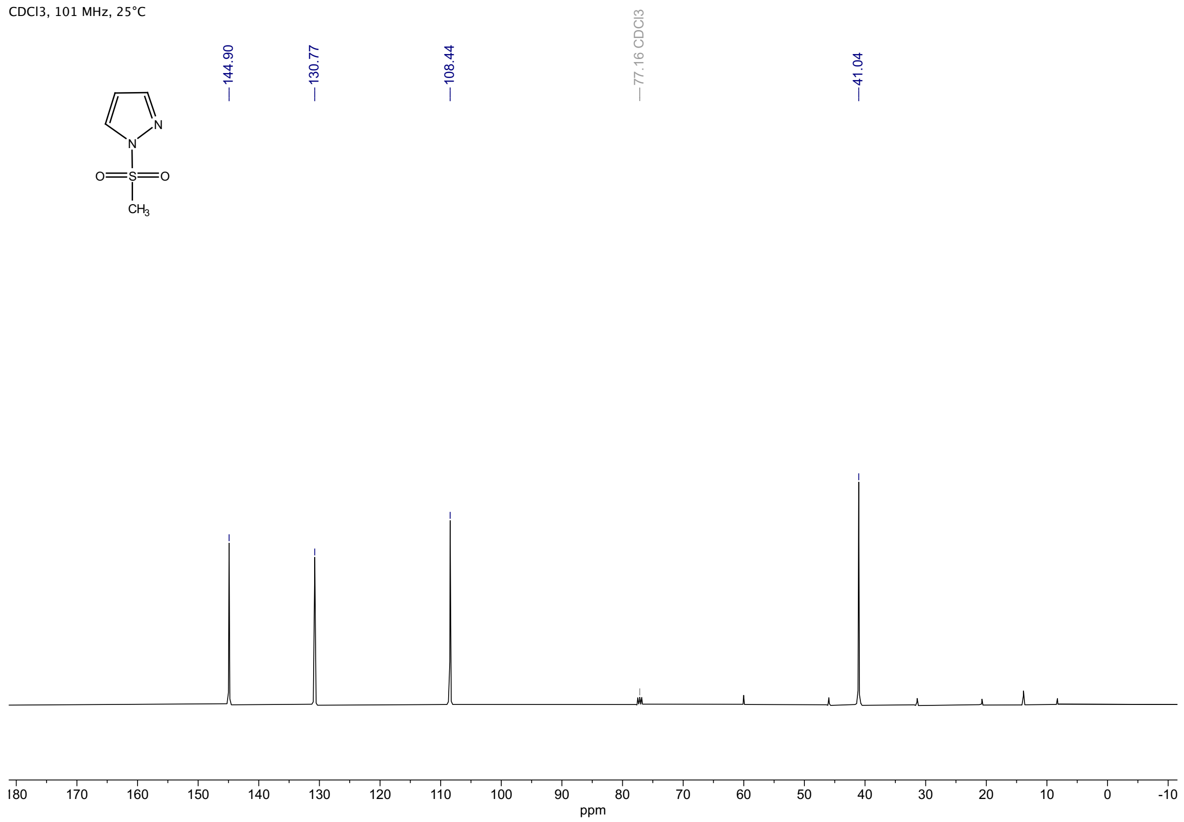

^{1}H NMR of cyclohexyl(*1H*-pyrazol-1-yl)methanone (**1e**)

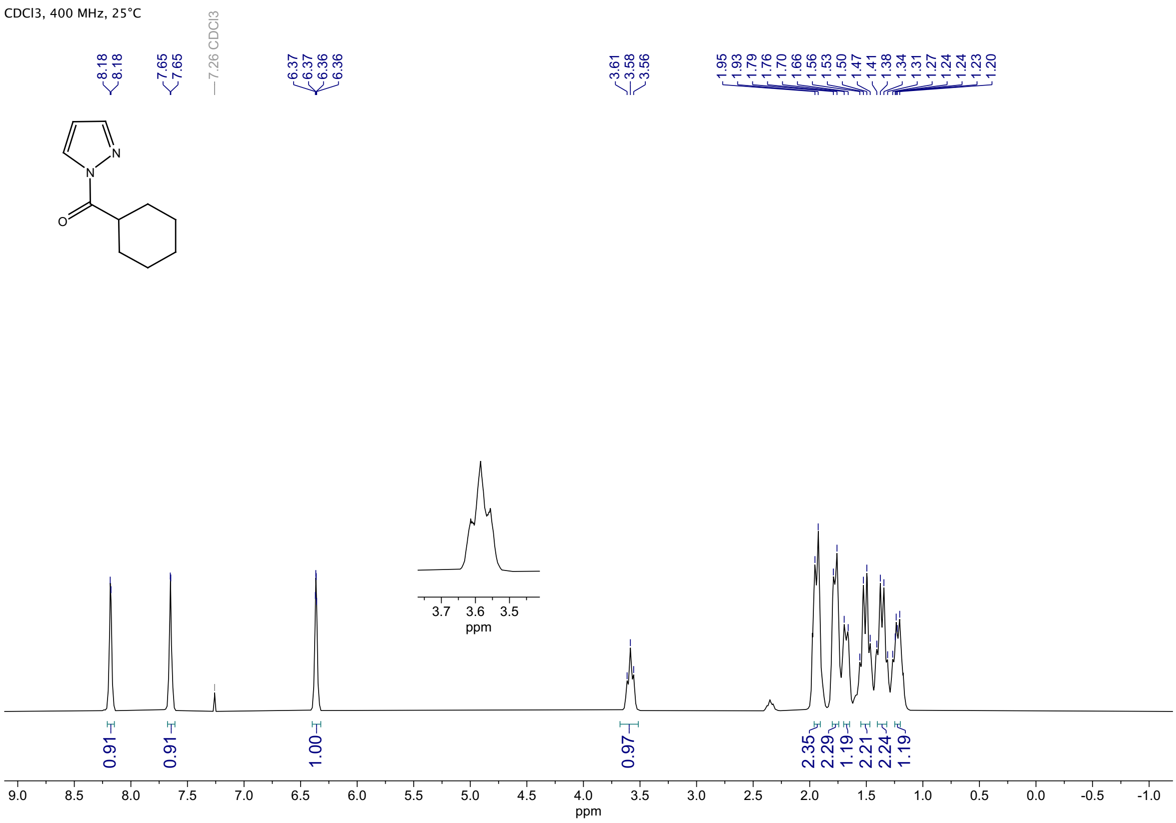

^{13}C NMR of cyclohexyl(*1H*-pyrazol-1-yl)methanone (**1e**)

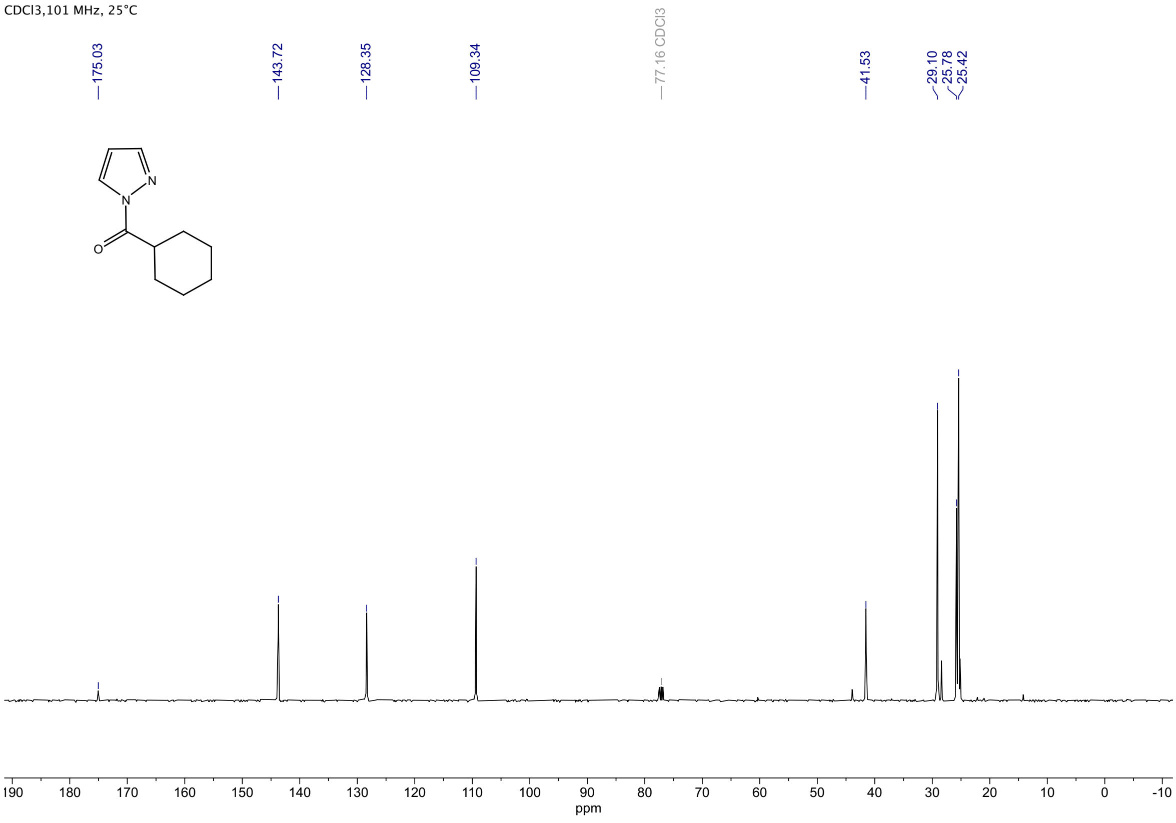

^{1}H NMR of perfluorophenyl(*1H*-pyrazol-1-yl)methanone (**1f**)

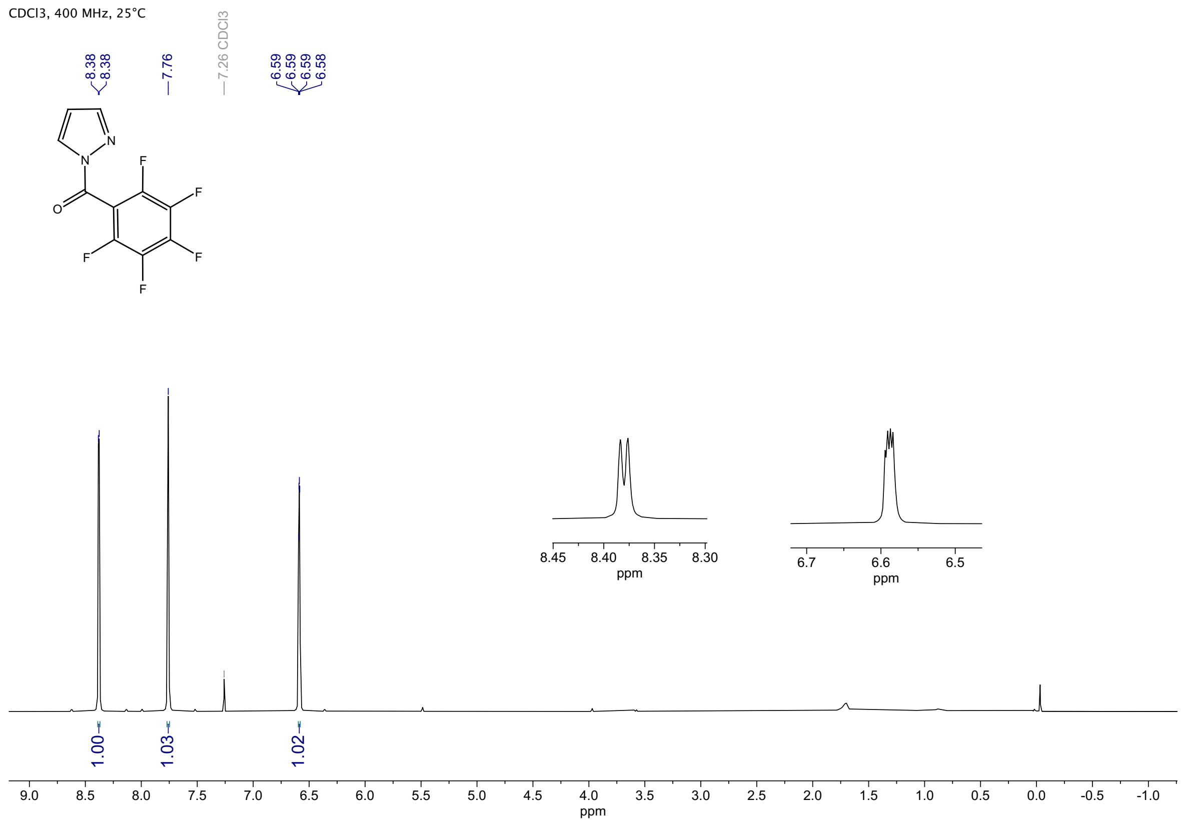

^{13}C NMR of perfluorophenyl(*1H*-pyrazol-1-yl)methanone (**1f**)

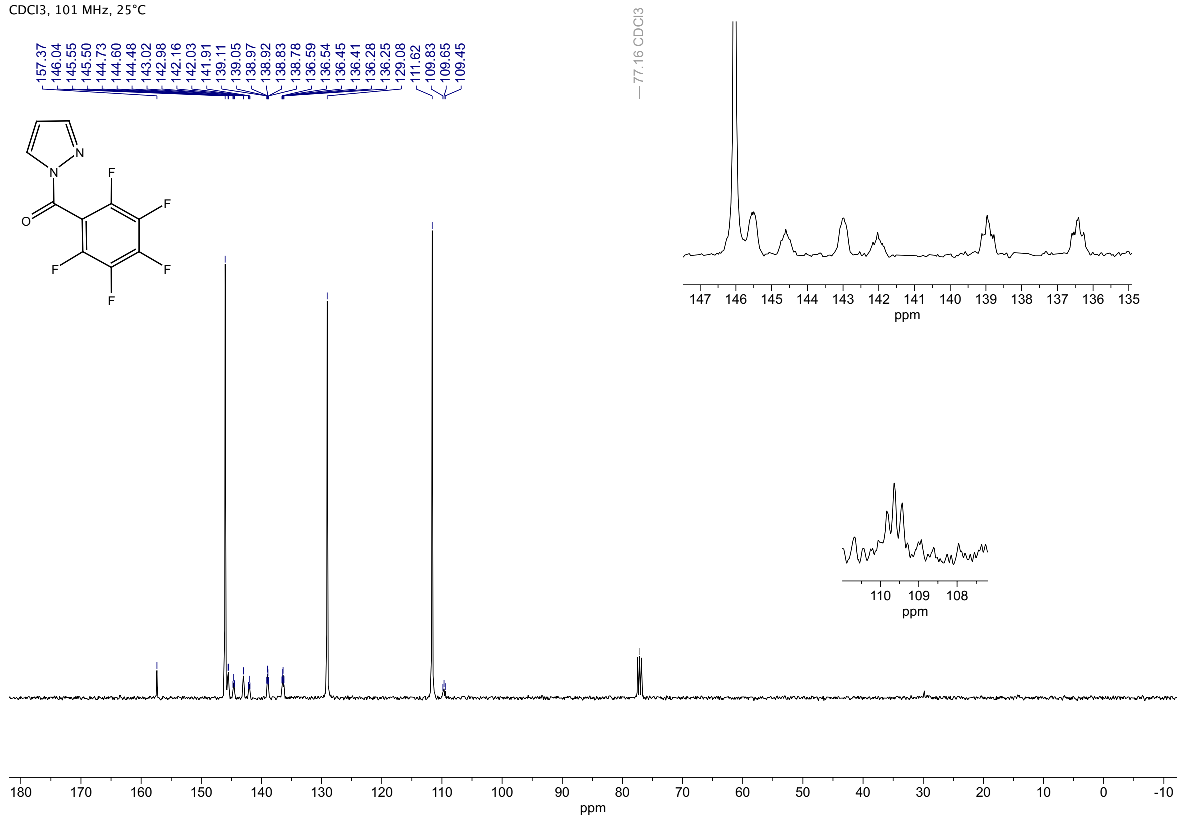

^{19}F NMR of perfluorophenyl(*1H*-pyrazol-1-yl)methanone (**1f**)

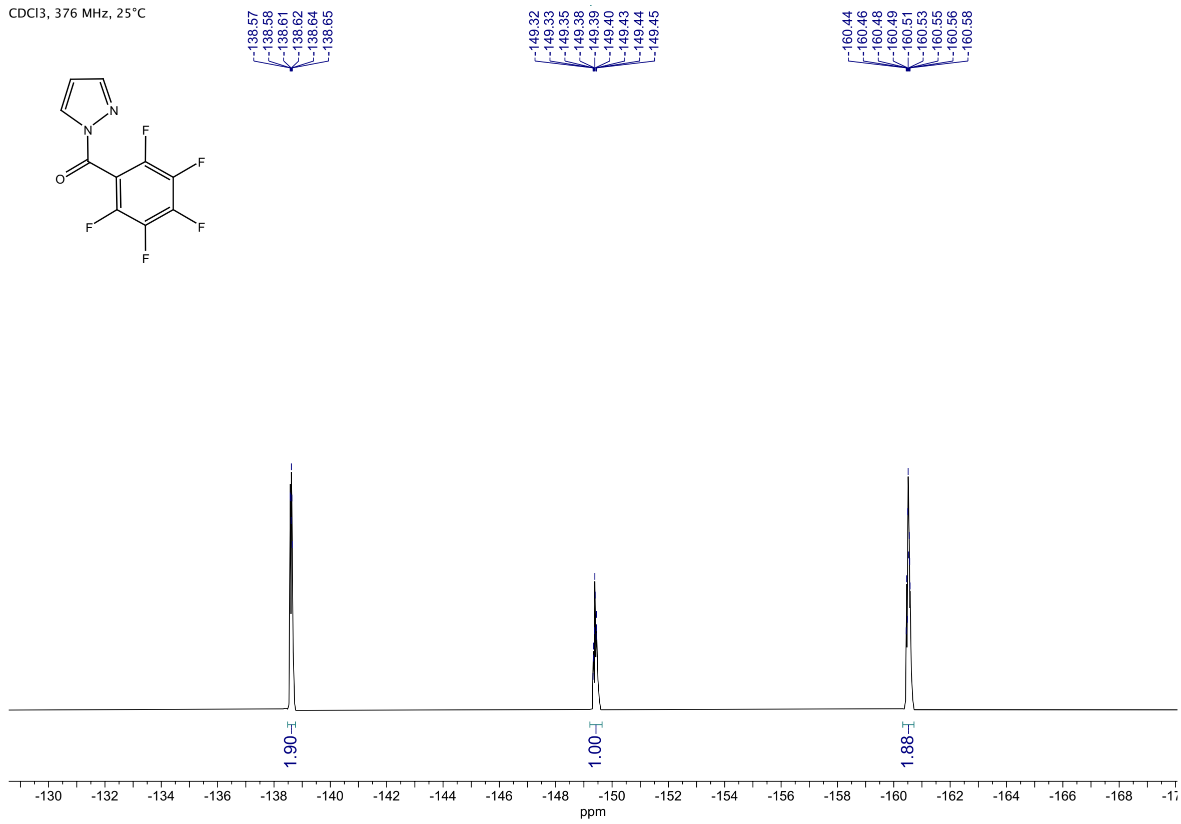

^{1}H NMR of *tert*-butyl *1H*-pyrazole-1-carboxylate (**1g**)

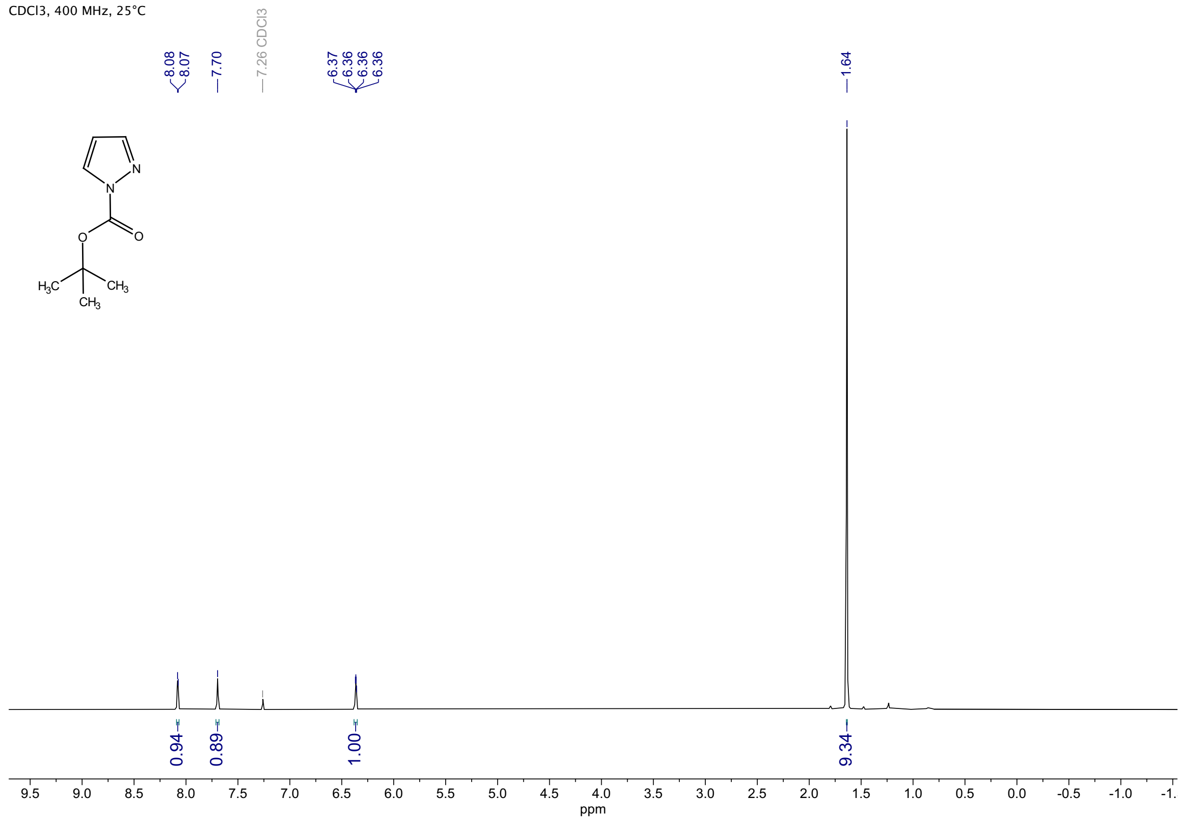

^{13}C NMR of *tert*-butyl *1H*-pyrazole-1-carboxylate (**1g**)

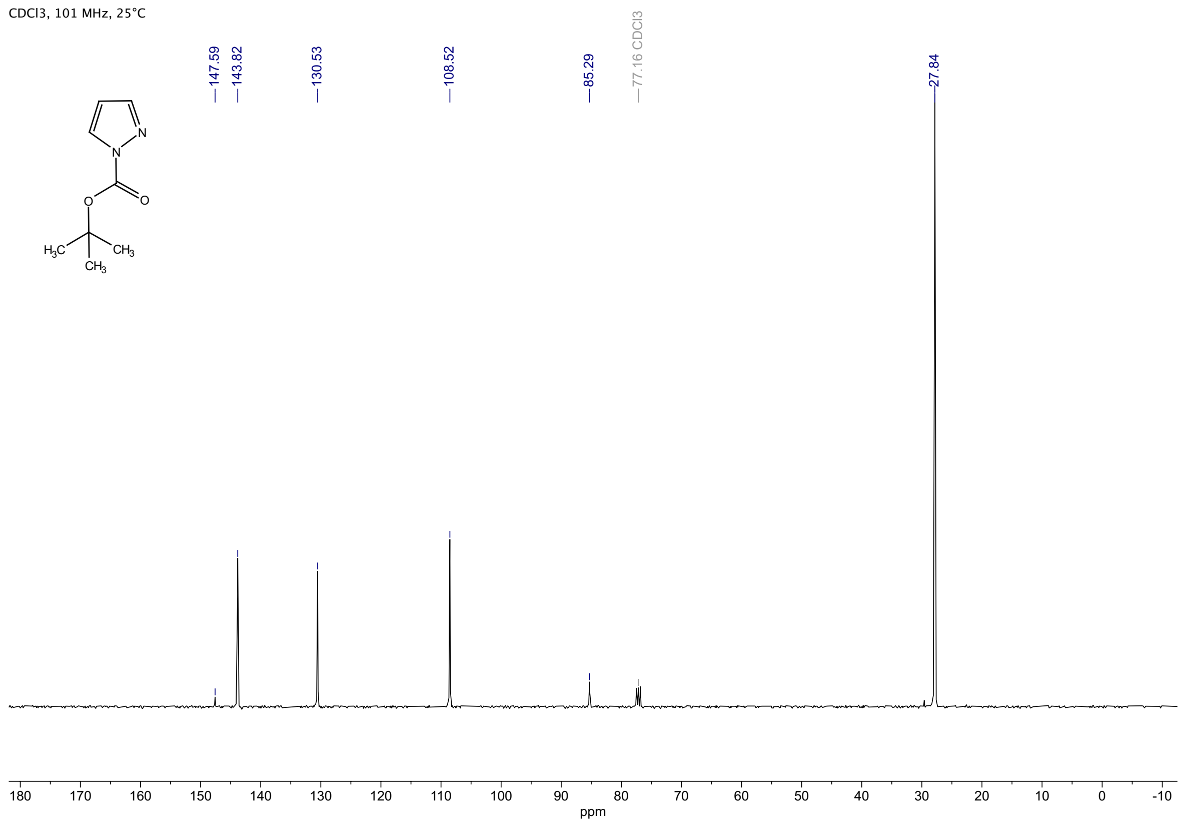

^{1}H NMR of 1-((perfluorophenoxy)methyl)-*1H*-pyrazole (**1h**)

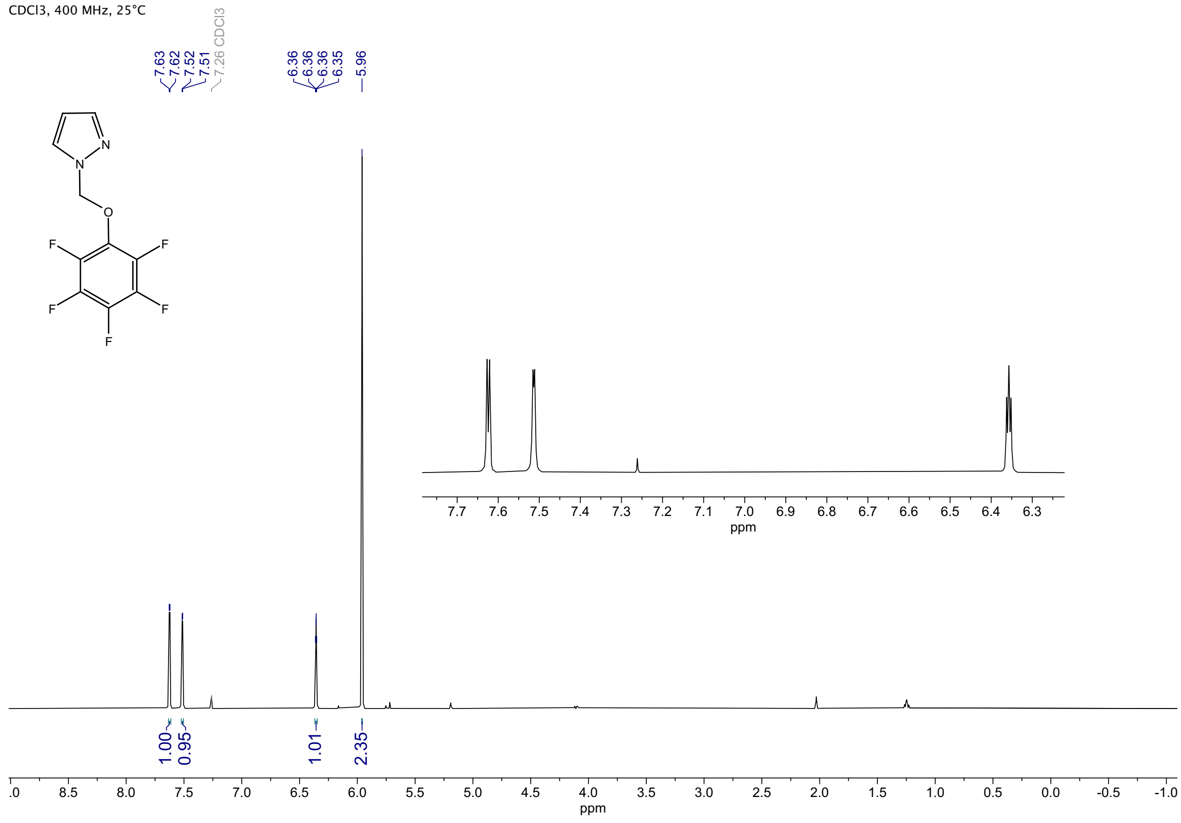

^{13}C NMR of 1-((perfluorophenoxy)methyl)-*1H*-pyrazole (**1h**)

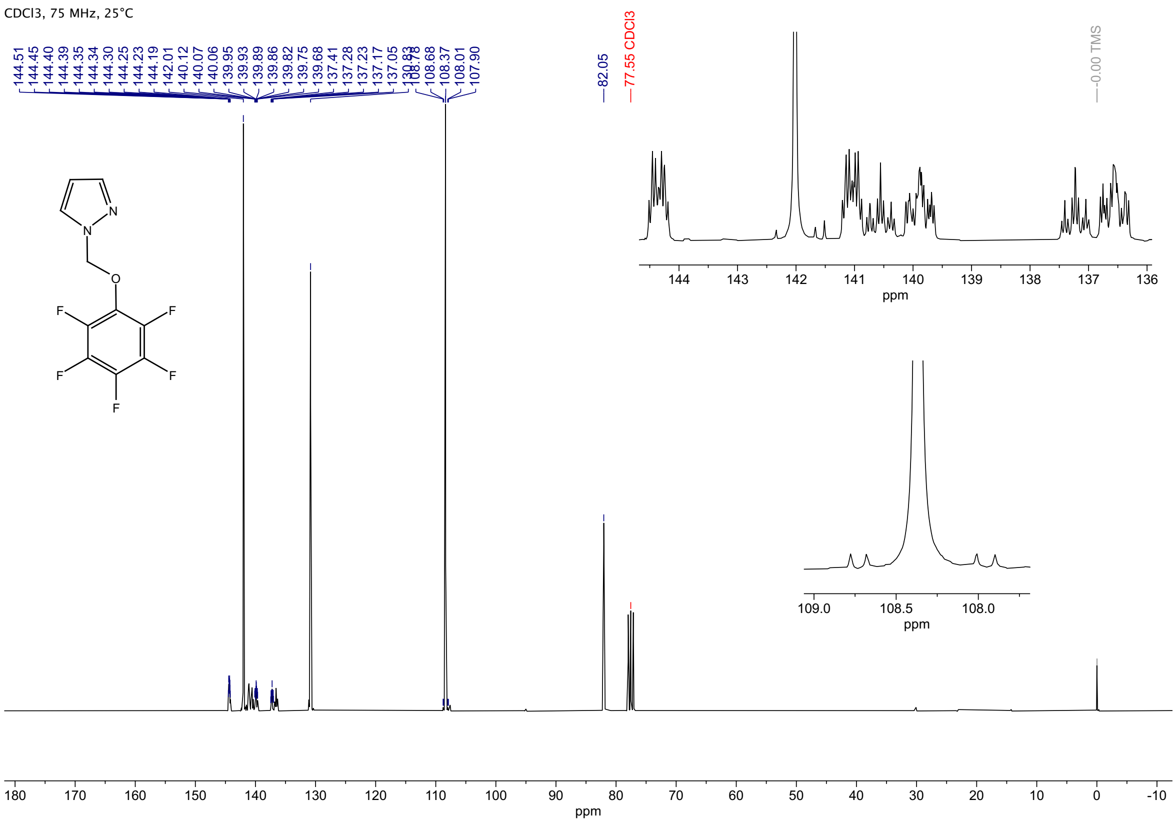

^{19}F NMR of 1-((perfluorophenoxy)methyl)-*1H*-pyrazole (**1h**)

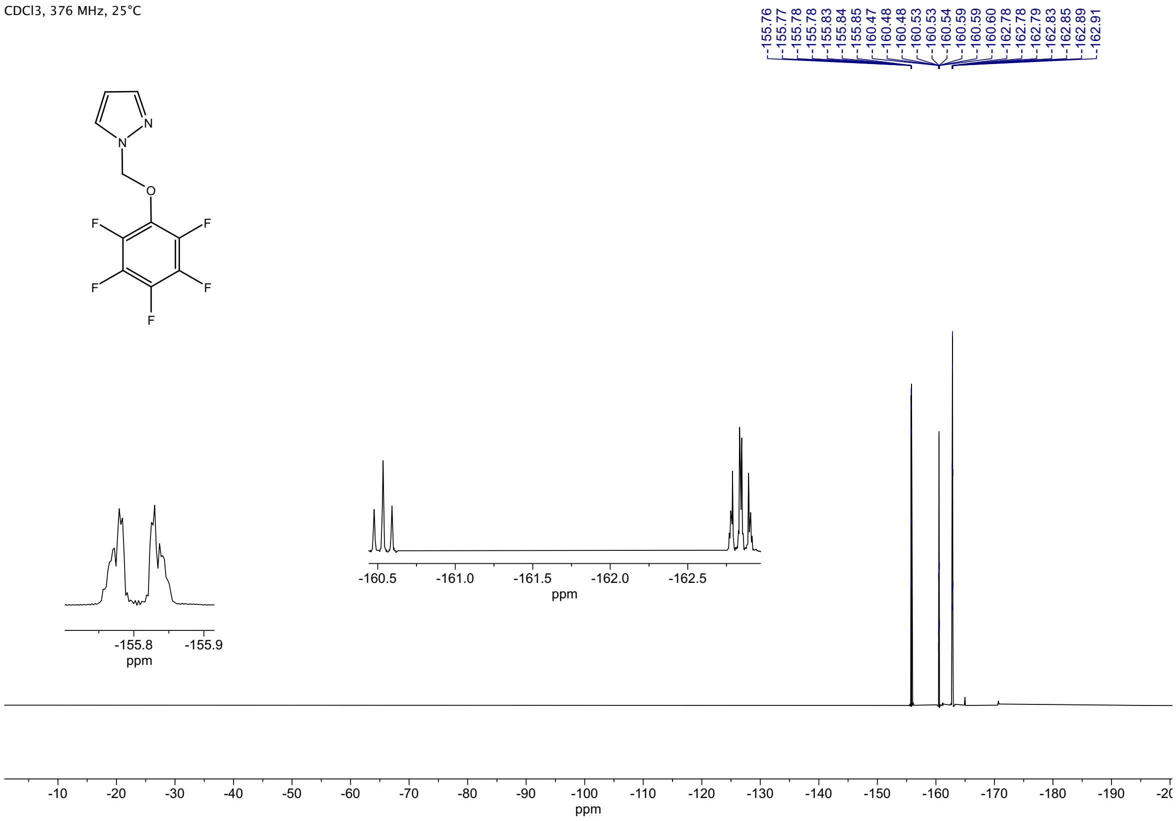

^{1}H NMR of 2,2-dimethyl-1-(*1H*-pyrazol-1-yl)propan-1-one (**1i**)

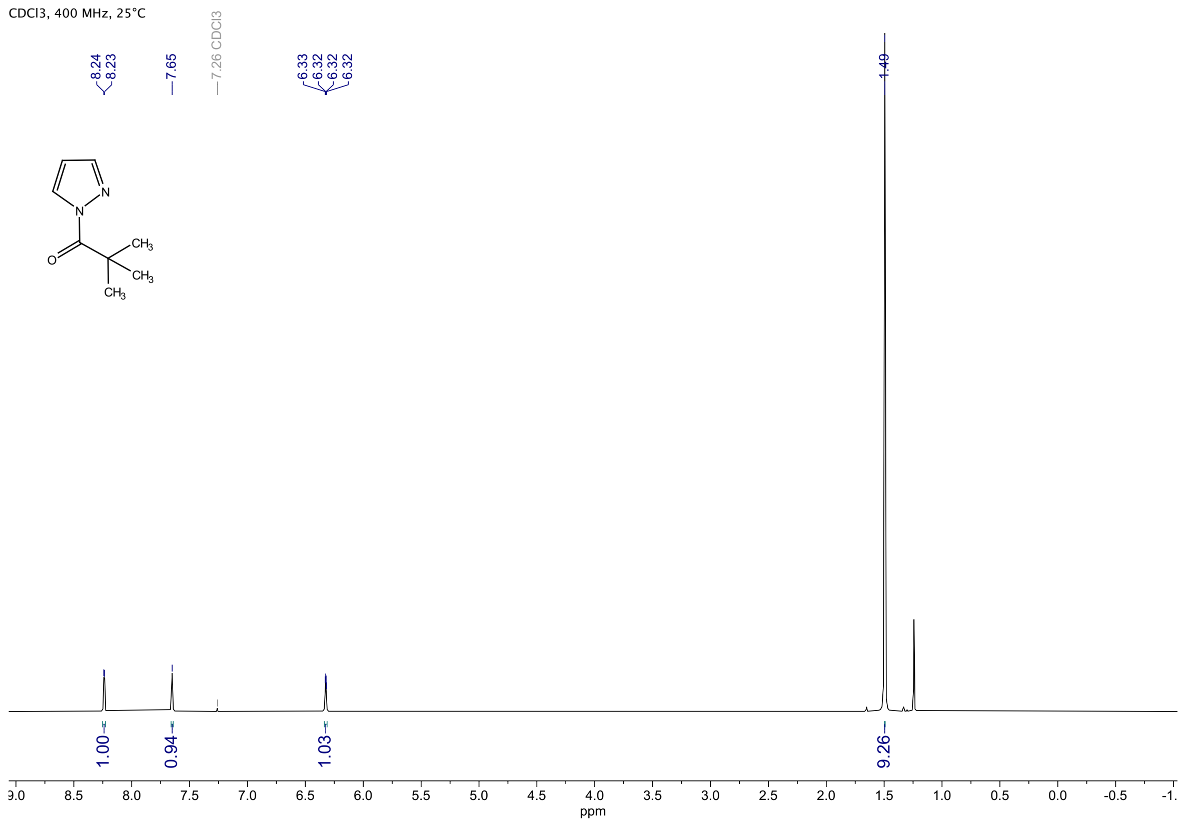

^{13}C NMR of 2,2-dimethyl-1-(*1H*-pyrazol-1-yl)propan-1-one (**1i**)

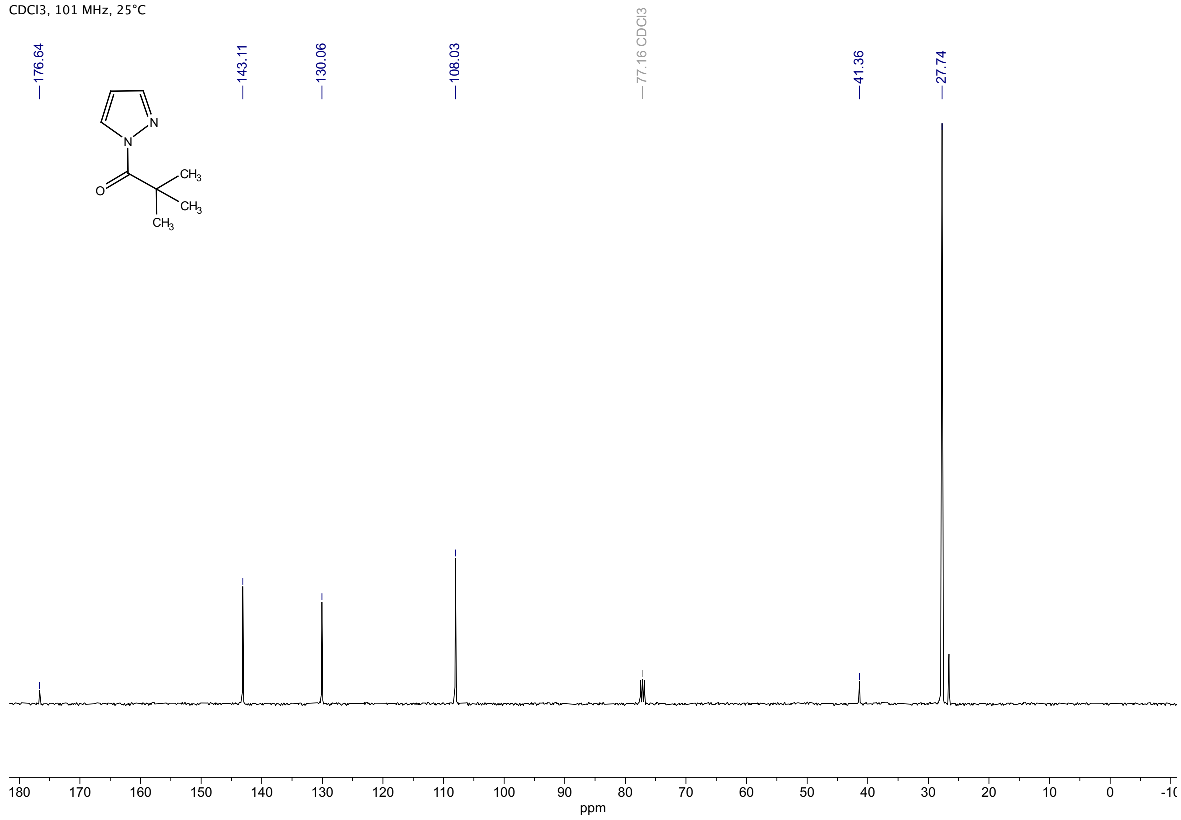

^{1}H NMR of 1-(tetrahydro-*2H*-pyran-2-yl)-*1H*-pyrazole (**1j**)

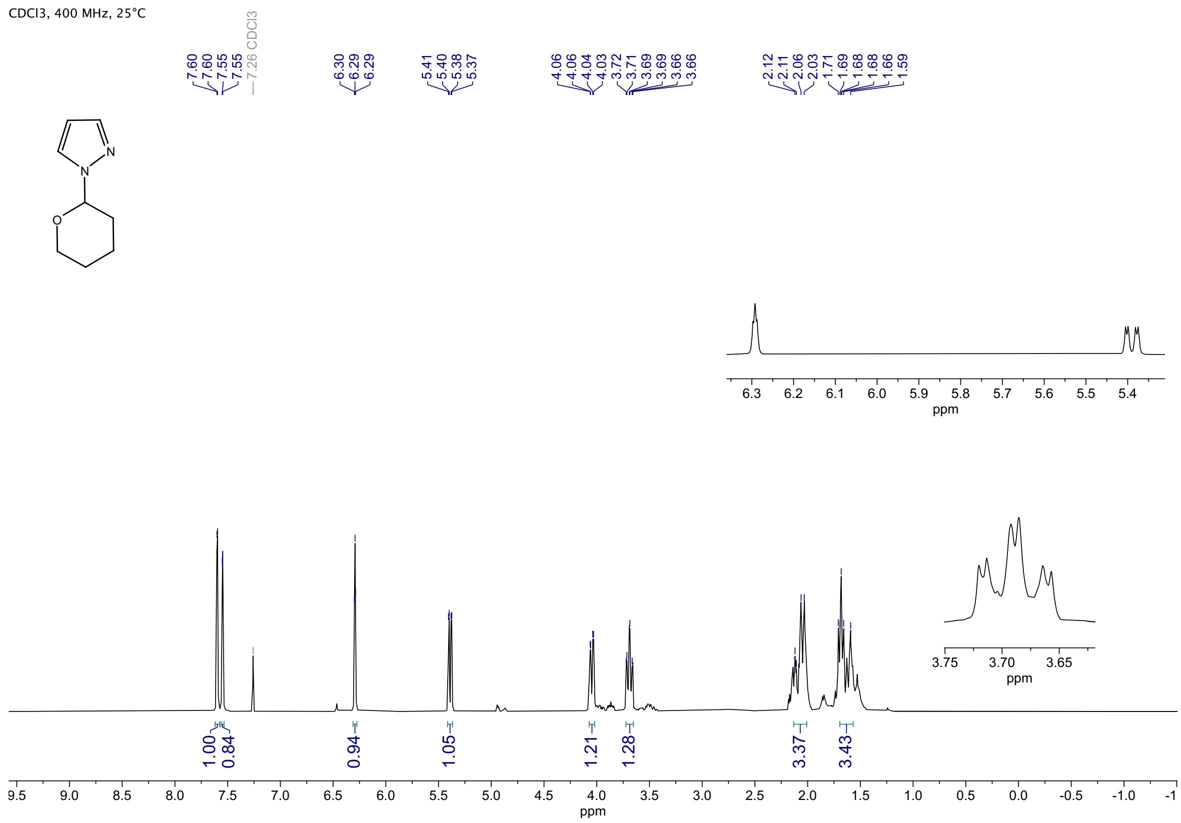

^{13}C NMR of 1-(tetrahydro-*2H*-pyran-2-yl)-*1H*-pyrazole (**1j**)

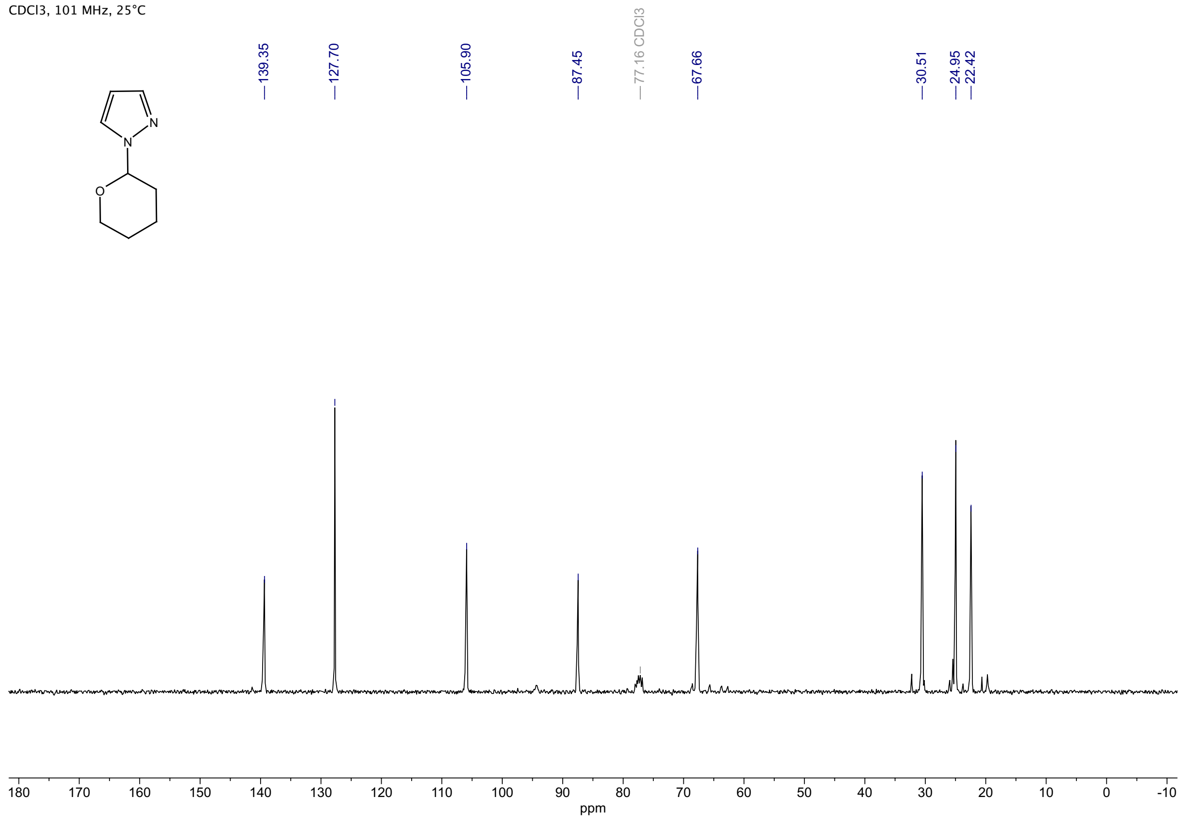

^{1}H NMR of 1-((2-(trimethylsilyl)ethoxy)methyl)-*1H*-pyrazole (**1k**)

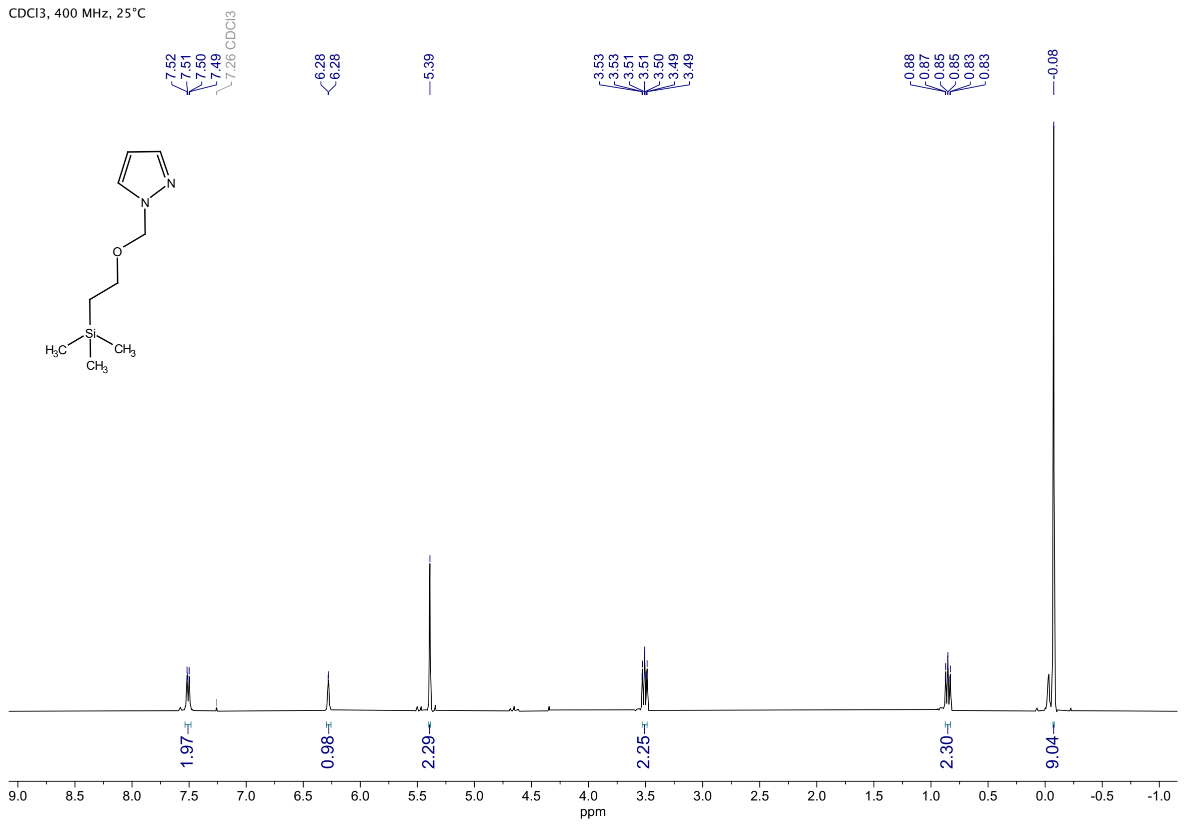

^{13}C NMR of 1-((2-(trimethylsilyl)ethoxy)methyl)-*1H*-pyrazole (**1k**)

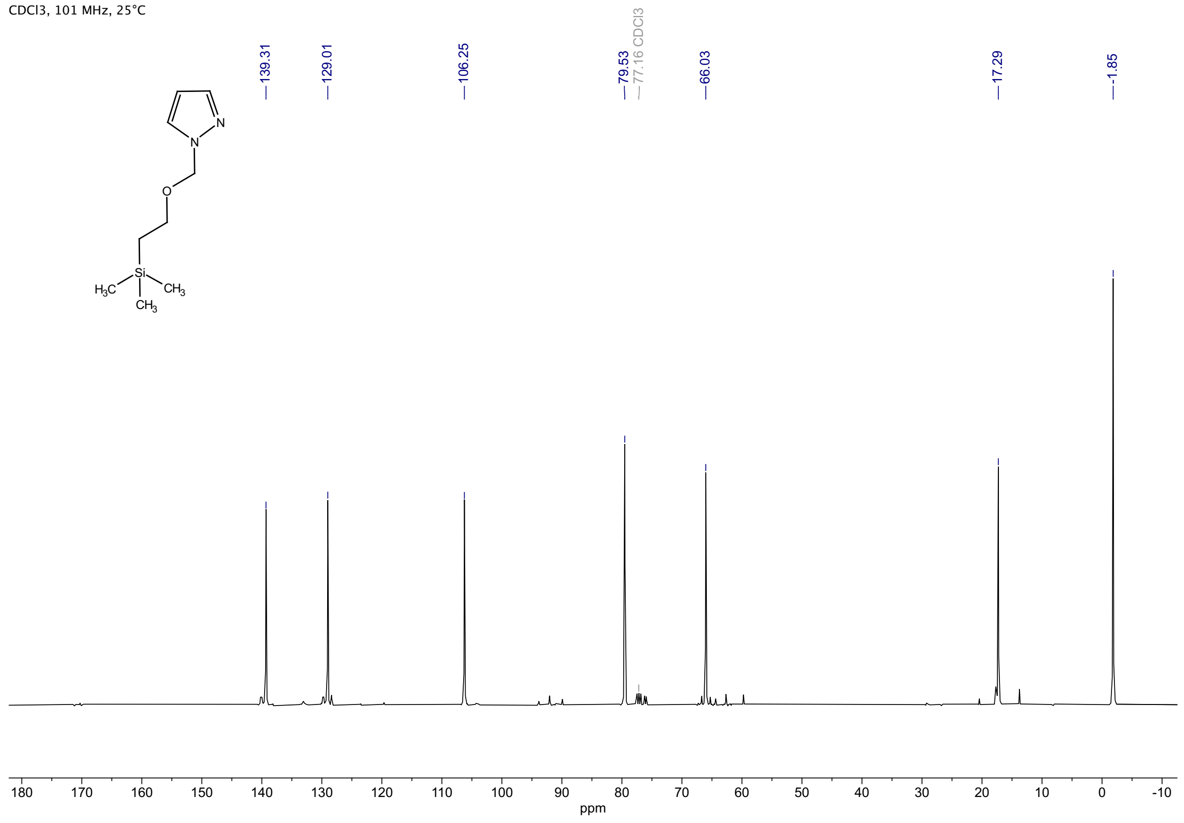

^{1}H NMR of 1-((perfluorophenyl)methyl)-1H-pyrazole (**1l**)

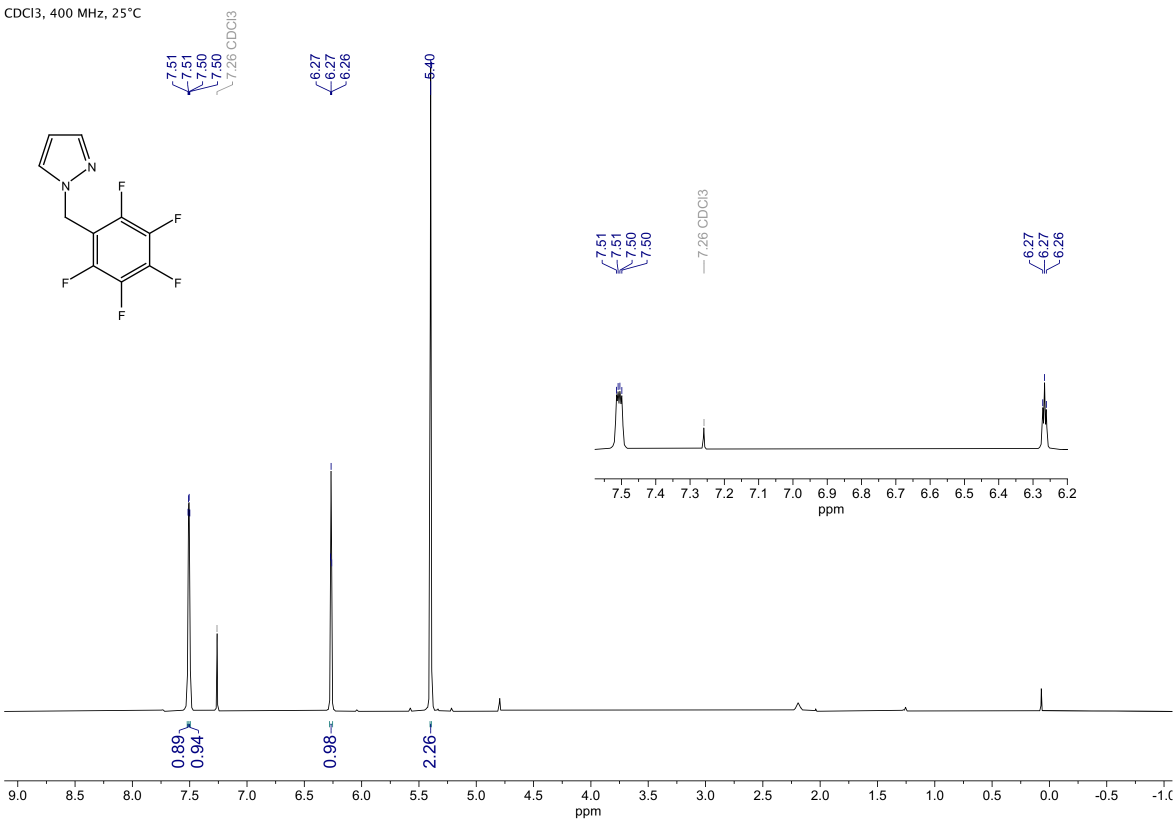

^{13}C NMR of 1-((perfluorophenyl)methyl)-1H-pyrazole (**1l**)

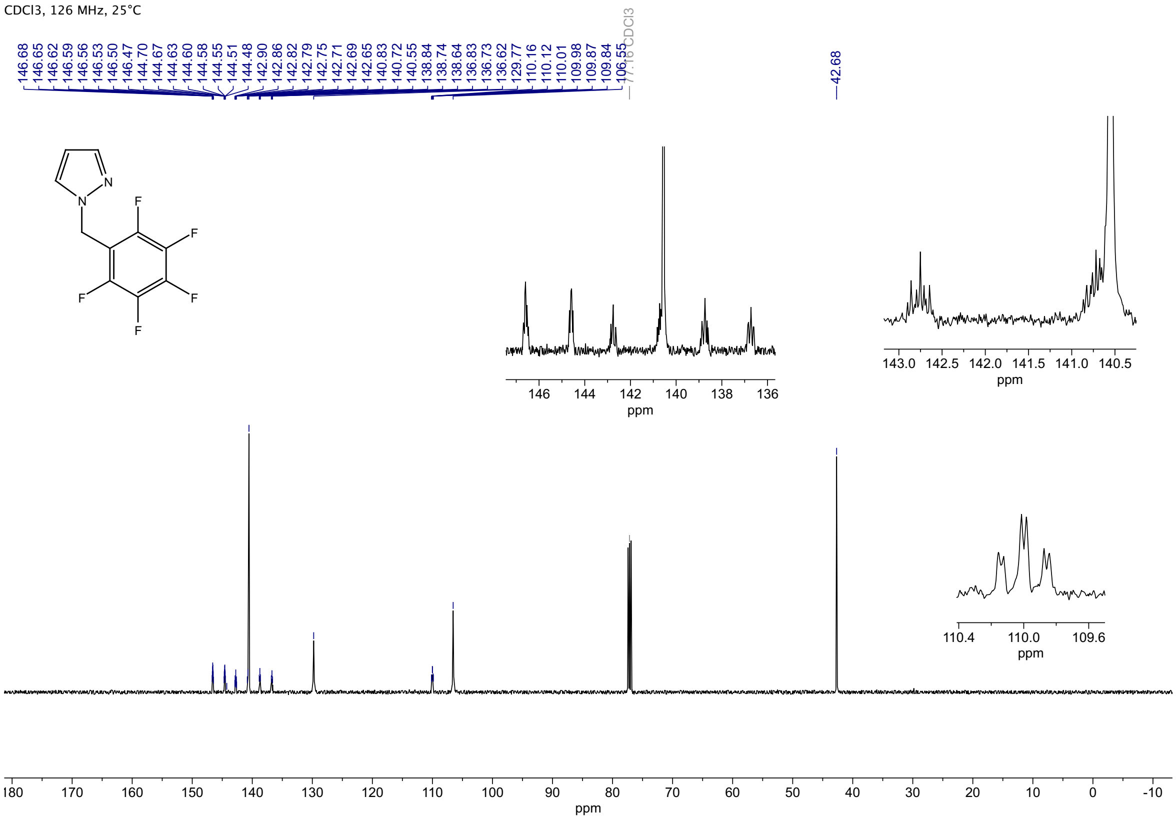

^{19}F NMR of 1-((perfluorophenyl)methyl)-1H-pyrazole (**1l**)

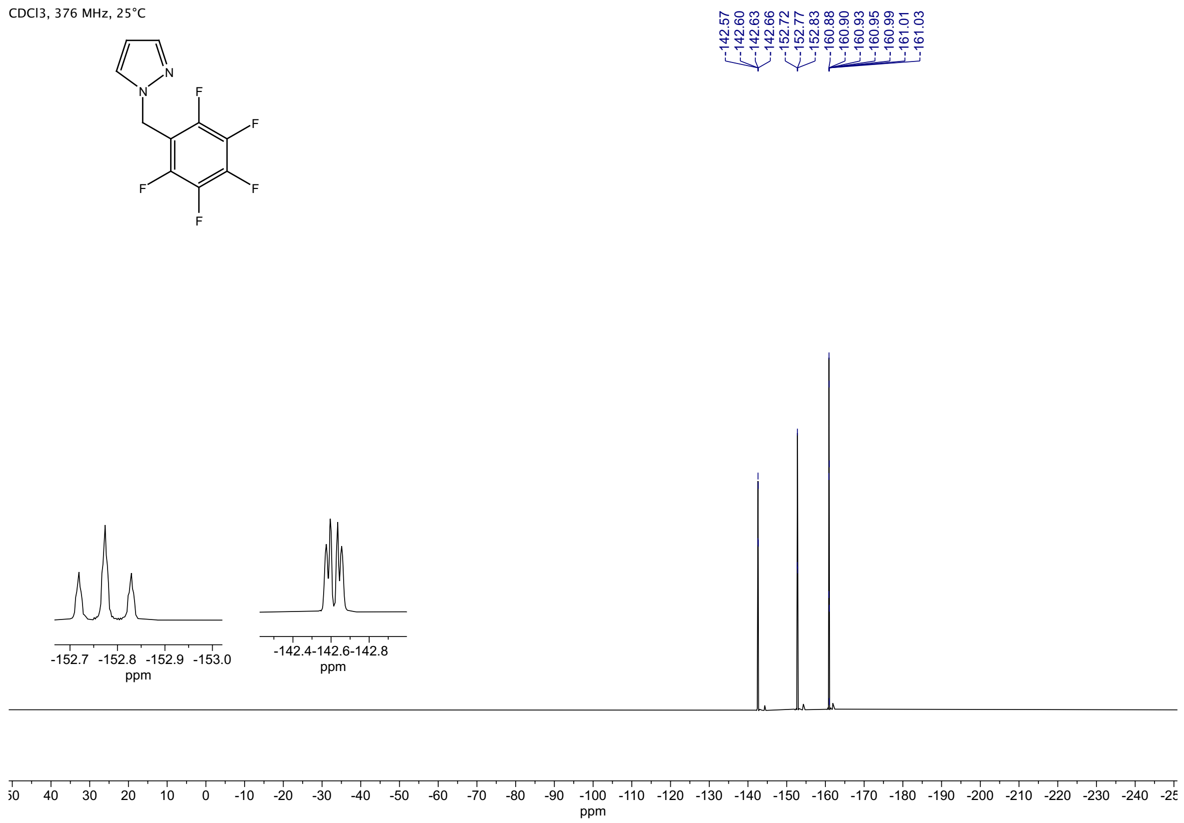

^{1}H NMR of 4-methoxy-1-((2-(trimethylsilyl)ethoxy)methyl)-*1H*-pyrazole (**3a-SM**)

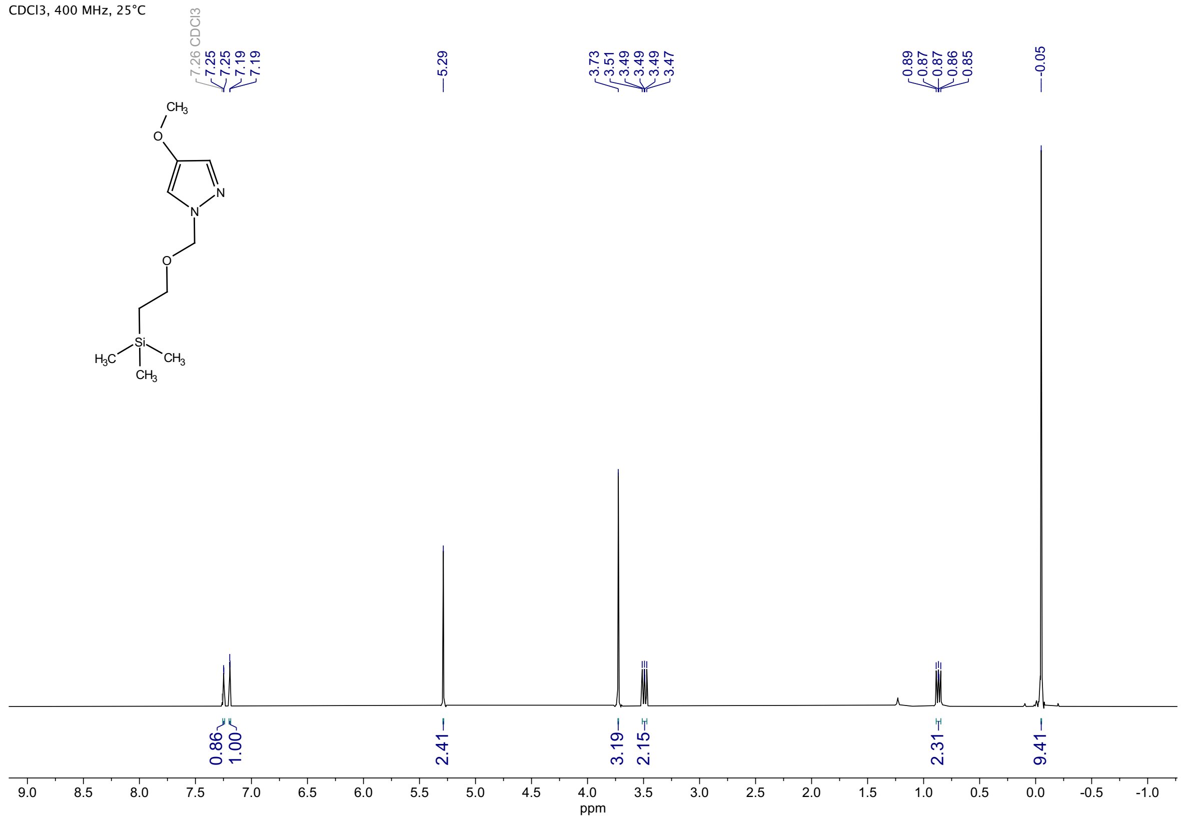

^{13}C NMR of 4-methoxy-1-((2-(trimethylsilyl)ethoxy)methyl)-*1H*-pyrazole (**3a-SM**)

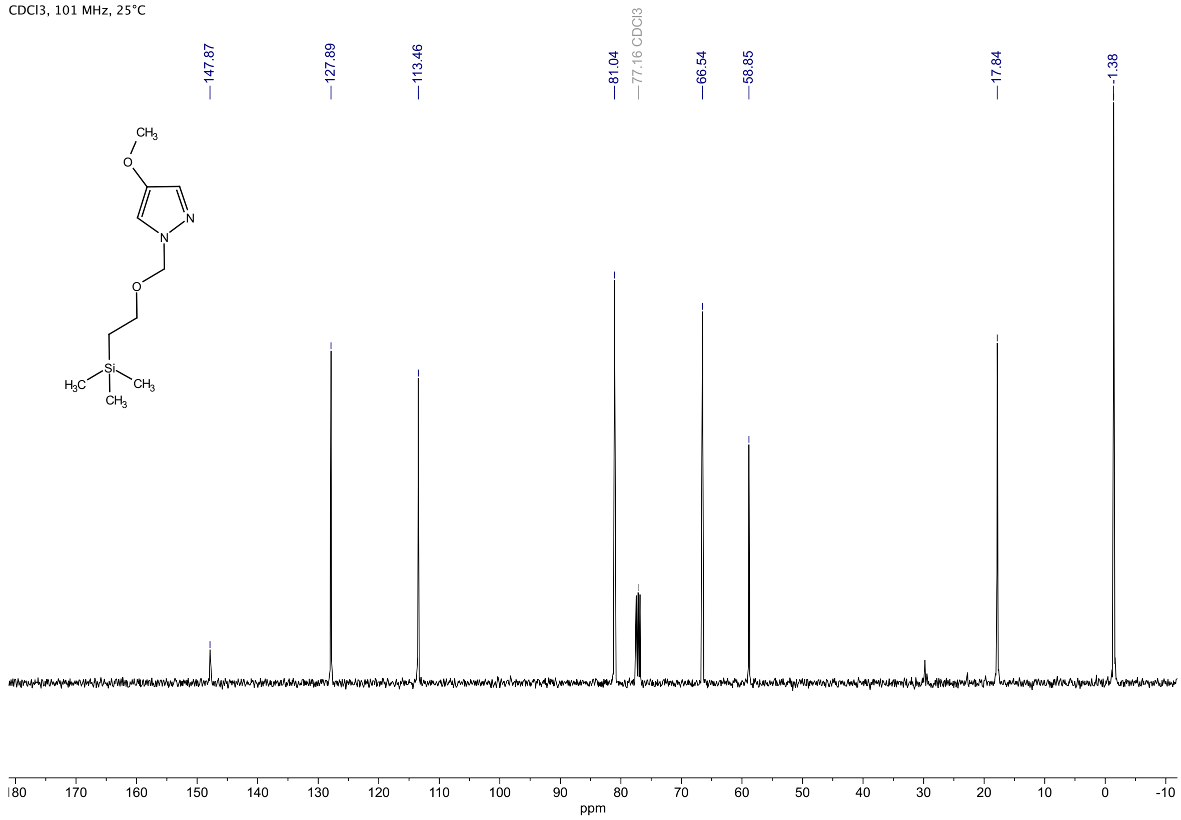

^{1}H NMR of 4-methyl-1-((2-(trimethylsilyl)ethoxy)methyl)-*1H*-pyrazole (**3b-SM**)

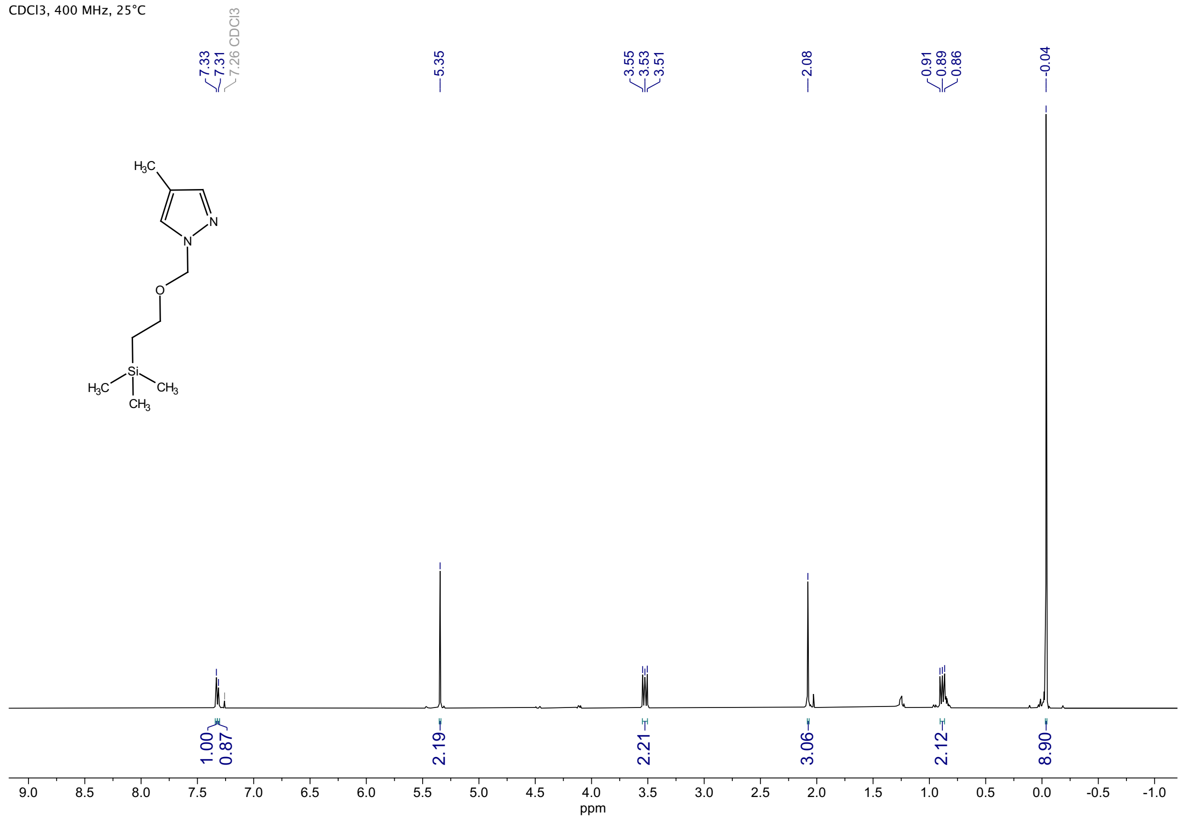

^{13}C NMR of 4-methyl-1-((2-(trimethylsilyl)ethoxy)methyl)-*1H*-pyrazole (**3b-SM**)

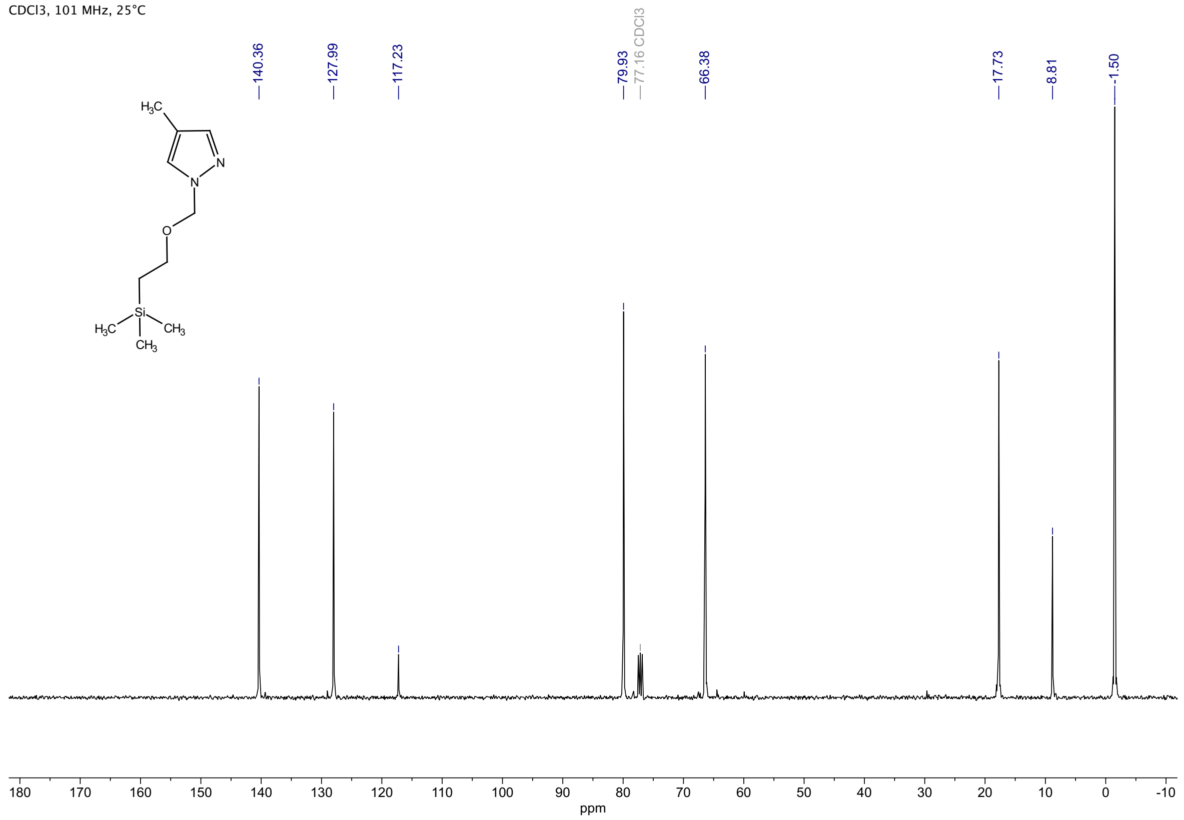

^{1}H NMR of 1-methyl-2-(*1H*-pyrazol-3-yl)pyridin-1-ium iodide

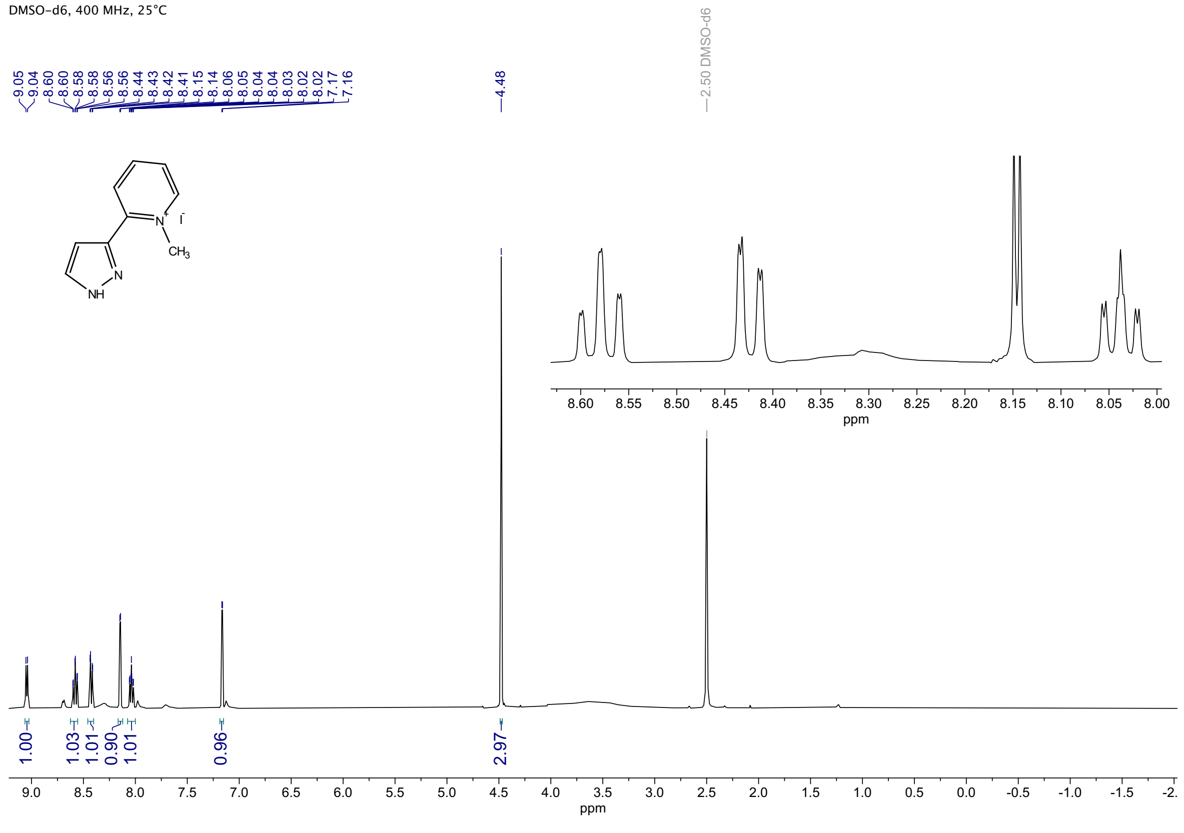

^{13}C NMR of 1-methyl-2-(*1H*-pyrazol-3-yl)pyridin-1-ium iodide

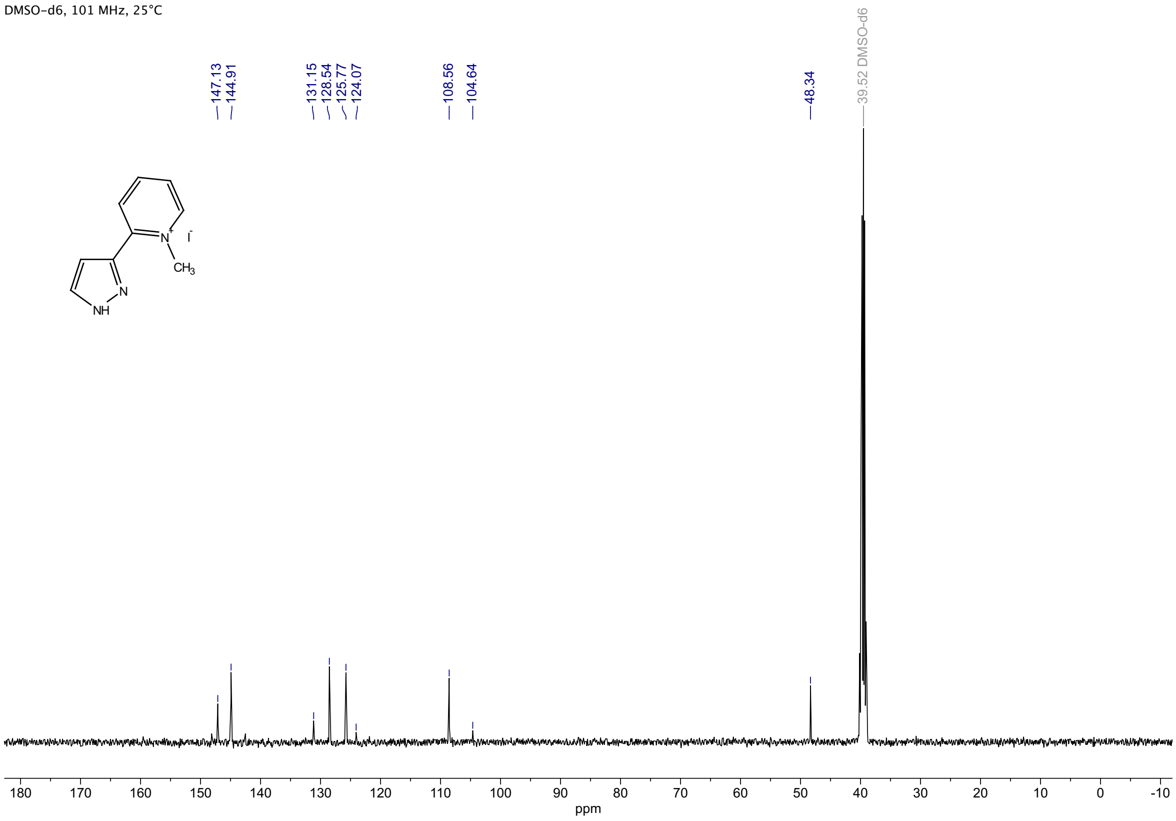

^{1}H NMR of 1-methyl-2-(*1H*-pyrazol-3-yl)-1,2,3,6-tetrahydropyridine

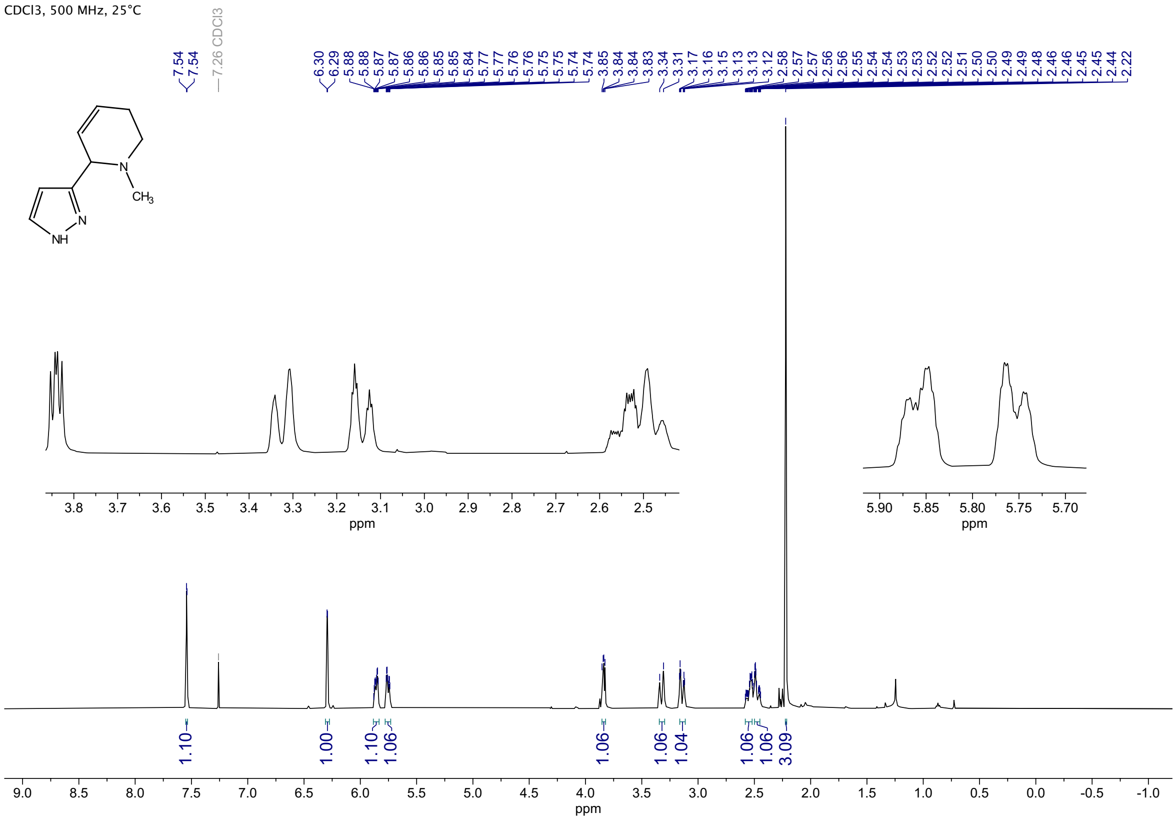

^{13}C NMR of 1-methyl-2-(*1H*-pyrazol-3-yl)-1,2,3,6-tetrahydropyridine

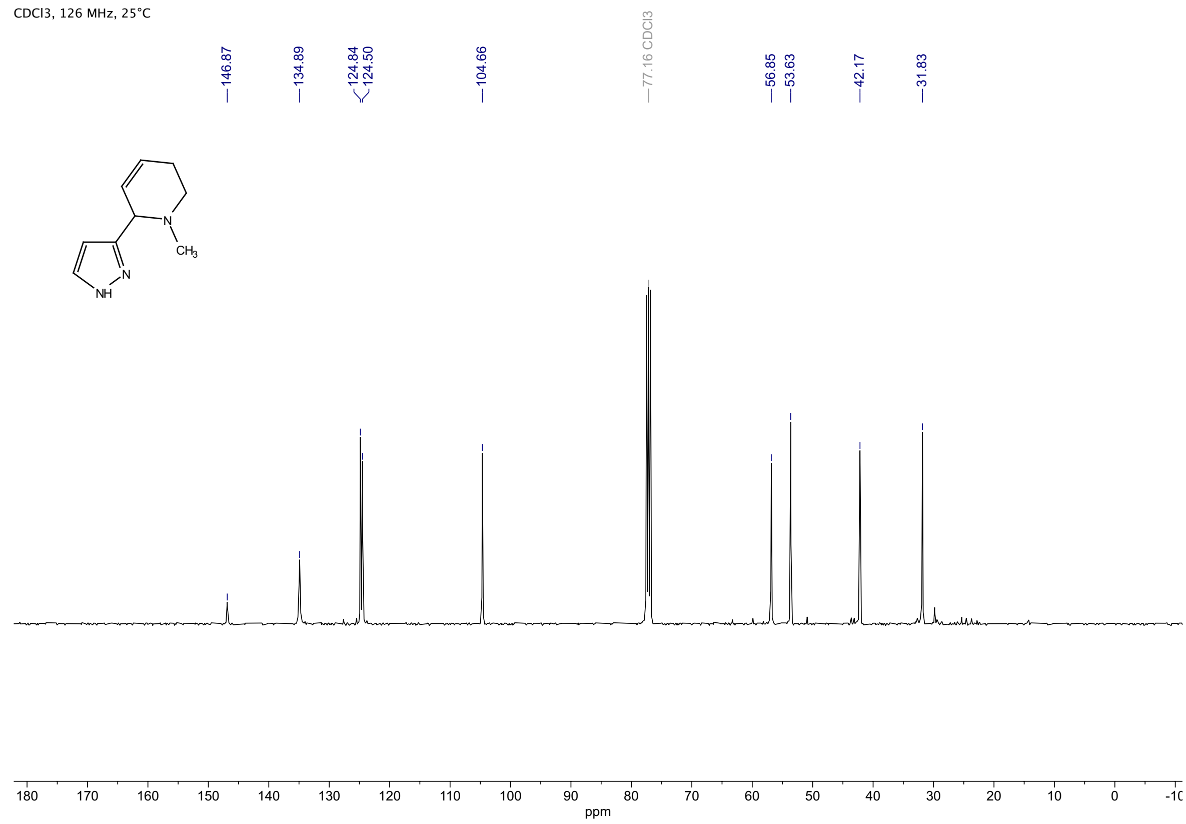

DEPT-135 of 1-methyl-2-(*1H*-pyrazol-3-yl)-1,2,3,6-tetrahydropyridine

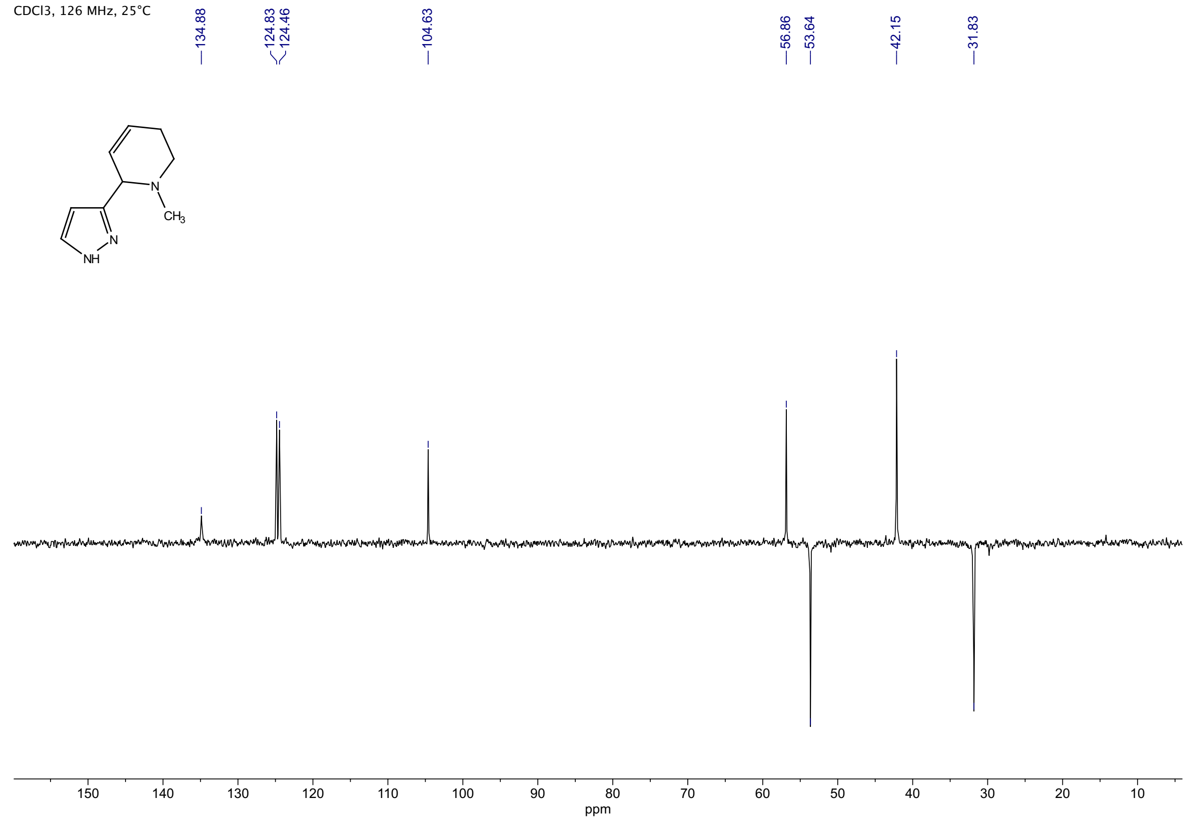

HSQC of 1-methyl-2-(*1H*-pyrazol-3-yl)-1,2,3,6-tetrahydropyridine

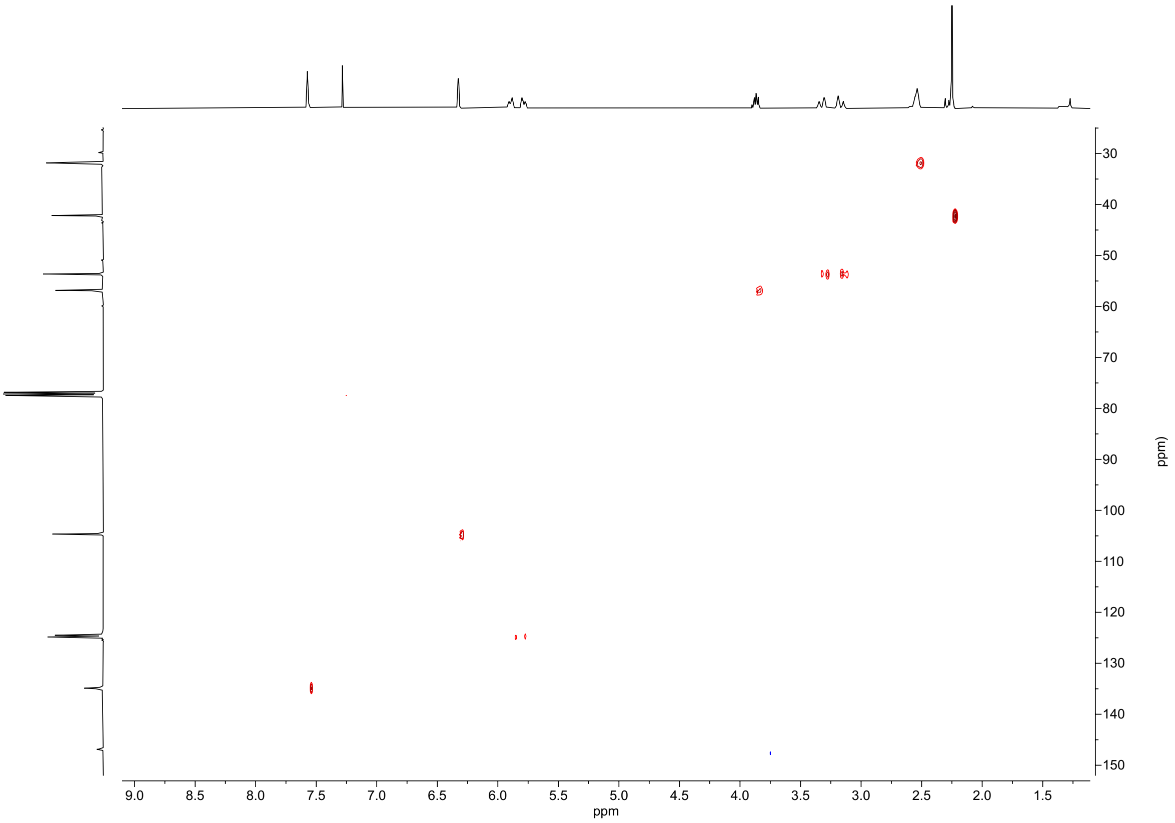

HMBC of 1-methyl-2-(*1H*-pyrazol-3-yl)-1,2,3,6-tetrahydropyridine

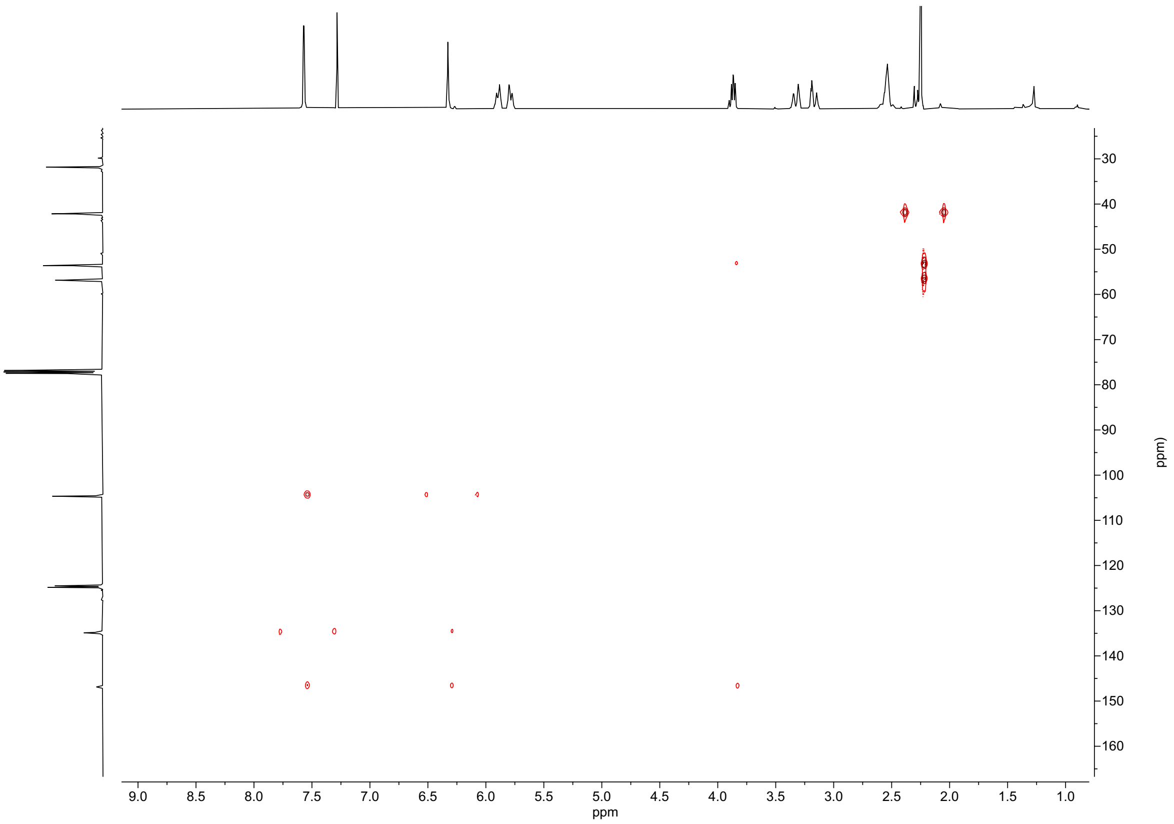

^{1}H NMR of 1-methyl-2-(*1H*-pyrazol-3-yl)piperidine (**5i-SM**)

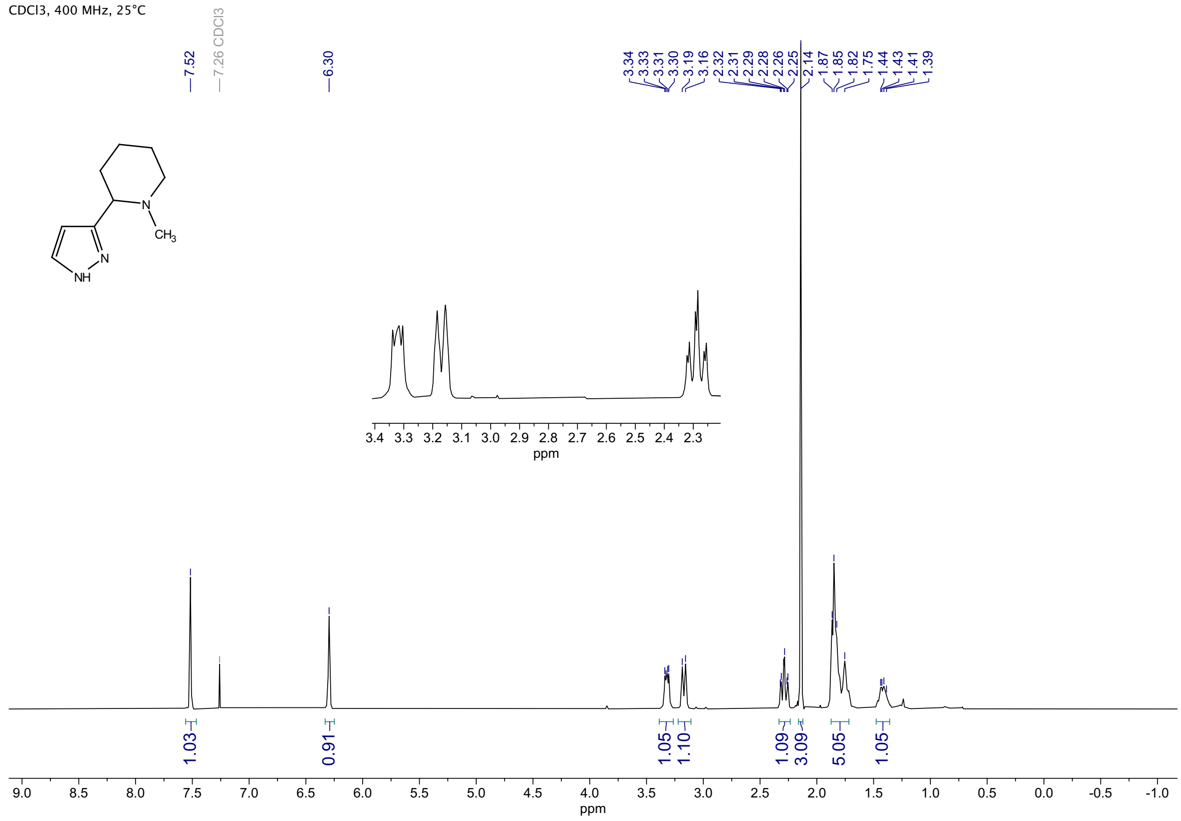

^{13}C NMR of 1-methyl-2-(*1H*-pyrazol-3-yl)piperidine (**5i-SM**)

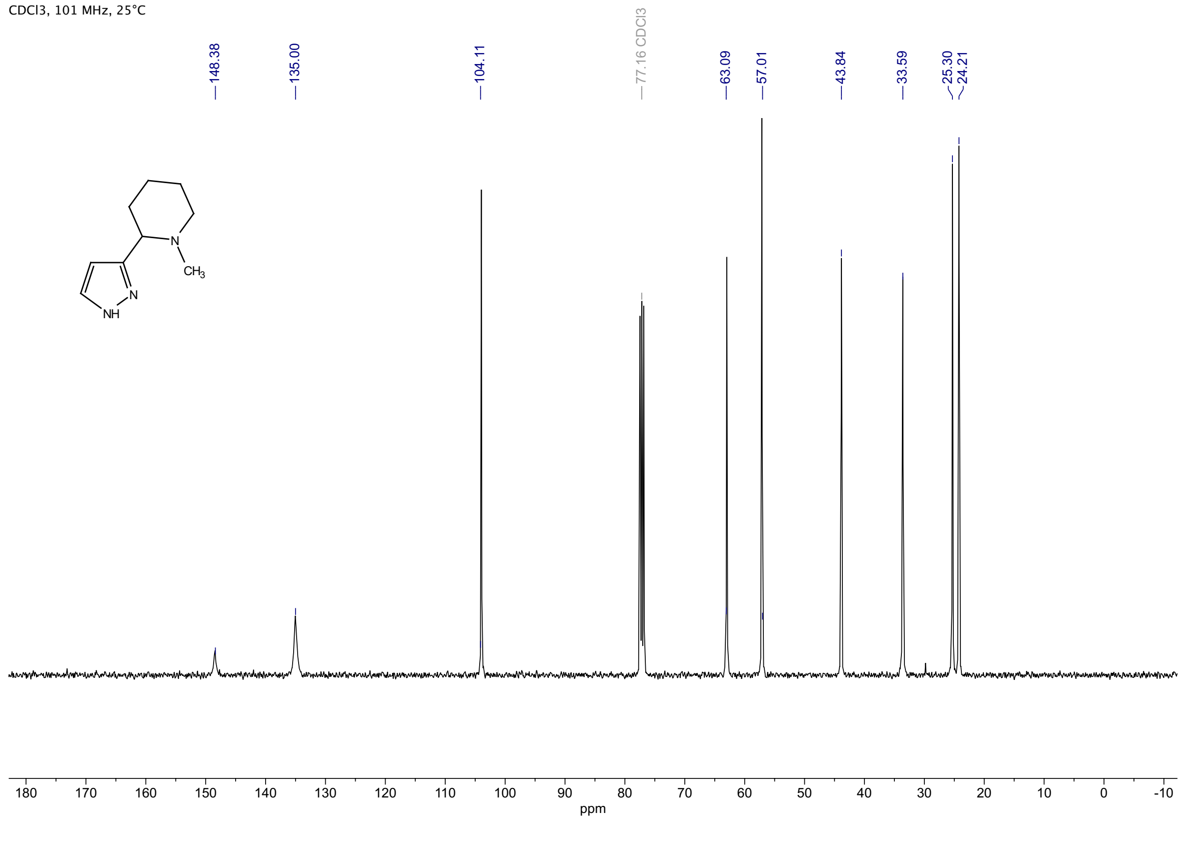

^{1}H NMR of 3-bromo-4-methoxy-*1H*-pyrazole (**6b-SM**)

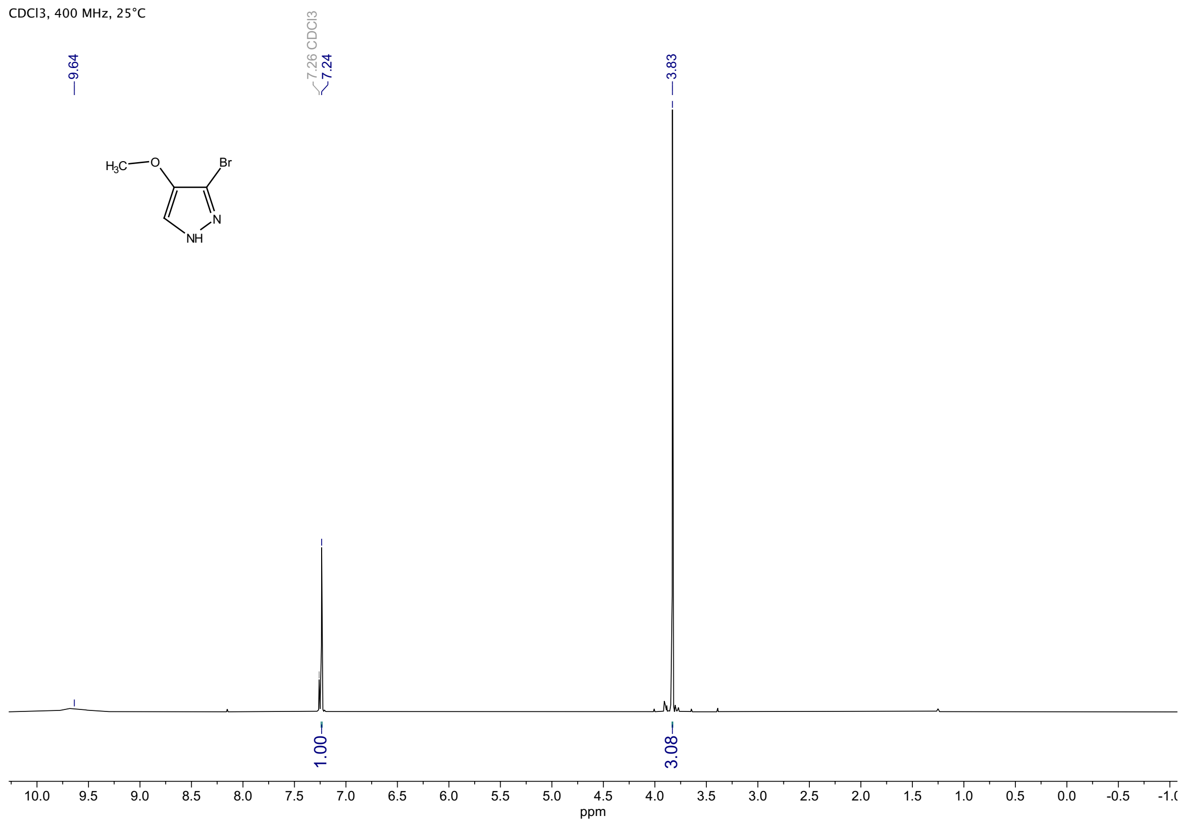

^{13}C NMR of 3-bromo-4-methoxy-*1H*-pyrazole (**6b-SM**)

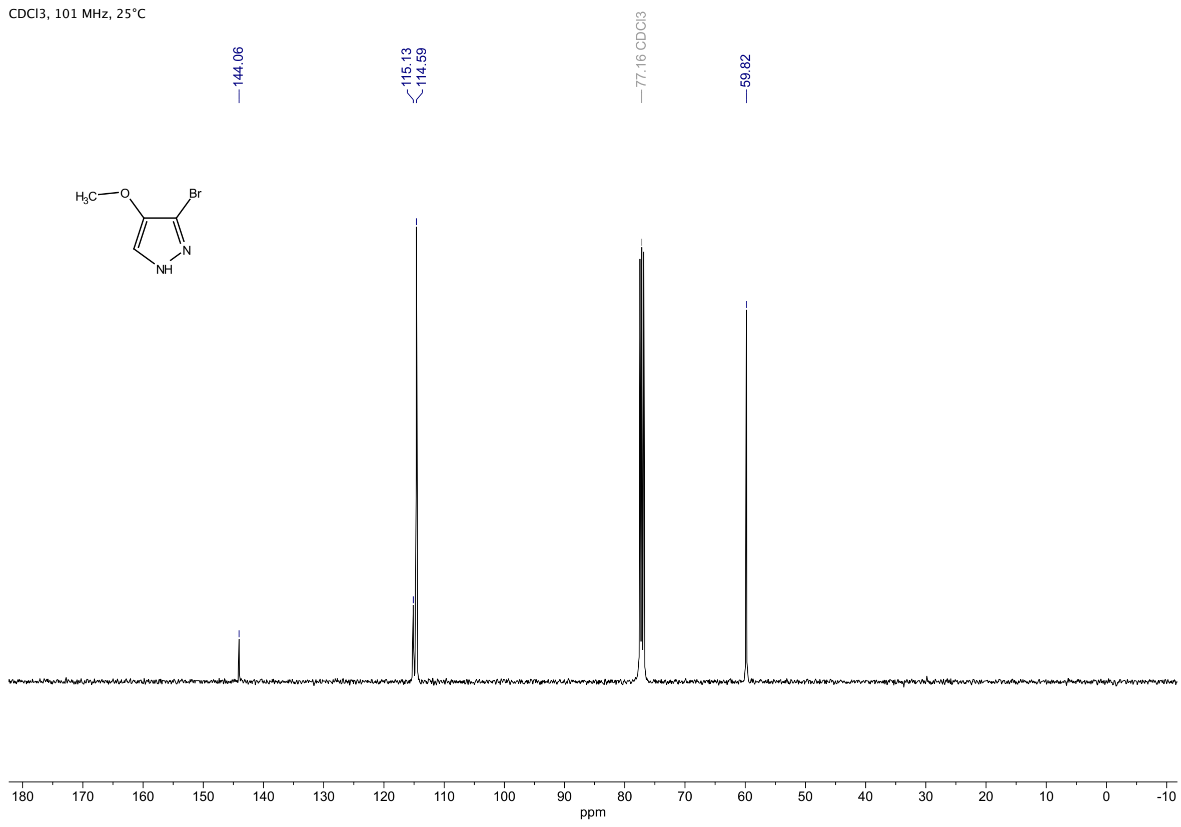

^{1}H NMR of 3-iodo-4-methoxy-*1H*-pyrazole (**6c-SM**)

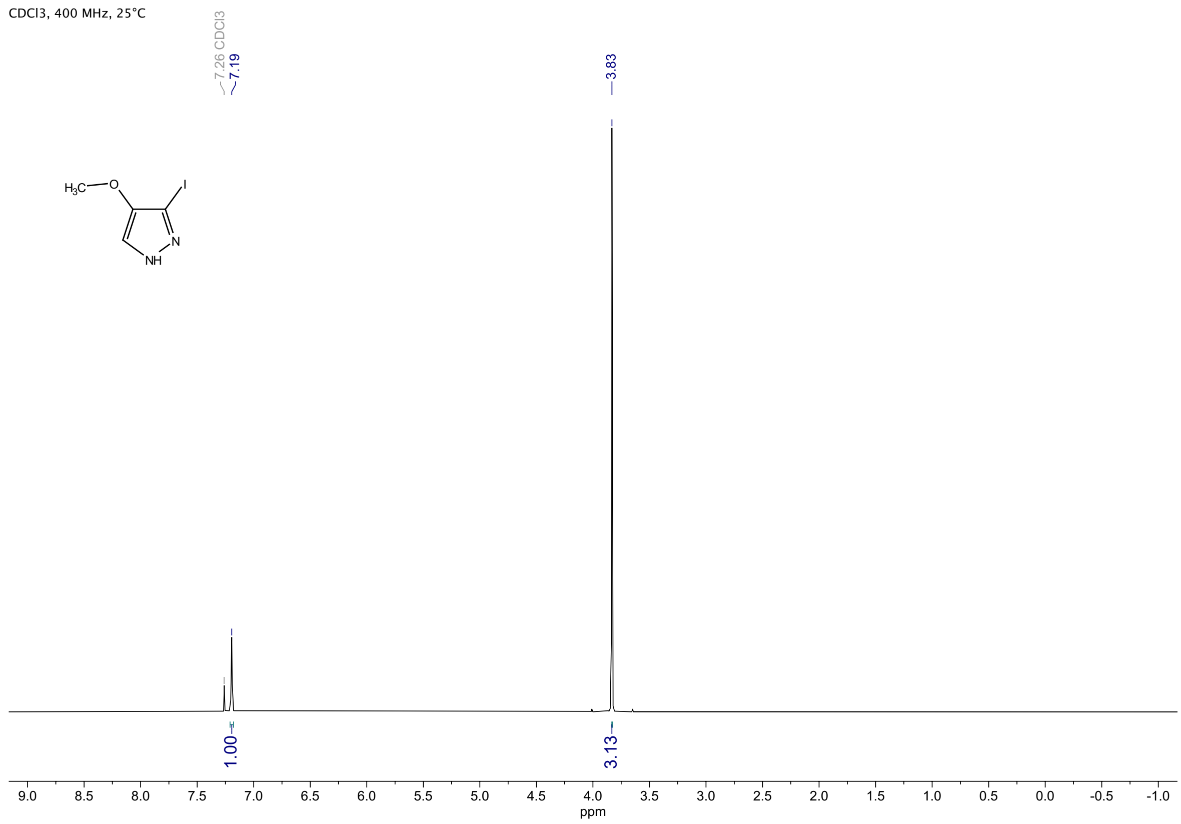

^{13}C NMR of 3-iodo-4-methoxy-*1H*-pyrazole (**6c-SM**)

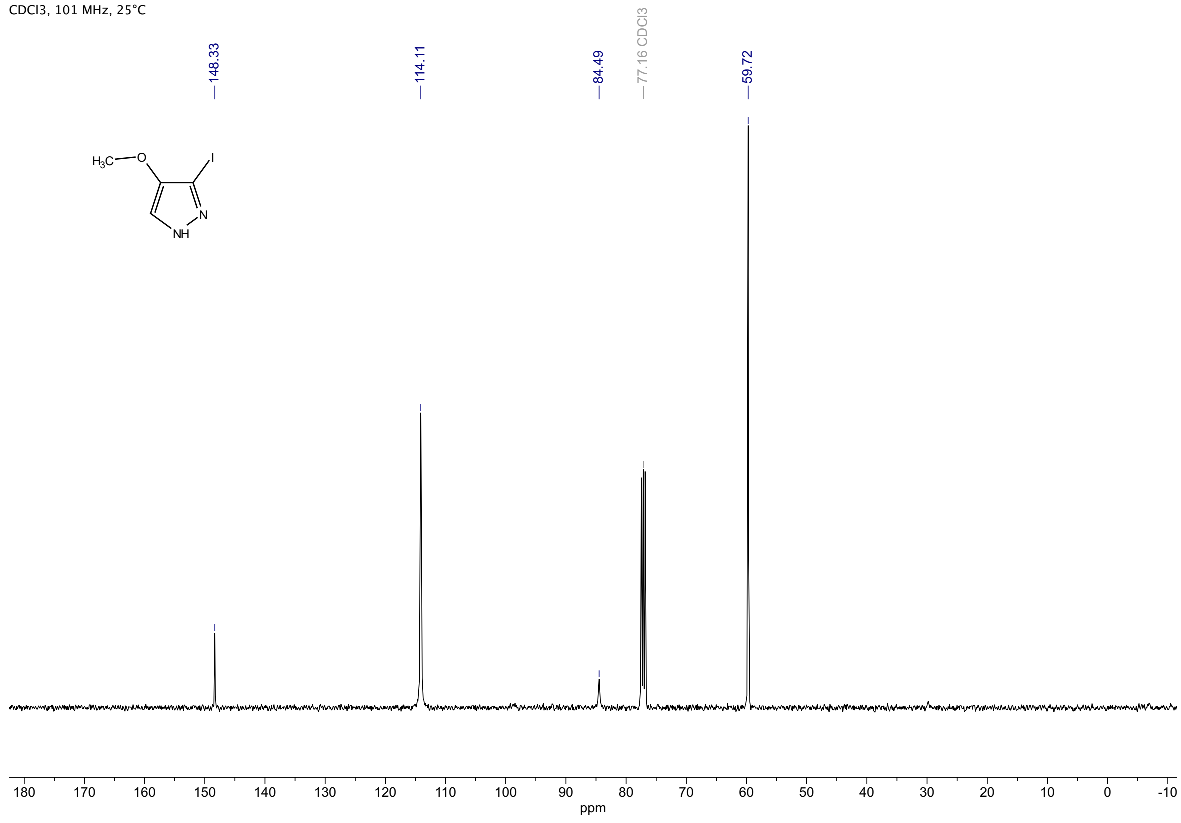

^{1}H NMR of 4-bromo-5-isopropyl-*1H*-pyrazole (**6h-SM**)

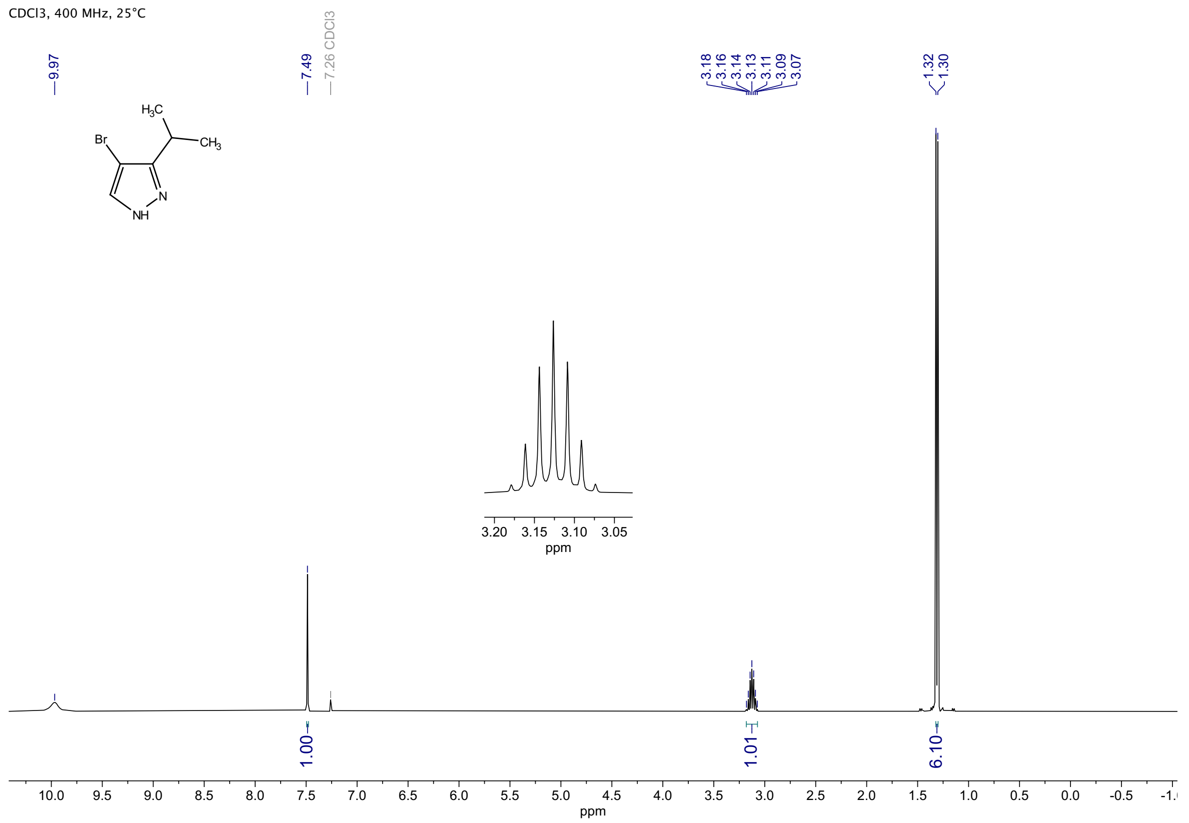

^{13}C NMR of 4-bromo-5-isopropyl-*1H*-pyrazole (**6h-SM**)

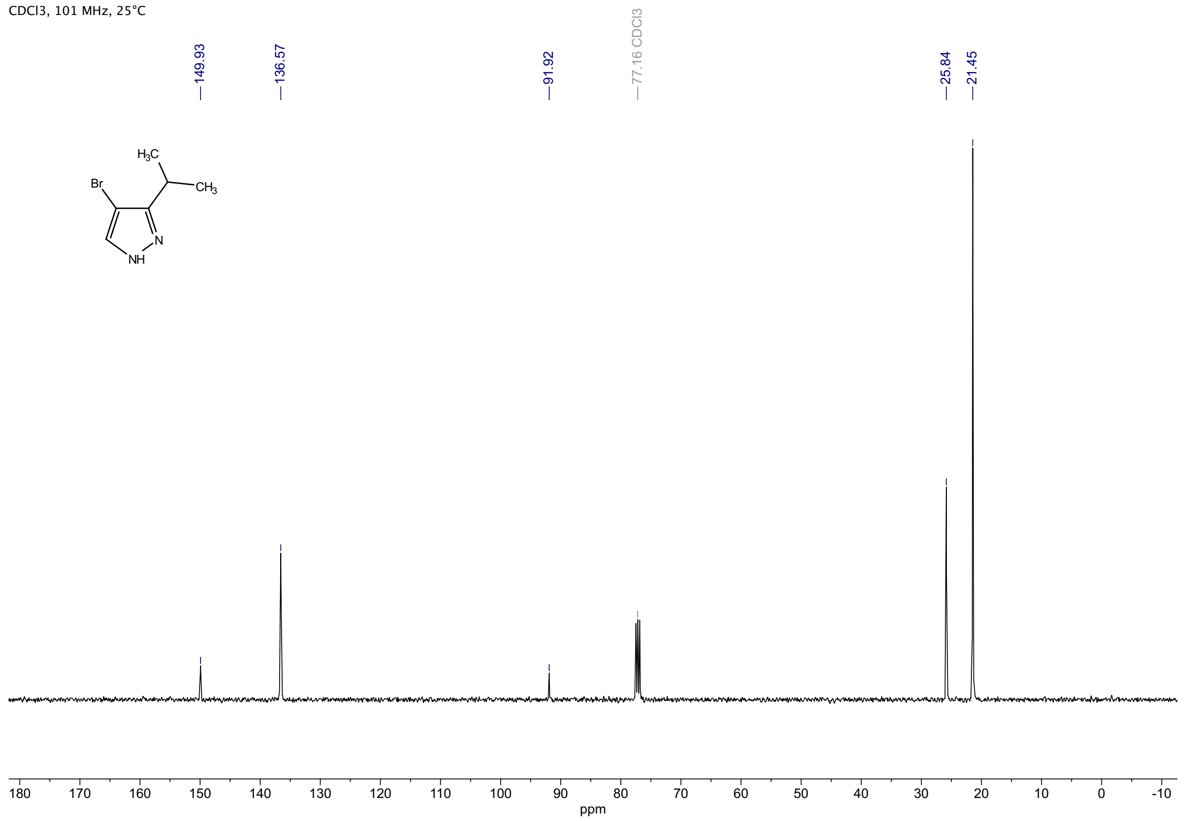

^{1}H NMR of 4-iodo-5-isopropyl-*1H*-pyrazole (**6i-SM**)

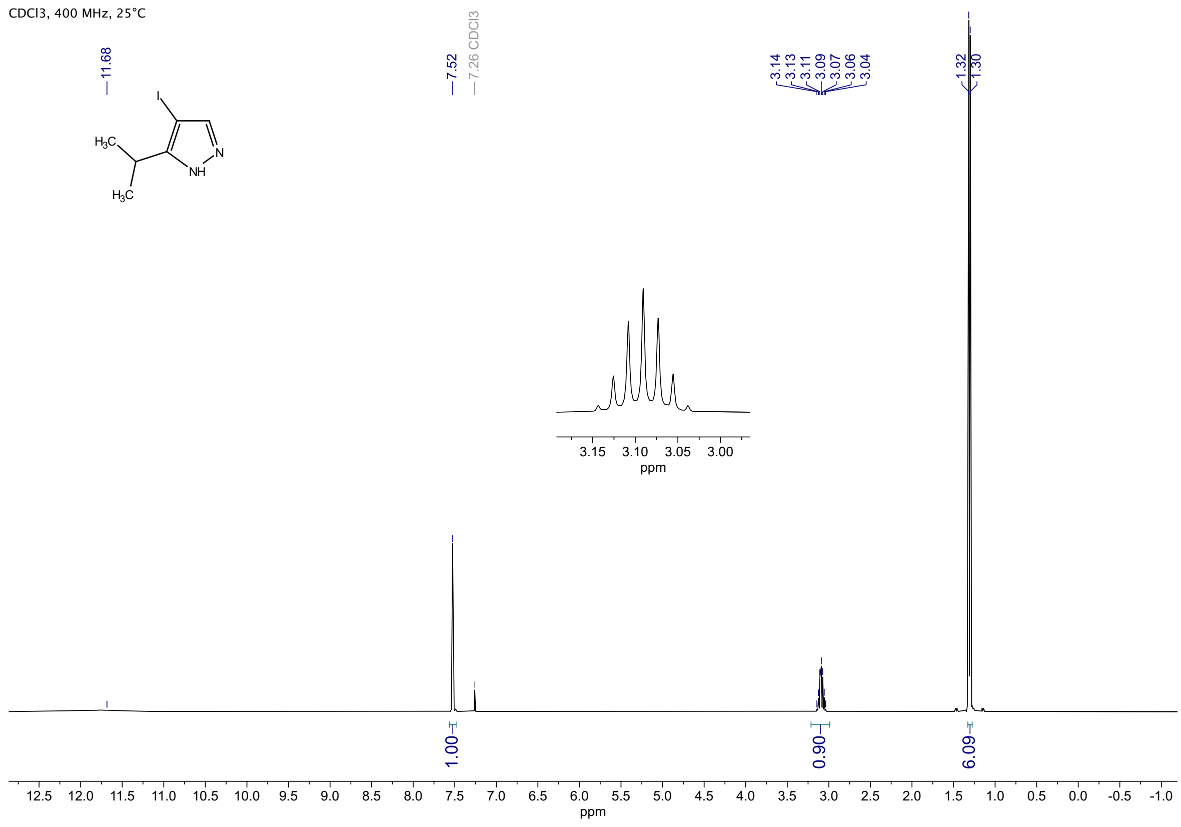

^{13}C NMR of 4-iodo-5-isopropyl-*1H*-pyrazole (**6i-SM**)

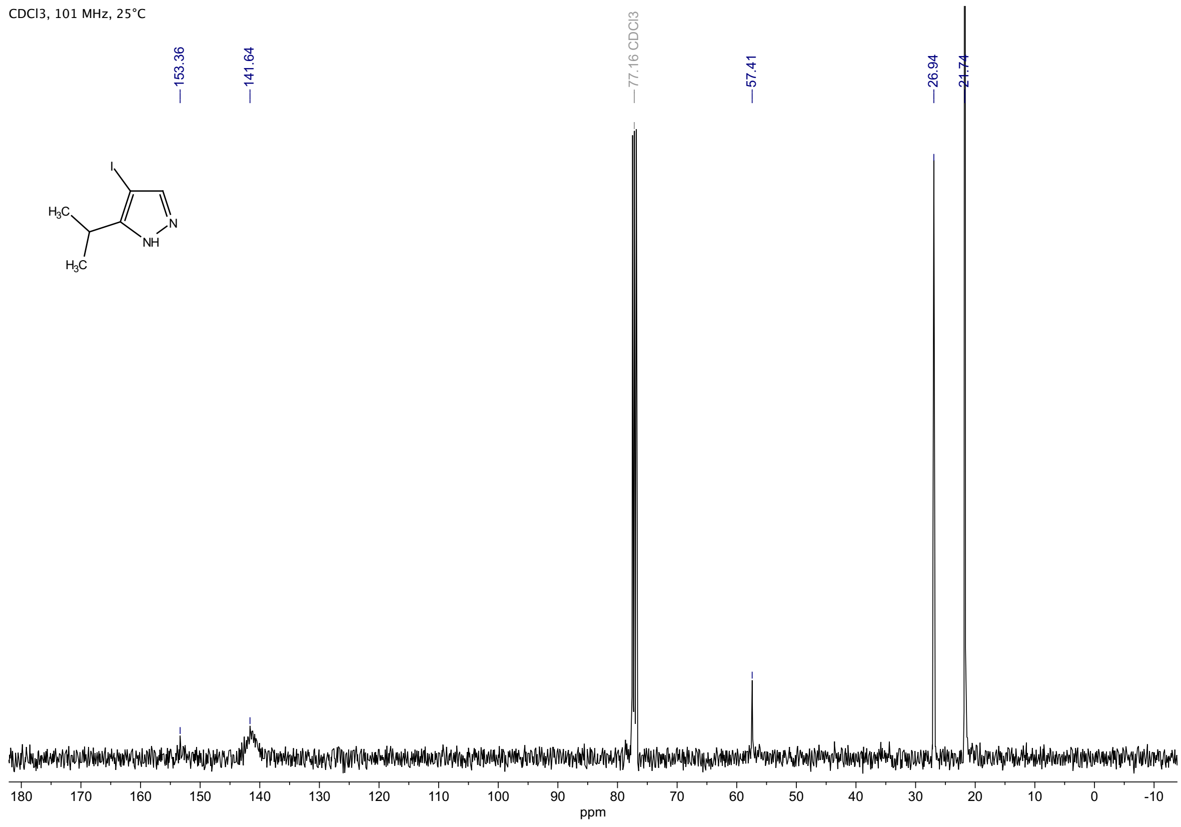

^{1}H NMR of 1-(4-bromo-*1H*-pyrazol-3-yl)ethan-1-one (**6j-SM**)

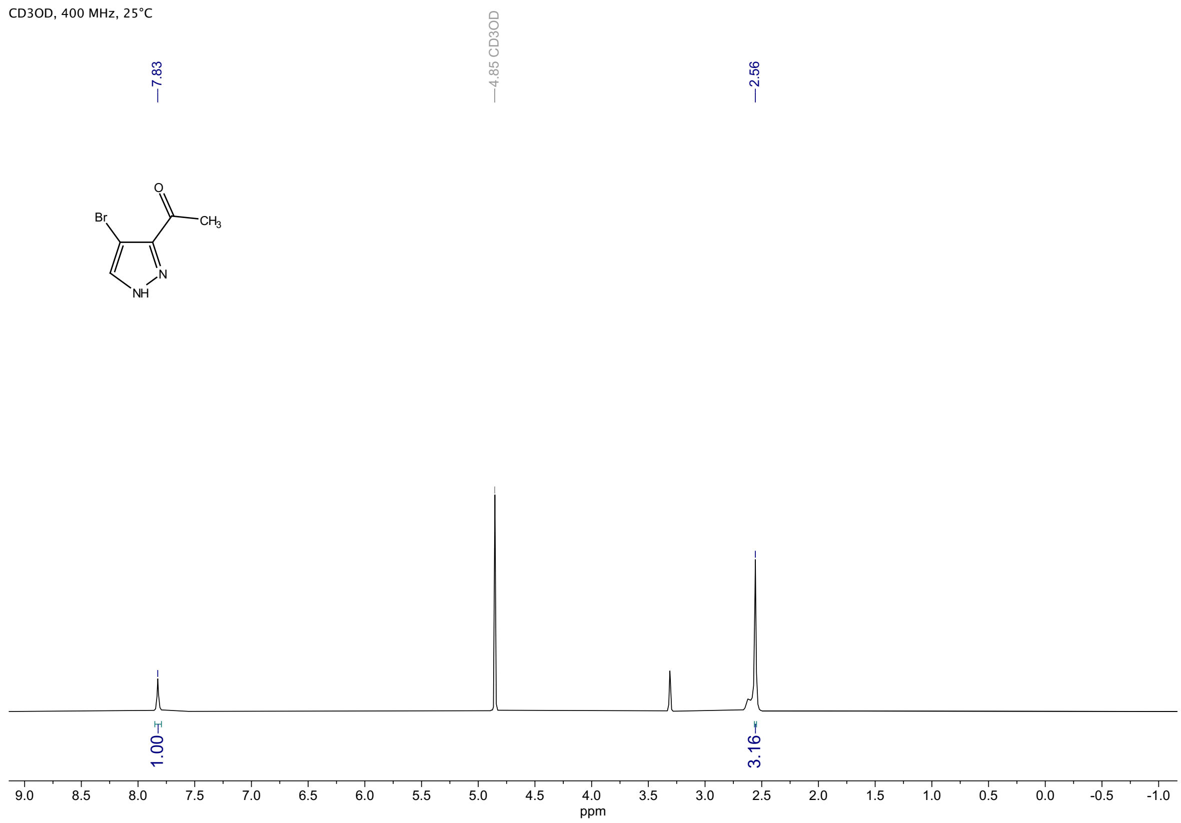

^{13}C NMR of 1-(4-bromo-*1H*-pyrazol-3-yl)ethan-1-one (**6j-SM**)

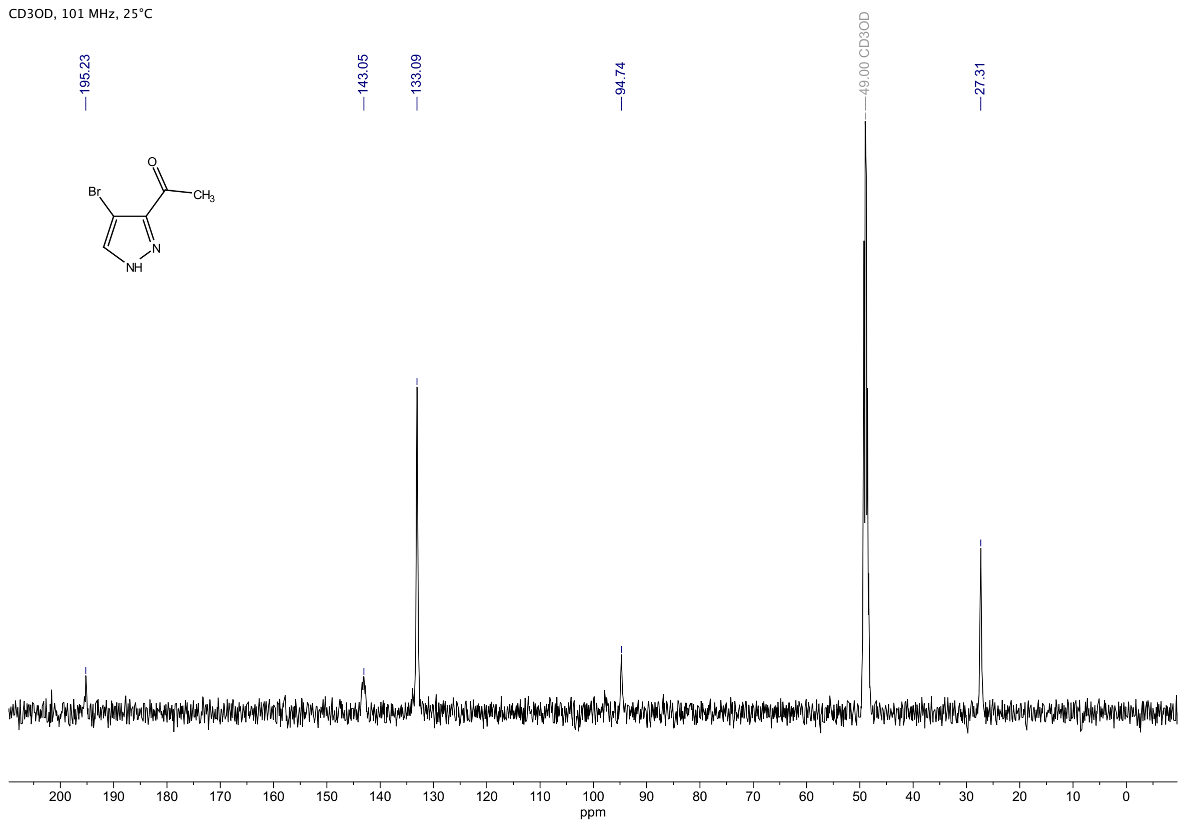

^{1}H NMR of 1-(4-iodo-*1H*-pyrazol-3-yl)ethan-1-one (**6l-SM**)

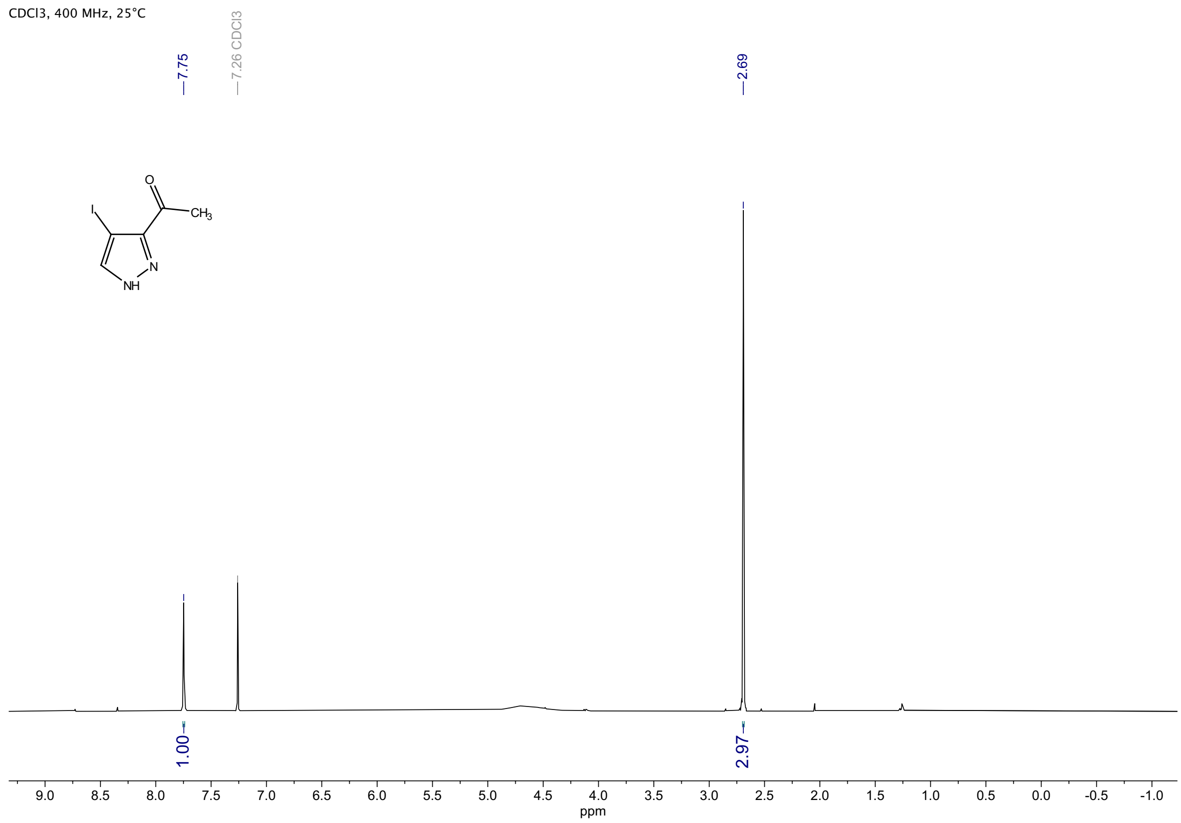

^{13}C NMR of 1-(4-iodo-*1H*-pyrazol-3-yl)ethan-1-one (**6l-SM**)

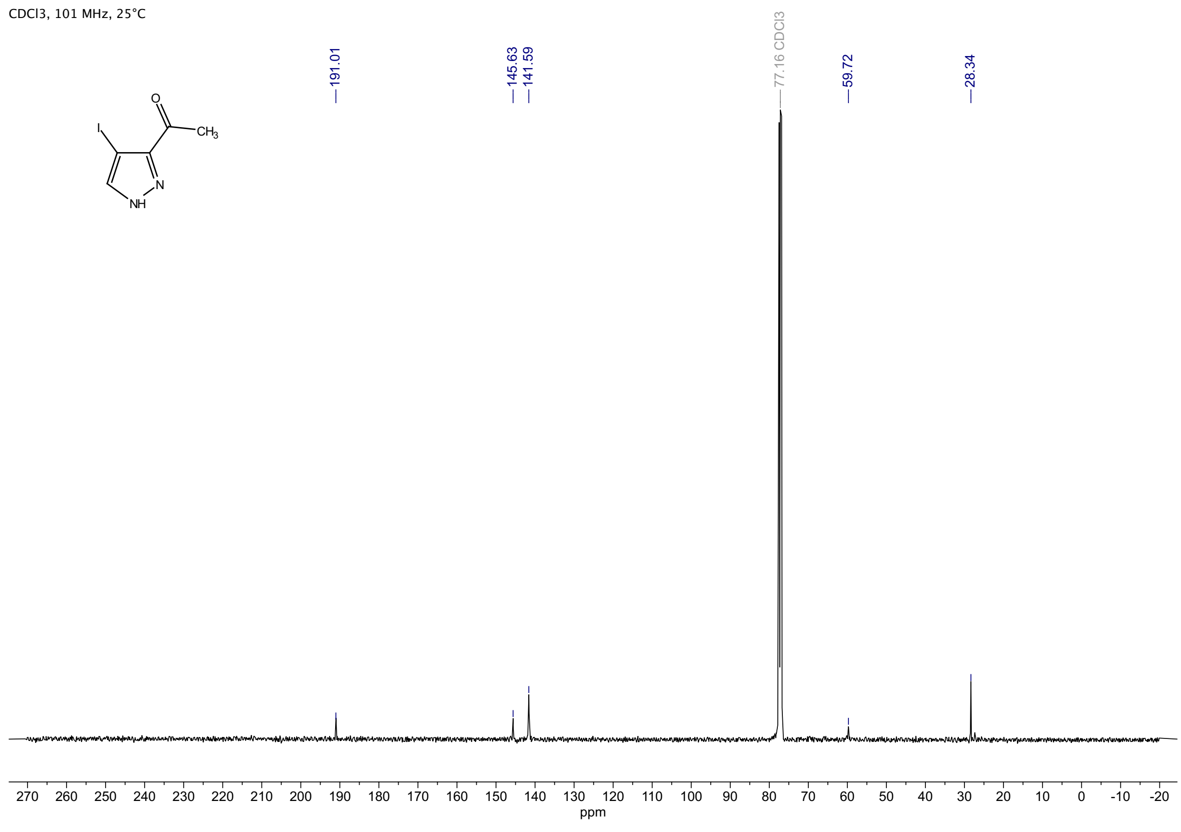

^{1}H NMR of Methyl 4-iodo-*1H*-pyrazole-3-carboxylate (**6m-SM**)

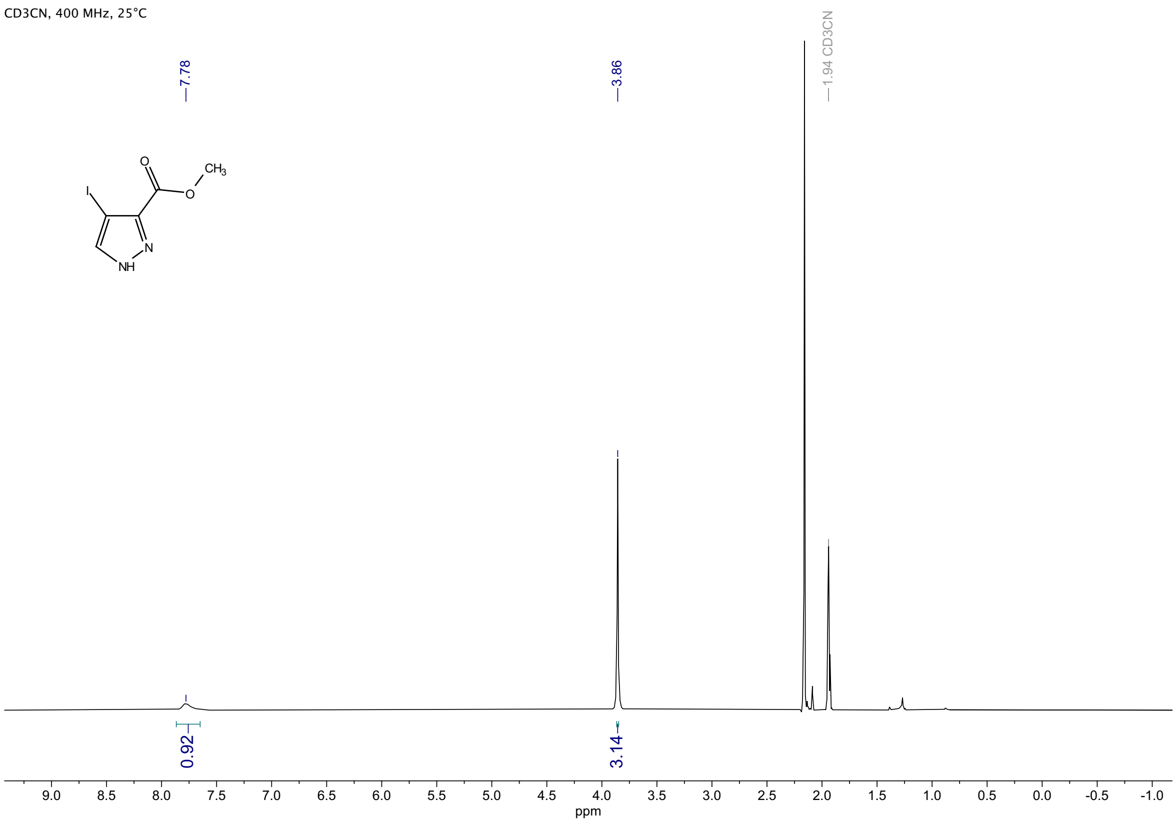

^{1}H NMR of *N*-(1,3,4-thiadiazol-2-yl)acetamide (**8-SM**)

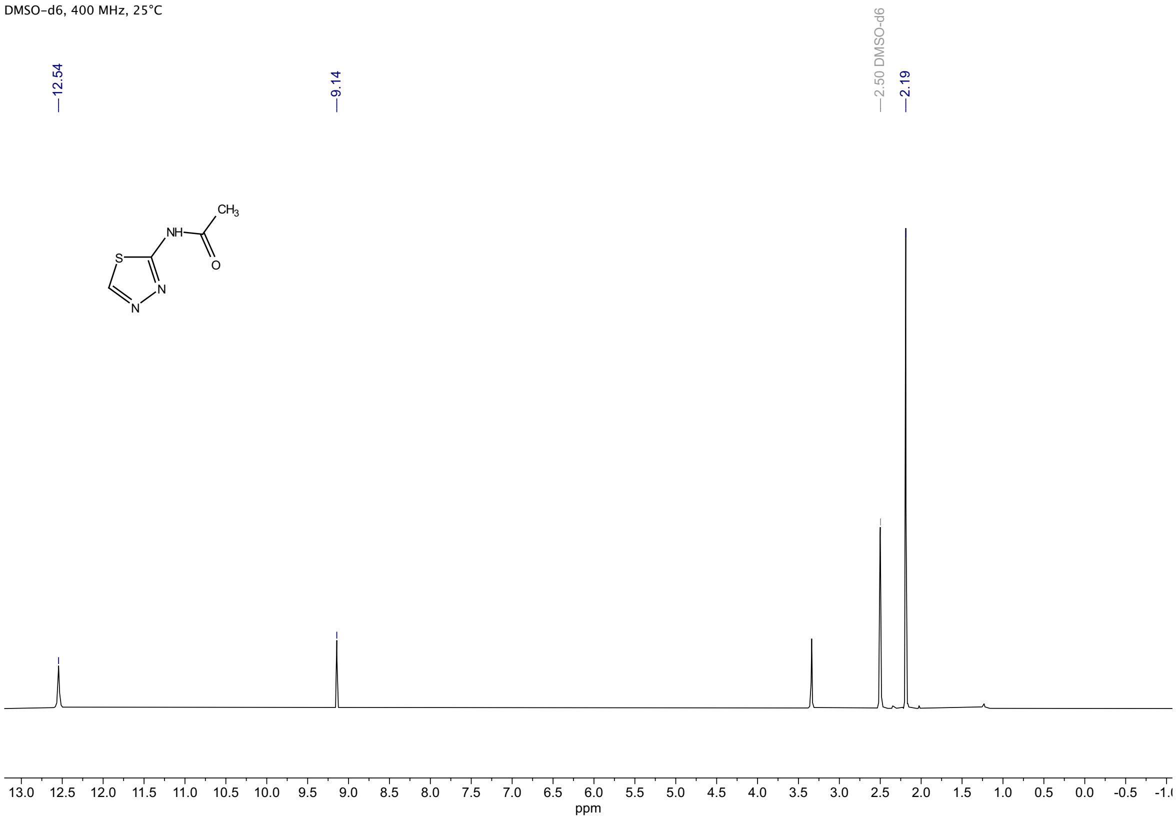

^{13}C NMR of *N*-(1,3,4-thiadiazol-2-yl)acetamide (**8-SM**)

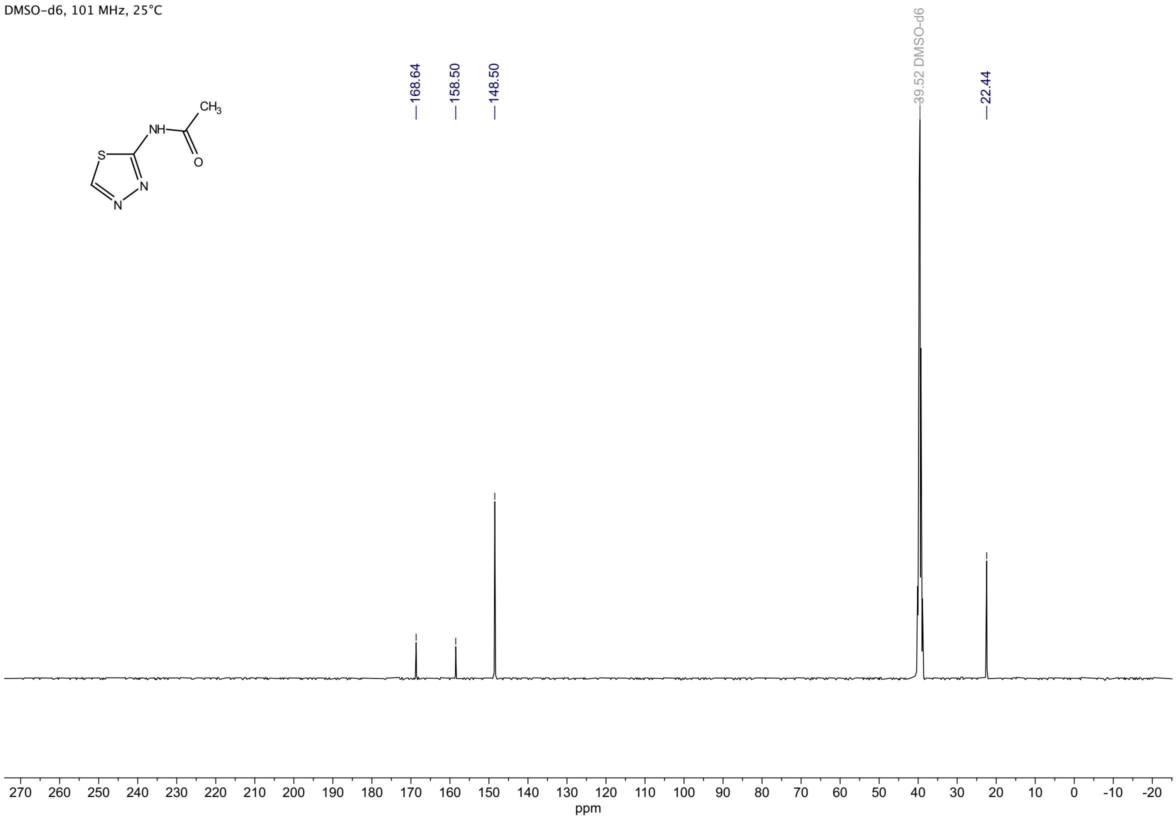

^{1}H NMR of 4-methoxy-5-(trifluoromethyl)-1-((2-(trimethylsilyl)ethoxy)methyl)-*1H*-pyrazole (**3a-C5-CF$_3$**)

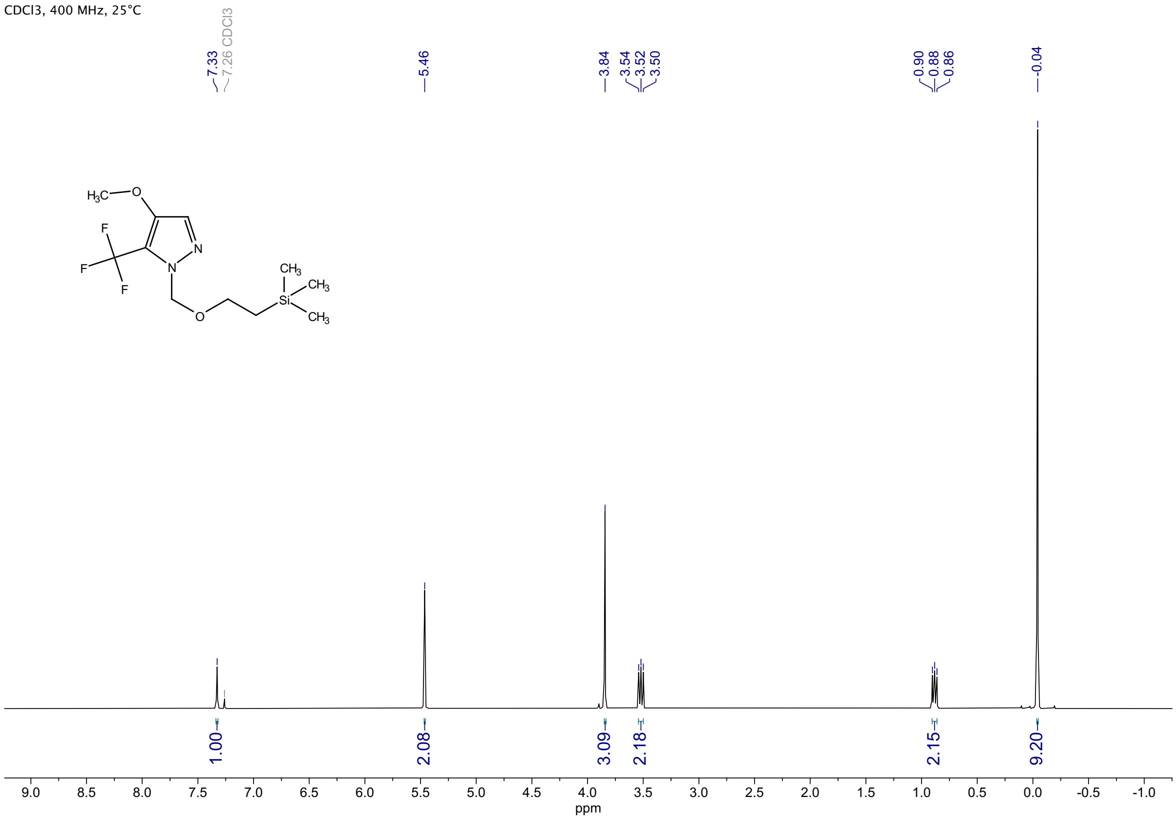

^{13}C NMR of 4-methoxy-5-(trifluoromethyl)-1-((2-(trimethylsilyl)ethoxy)methyl)-*1H*-pyrazole (**3a-C5-CF$_3$**)

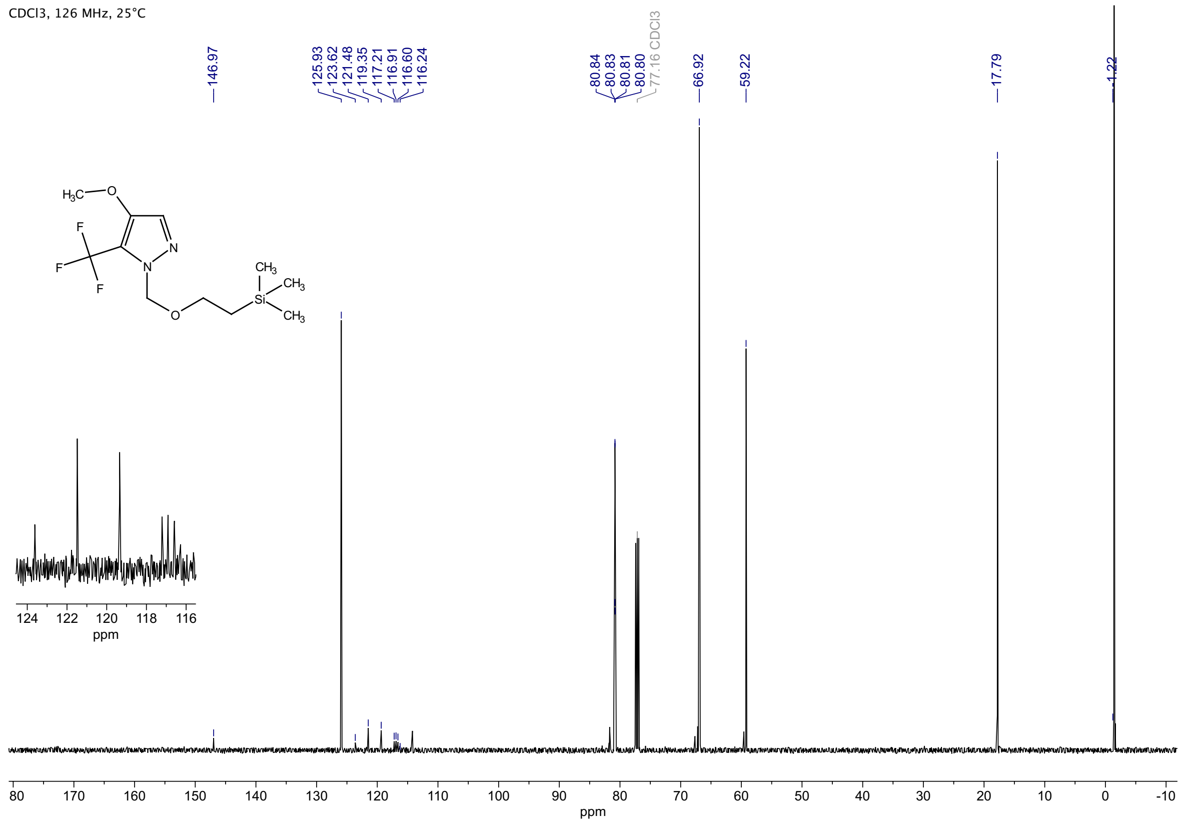

^{19}F NMR of 4-methoxy-5-(trifluoromethyl)-1-((2-(trimethylsilyl)ethoxy)methyl)-*1H*-pyrazole (**3a-C5-CF$_3$**)

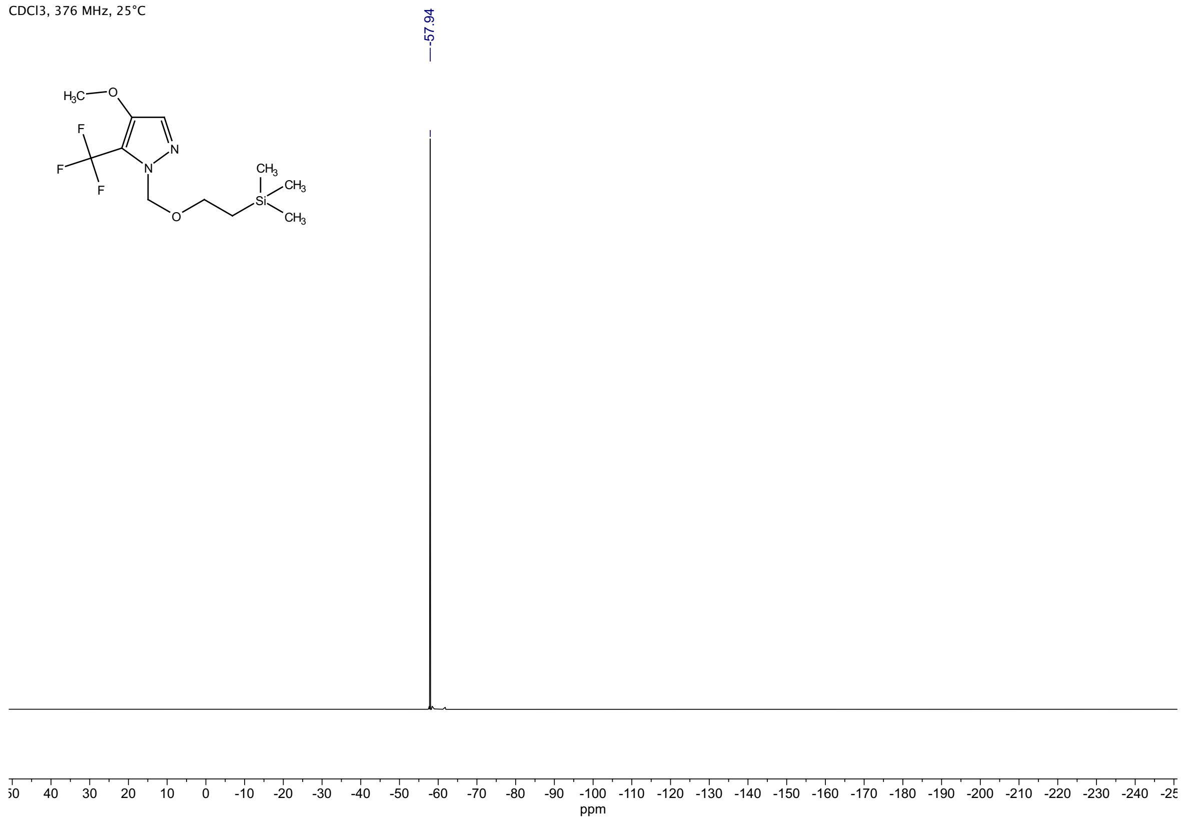

HSQC of 4-methoxy-5-(trifluoromethyl)-1-((2-(trimethylsilyl)ethoxy)methyl)-*1H*-pyrazole (**3a-C5-CF$_3$**)

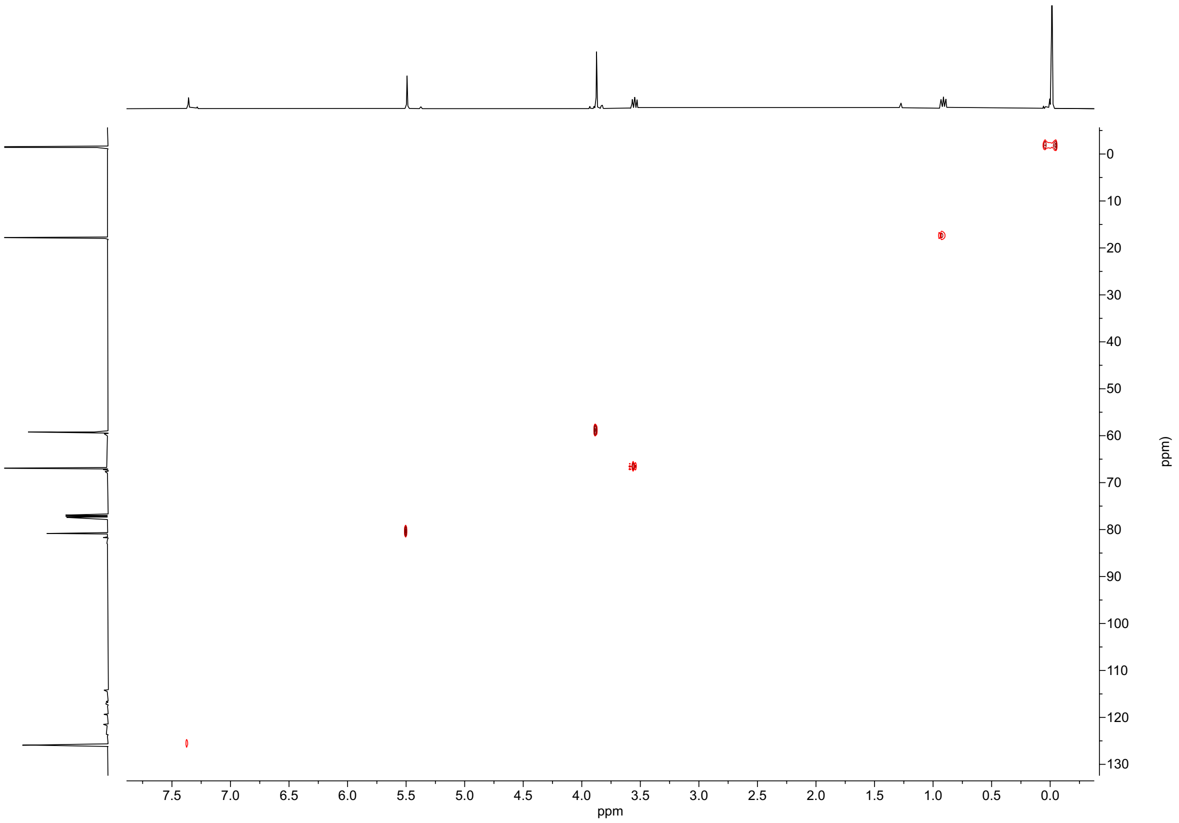

HMBC of 4-methoxy-5-(trifluoromethyl)-1-((2-(trimethylsilyl)ethoxy)methyl)-*1H*-pyrazole (**3a-C5-CF$_3$**)
(key interaction between H6 and C4)

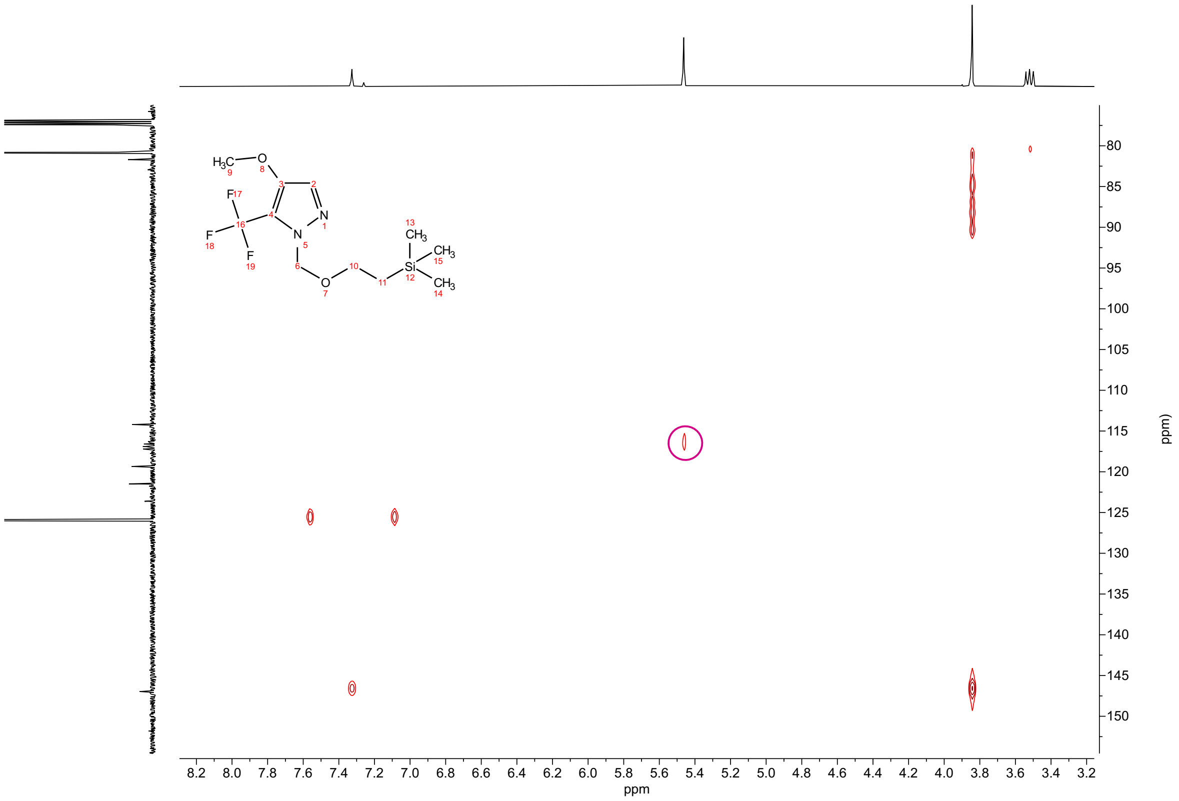

^{1}H NMR of 4-methoxy-3-(trifluoromethyl)-1-((2-(trimethylsilyl)ethoxy)methyl)-*1H*-pyrazole (**3a-C3-CF$_3$**)

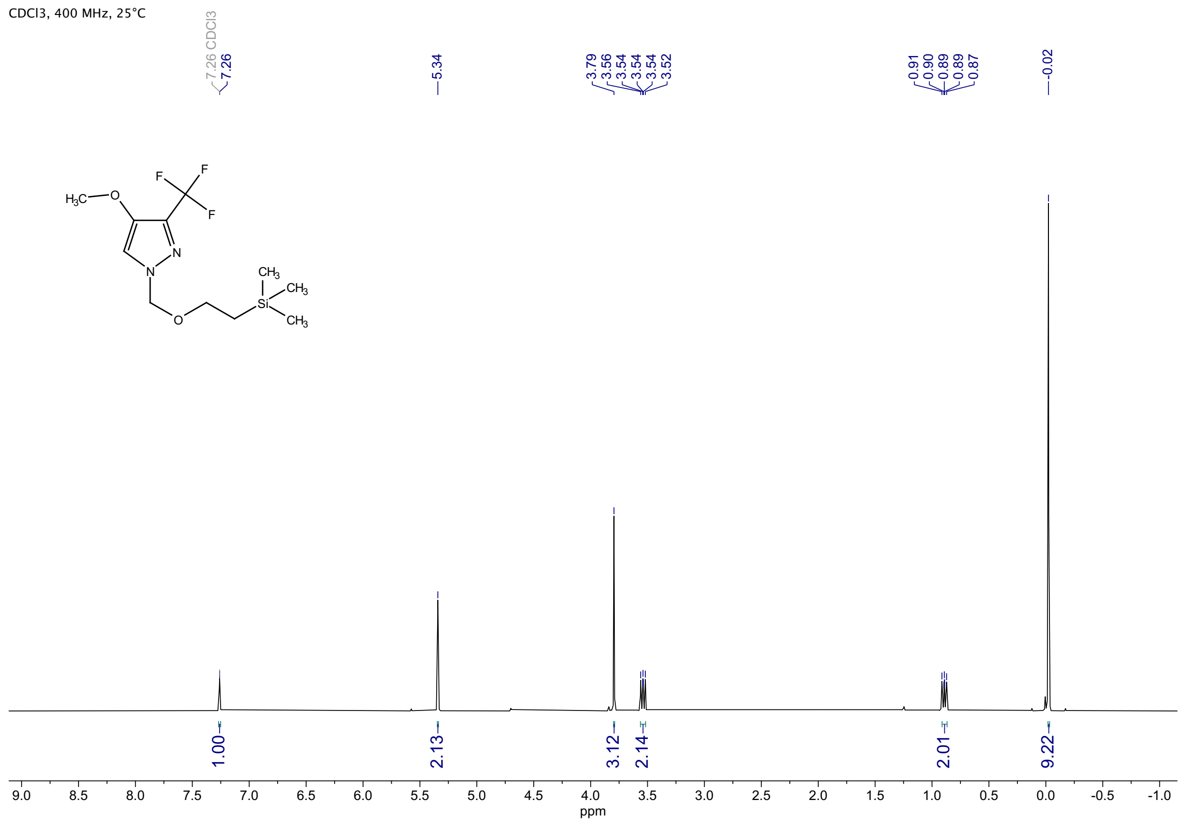

^{13}C NMR of 4-methoxy-3-(trifluoromethyl)-1-((2-(trimethylsilyl)ethoxy)methyl)-*1H*-pyrazole (**3a-C3-CF$_3$**)

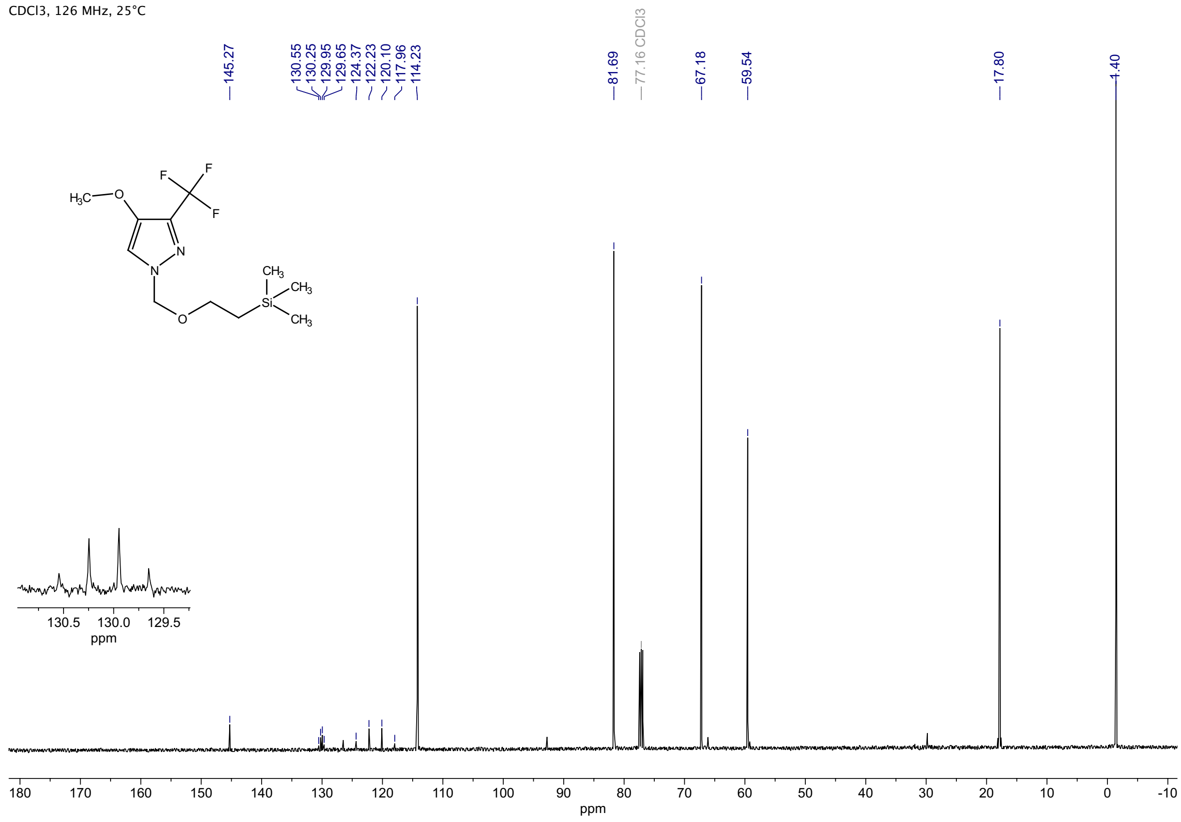

^{19}F NMR of 4-methoxy-3-(trifluoromethyl)-1-((2-(trimethylsilyl)ethoxy)methyl)-*1H*-pyrazole (**3a-C3-CF$_3$**)

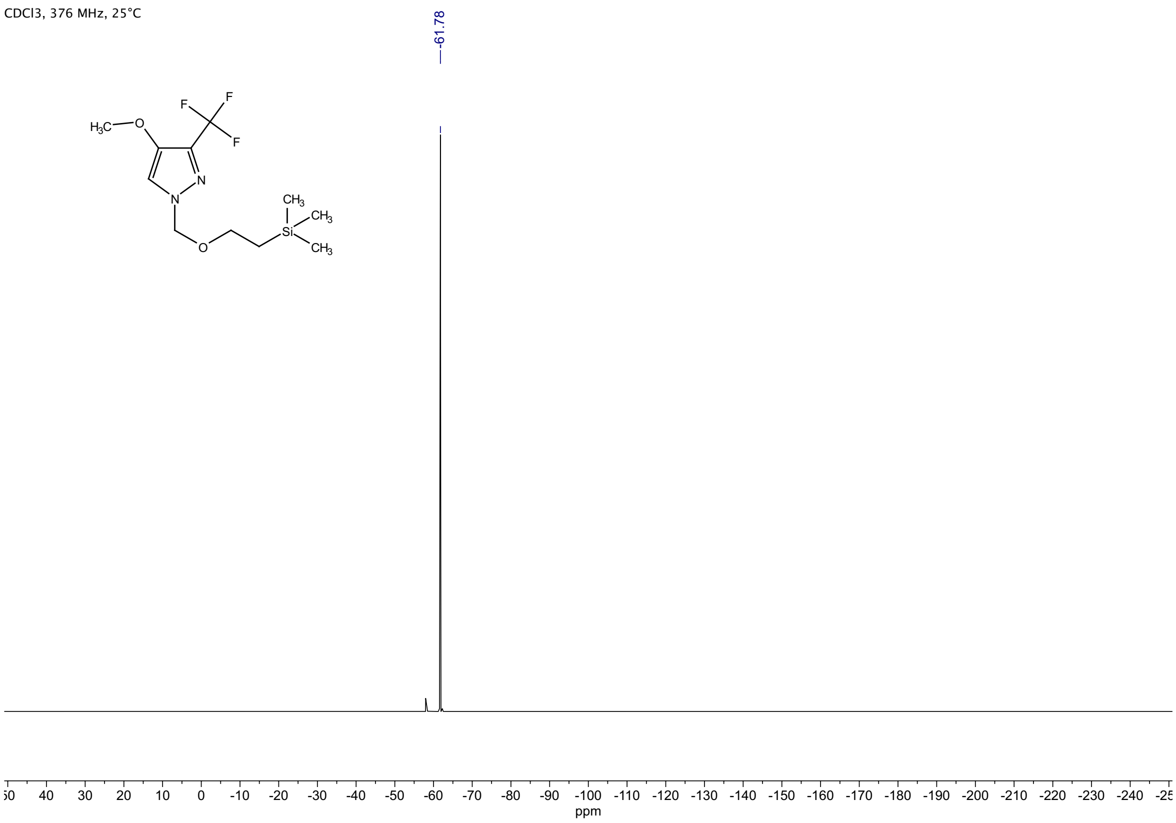

HSQC of 4-methoxy-3-(trifluoromethyl)-1-((2-(trimethylsilyl)ethoxy)methyl)-*1H*-pyrazole (**3a-C3-CF$_3$**)

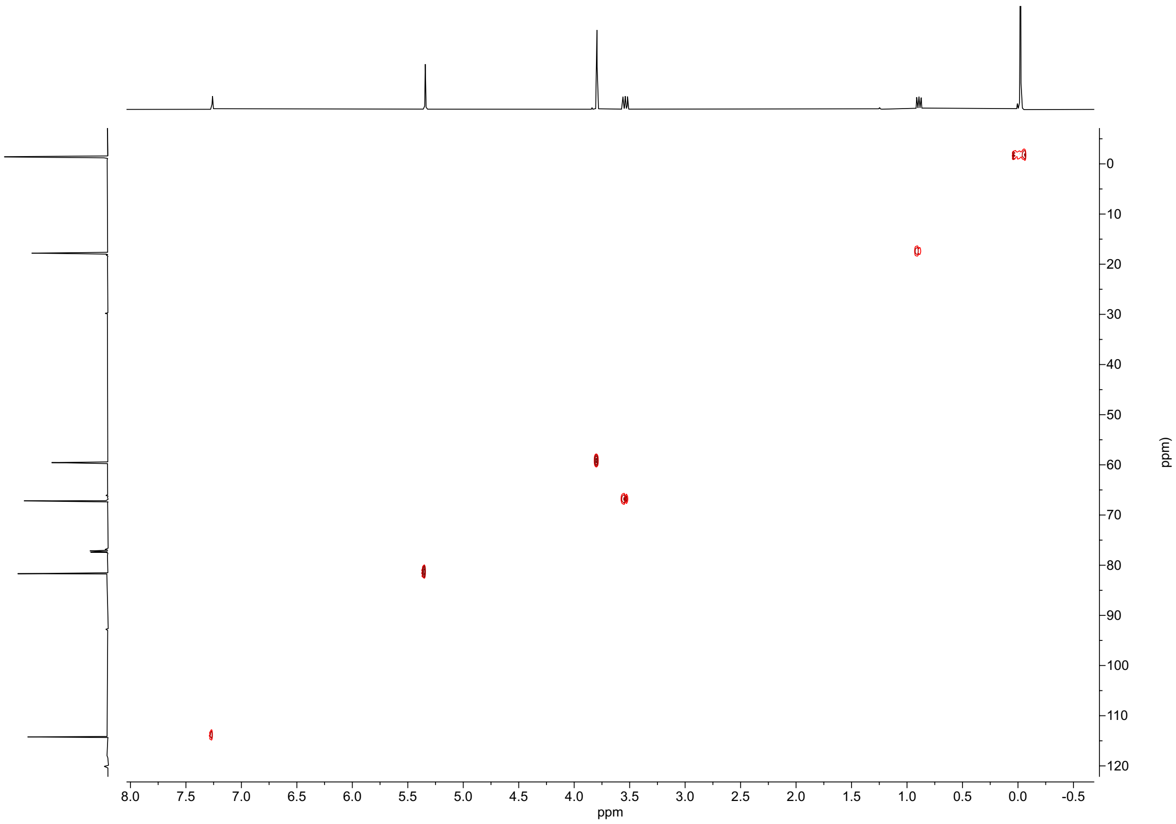

HMBC of 4-methoxy-3-(trifluoromethyl)-1-((2-(trimethylsilyl)ethoxy)methyl)-*1H*-pyrazole (**3a-C3-CF$_3$**) (key interaction between H6 and C4)

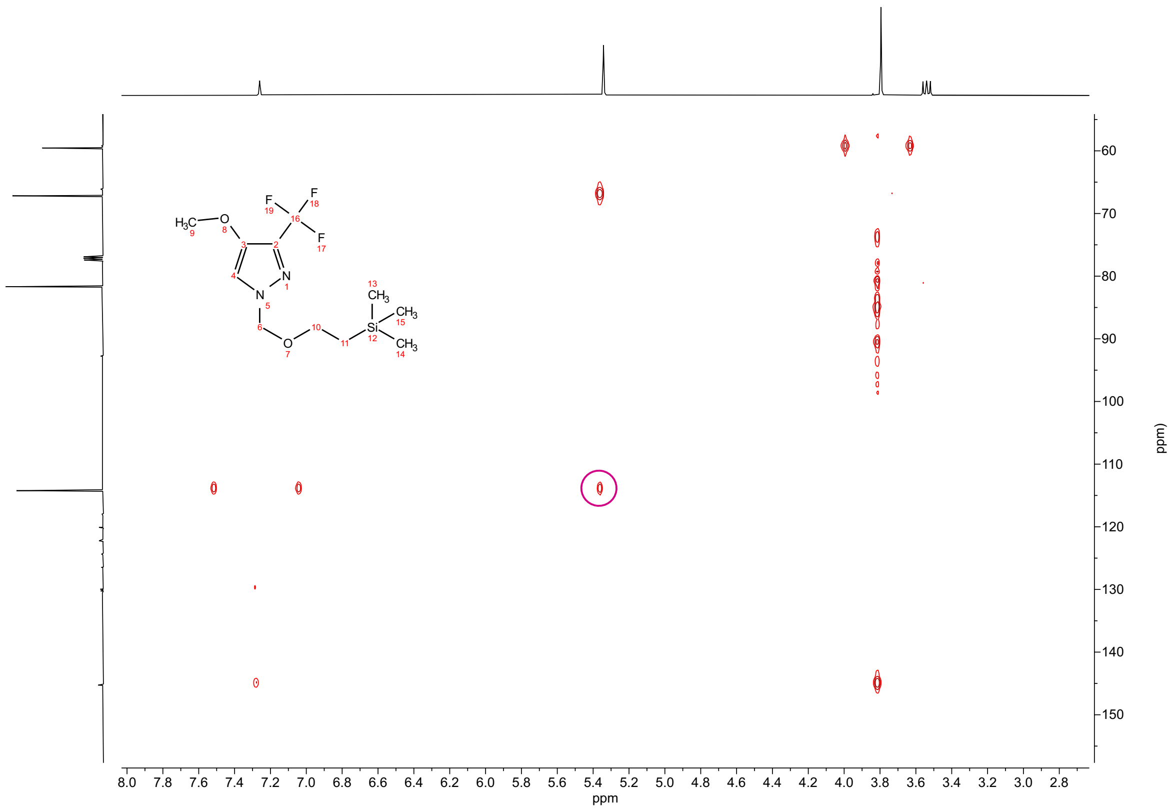

^{1}H NMR of 4-methyl-5-(trifluoromethyl)-1-((2-(trimethylsilyl)ethoxy)methyl)-*1H*-pyrazole (**3b-C5-CF$_3$**)

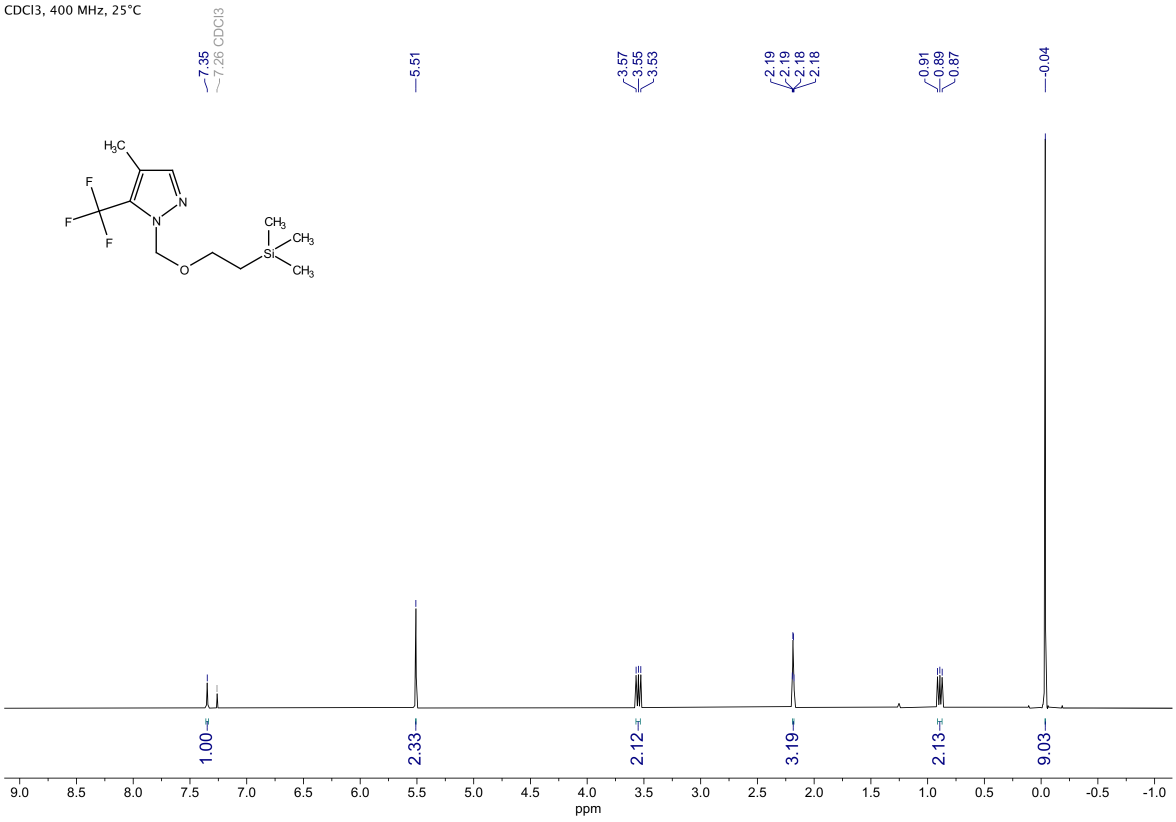

^{13}C NMR of 4-methyl-5-(trifluoromethyl)-1-((2-(trimethylsilyl)ethoxy)methyl)-*1H*-pyrazole (**3b-C5-CF$_3$**)

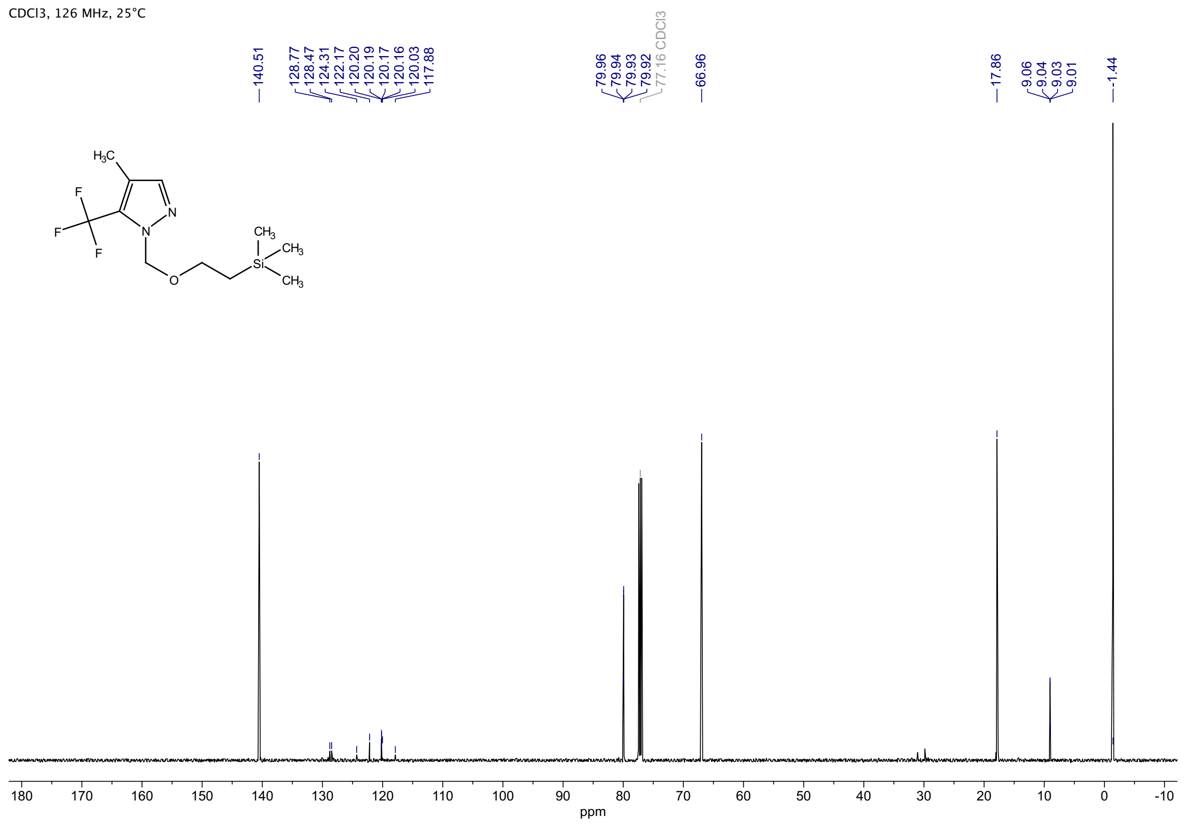

Zoom of ^{13}C NMR of 4-methyl-5-(trifluoromethyl)-1-((2-(trimethylsilyl)ethoxy)methyl)-*1H*-pyrazole (**3b-C5-CF$_3$**)

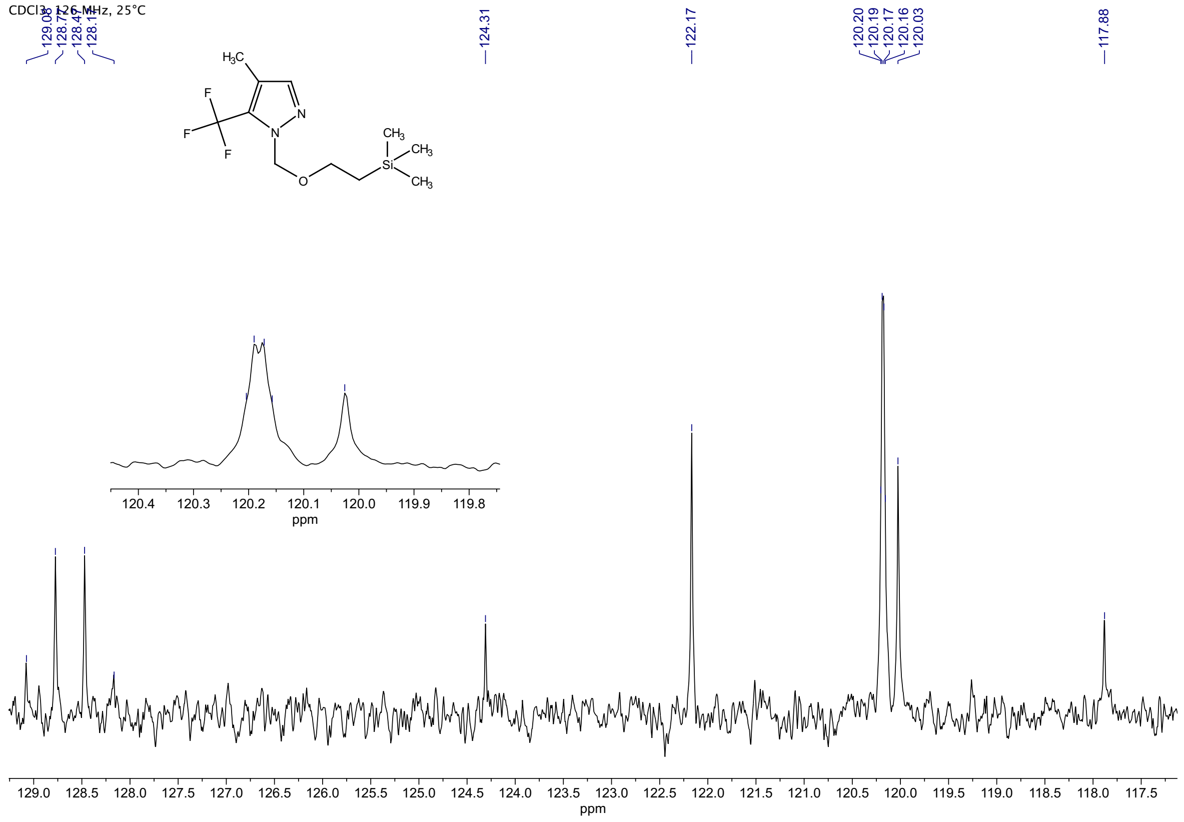

^{19}F NMR of 4-methyl-5-(trifluoromethyl)-1-((2-(trimethylsilyl)ethoxy)methyl)-*1H*-pyrazole (**3b-C5-CF$_3$**)

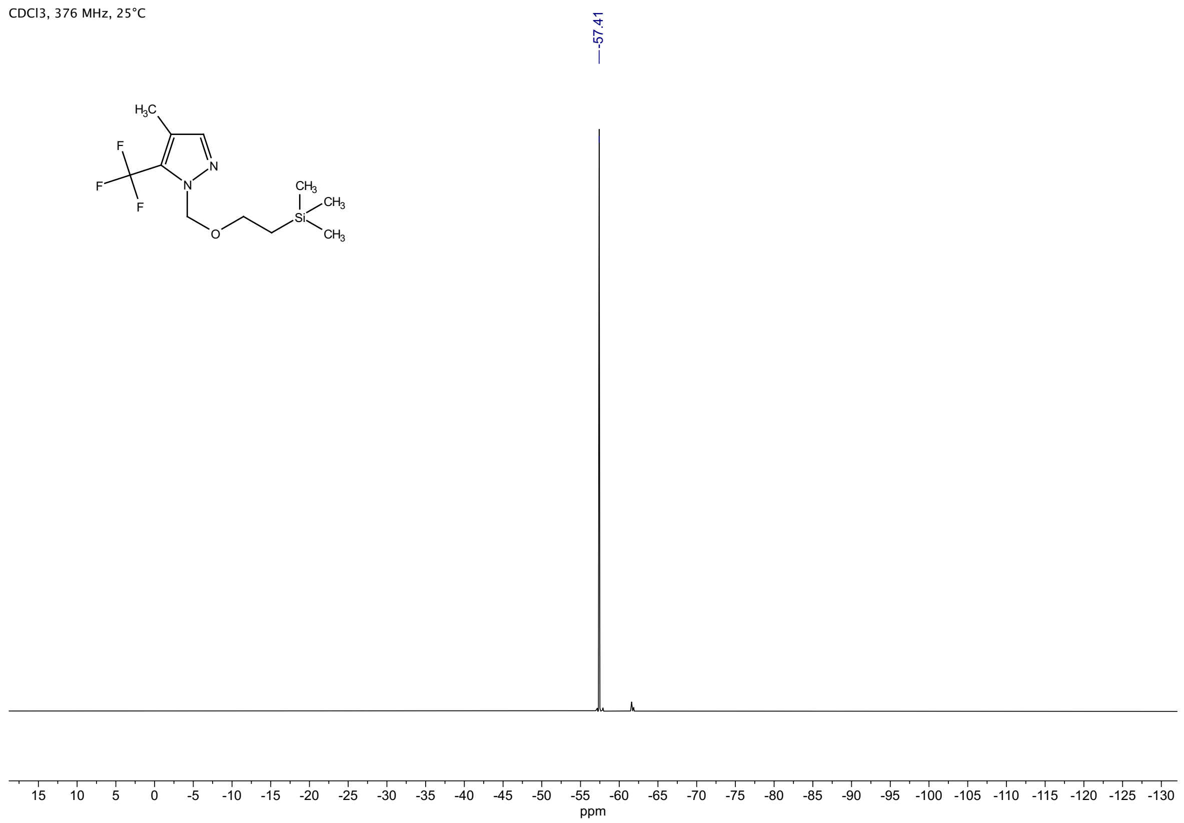

HSQC of 4-methyl-5-(trifluoromethyl)-1-((2-(trimethylsilyl)ethoxy)methyl)-*1H*-pyrazole (**3b-C5-CF$_3$**)

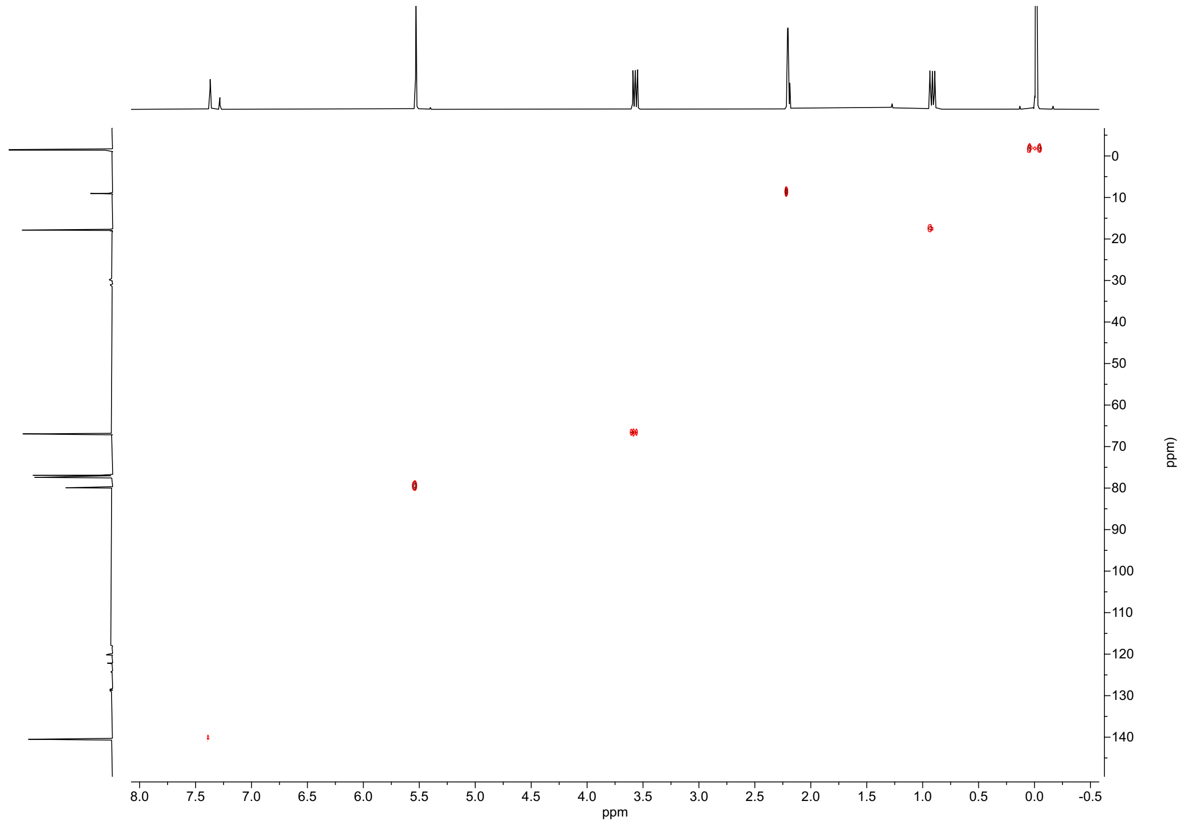

HMBC of 4-methyl-5-(trifluoromethyl)-1-((2-(trimethylsilyl)ethoxy)methyl)-*1H*-pyrazole (**3b-C5-CF$_3$**)
(key interaction between H6 and C4)

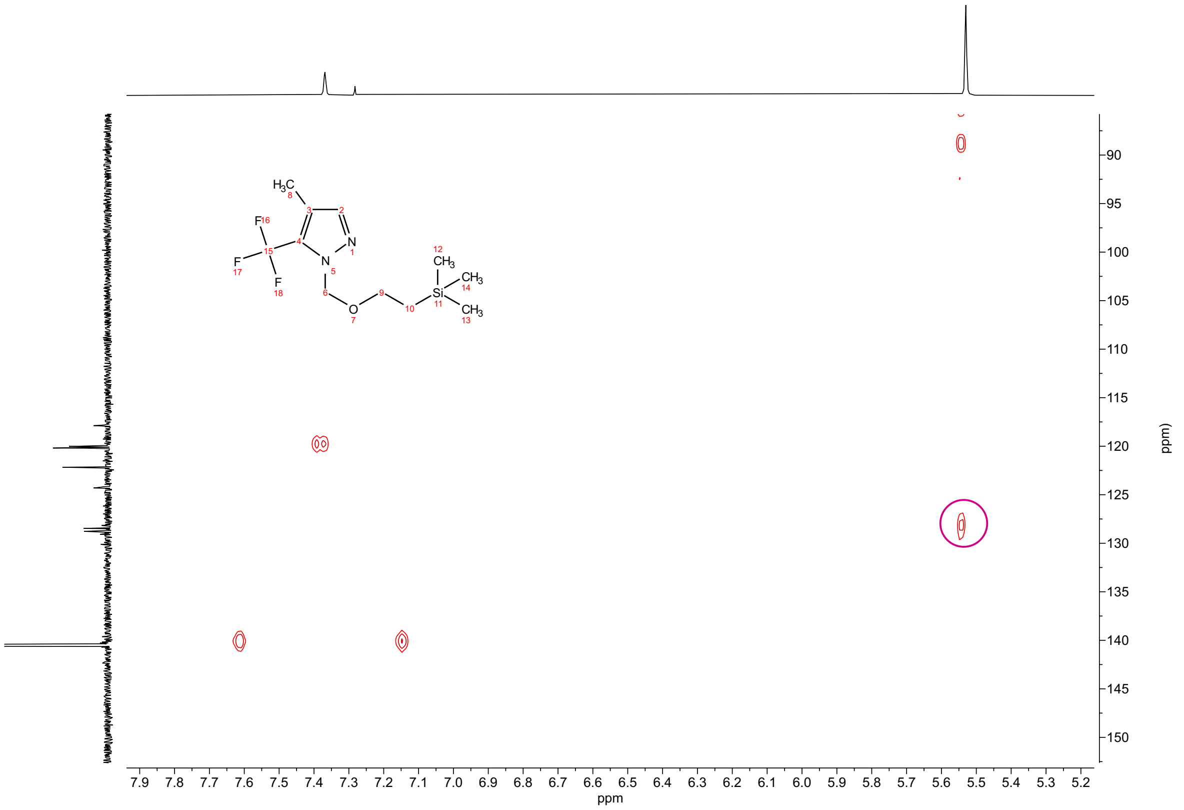

^{1}H NMR of 1-((perfluorophenyl)methyl)-5-(trifluoromethyl)-*1H*-pyrazole (**3c-C5-CF$_3$**)

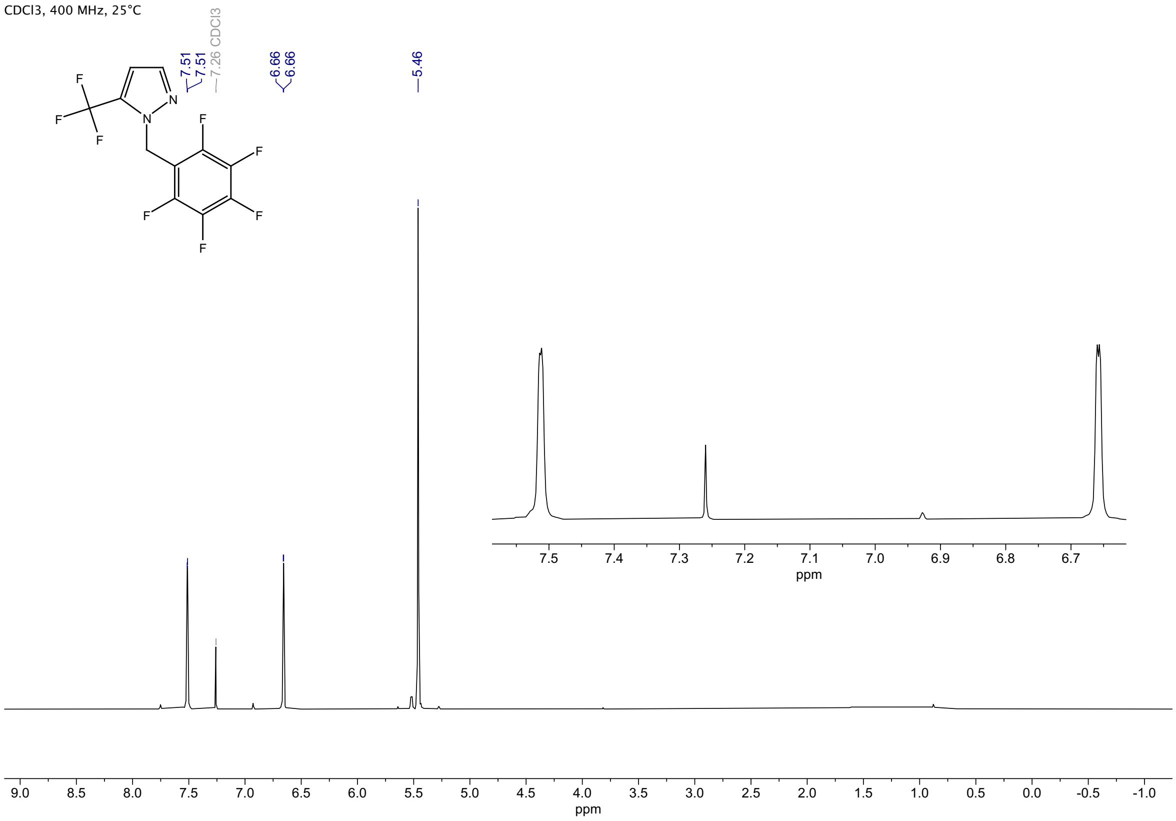

^{13}C NMR of 1-((perfluorophenyl)methyl)-5-(trifluoromethyl)-*1H*-pyrazole (**3c-C5-CF$_3$**)

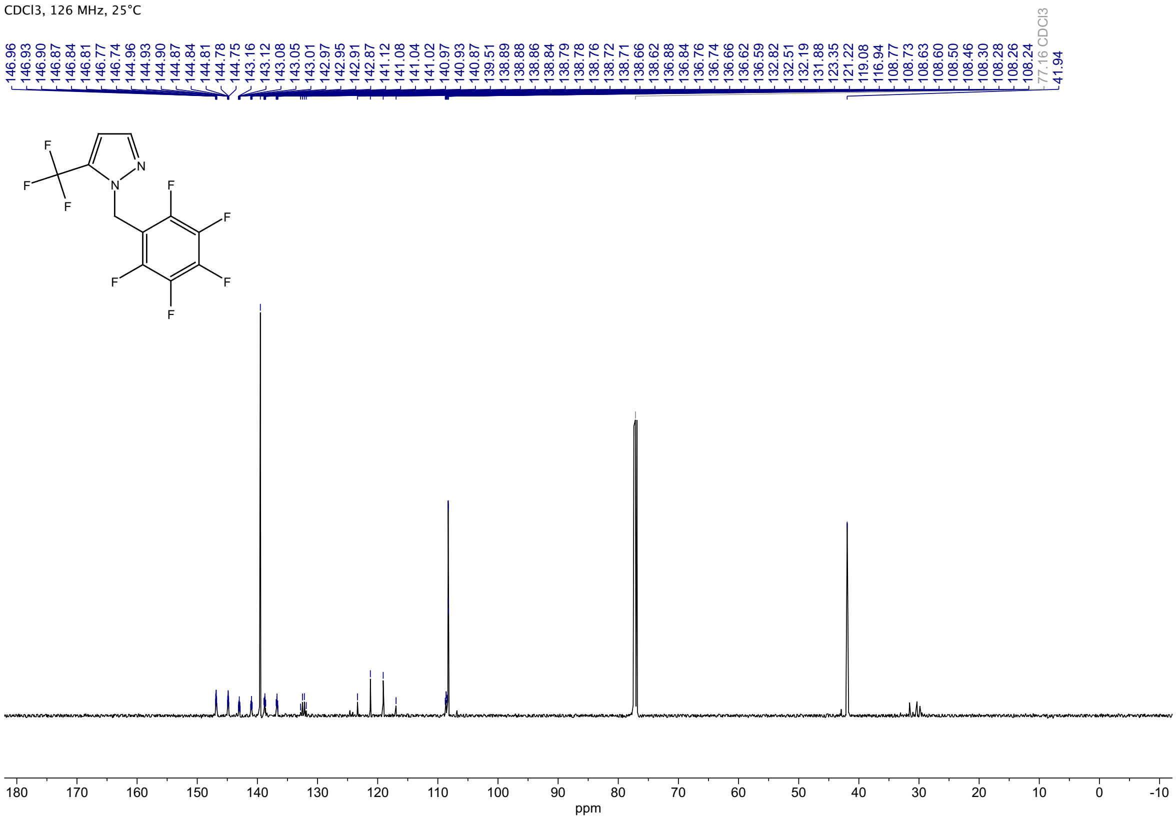

Zoom on ^{13}C NMR of 1-((perfluorophenyl)methyl)-5-(trifluoromethyl)-*1H*-pyrazole (**3c-C5-CF$_3$**)

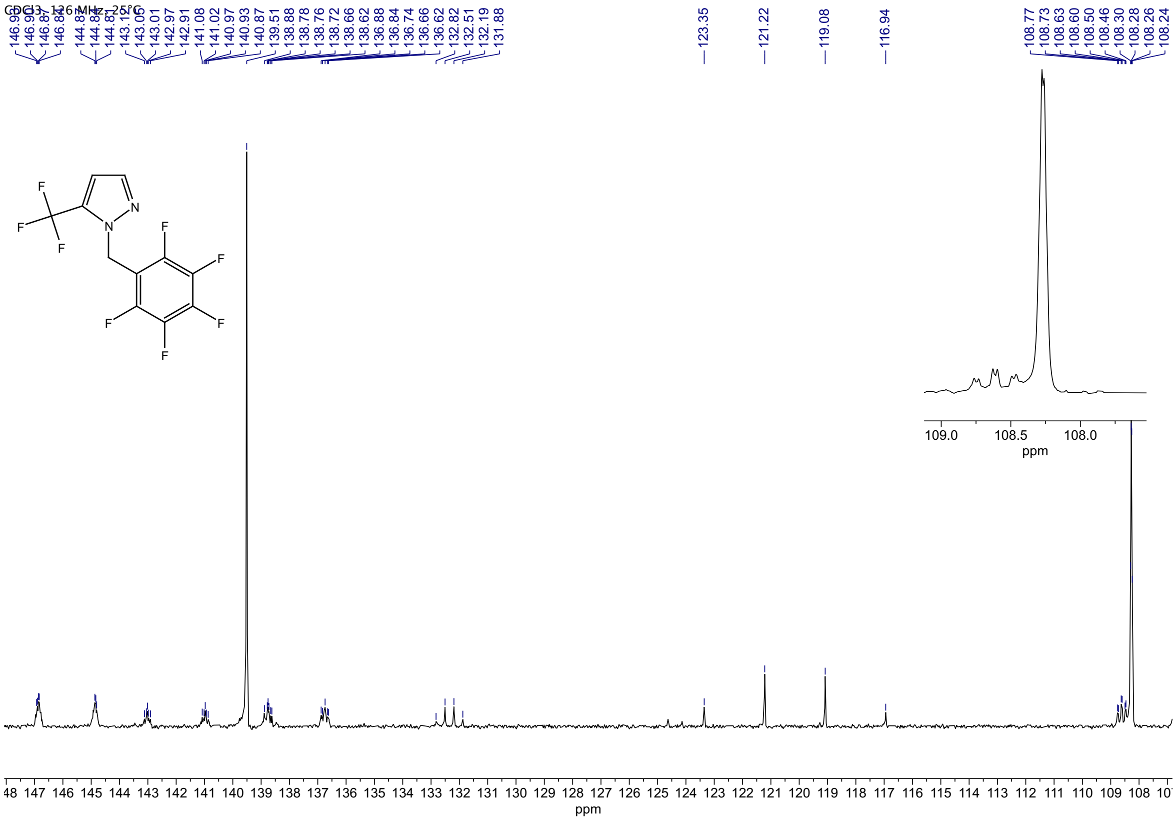

^{19}F NMR of 1-((perfluorophenyl)methyl)-5-(trifluoromethyl)-*1H*-pyrazole (**3c-C5-CF$_3$**)

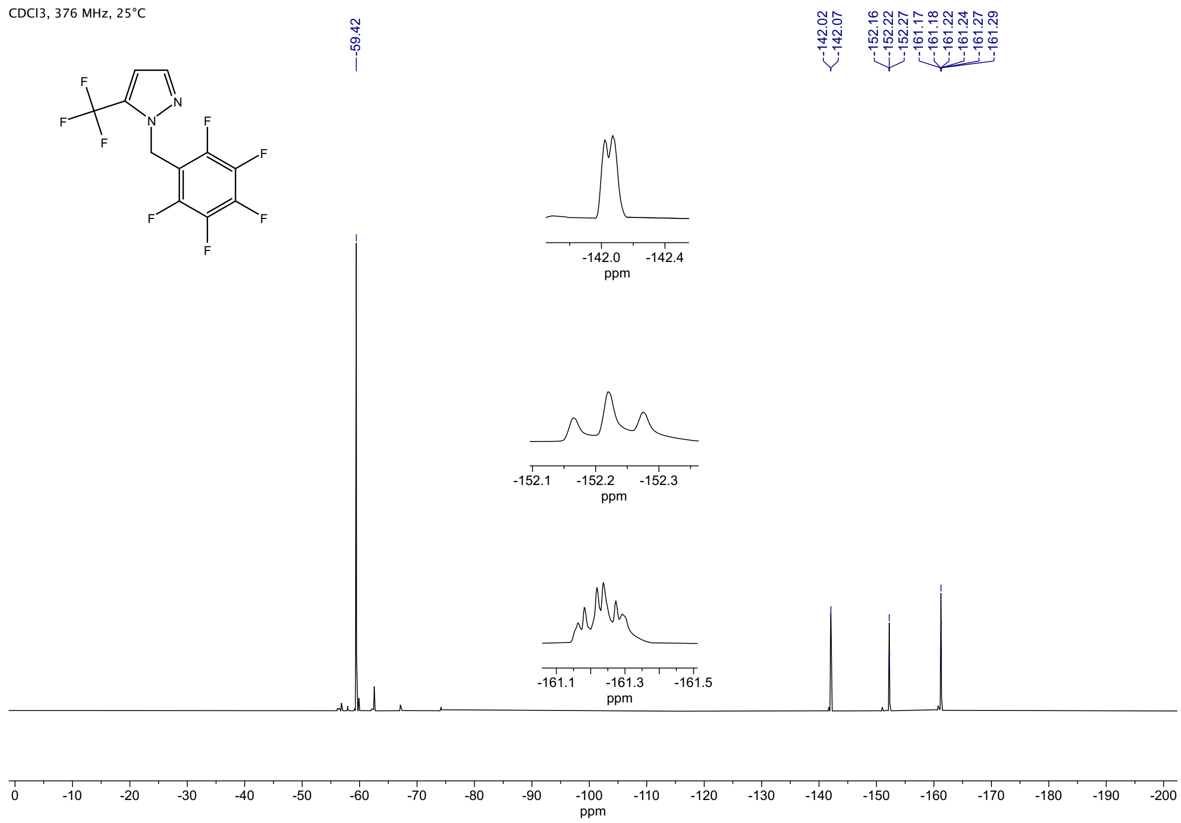

HSQC of 1-((perfluorophenyl)methyl)-5-(trifluoromethyl)-*1H*-pyrazole (**3c-C5-CF$_3$**)

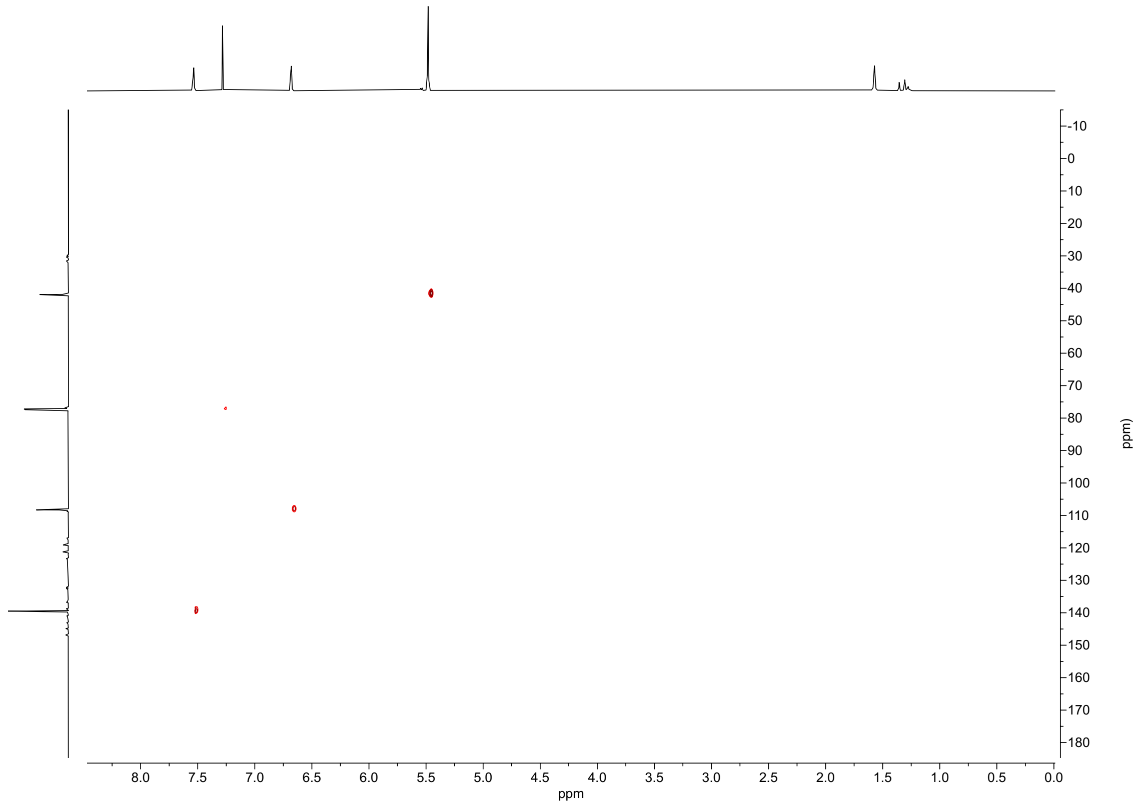

HMBC of 1-((perfluorophenyl)methyl)-5-(trifluoromethyl)-*1H*-pyrazole (**3c-C5-CF$_3$**)

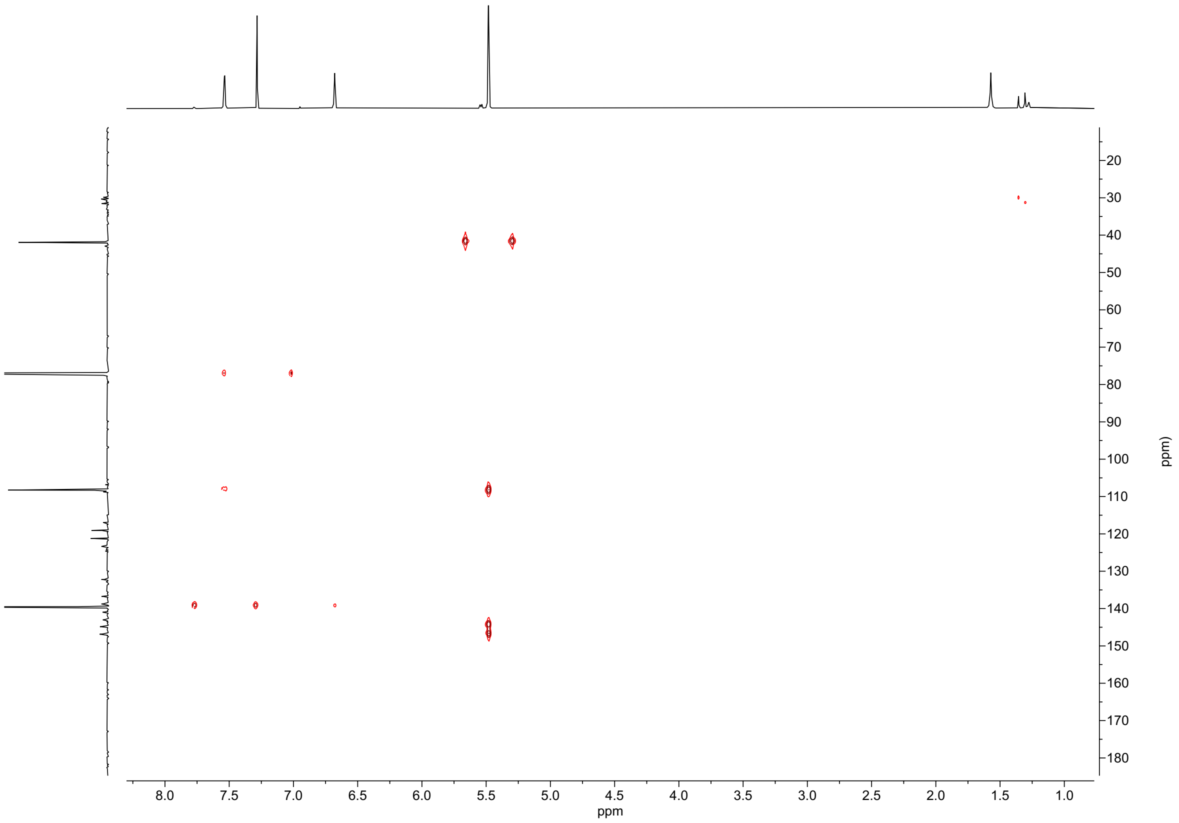

^{1}H NMR of 1-((perfluorophenyl)methyl)-3-(trifluoromethyl)-*1H*-pyrazole (**3c-C3-CF$_3$**)

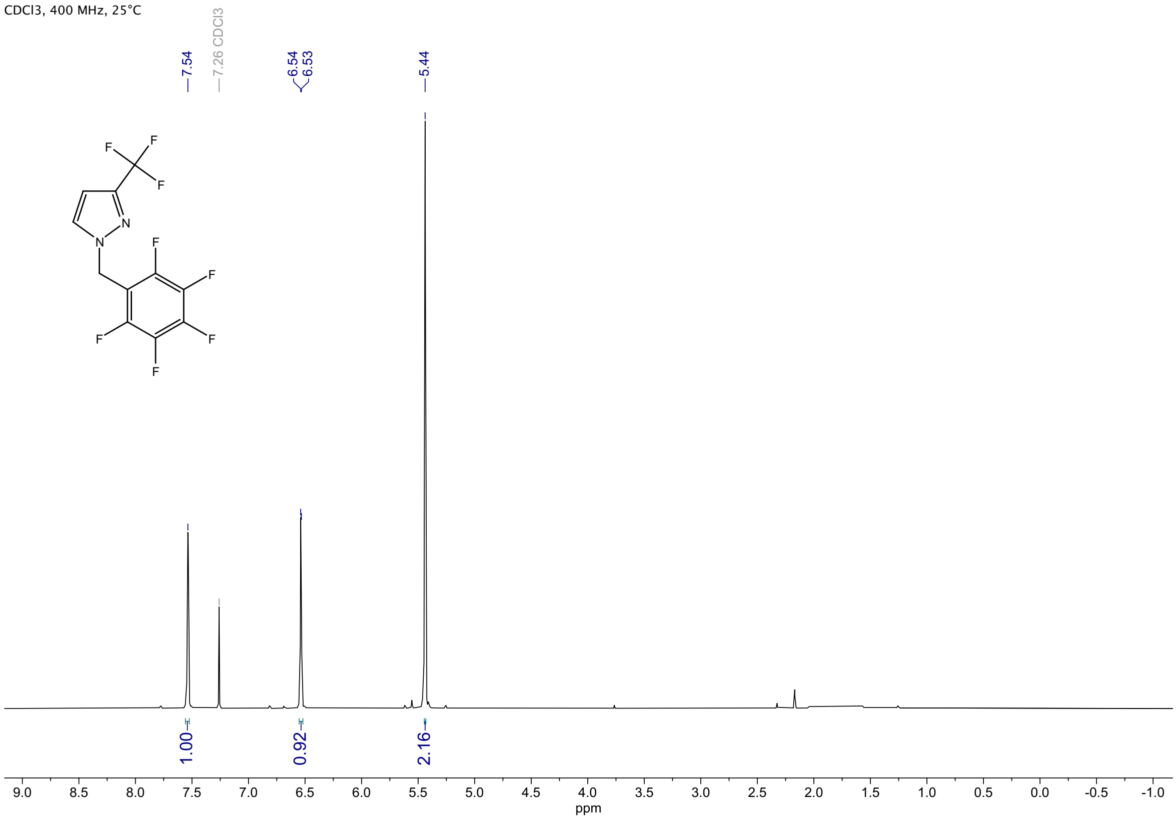

^{13}C NMR of 1-((perfluorophenyl)methyl)-3-(trifluoromethyl)-*1H*-pyrazole (**3c-C3-CF3**)

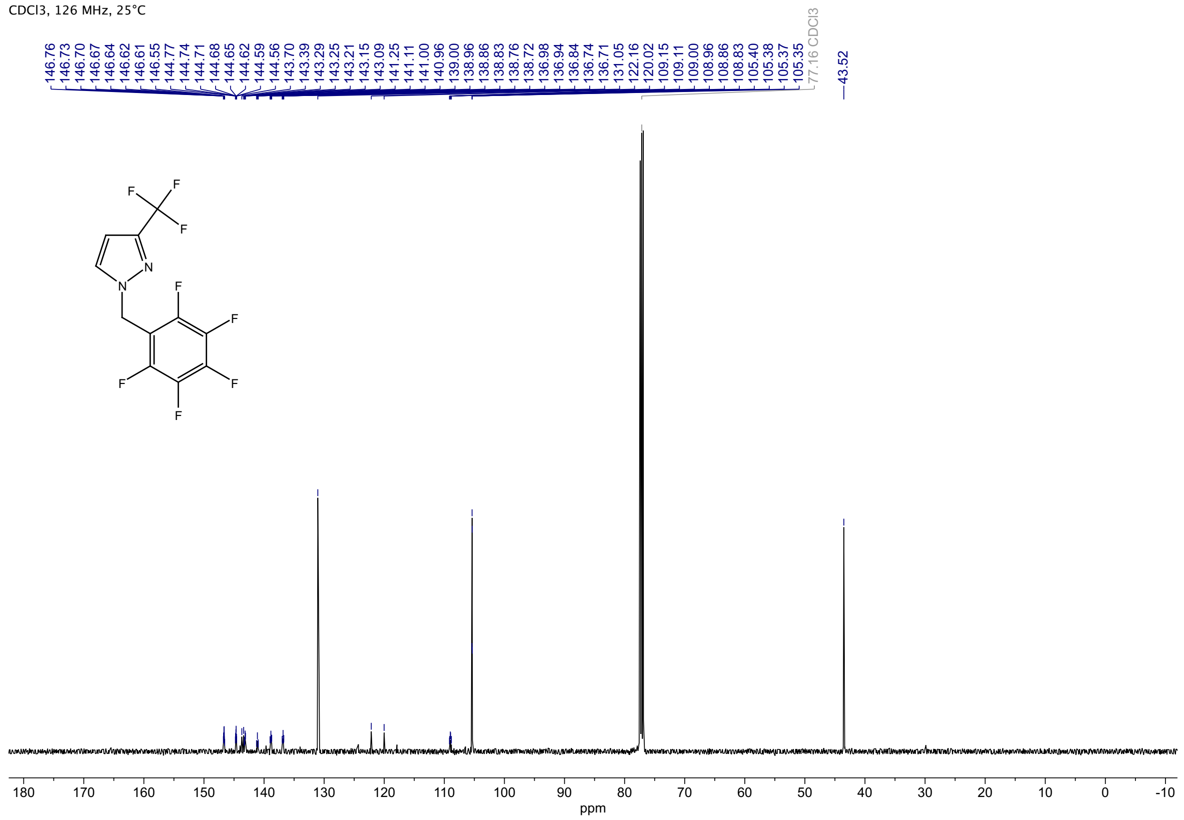

Zoom on ^{13}C NMR of 1-((perfluorophenyl)methyl)-3-(trifluoromethyl)-*1H*-pyrazole (**3c-C3-CF3**)

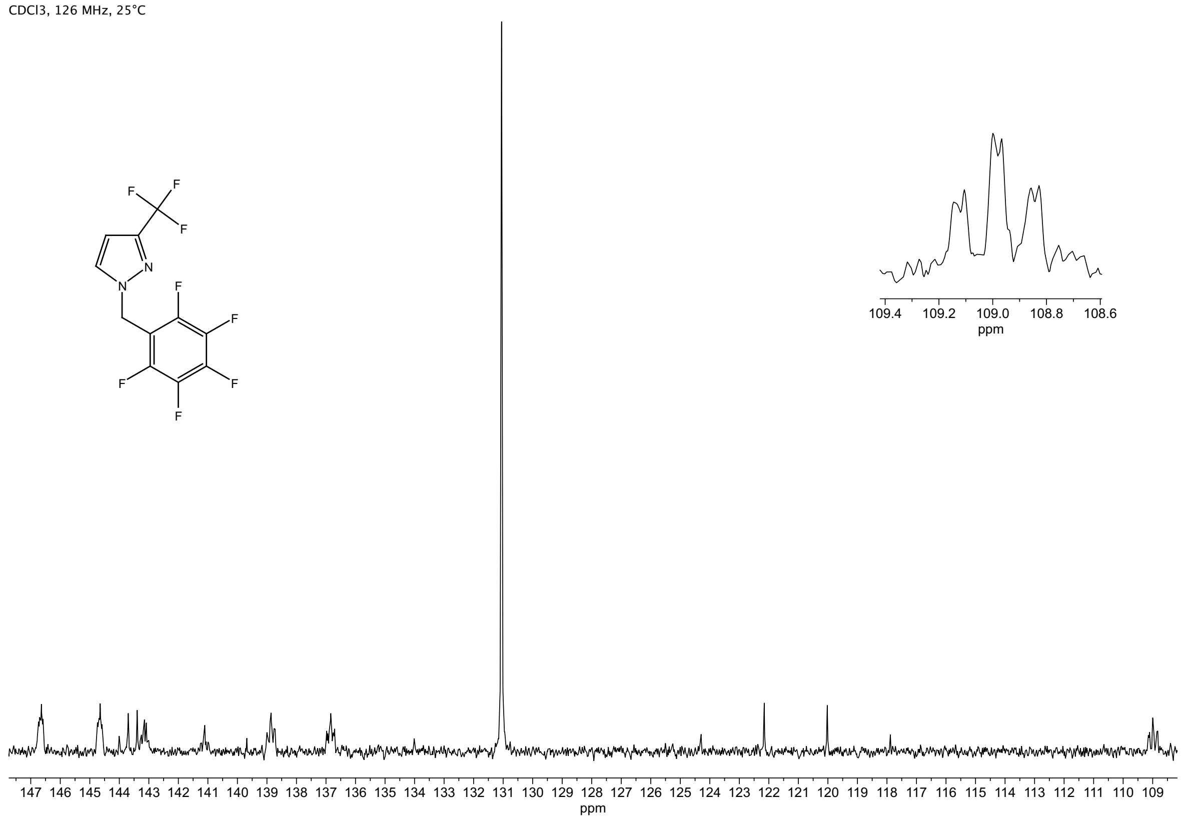

^{19}F NMR of 1-((perfluorophenyl)methyl)-3-(trifluoromethyl)-*1H*-pyrazole (**3c-C3-CF$_3$**)

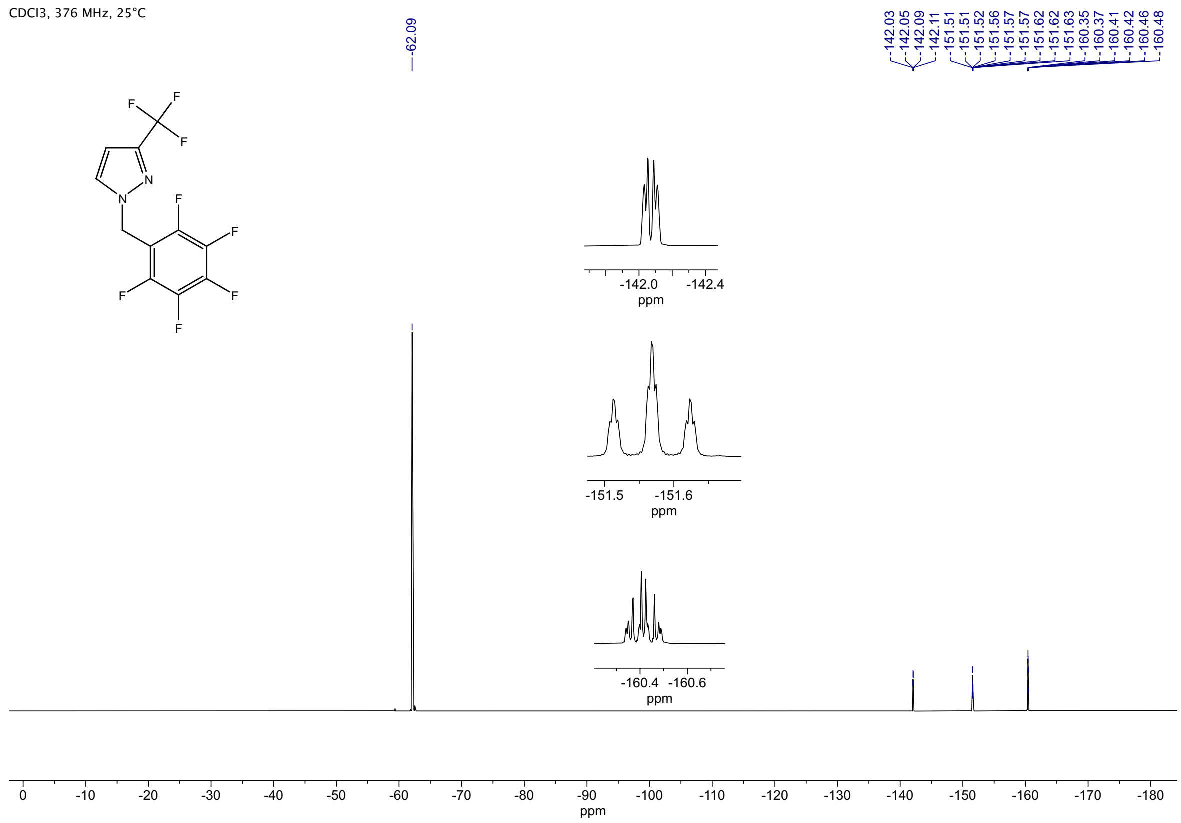

HSQC of 1-((perfluorophenyl)methyl)-3-(trifluoromethyl)-*1H*-pyrazole (**3c-C3-CF$_3$**)

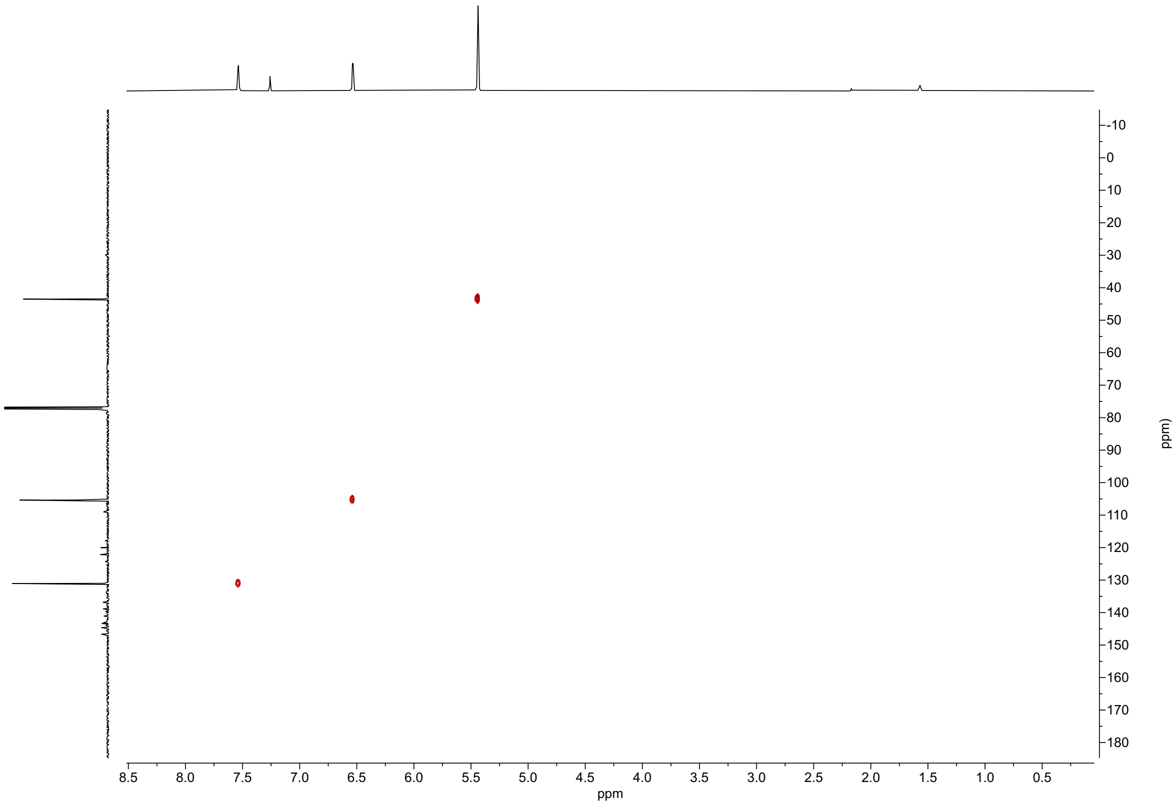

HMBC of 1-((perfluorophenyl)methyl)-3-(trifluoromethyl)-*1H*-pyrazole (**3c-C3-CF$_3$**)
(key interaction between H6 and C4)

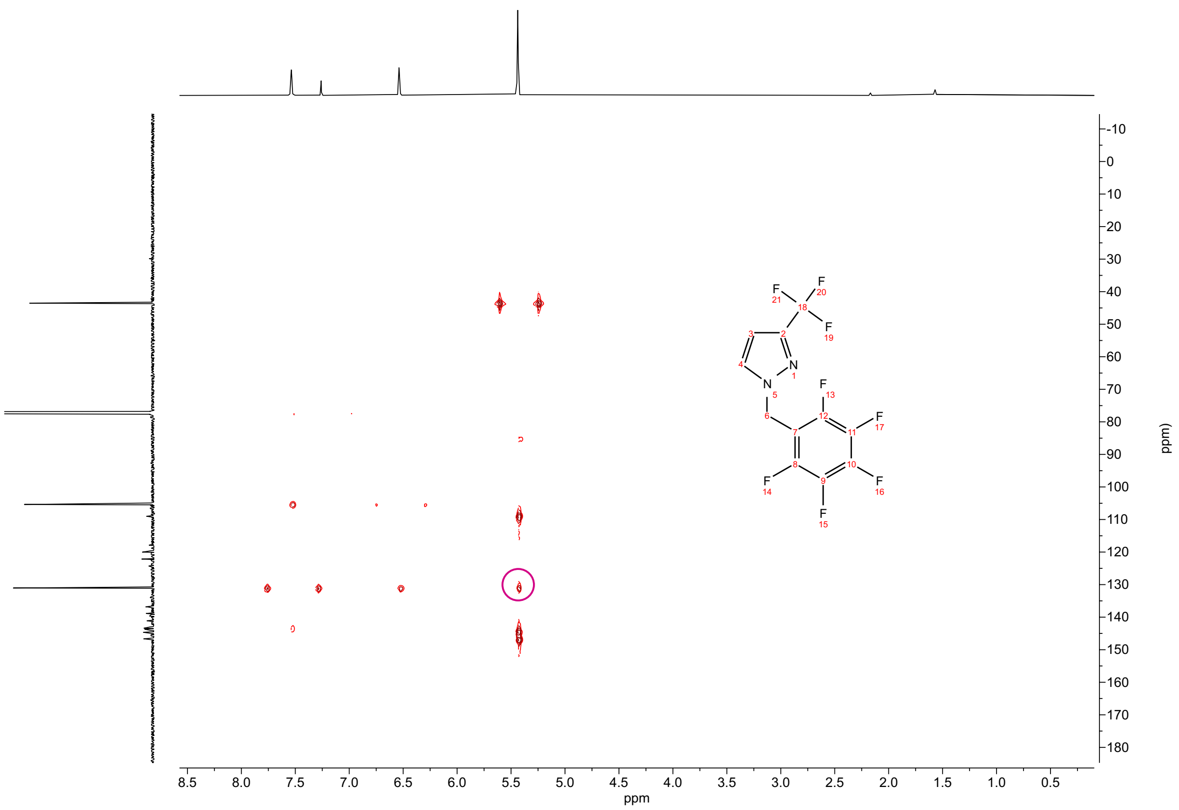

^{1}H NMR of 4-methoxy-5-(trifluoromethyl)-*1H*-pyrazole (**4a-monoCF$_3$**)

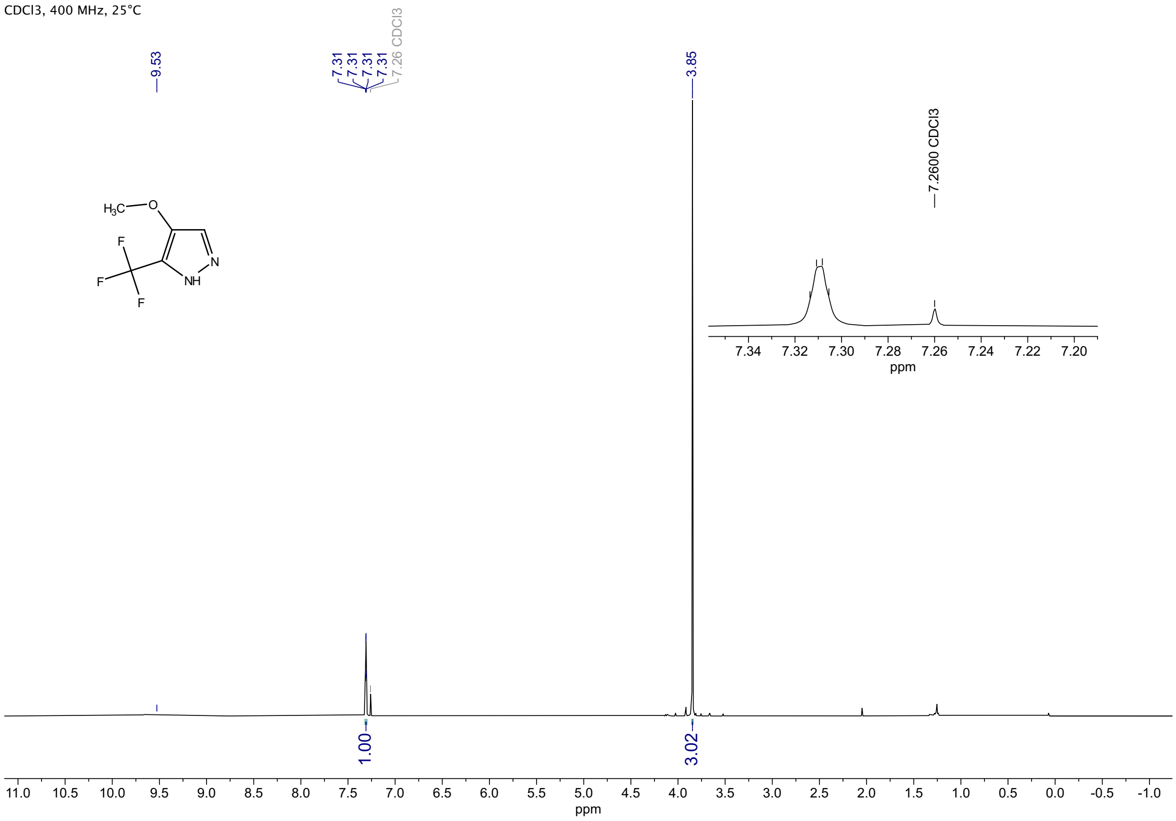

^{13}C NMR of 4-methoxy-5-(trifluoromethyl)-*1H*-pyrazole (**4a-monoCF$_3$**)

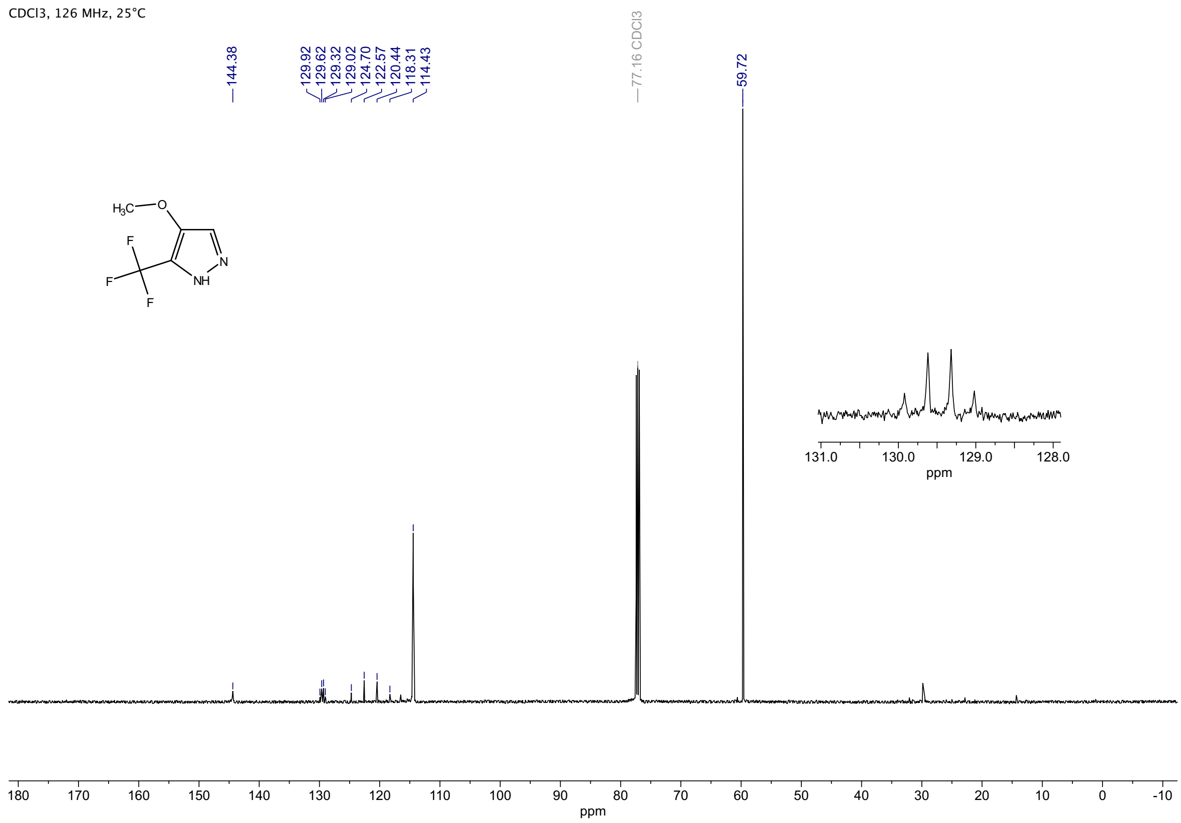

^{19}F NMR of 4-methoxy-5-(trifluoromethyl)-*1H*-pyrazole (**4a-monoCF$_3$**)

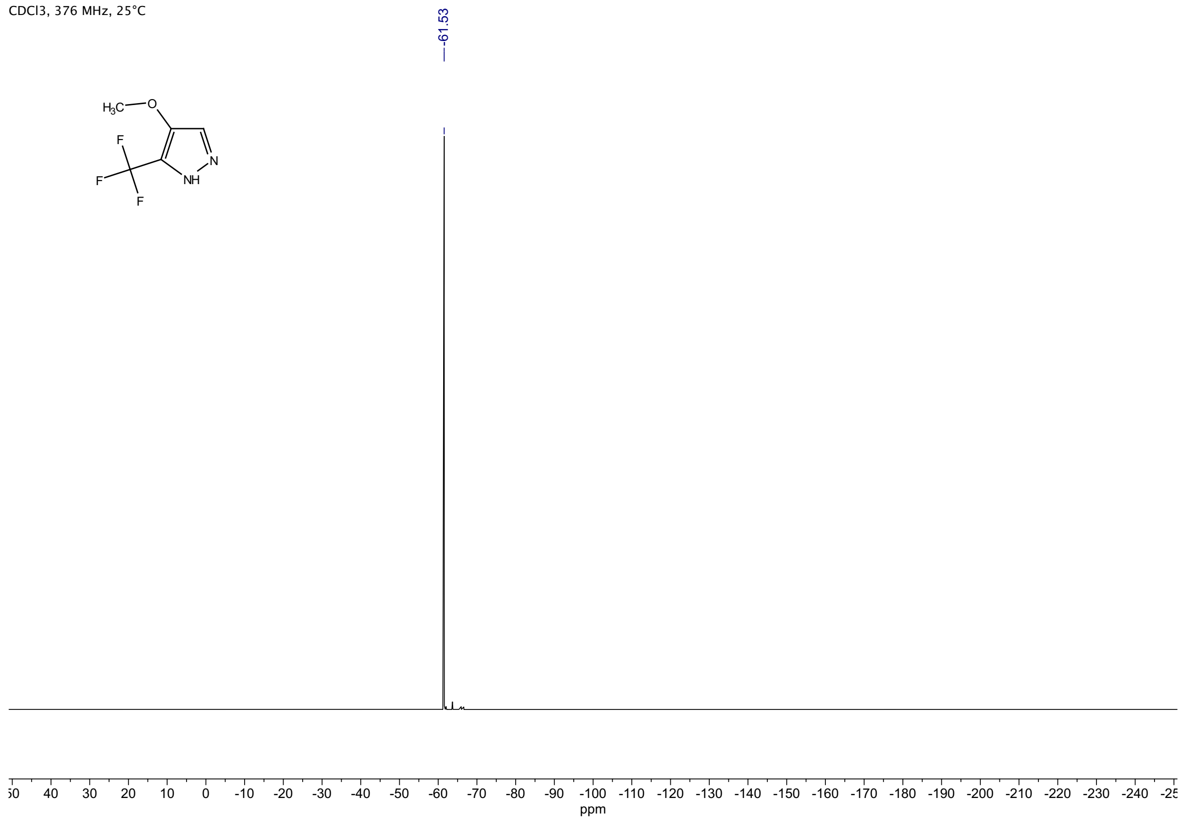

^{1}H NMR of 4-methoxy-3,5-bis(trifluoromethyl)-*1H*-pyrazole (**4a-diCF$_3$**)

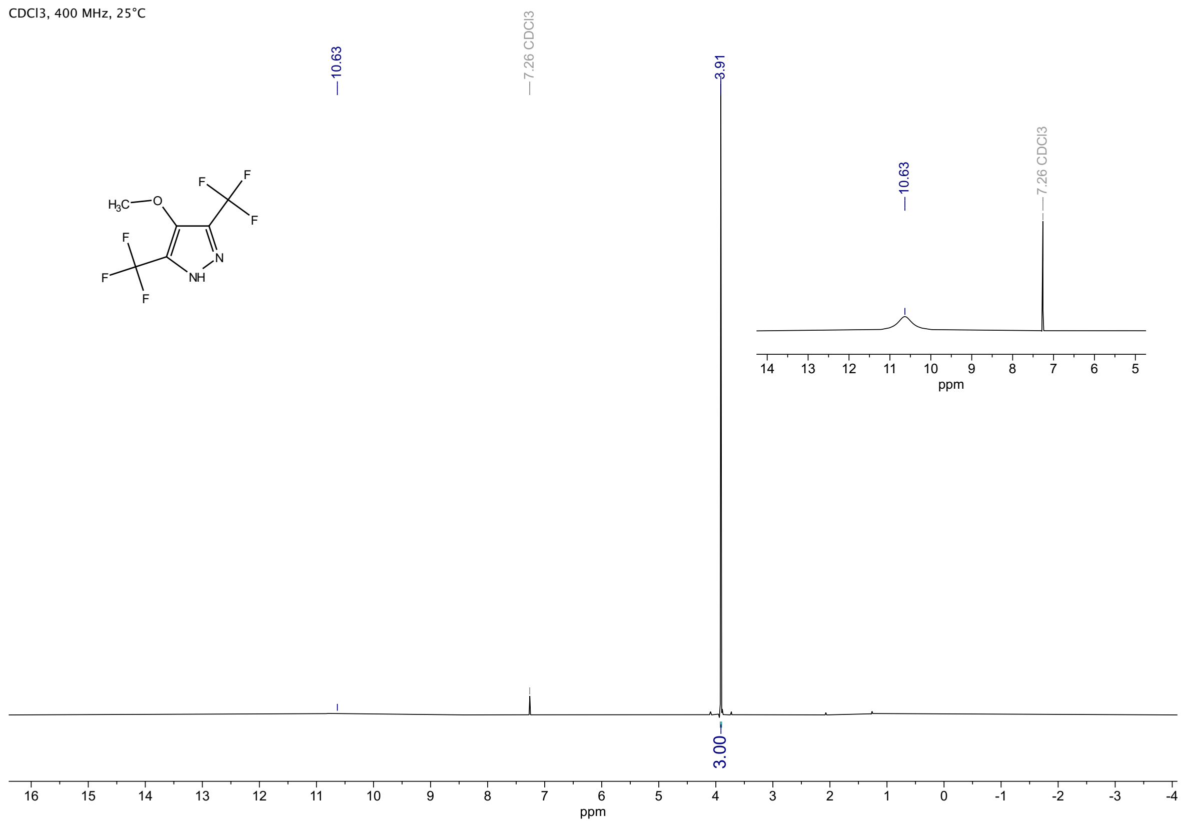

^{13}C NMR of 4-methoxy-3,5-bis(trifluoromethyl)-*1H*-pyrazole (**4a-diCF$_3$**)

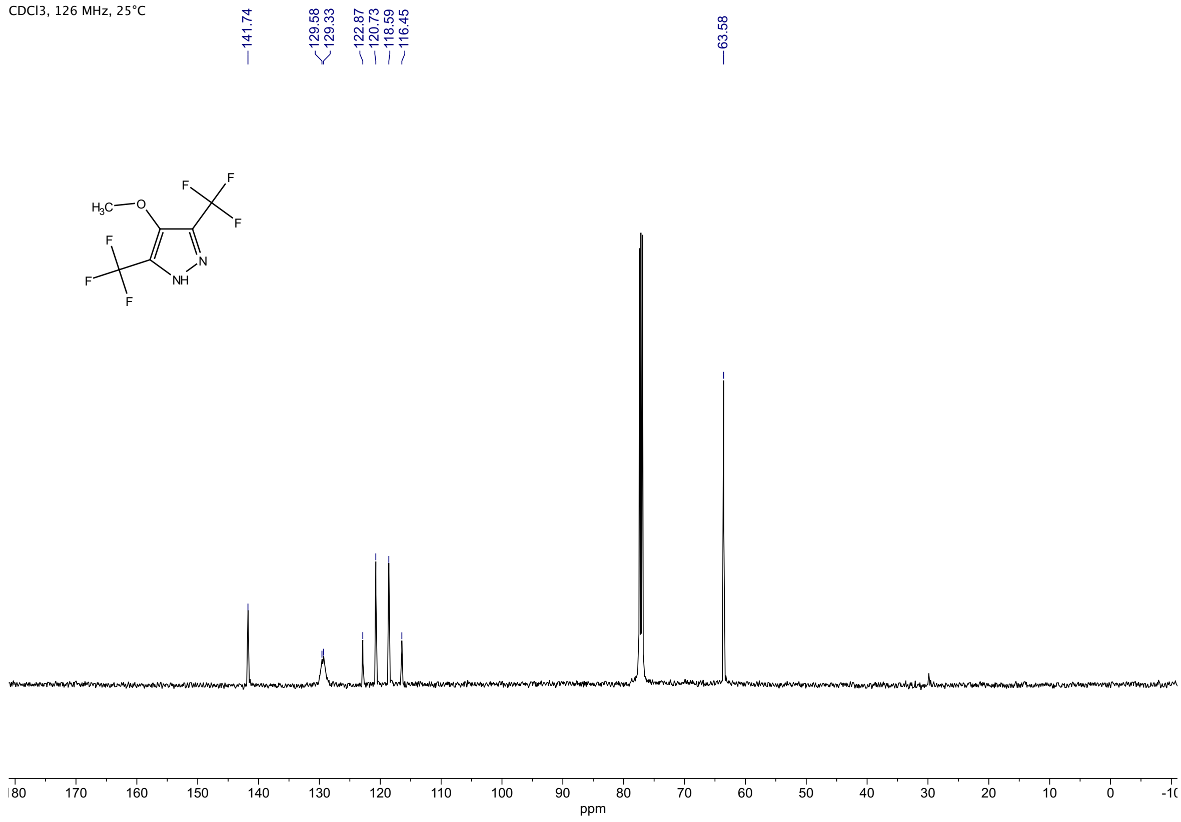

^{19}F NMR of 4-methoxy-3,5-bis(trifluoromethyl)-*1H*-pyrazole (**4a-diCF$_3$**)

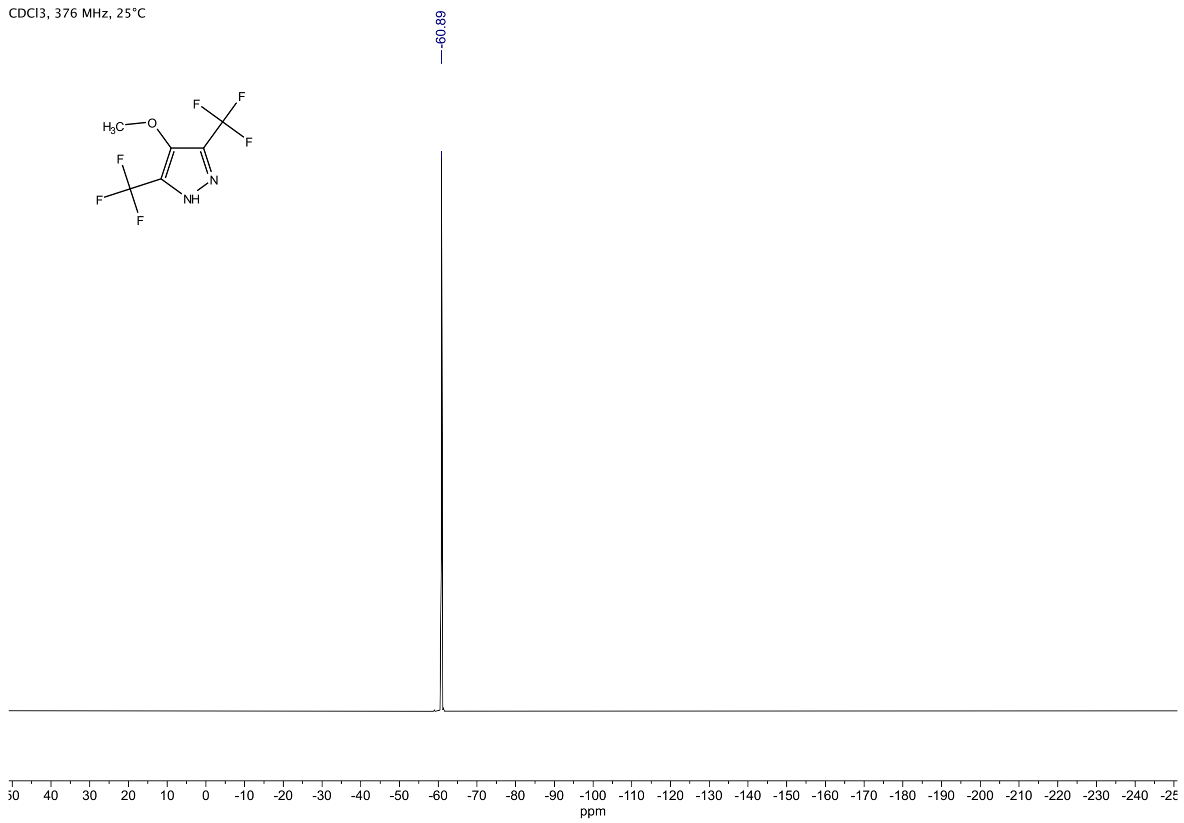

^{1}H NMR of 4-methyl-5-(trifluoromethyl)- *1H*-pyrazole (**4b-monoCF$_3$**)

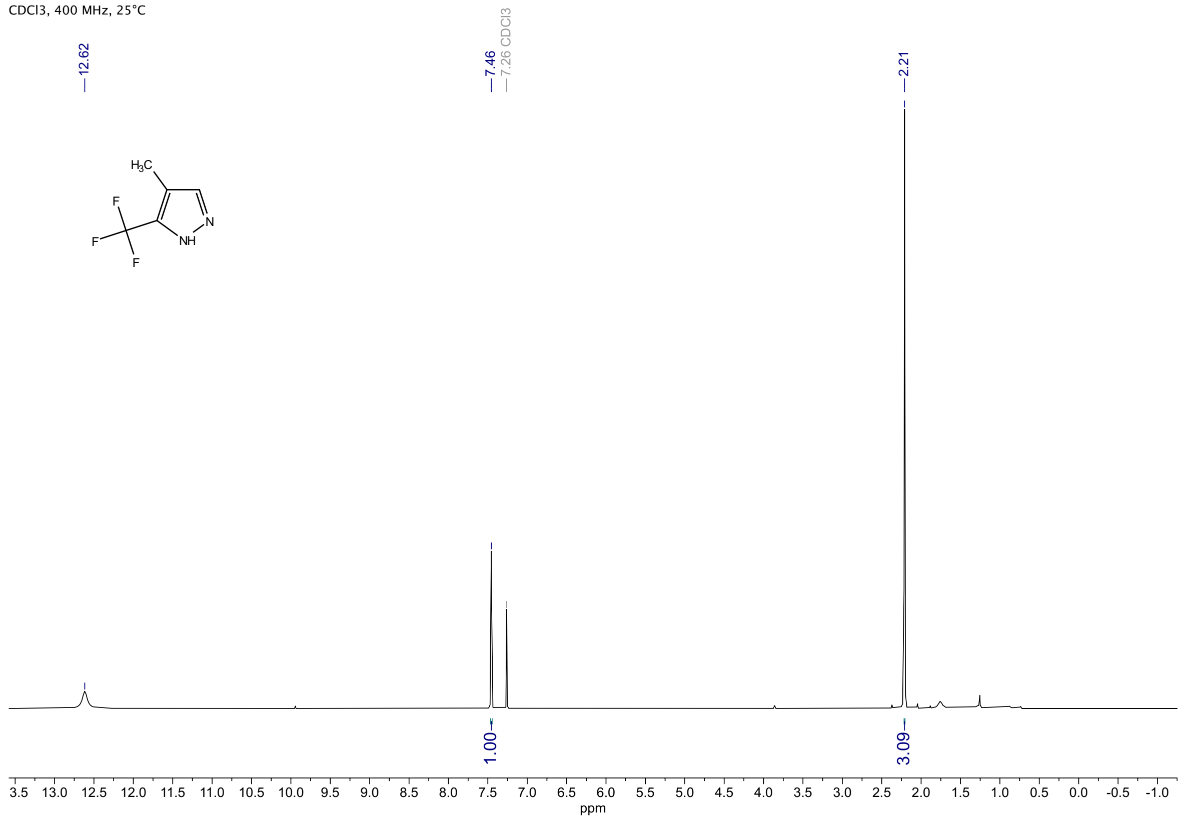

^{13}C NMR of 4-methyl-5-(trifluoromethyl)- *1H*-pyrazole (**4b-monoCF_3**)

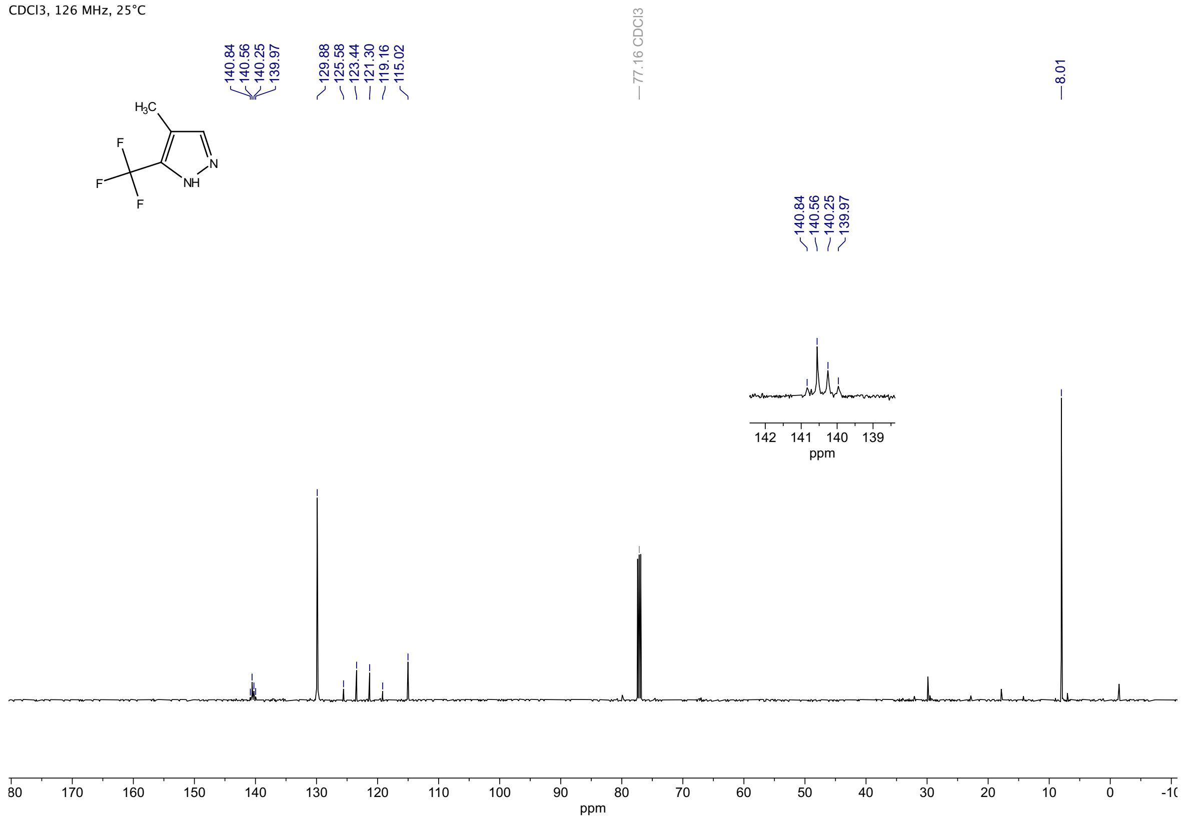

^{19}F NMR of 4-methyl-5-(trifluoromethyl)- *1H*-pyrazole (**4b-monoCF_3**)

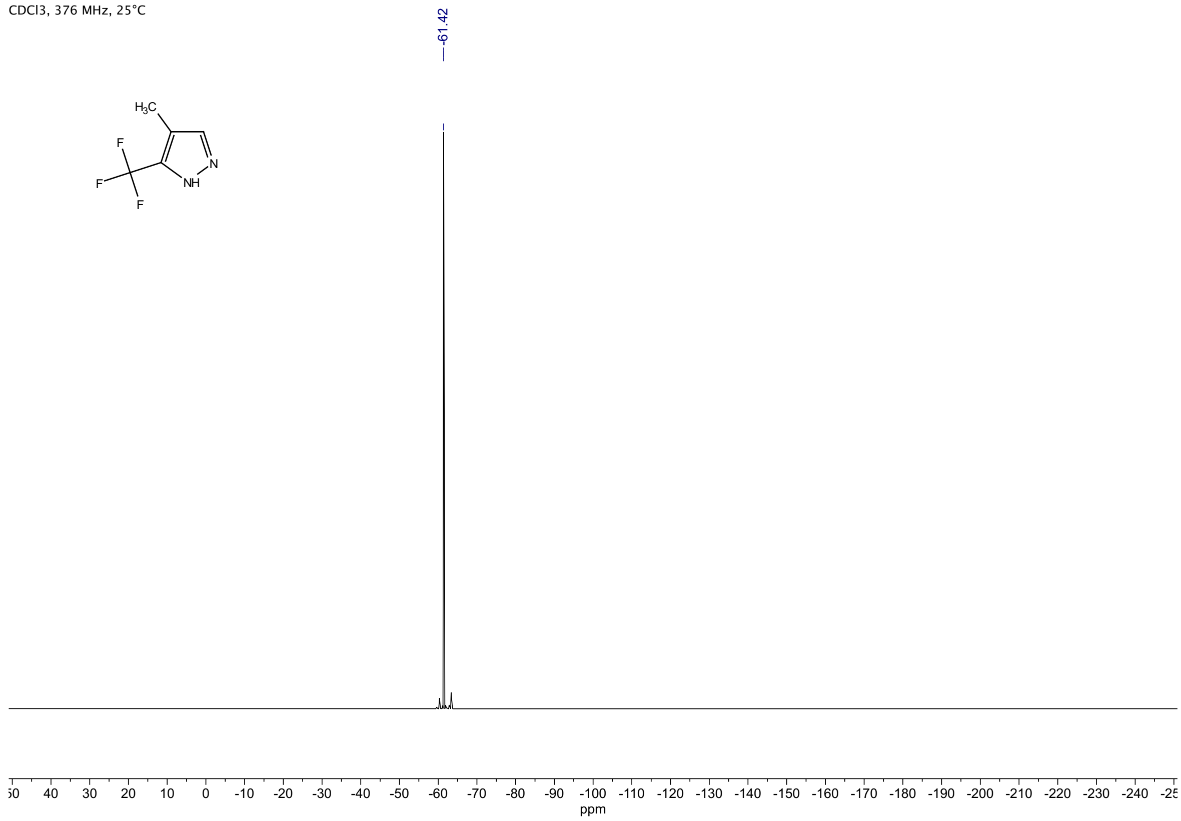

^{1}H NMR of 4-methyl-3,5-bis(trifluoromethyl)-*1H*-pyrazole (**4b-diCF$_3$**)

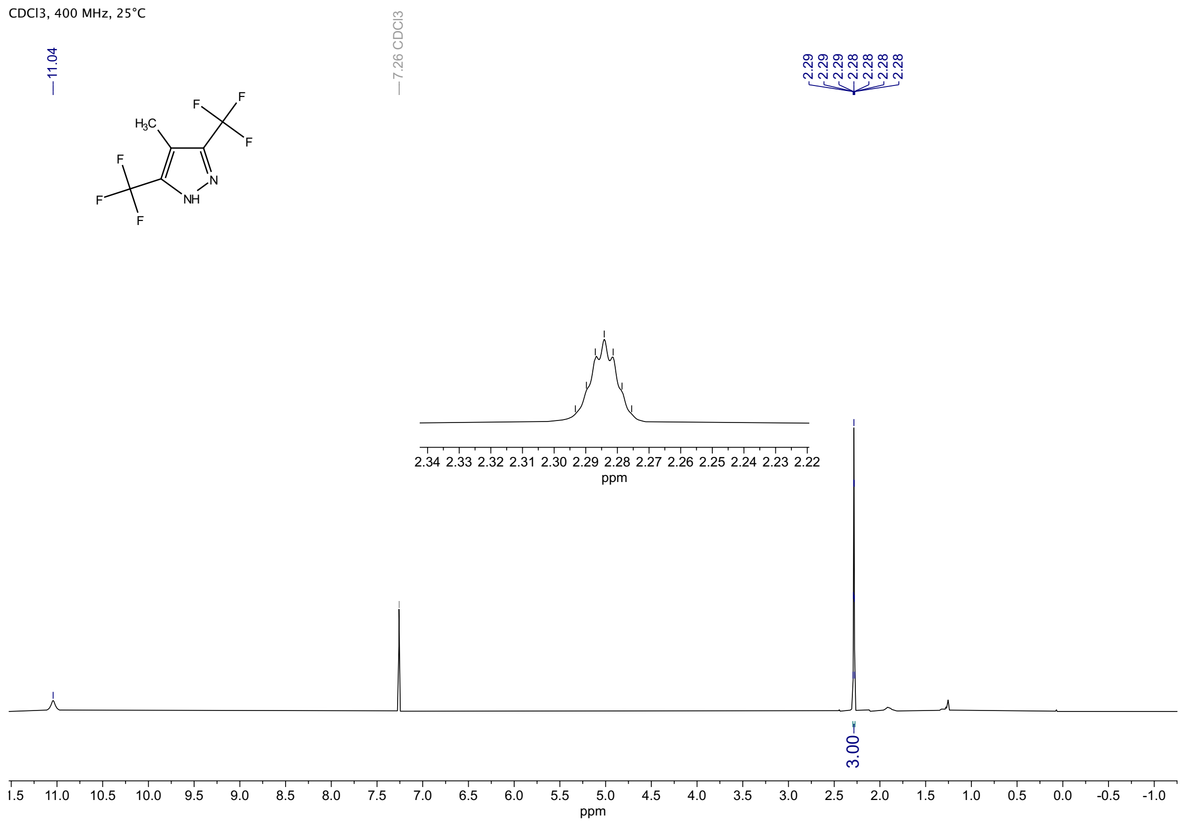

^{13}C NMR of 4-methyl-3,5-bis(trifluoromethyl)-*1H*-pyrazole (**4b-diCF$_3$**)

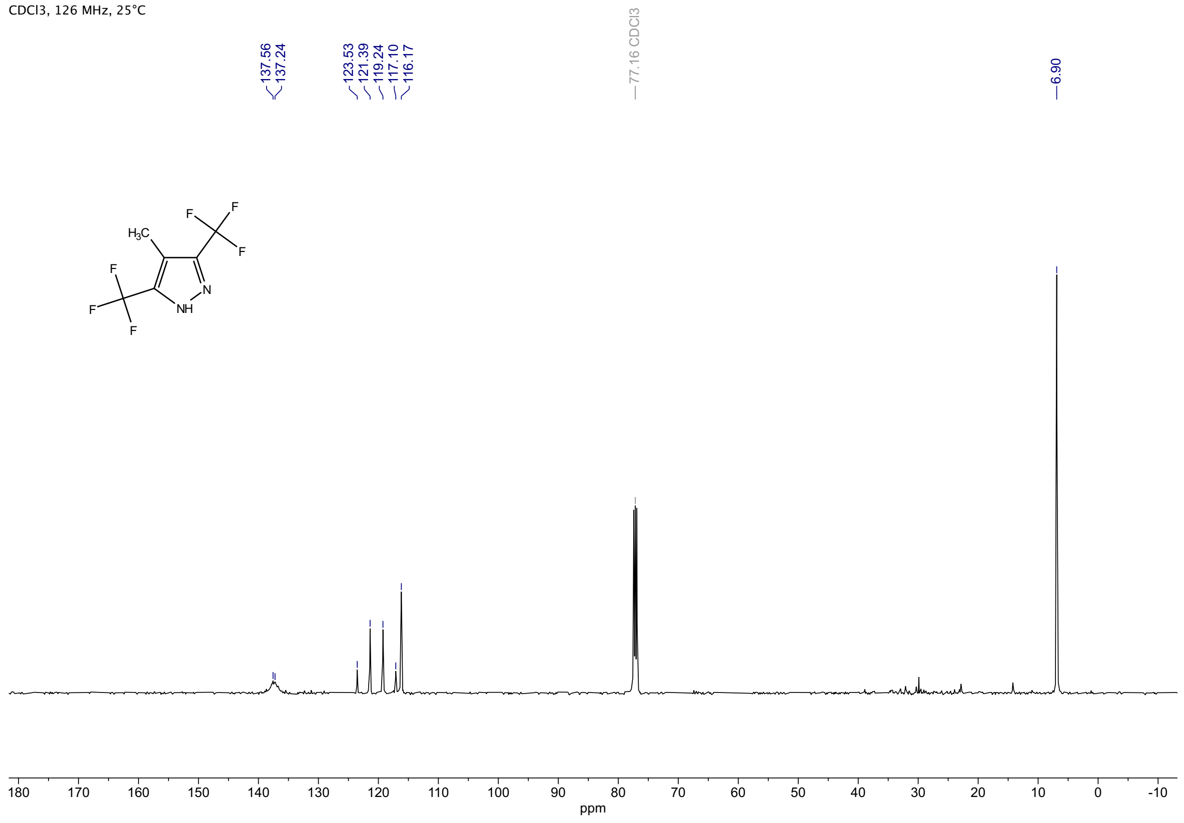

^{19}F NMR of 4-methyl-3,5-bis(trifluoromethyl)-*1H*-pyrazole (**4b-diCF$_3$**)

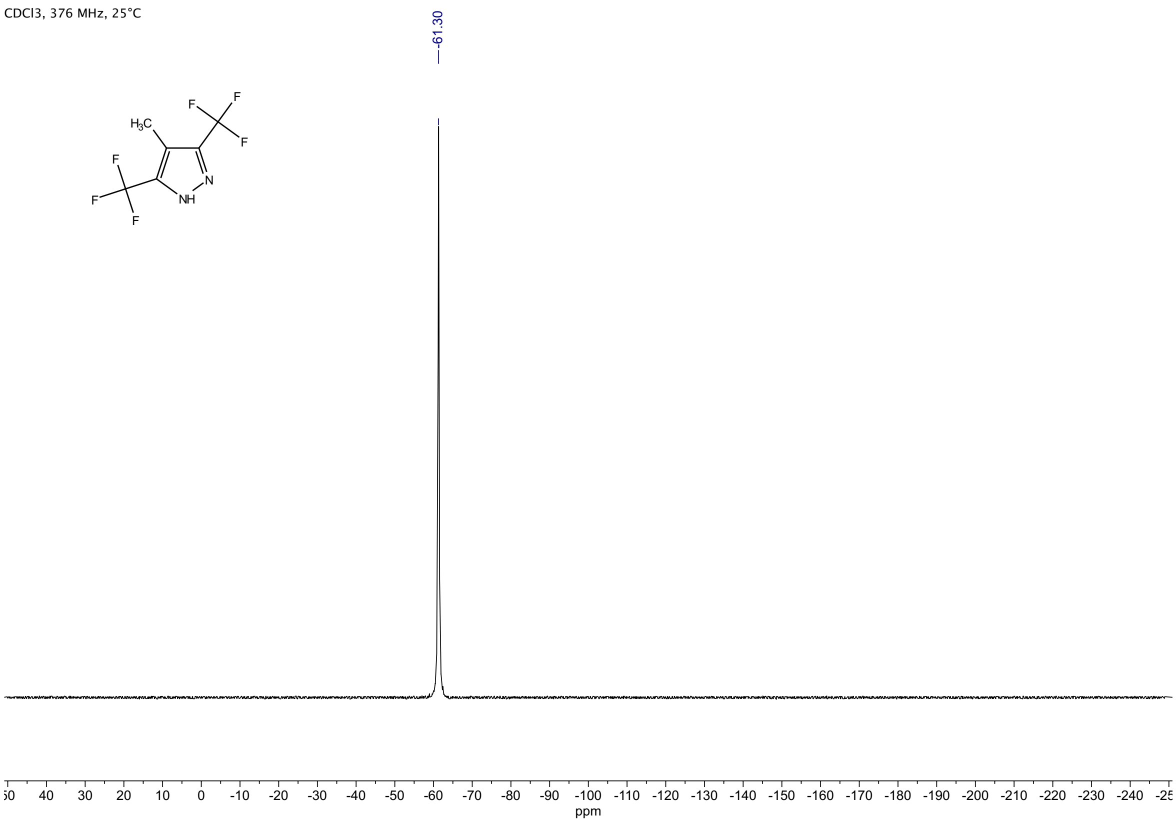

^{1}H NMR of methyl 3-(trifluoromethyl)-*1H*-pyrazole-4-carboxylate (**5c-monoCF$_3$**)

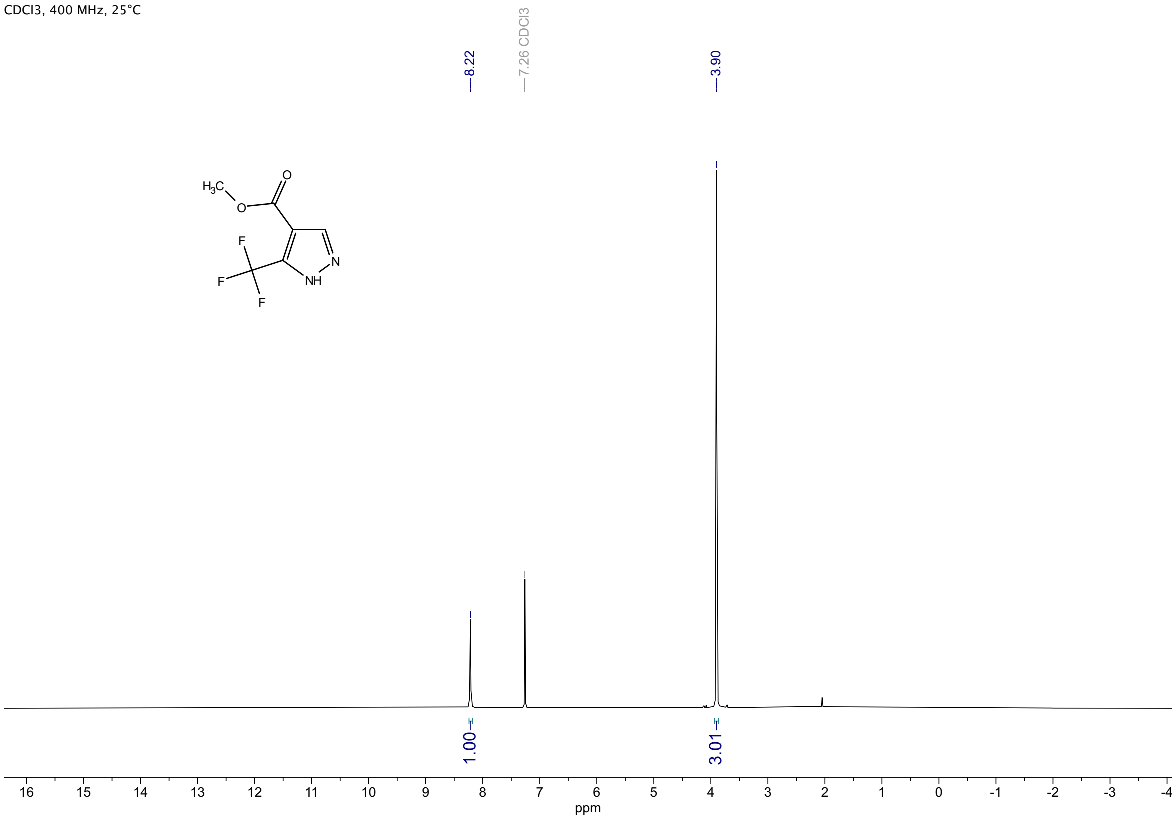

^{13}C NMR of methyl 3-(trifluoromethyl)-*1H*-pyrazole-4-carboxylate (**5c-monoCF3**)

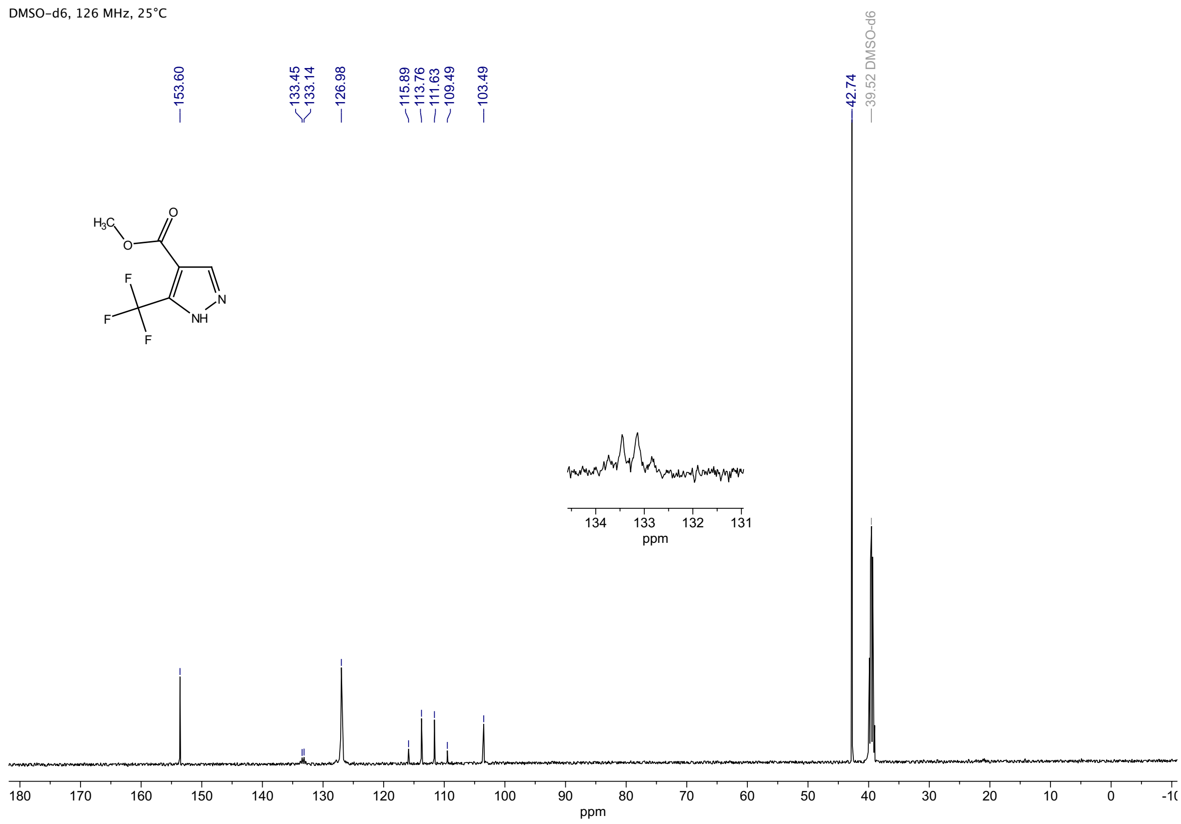

^{19}F NMR of methyl 3-(trifluoromethyl)-*1H*-pyrazole-4-carboxylate (**5c-monoCF3**)

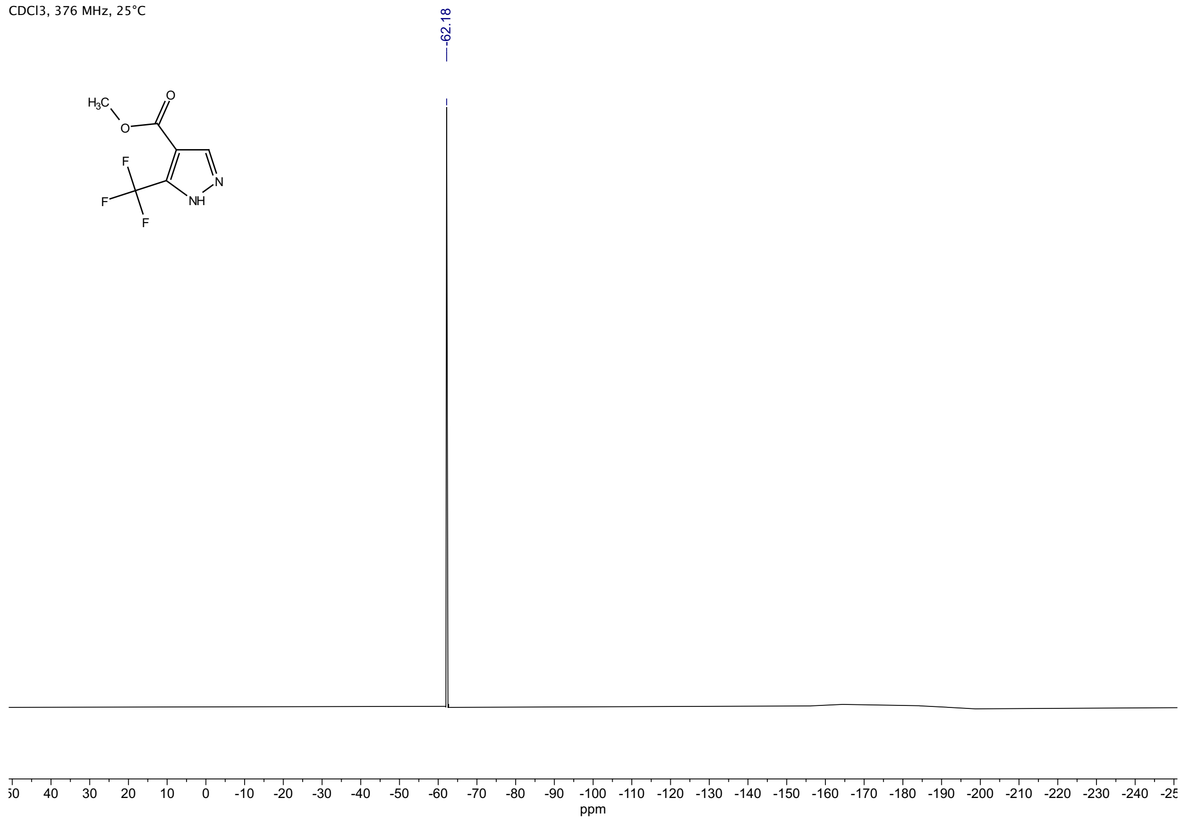

^{1}H NMR of 1-(5-(trifluoromethyl)-*1H*-pyrazol-4-yl)ethan-1-one (**5d-monoCF$_3$**)

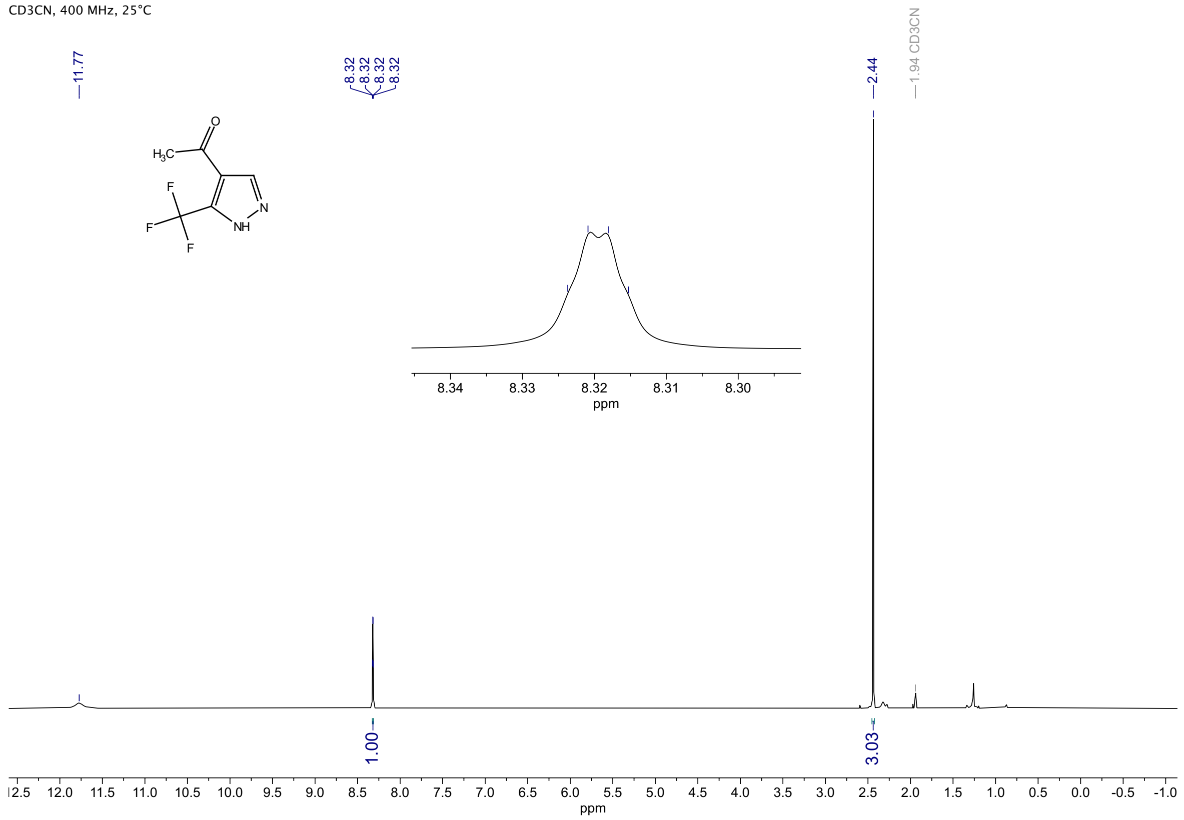

^{13}C NMR of 1-(5-(trifluoromethyl)-*1H*-pyrazol-4-yl)ethan-1-one (**5d-monoCF$_3$**)

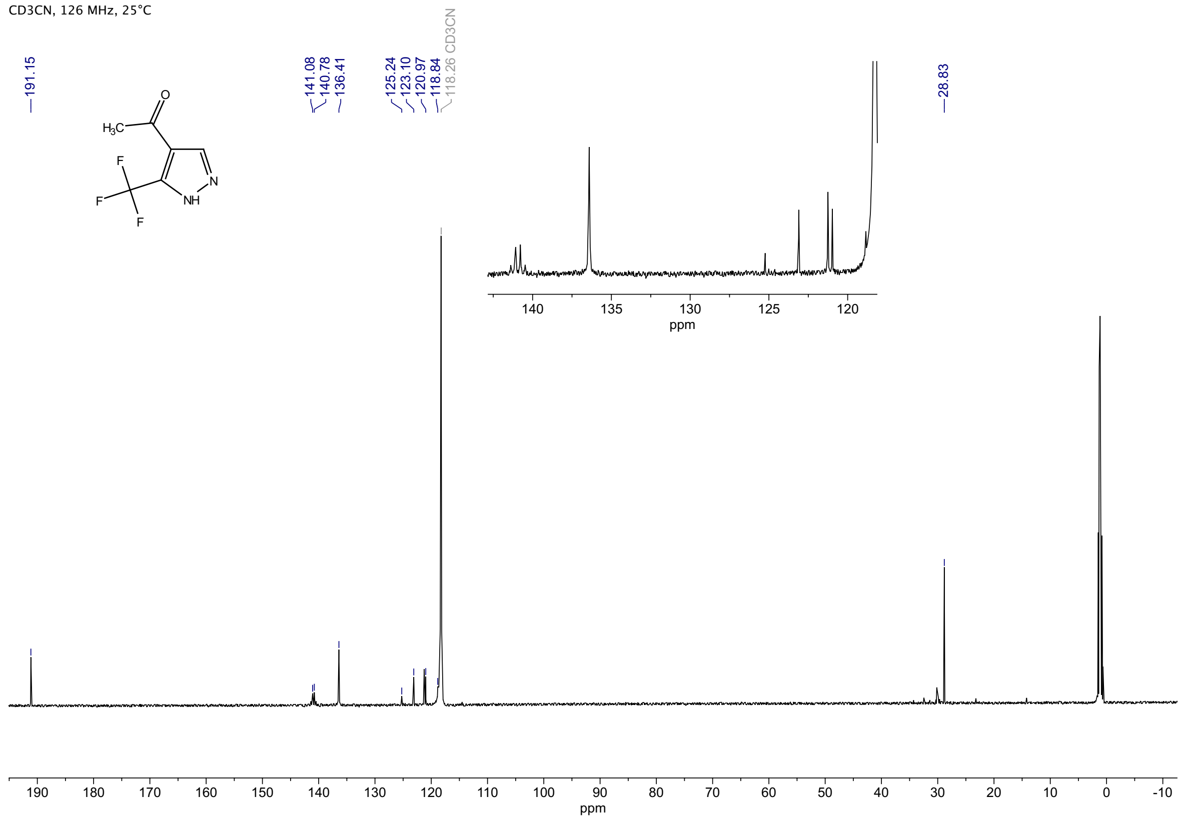

^{19}F NMR of 1-(5-(trifluoromethyl)-*1H*-pyrazol-4-yl)ethan-1-one (**5d-monoCF_3**)

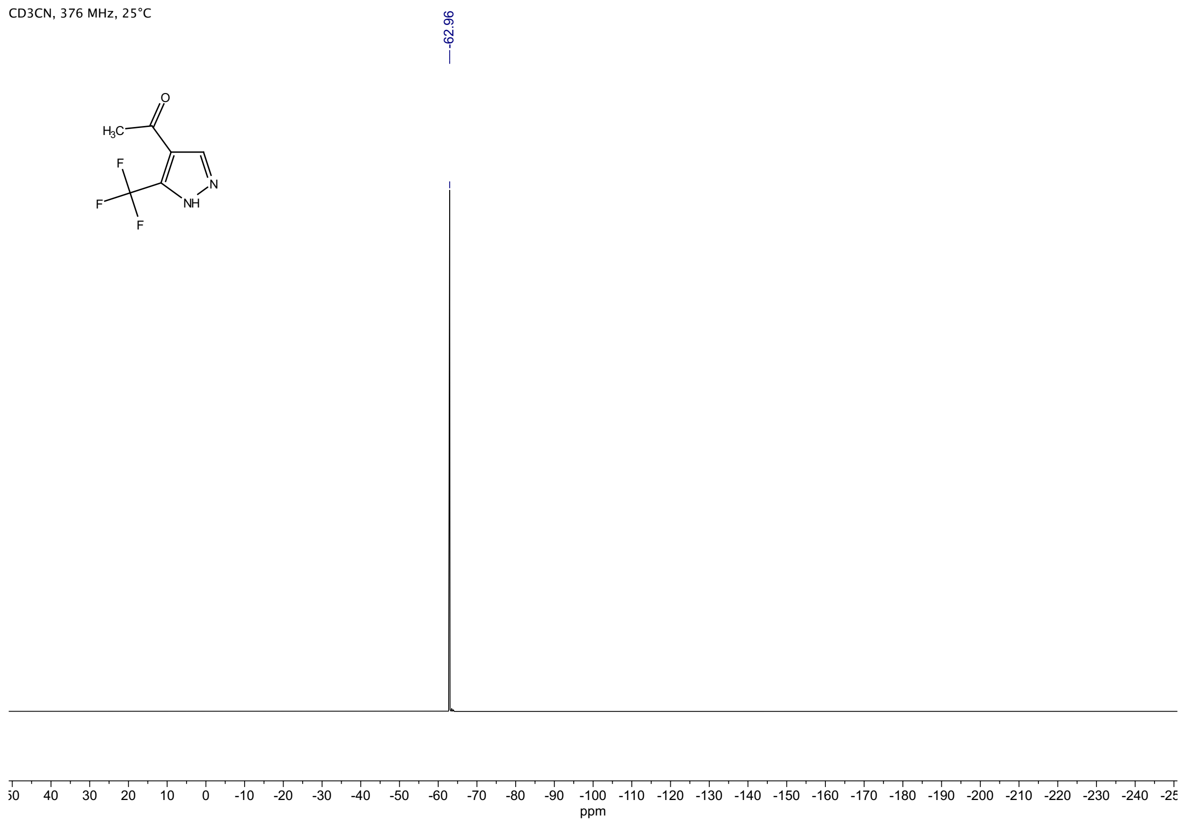

^{1}H NMR of 1-(3,5-bis(trifluoromethyl)-*1H*-pyrazol-4-yl)ethan-1-one (**5d-diCF_3**)

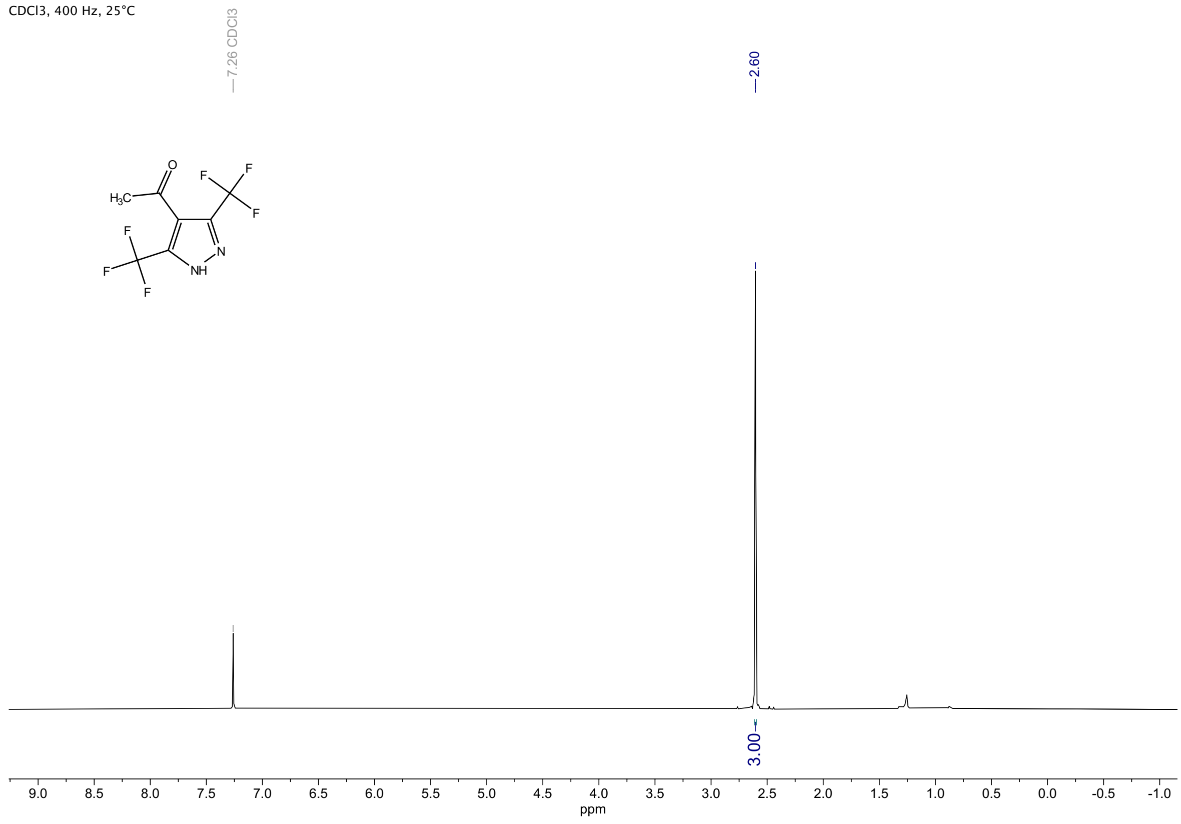

^{13}C NMR of 1-(3,5-bis(trifluoromethyl)-*1H*-pyrazol-4-yl)ethan-1-one (**5d-diCF$_3$**)

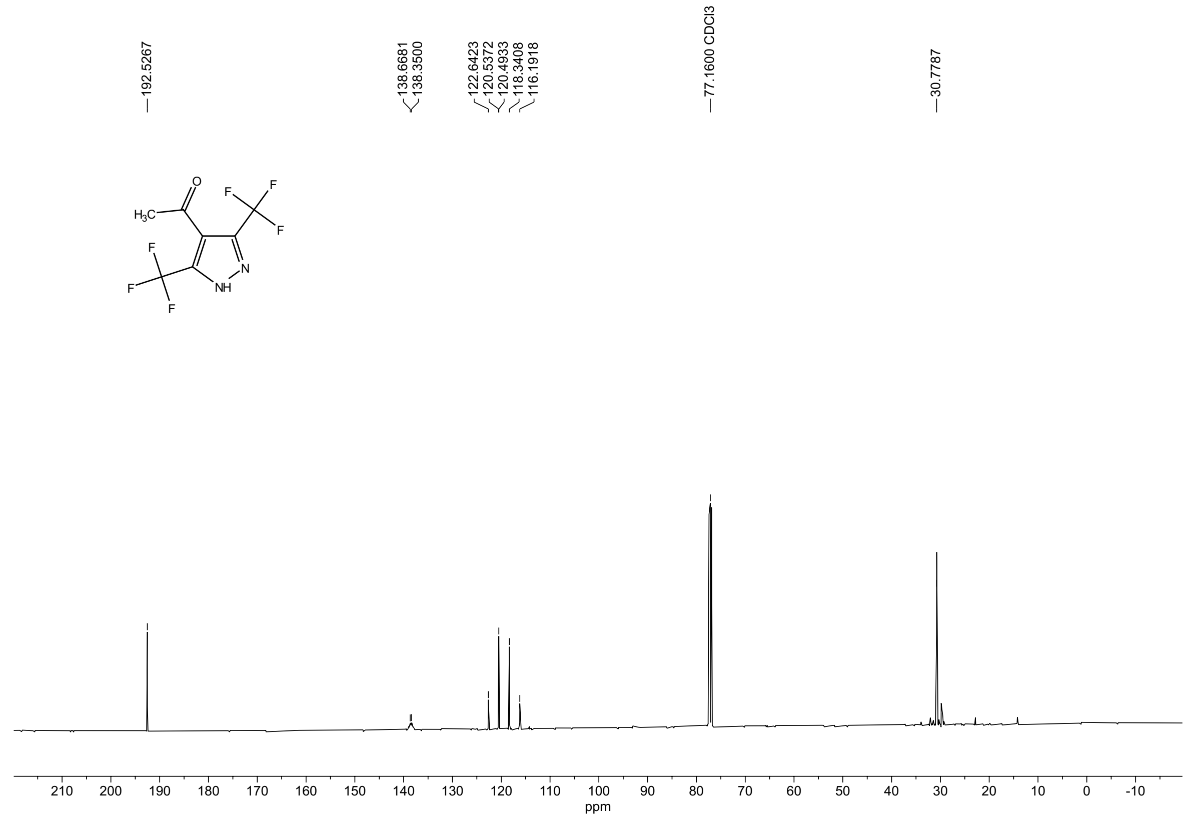

^{19}F NMR of 1-(3,5-bis(trifluoromethyl)-*1H*-pyrazol-4-yl)ethan-1-one (**5d-diCF$_3$**)

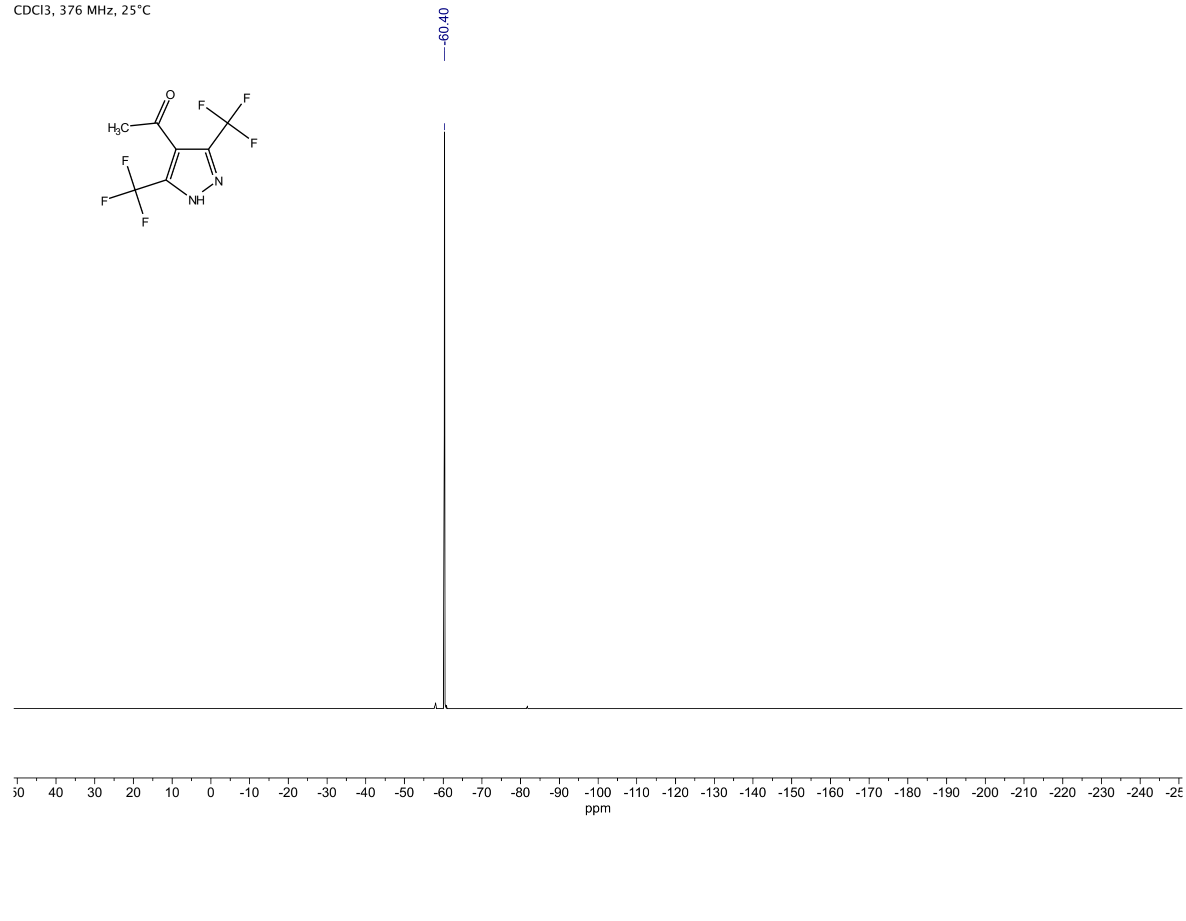

^{1}H NMR of 4-nitro-3-(trifluoromethyl)-1H-pyrazole (**5e-monoCF$_3$**)

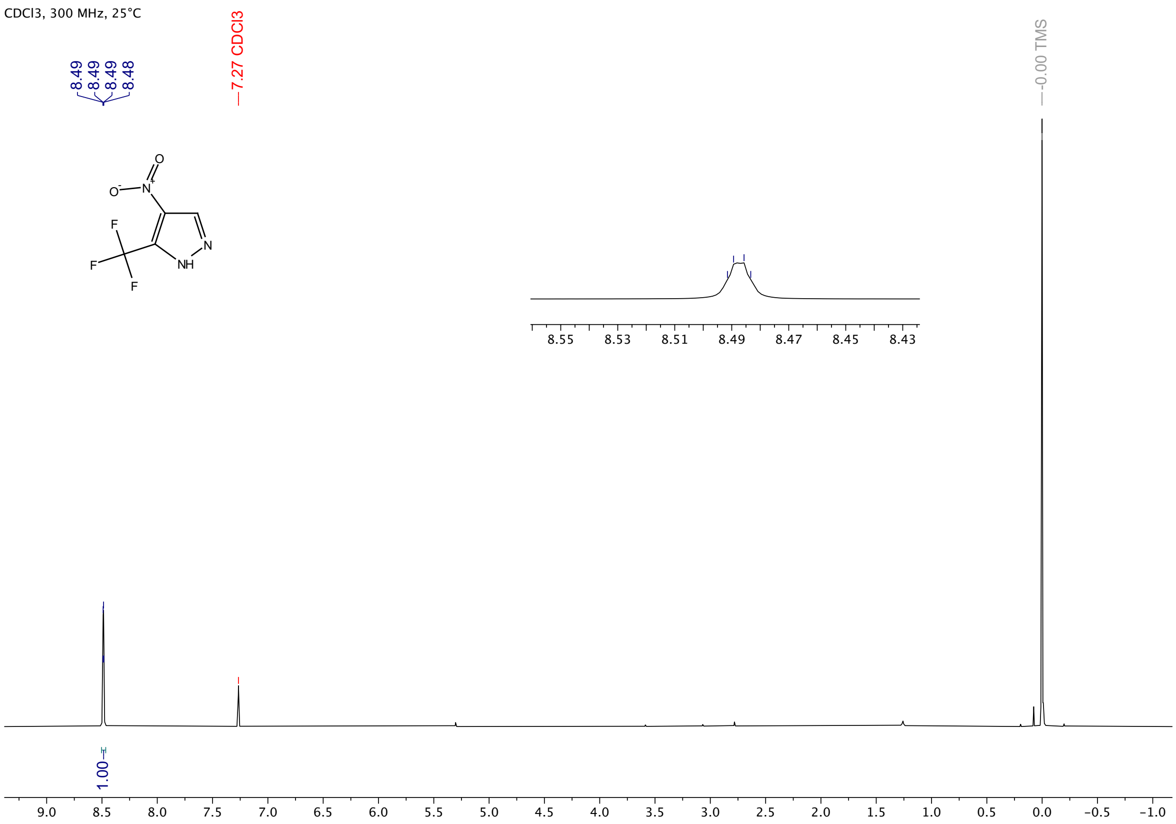

^{13}C NMR of 4-nitro-3-(trifluoromethyl)-1H-pyrazole (**5e-monoCF$_3$**)

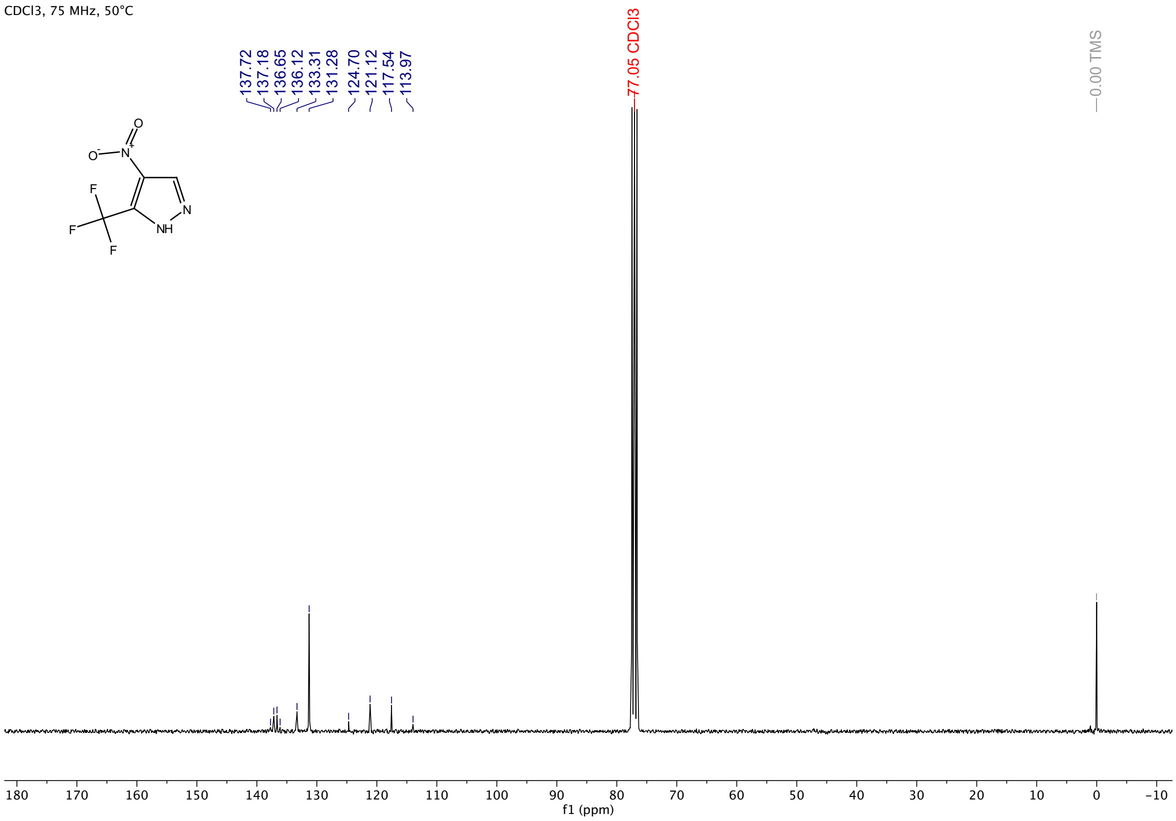

^{19}F NMR of 4-nitro-3-(trifluoromethyl)-1H-pyrazole (**5e-monoCF$_3$**)

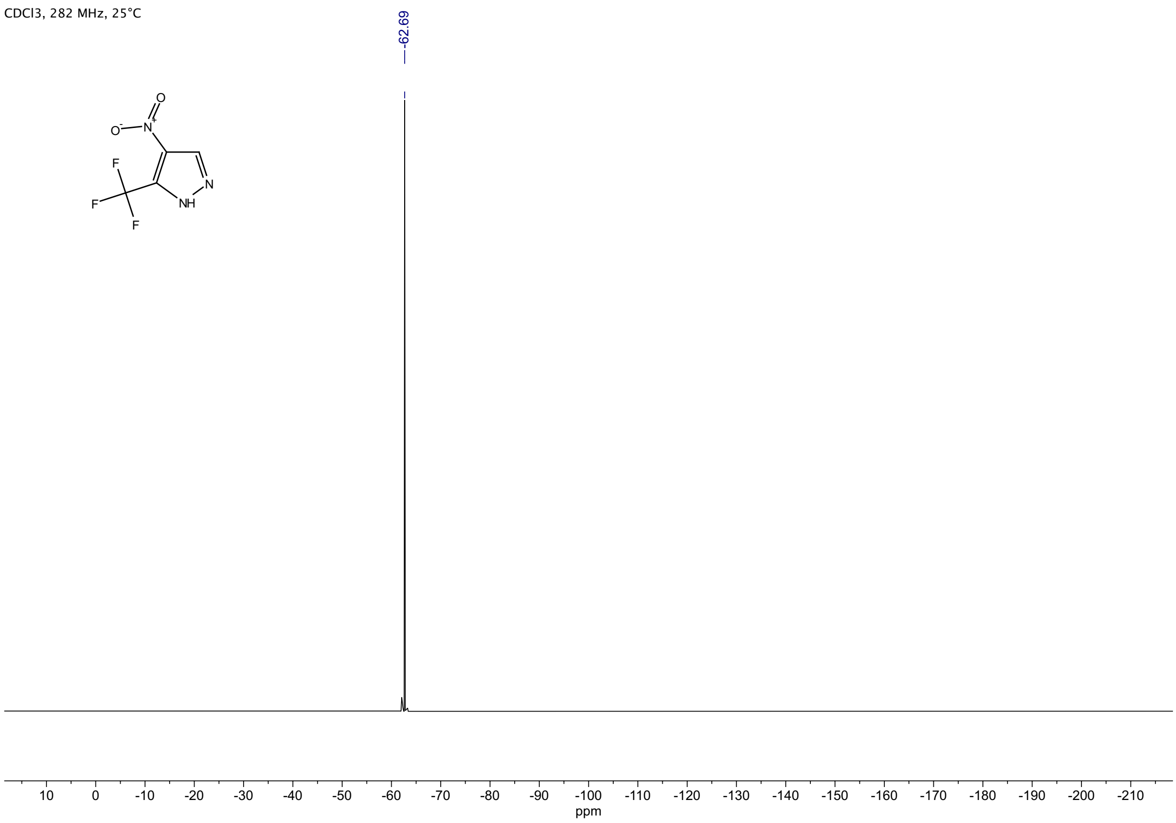

^{1}H NMR of 3-(trifluoromethyl)-*1H*-pyrazole-4-carboxylic acid (**5f-monoCF$_3$**)

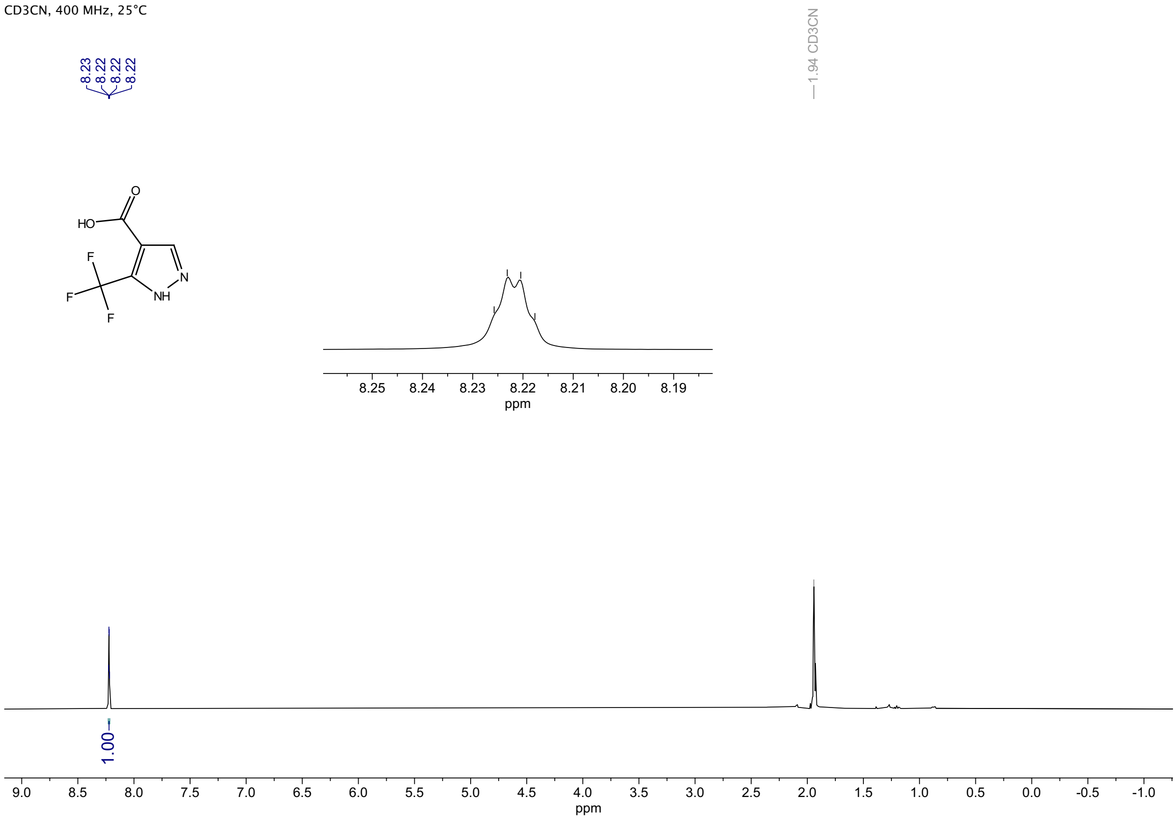

^{13}C NMR of 3-(trifluoromethyl)-*1H*-pyrazole-4-carboxylic acid (**5f-monoCF$_3$**)

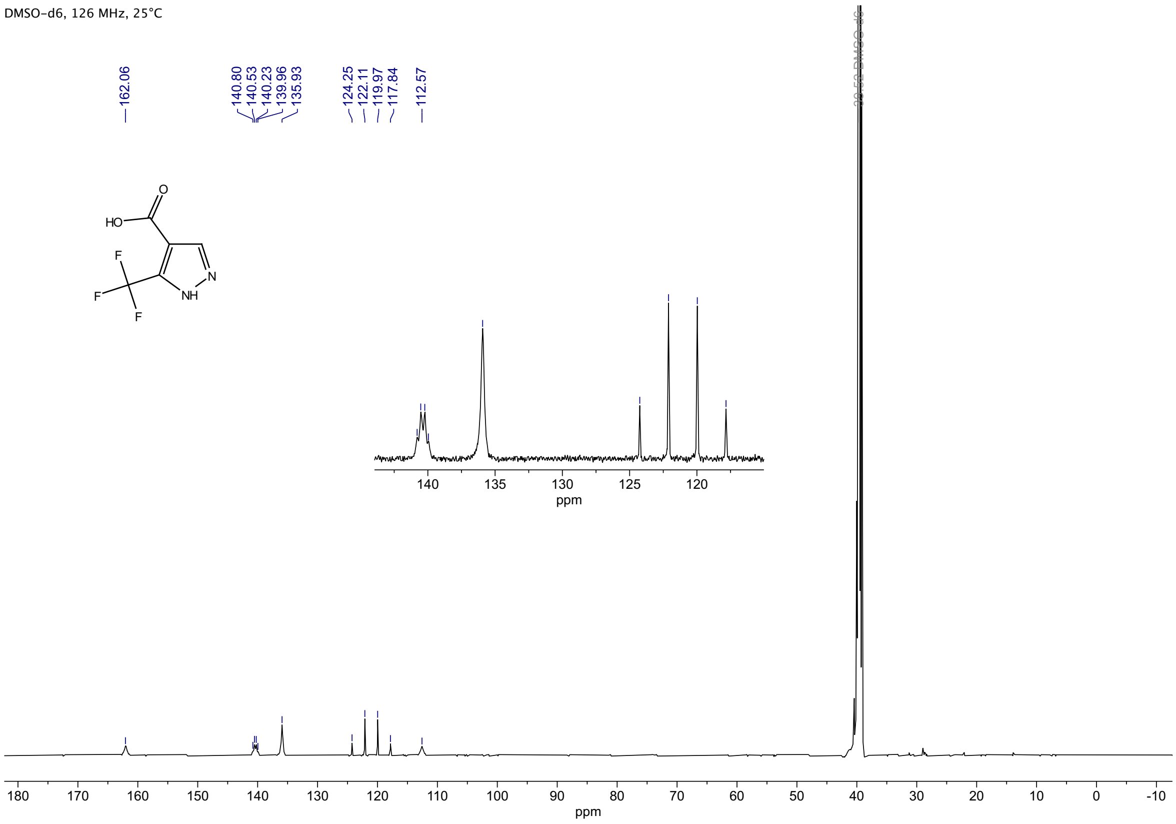

^{19}F NMR of 3-(trifluoromethyl)-*1H*-pyrazole-4-carboxylic acid (**5f-monoCF$_3$**)

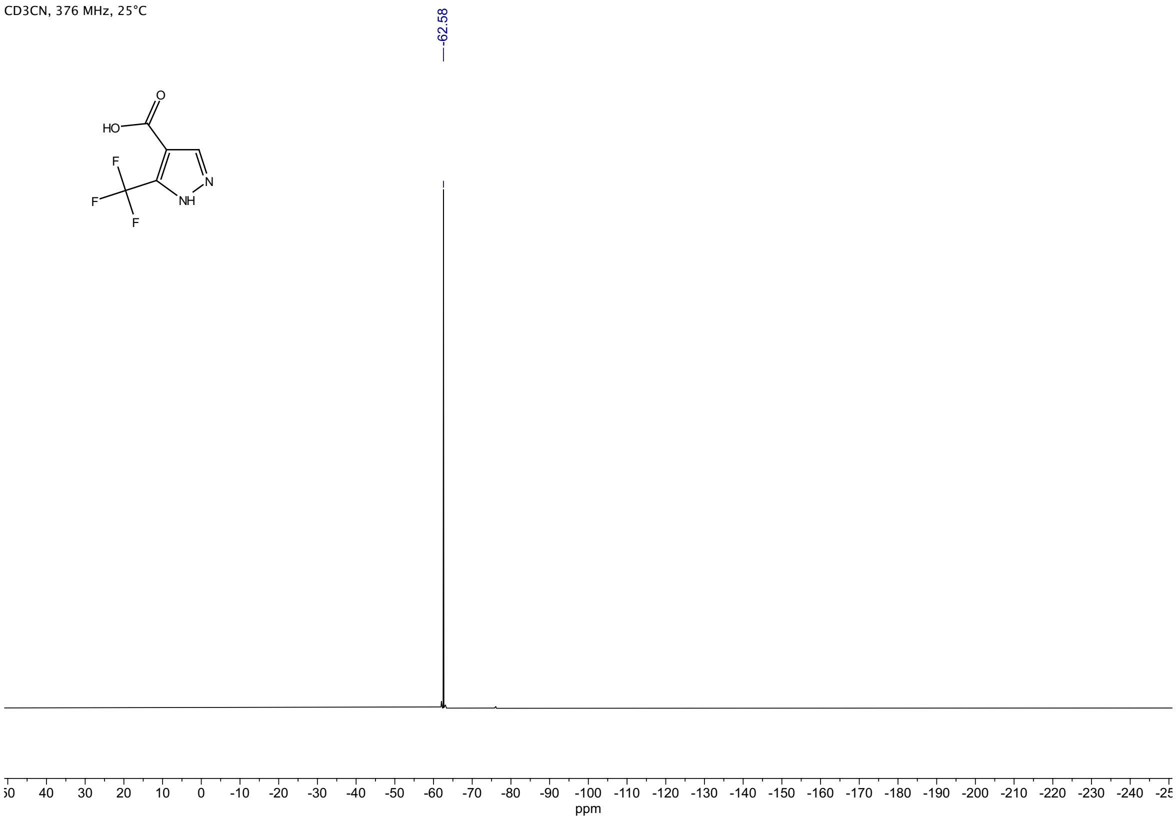

^{1}H NMR of 1-(5-(trifluoromethyl)-*1H*-pyrazol-3-yl)ethan-1-one (**5g-C5-CF$_3$**)

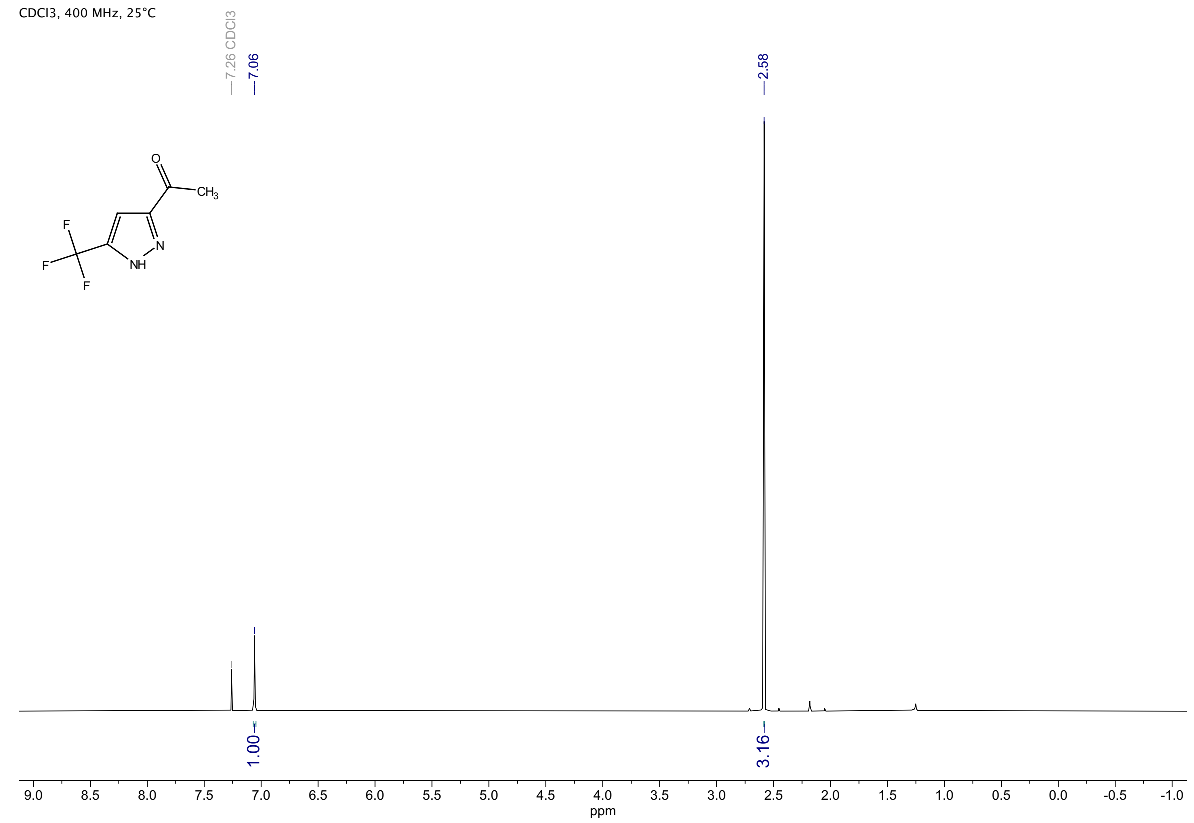

^{13}C NMR of 1-(5-(trifluoromethyl)-*1H*-pyrazol-3-yl)ethan-1-one (**5g-C5-CF$_3$**)

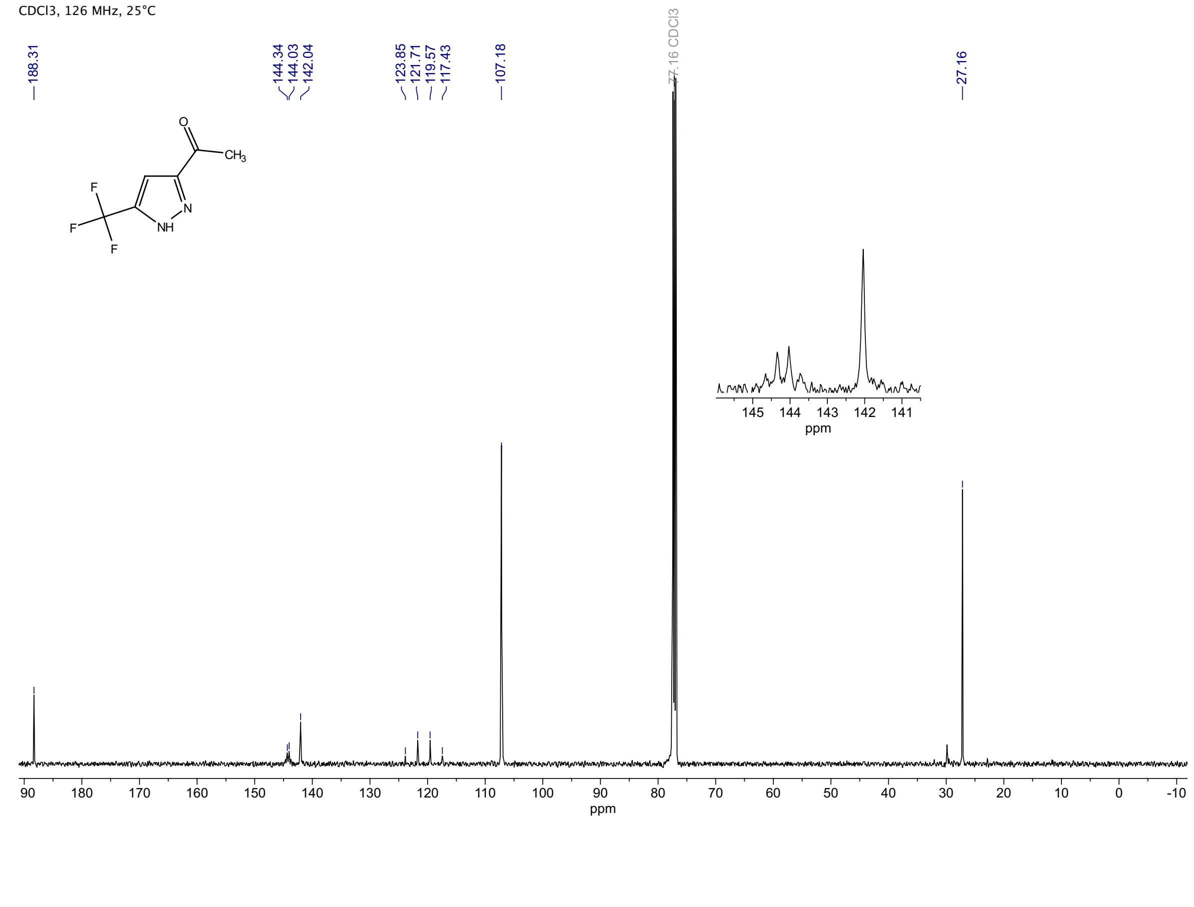

^{19}F NMR of 1-(5-(trifluoromethyl)-*1H*-pyrazol-3-yl)ethan-1-one (**5g-C5-CF**$_3$)

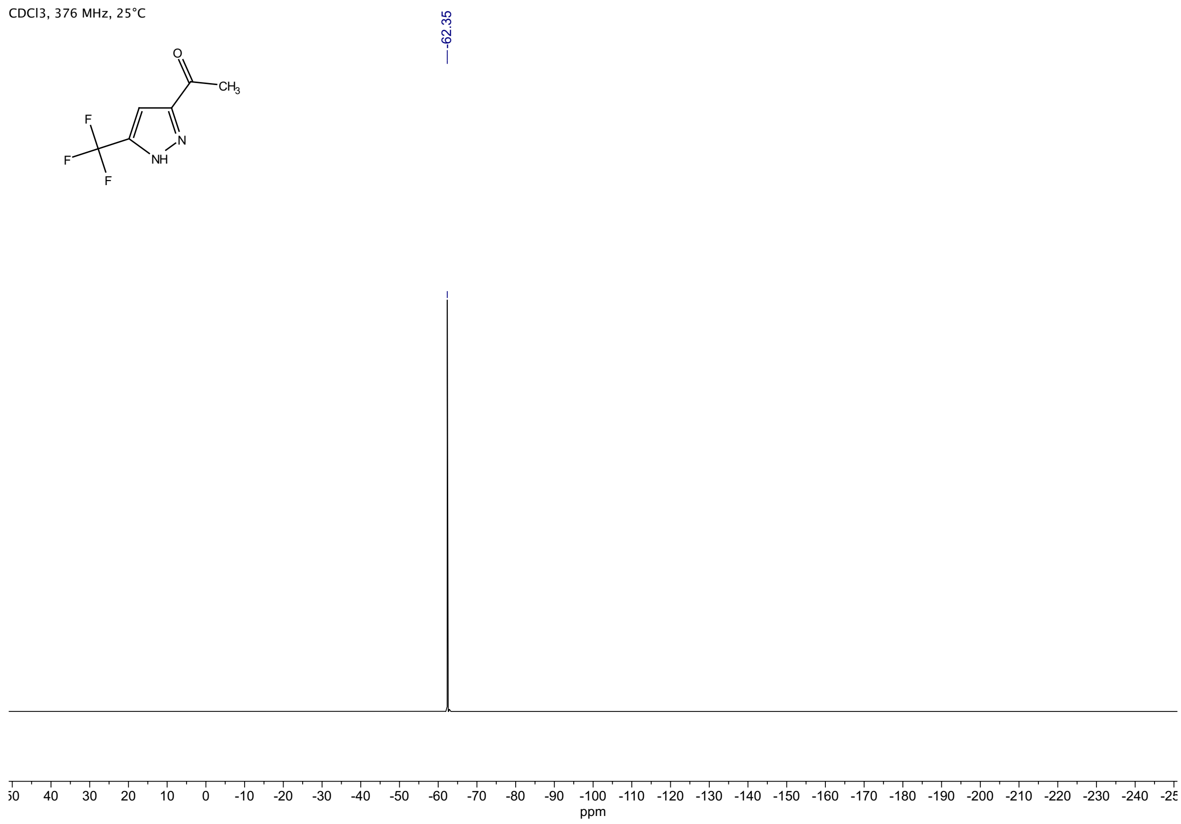

^{1}H NMR of 1-(4-(trifluoromethyl)-*1H*-pyrazol-3-yl)ethan-1-one (**5g-C4-CF**$_3$)

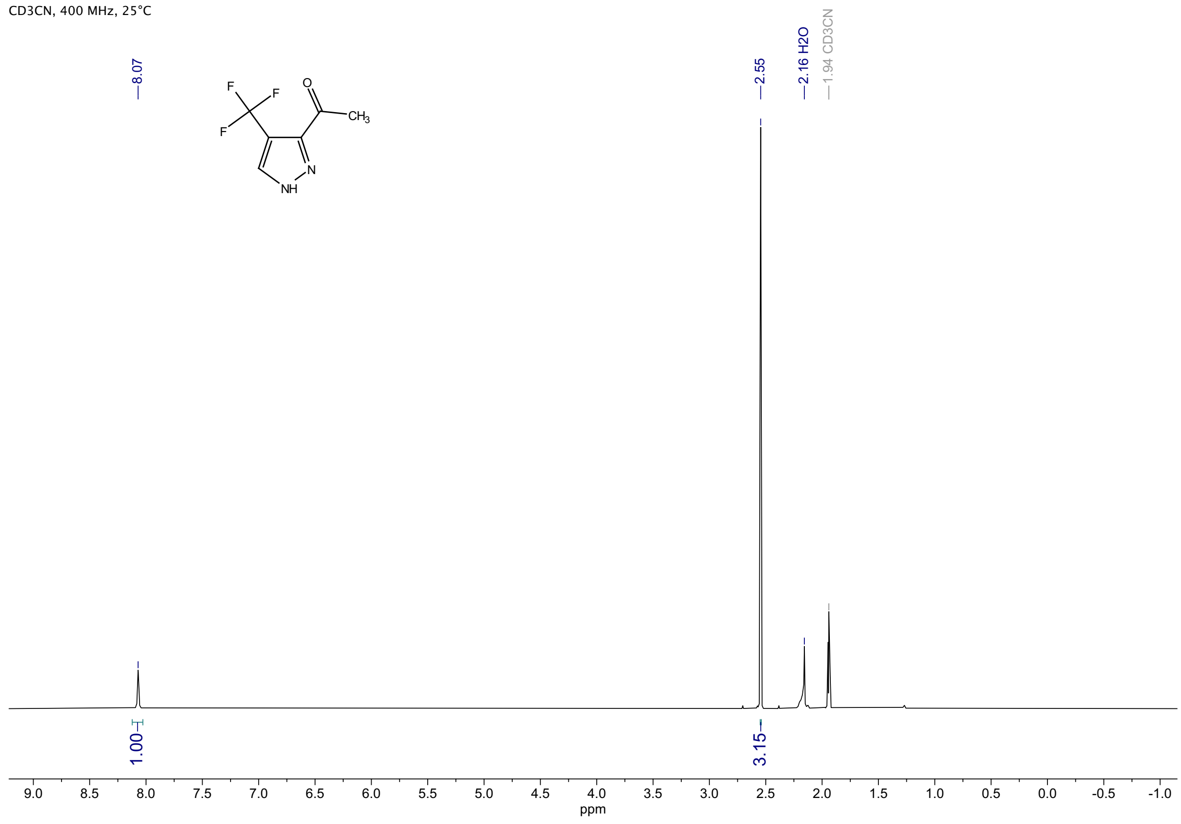

^{13}C NMR of 1-(4-(trifluoromethyl)-*1H*-pyrazol-3-yl)ethan-1-one (**5g-C4-CF$_3$**)

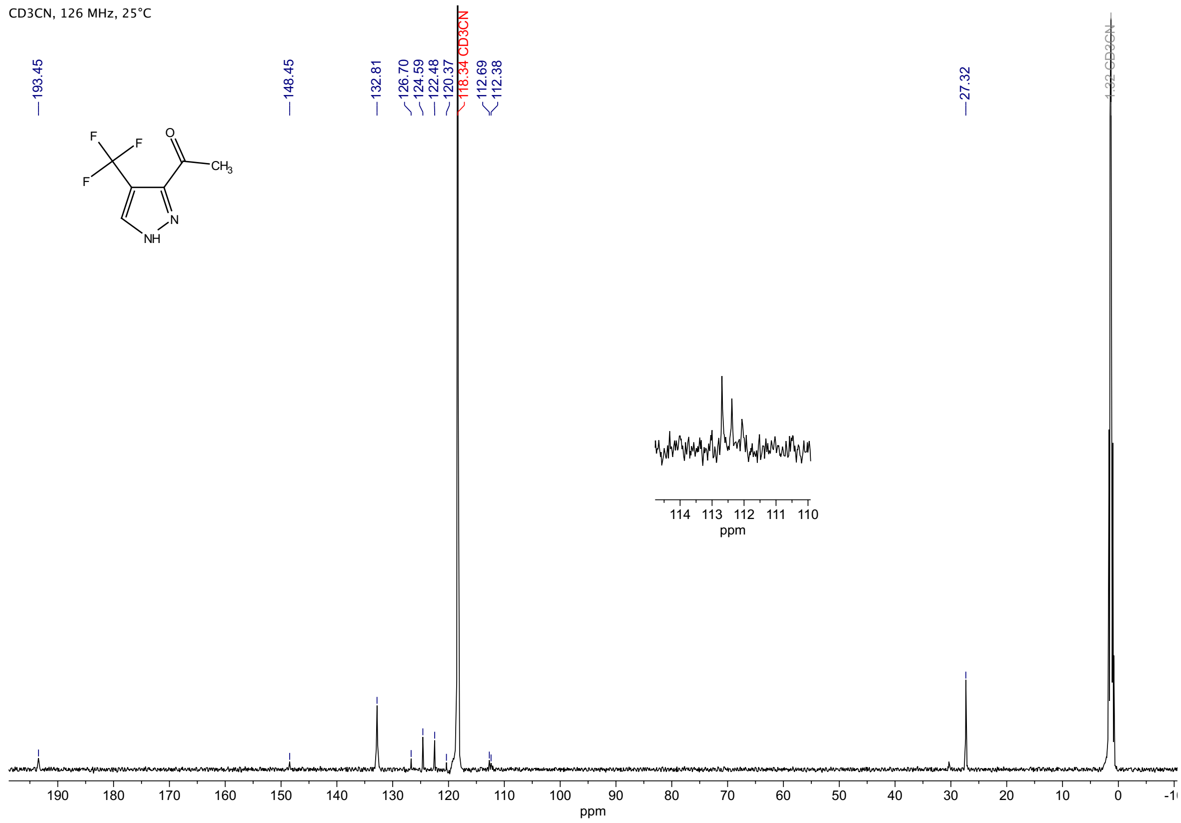

^{19}F NMR of 1-(4-(trifluoromethyl)-*1H*-pyrazol-3-yl)ethan-1-one (**5g-C4-CF$_3$**)

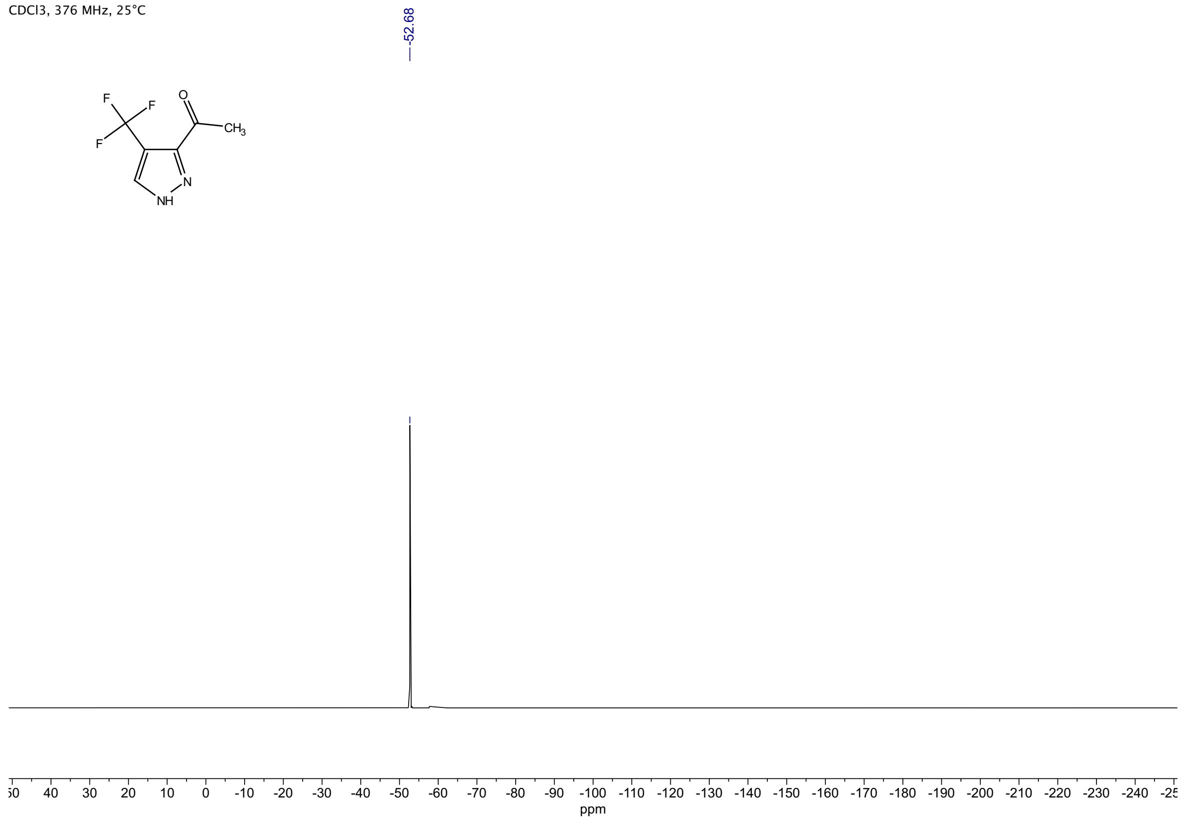

^{1}H NMR of methyl 5-(trifluoromethyl)-*1H*-pyrazole-3-carboxylate (**5h-C5-CF$_3$**)

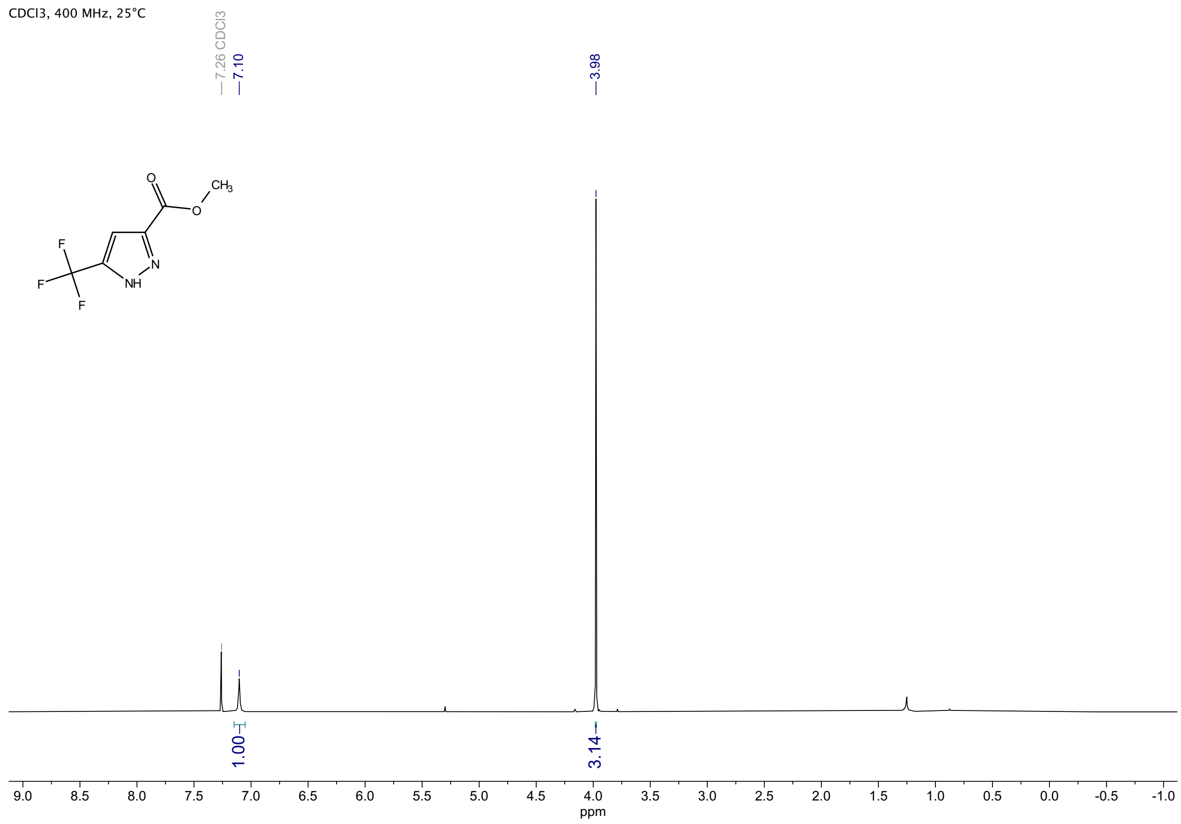

^{13}C NMR of methyl 5-(trifluoromethyl)-*1H*-pyrazole-3-carboxylate (**5h-C5-CF$_3$**)

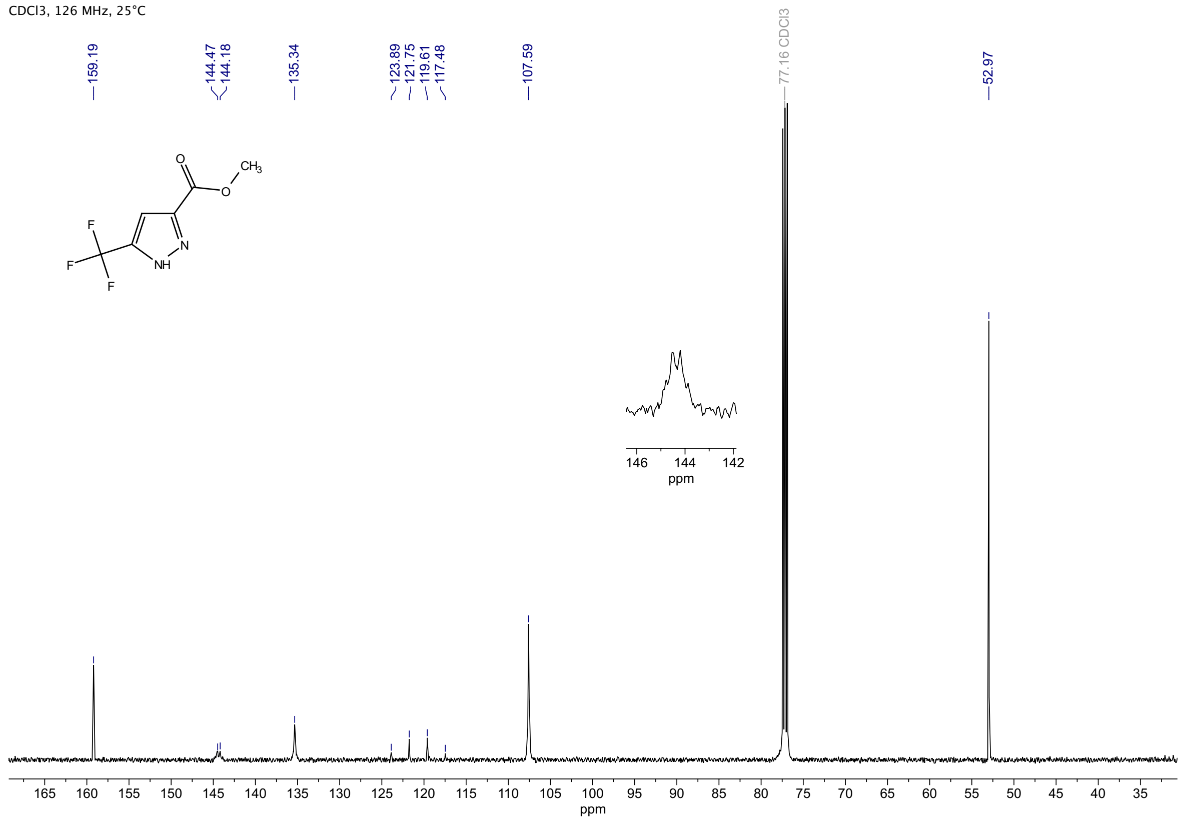

^{19}F NMR of methyl 5-(trifluoromethyl)-*1H*-pyrazole-3-carboxylate (**5h-C5-CF$_3$**)

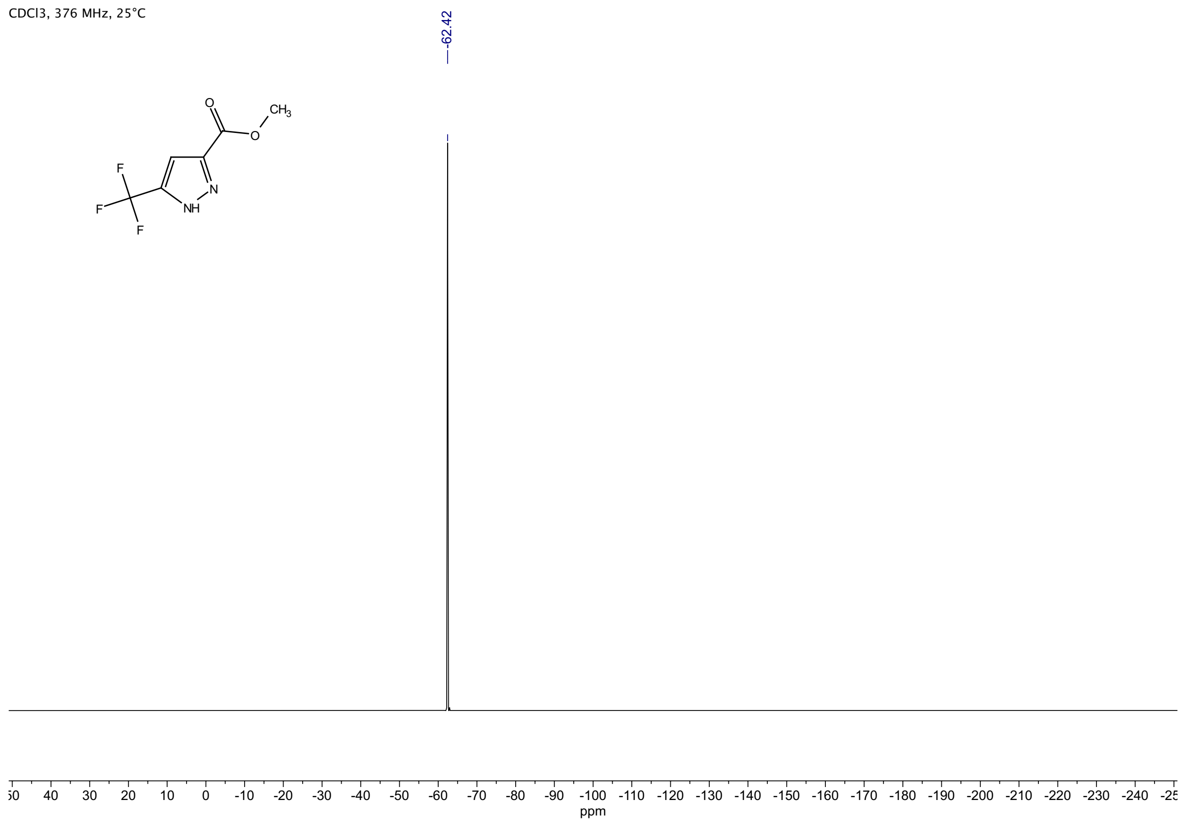

^{1}H NMR of methyl 4-(trifluoromethyl)-*1H*-pyrazole-3-carboxylate (**5h-C4-CF$_3$**)

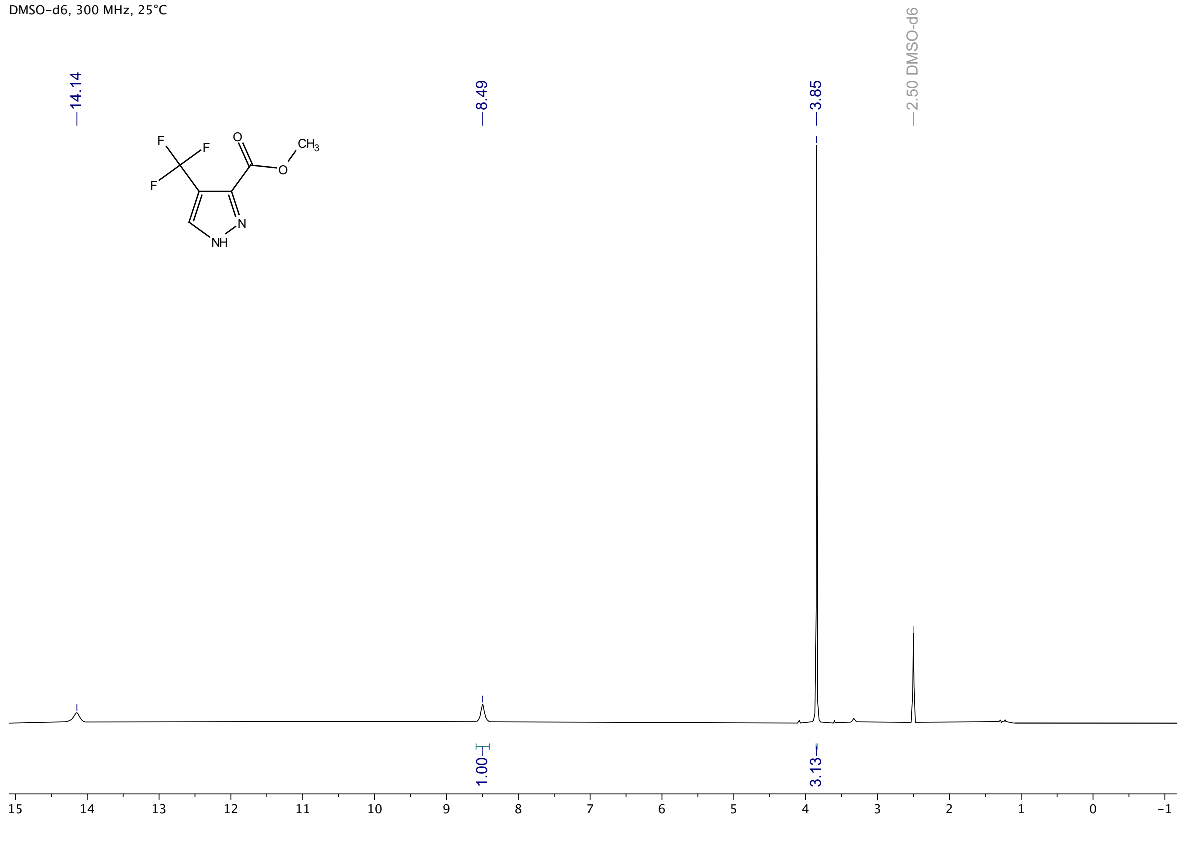

^{13}C NMR of methyl 4-(trifluoromethyl)-*1H*-pyrazole-3-carboxylate (**5h-C4-CF$_3$**)

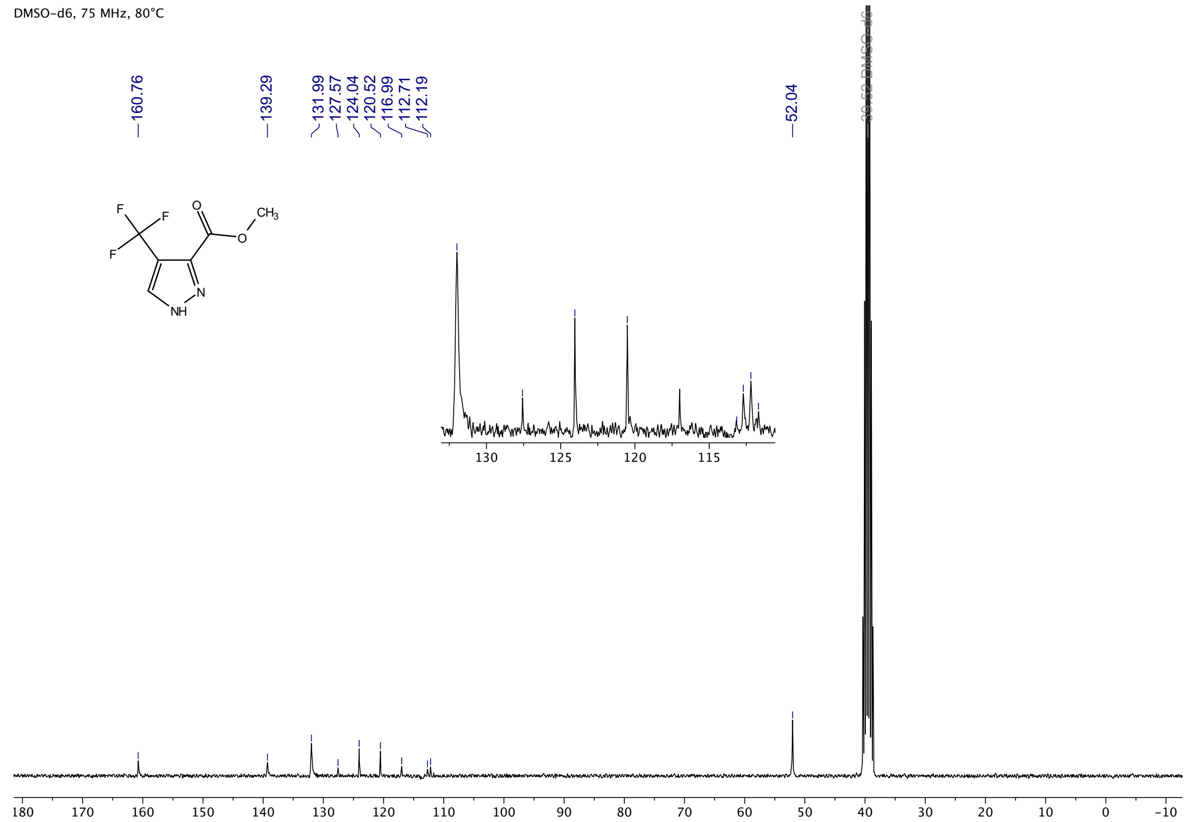

^{19}F NMR of methyl 4-(trifluoromethyl)-*1H*-pyrazole-3-carboxylate (**5h-C4-CF$_3$**)

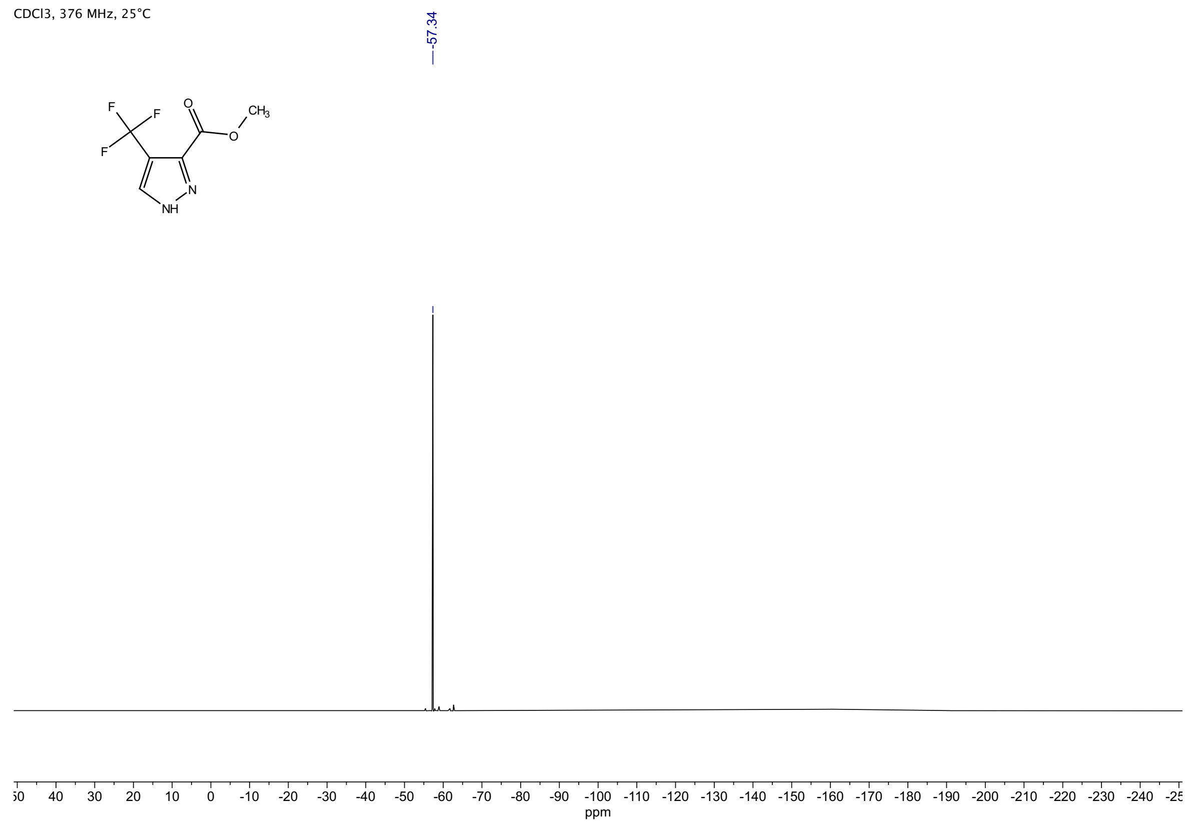

^{1}H NMR of 1-methyl-2-(5-(trifluoromethyl)-1H-pyrazol-3-yl)piperidine (**5i-C5-CF$_3$**)

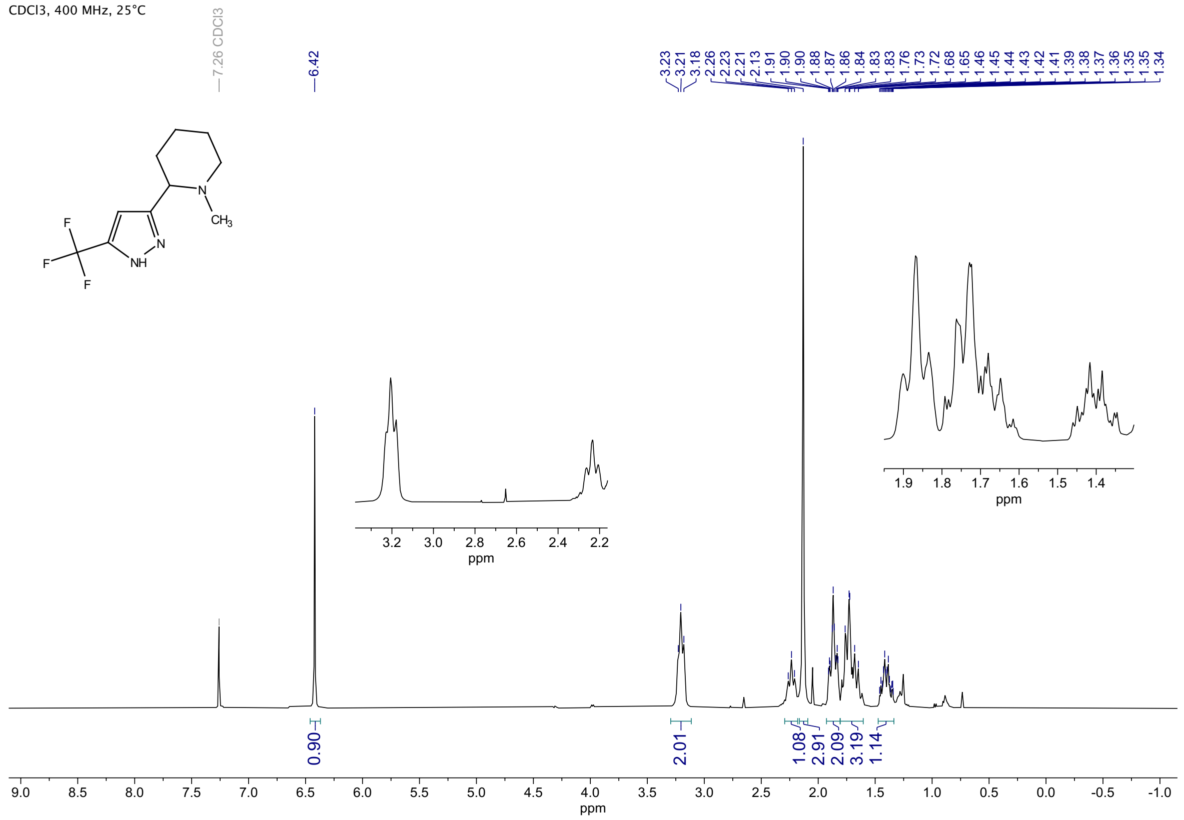

^{13}C NMR of 1-methyl-2-(5-(trifluoromethyl)-1H-pyrazol-3-yl)piperidine (**5i-C5-CF$_3$**)

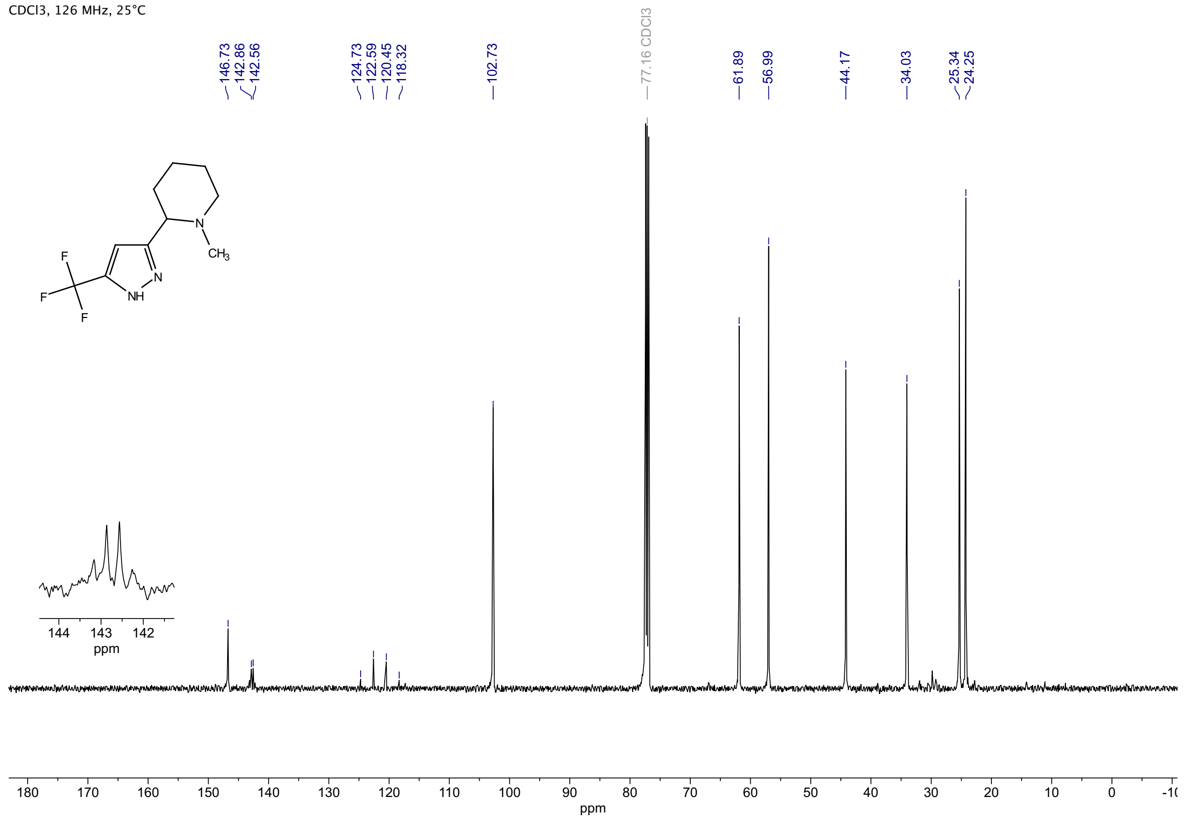

^{19}F NMR of 1-methyl-2-(5-(trifluoromethyl)-1H-pyrazol-3-yl)piperidine (**5i-C5-CF$_3$**)

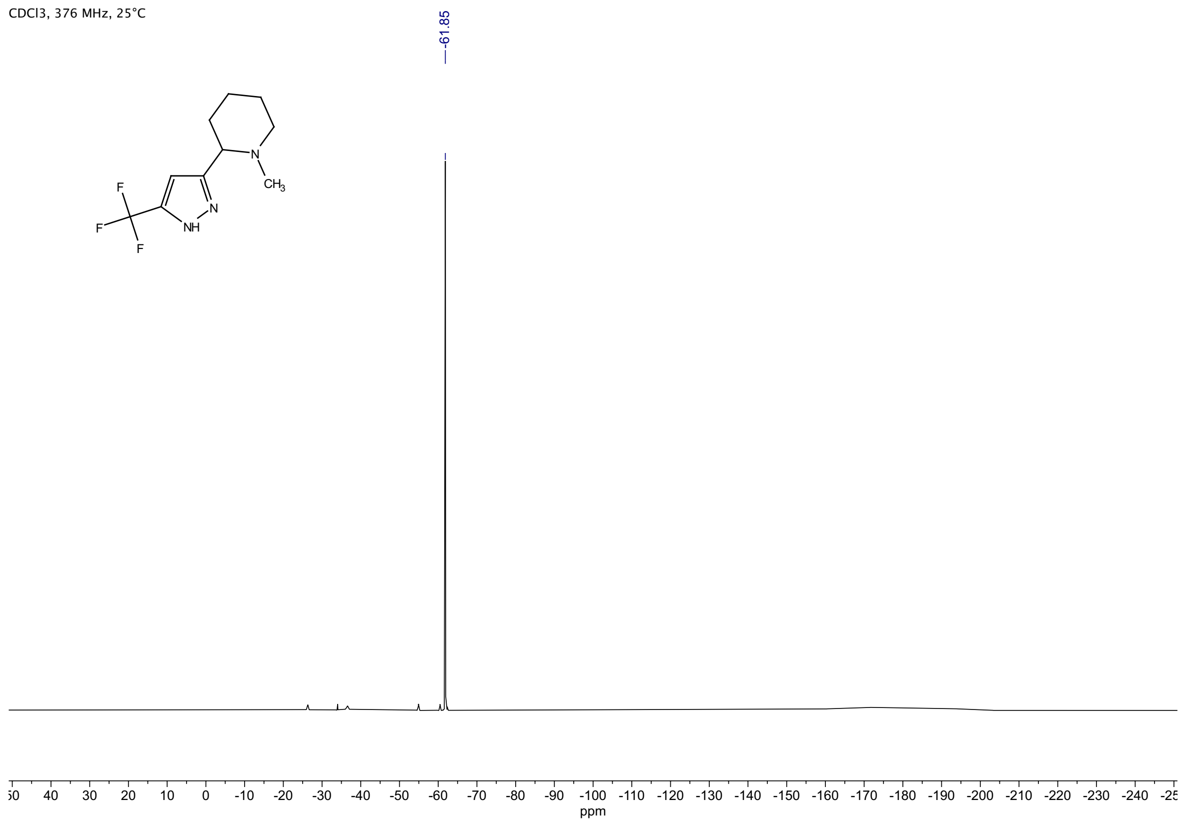

^{1}H NMR of 4-fluoro-5-(trifluoromethyl)-*1H*-pyrazole (**5j-monoCF$_3$**)

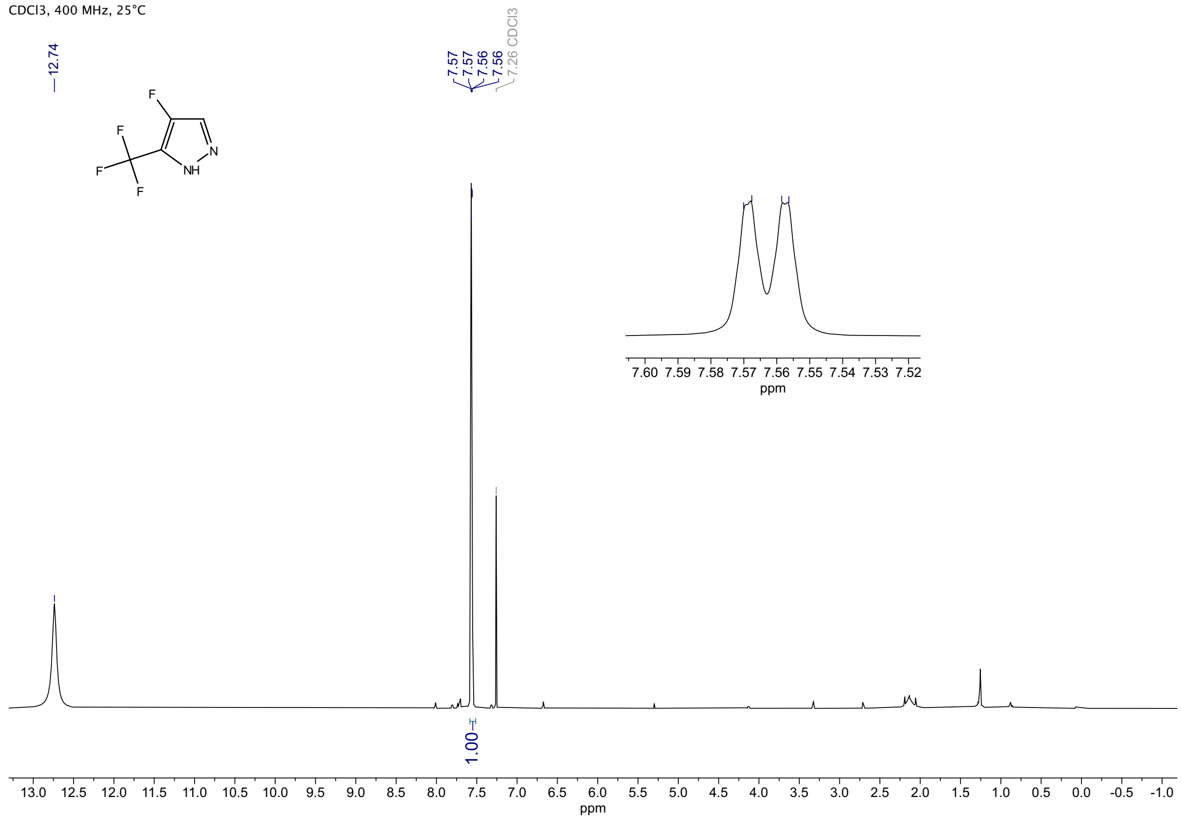

^{13}C NMR of 4-fluoro-5-(trifluoromethyl)-*1H*-pyrazole (**5j-monoCF$_3$**)

CDCl3, 126 MHz, 25°C

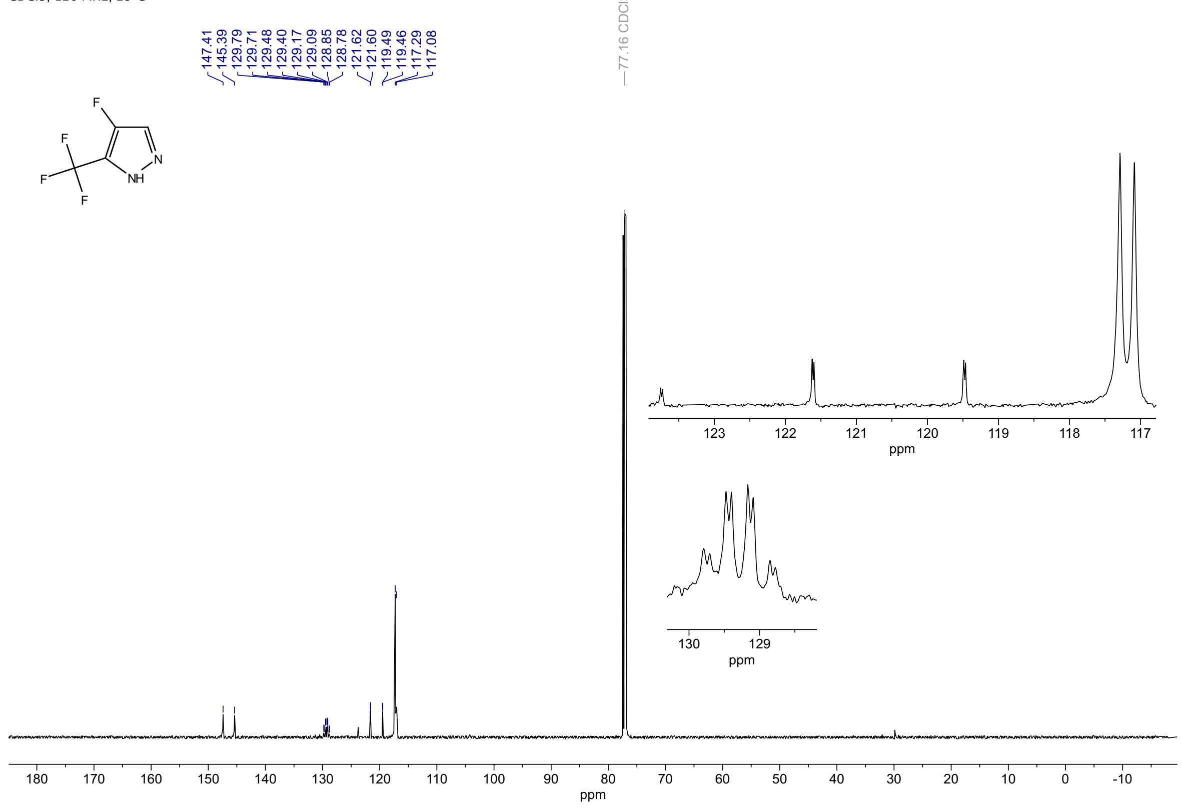

^{19}F NMR of 4-fluoro-5-(trifluoromethyl)-*1H*-pyrazole (**5j-monoCF$_3$**)

CDCl3, 376 MHz, 25°C

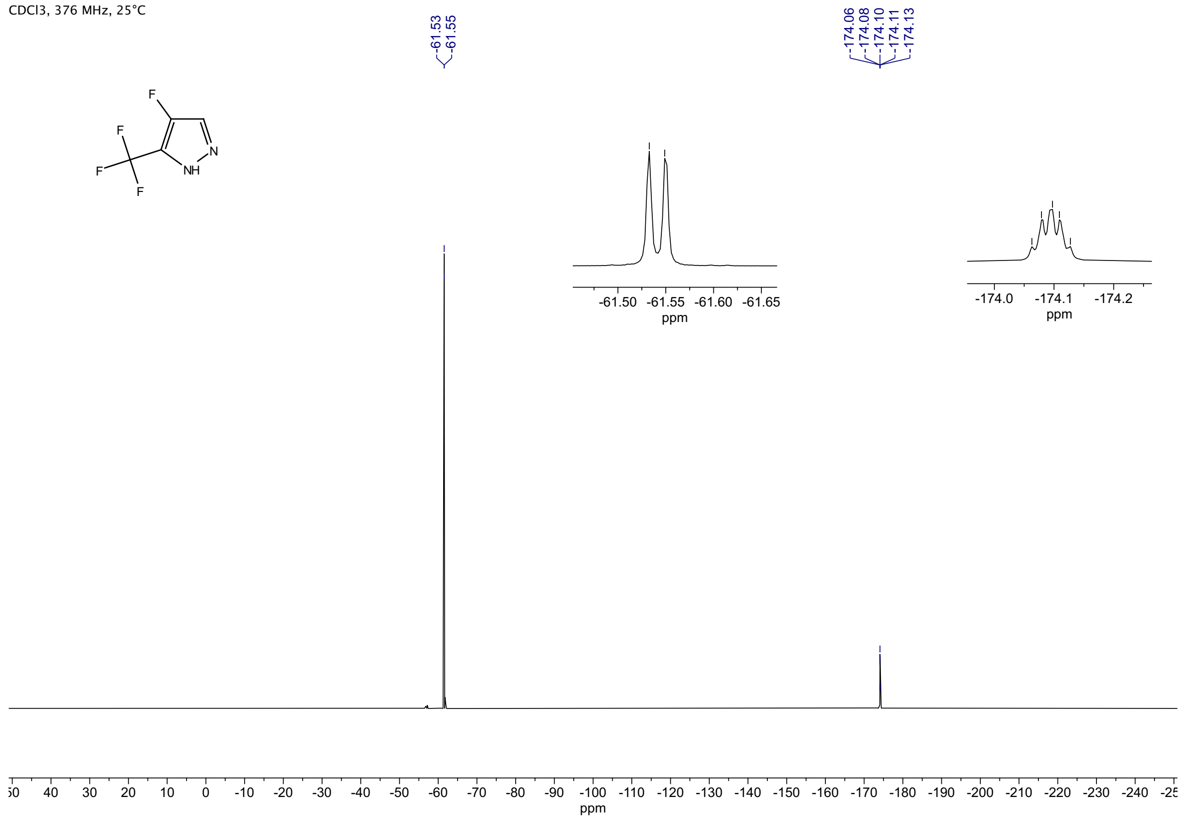

^{1}H NMR of 4-bromo-3-methoxy-5-(trifluoromethyl)-*1H*-pyrazole (**6a**)

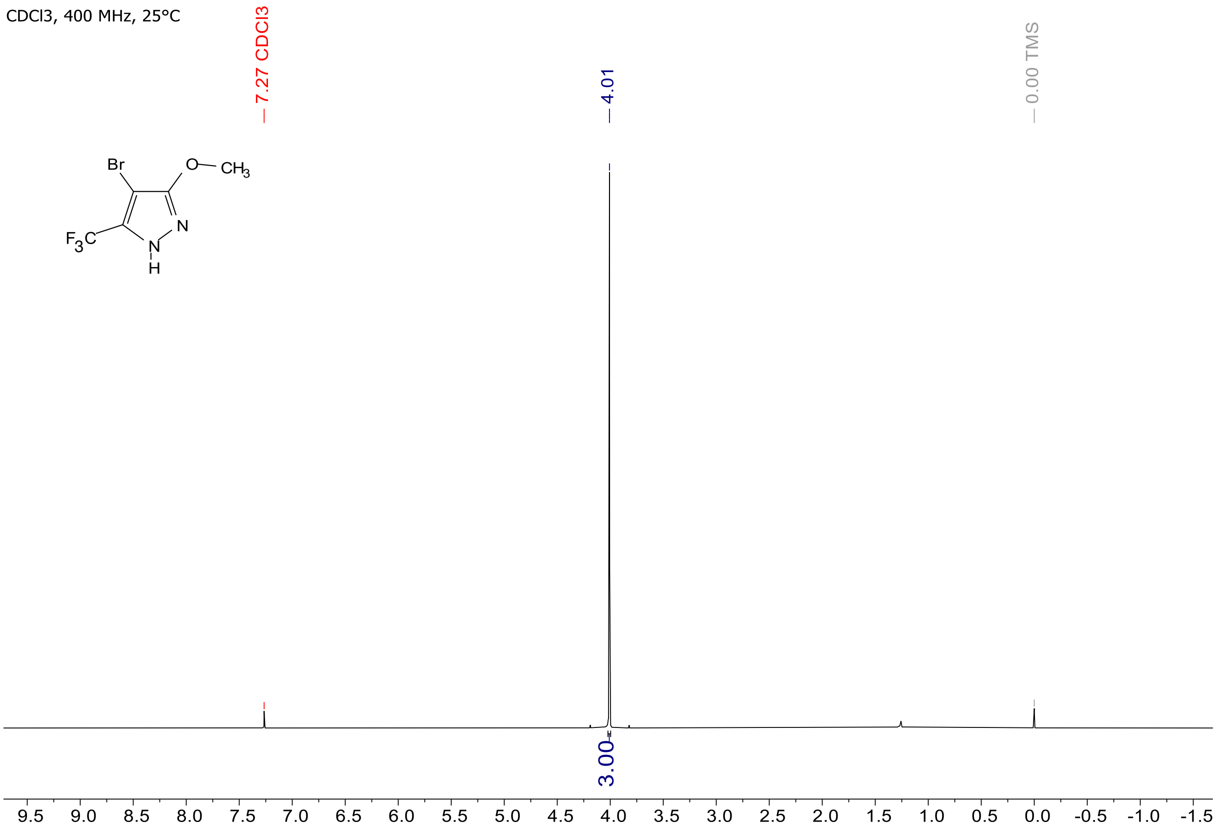

^{13}C NMR of 4-bromo-3-methoxy-5-(trifluoromethyl)-*1H*-pyrazole (**6a**)

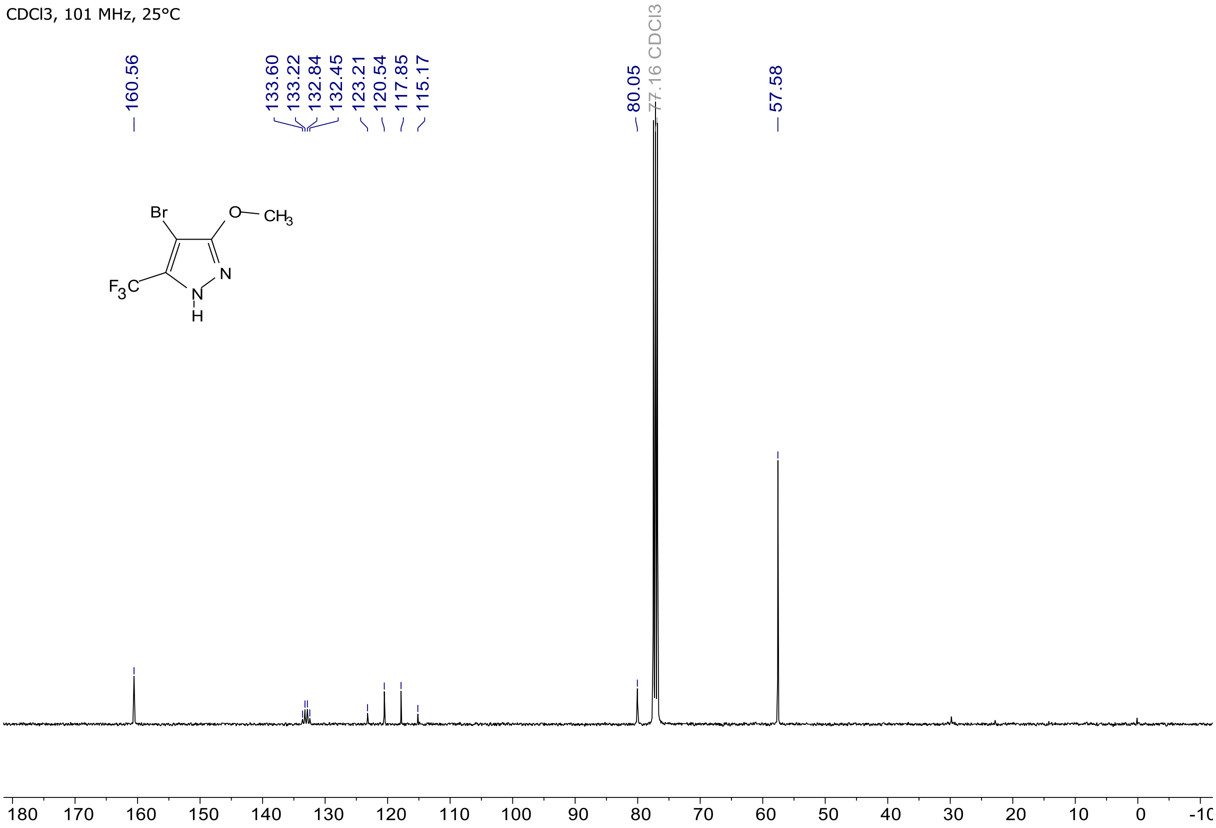

^{19}F NMR of 4-bromo-3-methoxy-5-(trifluoromethyl)-*1H*-pyrazole (**6a**)

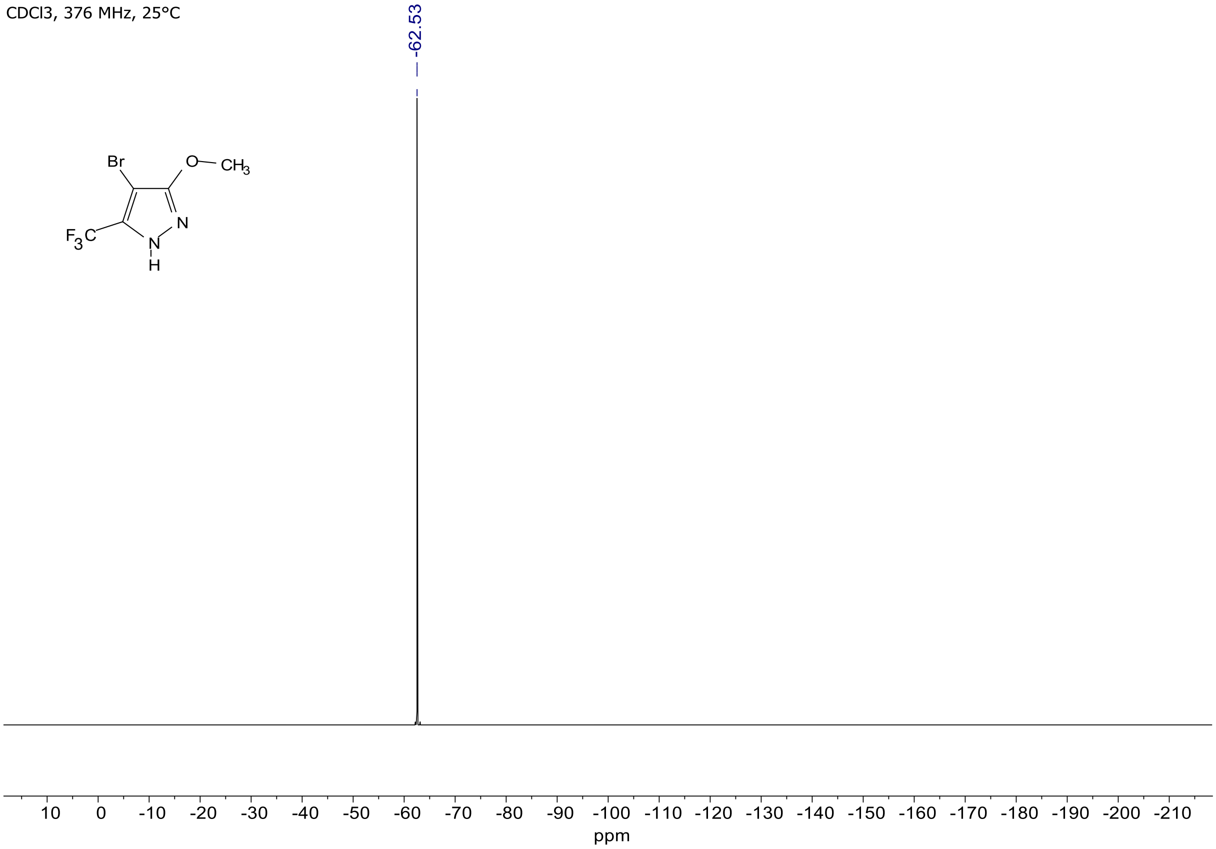

^{1}H NMR of 3-bromo-4-methoxy-5-(trifluoromethyl)-*1H*-pyrazole (**6b**)

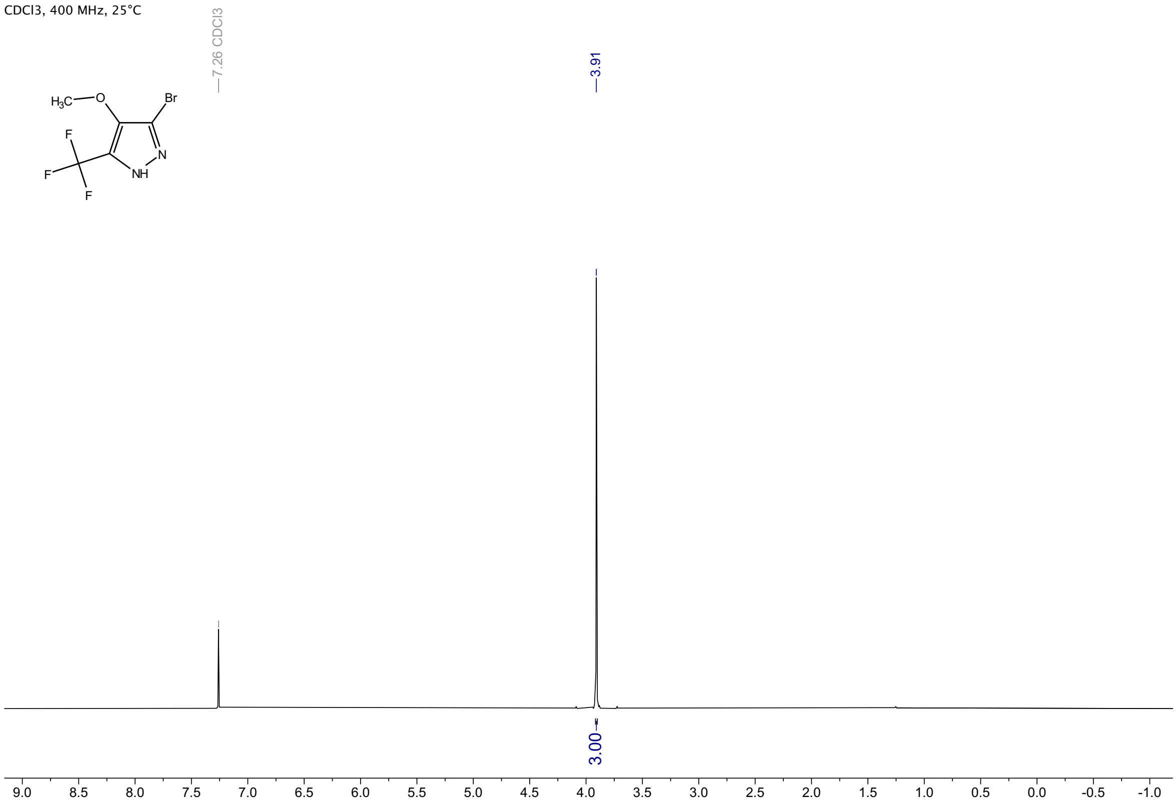

^{13}C NMR of 3-bromo-4-methoxy-5-(trifluoromethyl)-*1H*-pyrazole (**6b**)

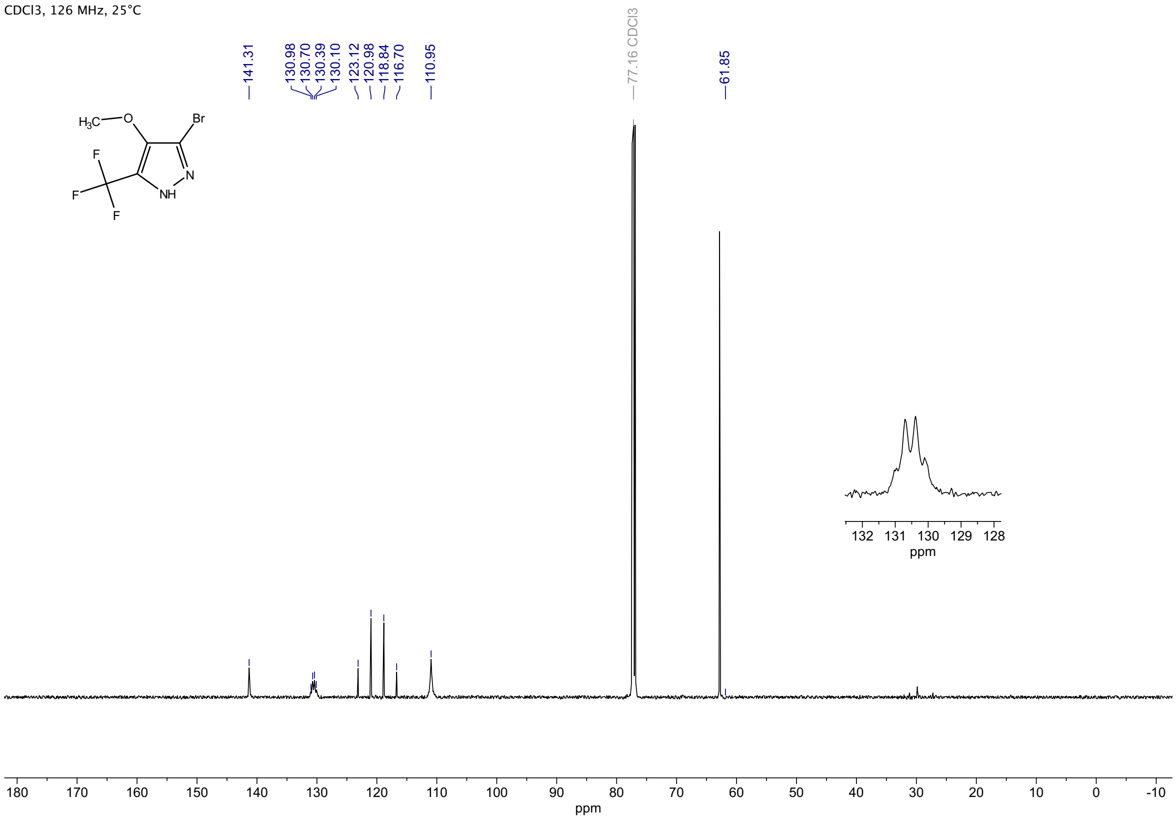

^{19}F NMR of 3-bromo-4-methoxy-5-(trifluoromethyl)-*1H*-pyrazole (**6b**)

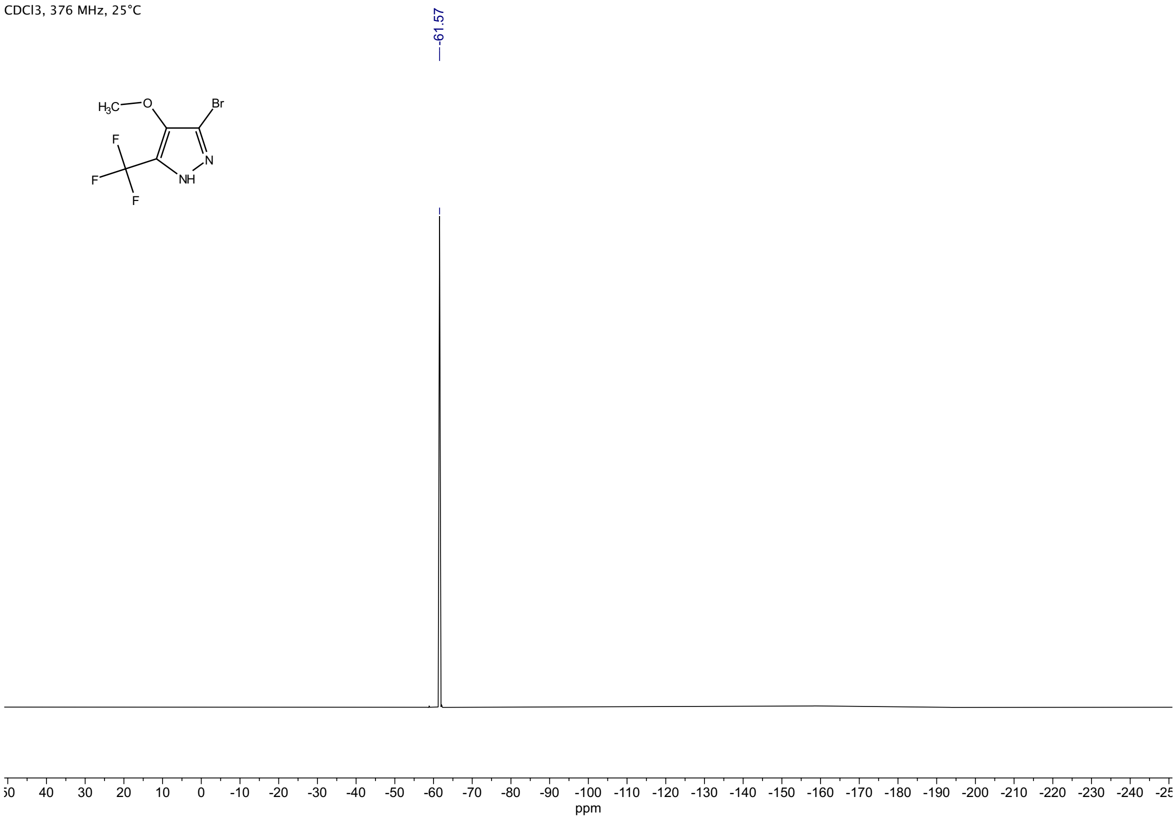

^{1}H NMR of 3-iodo-4-methoxy-5-(trifluoromethyl)-*1H*-pyrazole (**6c**)

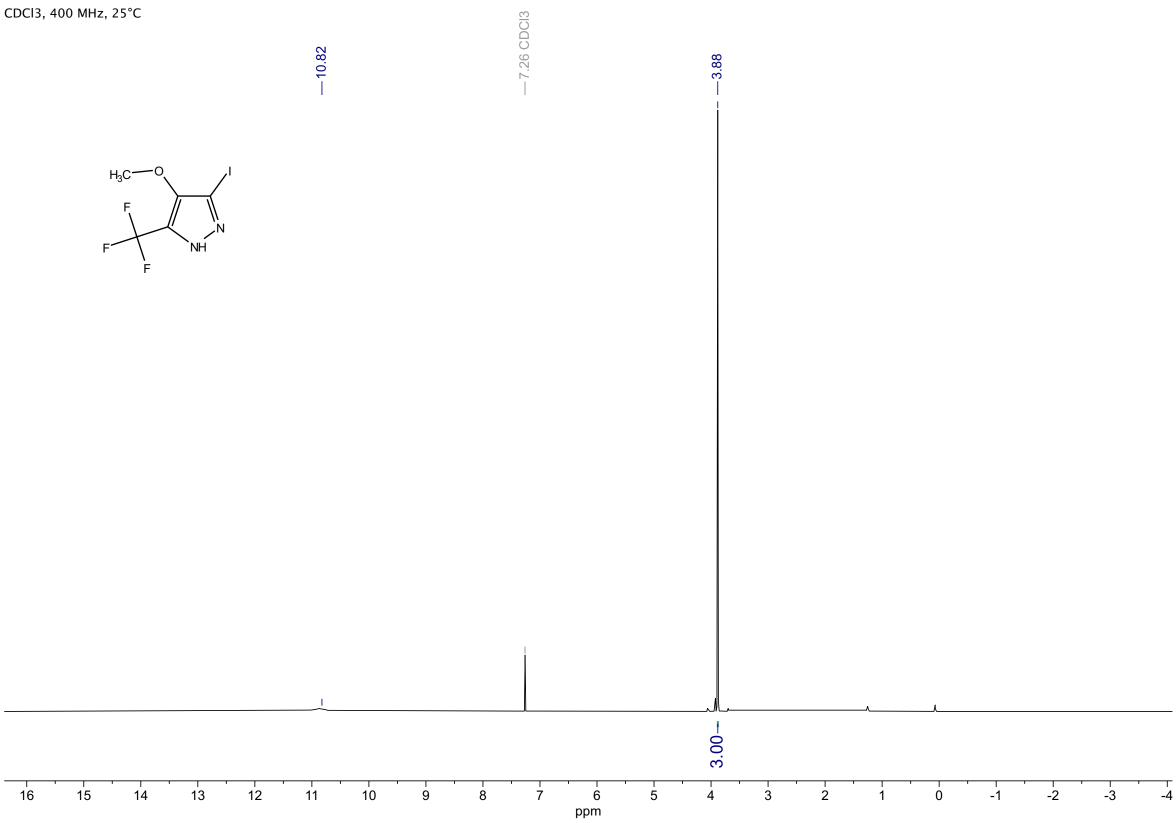

^{13}C NMR of 3-iodo-4-methoxy-5-(trifluoromethyl)-*1H*-pyrazole (**6c**)

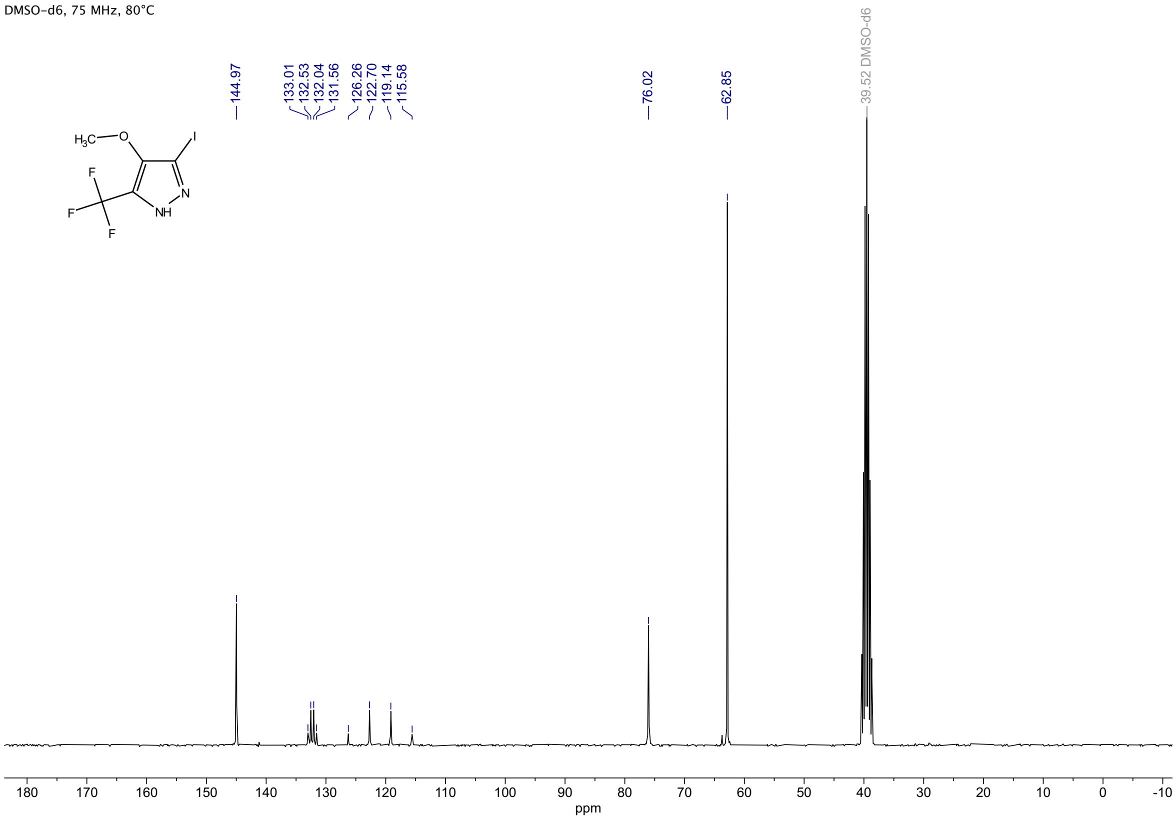

^{19}F NMR of 3-iodo-4-methoxy-5-(trifluoromethyl)-*1H*-pyrazole (**6b**)

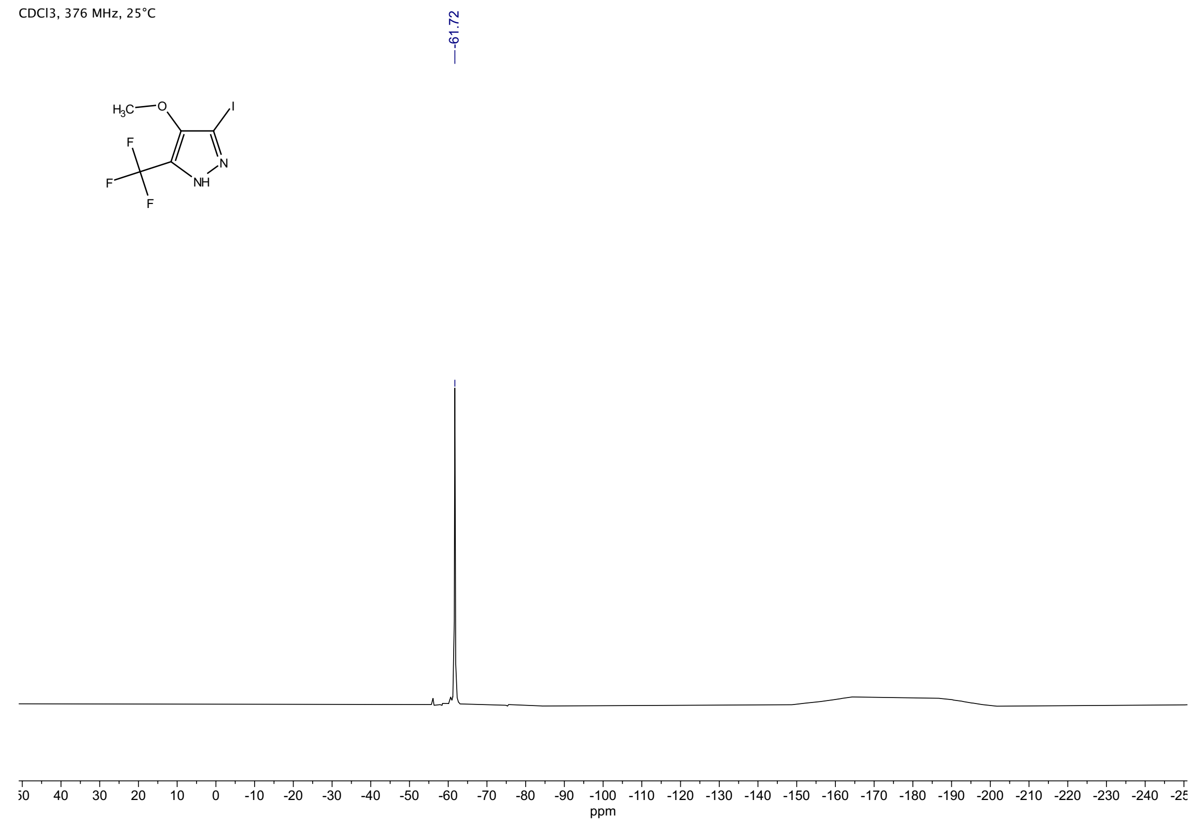

^{1}H NMR of 3-(trifluoromethyl)-6,7-dihydro-5*H*-pyrazolo[5,1-*b*][1,3]oxazine-2-carboxylic acid (**6d**)

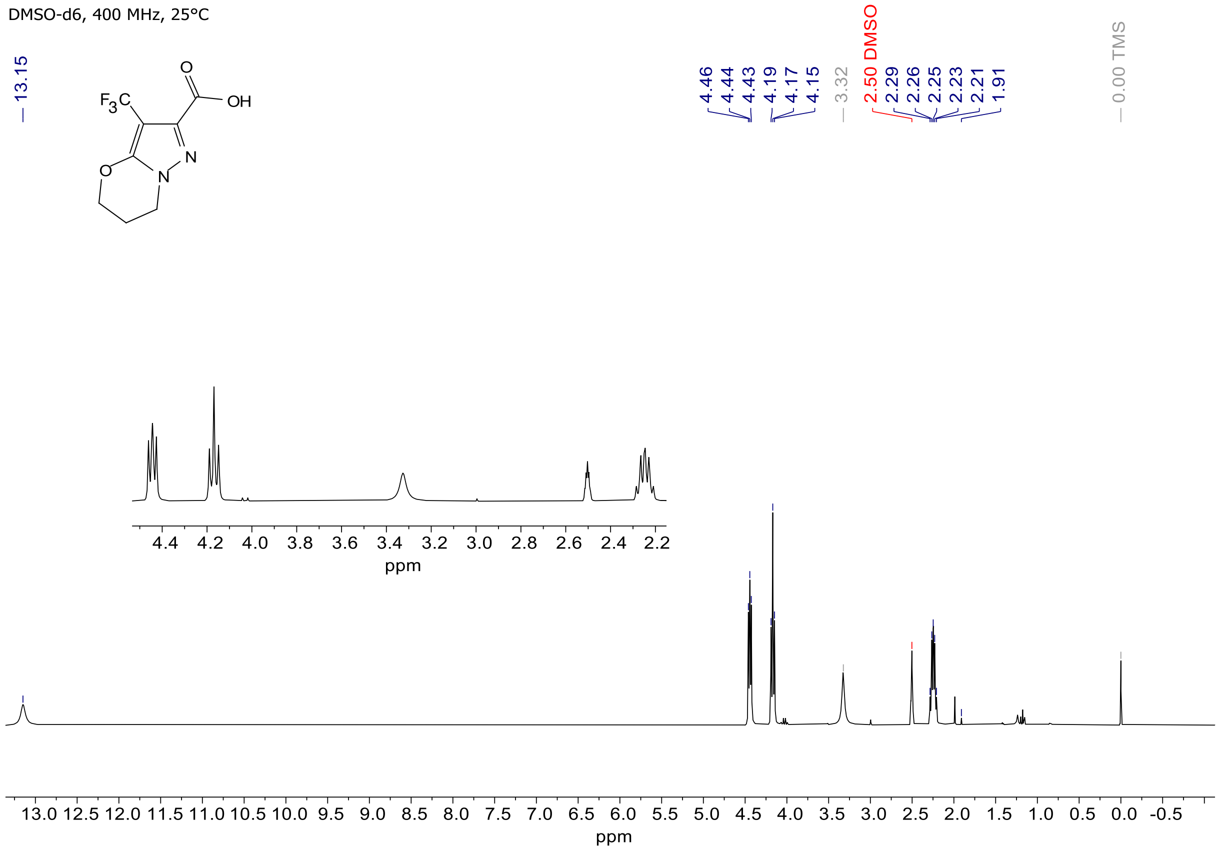

^{13}C NMR of 3-(trifluoromethyl)-6,7-dihydro-5*H*-pyrazolo[5,1-*b*][1,3]oxazine-2-carboxylic acid (**6d**)

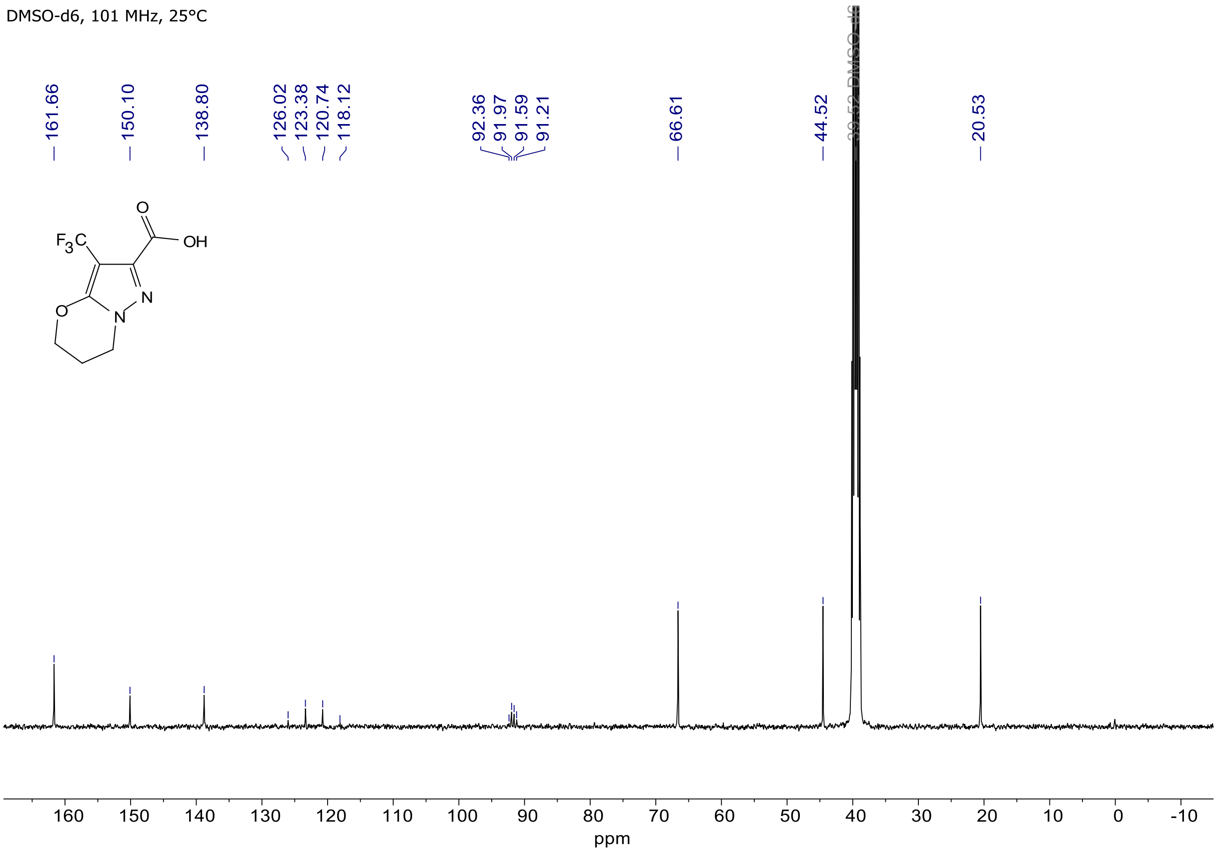

^{19}F NMR of 3-(trifluoromethyl)-6,7-dihydro-5*H*-pyrazolo[5,1-*b*][1,3]oxazine-2-carboxylic acid (**6d**)

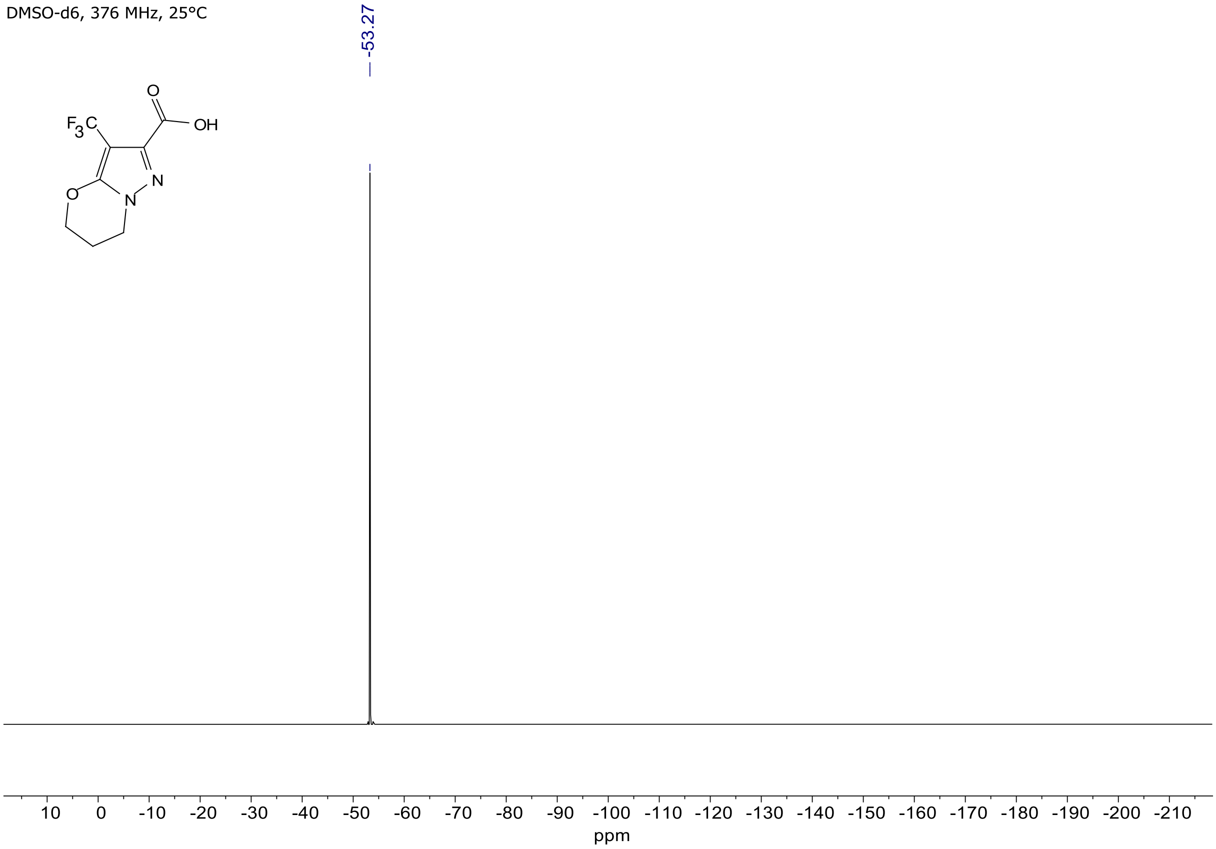

^{1}H NMR of 4-chloro-3-cyclopropyl-5-(trifluoromethyl)-*1H*-pyrazole (**6e**)

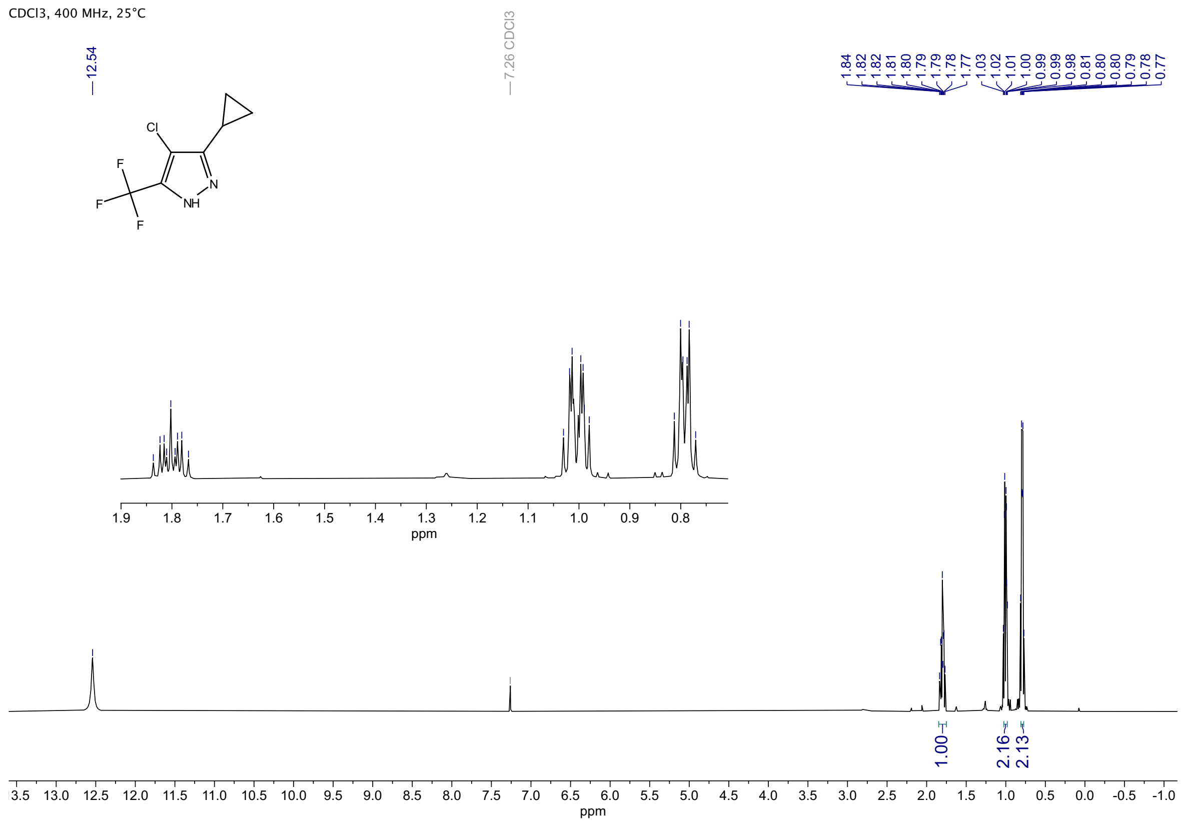

^{13}C NMR of 4-chloro-3-cyclopropyl-5-(trifluoromethyl)-*1H*-pyrazole (**6e**)

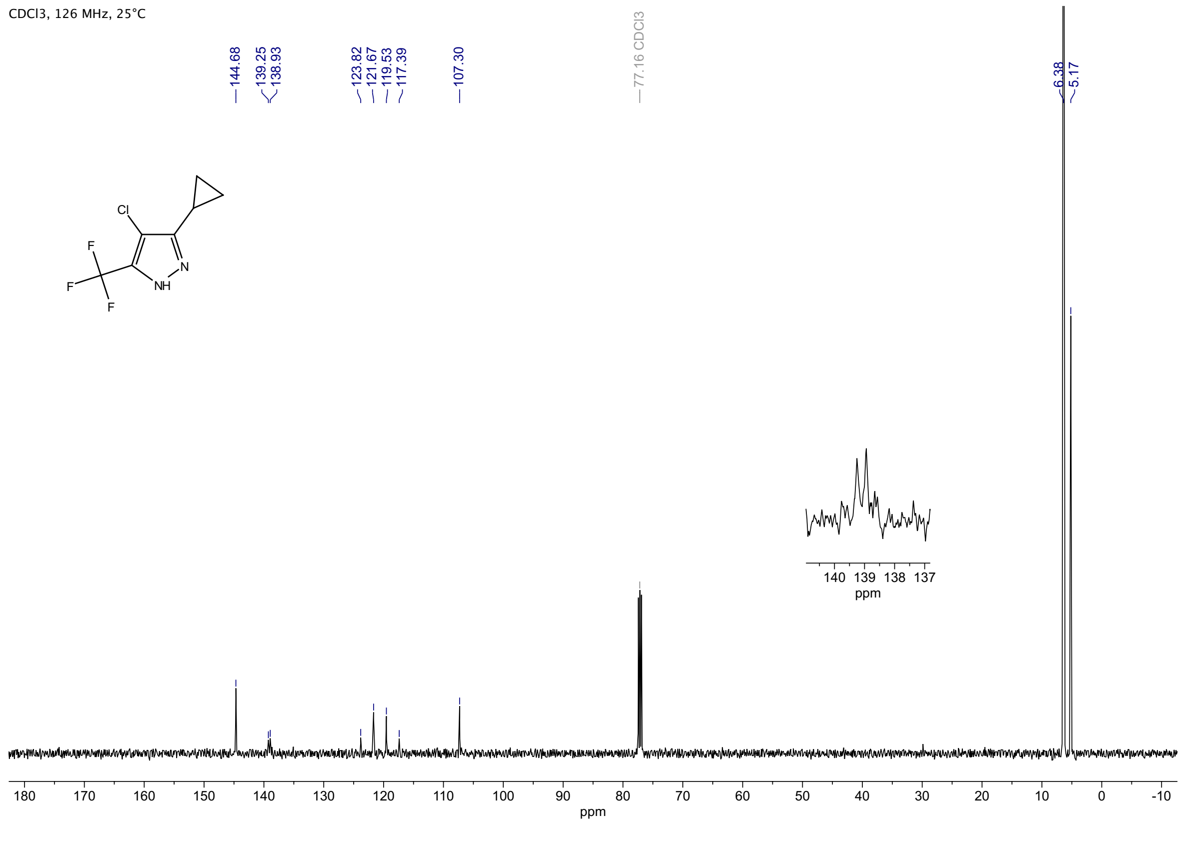

^{19}F NMR of 4-chloro-3-cyclopropyl-5-(trifluoromethyl)-*1H*-pyrazole (**6e**)

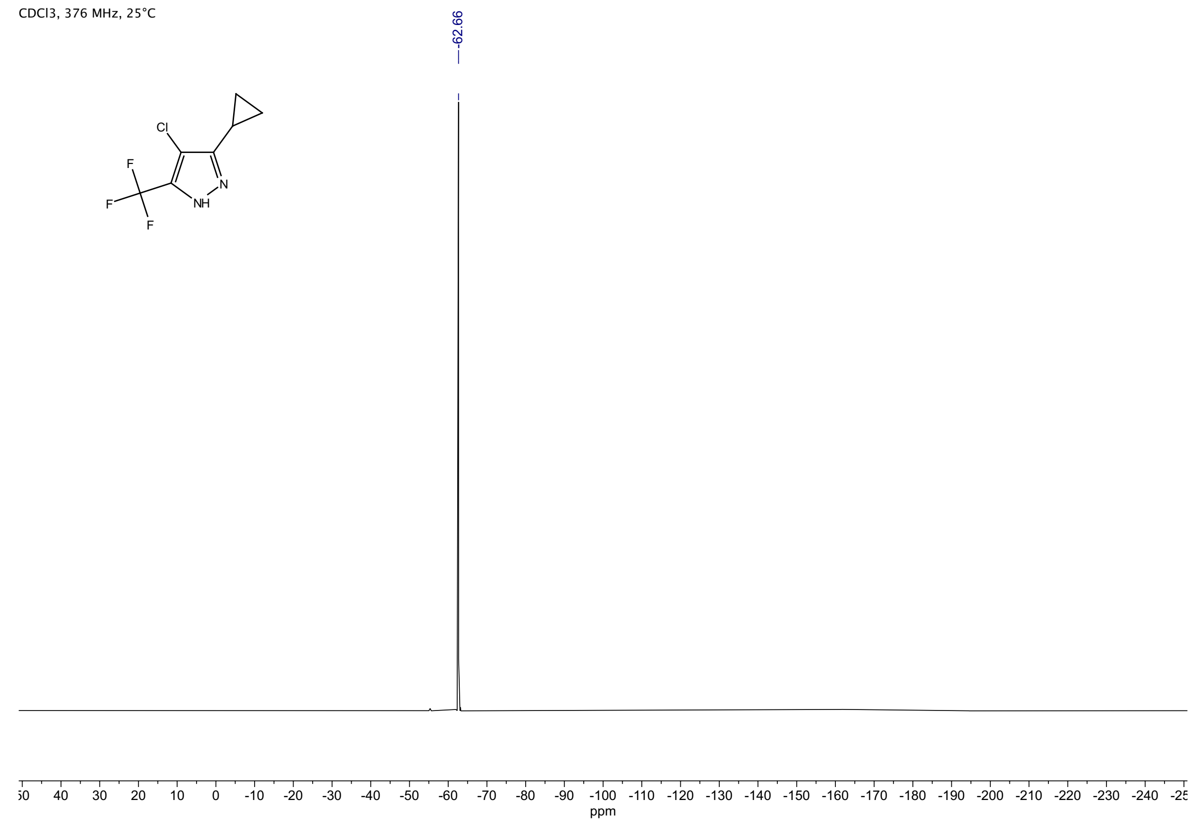

^{1}H NMR of methyl 4-chloro-5-(trifluoromethyl)-*1H*-pyrazole-3-carboxylate (**6f**)

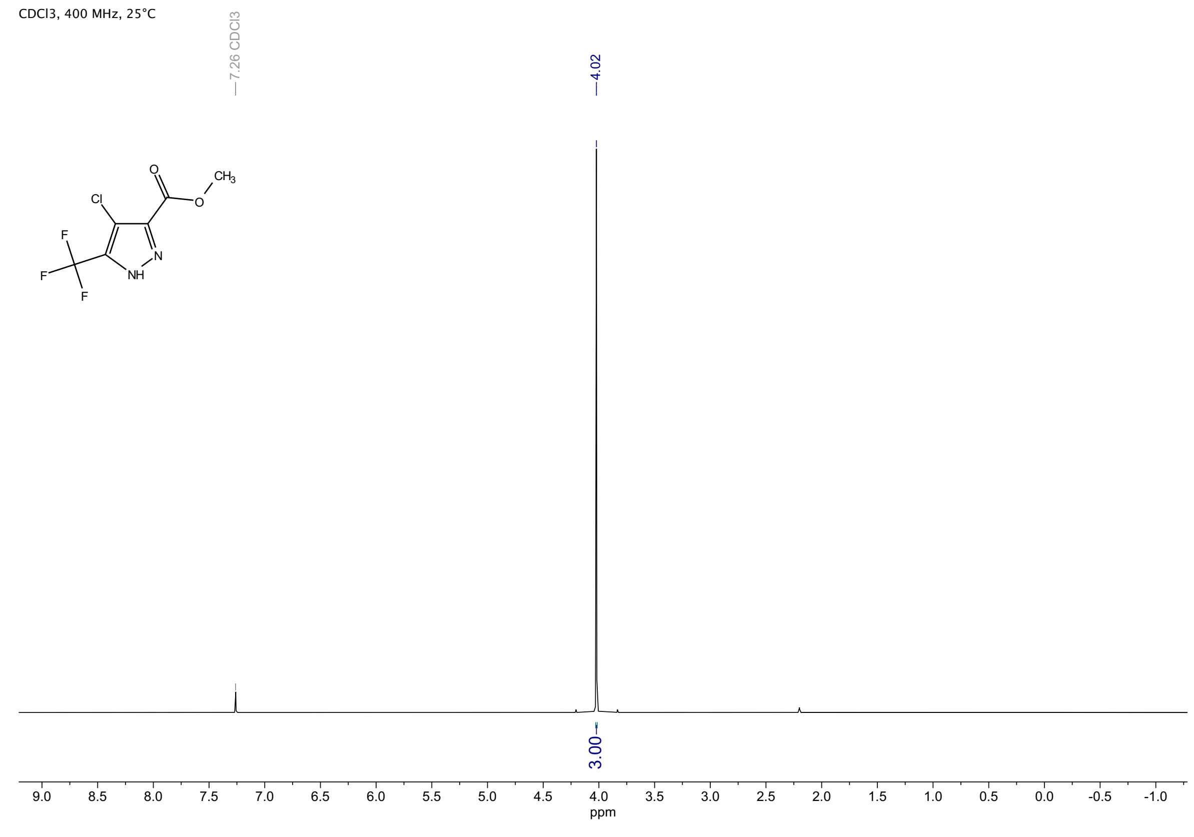

^{13}C NMR of methyl 4-chloro-5-(trifluoromethyl)-*1H*-pyrazole-3-carboxylate (**6f**)

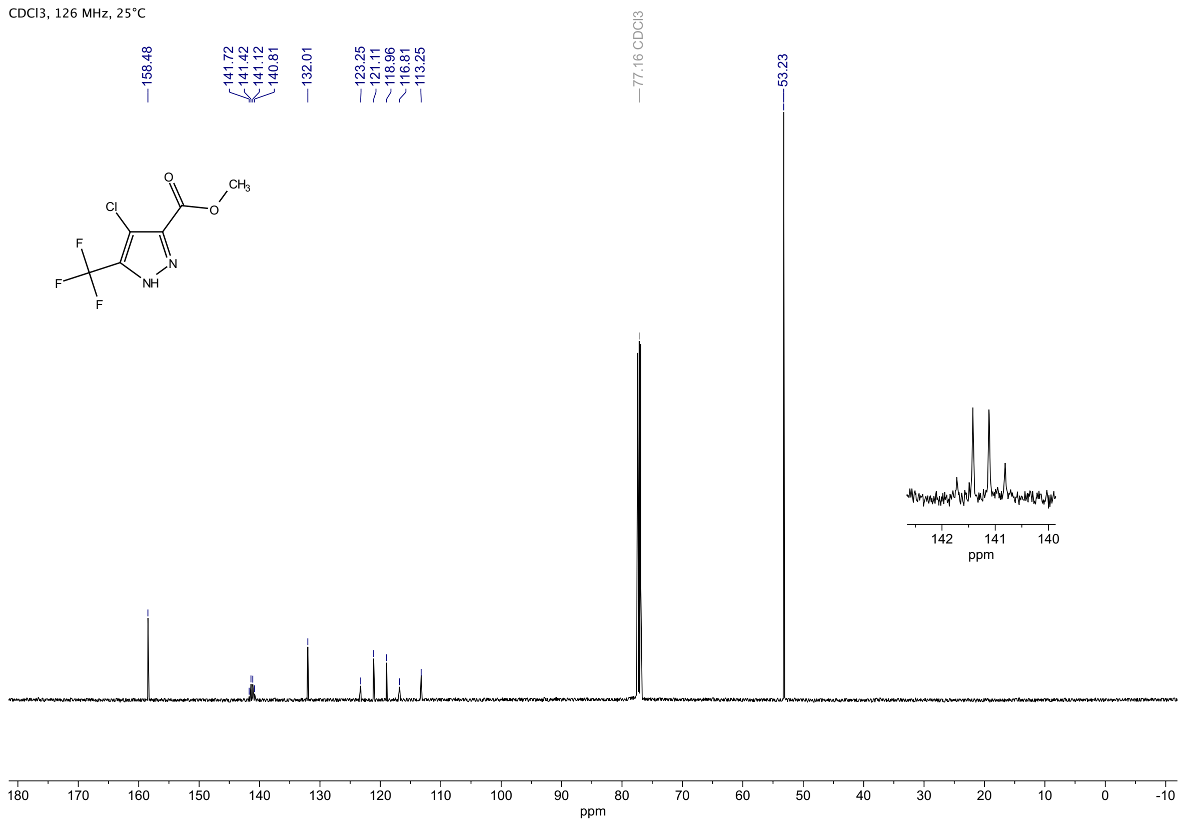

^{19}F NMR of methyl 4-chloro-5-(trifluoromethyl)-*1H*-pyrazole-3-carboxylate (**6f**)

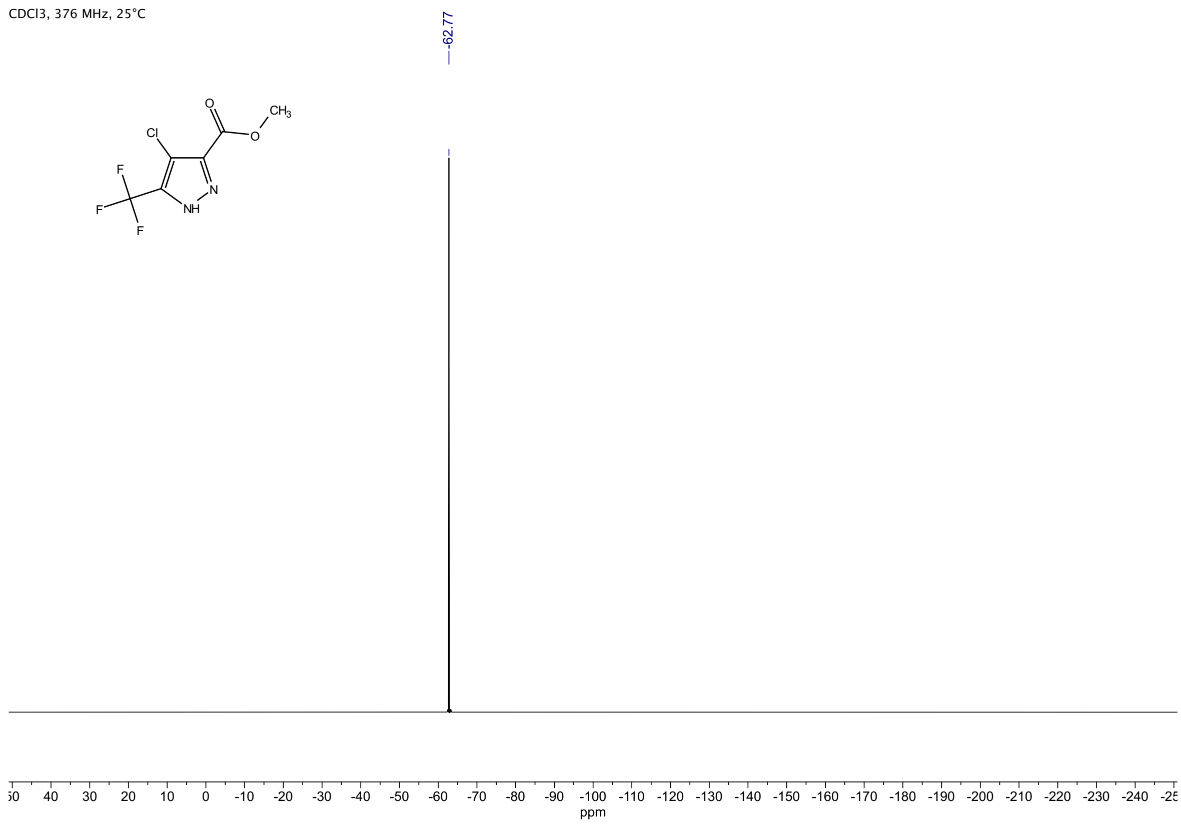

^{1}H NMR of methyl 4-bromo-5-(trifluoromethyl)-*1H*-pyrazole-3-carboxylate (**6g**)

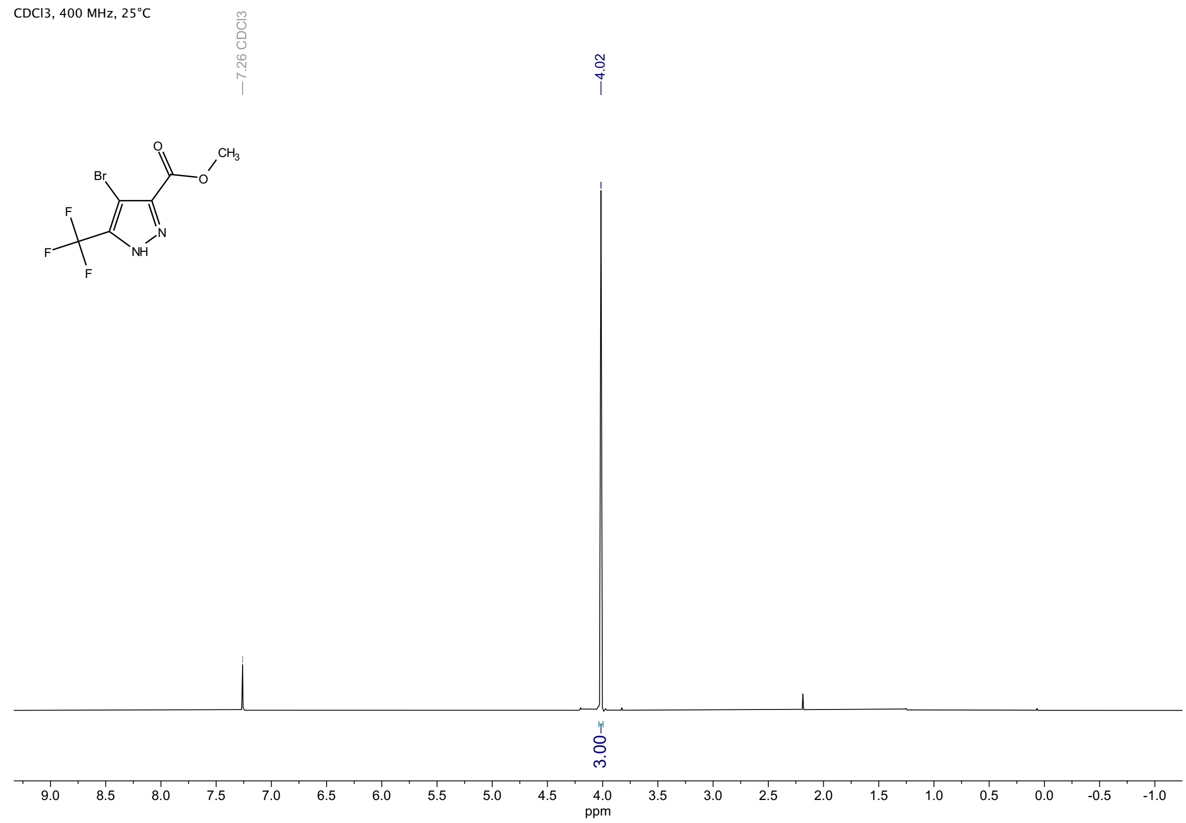

^{13}C NMR of methyl 4-bromo-5-(trifluoromethyl)-*1H*-pyrazole-3-carboxylate (**6g**)

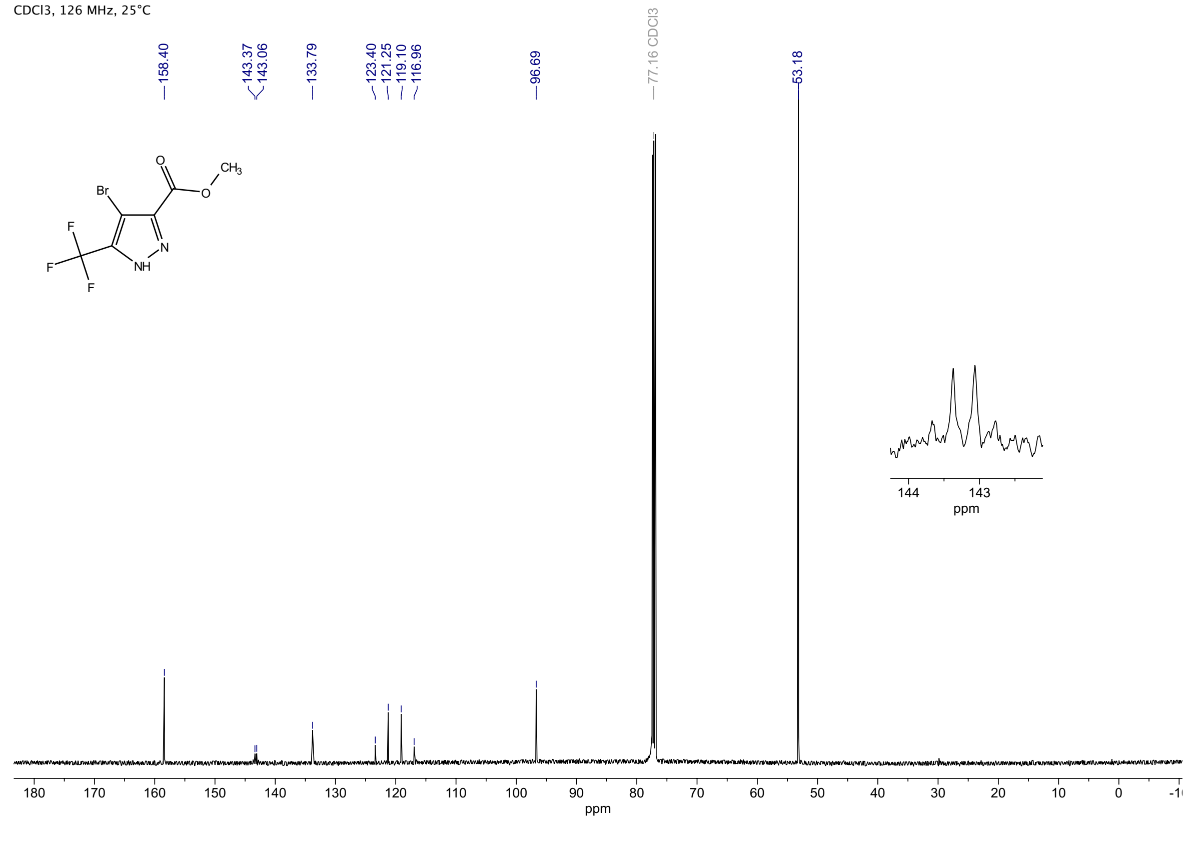

^{19}F NMR of methyl 4-bromo-5-(trifluoromethyl)-*1H*-pyrazole-3-carboxylate (**6g**)

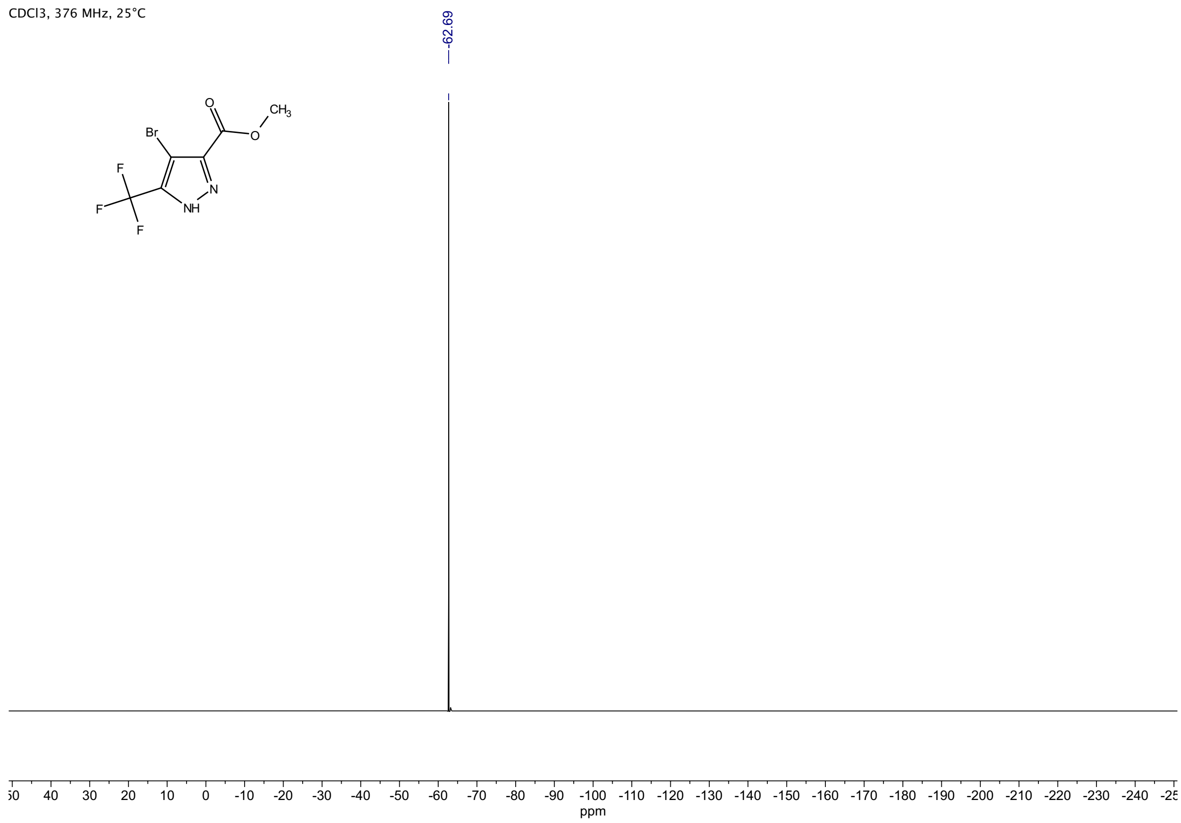

^{1}H NMR of 4-bromo-5-isopropyl-3-(trifluoromethyl)-*1H*-pyrazole (**6h**)

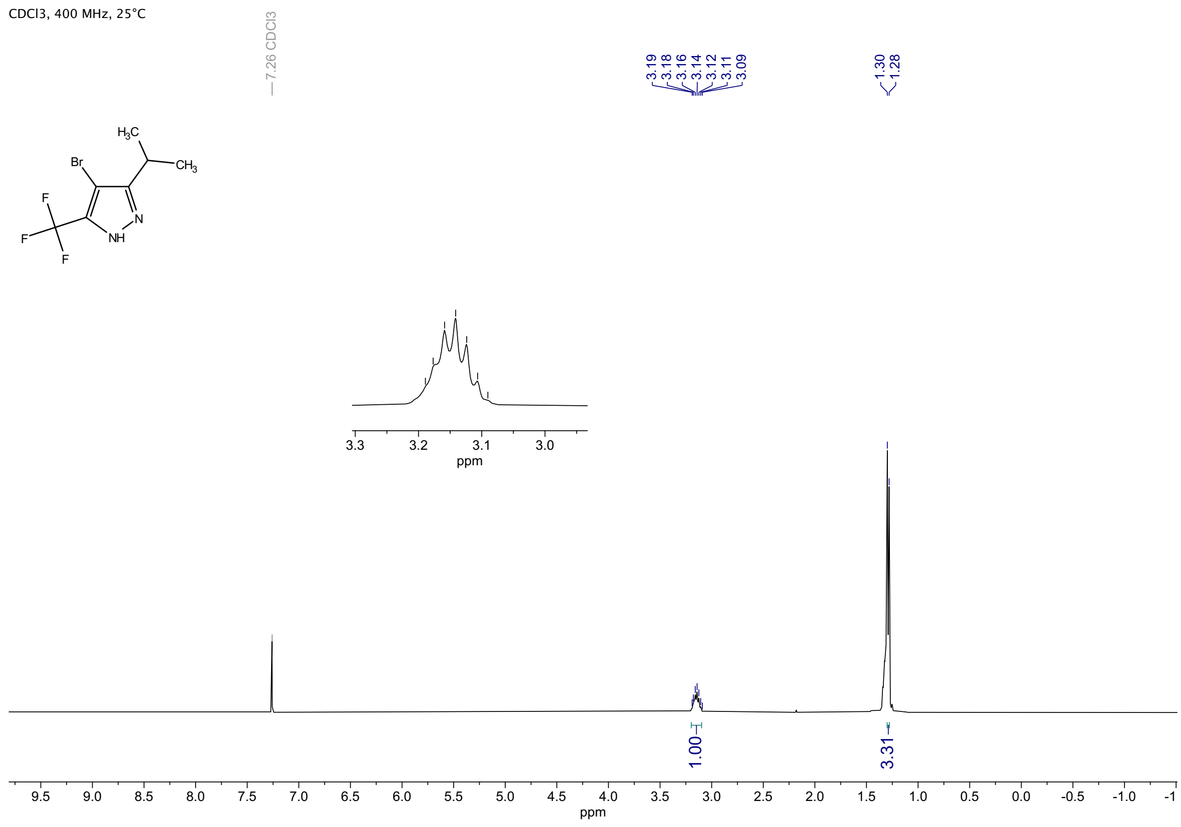

^{13}C NMR of 4-bromo-5-isopropyl-3-(trifluoromethyl)-*1H*-pyrazole (**6h**)

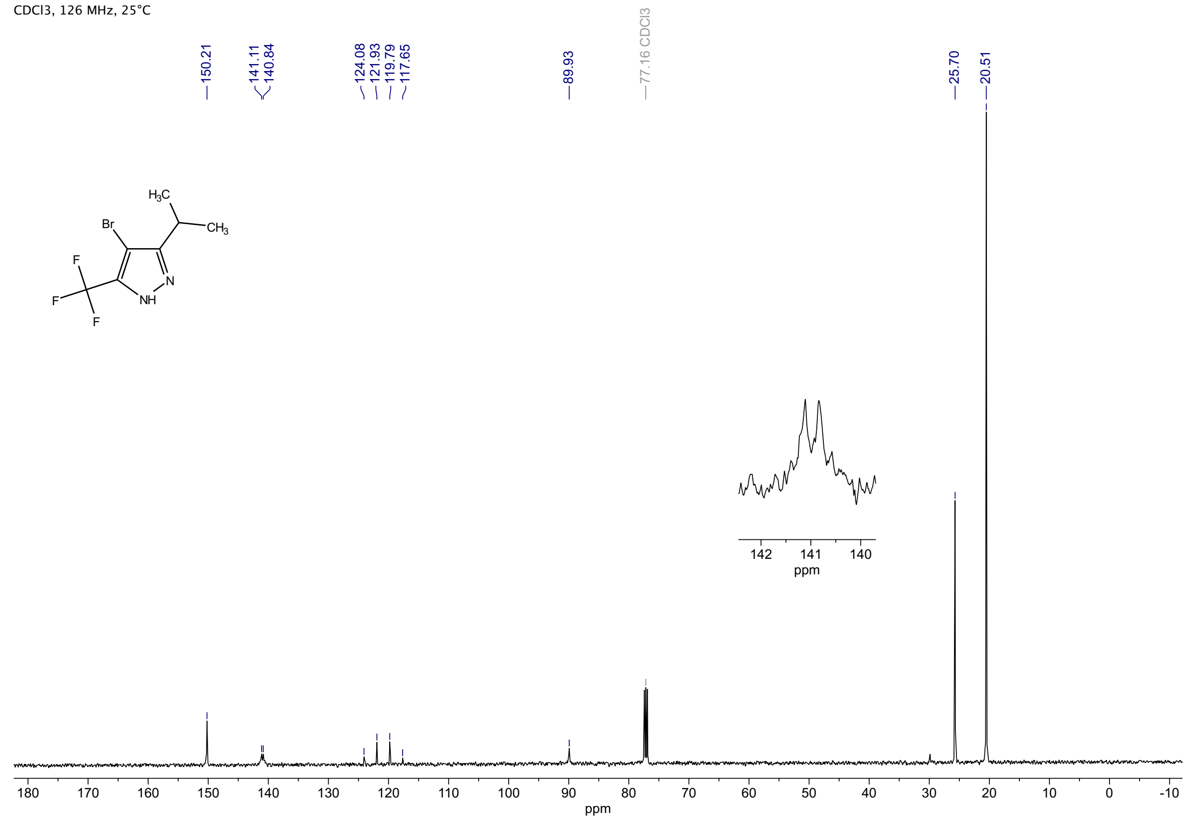

^{19}F NMR of 4-bromo-5-isopropyl-3-(trifluoromethyl)-*1H*-pyrazole (**6h**)

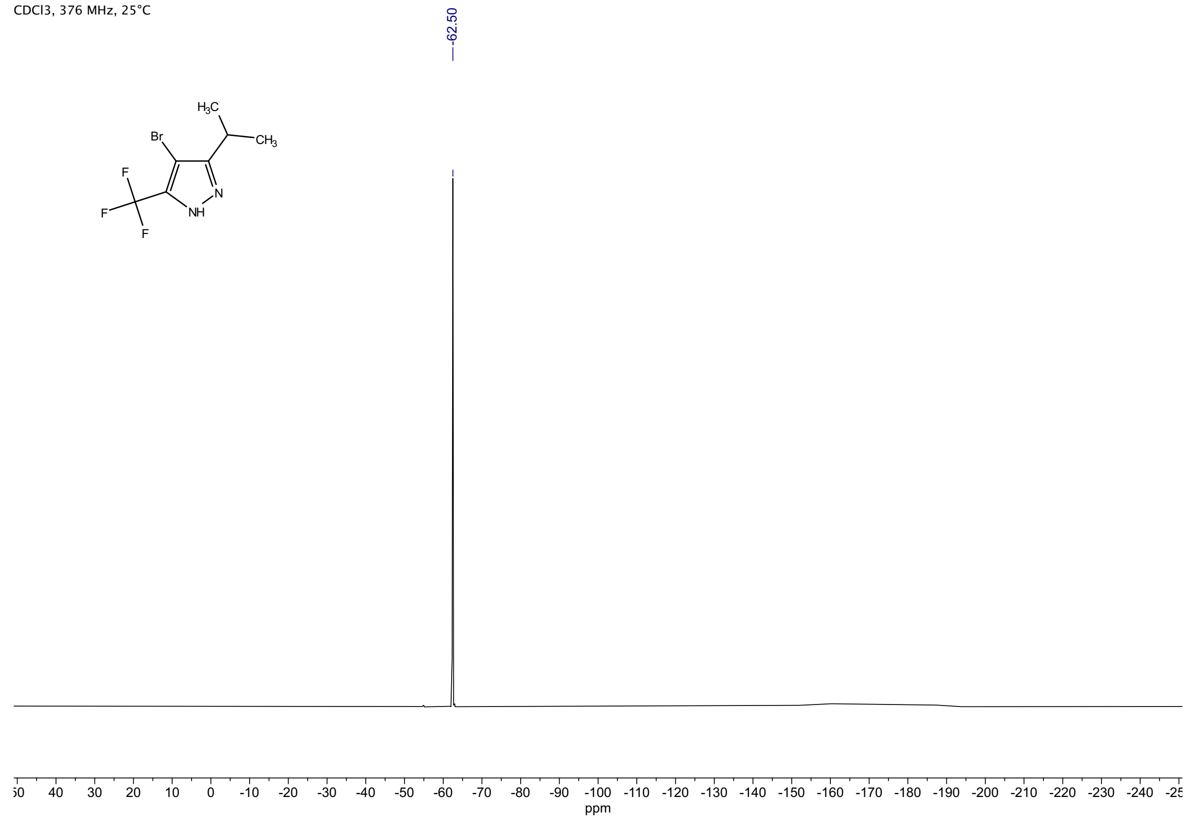

^{1}H NMR of 4-iodo-5-isopropyl-3-(trifluoromethyl)-*1H*-pyrazole (**6i**)

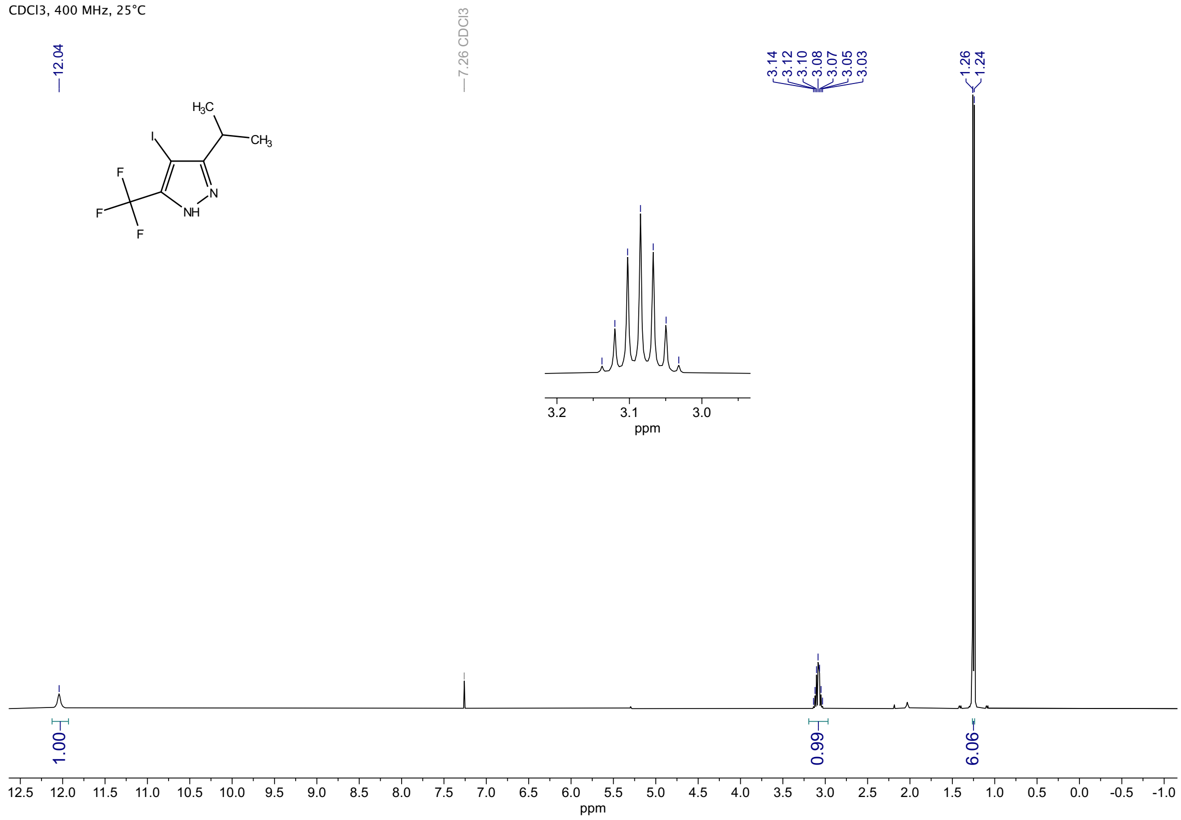

^{13}C NMR of 4-iodo-5-isopropyl-3-(trifluoromethyl)-*1H*-pyrazole (**6i**)

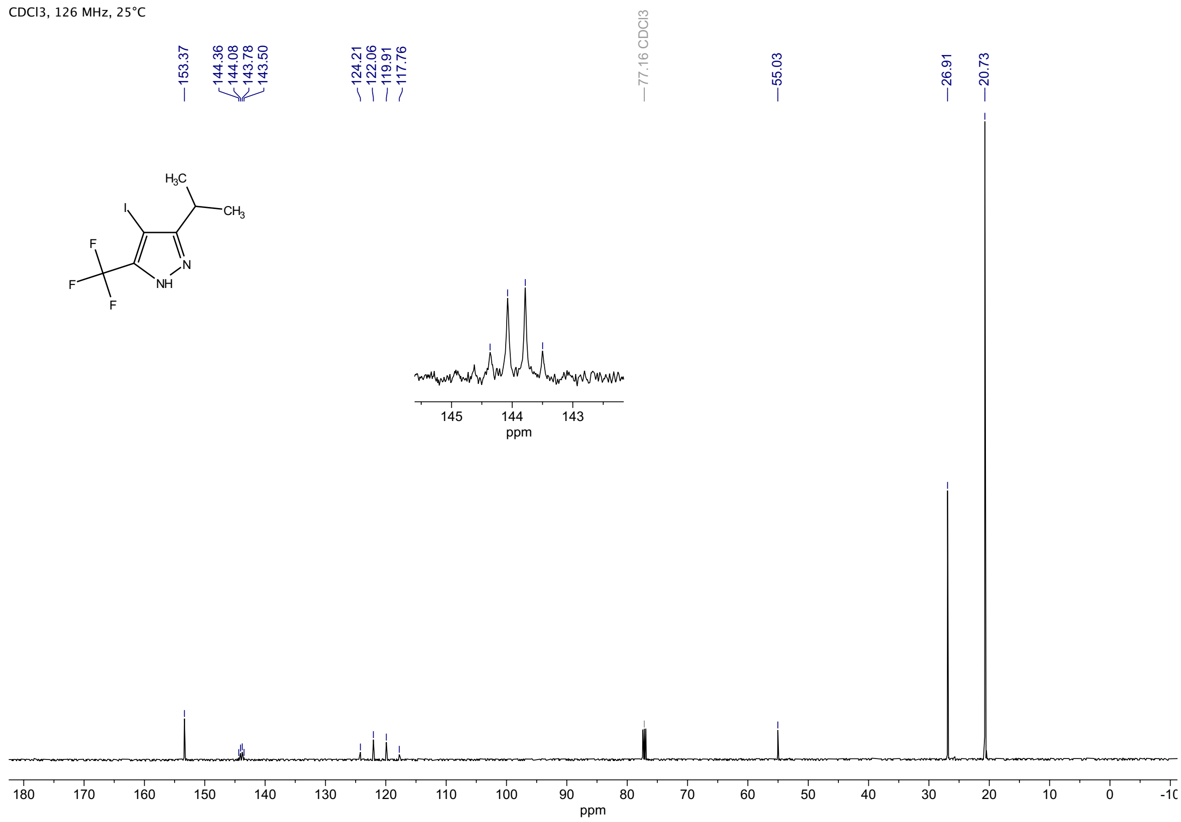

^{19}F NMR of 4-iodo-5-isopropyl-3-(trifluoromethyl)-*1H*-pyrazole (**6i**)

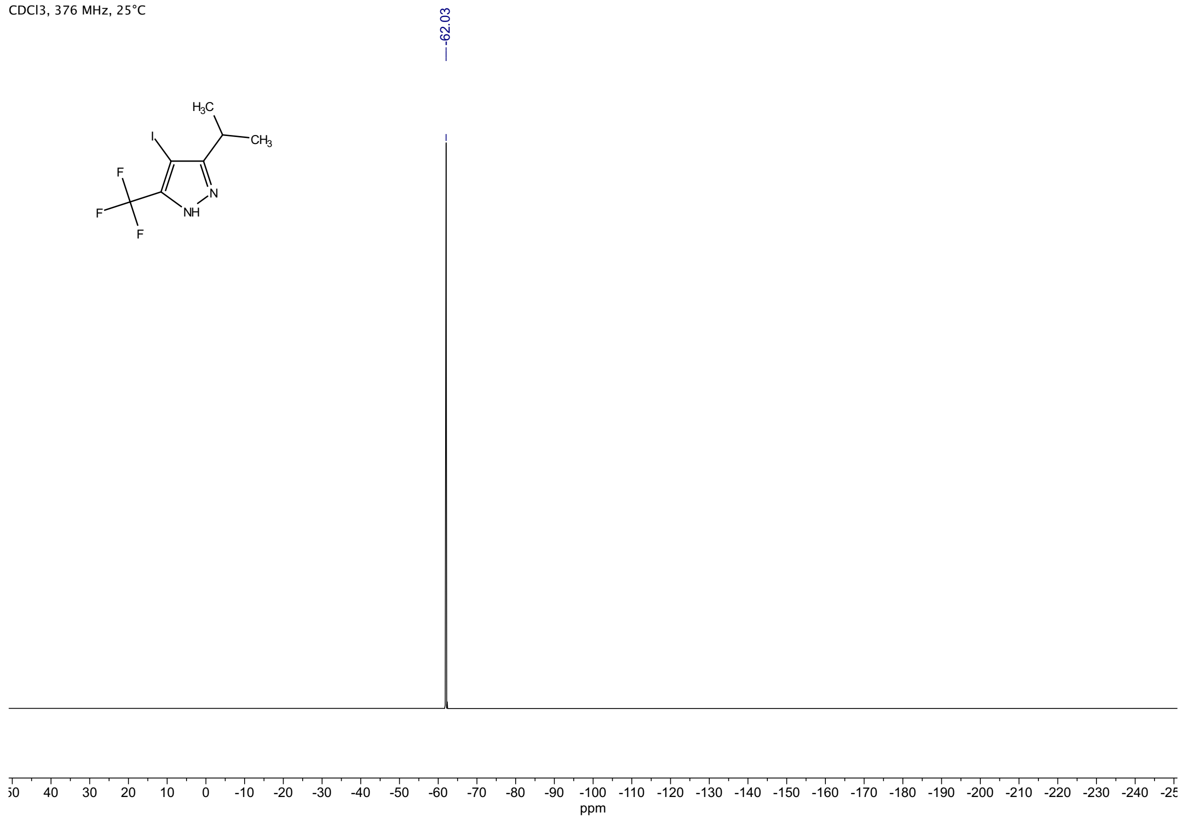

^{1}H NMR of 1-(4-bromo-5-(trifluoromethyl)-*1H*-pyrazol-3-yl)ethan-1-one (**6j**)

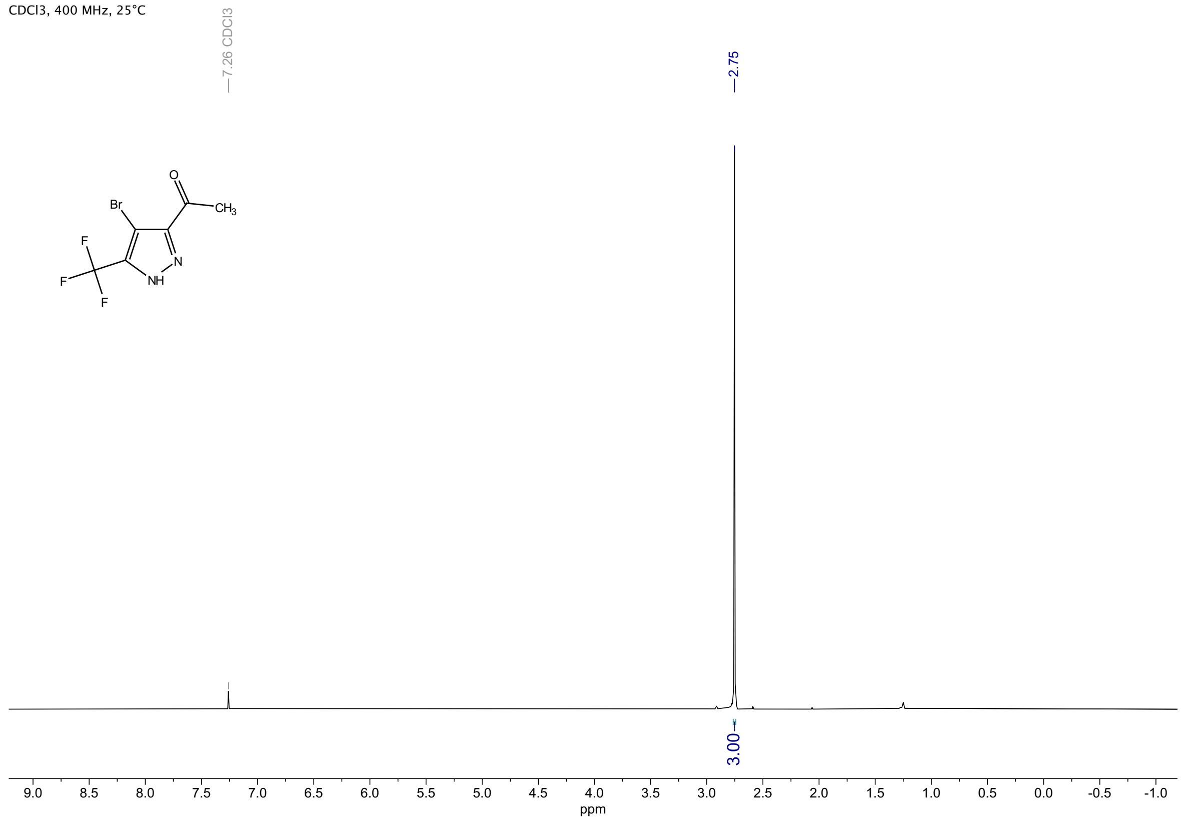

^{13}C NMR of 1-(4-bromo-5-(trifluoromethyl)-*1H*-pyrazol-3-yl)ethan-1-one (**6j**)

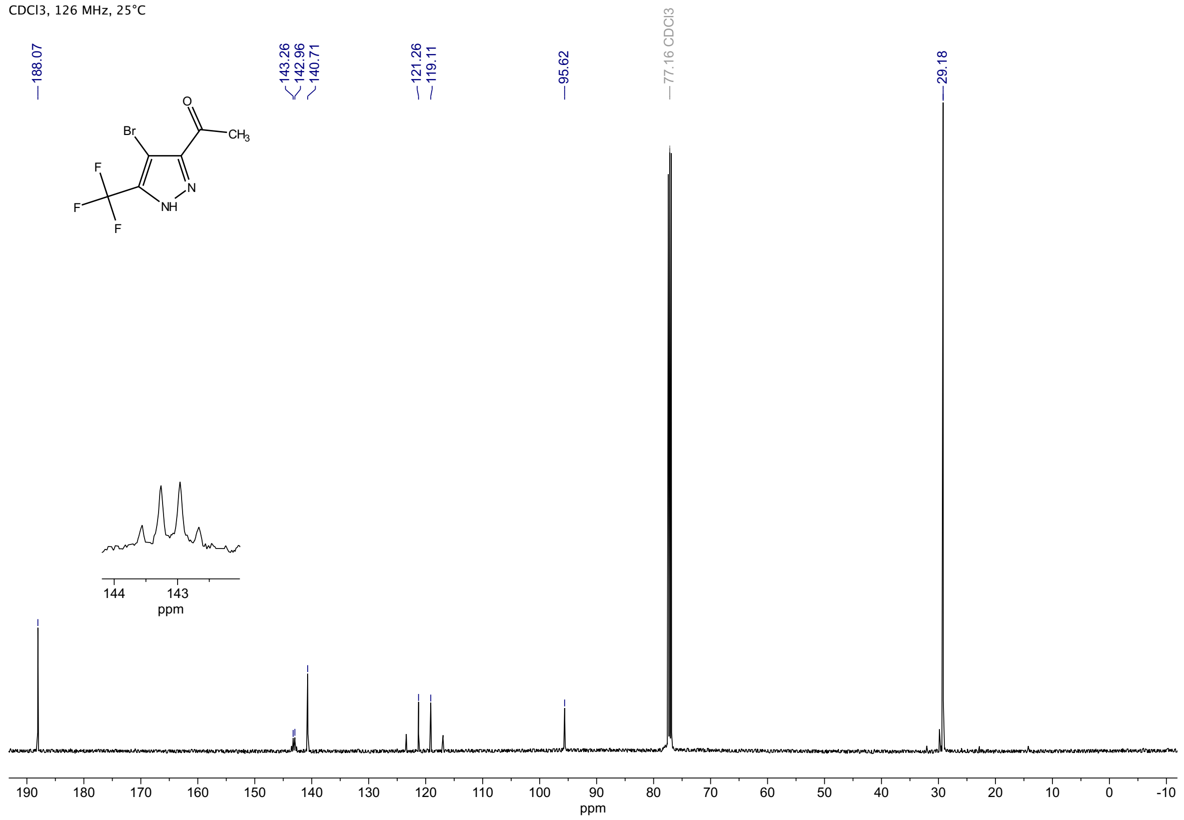

^{19}F NMR of 1-(4-bromo-5-(trifluoromethyl)-*1H*-pyrazol-3-yl)ethan-1-one (**6j**)

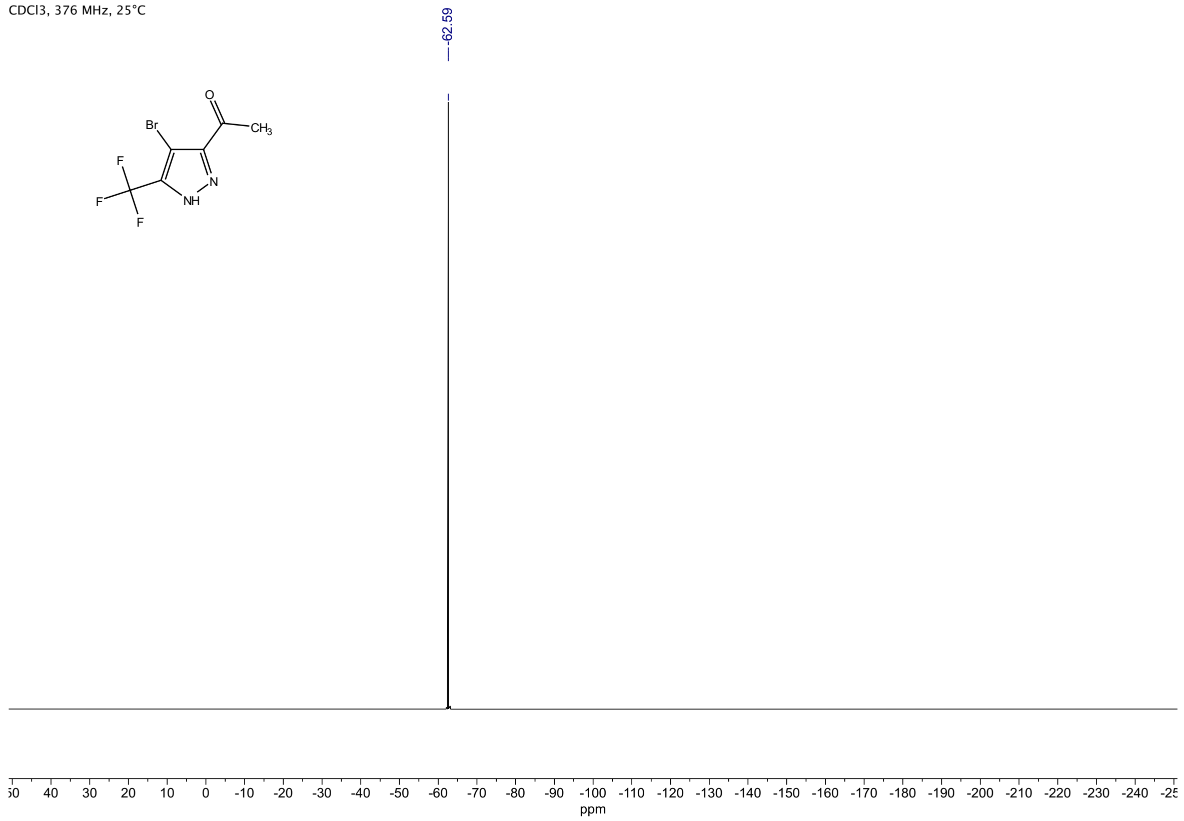

^{13}C NMR of 4-bromo-5-(trifluoromethyl)-*1H*-pyrazole-3-carbonitrile (**6k**)

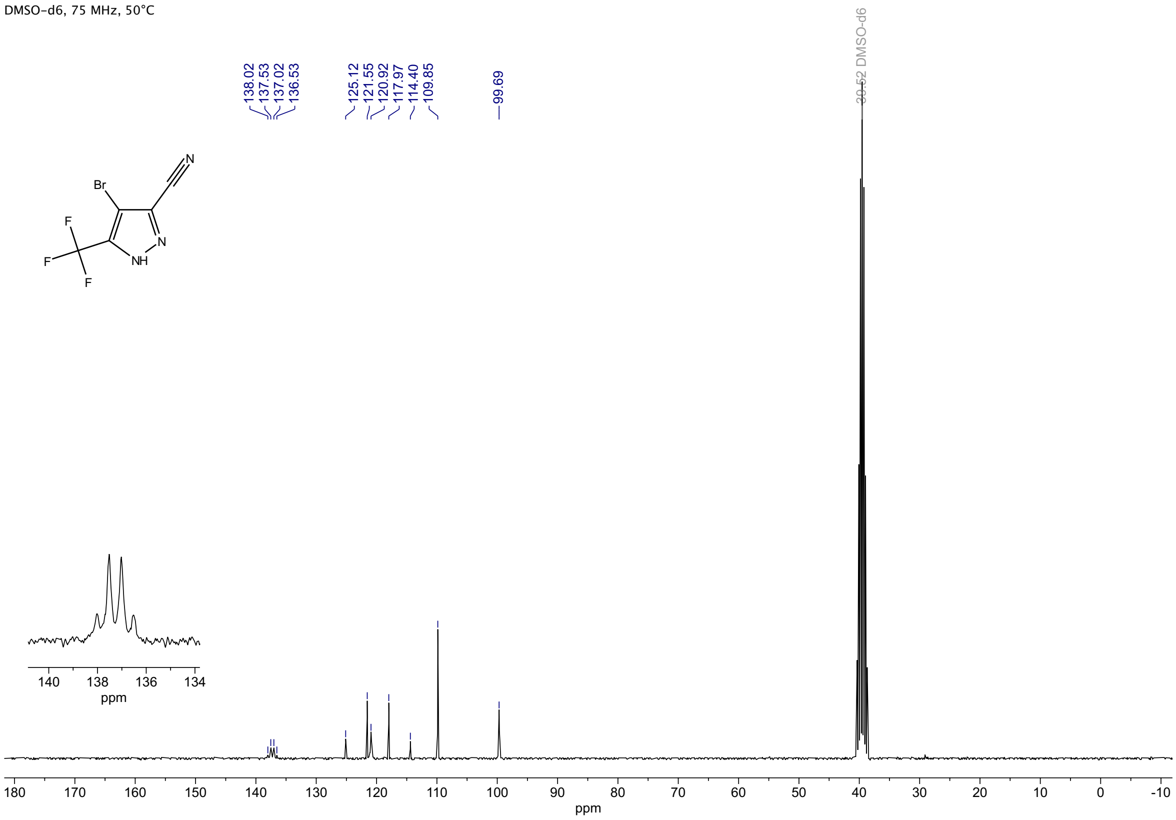

^{19}F NMR of 4-bromo-5-(trifluoromethyl)-*1H*-pyrazole-3-carbonitrile (**6k**)

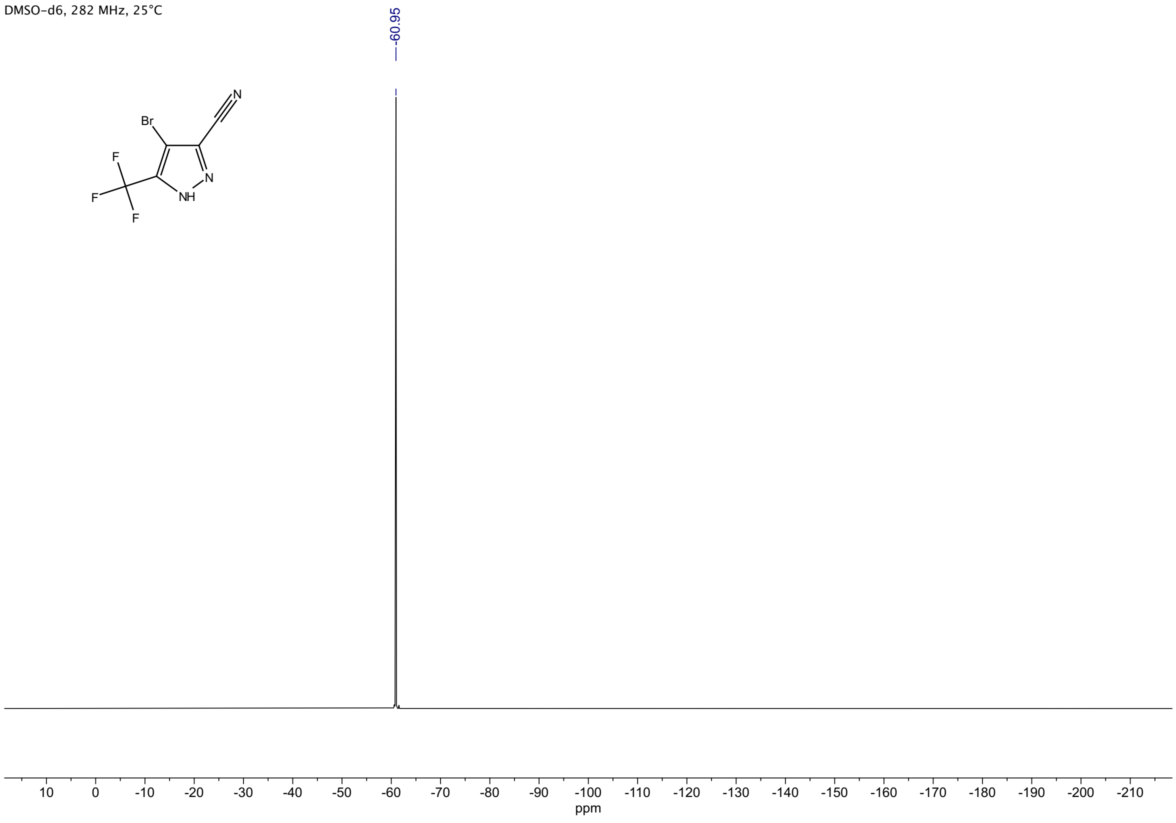

^{1}H NMR of 1-(4-iodo-5-(trifluoromethyl)-*1H*-pyrazol-3-yl)ethan-1-one (**6l**)

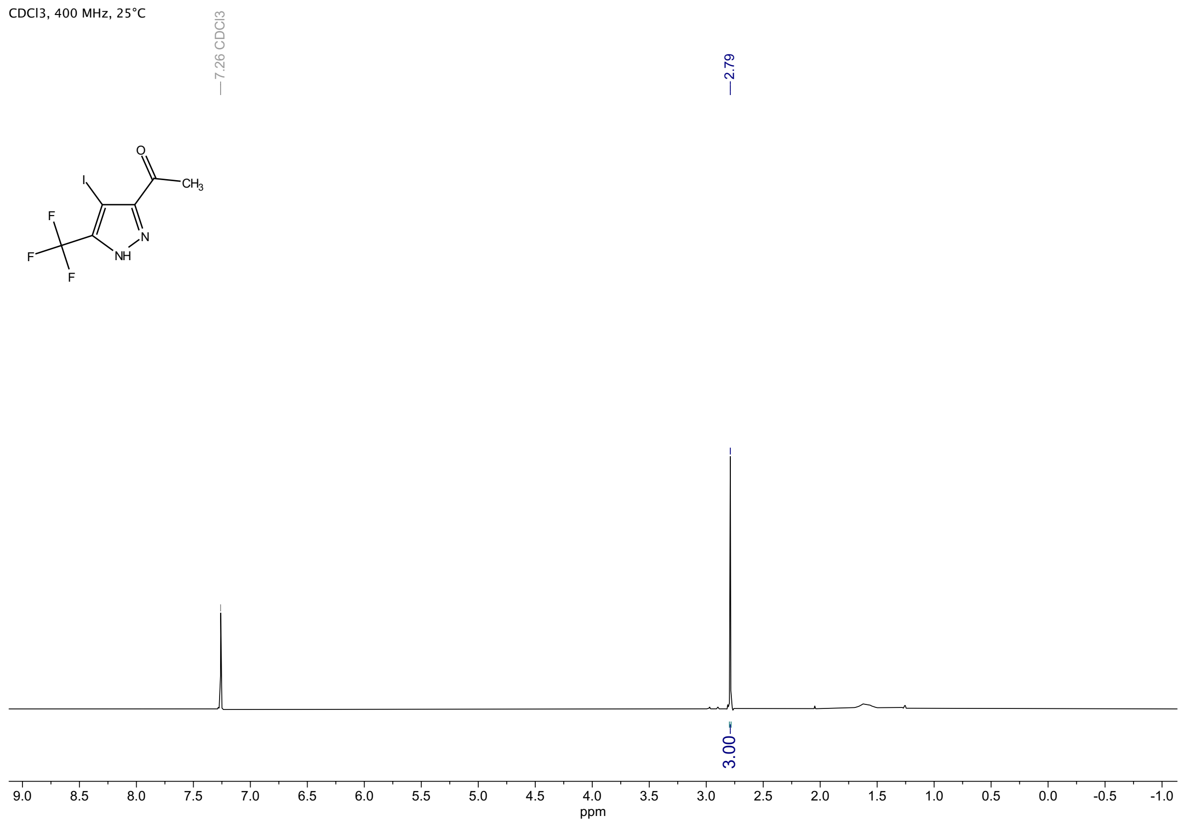

^{13}C NMR of 1-(4-iodo-5-(trifluoromethyl)-*1H*-pyrazol-3-yl)ethan-1-one (**6l**)

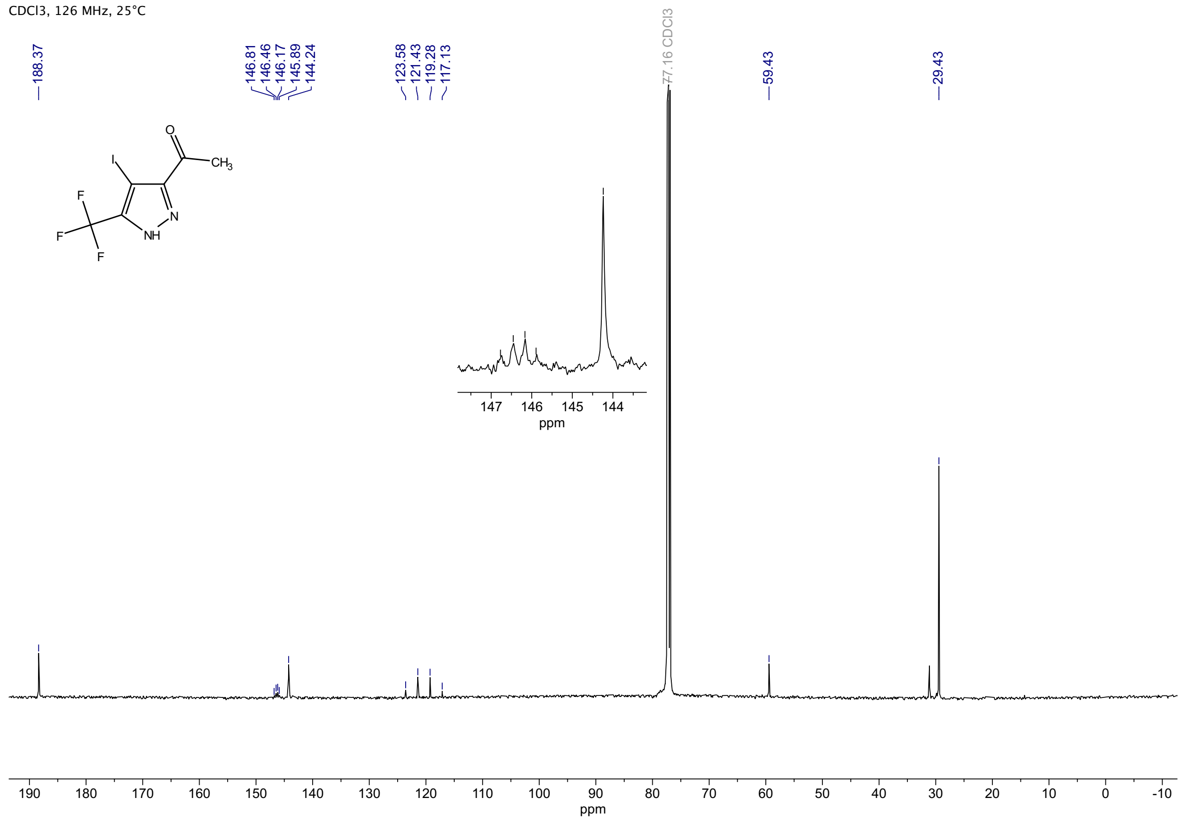

^{19}F NMR of 1-(4-iodo-5-(trifluoromethyl)-*1H*-pyrazol-3-yl)ethan-1-one (**6l**)

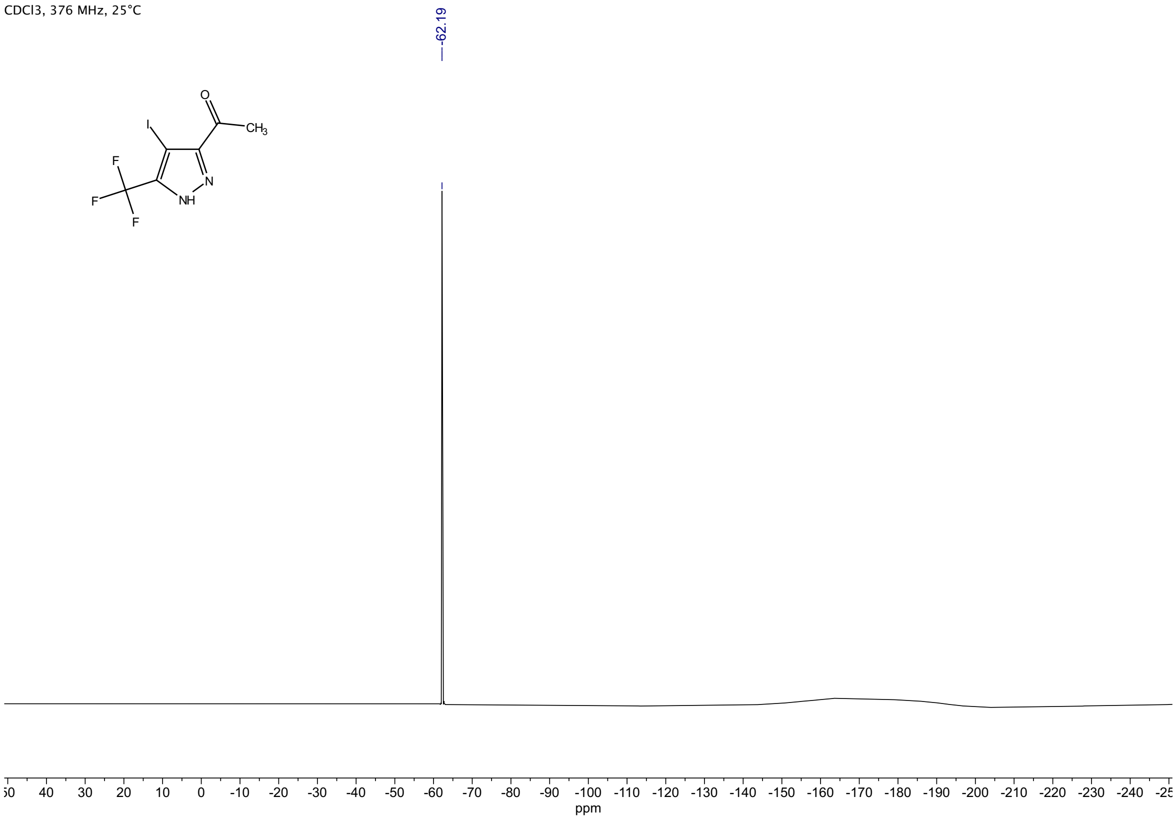

^{1}H NMR of methyl 4-iodo-5-(trifluoromethyl)-*1H*-pyrazole-3-carboxylate (**6m**)

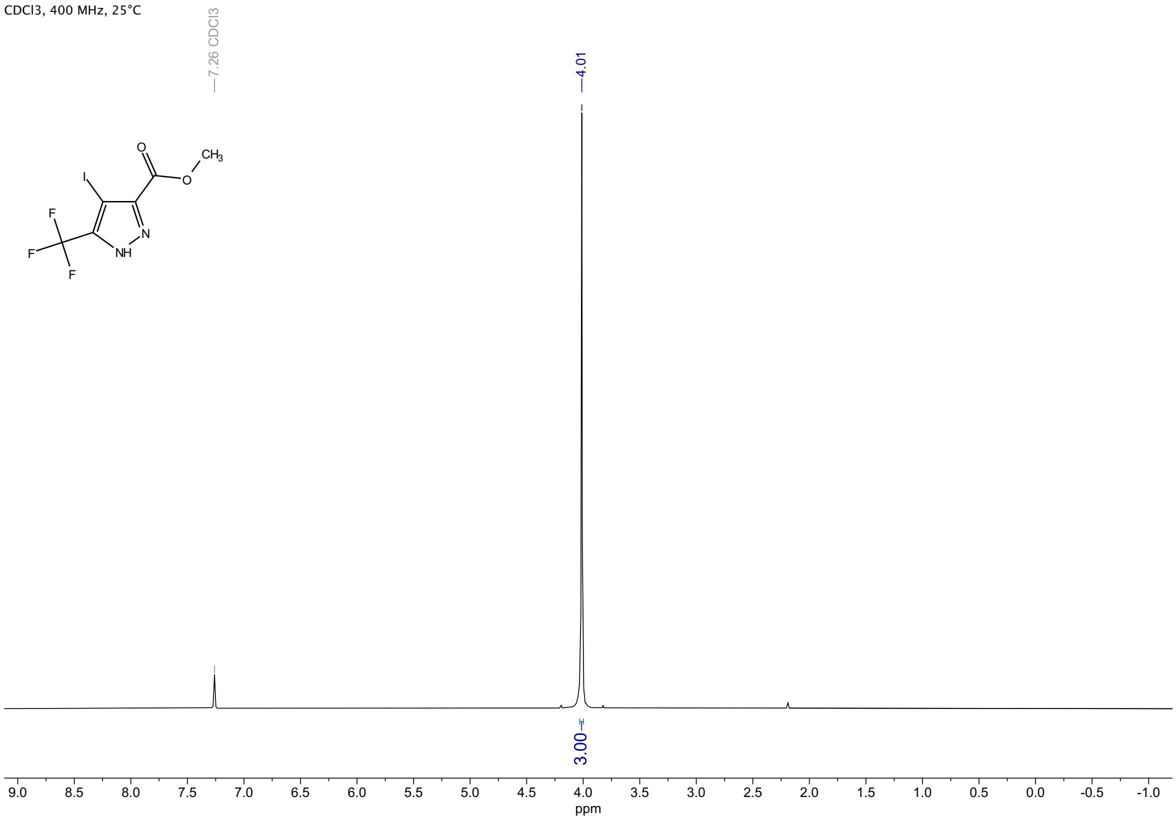

^{13}C NMR of methyl 4-iodo-5-(trifluoromethyl)-*1H*-pyrazole-3-carboxylate (**6m**)

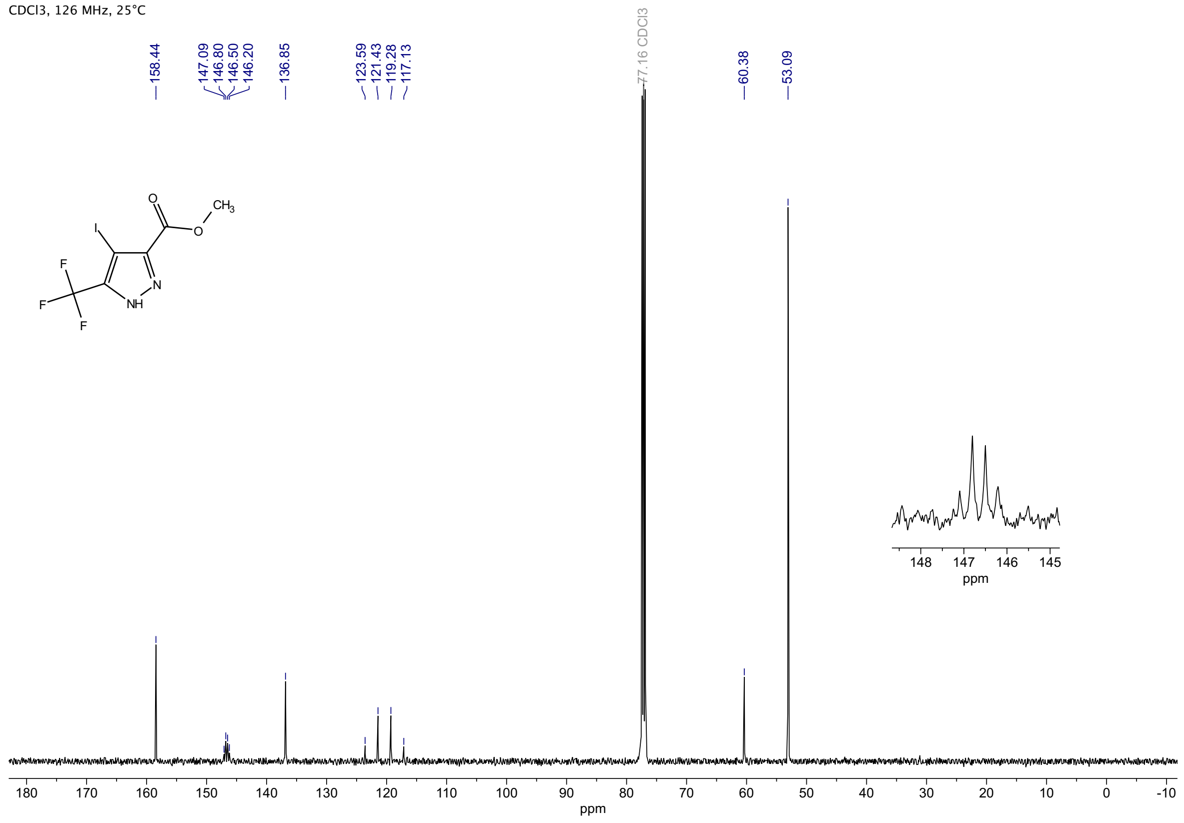

^{19}F NMR of methyl 4-iodo-5-(trifluoromethyl)-*1H*-pyrazole-3-carboxylate (**6m**)

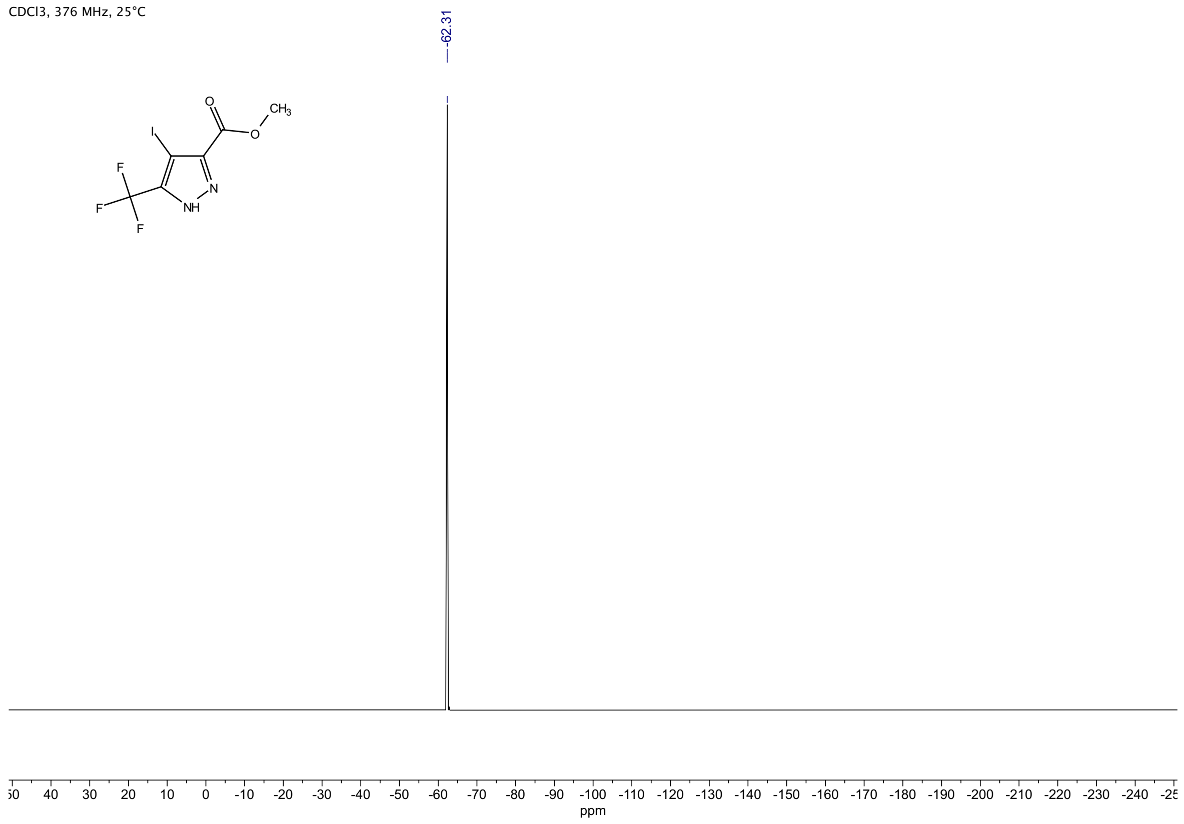

^{1}H NMR of ethyl 4-cyano-5-(trifluoromethyl)-*1H*-pyrazole-3-carboxylate (**6n**)

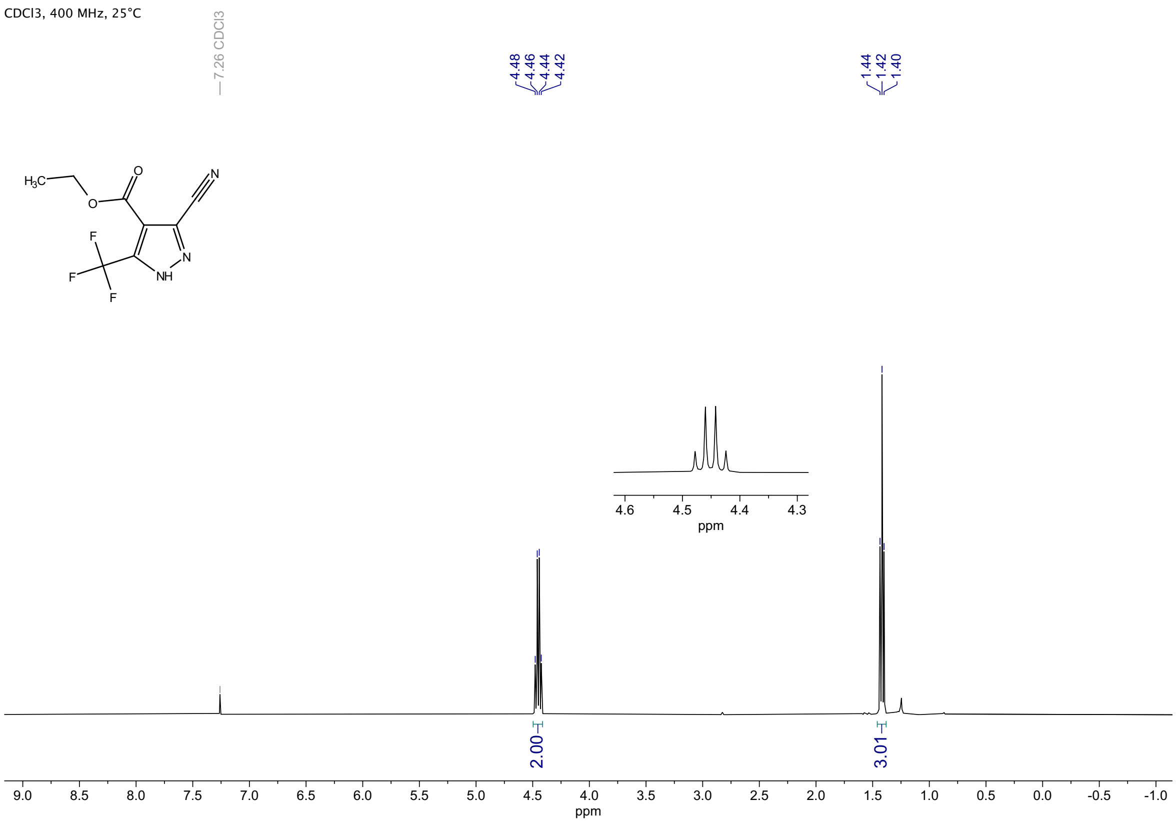

^{13}C NMR of ethyl 4-cyano-5-(trifluoromethyl)-*1H*-pyrazole-3-carboxylate (**6n**)

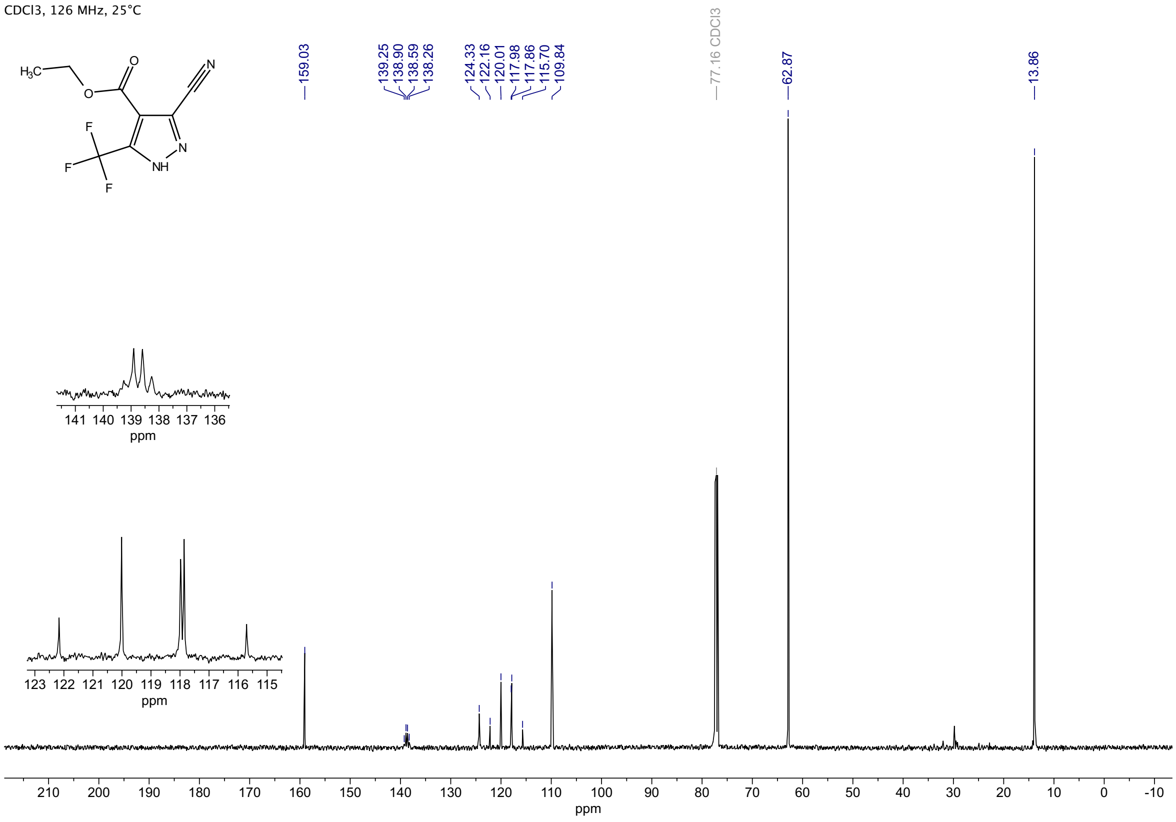

^{19}F NMR of ethyl 4-cyano-5-(trifluoromethyl)-*1H*-pyrazole-3-carboxylate (**6n**)

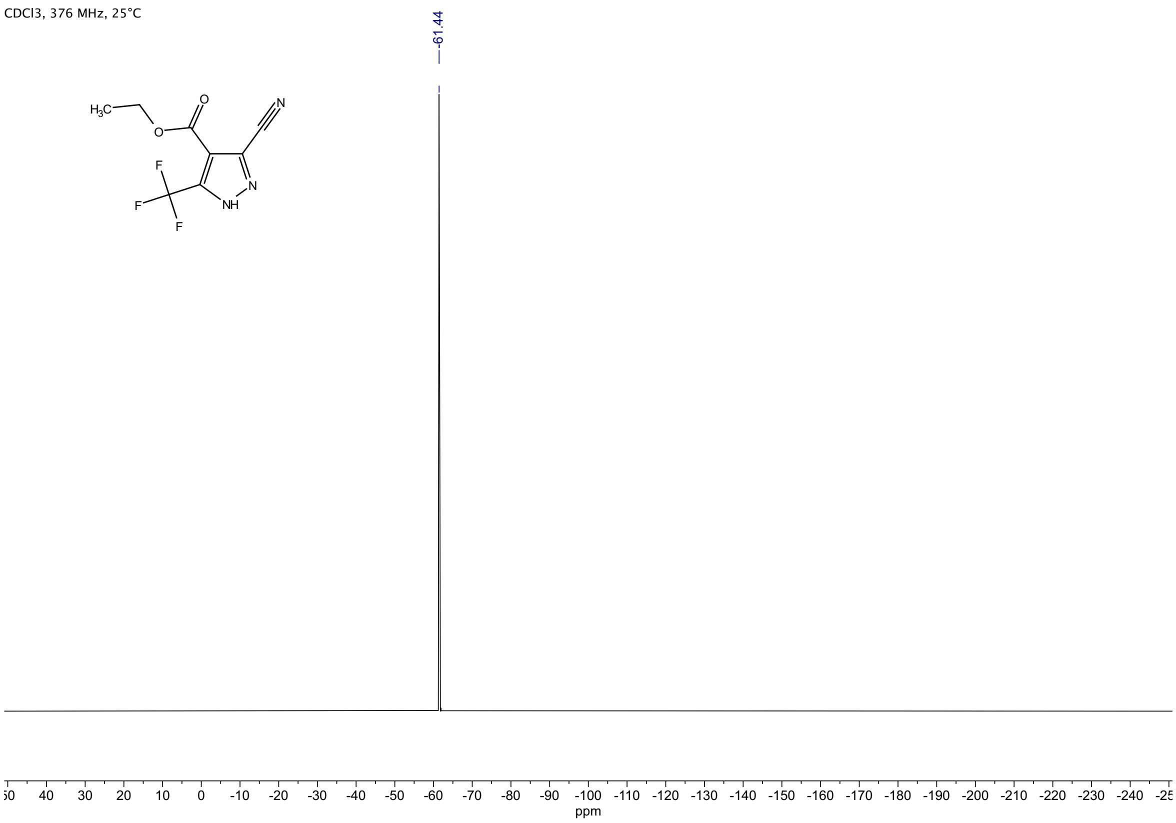

^{1}H NMR of 3-(tert-butyl)-5-(trifluoromethyl)-*1H*-pyrazole-4-carbonitrile (**6o**)

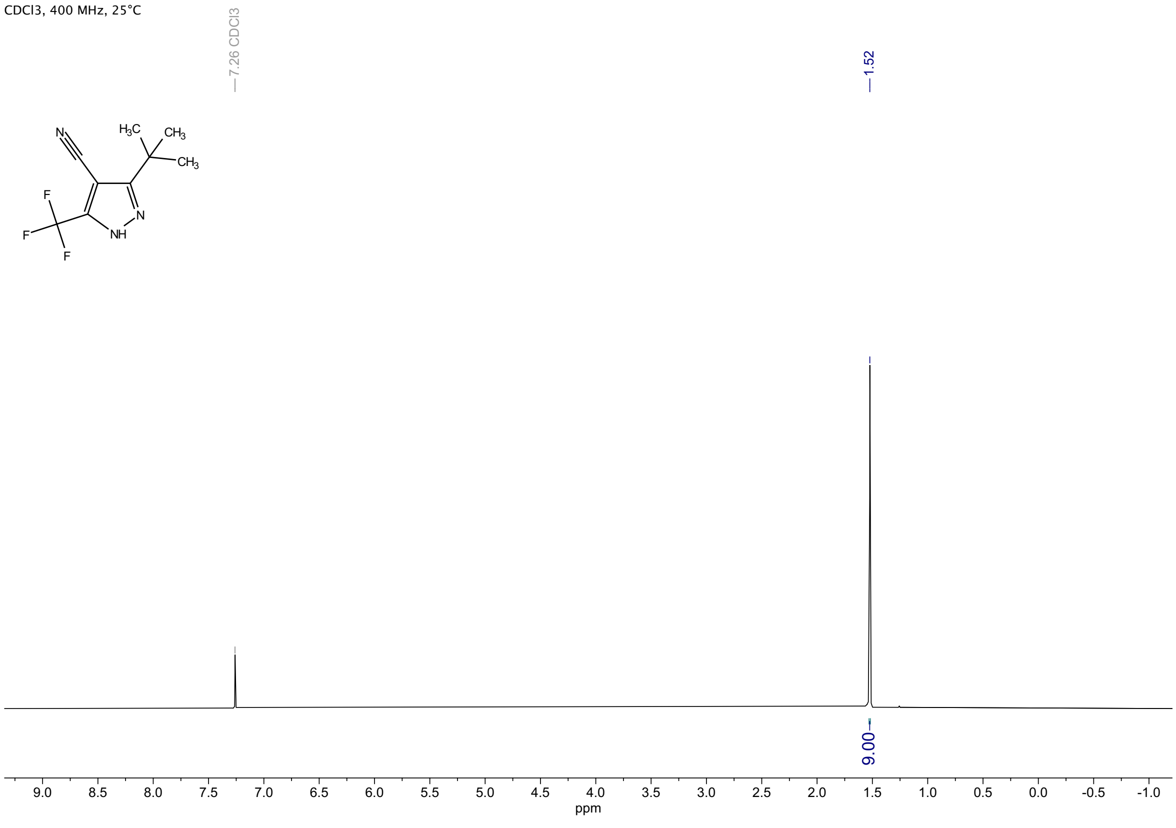

^{13}C NMR of 3-(tert-butyl)-5-(trifluoromethyl)-*1H*-pyrazole-4-carbonitrile (**6o**)

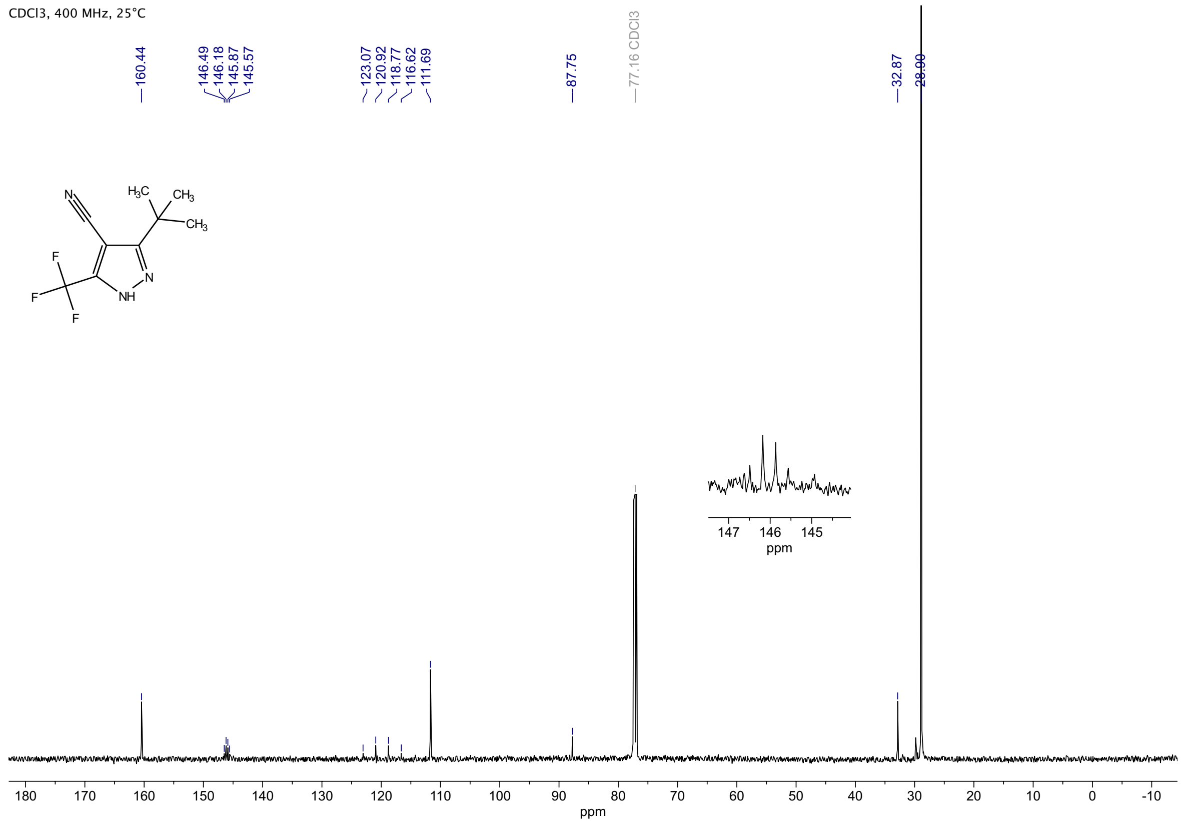

^{19}F NMR of 3-(tert-butyl)-5-(trifluoromethyl)-*1H*-pyrazole-4-carbonitrile (**6o**)

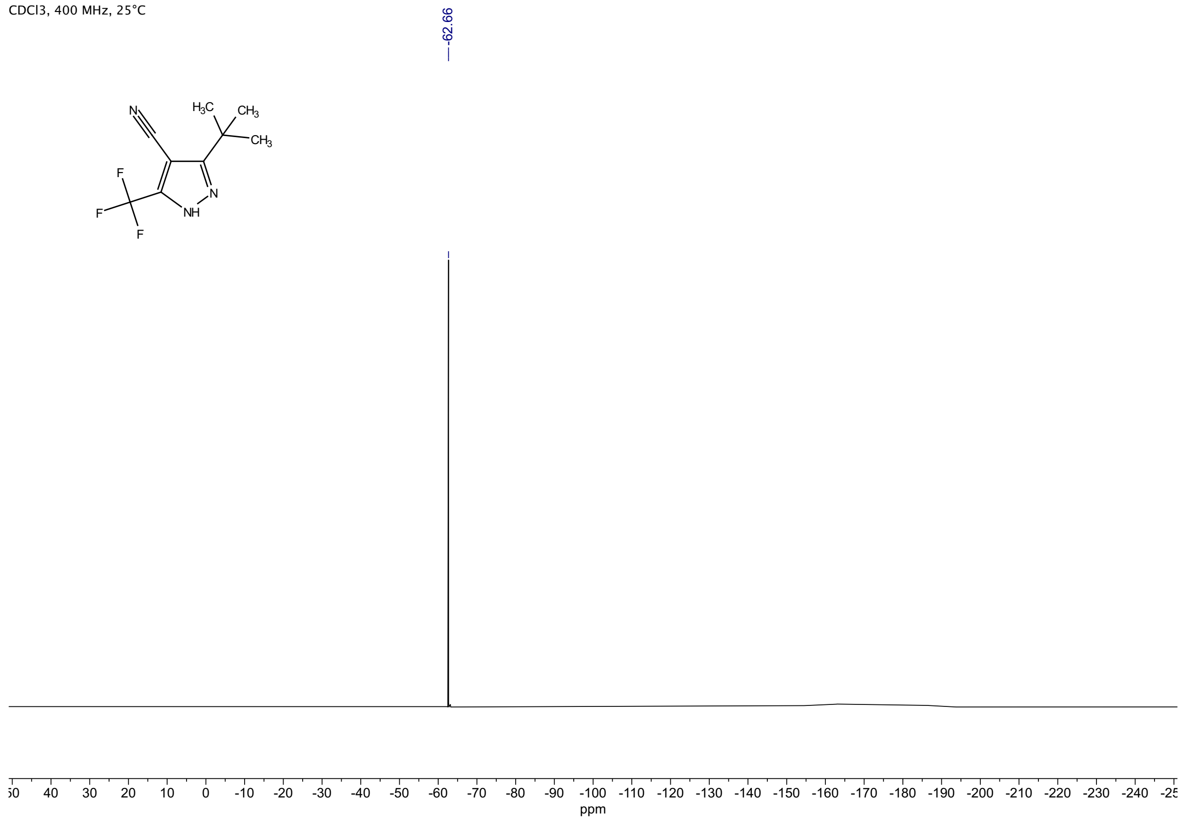

^{1}H NMR of 3-methyl-5-(trifluoromethyl)-*1H*-pyrazole-4-carboxylic acid (**6p**)

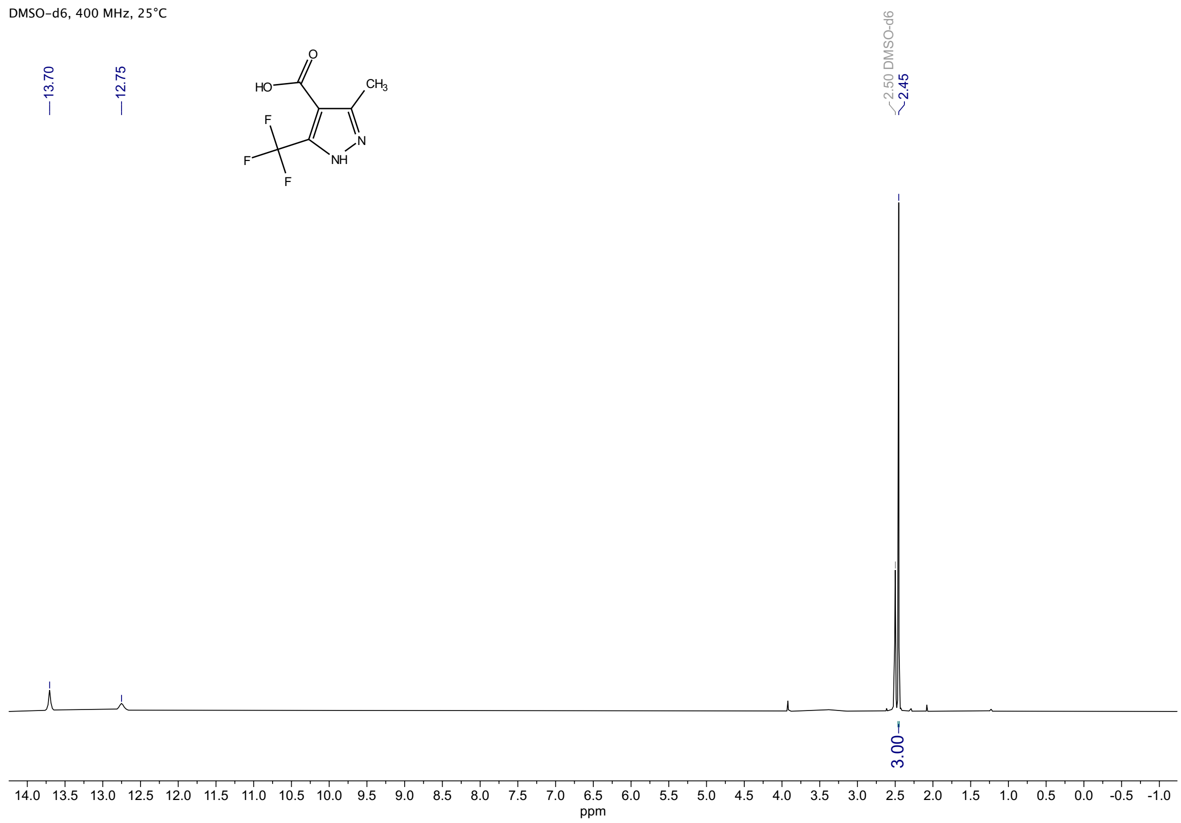

^{13}C NMR of 3-methyl-5-(trifluoromethyl)-*1H*-pyrazole-4-carboxylic acid (**6p**)

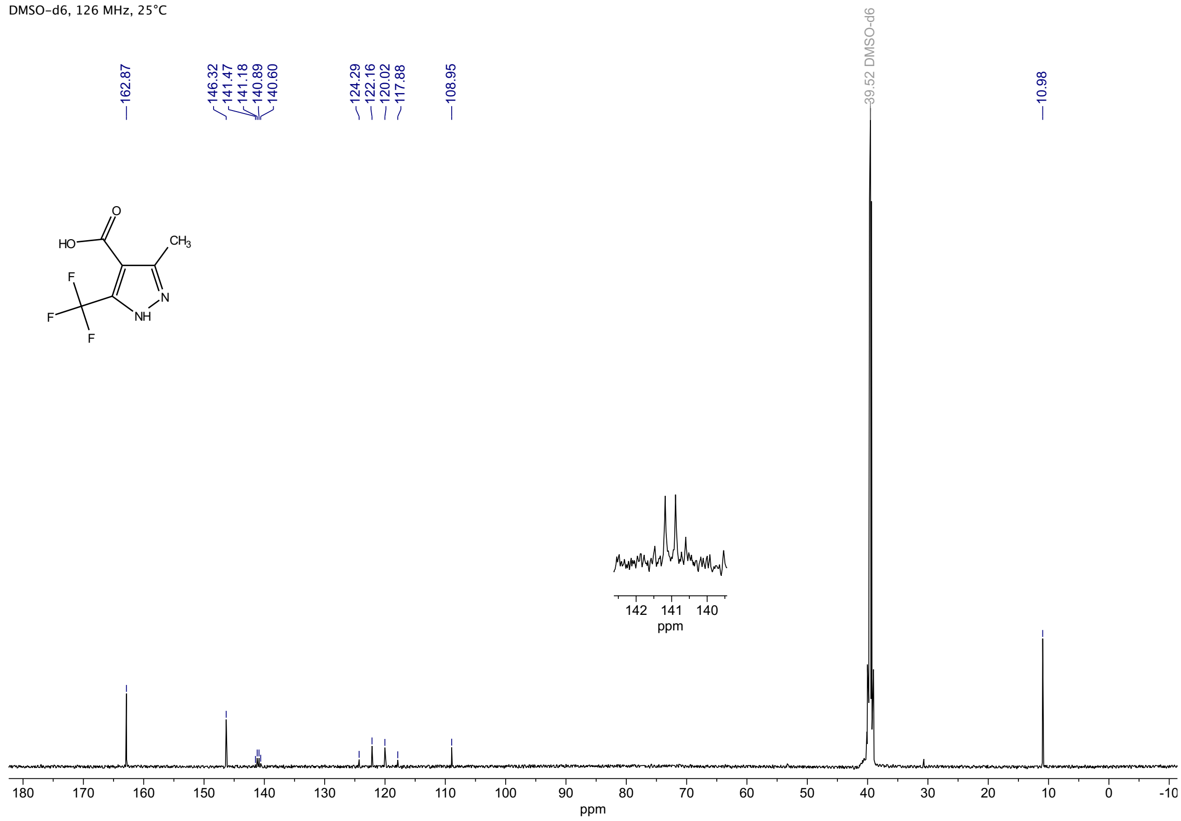

^{19}F NMR of 3-methyl-5-(trifluoromethyl)-*1H*-pyrazole-4-carboxylic acid (**6p**)

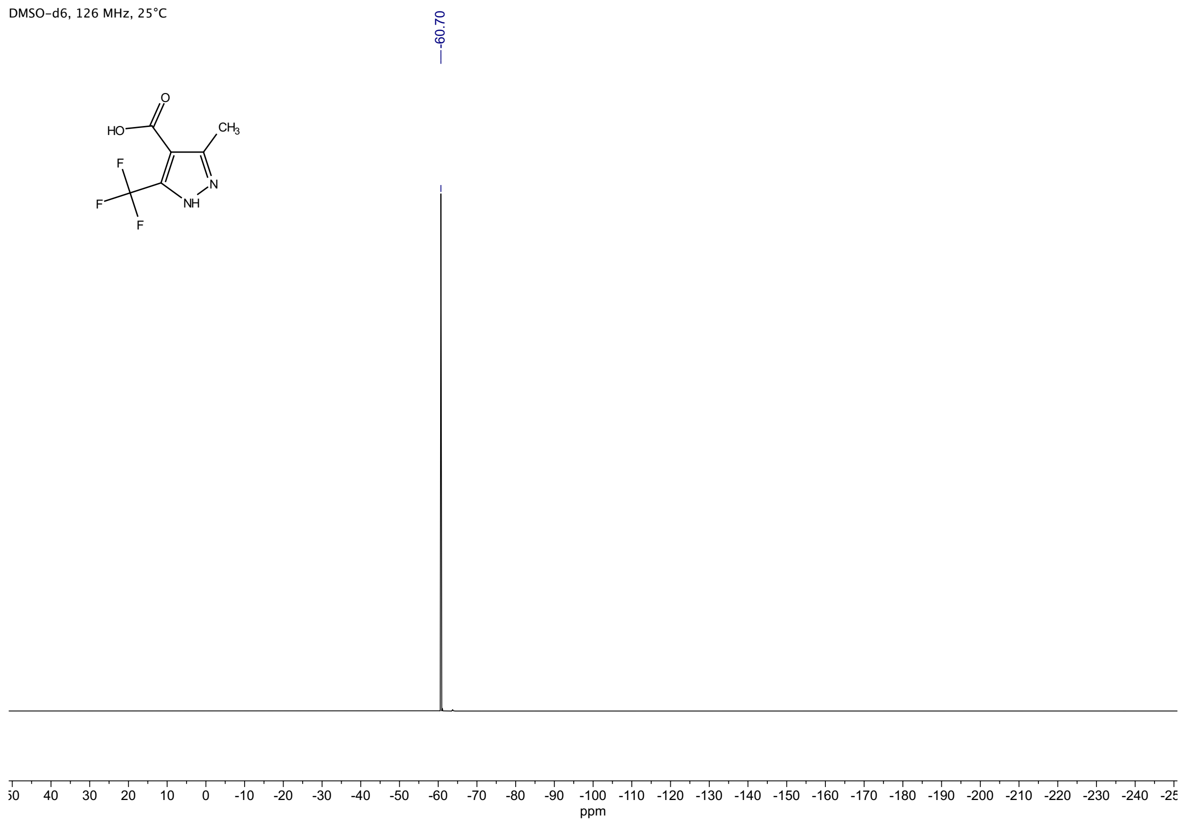

^{1}H NMR of 1-(3-methyl-5-(trifluoromethyl)-*1H*-pyrazol-4-yl)ethan-1-one (**6q**)

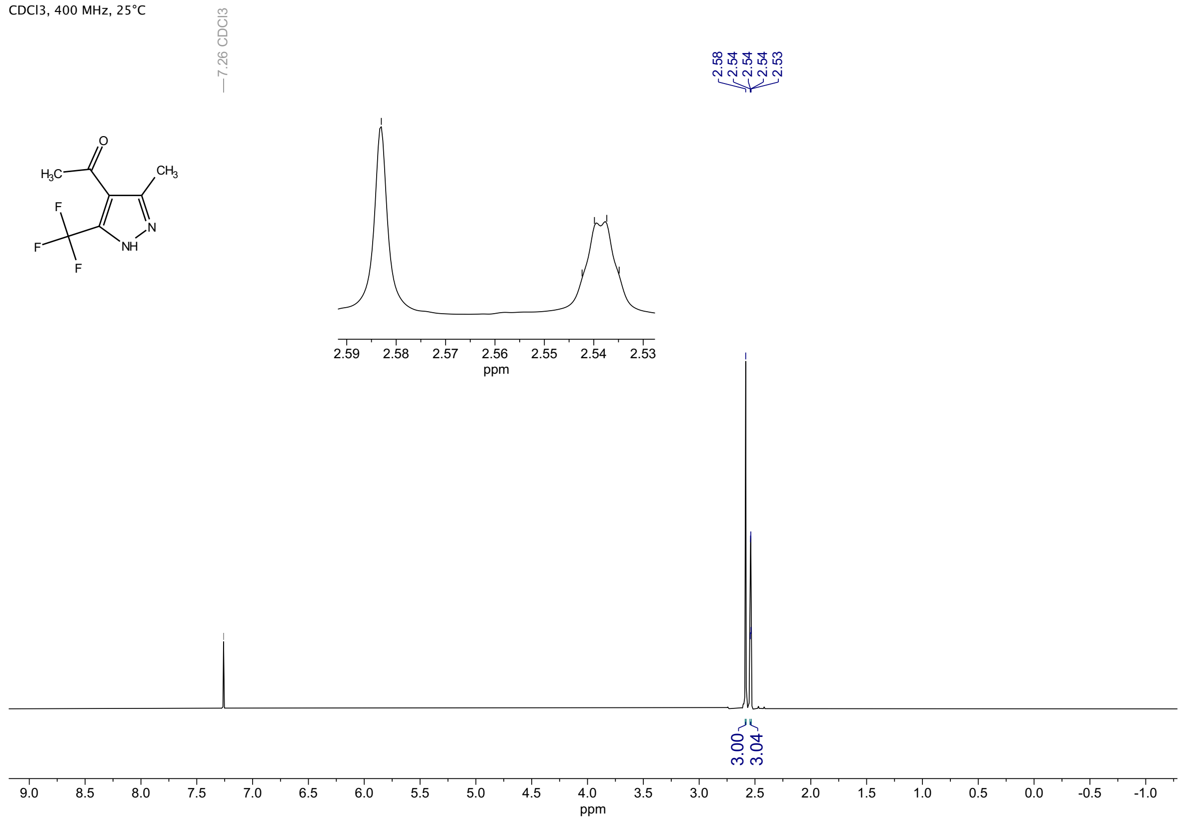

^{13}C NMR of 1-(3-methyl-5-(trifluoromethyl)-*1H*-pyrazol-4-yl)ethan-1-one (**6q**)

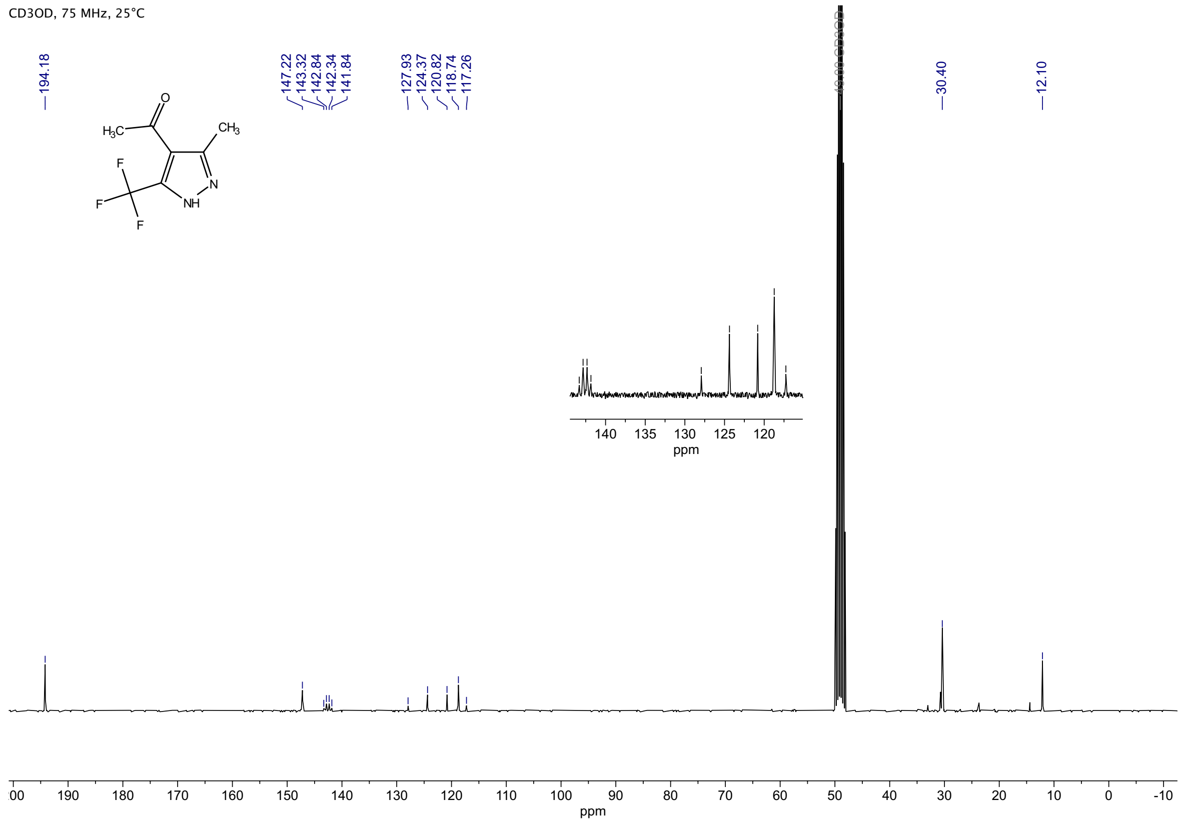

^{19}F NMR of 1-(3-methyl-5-(trifluoromethyl)-*1H*-pyrazol-4-yl)ethan-1-one (**6q**)

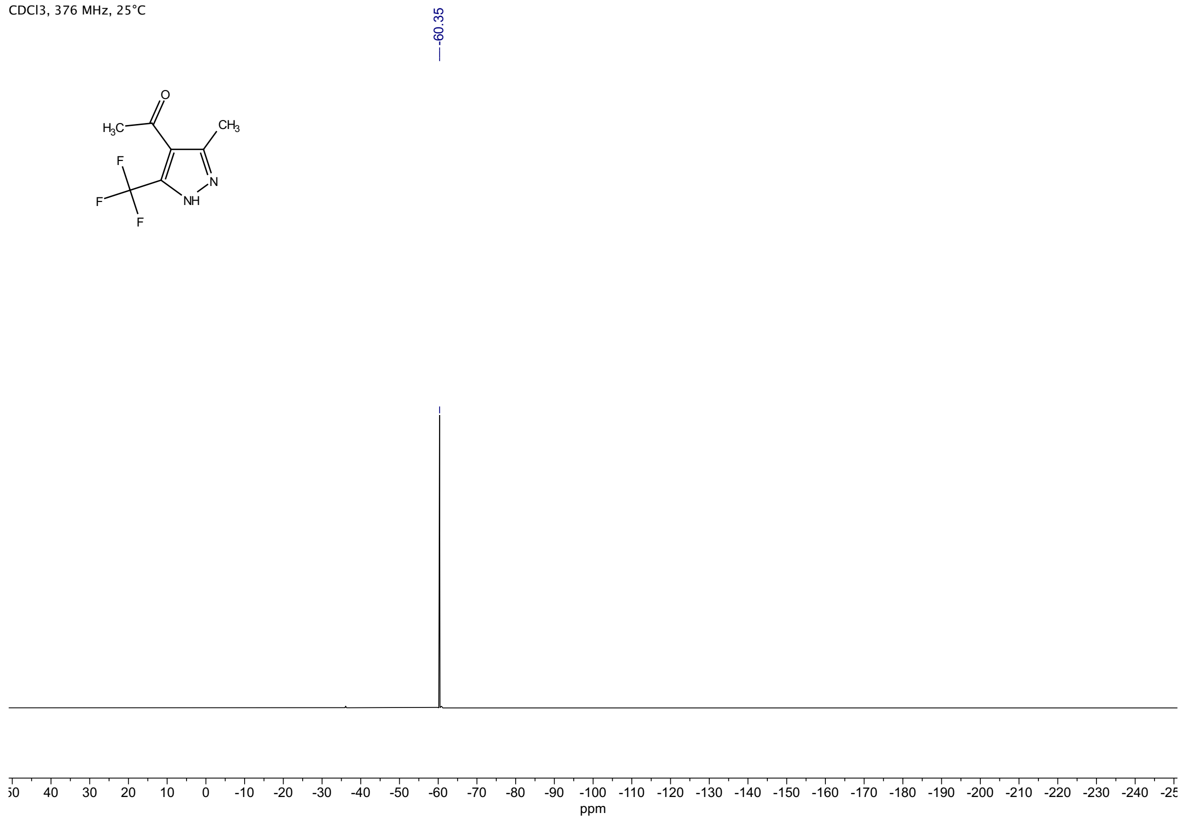

^{1}H NMR of 5-cyclopropyl-4-nitro-3-(trifluoromethyl)-*1H*-pyrazole (**6r**)

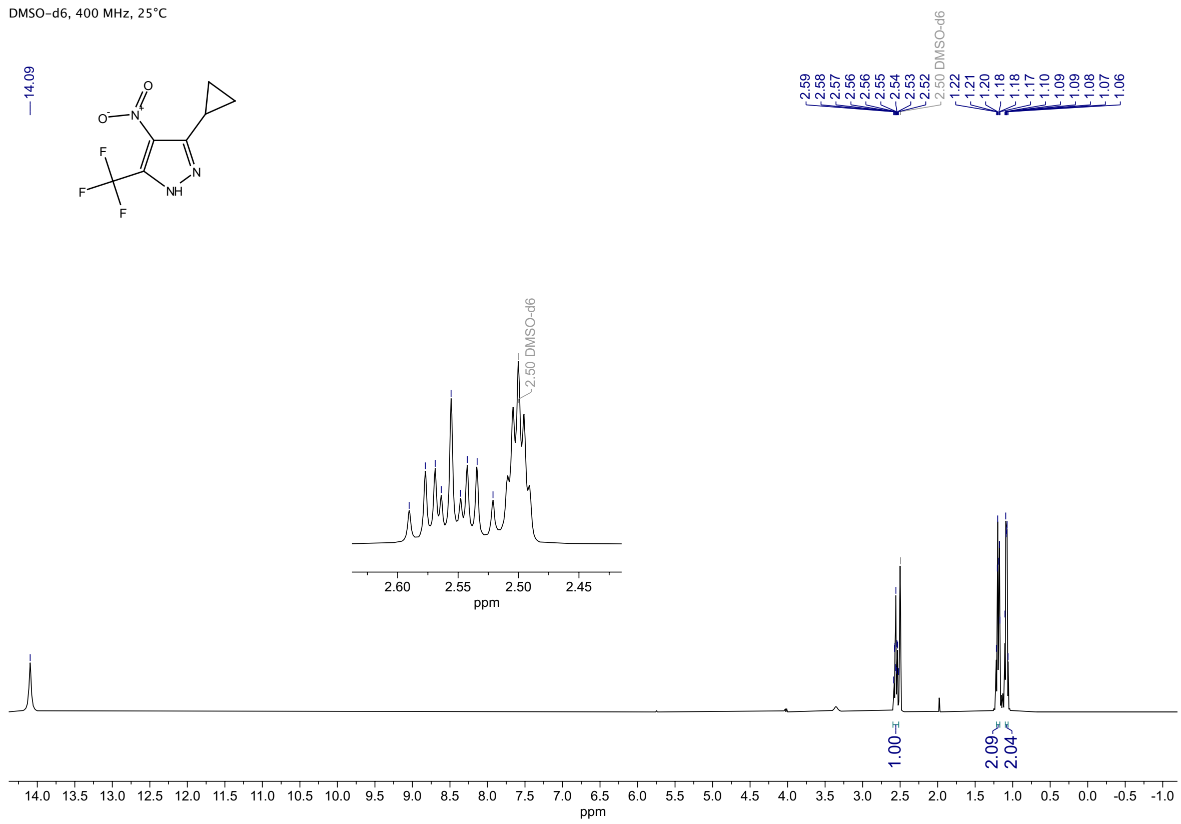

^{13}C NMR of 5-cyclopropyl-4-nitro-3-(trifluoromethyl)-*1H*-pyrazole (**6r**)

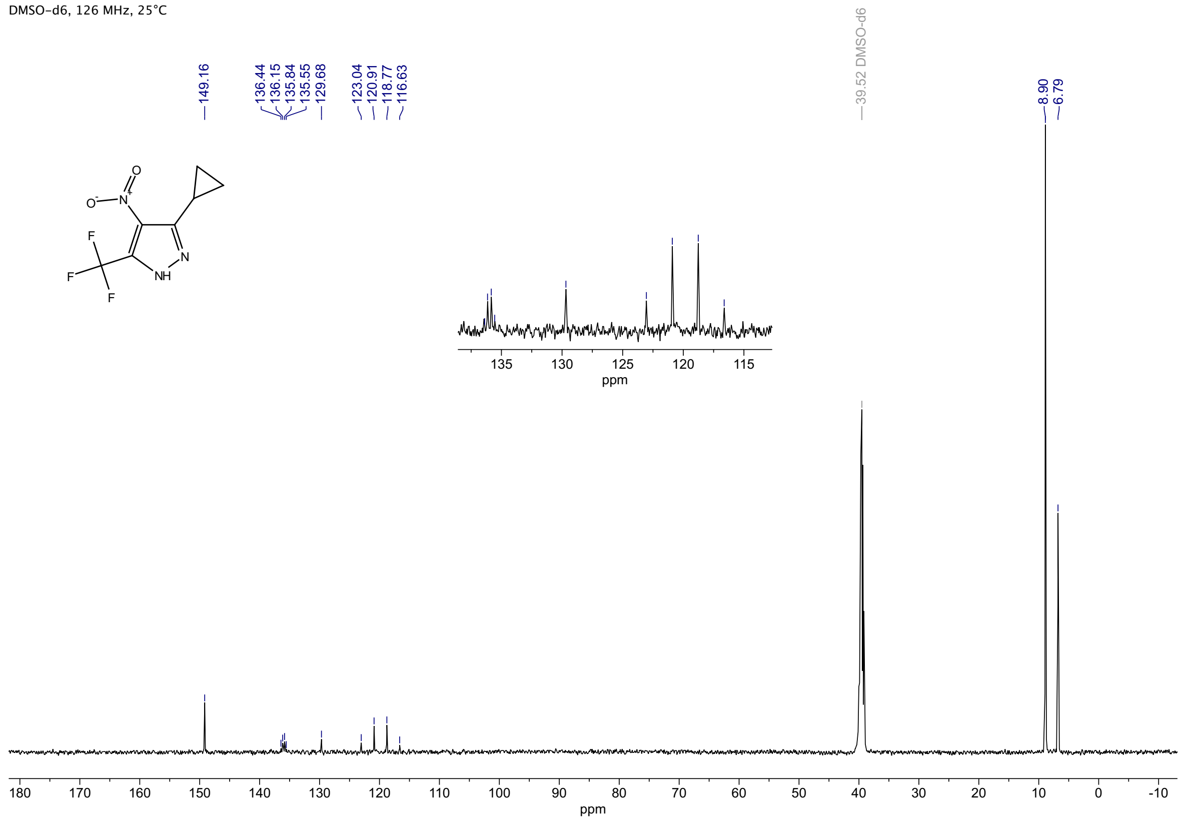

^{19}F NMR of 5-cyclopropyl-4-nitro-3-(trifluoromethyl)-*1H*-pyrazole (**6r**)

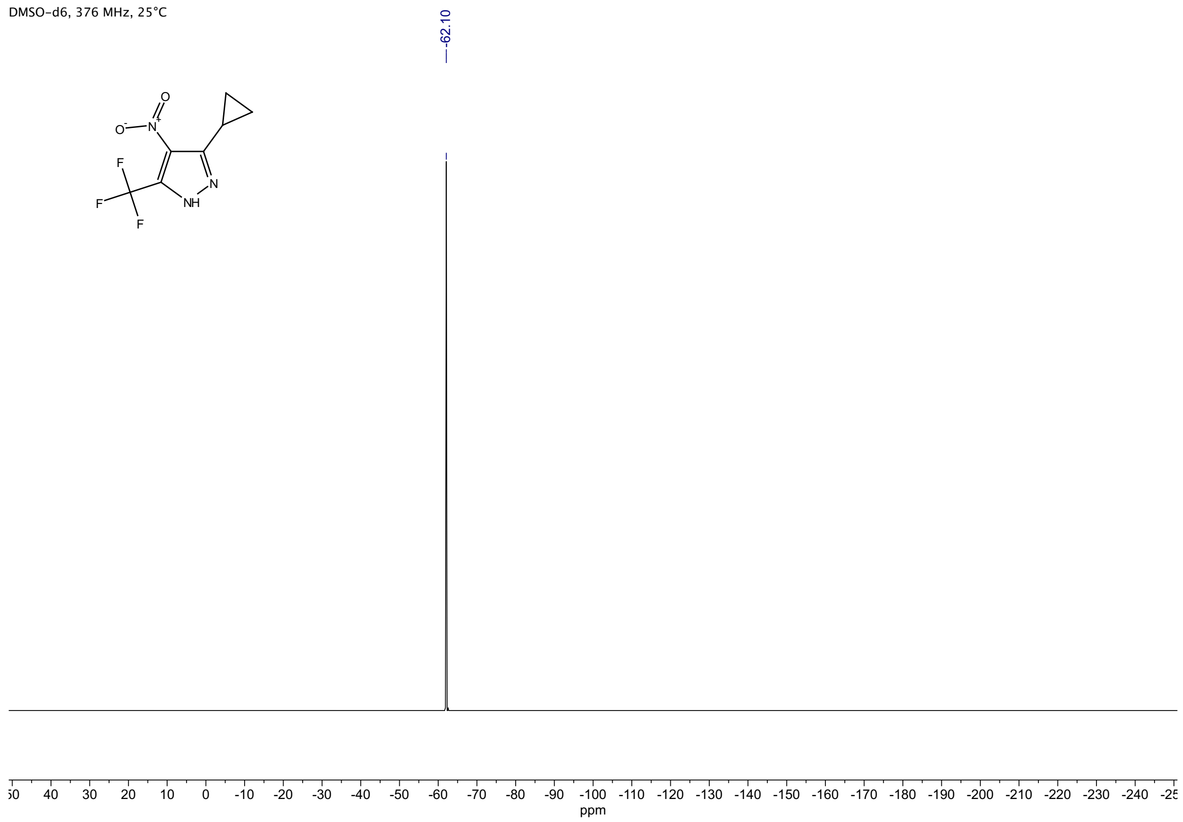

^{1}H NMR of 3-methyl-4-nitro-5-(trifluoromethyl)-*1H*-pyrazole (**6s**)

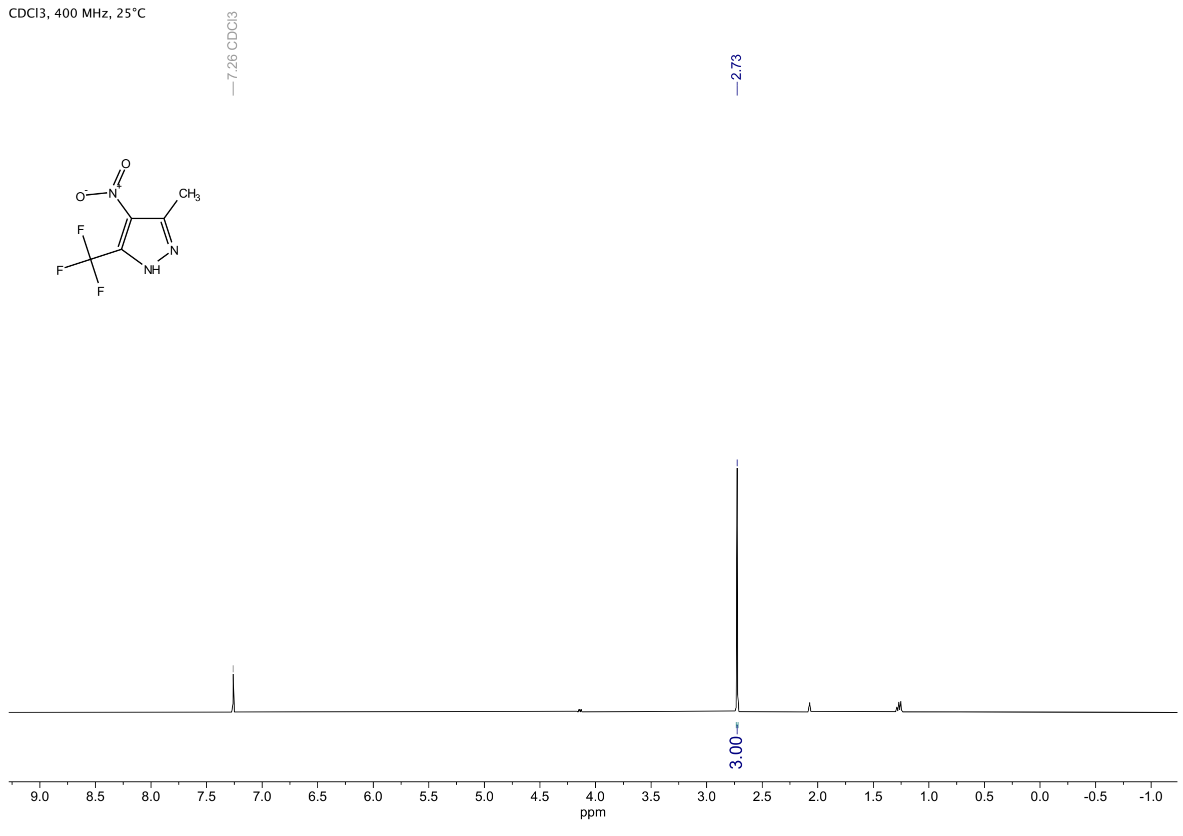

^{13}C NMR of 3-methyl-4-nitro-5-(trifluoromethyl)-*1H*-pyrazole (**6s**)

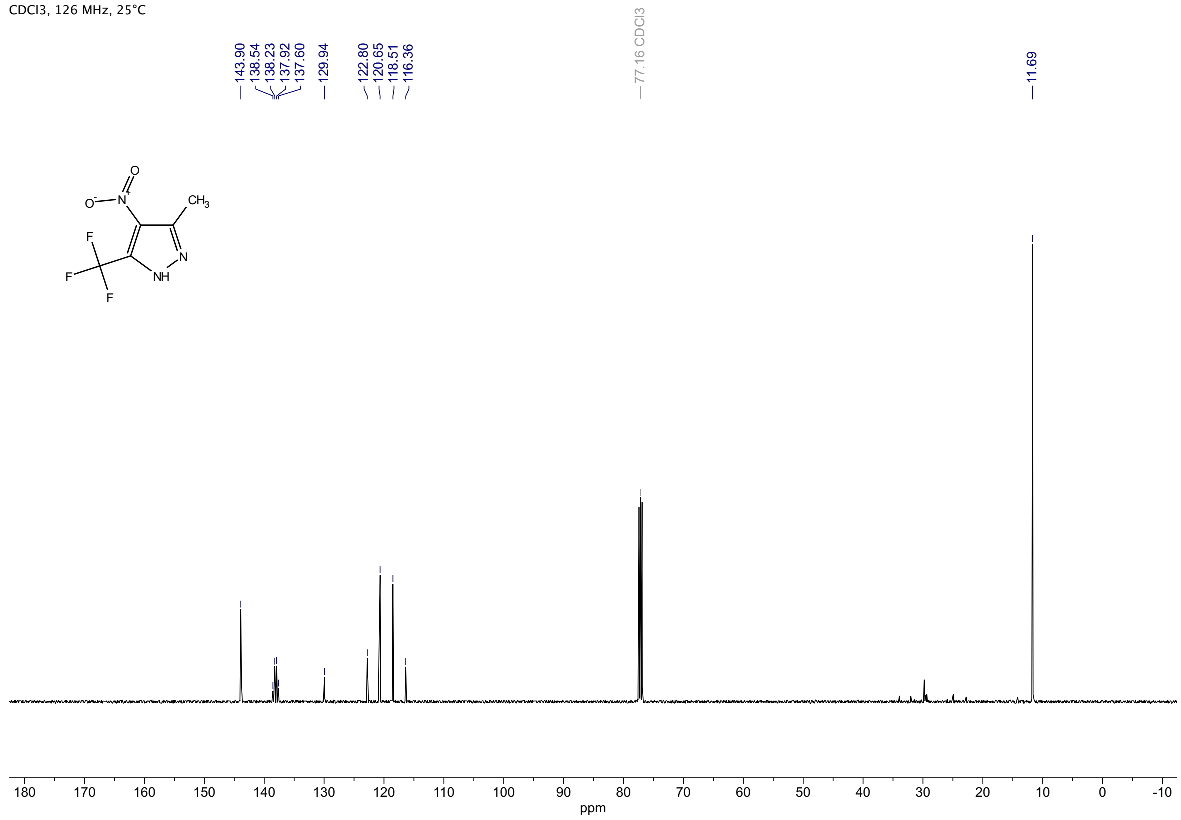

^{19}F NMR of 3-methyl-4-nitro-5-(trifluoromethyl)-*1H*-pyrazole (**6s**)

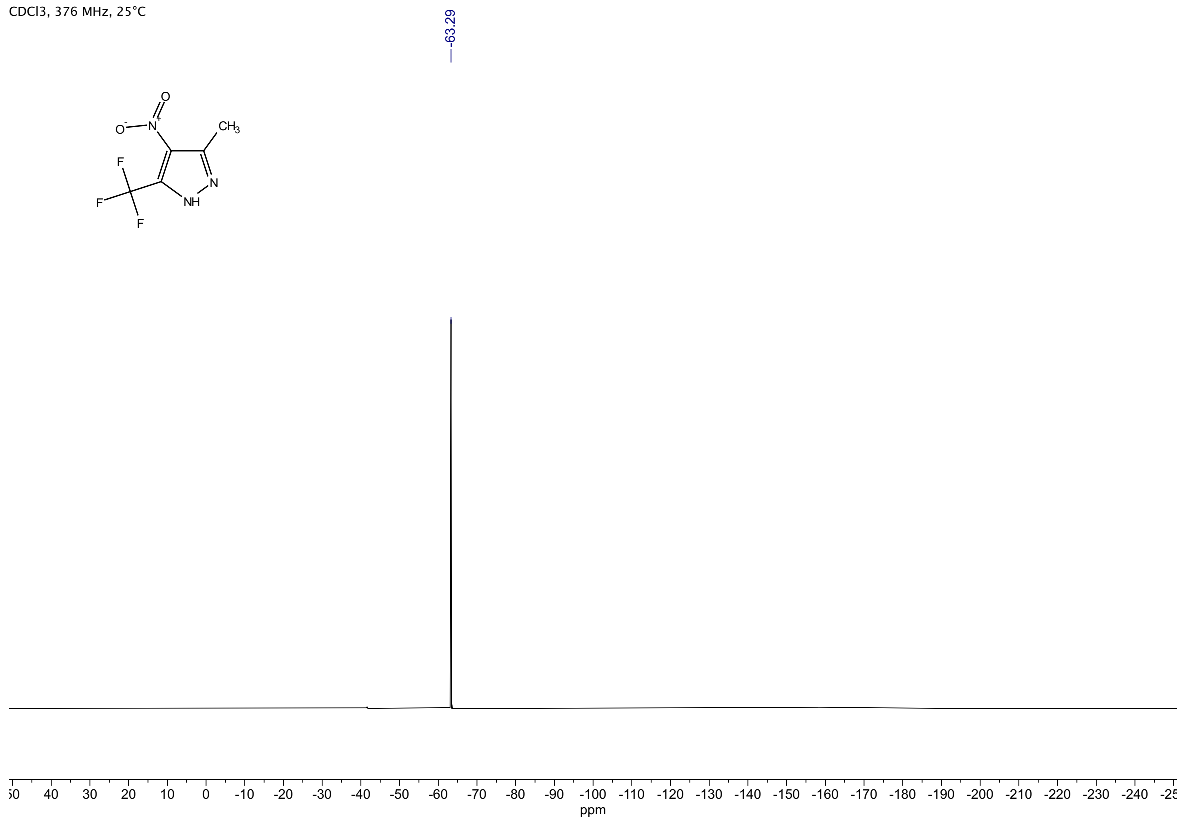

^{1}H NMR of 3-bromo-4-methyl-5-(trifluoromethyl)- *1H*-pyrazole (**6t**)

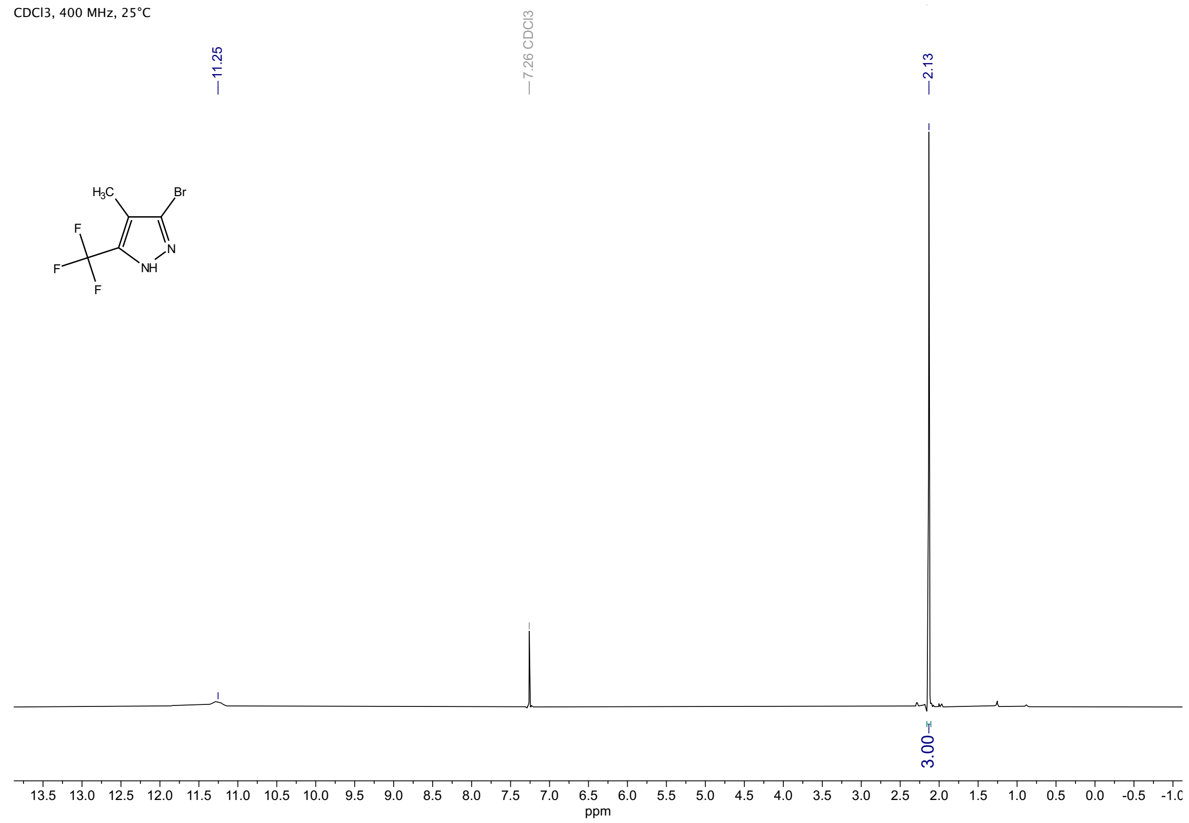

^{13}C NMR of 3-bromo-4-methyl-5-(trifluoromethyl)- *1H*-pyrazole (**6t**)

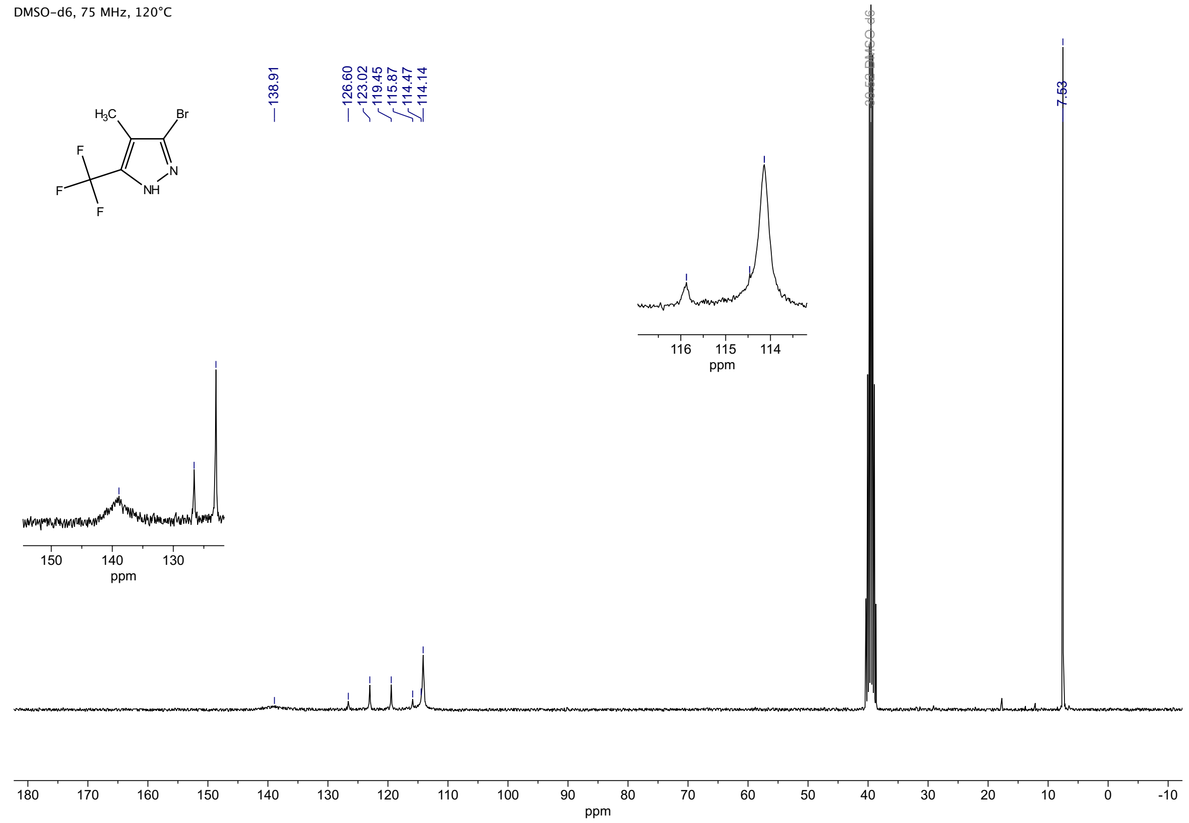

^{19}F NMR of 3-bromo-4-methyl-5-(trifluoromethyl)- *1H*-pyrazole (**6t**)

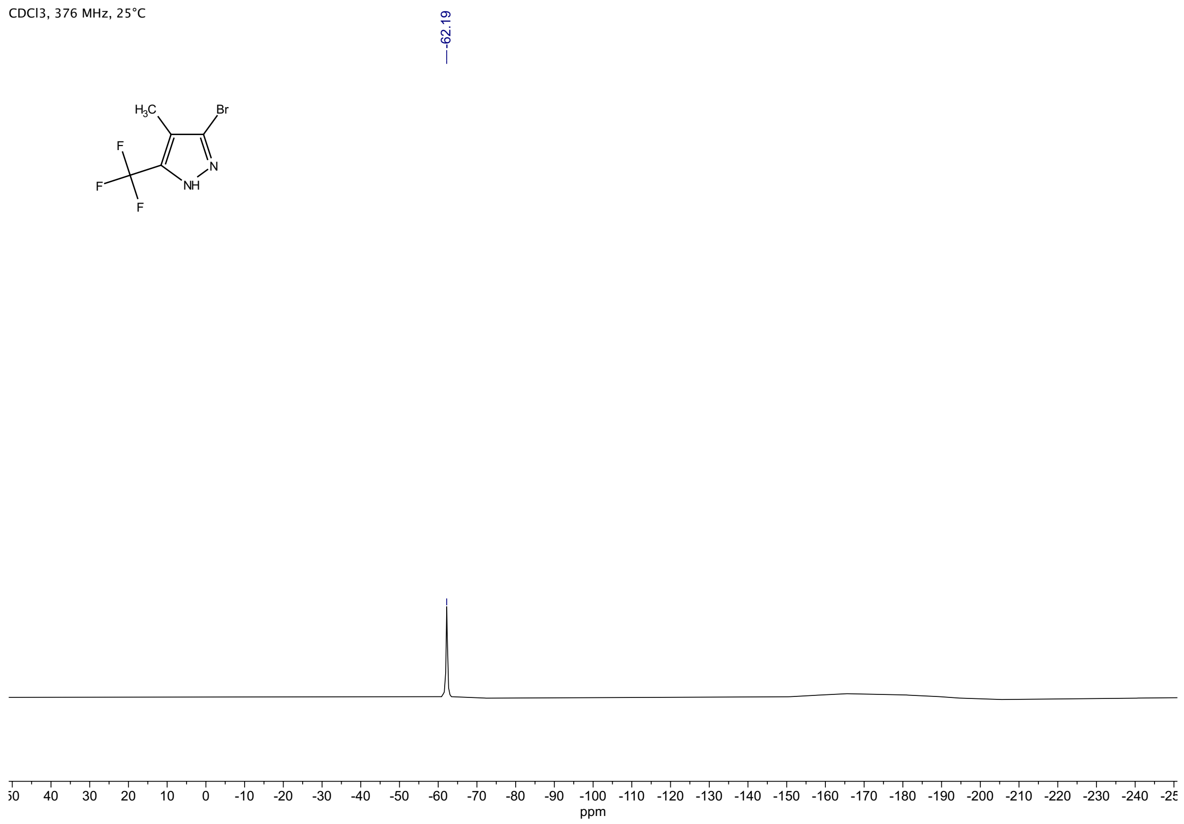

^{1}H NMR of 4-bromo-3-methyl-5-(trifluoromethyl)- *1H*-pyrazole (**6u**)

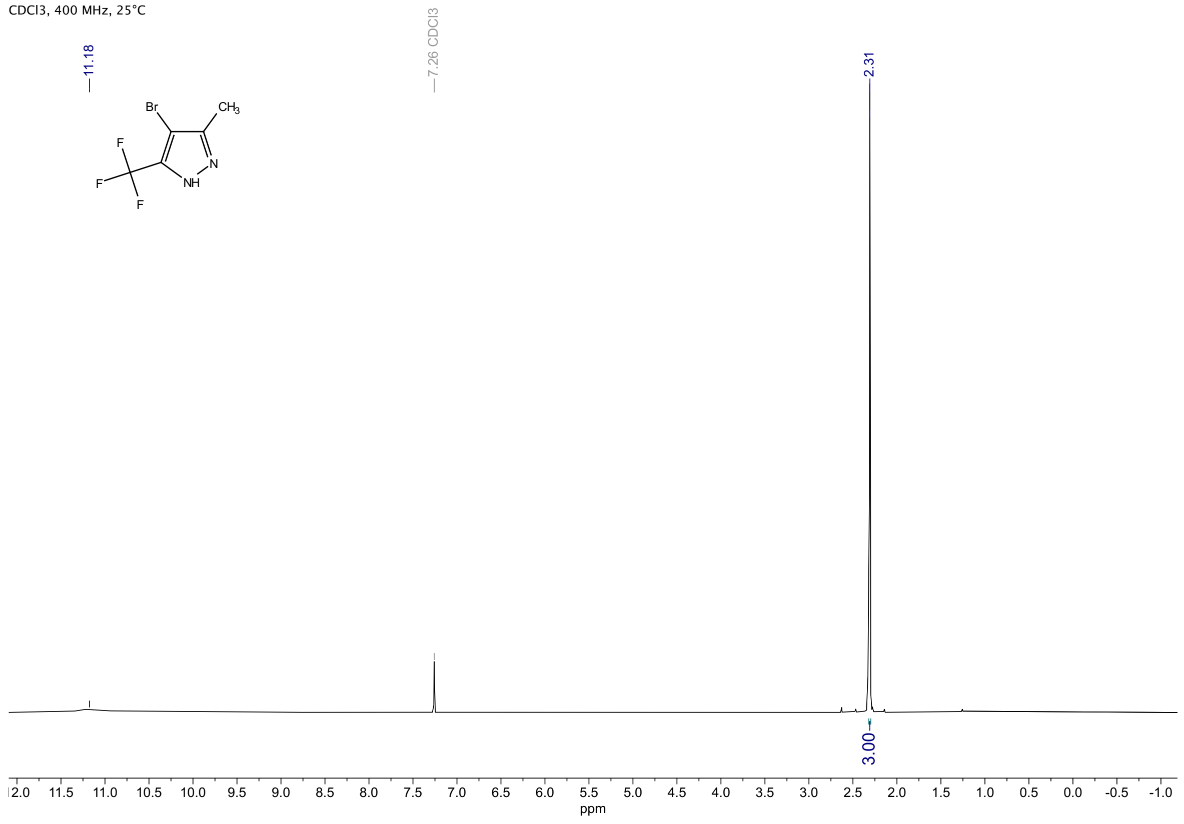

^{13}C NMR of 4-bromo-3-methyl-5-(trifluoromethyl)- *1H*-pyrazole (**6u**)

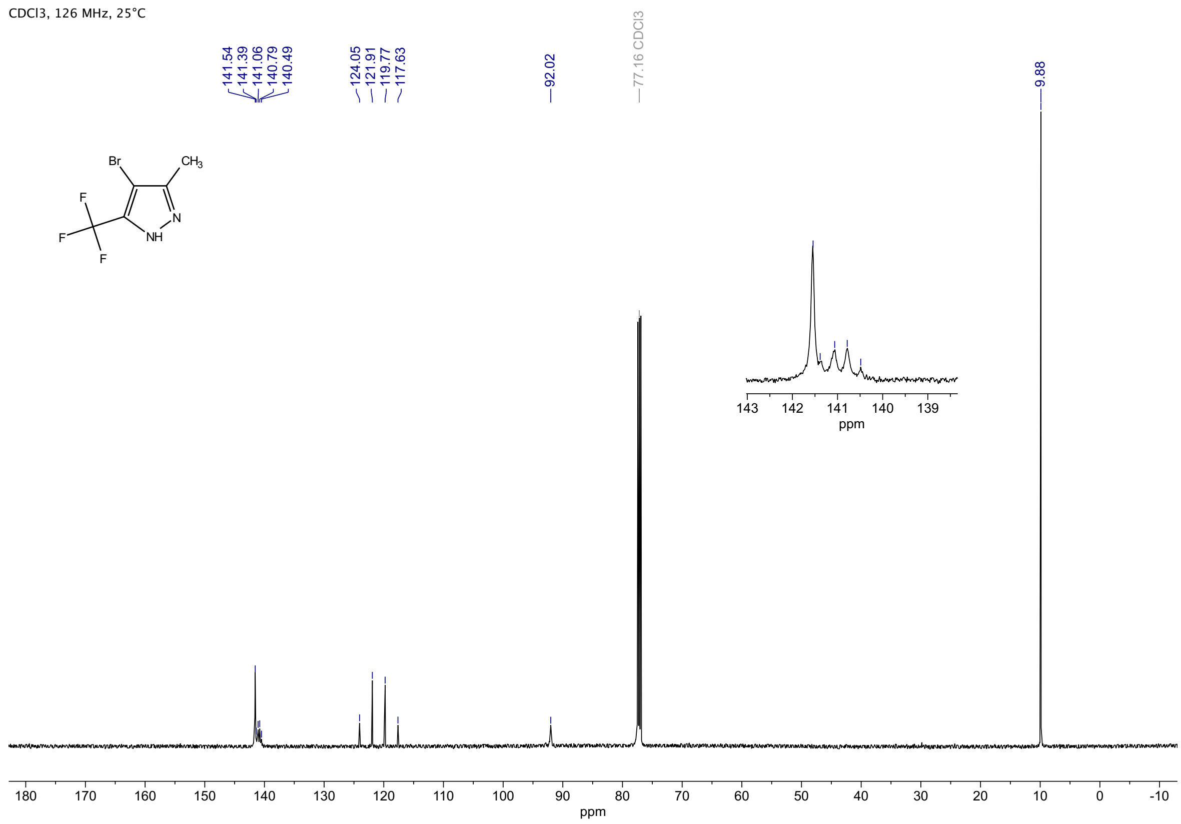

^{19}F NMR of 4-bromo-3-methyl-5-(trifluoromethyl)- *1H*-pyrazole (**6u**)

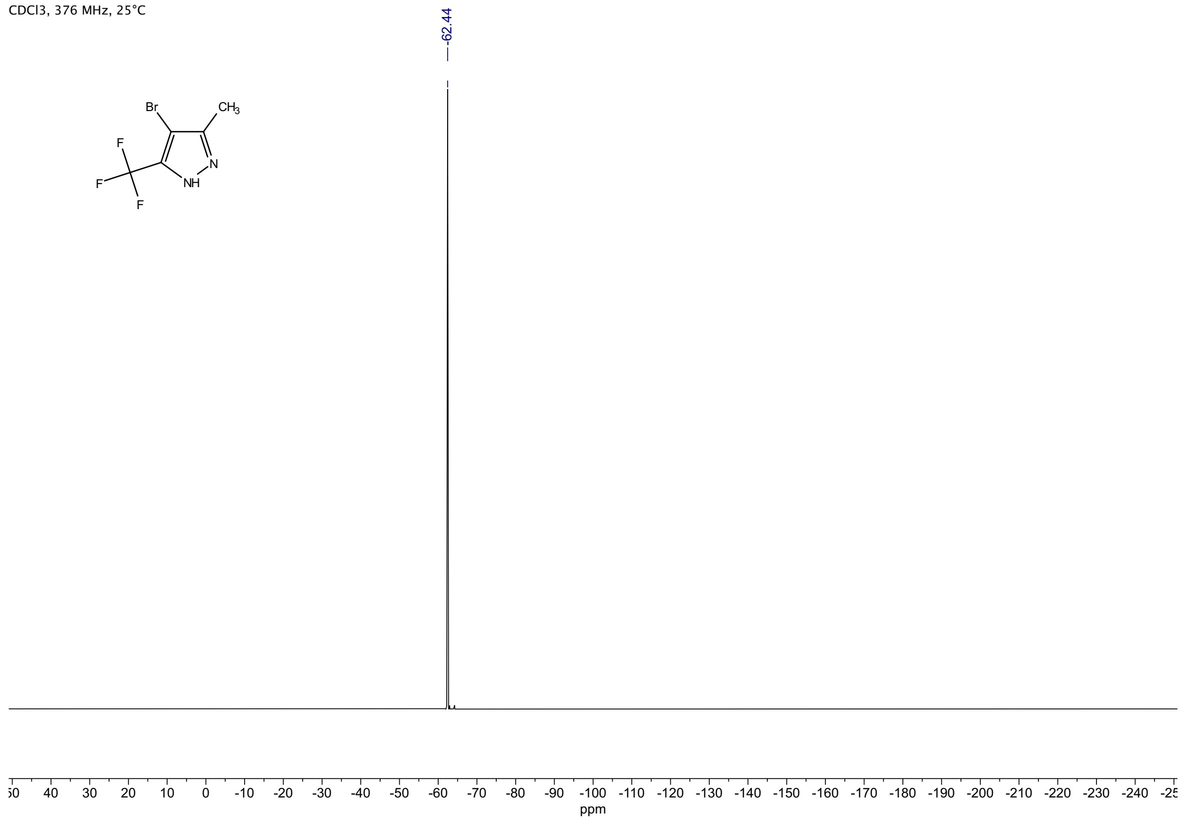

^{1}H NMR of 3-(trifluoromethyl)-2,5,6,7-tetrahydro-*4H*-indazol-4-one (**6v**)

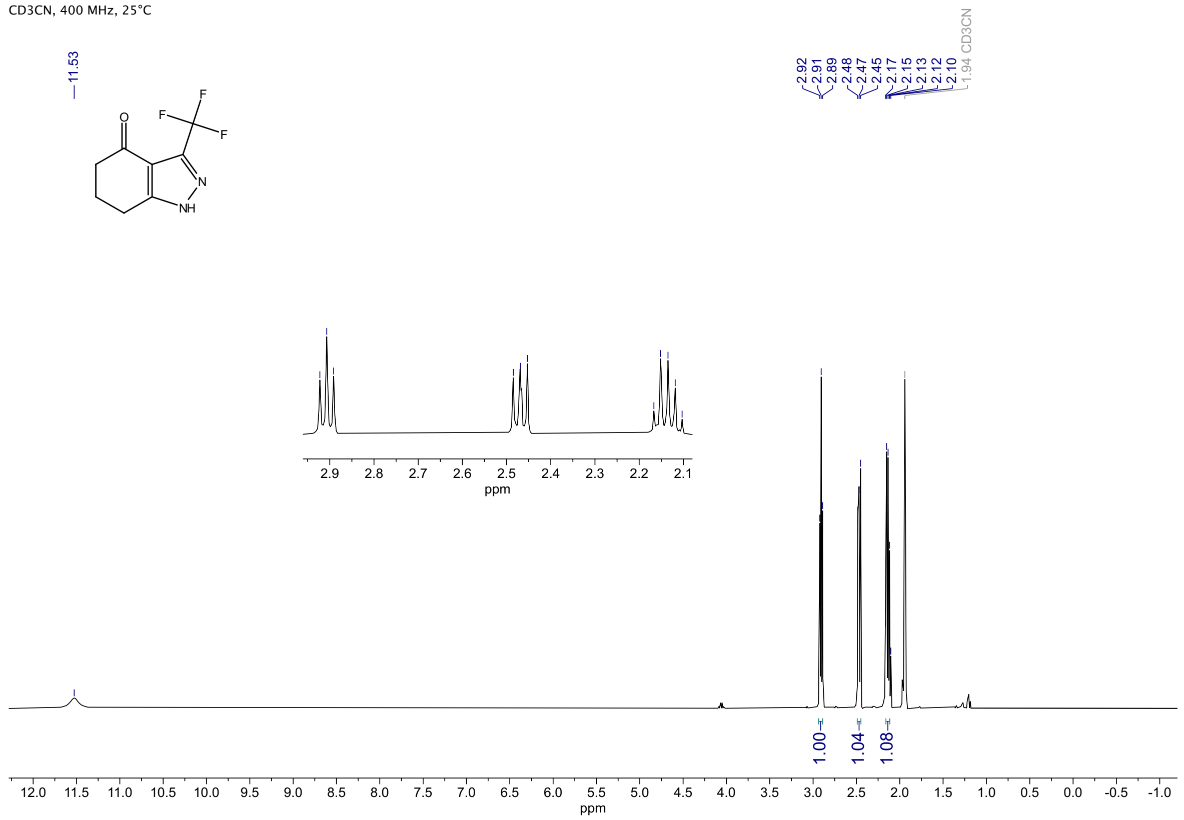

^{13}C NMR of 3-(trifluoromethyl)-2,5,6,7-tetrahydro-*4H*-indazol-4-one (**6v**)

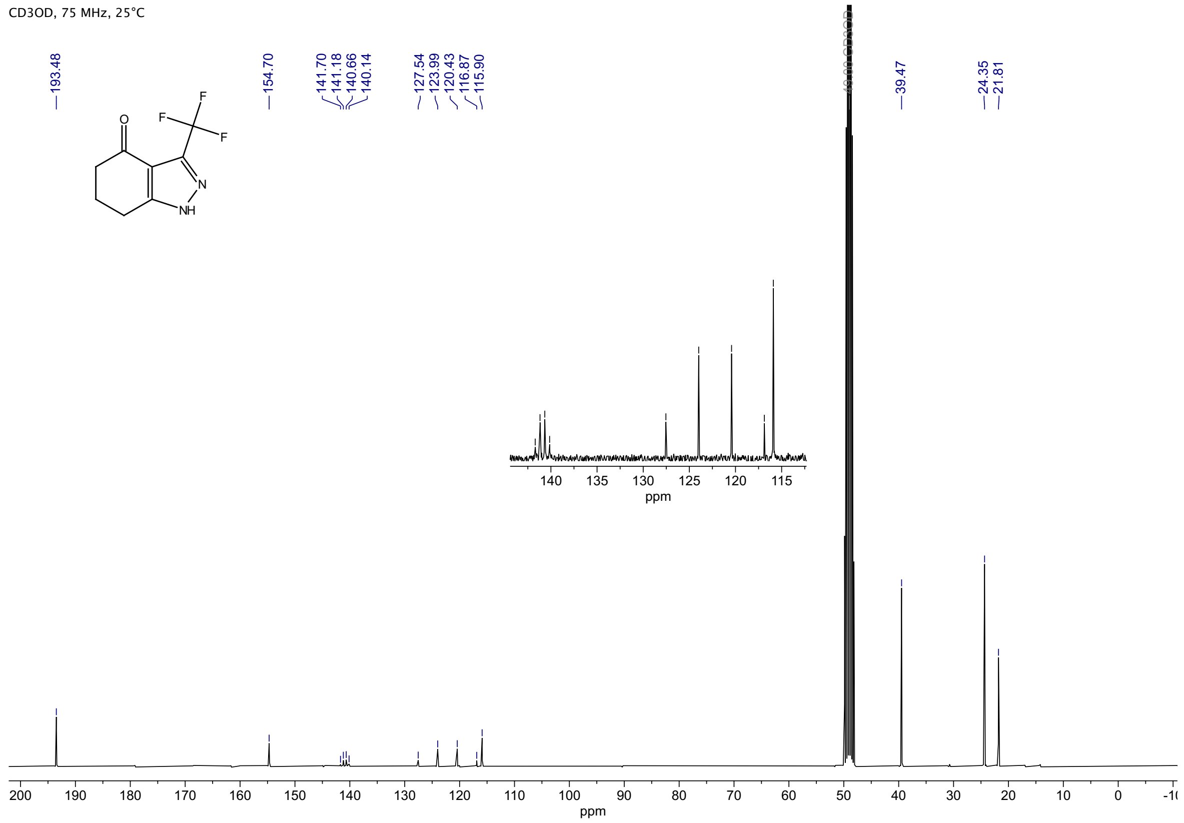

^{19}F NMR of 3-(trifluoromethyl)-2,5,6,7-tetrahydro-*4H*-indazol-4-one (**6v**)

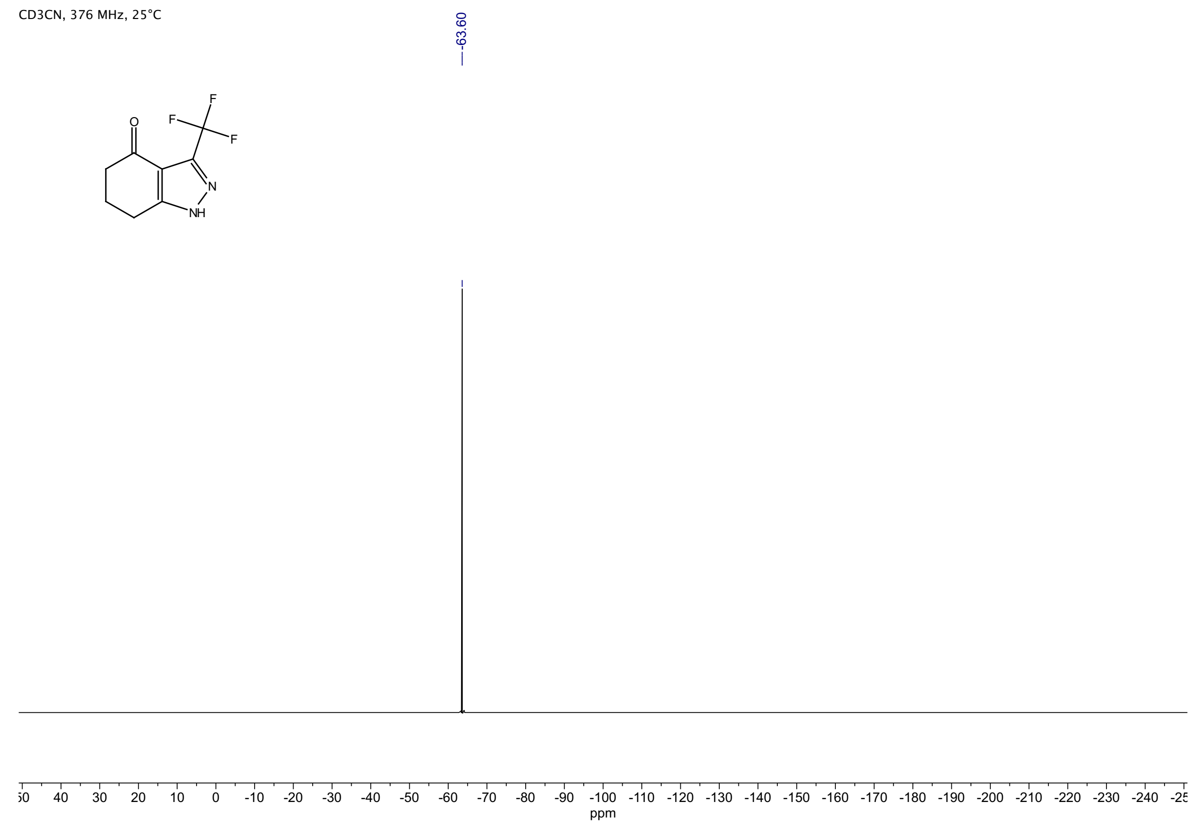

^{1}H NMR of 3-(trifluoromethyl)-1,4,5,6-tetrahydro-*7H*-pyrazolo[3,4-*c*]pyridin-7-one (**6w**)

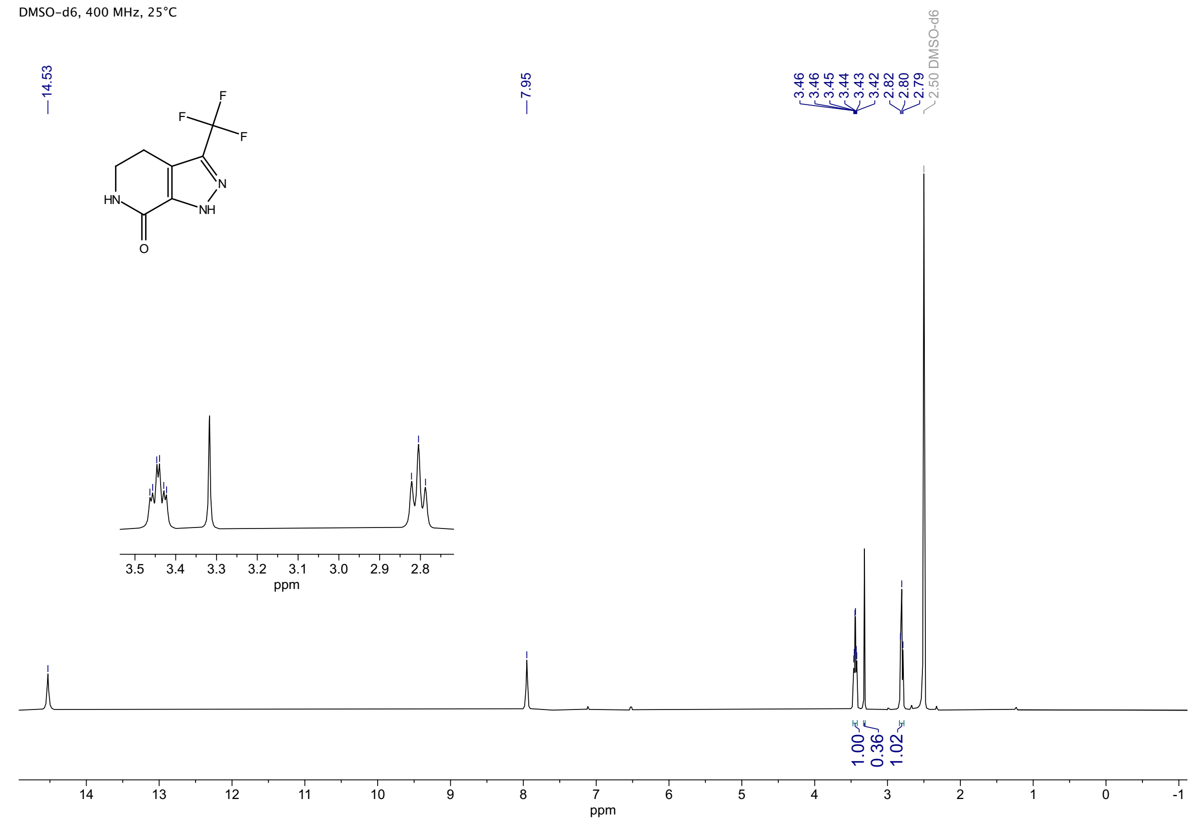

^{13}C NMR of 3-(trifluoromethyl)-1,4,5,6-tetrahydro-*7H*-pyrazolo[3,4-*c*]pyridin-7-one (**6w**)

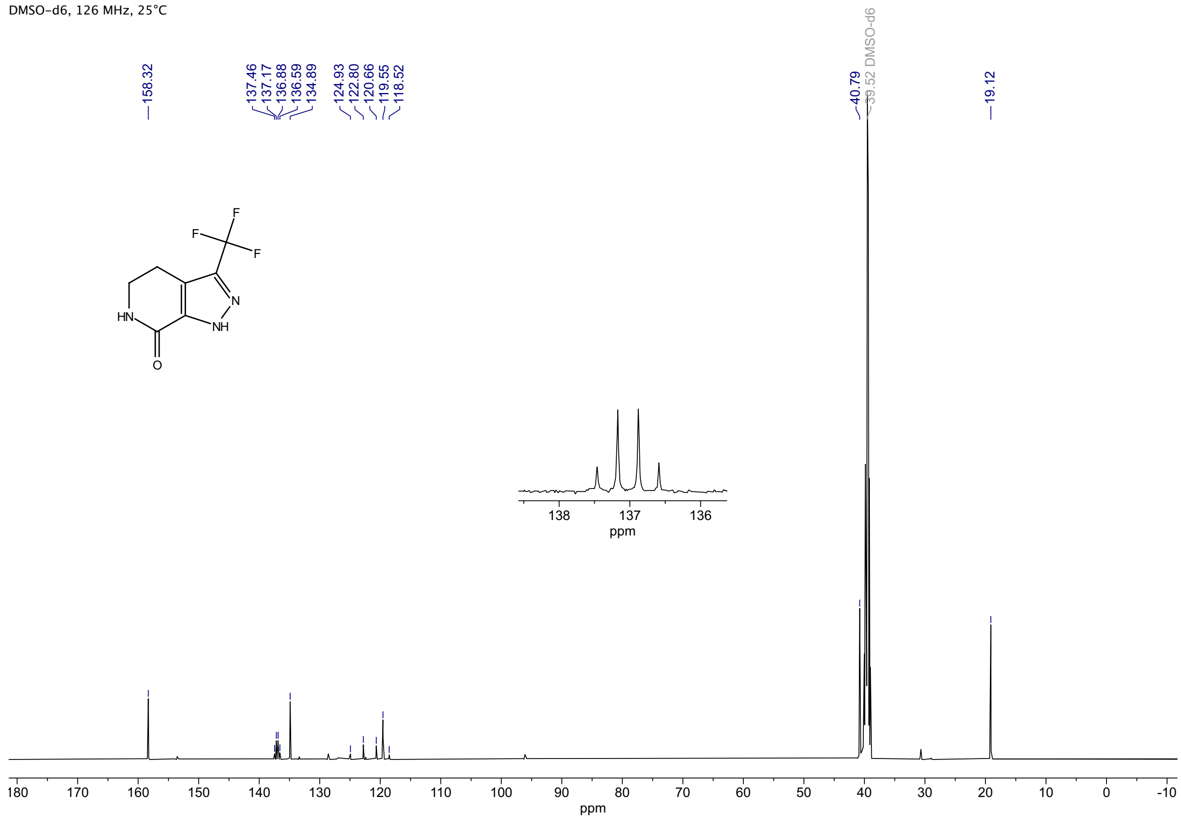

^{19}F NMR of 3-(trifluoromethyl)-1,4,5,6-tetrahydro-*7H*-pyrazolo[3,4-*c*]pyridin-7-one (**6w**)

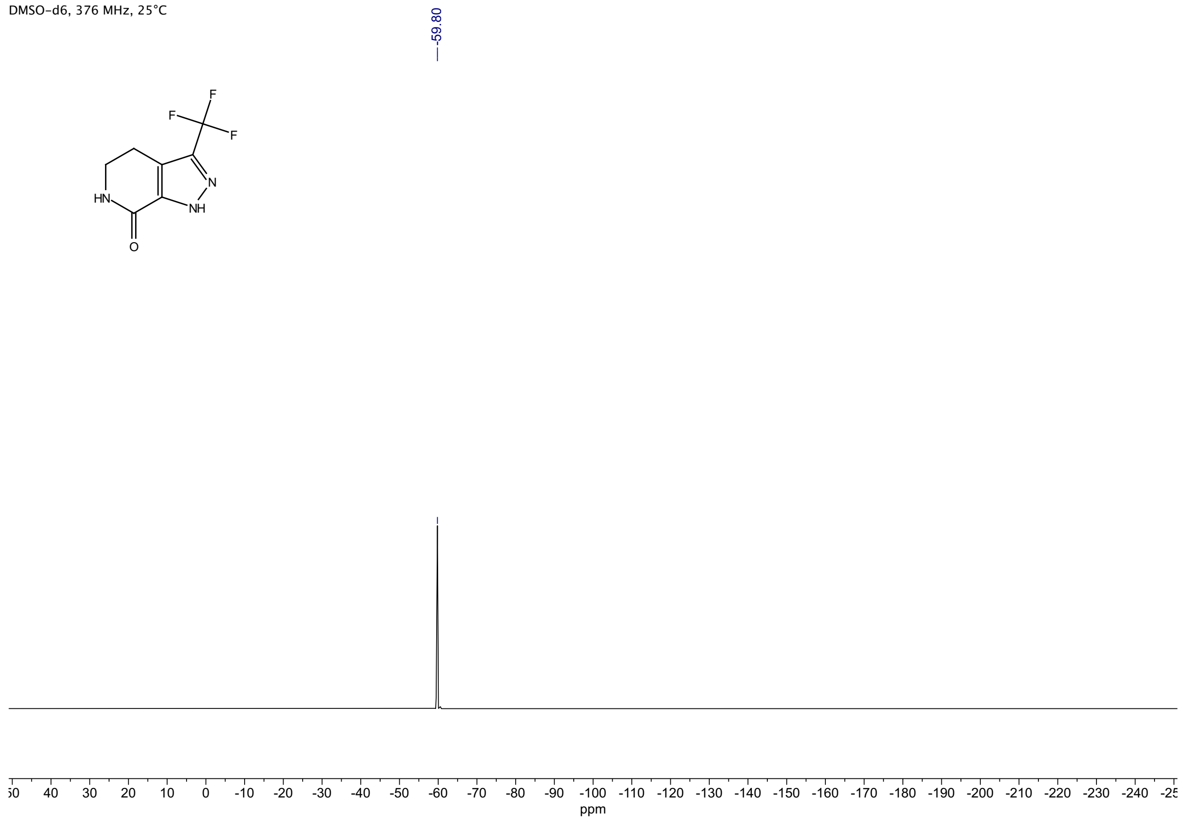

^{1}H NMR of 5-isopropyl-1-methyl-3-(trifluoromethyl)-1,7-dihydro-4*H*-pyrazolo[3,4-*d*]pyrimidine-4,6(5*H*)-dione (**6x**)

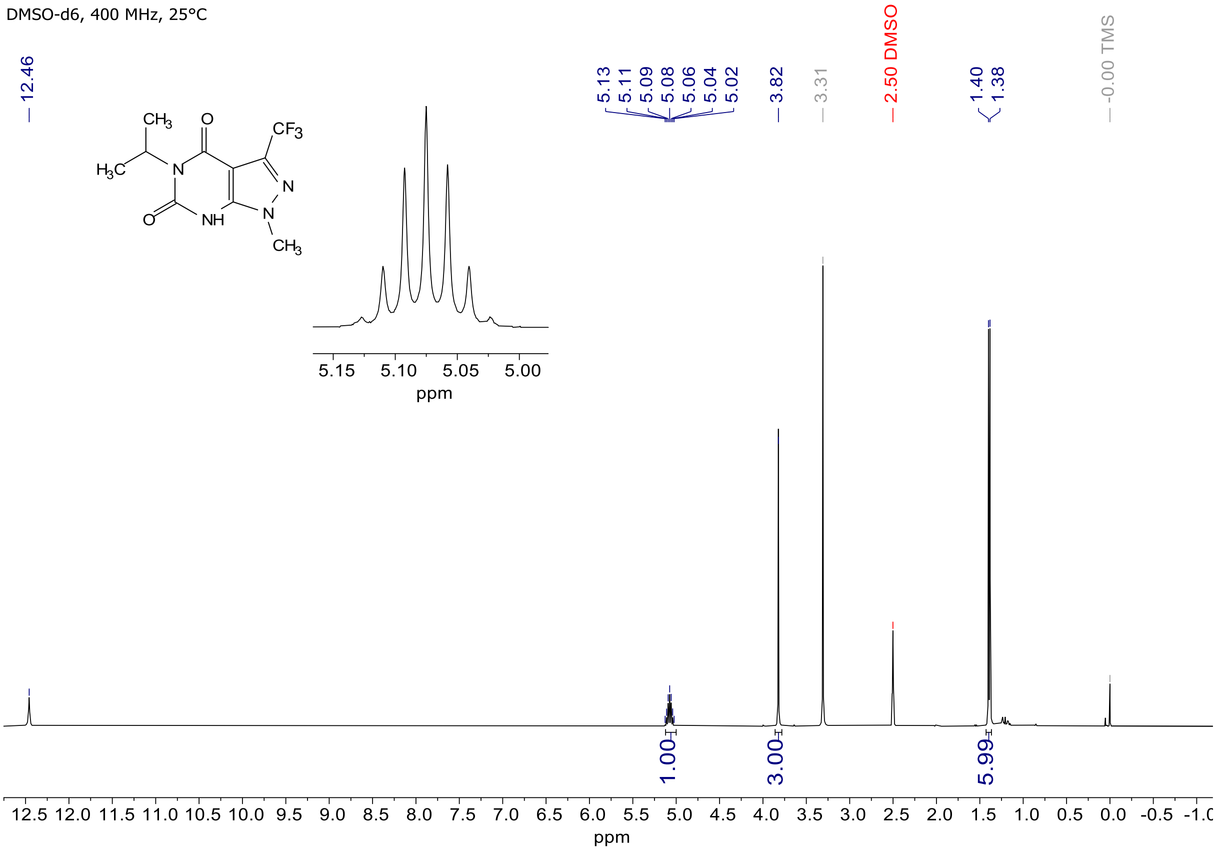

^{13}C NMR of 5-isopropyl-1-methyl-3-(trifluoromethyl)-1,7-dihydro-4*H*-pyrazolo[3,4-*d*]pyrimidine-4,6(5*H*)-dione (**6x**)

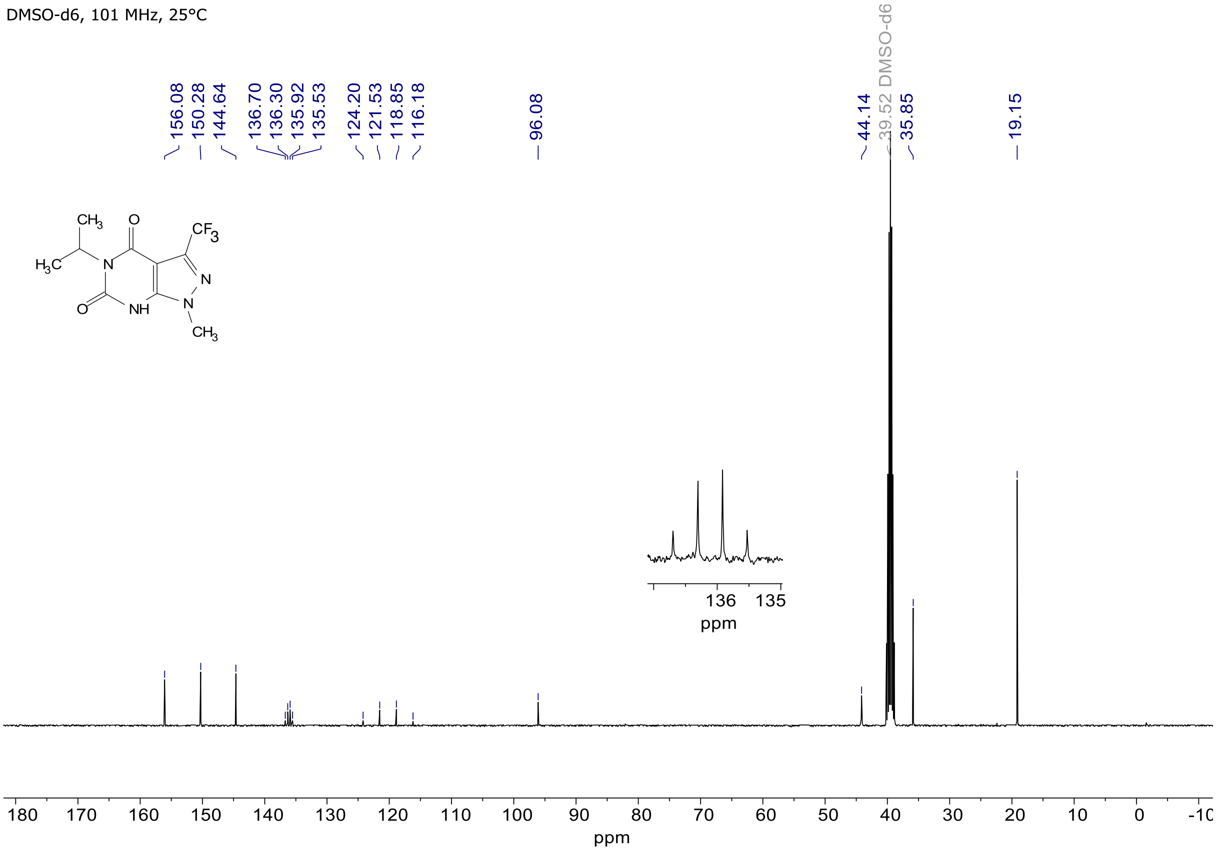

^{19}F NMR of 5-isopropyl-1-methyl-3-(trifluoromethyl)-1,7-dihydro-4*H*-pyrazolo[3,4-*d*]pyrimidine-4,6(5*H*)-dione (**6x**)

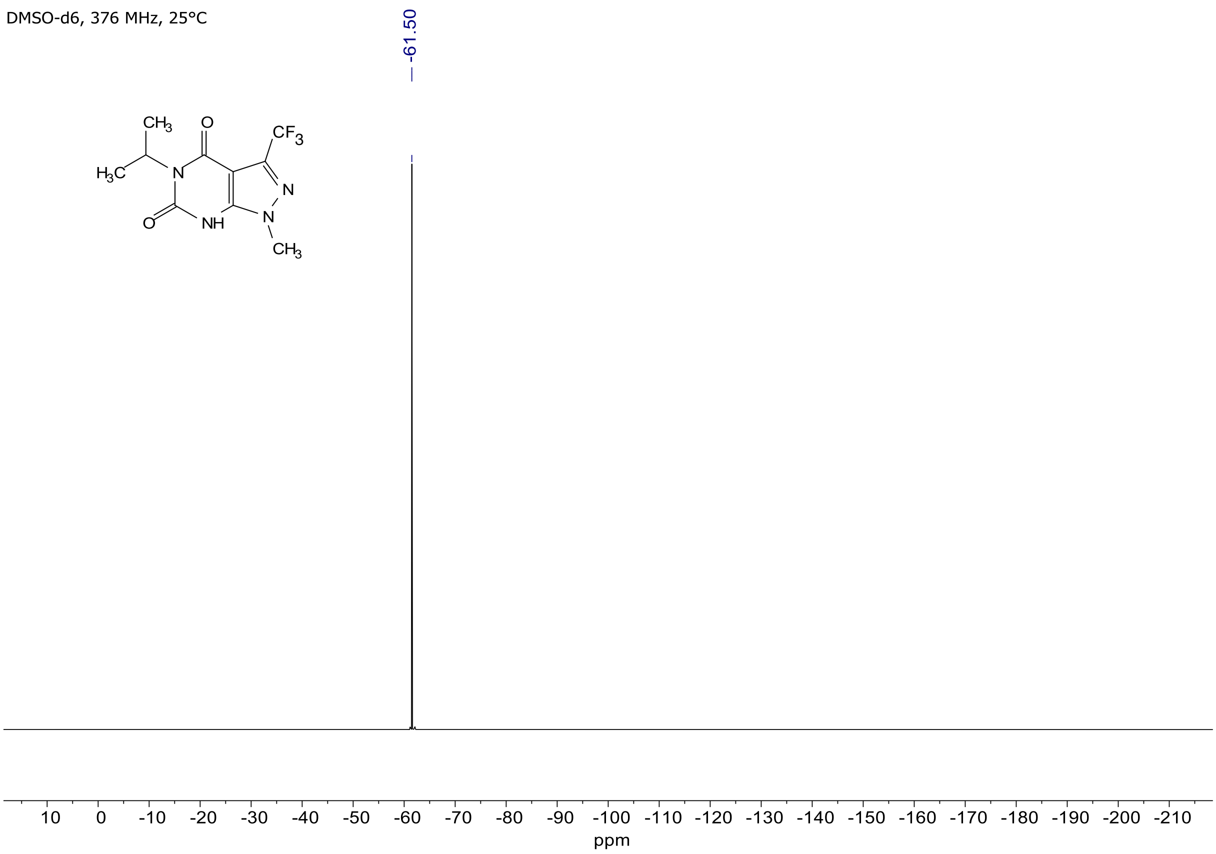

^{13}C NMR of 4,6-dichloro-3-(trifluoromethyl)-*2H*-pyrazolo[3,4-*d*]pyrimidine (**7**)

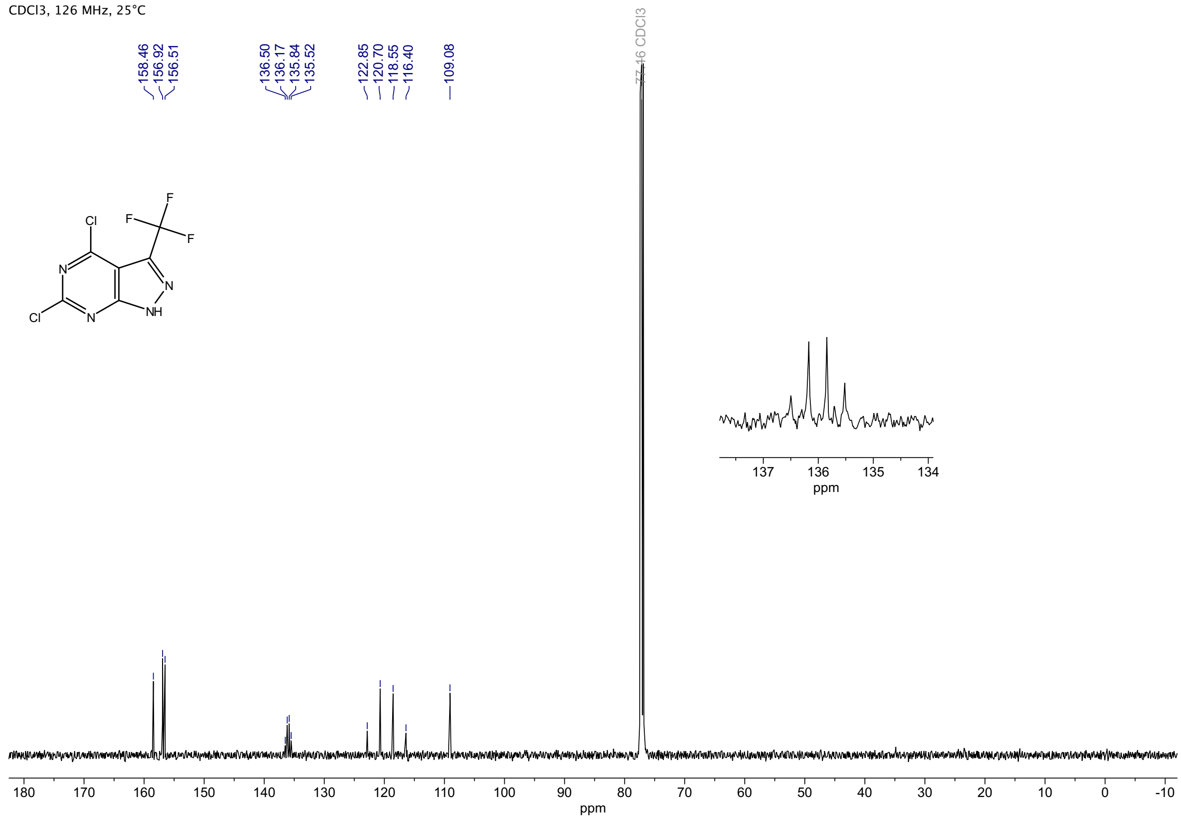

^{19}F NMR of 4,6-dichloro-3-(trifluoromethyl)-*2H*-pyrazolo[3,4-*d*]pyrimidine (**7**)

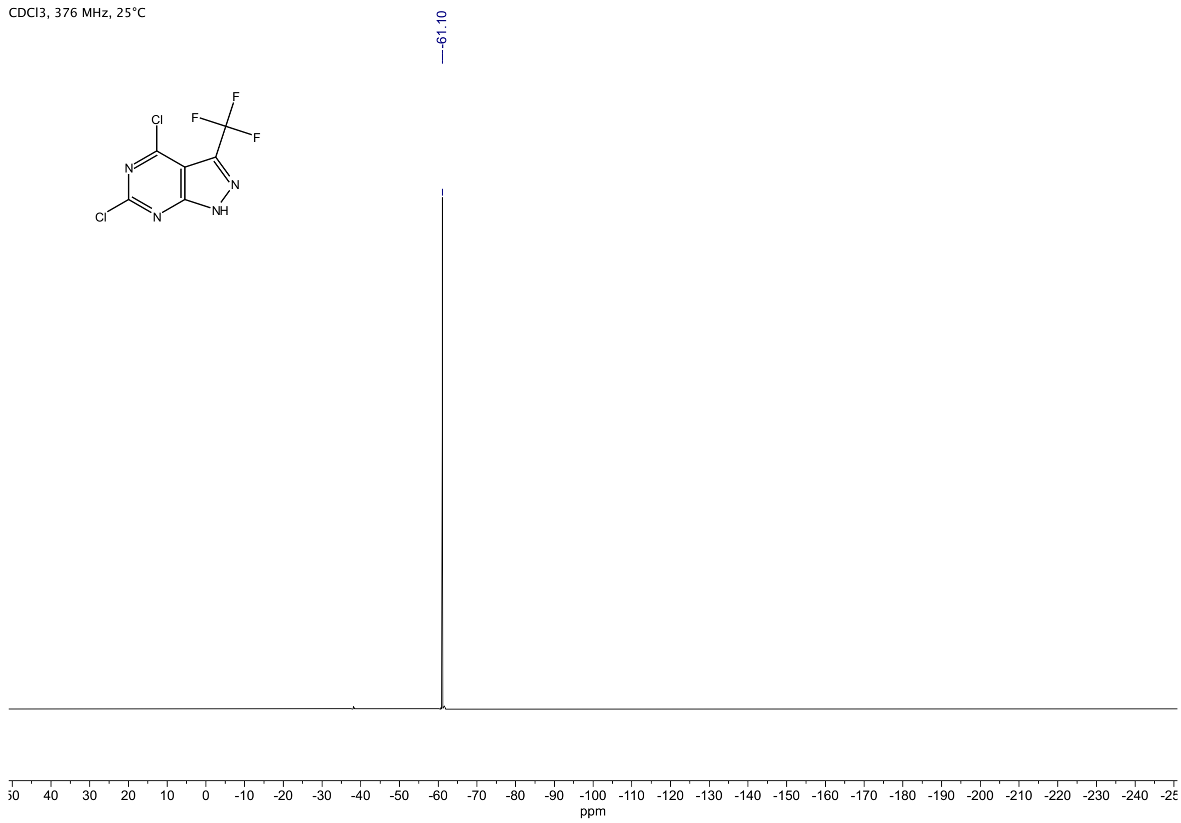

^{1}H NMR of *N*-(5-(trifluoromethyl)-1,3,4-thiadiazol-2-yl)acetamide (**8**)

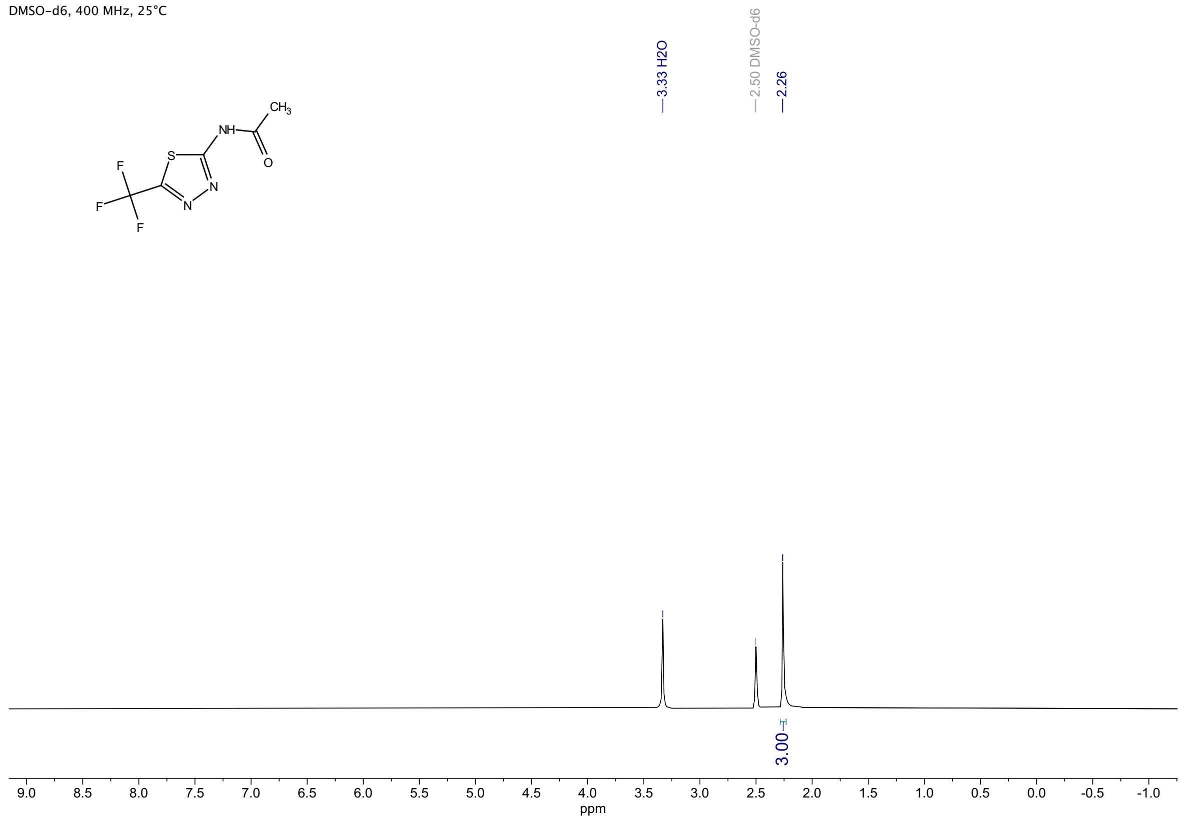

^{13}C NMR of *N*-(5-(trifluoromethyl)-1,3,4-thiadiazol-2-yl)acetamide (**8**)

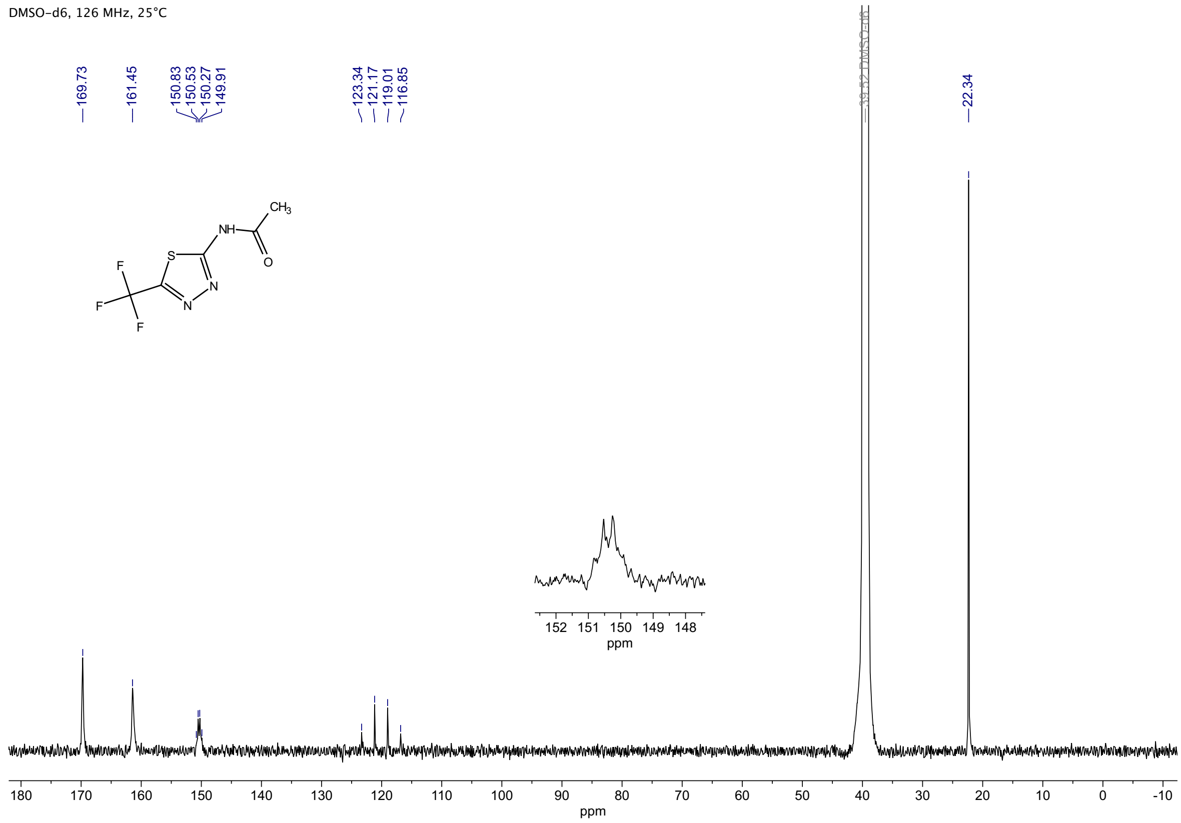

^{19}F NMR of *N*-(5-(trifluoromethyl)-1,3,4-thiadiazol-2-yl)acetamide (**8**)

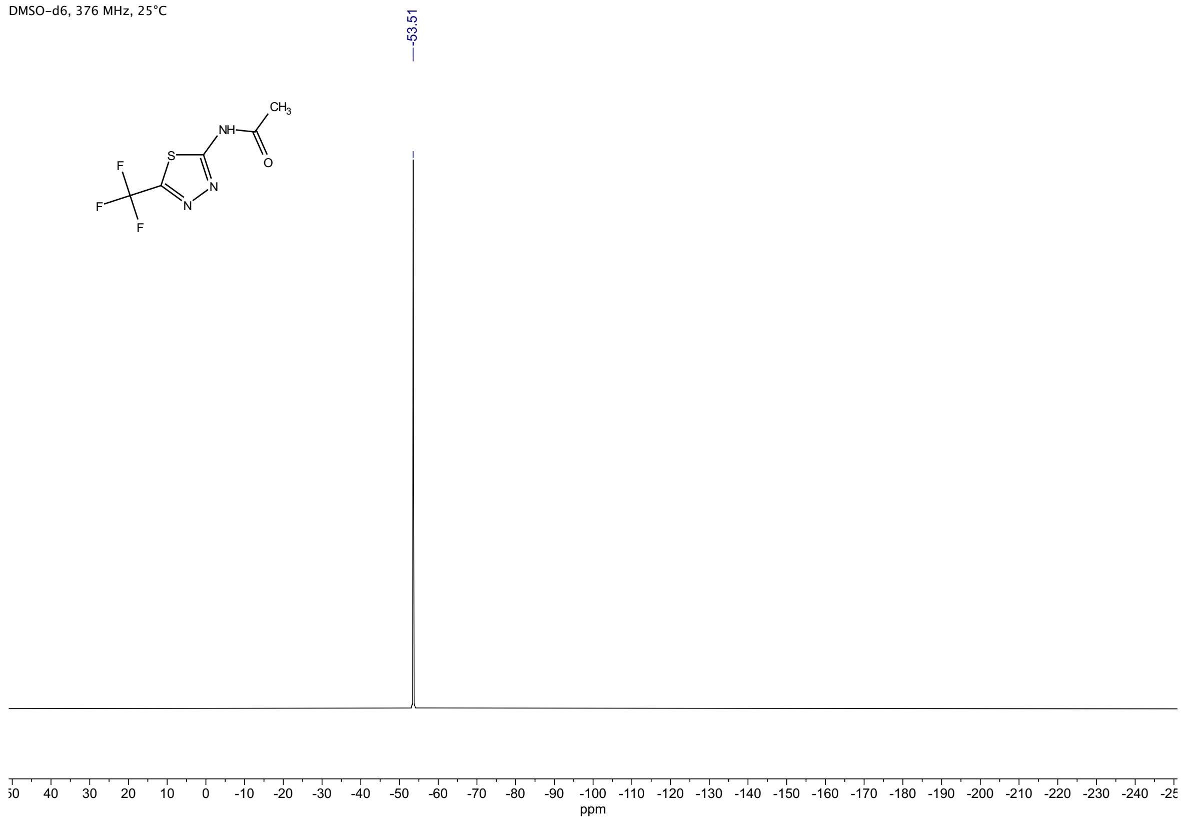

Supplement: Supplementary file 1 [file ao5c12212_si_001.pdf]
